# TRIM40 Drives Pathological Cardiac Hypertrophy and Heart Failure via Ubiquitination of PKN2

**DOI:** 10.1002/advs.202521337

**Published:** 2026-01-22

**Authors:** Risheng Zhao, Xiaoli Cui, Huizhu Du, Zhuoqun Wang, Chang Liu, Linxin Zhang, Jianing Qi, Di Yang, Hui Yu, Shuang Yan, Wei Liu, Haiming Sun, Mengyang Wang

**Affiliations:** ^1^ Department of Pharmacology College of Pharmacy Beihua University Jilin Jilin P. R. China; ^2^ Peking University People's Hospital Qingdao; Women and Children's Hospital Qingdao University Qingdao P. R. China; ^3^ Department of Ultrasonography Integrated Traditional Chinese and Western Medicine Hospital of Jilin City Jilin Province Jilin P. R. China

**Keywords:** angiotensin II, cardiac hypertrophy, heart failure, PKN2, transverse aortic constriction, TRIM40

## Abstract

Pathological cardiac hypertrophy is a major predisposing factor for heart failure (HF). This study investigates the role of the E3 ubiquitin ligase Tripartite Motif‐Containing 40 (TRIM40) in cardiac hypertrophy. Using TRIM40 knockout (TRIM40^−/−^), cardiac‐specific knockdown and overexpressing mice, pathological hypertrophy was induced by angiotensin II (Ang II) infusion or transverse aortic constriction (TAC). Results showed that TRIM40 expression was upregulated in hypertrophic hearts. TRIM40 deficiency attenuated cardiac hypertrophy and dysfunction, whereas its overexpression exacerbated pathological remodeling. Mechanistically, TRIM40 binds PKN2 via its B‐box domain and, in a manner requiring its C29‐dependent E3 ligase activity, promotes K63‐linked ubiquitination of PKN2. This leads to enhanced PKN2 phosphorylation at Ser815 and activation of downstream signaling. Pharmacological inhibition of PKN2 attenuated cardiac remodeling induced by TRIM40 overexpression. These findings reveal that TRIM40 drives cardiac hypertrophy through K63‐linked ubiquitination and activation of PKN2, identifying TRIM40 as a promising candidate for therapeutic intervention in HF.

## Graphical abstract

This study identifies the E3 ligase TRIM40 as a key driver of pathological cardiac hypertrophy. TRIM40 binds PKN2 via its B‐box domain and, through its C29‐dependent catalytic activity, mediates K63‐linked ubiquitination of PKN2. This modification enhances PKN2 phosphorylation at Ser815, thereby driving hypertrophy. Therefore, targeting TRIM40 represents a novel therapeutic strategy for heart failure.

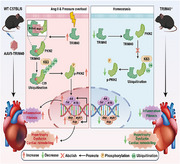

## 1 Introduction

Pathological cardiac hypertrophy is a critical predisposing factor for various cardiovascular diseases, including heart failure (HF), arrhythmia, and sudden cardiac death [1]. It is primarily characterized by an increase in cardiomyocyte size and impaired cardiac contractile function [2]. Under chronic hemodynamic and mechanical stress, the heart initially develops compensatory hypertrophy to maintain normal pump function [3]. However, persistent overload drives the transition from compensatory hypertrophy to a decompensated state, marked by aberrant cardiomyocyte enlargement and dysfunction, ultimately leading to irreversible pathological cardiac remodeling and HF [4]. Cardiomyocyte hypertrophy and functional deterioration are central pathological features during disease progression [5]. Currently, clinical management of pathological cardiac hypertrophy remains limited to ameliorating cardiac function and slowing the progression of HF. The precise molecular mechanisms governing cardiomyocyte hypertrophy are still not fully elucidated, and there is a lack of specific therapeutic agents targeting cardiomyocyte hypertrophy directly. Therefore, there is an urgent need to elucidate the underlying molecular mechanisms driving the initiation and progression of cardiomyocyte hypertrophy, in order to develop more specific and effective therapeutic targets for the diagnosis and treatment of pathological cardiac hypertrophy.

Previous studies have indicated that pathological cardiac hypertrophy is frequently associated with dysregulated protein accumulation and control [6]. The ubiquitin‐proteasome system (UPS) plays a central role in finely modulating protein stability, localization, activity, and interactions [7]. This system comprises multiple key components, such as E1 activating enzymes, E2 conjugating enzymes, E3 ubiquitin ligases, and deubiquitinating enzymes. Of these, E3 ubiquitin ligases display the greatest substrate specificity during ubiquitination by identifying target proteins and mediating the attachment of ubiquitin molecules. This process is essential for maintaining protein homeostasis or modulating diverse biological functions [8]. Conversely, deubiquitination also critically influences the initiation and progression of numerous diseases, including cardiovascular conditions [9, 10], neurological diseases [11, 12], and cancers [13, 14, 15]. A growing body of experimental evidence implicates specific E3 ubiquitin ligases and their corresponding substrates in the development and advancement of pathological cardiac hypertrophy [16, 17, 18]. With more than 600 E3 ubiquitin ligases encoded in the human genome, the roles of the majority in cardiac pathophysiology remain largely unexplored. Further investigation of their functions in cardiac hypertrophy may reveal novel therapeutic avenues for HF.

Tripartite Motif‐Containing 40 (TRIM40) is a member of the TRIM protein family, a highly evolutionarily conserved group encoded within the major histocompatibility complex class I region. It is characterized by the presence of three conserved domains: a RING finger domain, one or two B‐box domains, and a coiled‐coil region. Predominantly localized in the cytoplasm, TRIM40 functions as an E3 ubiquitin ligase that mediates the targeted degradation of substrate proteins via the UPS, thereby modulating multiple signaling pathways [19]. It has been reported that TRIM40 binds to MDA5 and RIG‐I, which promotes their polymerization and accelerates their degradation, accordingly, limiting antiviral immune responses and maintaining immune homeostasis [20]. TRIM40 is highly expressed in normal gastrointestinal epithelium, where it normally inhibits local NF‐κB activity to mitigate the occurrence of chronic inflammation [21, 22]. Currently, research on TRIM40 has primarily focused on its roles in cancer [21, 23] and viral immunity [24, 25] in mammals, while its function in cardiac pathophysiology, particularly in hypertrophy, remains completely unknown.

Given the lack of knowledge regarding TRIM40's function in the heart, we sought to investigate its role in pathological cardiac hypertrophy. Using models of angiotensin II (Ang II) infusion and pressure overload, we examined whether TRIM40 expression is altered and assessed its functional impact on cardiac remodeling. Furthermore, we explored the molecular mechanism by which TRIM40 might regulate hypertrophy, with a particular focus on its potential interaction with and ubiquitination of Protein kinase N2 (PKN2). Our results establish TRIM40 as a novel promoter of pathological cardiac hypertrophy and delineate a previously unrecognized TRIM40‐PKN2 signaling axis.

## 2 Results

### 2.1 TRIM40 is Upregulated in Heart Tissues of Mice Challenged with Ang II

**FIGURE 1:**
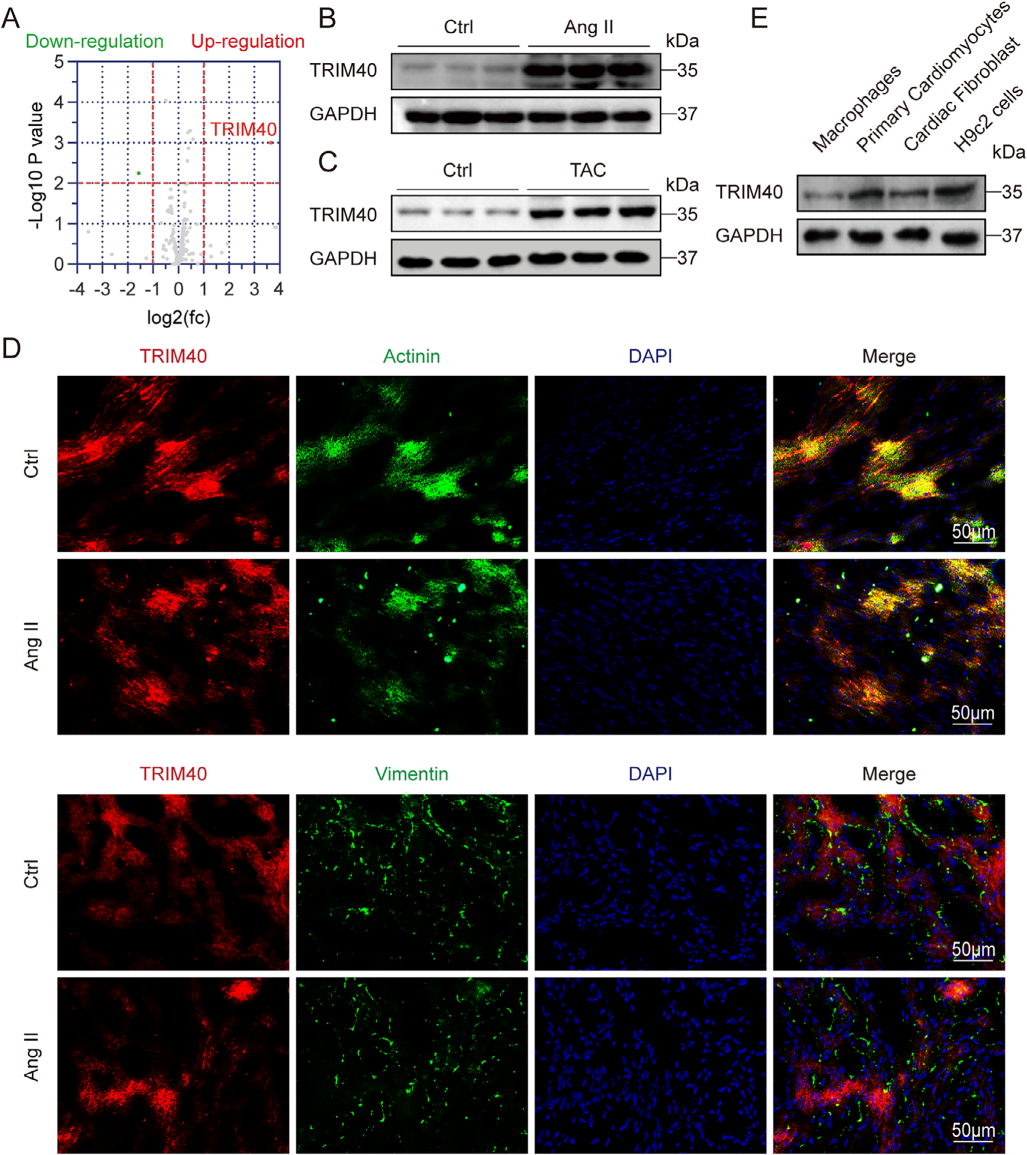
TRIM40 is upregulated in heart tissues of mice challenged with Ang II. (A) RNA sequencing was performed on mouse cardiac tissues to analyze the expression profile of E3 ubiquitin ligases (red indicates up‐regulated genes, green indicates down‐regulated genes). Gray dots represent E3 ubiquitin ligases with no statistically significant difference compared to the control group. A fold change > 2 and *p* < 0.05 were considered statistically significant. (B) Representative western blot analysis of TRIM40 protein levels in cardiac tissues from Ctrl and Ang II‐induced mice. Glyceraldehyde‐3 phosphate dehydrogenase (GAPDH) was used as a loading control (n = 6). (C) Representative western blot analysis of TRIM40 protein levels in cardiac tissues from Ctrl and transverse aortic constriction (TAC)‐induced mice. GAPDH was used as a loading control (n = 6). (D) Immunofluorescence staining of mouse cardiac tissues showing the localization of TRIM40 (red), vimentin (green), and sarcomeric α‐actinin (green). Nuclei were counterstained with 4’,6‐Diamidino‐2’‐phenylindole (DAPI) (blue). Scale bar = 50 µm (n = 6). (E) Representative western blot analysis of TRIM40 protein levels in macrophages, NRVMs, NRCFs, and H9c2 cells. GAPDH was used as a loading control (n = 3).

We initially sought to profile alterations in E3 ubiquitin ligase expression during cardiac hypertrophy and remodeling. To this end, we employed a well‐established mouse model involving continuous infusion of Ang II at a dose of 1 µg/kg/min. RNA‐sequencing analysis of heart tissues from mice treated with Ang II for 4 weeks revealed significant changes in the expression of multiple E3 ubiquitin ligases (Figure 1A; Figure S1A and Table S1). Strikingly, TRIM40 expression was consistently and markedly upregulated in cardiac tissues of Ang II‐infused mice. Functional enrichment analysis (GO analysis) of the differentially expressed genes further revealed their significant enrichment in biological processes related to inflammatory responses, among others (Figure S1B). This increase in TRIM40 expression was further confirmed at the protein level by immunoblotting of heart tissue lysates (Figure 1B,C; Figure S1C,D). To identify the cellular origin of elevated TRIM40 in the heart, we performed co‐staining of tissue sections for TRIM40, α‐actinin (a cardiomyocyte marker), and vimentin (a fibroblast marker). Our data indicated that the increase in TRIM40 following Ang II stimulation primarily originated from cardiomyocytes (Figure 1D; Figure S1E), as evidenced by the predominant immunoreactivity of TRIM40 in α‐actinin‐positive but vimentin‐negative cells. We further validated these findings in isolated primary cells from wild type (WT) mice, including primary macrophages, neonatal rat ventricular myocytes (NRVMs), and neonatal rat cardiac fibrosis (NRCFs). Immunoblot analysis demonstrated high expression levels of TRIM40 in both primary cardiomyocytes and H9c2 cardiomyocyte‐like cells (Figure 1E; Figure S1F). Collectively, these data demonstrate that TRIM40 is upregulated in cardiomyocytes upon Ang II challenge, implicating it as a candidate regulator of pathological cardiac remodeling.

### 2.2 TRIM40 Deficiency Attenuates Ang II‐Induced Pathological Hypertrophy and Dysfunction in Mice

**FIGURE 2:**
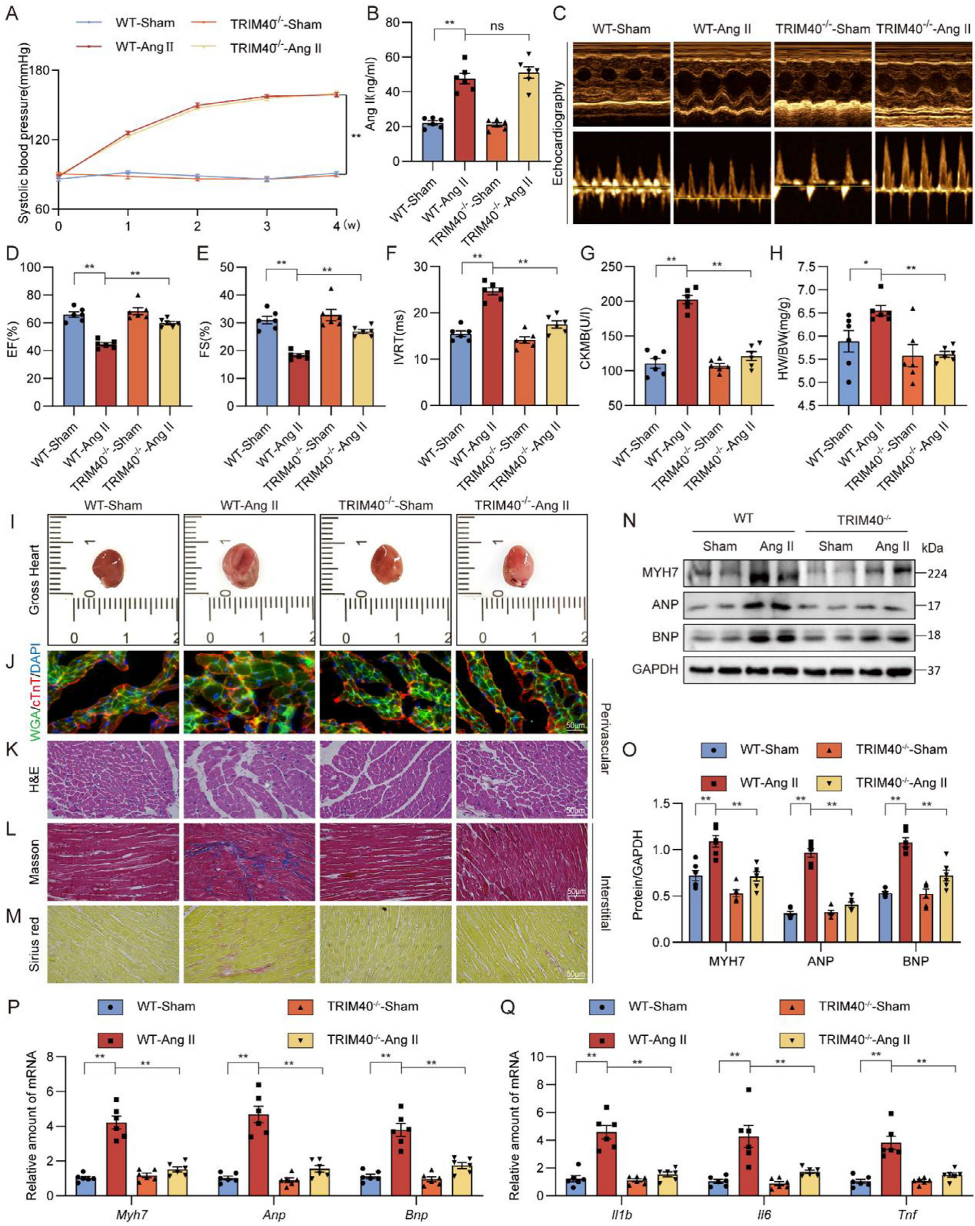
TRIM40 deficiency protects against Ang II‐induced myocardial hypertrophy and fibrosis. WT and TRIM40^−/−^ mice were infused with saline or Ang II for 4 weeks. (A) Mouse systolic blood pressure was measured weekly using a non‐invasive tail‐cuff method (n = 6). (B) Serum Ang II concentration was detected in mice (n = 6). (C) Representative echocardiographic images of mice from each group (n = 6). (D–F) Cardiac function parameters showing EF (D), FS (E), and IVRT (F) (n = 6). (G) Serum CK‐MB levels were measured using an ELISA kit (n = 6). (H) HW/BW of mice in each group (n = 6). (I) Representative freshly isolated heart specimens photographed against a white background (scale bar = 5 mm) (n = 6). (J) Cardiomyocyte cross‐sectional area was assessed by dual immunofluorescence staining for cTnT (red) to mark cardiomyocytes and fluorescein‐labeled WGA (green) to delineate cell membranes (scale bar = 50 µm) (n = 6). (K) H&E staining of heart tissue sections (scale bar = 50 µm) (n = 6). (L, M) Myocardial fibrosis was evaluated by Masson's trichrome staining (L) and Picrosirius red staining (M) (scale bar = 50 µm) (n = 6). (N) Representative western blot analyses of MYH7, ANP, and BNP in heart tissue, with GAPDH as a loading control (n = 6). (O) Densitometric quantification of the blots in N (n = 6). (P, Q) mRNA expression levels of hypertrophy‐associated genes (P) and inflammation‐related genes (Q) in heart tissues of mice. Data was normalized to *Actb* (n = 6). All quantitative data are presented as mean ± SEM. Data between two groups were compared by independent‐sample two‐tailed Student's t‐test. Data among multiple groups were compared by one‐way ANOVA test, followed by Tukey post hoc test. ns indicates not statistically significant; *p < 0.05, **p < 0.01.

To investigate the role of TRIM40 in cardiac dysfunction, we utilized a TRIM40^−/−^ mouse model (Figure S2A). WT and TRIM40^−/−^ mice were continuously infused with either saline or Ang II for 4 weeks (Figure S2B). The results showed that compared to the saline group, both WT and TRIM40^−/−^ mice treated with Ang II exhibited elevated systolic blood pressure (Figure 2A) and increased plasma Ang II levels (Figure 2B), indicating that TRIM40 deficiency does not affect the systemic pressor response to Ang II. And echocardiographic analysis revealed that TRIM40^−/−^ significantly alleviated Ang II‐induced cardiac dysfunction, specifically manifested as evidenced by preserved ejection fraction (EF) and fractional shortening (FS), along with ameliorated prolongation of the isovolumic relaxation time (IVRT) (Figure 2C–F; Table S2). Moreover, Serum level of creatine kinase‐MB (CK‐MB) was increased in wildtype mice infused with Ang II but not TRIM40 knockout mice (Figure 2G). While the heart weight/body weight (HW/BW) was markedly elevated in Ang II‐treated WT mice, these parameters remained stable in TRIM40^−/−^ mice (Figure 2H; Table S2).

Gross examination of heart tissues indicated a protective effect of TRIM40^−/−^ against Ang II‐induced cardiac hypertrophy (Figure 2I; Figure S2C). Histological analysis using hematoxylin and eosin (H&E) and dual immunofluorescence staining for wheat germ agglutinin (WGA) and cardiac troponin T (cTnT) further confirmed that Ang II stimulation led to significant hypertrophic changes and structural abnormalities in the hearts of WT mice (Figure 2J,K; Figure S2D,E), whereas these pathological responses were markedly attenuated in TRIM40^−/−^ mice. Similarly, Picro Sirius Red and Masson's trichrome staining demonstrated that the degree of cardiac fibrosis was significantly reduced in TRIM40^−/−^ mice compared to the WT control group (Figure 2L,M; Figure S2F–K). At the molecular level, consistent with the above findings, the expression levels of hypertrophy‐related markers‐including myosin heavy chain 7 (MYH7), atrial natriuretic peptide (ANP), and brain natriuretic peptide (BNP)‐in the hearts of TRIM40^−/−^ mice after Ang II infusion were significantly lower than those in WT mice (Figure 2N,O). Concurrently, the mRNA levels of hypertrophy‐ and inflammation‐related genes were significantly upregulated in the hearts of WT mice following Ang II stimulation, but this phenomenon was not observed in TRIM40^−/−^ mice (Figure 2P,Q). In summary, these findings demonstrate that genetic ablation of TRIM40 attenuates Ang II‐induced cardiac hypertrophy, fibrosis, and inflammatory activation.

### 2.3 TRIM40 Knockout Alleviates TAC‐Induced Cardiac Remodeling and Dysfunction in Mice

**FIGURE 3:**
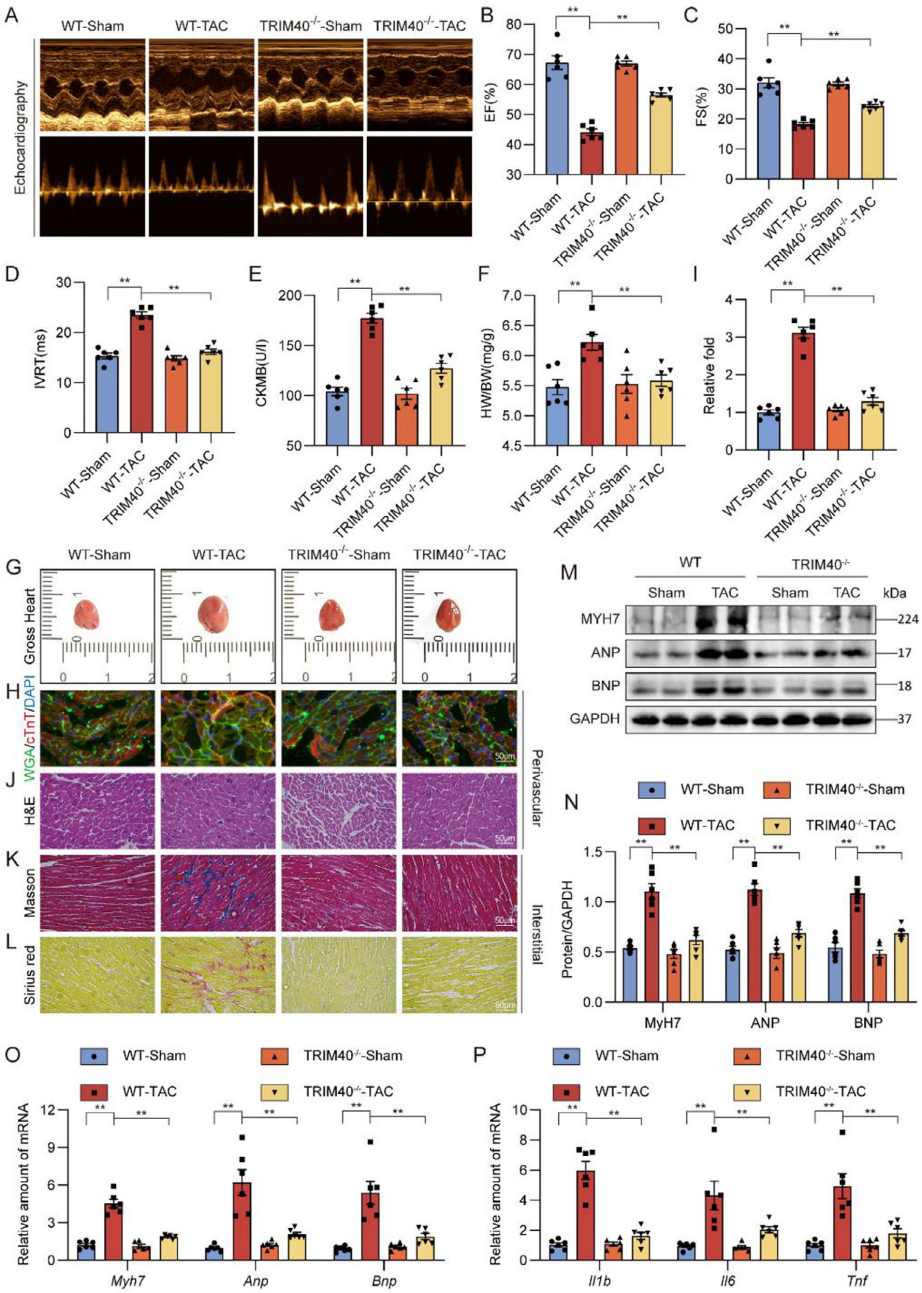
TRIM40 deficiency prevents TAC‐induced myocardial hypertrophy and fibrosis. WT and TRIM40^−/−^ mice were subjected to TAC to model pressure overload‐induced cardiac hypertrophy and remodeling. (A) Representative echocardiographic images of mice from each group (n = 6). (B–D) Cardiac function parameters showing EF (B), FS (C), and IVRT (D) (n = 6). (E) Serum CK‐MB levels were measured using an ELISA kit (n = 6). (F) HW/BW of mice in each group (n = 6). (G) Representative freshly isolated heart specimens photographed against a white background (scale bar = 5 mm) (n = 6). (H) Cardiomyocyte cross‐sectional area was assessed by dual immunofluorescence staining for cTnT (red) to mark cardiomyocytes and fluorescein‐labeled WGA (green) to delineate cell membranes (scale bar = 50 µm) (n = 6). (I) Quantitative analysis of cardiomyocyte cross‐sectional area. A minimum of 100 cells were measured from different fields across at least four samples per group (n = 6). (J) H&E staining of heart tissue sections (scale bar = 50 µm) (n = 6). (K, L) Myocardial fibrosis was evaluated by Masson's trichrome staining (K) and Picrosirius red staining (L) (scale bar = 50 µm) (n = 6). (M) Representative western blot analyses of MYH7, ANP, and BNP in heart tissue, with GAPDH as a loading control (n = 6). (N) Densitometric quantification of the blots in M (n = 6). (O, P) mRNA expression levels of hypertrophy‐associated genes (O) and inflammation‐related genes (P) in heart tissues. Data were normalized to *Actb* (n = 6). All quantitative data are presented as mean ± SEM. Data between two groups were compared by independent‐sample two‐tailed Student's t‐test. Data among multiple groups were compared by one‐way ANOVA test, followed by Tukey post hoc test; **p < 0.01.

Building upon these findings, we further investigated the role of TRIM40 using a TAC model to study its function in pressure overload‐induced cardiac hypertrophy (Figure S3A). TAC surgery was performed on WT and TRIM40^−/−^ mice, with cardiac phenotypes evaluated 4 weeks post‐surgery. Similar to our studies using Ang II‐challenged mice, TRIM40 deficiency attenuated against cardiac dysfunction in TAC mice (Figure 3A–D; Table S3). Relative to sham controls, WT‐TAC mice showed significant increases in serum CK‐MB level, while these increases were normalized in TRIM40^−/−^‐TAC mice (Figure 3E). Furthermore, TRIM40^−/−^ prevented the TAC‐induced elevation in HW/BW (Figure 3F). Genetic deletion of TRIM40 also resulted in a significant reduction of TAC model mice (Figure 3G–O; Figure S3B–I). Similar changing profiles were observed when we examined inflammatory markers (Figure 3P). Taken together, these data establish that TRIM40 deficiency mitigates pathological cardiac remodeling induced by pressure overload.

### 2.4 Cardiomyocyte‐Specific Knockdown of TRIM40 Alleviates Ang II‐Induced Cardiac Remodeling

**FIGURE 4:**
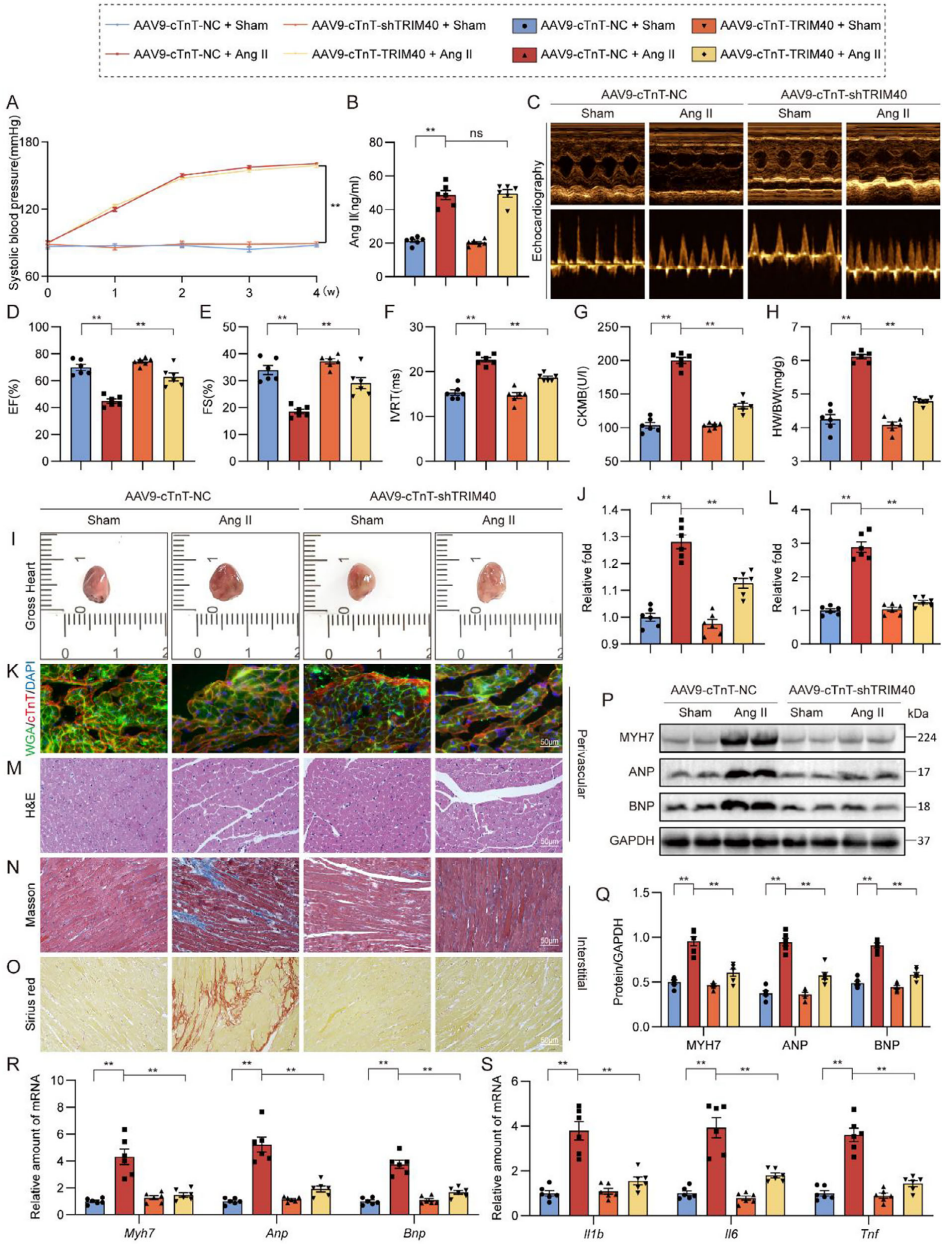
Cardiomyocyte‐specific knockdown of TRIM40 alleviates Ang II‐induced cardiac remodeling. (A) Mouse systolic blood pressure was measured weekly using a non‐invasive tail‐cuff method (n = 6). (B) Serum Ang II concentration was detected in mice (n = 6). (C) Representative echocardiographic images from each group of mice (n = 6). (D‐F) Cardiac function parameters: EF (D), FS (E), and IVRT (F) (n = 6). (G) Serum CK‐MB levels (n = 6). (H) Heart weight/body weight ratio (n = 6). (I) Representative images of freshly isolated heart specimens (n = 6). (J) Quantitative analysis of the cross‐sectional area of heart specimens shown in (I) (n = 6). (K) Cardiomyocyte cross‐sectional area was assessed by dual immunofluorescence staining for cTnT (red) to mark cardiomyocytes and fluorescein‐labeled WGA (green) to delineate cell membranes (scale bar = 50 µm) (n = 6). (L) Quantitative analysis of cardiomyocyte cross‐sectional area based on K. A minimum of 100 cells were measured from different fields across at least four samples per group (n = 6). (M) H&E staining of heart tissue sections (scale bar = 50 µm) (n = 6). (N, O) Myocardial fibrosis evaluated by Picrosirius Red (N) and Masson's trichrome (O) staining (scale bar = 50 µm) (n = 6). (P) Representative Western blots of MYH7, ANP, and BNP protein expression in heart tissue, with GAPDH as a loading control (n = 6). (Q) Densitometric quantification of the blots shown in (P) (n = 6). (R, S) mRNA expression levels of hypertrophy‐associated (R) and inflammation‐related (S) genes in heart tissues (n = 6). Data were normalized to *Actb* (n = 6). All quantitative data are presented as mean ± SEM. Data between the two groups were compared by independent‐sample two‐tailed Student's t‐test. Data among multiple groups were compared by one‐way ANOVA test, followed by Tukey post hoc test. ns indicates not statistically significant; **p < 0.01.

To delineate the direct role of TRIM40 in cardiomyocytes, we achieved cardiomyocyte‐specific knockdown of TRIM40 in mice via tail vein injection of AAV9‐cTnT‐shTRIM40 (experimental design shown in Figure S4A, followed by a 4‐week infusion of Ang II or saline. The results showed that cardiomyocyte‐specific TRIM40 knockdown did not affect the Ang II‐induced elevation in systemic blood pressure (Figure 4A) or changes in plasma Ang II concentration (Figure 4B), but it led to significant improvements in cardiac functional parameters, including preserved EF and FS, along with a shortened IVRT (Figure 4C–F; Table S4). Concurrently, this intervention reduced serum levels of the myocardial injury marker CK‐MB (Figure 4G) and suppressed the increase in the HW/BW (Figure 4H). Both gross morphological examination of the hearts (Figure 4I) and quantitative analysis of their cross‐sectional area (Figure 4J) demonstrated significant alleviation of cardiac hypertrophy. Dual immunofluorescence staining for cTnT and WGA, along with its quantitative analysis, further confirmed that this approach prevented the increase in cardiomyocyte cross‐sectional area (Figure 4K,L). H&E staining revealed improvements in tissue architecture (Figure 4M; Figure S4B), while Picrosirius Red and Masson's trichrome staining showed a significant reduction in the extent of myocardial fibrosis (Figure 4N,O). Further quantitative analysis of interstitial and perivascular fibrosis (Figure S4C–H) consistently supported these findings. At the molecular level, cardiomyocyte‐specific TRIM40 knockdown downregulated the protein (Figure 4P,Q) and mRNA (Figure 4R) expression of hypertrophy markers (MYH7, ANP, and BNP) and inhibited the upregulation of inflammation‐related genes (Figure 4S). These results demonstrate that the autonomous function of TRIM40 within cardiomyocytes is a critical driver of Ang II‐induced pathological cardiac remodeling.

### 2.5 TRIM40 Mediated Ang II‐Induced Cardiomyocyte Hypertrophy In Vitro

**FIGURE 5:**
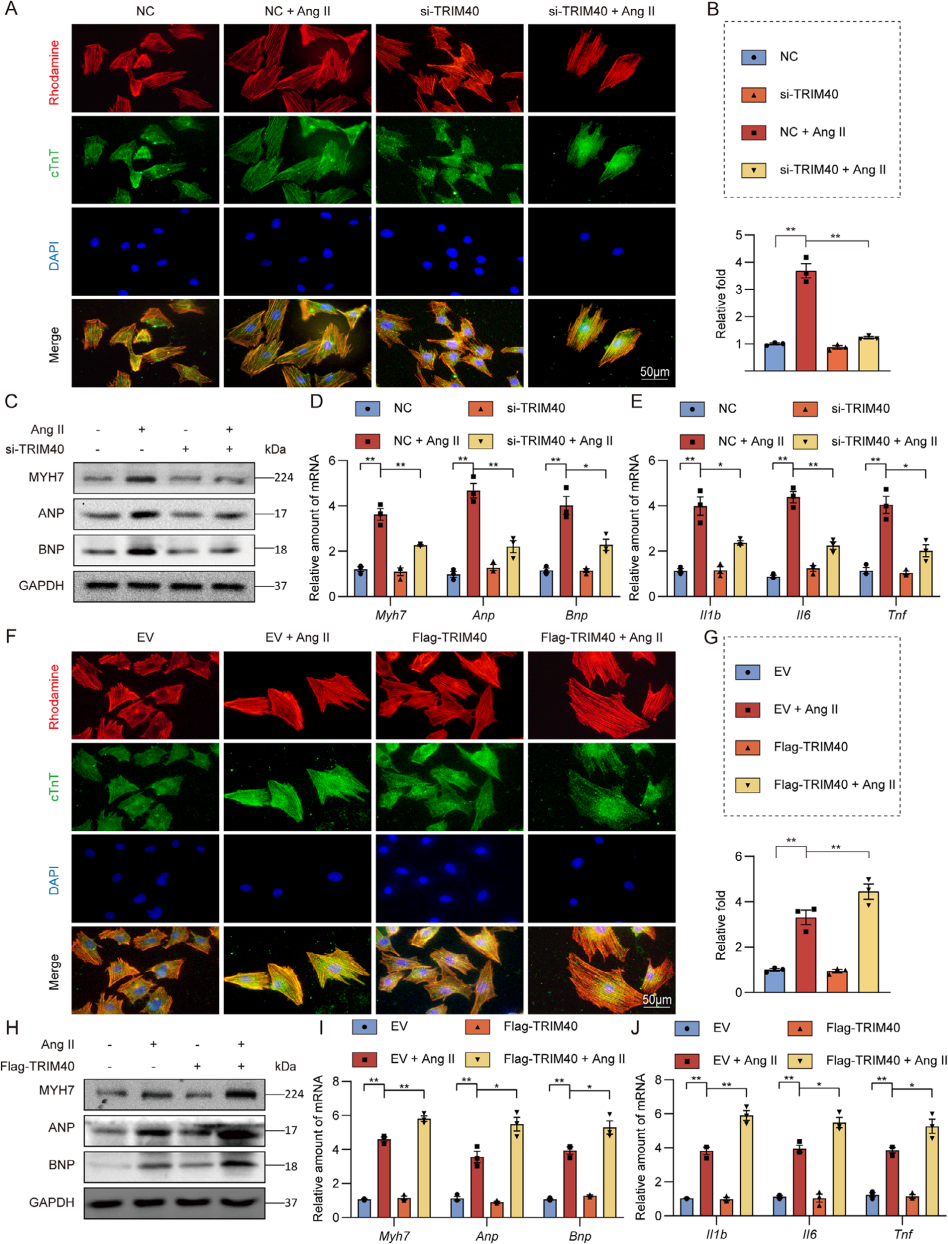
TRIM40 regulates cardiomyocyte hypertrophic and fibrotic responses in culture. (A) After transfection with either a negative control (NC) or TRIM40 siRNA (si‐TRIM40), NRVMs were treated with Ang II (1 µm for 24 h). Cardiomyocyte size was assessed by dual immunofluorescence staining for cTnT (green) and Rhodamine‐phalloidin (red). Nuclei were counterstained with DAPI (blue) (scale bar = 50 µm) (n = 3). (B) Changes in NRVMs size in response to Ang II. At least 100 cells from each of three independent samples were analyzed per group (n = 3). (C) Representative western blot of MYH7, ANP, and BNP proteins in NRVMs, with GAPDH as loading control (n = 3). (D, E) The mRNA levels of hypertrophy‐associated genes (D) and inflammation‐related genes (E) in NRVMs treated as indicated. Data was normalized to *Actb* (n = 3). (F) Following transfection with Flag‐TRIM40 or an empty vector (EV), NRVMs were treated with Ang II (1 µm for 24 h). Cardiomyocyte size was assessed by the dual immunofluorescence staining method described in (A) (scale bar = 50 µm) (n = 3). (G) Changes in NRVMs size in response to Ang II. At least 100 cells from each of three independent samples were analyzed per group (n = 3). (H) Representative western blot of MYH7, ANP, and BNP proteins in NRVMs, with GAPDH as loading control (n = 3). (I, J) mRNA expression levels of hypertrophy‐associated genes (I) and inflammation‐related genes (J) in NRVMs. Data were normalized to *Actb* (n = 3). All quantitative data are presented as mean ± SEM. Data between the two groups were compared by independent‐sample two‐tailed Student's t‐test. Data among multiple groups were compared by one‐way ANOVA test, followed by Tukey post hoc test; *p < 0.05, **p < 0.01.

To further investigate the functional mechanisms of TRIM40 in cardiomyocytes, we exposed NRVMs to 1 µm Ang II at different time points and monitored the dynamic changes in TRIM40 protein expression. The results demonstrated that Ang II stimulation led to a rapid increase in TRIM40 protein levels, detectable within 60 min (Figure S5A,B). After screening multiple small interfering RNA (siRNA) sequences (Table S5 and Figure S5C,D), we identified the most efficient sequence (#2) and used it for all subsequent TRIM40 knockdown experiments. As expected, dual immunofluorescence staining for cTnT and Rhodamine‐phalloidin revealed that Ang II stimulation significantly increased cardiomyocyte surface area, while TRIM40 knockdown prevented this Ang II‐induced increase (Figure 5A,B). At the molecular level, TRIM40 knockdown similarly inhibited the expression of hypertrophy‐related markers (Figure 5C; Figure S5E). Notably, when TRIM40 was silenced, Ang II failed to induce the upregulation of hypertrophy‐ and inflammation‐related genes (Figure 5D,E), thereby recapitulating and validating our in vivo observations. We then expressed TRIM40 in NRVMs (Figure S5F,G) and performed similar experiments as with silencing. The results showed that TRIM40 overexpression enhanced Ang II‐induced cellular hypertrophy and inflammatory responses (Figure 5F–J; Figure S5H). These findings collectively demonstrate that TRIM40 plays an indispensable and critical role in Ang II‐induced cardiomyocyte hypertrophy and inflammatory activation.

### 2.6 TRIM40 Directly Interacts with PKN2

**FIGURE 6:**
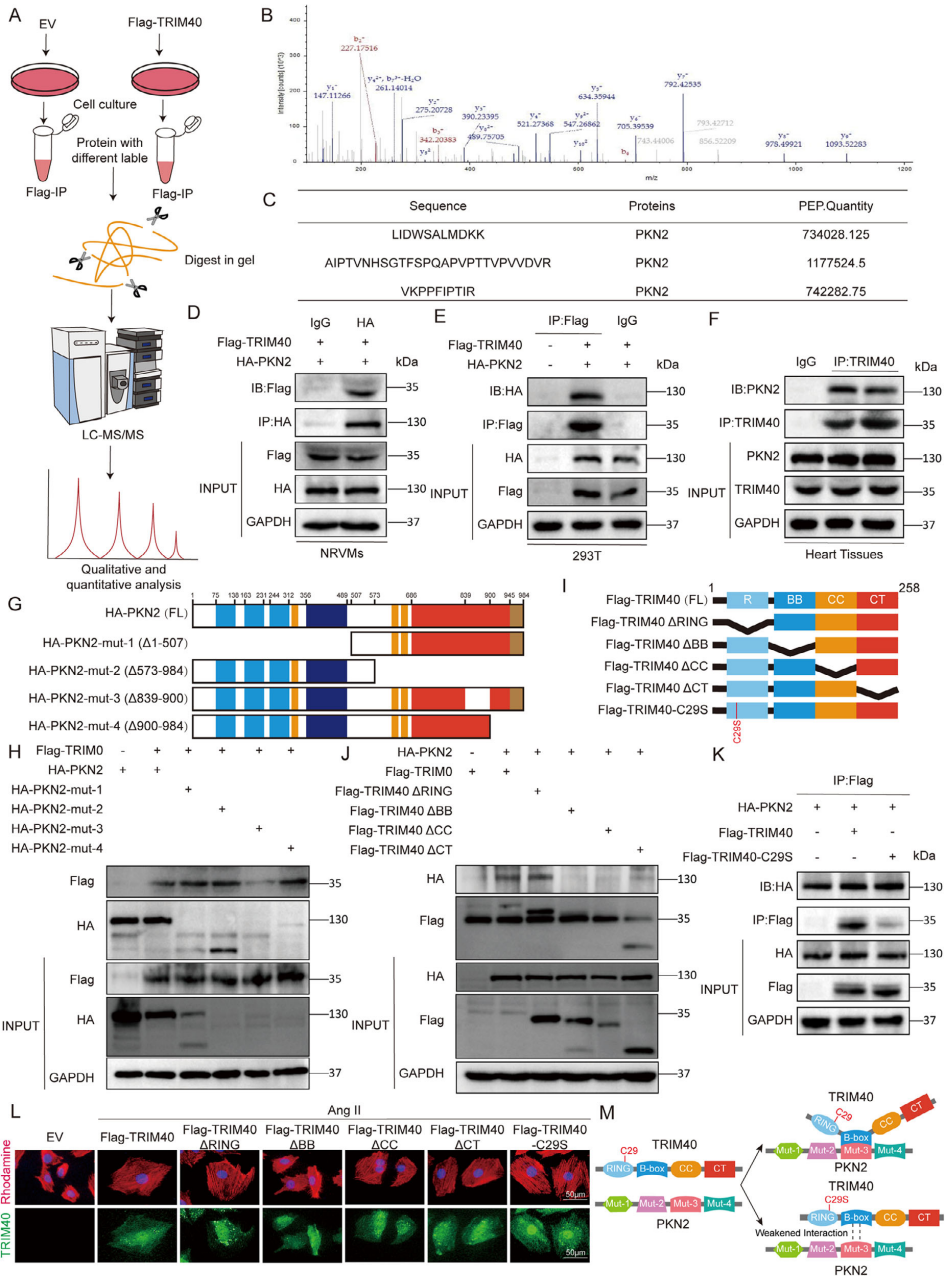
TRIM40 interacts directly with PKN2. (A) Schematic diagram of the quantitative proteomic screening workflow for identifying TRIM40‐interacting proteins. (B) Tandem mass spectrum of representative peptide fragments from PKN2. (C) Amino acid sequence information of the identified PKN2 peptides. (D, E) Co‐IP assays using anti‐Flag antibody in NRVMs (D) and HEK‐293T cells (E) transfected with Flag‐tagged TRIM40, followed by immunoblotting to detect PKN2 association. IgG served as a NC for Co‐IP (n = 3). (F) Endogenous PKN2 binding was detected by immunoblotting after Co‐IP with anti‐TRIM40 antibody from mouse heart tissue lysates. IgG was used as a control (n = 6). (G) Schematic representation of the domain deletion mutants of PKN2. (H) HEK‐293T cells were co‐transfected with HA‐tagged full‐length PKN2 or its deletion mutants together with Flag‐TRIM40. Immunoprecipitation was performed using anti‐HA antibody, followed by immunoblotting to detect Flag‐TRIM40 binding (n = 3). (I) Schematic diagrams of TRIM40 domain deletion mutants and its catalytically inactive mutant (C29S). (J) HEK‐293T cells were co‐transfected with Flag‐tagged full‐length TRIM40 or its mutants together with HA‐PKN2. Immunoprecipitation with anti‐Flag antibody was used to assess HA‐PKN2 binding (n = 3). (K) Ubiquitination assay of PKN2 in HEK‐293T cells co‐expressing Myc‐Ub, HA‐PKN2, and the catalytically inactive mutant Flag‐TRIM40‐C29S. HA immunoprecipitates were analyzed by immunoblotting to detect PKN2 ubiquitination. (n = 3) (L) Confocal microscopy images showing the effects of different TRIM40 variants (full‐length, deletion mutants, and C29S mutant) on F‐actin cytoskeleton organization in NRVMs. Cells were stained with rhodamine‐conjugated phalloidin (red, labeling F‐actin) and anti‐TRIM40 antibody (green, indicating transfected TRIM40 variants), with nuclei counterstained by DAPI (blue). Scale bar = 50 µm (n = 3). (M) Structural basis of the TRIM40‐PKN2 interaction.

To systematically screen for potential TRIM40 substrates in the heart, we performed co‐immunoprecipitation coupled with mass spectrometry (Co‐IP/MS) (Figure 6A). Among the potential TRIM40‐binding proteins, PKN2 attracted our attention due to its established involvement in cardiac development and pathological hypertrophy [26]. Liquid chromatography‐tandem mass spectrometry (LC‐MS/MS) analysis identified 3 PKN2‐matching unit peptides in Flag‐TRIM40 group, suggesting a potential interaction between TRIM40 and PKN2 (Figure 6B,C). Next, Co‐IP assay verified that the complex of TRIM40 and PKN2 was formed in co‐transfected NRVMs and 293T cells (Figure 6D,E). Furthermore, the same interaction was observed in endogenous Co‐IP experiments using mouse cardiac tissues (Figure 6F), indicating that TRIM40 interacts with PKN2 under physiological conditions. PKN2 contains Mut‐1(Δ1–507) domain, Mut‐2(Δ573–984) domain, Mut‐3(Δ839‐900) domain and Mut‐4(Δ900–984) domain [27]. To investigate the TRIM40‐PKN2 binding interface, we generated a series of truncated PKN2 mutants (Figure 6G). Co‐IP analysis revealed that the Mut‐3 domain of PKN2 is required for its binding to TRIM40 (Figure 6H). Similarly, we generated a series of TRIM40 mutants, including RING, B‐box, CC, CT domain (Figure 6I). And the B‐Box domain of TRIM40 was identified as the primary region mediating its interaction with PKN2 (Figure 6J). After identifying TRIM40‐PKN2 interaction site, we mutated active sites on TRIM40 (cysteine at position 29 to serine). Co‐IP assays revealed that this mutant exhibited markedly impaired binding capability to PKN2 compared to WT TRIM40 (Figure 6K). To systematically decipher the structural basis of TRIM40‐driven cardiomyocyte hypertrophy, we expressed WT TRIM40 and a series of domain‐specific truncation mutants in NRVMs, and assessed cellular hypertrophy through phalloidin staining. The results demonstrated that under Ang II stimulation, WT‐TRIM40 overexpression induced significant cardiac hypertrophy, characterized by increased cell surface area. In stark contrast, ΔB‐box mutants completely lost their pro‐hypertrophic capacity, with cellular phenotypes indistinguishable from the EV control group. Notably, the E3 ligase‐inactive mutant partially attenuated TRIM40's pro‐hypertrophic effect (Figure 6L). These results define the TRIM40‐PKN2 interaction, mediated by the TRIM40 B‐box domain and dependent on C29, as a critical event for TRIM40 to promote cardiomyocyte hypertrophy (Figure 6M).

### 2.7 TRIM40 Regulates the Activity of PKN2 Through Ubiquitination

**FIGURE 7:**
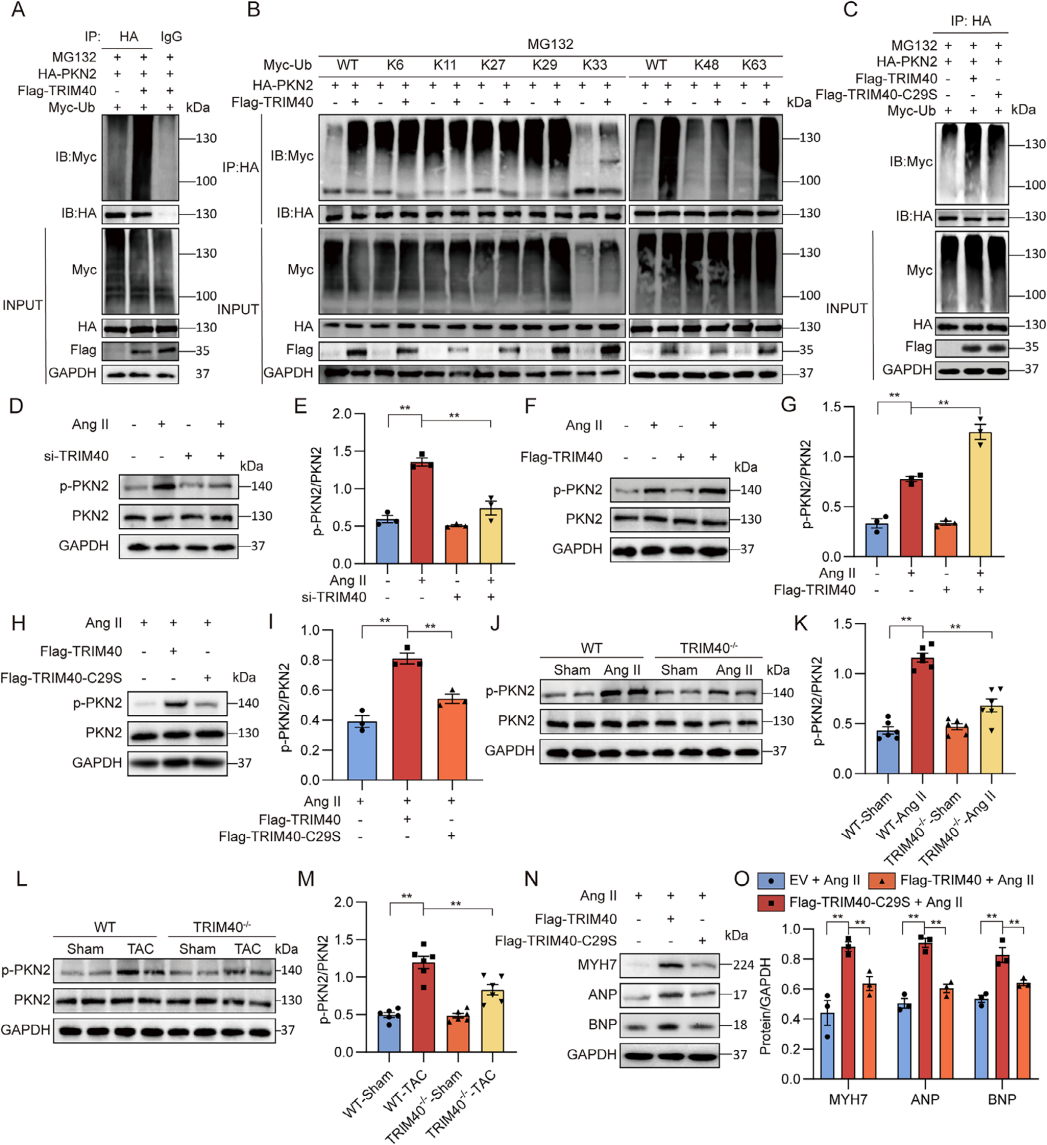
TRIM40 Positively Regulates the Phosphorylation of PKN2 by Mediating Its K63‐Linked Ubiquitination. (A) HEK‐293T cells were transfected with HA‐PKN2, Myc‐Ub, and Flag‐TRIM40, and treated with 10 µm MG132 for 6 h before harvesting. The ubiquitination level of PKN2 was detected by immunoblotting using an anti‐HA antibody (Control = IgG) (n = 3). (B) HEK‐293T cells were co‐transfected with HA‐PKN2, Flag‐TRIM40, and various types of Myc‐Ub (including WT, K6‐, K11‐, K27‐, K29‐, K33‐, K48‐, and K63‐linked ubiquitin chains). After immunoprecipitation with anti‐HA magnetic beads, the ubiquitination of PKN2 was analyzed by immunoblotting. Cells were pretreated with 10 µm MG132 for 6 h before harvesting (n = 3). (C) HEK‐293T cells were transfected with HA‐PKN2, Myc‐Ub, EV, Flag‐TRIM40, and Flag‐TRIM40‐29S, treated with 10 µm MG132 for 6 h before harvesting. The ubiquitination level of PKN2 was detected by immunoblotting using an anti‐HA antibody (n = 3). (D) NRVMs were transfected with siRNA targeting TRIM40, followed by treatment with 1 µm Ang II for 12 h. The phosphorylation level of PKN2 was detected by immunoblotting, with GAPDH used as the loading control (n = 3). (E) Quantitative data of the blot intensity of corresponding proteins determined by Image J software in panel D (n = 3). (F) NRVMs were transfected with a TRIM40 expression vector, followed by treatment with 1 µm Ang II for 12 h. The phosphorylation level of PKN2 was detected by immunoblotting, with GAPDH used as the loading control (n = 3). (G) Quantitative data of the blot intensity of corresponding proteins determined by Image J software in panel F (n = 3). (H) NRVMs overexpressing Flag‐TRIM40 or Flag‐TRIM40‐C29S were treated with Ang II (1 µm) for 24 h. Phosphorylation of PKN2 at Ser815 (p‑PKN2) was detected by Western blot. GAPDH served as a loading control (n = 3). (I) Densitometric quantification of the Western blot bands from Figure 7H (n = 3). (J) The level of p‐PKN2 was detected by immunoblotting in whole heart tissue lysates from mice infused with Ang II for 4 weeks (n = 6). (K) Quantitative data of the blot intensity of corresponding proteins determined by Image J software in panel J (n = 6). (L) The level of p‐PKN2 was detected by immunoblotting in whole heart tissue lysates from mice subjected to TAC surgery (n = 6). (M) Quantitative data of the blot intensity of corresponding proteins determined by Image J software in panel L (n = 6). (N) NRVMs overexpressing Flag‐TRIM40 or Flag‐TRIM40‐C29S were treated with Ang II (1 µm) for 24 h. Protein levels of MYH7, ANP and BNP were detected by Western blot. GAPDH served as a loading control (n = 3). (O) Densitometric quantification of the Western blot bands from Figure 7N (n = 3). All quantitative data are presented as mean ± SEM. Data between two groups were compared by independent‐sample two‐tailed Student's t‐test. Data among multiple groups were compared by one‐way ANOVA test, followed by Tukey post hoc test; **p < 0.01.

To investigate whether TRIM40 directly regulates PKN2 through ubiquitination, we co‐transfected HA‐PKN2, Flag‐TRIM40, and Myc‐Ubiquitin (Ub) plasmids into HEK‐293T cells and treated the cells with MG132 to prevent proteasomal degradation of PKN2. The results showed that TRIM40 effectively promotes the ubiquitination level of PKN2 (Figure 7A). To identify the type of ubiquitin chain mediated by TRIM40, we performed ubiquitination assays using a series of ubiquitin mutants (K6, K11, K27, K29, K33, K48, K63). Figure 7B demonstrates that TRIM40 primarily promotes K63‐linked ubiquitination of PKN2. To further explore the direct effect of K63 ubiquitination on PKN2 activity, we conducted an in vitro kinase assay. Using the ADP‐Glo kinase assay system, we found that, compared to the basal control and the Ub‐K63R control, K63‐linked ubiquitination significantly enhanced the kinase activity of PKN2 (Figure S6A). Notably, this enhancement in kinase activity occurred without a concomitant increase in the phosphorylation level of PKN2 at its classical Ser815 site (Figure S6B,C), suggesting that K63 ubiquitination enhances PKN2 activity via a mechanism that does not require increased phosphorylation at its canonical Ser815 site, potentially involving allosteric regulation. To confirm the necessity of TRIM40's E3 ubiquitin ligase activity in this process, we constructed a key site mutant (C29S) in its RING domain. As shown in Figure 7C, compared to wild‐type TRIM40, the C29S mutant significantly attenuated the ability to promote PKN2 ubiquitination.

The effect of PKN2 ubiquitination on its phosphorylation status in cells has not been previously reported. We subsequently examined the phosphorylation level of PKN2 at Ser815 in cardiomyocytes following modulation of TRIM40. First, we silenced TRIM40 expression in NRVMs and treated the cells with 1 µm Ang II for 12 h. The results showed that knockdown of TRIM40 significantly inhibited the Ang II‐induced increase in p‐PKN2 levels in cardiomyocytes (Figure 7D,E). Conversely, overexpression of TRIM40 in cardiomyocytes further enhanced the Ang II‐induced increase in p‐PKN2 (Figure 7F,G). Notably, overexpression of the TRIM40‐C29S mutant failed to enhance Ang II‐induced PKN2 phosphorylation as the wild‐type did (Figure 7H,I). To validate this phenomenon at the whole‐organism level, we assessed the effect of TRIM40 deficiency on PKN2 activation in mouse heart tissues induced by Ang II infusion and TAC surgery. Analysis of heart tissue lysates indicated that both Ang II and TAC treatment enhanced PKN2 phosphorylation in WT mice, whereas this enhancement was markedly suppressed in TRIM40^−/−^ mice (Figure 7J–M).

Finally, we performed a functional rescue experiment in the cardiomyocyte hypertrophy model. Consistent with the above molecular findings, overexpression of the TRIM40‐C29S mutant did not exacerbate Ang II‐induced cellular hypertrophy as wild‐type TRIM40 did (Figure 7N,O). In summary, these experiments collectively demonstrate that TRIM40, via its E3 ubiquitin ligase activity (dependent on the C29 residue within its RING domain), promotes K63‐linked ubiquitination of PKN2. This modification facilitates the phosphorylation of PKN2 at Ser815 and enhances its kinase activity, ultimately driving cardiomyocyte hypertrophy.

### 2.8 TRIM40 Increases Ang II‐Induced Pathological Hypertrophy and Dysfunction in Mice by Regulating PKN2

To determine whether PKN2 mediates the pro‐hypertrophic effects of TRIM40 in vivo, we established a cardiac‐specific overexpression model by tail vein injection of adeno‐associated virus (AAV9) viruses carrying the TRIM40 coding sequence, followed by a 2‐week Ang II infusion in mice. Experimental groups also included mice treated with the PKN2 inhibitor PKN1/2‐IN‐1. To clarify whether PKN1/2‐IN‐1 exerts its protective effects primarily by inhibiting PKN2, we first systematically validated its specificity at the cellular level. Western blot analysis confirmed that this inhibitor effectively reduces PKN2 protein levels (Figure S7A,B). Further evaluation of its selectivity toward phosphorylation showed that Ang II stimulation increased the phosphorylation of both PKN1 and PKN2; however, treatment with PKN1/2‐IN‐1 led to a significantly stronger inhibition of PKN2 phosphorylation than of PKN1 phosphorylation (Figure S7C,D). Genetic evidence demonstrated that siRNA‐mediated knockdown of PKN2 exerted a significantly stronger inhibitory effect on Ang II‐induced upregulation of hypertrophy markers than knockdown of PKN1, and its effect was comparable to that of PKN1/2‐IN‐1 treatment (Figure S7E,F). These results provide evidence supporting that the protective effects of PKN1/2‐IN‐1 in our system are largely attributable to the inhibition of PKN2, justifying its use in subsequent animal experiments.

**FIGURE 8:**
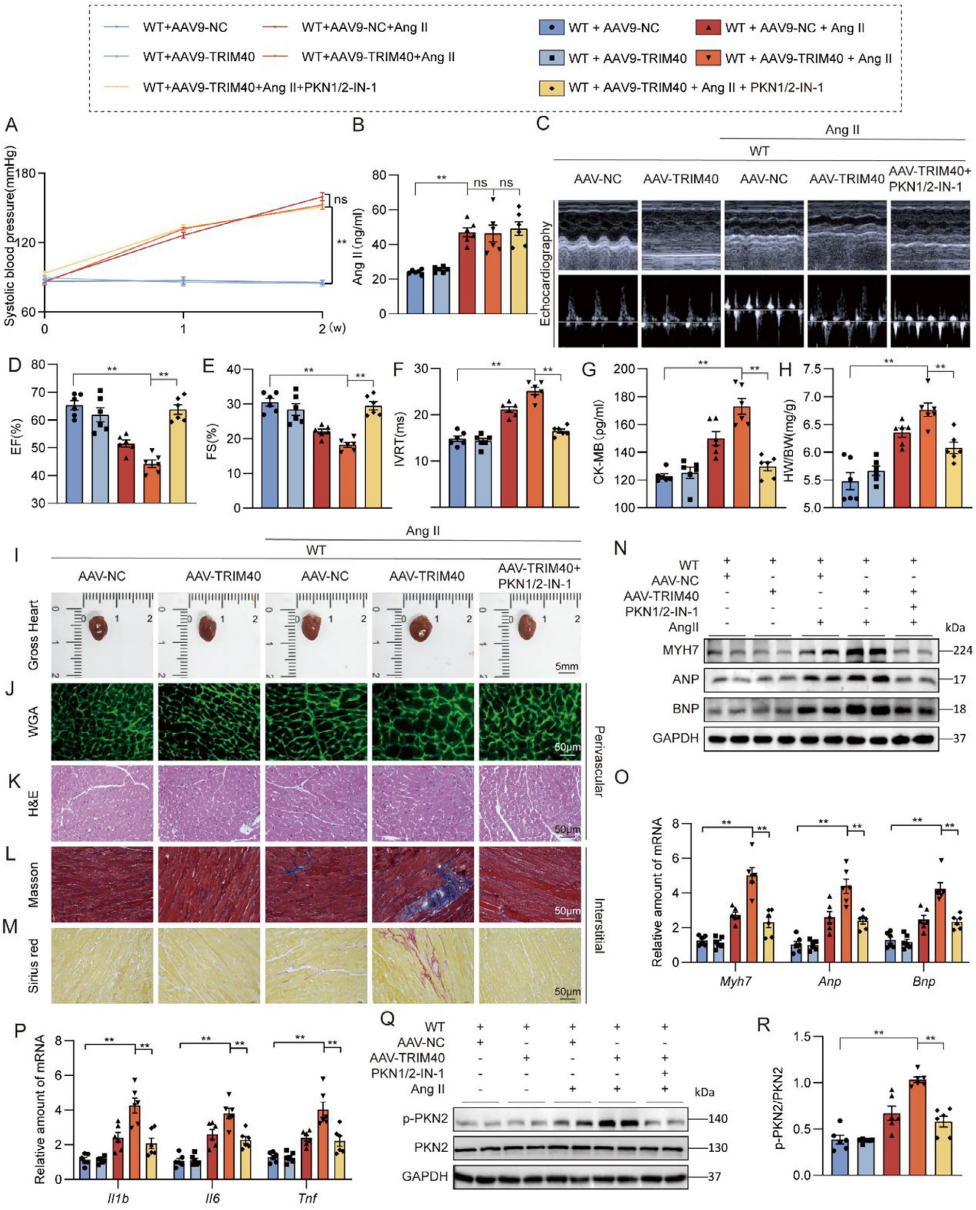
TRIM40 exacerbates Ang II‐induced cardiomyocyte hypertrophy and fibrosis by regulating PKN2. C57BL/6 mice received two injections of AAV9 encoding TRIM40 at one‐month intervals, followed by continuous infusion of saline or Ang II for two weeks. (A) Mouse systolic blood pressure was measured weekly using a non‐invasive tail‐cuff system (n = 6). (B) Serum Ang II concentration was detected in mice (n = 6). (C) Representative echocardiographic images of mice from each experimental group (n = 6). (D–F) Cardiac function parameters showing EF (D), FS (E), and IVRT (F) (n = 6). (G) Serum CK‐MB levels in mice (n = 6). (H) HW/BW of mice in each group (n = 6). (I) Representative freshly isolated heart specimens photographed against a white background (scale bar = 5 mm) (n = 6). (J) Cardiomyocyte size was assessed by fluorescein‐conjugated WGA staining (scale bar = 50 µm) (n = 6). (K) H&E staining of heart tissue sections (scale bar = 50 µm) (n = 6). (L, M) Myocardial fibrosis was evaluated by Masson's trichrome staining (L) and Picrosirius red staining (M) (scale bar = 50 µm) (n = 6). (N) Western blot analysis of MYH7, ANP, and BNP in myocardial tissues, with GAPDH as a loading control (n = 6). (O, P) mRNA expression levels of hypertrophy‐associated genes (O) and inflammation‐related genes (P) in myocardial tissues, normalized to *Actb* (n = 6). (Q) p‐PKN2 levels were detected by immunoblotting in whole heart lysates from mice infused with Ang II for 4 weeks (n = 6). (R) Quantitative data of the blot intensity of corresponding proteins determined by Image J software in panel Q (n = 6). All quantitative data are presented as mean ± SEM. Data between the two groups were compared by independent‐sample two‐tailed Student's t‐test. Data among multiple groups were compared by one‐way ANOVA test, followed by a Tukey post hoc test. ns indicates not statistically significant; *p < 0.05, **p < 0.01.

The specific experimental design is illustrated in Figure S8A. AAV9 successfully transduced mouse cardiac tissue and significantly elevated TRIM40 protein expression levels (Figure S8B,C). Ang II infusion effectively induced a hypertensive model, with experimental mice exhibiting significantly elevated systolic blood pressure (Figure 8A) and markedly increased plasma Ang II concentration (Figure 8B), indicating that TRIM40 expression does not affect the systemic pressor response to Ang II. Echocardiographic analysis revealed that cardiac TRIM40 expression exacerbated Ang II‐induced cardiac dysfunction, specifically manifested as significantly reduced EF and FS, along with prolonged IVRT. This exacerbation was prevented by treatment with the PKN2 inhibitor PKN1/2‐IN‐1 (Figure 8C–F; Table S6). Furthermore, CK‐MB levels were elevated in cardiac tissue expressing TRIM40 and decreased after PKN1/2‐IN‐1 treatment (Figure 8G). Additionally, TRIM40 overexpression resulted in increased HW/BW in mice, and these TRIM40‐induced increases were attenuated by PKN1/2‐IN‐1 treatment (Figure 8H). Gross examination of heart tissues indicated that TRIM40 expression promotes Ang II‐induced cardiac hypertrophy (Figure 8I; Figure S8D). Histological analysis using H&E and WGA staining further confirmed that cardiac TRIM40 expression enhanced the hypertrophic changes and structural abnormalities caused by Ang II stimulation (Figure 8J,K; Figure S8E,F), and these pathological responses were significantly attenuated after PKN1/2‐IN‐1 treatment. Similarly, Picro Sirius Red and Masson's trichrome staining showed that TRIM40 expression aggravated the degree of cardiac fibrosis induced by Ang II stimulation (Figure 8L,M; Figure S8G–L), and these pathological alterations were markedly improved following PKN2 inhibitor treatment. At the molecular level, consistent with the above findings, TRIM40 expression significantly enhanced the upregulation of cardiac hypertrophy‐related proteins and marker genes (Figure 8N,O; Figure S8M) as well as inflammation‐related genes (Figure 8P) under Ang II stimulation. These changes were effectively blocked by PKN2 inhibition. More importantly, we found that Ang II‐induced PKN2 phosphorylation levels were significantly elevated in hearts expressing TRIM40, and this activation process was effectively suppressed by PKN2 inhibitor treatment (Figure 8Q,R). These in vivo data establish that the pro‐hypertrophic effects of TRIM40 are largely dependent on PKN2 activation.

### 2.9 TRIM40 Increases TAC‐Induced Pathological Hypertrophy and Dysfunction in Mice by Regulating PKN2

**FIGURE 9:**
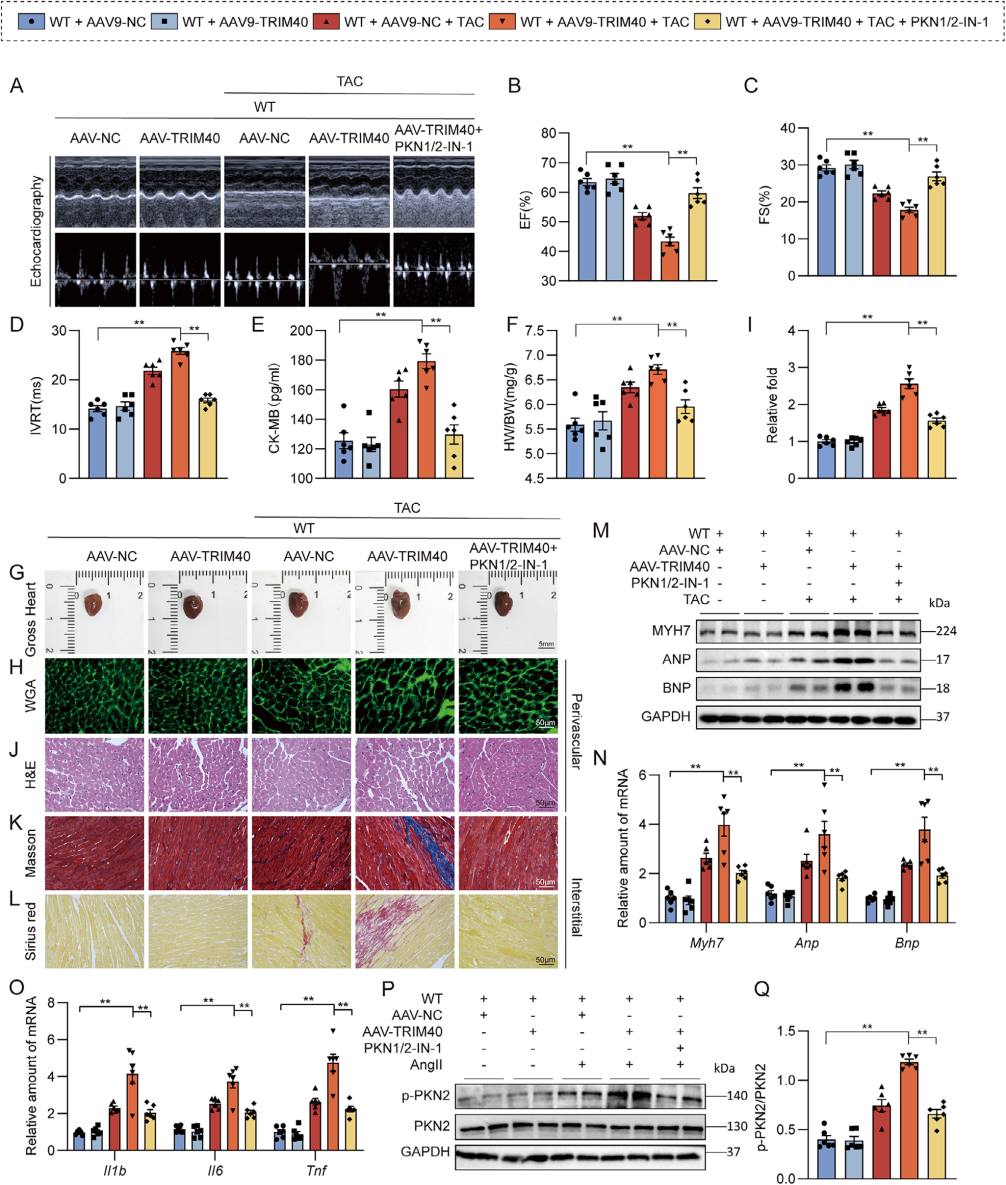
TRIM40 exacerbates TAC‐induced cardiomyocyte hypertrophy and fibrosis by regulating PKN2. C57BL/6 mice received two injections of AAV9 encoding TRIM40 at one‐month intervals, followed by sham surgery or TAC surgery for two weeks. (A) Representative echocardiographic images of mice from each experimental group (n = 6). (B–D) Cardiac function parameters showing EF (B), FS (C), and IVRT (D) (n = 6). (E) Serum CK‐MB levels in mice (n = 6). (F) HW/BW of mice in each group (n = 6). (G) Representative freshly isolated heart specimens photographed against a white background (scale bar = 5 mm) (n = 6). (H) Cardiomyocyte size was assessed by fluorescein‐conjugated WGA staining (scale bar = 50 µm) (n = 6). (I) Cardiomyocyte hypertrophy evaluated by fluorescein‐conjugated WGA staining (scale bar = 50 µm) (n = 6). (J) H&E staining of heart tissue sections (scale bar = 50 µm) (n = 6). (K, L) Myocardial fibrosis was evaluated by Masson's trichrome staining (K) and Picrosirius red staining (L) (scale bar = 50 µm) (n = 6). (M) Western blot analysis of MYH7, ANP, and BNP in myocardial tissues, with GAPDH as a loading control (n = 6). (N, O) mRNA expression levels of hypertrophy‐associated genes (N) and inflammation‐related genes (O) in myocardial tissues, normalized to *Actb* (n = 6). (P) p‐PKN2 levels were detected by immunoblotting in whole heart lysates from mice infused with Ang II for 4 weeks (n = 6). (Q) Quantitative data of the blot intensity of corresponding proteins determined by Image J software in panel P (n = 6). All quantitative data are presented as mean ± SEM. Data between the two groups were compared by independent‐sample two‐tailed Student's t‐test. Data among multiple groups were compared by one‐way ANOVA test, followed by Tukey post hoc test; **p < 0.01.

Finally, to extend our findings to a model of pressure overload, we assessed the role of TRIM40 and its dependence on PKN2 using TAC surgery. Mice were treated with the pharmacological PKN2 inhibitor PKN1/2‐IN‐1. The specific experimental design is shown in Figure S9A. Consistent with the observations in the Ang II model, TRIM40 expression increased TAC‐induced cardiac dysfunction (Figure 9A–D; Table S7), and this exacerbation was attenuated by the PKN2 inhibitor. TRIM40 expression also elevated serum CK‐MB levels, which were reduced following PKN1/2‐IN‐1 treatment (Figure 9E). Moreover, TRIM40 expression further enhanced the TAC‐induced increases in HW/BW ratios (Figure 9F). In addition, TRIM40 expression aggravated TAC‐induced cardiac hypertrophy, structural abnormalities, and fibrosis (Figure 9G–N; Figure S9B–J), and these pathological changes were also significantly suppressed by PKN1/2‐IN‐1. Assessment of inflammatory markers revealed a similar trend of upregulation (Figure 9O). More importantly, TRIM40 expression enhanced the TAC‐induced elevation in p‐PKN2 levels, and this activation was similarly inhibited by PKN1/2‐IN‐1 (Figure 9P,Q). Thus, the pro‐hypertrophic function of TRIM40 and its reliance on PKN2 are conserved across distinct models of cardiac stress.

## 3 Discussion

Due to the limited regenerative capacity of cardiomyocytes, the UPS serves as the major pathway for protein quality control and is essential for preserving cellular homeostasis and cardiac function [28]. Targeting the stability of key regulatory proteins within cardiomyocytes thus represents a promising therapeutic strategy for heart diseases. As central components of the UPS, E3 ubiquitin ligases have been implicated in the regulation of cardiac hypertrophy through modulating the turnover of critical proteins. For instance, RNF207 promotes cardiac hypertrophy by mediating K63‐linked ubiquitination of TAB1 [29], whereas WWP2 attenuates isoproterenol‐induced cardiac remodeling by facilitating the degradation of PARP1 [30]. Similarly, MARCH2 interacts with PGAM5 and promotes its proteasomal degradation via K48‐linked polyubiquitination in cardiomyocytes, thereby alleviating inflammatory responses and pyroptotic cell death following ischemia/reperfusion (I/R) injury [31]. These findings collectively underscore the pivotal roles of E3 ubiquitin ligases in the pathogenesis of heart disease. Here, we identifyTRIM40 as an important regulator of inflammation in cardiac hypertrophy induced by Ang II or pressure overload. Our study demonstrates that TRIM40 deficiency attenuates cardiac hypertrophy and improves cardiac function by reducing PKN2 phosphorylation through decreased ubiquitination of PKN2, thereby lowering the level of cardiomyocyte inflammation. Importantly, loss of TRIM40 does not alter the baseline inflammatory activity required for maintaining physiological cardiac function, nor does it impair normal heart development. These findings implicate cardiomyocyte TRIM40 as a potential candidate for therapeutic intervention for HF.

It is well established that cardiac hypertrophy, characterized by thickening of the heart muscle, is closely associated with chronic inflammation, which involves immune cell activation and the release of inflammatory mediators [32]. This persistent inflammatory response can be triggered by various cardiac stressors, such as hypertension or myocardial injury [33]. It may both contribute to the development of hypertrophy and result from it, thereby exacerbating cardiac dysfunction and ultimately leading to HF [1]. During this process, the renin‐angiotensin system (RAS) and the sympathetic‐adrenomedullary system are activated. As cardiac hypertrophy progresses, the secretion of pro‐inflammatory mediators‐including inflammatory cytokines and chemokines‐is upregulated [34]. Under the guidance of chemokines, immune cells migrate from the circulation into the heart, where they release pro‐hypertrophic cytokines such as tumor necrosis factor α (TNF‐α), interleukin 1b (IL‐1β), and interleukin 6 (IL‐6). In this context, the upregulation of cytokine expression not only promotes cardiac hypertrophy but also enhances Ang II production via activation of the RAS, thereby accelerating the progression of hypertrophy and contributing to impaired cardiac function [35]. In this study, we observed that both Ang II infusion and TAC surgery significantly increased the levels of inflammation in cardiomyocytes during the induction of hypertrophy. Notably, TRIM40 expression was significantly upregulated in hypertrophic hearts, which was accompanied by increased PKN2 phosphorylation and elevated inflammation. Overall, TRIM40 deficiency ameliorated cardiac hypertrophy by alleviating cardiomyocyte hypertrophy and inflammation under pathological conditions.

The protein kinase N (PKN) family has recently emerged as a potential therapeutic target for heart disease. In mammalian cells, three isoforms of PKN have been identified: PKN1, PKN2, and PKN3 [36]. Previous studies have demonstrated that PKN is involved in regulating the transition of fibroblasts to myofibroblasts during fibrosis [37]. It is noteworthy that deficiency of PKN2 in cardiomyocytes severely impairs ventricular myocardial development, while cardiomyocyte‐specific KO of PKN2 attenuates cardiac hypertrophy induced by Ang II or pressure overload in vivo [26]. Cardiac myocyte PKN2 is not only essential for cardiac development and the formation of compact myocardium, but also required for cardiac hypertrophy in response to hypertension [26]. Although PKN2 represents promising therapeutic targets, and their potential has been demonstrated in various animal models, the outcomes of many ongoing clinical trials aimed at evaluating their efficacy and safety remain inconclusive. Therefore, exploring upstream regulatory proteins of PKN2 is another strategy for cardiac hypertrophy therapy. Our data indicate that TRIM40 acts as a regulator of PKN2 protein and demonstrate that loss of TRIM40 reduces the phosphorylation level of PKN2, effectively alleviating inflammatory injury in cardiac hypertrophy induced by TAC or Ang II.

The activation of PKN is an exceptionally complex process, involving membrane binding of the enzyme, phosphorylation‐induced initiation, conformational changes triggered by the binding of proteins or second messengers (such as Ca^2^^+^ and phosphatidylserine), as well as the release of pseudo substrates. This enables substrates to access the catalytic cleft within the kinase domain [36]. It has been demonstrated that PKN2 may be activated by MEK kinase 2 (MEKK2), a mitogen‐activated protein kinase kinase [38]. The unique features of ubiquitin (Ub) and its ability to form various homo‐ and heterotypic link age types involving one of the seven different lysine residues or the free amino group located at its N‐terminus. K48‐ and K63‐linked chains are broadly covered in the literature [39]. The major function of K48 linkages is to regulate protein stability negatively. Most other lysine‐mediated linkage types, apart from K63 linkages, are also thought to target proteins for proteasomal degradation. K63 linkages have been crucially implicated in nonproteolytic signaling processes [40]. In this study, we provide the first evidence that TRIM40 promotes K63‐linked ubiquitination on PKN2 protein, which affects PKN2 phosphorylation and thereby regulates pathological cardiac hypertrophy. Our study establishes TRIM40 as an E3 ligase for PKN2 and demonstrates that TRIM40‐mediated ubiquitination and phosphorylation of PKN2 play a role in inflammation‐related cardiac hypertrophy. Furthermore, mutation of the conserved C29 residue (C29S) impaired the interaction between TRIM40 and PKN2. Targeting this specific site to reduce PKN2 phosphorylation could therefore be explored as a potential therapeutic avenue for treating cardiac hypertrophy and other inflammation‐related diseases. Unfortunately, the effect of PKN2 ubiquitination on its phosphorylation status has not been previously reported. It would be of great interest to explore the mechanism by which PKN2 ubiquitination influences its phosphorylation. We hypothesize that TRIM40‐mediated ubiquitination could alter the conformational dynamics of PKN2, potentially facilitating its autophosphorylation. This hypothesis warrants further investigation through structural biology approaches in future studies.

This study has several limitations. First, our in vivo models capture the early hypertrophic phase but not the chronic progression to heart failure. Second, while genetic data support the specific role of PKN2, the use of a dual PKN1/2 inhibitor means a minor contribution from PKN1 inhibition cannot be entirely excluded. Third, the mechanistic findings of this study are primarily based on preclinical models, and the direct clinical relevance of the TRIM40‐PKN2 axis in human heart failure remains to be established. Future validation of this pathway at both the protein and functional levels in human cardiac tissue samples is warranted. Future studies addressing these points will help translate these findings.

**FIGURE 10:**
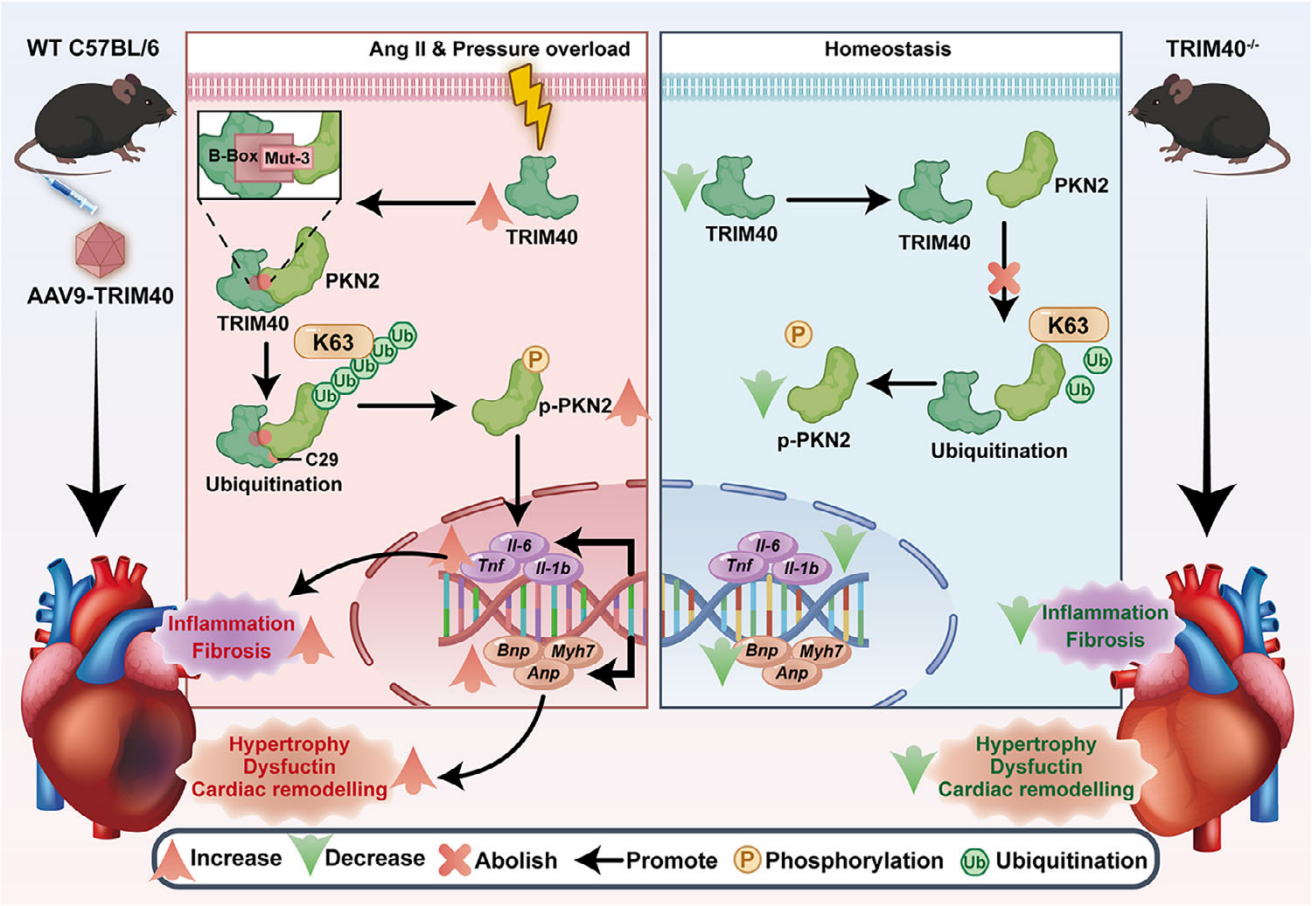
A Mechanism of TRIM40 Driving Cardiac Hypertrophy through PKN2 Ubiquitination.

In conclusion, this study defines TRIM40 as a key regulator of pathological cardiac hypertrophy. The E3 ubiquitin ligase activity of TRIM40 constitutes an essential element of the TRIM40‐PKN2 axis, modulating cardiac hypertrophy by regulating both ubiquitination and phosphorylation processes. Mechanistically, the B‐box domain of TRIM40 directly binds to the Mut‐3 region of PKN2, facilitating K63‐linked ubiquitination. Furthermore, the cysteine residue at position 29 of TRIM40 serves as a critical site mediating its binding to PKN2; the C29S mutation impairs this interaction, thereby suppressing the TRIM40‐driven pro‐hypertrophic response (Figure 10). These findings elucidate a previously unrecognized mechanism driving pathological cardiac hypertrophy and highlight the therapeutic potential of modulating the TRIM40‐PKN2 axis for HF.

## 4 Methods

### 4.1 Reagents

Ang II (cat# 4474913) was purchased from Aladdin (Shanghai, China). siRNAs against TRIM40 and scrambled sequences were purchased from Genepharma (Shanghai, China). siRNAs targeting PKN1 and PKN2 were purchased from Ambion (Thermo Fisher Scientific, USA). The overexpression plasmids, including Flag‐TRIM40, Flag‐TRIM40 ΔRING, Flag‐TRIM40 ΔBB, Flag‐TRIM40 ΔCC, Flag‐TRIM40 ΔCT, Flag‐TRIM40‐C29S, HA‐PKN2, HA‐PKN2‐mut‐1 (Δ1–507), HA‐PKN2‐mut‐2 (Δ573–984), HA‐PKN2‐mut‐3 (Δ839–900), HA‐PKN2‐mut‐4 (Δ900–984), Myc‐Ub WT, Myc‐Ub‐K6, Myc‐Ub‐K11, Myc‐Ub‐K27, Myc‐Ub‐K29, Myc‐Ub‐K33, Myc‐Ub‐K48, and Myc‐Ub‐K63, Myc‐Ub‐K63R were purchased from Tsingke Biotechnology Co., Ltd. (Beijing, China). AAV9‐TRIM40, EV, AAV9‐cTNT‐TRIM40, and AAV9‐cTNT‐NC were obtained from Genechem (Shanghai, China). Antibodies against TRIM40 (24526‐1‐AP), MYH7 (22280‐1‐AP), ANP (27426‐1‐AP), BNP (13299‐1‐AP), PKN2 (14608‐1‐AP), cTnT (68300‐1‐Ig), Rabbit IgG (B900610), His (6005‐1‐lg), Flag (20543‐1‐AP) and HA (51064‐2‐AP) were purchased from Proteintech (IL, USA). Antibodies against alpha actinin (ab68167), and vimentin (ab8978) were obtained from Abcam (Cambridge, UK). Antibodies against phospho‐PKN2 (Ser815) (TA7406) was purchased from Abmart (Shanghai, China). Antibodies against GAPDH (5174) and phospho‐PINK1 (Ser228) (46421) were purchased from Cell Signaling Technology (MA, USA).

Ang II ELISA kit was purchased from Shanghai Tongwei Biological Technology Co., Ltd (Shanghai, China). CK‐MB kits (cat# H180, E006‐1‐1) was purchased from Nanjing Jiancheng Bioengineering Institute (Nanjing, China). ADP‐Glo Kinase Assay kit (cat# V9101) was purchased from Promega Corporation (Madison, WI, USA). Wheat germ agglutinin (WGA, cat# GTX01502) was purchased from Gene Tex (CA, USA). Rhodamine‐conjugated Phalloidin (Phalloidin‐Rho, cat# CA1610‐300T), DAPI (cat# S2110), hematoxylin and eosin (H&E) kit (cat# G1120), Picro Sirius Red stain (cat# S8060), and Masson's Trichrome kit (cat# G1340) were purchased from Solarbio Life Sciences (Beijing, China). PKN1/2‐IN‐1 (HY‐145899), a PKN2 inhibitor, was purchased from MedChemExpress (New Jersey, USA).

### 4.2 Experimental Animals and Ethical Considerations

WT C57BL/6 mice were supplied by Changchun Yisi Laboratory Animal Technology Co., Ltd. TRIM40 knockout (TRIM40^−/−^) mice on a C57BL/6J background were generated by Nanjing GemPharmatech Co., Ltd. Using CRISPR/Cas9 technology, the Trim40 gene was modified. Briefly, gRNA was transcribed in vitro, and then both Cas9 mRNA and gRNA were microinjected into the fertilized eggs of C57BL/6JGpt mice. These microinjected fertilized eggs were then transplanted to produce F0 mice. Potential founders were identified by PCR and sequencing. Finally, a stable F1 generation mouse model was established by crossing PCR‐positive F0 mice with wild‐type C57BL/6JGpt mice.

All animal experiments were conducted in compliance with the guidelines of the Animal Policy and Welfare Committee of Beihua University, and the study protocol was approved by this committee (No. SCXK(JI)2023‐0002). Every effort was made to minimize animal suffering and to reduce the number of animals used. The study also adhered to the National Institutes of Health (NIH) principles and the ARRIVE guidelines for reporting animal research [41]. Prior to experimentation, all animals were acclimatized to the laboratory environment for at least two weeks. All procedures and data analyses were performed in a blinded manner, and treatment groups were assigned in a randomized fashion.

### 4.3 Animal Experiments

To induce pathological cardiac hypertrophy and remodeling, this study employed both chronic Ang II infusion and TAC models.

1. In the Ang II infusion model, 6‐ to 8‐week‐old male WT and TRIM40^−/−^ mice were subcutaneously implanted in the back with an osmotic minipump (Alzet Model 1004) containing either saline or Ang II (1 µg/kg/min). The experimental groups (n = 6 per group) included: WT‐Sham, WT‐Ang II, TRIM40^−/−^‐Sham, and TRIM40^−/−^‐Ang II. BW and non‐invasive blood pressure were monitored weekly. After 4 weeks of intervention, the animals were euthanized, and serum and heart tissues were collected for subsequent analysis.
2. In the TAC model, mice were anesthetized with isoflurane, followed by tracheal intubation and connection to a ventilator. After opening the thoracic cavity, the aortic arch was isolated between the innominate artery and the left common carotid artery. A 6‐0 suture was used to ligate the transverse aorta against a blunted 27‐gauge needle, which was then removed to establish constriction. The sham‐operated group underwent the same procedure without aortic ligation. The model demonstrated a high success rate, with over 90% survival within the first 2 h post‐surgery, and no significant difference in early postoperative survival was observed between WT and TRIM40^−/−^ mice. Experimental groups (n = 6 per group) included: WT‐Sham, WT‐TAC, TRIM40^−/−^‐Sham, and TRIM40^−/−^‐TAC. After 4 weeks of intervention, the animals were euthanized for the collection of serum and heart tissues.
3. To investigate the effect of cardiomyocyte‐restricted TRIM40 knockdown, a separate cohort of C57BL/6J WT mice received tail vein injections of AAV9 vectors (HANBIO, Shanghai, China) at a dose of 1 × 10^1^ ^2^ viral genome particles per mouse. These vectors were driven by the cardiac‐specific troponin T (cTnT) promoter to mediate the expression of a short hairpin RNA targeting TRIM40 (AAV9‐cTnT‐shTRIM40) or a non‐targeting control (AAV9‐cTnT‐CTL). Following a 2‐week period for sufficient viral transduction and gene knockdown, mice were subjected to either Sham surgery or chronic Ang II infusion (1 µg/kg/min for 4 weeks) via osmotic minipump. Thus, the experimental groups (n = 6 per group) were: AAV9‐cTnT‐CTL + Sham, AAV9‐cTnT‐CTL + Ang II, AAV9‐cTnT‐shTRIM40 + Sham, and AAV9‐cTnT‐shTRIM40 + Ang II.
4. To investigate the role of TRIM40 in cardiac hypertrophy and remodeling, TRIM40 was overexpressed in C57BL/6J WT mice. Specifically, AAV9 carrying the mouse TRIM40 gene (AAV9‐TRIM40) or the corresponding control (AAV9‐NC) was administered via tail vein injection at a dose of 3 × 10^1^ ^1^ viral particles per mouse. The viruses were constructed and prepared by GeneChem Co., Ltd. (Shanghai, China). A 4‐week period was allowed to ensure stable TRIM40 overexpression in myocardial tissue. After this viral expression period, the mice were subjected to either the Ang II infusion or TAC surgery, as described above, to induce cardiac remodeling. Additional subgroups received the PKN2 inhibitor PKN1/2‐IN‐1 during the final two weeks of intervention. For the Ang II infusion model, experimental groups (n = 6 per group) were: AAV9‐NC, AAV9‐TRIM40, AAV9‐NC + Ang II, AAV9‐TRIM40 + Ang II, and AAV9‐TRIM40 + Ang II + PKN1/2‐IN‐1. For the TAC model, groups (n = 6 per group) were: AAV9‐NC, AAV9‐TRIM40, AAV9‐NC + TAC, AAV9‐TRIM40 + TAC, and AAV9‐TRIM40 + TAC + PKN1/2‐IN‐1.

One day prior to euthanasia, all mice underwent echocardiographic examination under isoflurane anesthesia to assess cardiac function. After euthanasia, hearts were harvested and weighed, and tibia length (TL) was measured to calculate the HW/TL. Portions of the cardiac tissue were snap‐frozen in liquid nitrogen for subsequent molecular biological analysis, while other portions were fixed in 4% paraformaldehyde for histological examination. Blood samples were centrifuged at 4°C and 3000 rpm for 15 min to obtain serum, which was used to measure Ang II and CK‐MB levels using commercial ELISA kits.

### 4.4 RNA‐Sequencing Analysis

RNA was isolated from murine heart tissues using TRIzol reagent (cat# 15596018; Thermo Fisher) after 4 weeks of saline or Ang II infusion. Following RNA quality control by denaturing agarose gel electrophoresis, cDNA library preparation was performed involving fragmentation of poly(A) RNA, reverse transcription with SuperScript II Reverse Transcriptase (cat# 1896649; Thermo Fisher), and PCR amplification. The final libraries (average size: 300 ± 50 bp) were sequenced on an Illumina Novaseq 6000 (LC‐BioTechnology Co., Ltd.) in PE150 mode. Differential gene expression analysis applied a fold‐change cutoff of 2.0 (up‐ or down‐regulated) and a significance level of *p* < 0.05. Gene‐set enrichment analysis was carried out using LC‐Bio's standard pipeline (https://www.lc‐bio.cn/).

### 4.5 Cardiac Tissue Staining

Mouse heart tissues were processed using both cryosectioning and paraffin embedding methods. Paraffin‐embedded tissues were sectioned into 5 µm thick slices for histological analysis, which included H&E staining, Masson's trichrome staining, and Sirius red staining. The stained sections were imaged using a light microscope (400x magnification; Leica Microsystems, Germany). For quantitative analysis of Sirius red and Masson's trichrome staining, five random fields per slide were measured and averaged.

To determine cardiomyocyte cross‐sectional area, 5 µm thick cardiac cryosections were permeabilized with 0.1% Triton X‐100 for 10 min, followed by incubation with WGA (1:200) at 37°C for 30 min. Nuclei were counterstained with DAPI. All images were acquired using a Nikon fluorescence microscope (Tokyo, Japan).

For immunofluorescence staining, heart tissues were embedded in OCT compound and sectioned into 5 µm thick slices. The sections were fixed with 4% paraformaldehyde for 15 min, permeabilized with 0.1% Triton X‐100 for 10 min, and then blocked with 5% bovine serum albumin for 45 min. The sections were subsequently incubated overnight at 4°C with the following primary antibodies: TRIM40 (1:200), α‐actinin (1:200), vimentin (1:200), and cTnT (1:200). Antigen‐antibody complexes were detected with corresponding fluorescent secondary antibodies, and nuclei were counterstained with DAPI for 5 min. Images were finally acquired using a Nikon A1R HD laser confocal microscope (Japan).

### 4.6 Cell Culture Studies

The human embryonic kidney HEK‐293T cell line (RRID: CVCL_0063) and the immortalized rat cardiomyocyte cell line H9c2 (RRID: CVCL_0286) were purchased from the Shanghai Cell Resource Center of the Chinese Academy of Sciences (Shanghai, China). Both cell types were cultured in Dulbecco's Modified Eagle Medium (DMEM; Gibco/BRL, Germany) supplemented with 10% fetal bovine serum (FBS; Hyclone, USA), 100 U/mL penicillin, and 100 U/mL streptomycin. All cell lines were routinely tested and confirmed to be free of mycoplasma contamination. Cells were maintained in a humidified incubator at 37°C with 5% CO_2_, subcultured every 2–3 days at a split ratio of 1:4 to 1:6, and cells in the logarithmic growth phase were consistently selected for experiments.

NRVMs isolation from 5‐day‐old rats and the NRVMs culture were conducted as in past reports [42]. Briefly, trypsin was used to dissociate cardiac tissue from neonatal Wistar rats, after which they were transferred into tissue culture dishes containing DMEM supplemented with 10% FBS for 1 h to allow for the removal of a proportion of the non‐myocyte cells. Differential adherence culture was used to remove NRCFs, culturing those cells in DMEM with 4.5 g/L glucose and 10% FBS. Those cells that remained non‐adherent were transferred into 6‐well plates. Primary cardiomyocytes were cultured in DMEM with 10% FBS and penicillin/streptomycin.

Primary mouse peritoneal macrophages (MPMs) were isolated from C57BL/6 wild‐type mice. The mice received an intraperitoneal injection of 1 mL 4% thioglycolate in PBS. After three days, peritoneal cells were harvested and cultured in DMEM/F12 medium supplemented with 10% fetal bovine serum for 4 h. The cells were subsequently maintained at 37°C for 6 h, followed by PBS washing to remove non‐adherent cells. The remaining adherent cells were utilized as peritoneal macrophages for subsequent experiments.

### 4.7 LC‐MS/MS Analysis

TRIM40 antibody was added to NRVMs lysates for IP. Rabbit IgG was used as a NC. Then, the LC‐MS/MS analysis was carried out by PTM Bio Co., Ltd (Zhejiang, China). Finally, we screened out the substrate proteins that could bind to TRIM40 according to the score and the mass of detected proteins.

### 4.8 Gene Silencing and Overexpression

Gene silencing was achieved by transfecting cells with siRNA. Specific siRNAs were designed and synthesized for the rat TRIM40 gene. For the knockdown of PKN1 and PKN2, specific siRNAs targeting each gene were used. All siRNA sequences used for gene knockdown in NRVMs are listed in Tables S5 and S8. Transfection of NRVMs was carried out using Lipofectamine RNA iMAX Transfection Reagent (Invitrogen, Carlsbad, CA, USA).

The overexpression plasmids, including Flag‐TRIM40, Flag‐TRIM40 ΔRING, Flag‐TRIM40 ΔBB, Flag‐TRIM40 ΔCC, Flag‐TRIM40 ΔCT, Flag‐TRIM40‐C29S, HA‐PKN2, HA‐PKN2‐mut‐1 (Δ1‐507), HA‐PKN2‐mut‐2 (Δ573‐984), HA‐PKN2‐mut‐3 (Δ839‐900), HA‐PKN2‐mut‐4 (Δ900‐984), Myc‐Ub WT, Myc‐Ub‐K6, Myc‐Ub‐K11, Myc‐Ub‐K27, Myc‐Ub‐K29, Myc‐Ub‐K33, Myc‐Ub‐K48, and Myc‐Ub‐K63, Myc‐Ub‐K63R were purchased from Tsingke Biotechnology Co., Ltd. (Beijing, China). For transient transfection into HEK‐293T cells, these plasmids were introduced using Lipofectamine 3000 reagent (Invitrogen, Carlsbad, CA, USA) following the manufacturer's instructions.

### 4.9 Double Immunofluorescence and Rhodamine Phalloidin Staining

To determine the subcellular localization of TRIM40 protein and visualize the cytoskeletal architecture, a dual‐label fluorescence staining procedure was employed. Briefly, cells were fixed with 4% paraformaldehyde for 15 min at room temperature and subsequently permeabilized with 0.1% Triton X‐100 for 10 min. After blocking with 5% bovine serum albumin for 1 h to minimize nonspecific binding, the samples were incubated with a primary antibody against TRIM40 overnight at 4°C. The following day, unbound primary antibody was removed by three washes with PBS, and the cells were incubated with a species‐appropriate Alexa Fluor 488‐conjugated secondary antibody for 1 h at room temperature, protected from light. Following additional PBS washes, F‐actin was counterstained by incubating the cells with Rhodamine Phalloidin for 30 min under light‐protected conditions. Finally, cell nuclei were labeled using a DAPI‐containing mounting medium. Fluorescence images were acquired using a confocal microscope.

For specific quantification of the surface area of cultured cardiomyocytes, a dual immunofluorescence staining targeting cTnT and F‐actin was performed. The procedures for cell fixation and permeabilization were the same as described above. After blocking, the samples were incubated with an anti‐cTnT antibody (1:200) overnight at 4°C. Following washes, they were incubated with an Alexa Fluor 488‐conjugated secondary antibody (1:500) for 1 h at room temperature, protected from light. Subsequently, F‐actin was visualized by staining with Rhodamine Phalloidin (1:200) for 30 min under light‐protected conditions. Nuclei were counterstained with DAPI. Fluorescence images were acquired using a confocal microscope.

### 4.10 Real‐Time qPCR

Total RNA was extracted from cardiac tissues and cultured cells using TRIzol reagent (Thermo Fisher). Subsequently, RNA was reverse‐transcribed into cDNA using Prime Script RT reagent (Takara Bioscience, Ann Arbor, MI, USA). All quantitative real‐time PCR (qPCR) reactions were performed on a CFX96 Touch system (Bio‐Rad) with SYBR Green fluorescence dye (Vazyme Biotech Co., Ltd., Nanjing, China) for amplification signal detection. The primer sequences used are listed in Table S9. The relative expression levels of target genes were calculated by the 2^−ΔΔCT^ method using *Actb* as the internal reference gene.

### 4.11 Western Blot Analysis

Total proteins were extracted from cells and tissues using RIPA lysis buffer (Beyotime Biotechnology). Protein samples were separated by 8%, 10%, or 12% SDS‐polyacrylamide gel electrophoresis and subsequently transferred to polyvinylidene fluoride membranes using a wet transfer system. After blocking with 5% skim milk for 1 h at room temperature, the membranes were incubated with specific primary antibodies overnight at 4°C. The following day, the membranes were washed with TBST buffer and then incubated with corresponding secondary antibodies for 1 h at room temperature. Target protein bands were visualized using enhanced chemiluminescence (ECL) detection reagent (Bio‐Rad) and quantified by grayscale analysis with Image J software version 1.53i. Protein expression levels were normalized to GAPDH as an internal control.

### 4.12 Co‐Immunoprecipitation (Co‐IP)

To investigate protein–protein interactions, protein A+G agarose beads (Beyotime Biotechnology) were incubated with specific antibodies to form immunocomplexes. Normal IgG antibody was used as a NC. After overnight incubation at 4°C, the agarose beads were washed six times with ice‐cold PBS and collected by centrifugation at 3000 ×*g* for 2 min each time. The proteins specifically bound to the target protein were finally detected by immunoblotting.

### 4.13 Statistical Analysis

Data are expressed as mean ± standard error of the mean (SEM). The exact group size (n), representing biological replicates (n = 6 for animal experiments; n = 3 for cell experiments), is provided in figure legends. All data were collected and analyzed by observers blinded to group assignments. Data preprocessing included checking for outliers and normalization when appropriate. Normality was assessed using the Shapiro‑Wilk test for in‑vivo experiments (*p* > 0.05 considered approximately normal) and assumed for in‑vitro data based on the central limit theorem. Homogeneity of variances was verified before applying parametric tests. Comparisons between two groups used a two‑tailed unpaired Student's t‑test. For comparisons among more than two groups, one‑way ANOVA was performed, followed by Tukey's post‑hoc test only if the overall ANOVA was significant (F‑test *p* < 0.05) and variances were homogeneous. For repeated measures from the same individual, a two‑way repeated‑measures ANOVA with single pooled variance was applied, followed by Tukey's correction. All tests were two‑sided with a significance level (alpha) of 0.05 (*p* < 0.05 considered significant). Analyses were performed using GraphPad Prism 8.0 (GraphPad, San Diego, CA, USA).

## Author Contributions

**Mengyang Wang**: Writing – original draft, Writing – review and editing, Data curation, Funding acquisition, Project administration, Resources. **Xiaoli Cui**: Methodology, Validation, Writing – review and editing. **Huizhu Du**: Software, Methodology. **Zhuoqun Wang**: Validation, Visualization. **Chang Liu**: Software, Methodology, Formal analysis. **Linxin Zhang**: Methodology, Validation. **Jianing Qi**: Validation, Visualization. **Di Yang**: Conceptualization, Methodology, Formal analysis. **Hui Yu**: Methodology, Validation. **Shuang Yan**: Investigation, Resources, Supervision. **Wei Liu**: Software, Methodology, Formal analysis. **Risheng Zhao**: Writing – original draft, Data curation. **Haiming Sun**: Writing – review and editing, Resources.

## Ethics Approval

All animal experiments were conducted in accordance with the guidelines of the Animal Policy and Welfare Committee of Beihua University. The study protocol was reviewed and approved by the same committee (No. SCXK(JI)2023‐0002).

## Conflicts of Interest

The authors declare no conflicts of interest.

## Supporting information




**Supporting File 1**: advs73796‐sup‐0001‐SuppMat.docx.

**Supporting File 2**: advs73796‐sup‐0002‐Supplementary Figures_Raw_Data_Figures.zip.

**Supporting File 3**: advs73796‐sup‐0003‐Western Blot_Raw_Data_Figures.pdf.

**Supporting File 4**: advs73796‐sup‐0004‐Data.zip.

## Data Availability

The data that support the findings of this study are available in the supplementary material of this article.
