# Supplementary material for: TRIM40 Drives Pathological Cardiac Hypertrophy and Heart Failure via Ubiquitination of PKN2
Source: Adv Sci (Weinh). 2026 Jan 22;13(17):e21337. doi: 10.1002/advs.202521337 (PMC13042792; doi:10.1002/advs.202521337)
Supplement: Supplementary file 1 — Supporting File 1: advs73796‐sup‐0001‐SuppMat.docx. [file ADVS-13-e21337-s002.docx]

*Supplemental Information*

**TRIM40 Drives Pathological Cardiac Hypertrophy and Heart Failure via Ubiquitination of PKN2**

**Running Title:** TRIM40 promotes pathological cardiac hypertrophy.

**Supplementary Table S1:** Differentially expressed E3 ubiquitin ligases in cardiac tissue from Ang II-infused mice vs. control mice.

| gene_name | log2FoldChange | pvalue | level |
| --- | --- | --- | --- |
| TRIM40 | 3.6536993 | 0.0010125 | up |
| Rnf180 | -1.56227 | 0.0057077 | down |
| Trim43a | -4.331922 | 0.0580621 | ns |
| Trim69 | 3.843801 | 0.1229531 | ns |
| Marchf11 | -3.549829 | 0.1577628 | ns |
| Rnf182 | -2.626732 | 0.5692396 | ns |
| Trim38 | 1.9352421 | 0.313748 | ns |
| Rnf32 | 1.9352278 | 0.3138103 | ns |
| Rnf223 | 1.7145927 | 0.5199415 | ns |
| Trim9 | 1.2915868 | 0.6545169 | ns |

Genes are ranked by absolute log₂ Fold Change value. “Up” or “Down” indicates significantly dysregulated (adjusted p-value < 0.01 & log₂FC > 1); ns indicates not significant.

**Supplementary Table S2:** Biometric and echocardiographic parameters in Ang II-challenged mice.

| Model 1 (Ang II model) | sham | | Continuous Ang II Pump Infusion | |
| --- | --- | --- | --- | --- |
|  | WT | TRIM40^-/-^ | WT | TRIM40^-/-^ |
|  | n=6 | n=6 | n=6 | n=6 |
| HR, bpm | 527.67±24.44 | 552.83±20.31 | 537.50±24.17 | 541.33±19.24 |
| EF, % | 65.89±4.78 | 68.31±6.12 | 44.50±2.89** | 59.76±2.85## |
| FS, % | 30.97±3.35 | 32.78±4.91 | 18.39±1.47** | 26.87±1.79## |
| LVIDd, mm | 2.58±0.08 | 2.43±0.19 | 2.90±0.14** | 2.67±0.22# |
| IVSD, mm | 0.64±0.05 | 0.67±0.04 | 0.54±0.06** | 0.70±0.03## |
| PWD, mm | 0.65±0.03 | 0.65±0.05 | 0.83±0.06** | 0.66±0.06## |
| Tei Index | 0.62±0.06 | 0.62±0.06 | 0.93±0.08** | 0.79±0.11# |
| IVRT, ms | 15.50±1.52 | 14.17±1.72 | 24.67±1.86** | 17.50±1.87## |
| HW/BW, mg/g | 5.89±0.57 | 5.58±0.58 | 6.55±0.27* | 5.61±0.16## |
| HW/TL, mg/mm | 6.87±0.40 | 7.11±0.62 | 7.66±0.11** | 7.23±0.34# |

Transthoracic echocardiography was performed on mice at the end of the animal study. Ang II = angiotensin II; BW = body weight; EF = ejection fraction; FS = fractional shortening; HR = heart rate; HW = heart weight; IVRT = isovolumic relaxation time; IVSD = diastole interventricular septal thickness; LVIDd = diastole left ventricle internal dimension; PWD = diastole posterior wall thickness; Tei index = a myocardial performance index. Data presented as Mean ± SEM; * = *p* < 0.05 and ** = *p* < 0.01 compared to WT-Sham; # = *p* < 0.05 and ## = *p* < 0.01 compared to WT + Ang II.

**Supplementary Table S3:** Biometric and echocardiographic parameters in TAC-challenged mice.

| Model 2 (TAC model) | sham | | TAC | |
| --- | --- | --- | --- | --- |
|  | WT | TRIM40^-/-^ | WT | TRIM40^-/-^ |
|  | n=6 | n=6 | n=6 | n=6 |
| HR, bpm | 531.33±16.68 | 549.33±18.02 | 542.00±17.69 | 542.33±19.43 |
| EF, ％ | 67.24±0.05 | 66.97±0.02 | 44.11±0.03** | 56.45±0.02## |
| FS, ％ | 31.99±0.04 | 31.64±0.02 | 18.21±0.01** | 24.25±0.01## |
| LVIDd, mm | 2.65±0.14 | 2.68±0.17 | 3.02±0.12** | 2.75±0.30 |
| IVSD, mm | 0.64±0.04 | 0.63±0.03 | 0.88±0.03** | 0.69±0.04## |
| PWD, mm | 0.64±0.04 | 0.63±0.03 | 0.81±0.06** | 0.70±0.24## |
| Tei Index | 0.64±0.05 | 0.60±0.05 | 0.97±0.14** | 0.67±0.08## |
| IVRT, ms | 15.33±1.37 | 14.83±1.33 | 23.50±1.52** | 16.12±1.33## |
| HW/BW, | 5.47±0.31 | 5.53±0.38 | 6.22±0.32** | 5.58±0.23## |
| mg/g |  |  |  |  |
| HW/TL, mg/mm | 7.04±0.51 | 7.11±0.52 | 7.83±0.39* | 7.20±0.43# |

Transthoracic echocardiography was performed on mice at the end of the animal study. TAC = transverse aortic constriction; BW = body weight; EF = ejection fraction; FS = fractional shortening; HR = heart rate; HW = heart weight; IVRT = isovolumic relaxation time; IVSD = diastole interventricular septal thickness; LVIDd = diastole left ventricle internal dimension; PWD = diastole posterior wall thickness; Tei index = a myocardial performance index. Data presented as Mean ± SEM; * = *p* < 0.05 and ** = *p* < 0.01 compared to WT-Sham; # = *p* < 0.05 and ## = *p* < 0.01 compared to WT + TAC.

**Supplementary Table S4:** Biometric and echocardiographic parameters in Ang II-challenged mice treated with AAV9-cTnT-TRIM40.

| Model 3 (Ang II model) |  | | Continuous Ang II Pump Infusion | |
| --- | --- | --- | --- | --- |
|  | AAV9-cTnT-NC | AAV9-cTnT-TRIM40 | AAV9-cTnT-NC | AAV9-cTnT-TRIM40 |
|  | n=6 | n=6 | n=6 | n=6 |
| HR, bpm | 533.33±22.15 | 555.33±18.27 | 538.17±24.34 | 539.67±11.38 |
| EF, ％ | 69.86±5.47 | 74.25±2.85 | 44.79±3.97** | 62.78±7.28## |
| FS, ％ | 33.92±4.20 | 37.22±2.34 | 18.57±2.02** | 29.07±5.04## |
| LVIDd, mm | 2.65±0.08 | 2.55±0.10 | 2.97±0.12** | 2.75±0.14# |
| IVSD, mm | 0.64±0.04 | 0.66±0.03 | 0.83±0.05** | 0.71±0.03## |
| PWD, mm | 0.65±0.03 | 0.64±0.04 | 0.83±0.04** | 0.67±0.05## |
| Tei Index | 0.64±0.05 | 0.65±0.05 | 0.84±0.06** | 0.72±0.03## |
| IVRT, ms | 15.33±1.51 | 14.67±1.63 | 22.67±1.21** | 18.67±0.82 |
| HW/BW, mg/g | 4.25±0.35 | 4.08±0.22 | 6.11±0.16** | 4.79±0.11## |
| HW/TL, mg/mm | 5.34±0.31 | 5.21±0.31 | 7.74±0.30** | 6.12±0.23## |

Transthoracic echocardiography was performed on mice at the end of the animal study. Ang II = angiotensin II; BW = body weight; EF = ejection fraction; FS = fractional shortening; HR = heart rate; HW = heart weight; IVRT = isovolumic relaxation time; IVSD = diastole interventricular septal thickness; LVIDd = diastole left ventricle internal dimension; PWD = diastole posterior wall thickness; Tei index = a myocardial performance index. Data presented as Mean ± SEM; ** = *p* < 0.01 compared to AAV9-cTnT-NC; ## = *p* < 0.01 compared to AAV9-cTnT-NC + Ang II.

**Supplementary Table S5:** siRNA sequences for TRIM40 knockdown in NRVMs cells

| **siRNA** | **Species** | **Sequence** |
| --- | --- | --- |
| TRIM40 #1 | Rat | 5’-CAGGAGUUCGCAACAGAAA-3  5’-UUUCUGUUGCGAACUCCUG-3’ |
| TRIM40 #2 | Rat | 5’-GAAAGACUCAAUCGGAGAA-3  5’-UUCUCCGAUUGAGUCUUUC-3’ |
| TRIM40 #3 | Rat | 5’-CAGUCCUUGGAGACAAUUA-3  5’-UAAUUGUCUCCAAGGACUG-3’ |
| Negative control | Rat | 5’-UUCUCCGAACGUGUCACGUTT-3  5’-ACGUGACACGUUCGGAGAATT-3’ |

**Supplementary Table S6:** Biometric and echocardiographic parameters in Ang II-challenged mice treated with TRIM40-AAV9.

| Model 4 (Ang II model) |  |  | Continuous Ang II Pump Infusion | | |
| --- | --- | --- | --- | --- | --- |
|  | WT+AAV-NC | WT+AAV-TRIM40 | WT+AAV-NC | WT+AAV-TRIM40 | WT+AAV-TRIM40+  PKN1/2-IN-1 |
|  | n=6 | n=6 | n=6 | n=6 | n=6 |
| HR, bpm | 512.50±15.41 | 511.00±11.28 | 517.00±8.67 | 517.83±15.72 | 515.33±19.10 |
| EF, ％ | 65.32±4.04 | 61.92±6.22 | 52.91±3.14 | 43.04±8.13** | 62.22±4.78## |
| FS, ％ | 30.54±2.76 | 28.44±4.15 | 22.88±1.74 | 17.80±4.02** | 28.52±3.11## |
| LVIDd, mm | 2.62±0.21 | 2.85±0.29 | 2.91±0.15 | 3.00±0.15** | 2.75±0.23# |
| IVSD, mm | 0.55±0.11 | 0.73±0.06 | 0.69±0.07 | 0.69±0.05* | 0.67±0.06 |
| PWD, mm | 0.57±0.05 | 0.61±0.04 | 0.80±0.04 | 1.03±0.15** | 0.73±0.04## |
| Tei Index | 0.60±0.04 | 0.83±0.16 | 0.76±0.06 | 0.97±0.09** | 0.71±0.12## |
| IVRT, ms | 13.83±1.47 | 13.67±1.03 | 21.50±1.05 | 25.00±0.89** | 16.50±1.05## |
| HW/BW, mg/g | 5.48±0.38 | 5.67±0.21 | 6.36±0.21 | 6.77±0.30** | 6.08±0.25## |
| HW/TL, mg/mm | 7.07±0.54 | 7.03±0.50 | 7.61±0.28 | 7.81±0.22** | 7.39±0.21# |

Transthoracic echocardiography was performed on mice at the end of the animal study. Ang II = angiotensin II; BW = body weight; EF = ejection fraction; FS = fractional shortening; HR = heart rate; HW = heart weight; IVRT = isovolumic relaxation time; IVSD = diastole interventricular septal thickness; LVIDd = diastole left ventricle internal dimension; PWD = diastole posterior wall thickness; Tei index = a myocardial performance index. Data presented as Mean ± SEM; * = *p* < 0.05 and ** = *p* < 0.01 compared to WT+AAV-NC; # = *p* < 0.05 and ## = *p* < 0.01 compared to WT+AAV-TRIM40 + Ang II.

**Supplementary Table S7:** Biometric and echocardiographic parameters in TAC-challenged mice treated with TRIM40-AAV9.

| Model 5 (TAC model) |  |  | TAC | | |
| --- | --- | --- | --- | --- | --- |
|  | WT+AAV-NC | WT+AAV-TRIM40 | WT+AAV-NC | WT+AAV-TRIM40 | WT+AAV-TRIM40+  PKN1/2-IN-1 |
|  | n=6 | n=6 | n=6 | n=6 | n=6 |
| HR, bpm | 531.83±14.03 | 541.33±22.49 | 542.00±14.58 | 544.17±16.17 | 542.50±16.08 |
| EF, ％ | 63.41±2.95 | 64.62±4.25 | 51.96±2.82 | 43.35±3.89** | 59.72±4.49## |
| FS, ％ | 29.21±2.00 | 30.12±2.85 | 22.37±1.59 | 17.85±1.79** | 26.92±2.90## |
| LVIDd, mm | 2.62±0.19 | 2.83±0.20 | 3.05±0.18 | 3.08±0.12** | 2.77±0.30# |
| IVSD, mm | 0.59±0.03 | 0.60±0.03 | 0.74±0.02 | 0.83±0.02* | 0.72±0.04 |
| PWD, mm | 0.62±0.04 | 0.64±0.02 | 0.81±0.02 | 1.07±0.12** | 0.72±0.06## |
| Tei Index | 0.56±0.08 | 0.61±0.06 | 0.83±0.07 | 1.03±0.07** | 0.68±0.08## |
| IVRT, ms | 13.50±1.38 | 13.33±1.21 | 21.17±2.32 | 24.17±1.17** | 17.50±1.52## |
| HW/BW, mg/g | 5.59±0.33 | 5.67±0.45 | 6.35±0.26 | 6.71±0.24** | 5.96±0.34## |
| HW/TL, mg/mm | 7.00±0.26 | 6.84±0.46 | 7.88±0.34 | 8.24±0.35** | 7.58±0.51# |

Transthoracic echocardiography was performed on mice at the end of the animal study. Ang II = angiotensin II; BW = body weight; EF = ejection fraction; FS = fractional shortening; HR = heart rate; HW = heart weight; IVRT = isovolumic relaxation time; IVSD = diastole interventricular septal thickness; LVIDd = diastole left ventricle internal dimension; PWD = diastole posterior wall thickness; Tei index = a myocardial performance index. Data presented as Mean ± SEM; * = *p* < 0.05 and ** = *p* < 0.01 compared to WT+AAV-NC; # = *p* < 0.05 and ## = *p* < 0.01 compared to WT+AAV-TRIM40 + TAC.

**Supplementary Table S8:** siRNA sequences for TRIM40 knockdown in NRVMs cells

| **siRNA** | **Sequence** |
| --- | --- |
| PKN1 | GCACUGUGCUUAAGCUGGAtt |
| PKN2 | GCACCCAUUUUUCCGGCUAtt |

**Supplementary Table S9:** Primer sequences for qPCR assay.

| Gene | Species | Sequence |
| --- | --- | --- |
| *β-Actin* | Rat | AAGTCCCTCACCCTCCCAAAAG  AAGCAATGCTGTCACCTTCCC |
| *Myh7* | Rat | GAGGAGAGGGCGGACATT  ACTCTTCATTCAGGCCCTTG |
| *Anp* | Rat | AGGGTCTGGGGGAGGGAGGGTTACT  CTCCCCATTCTGTCACTTGCGGCGG |
| *Bnp* | Rat | TCTGGGGGAGGGAGGGTTACTGGAG  AACCCTCCCCATTCTGTCACTTGCG |
| *Il1b* | Rat | CACCTCTCAAGCAGAGCACAG  GGGTTCCATGGTGAAGTCAAC |
| *Il6* | Rat | GAGTTGTGCAATGGCAATTC  ACTCCAGAAGACCAGAGCAG |
| *Tnf* | Rat | TACTCCCAGGTTCTCTTCAAGG  GGAGGCTGACTTTCTCCTGGTA |
| *β-Actin* | Mouse | CCGTGAAAAGATGACCCAGA TACGACCAGAGGCATACAG |
| *Myh7* | Mouse | CAAAGGCAAGGCAAAGAAAG  TCACCCCTGGAGACTTTGTC |
| *Anp* | Mouse | AAGAACCTGCTAGACCACCTGGAG  TGCTTCCTCAGTCTGCTCACTCAG |
| *Bnp* | Mouse | GAGGTCACTCCTATCCTCTGG  GCCATTTCCTCCGACTTTTCTC |
| *Il1b* | Mouse | TCGCAGCAGCACATCAACAAGAG  AGGTCCACGGGAAAGACACAGG |
| *Il6* | Mouse | GAGGATACCACTCCCAACAGACC  AAGTGCATCATCGTTGTTCATACA |
| *Tnf* | Mouse | TGATCCGCGACGTGGAA  ACCGCCTGGAGTTCTGGAA |


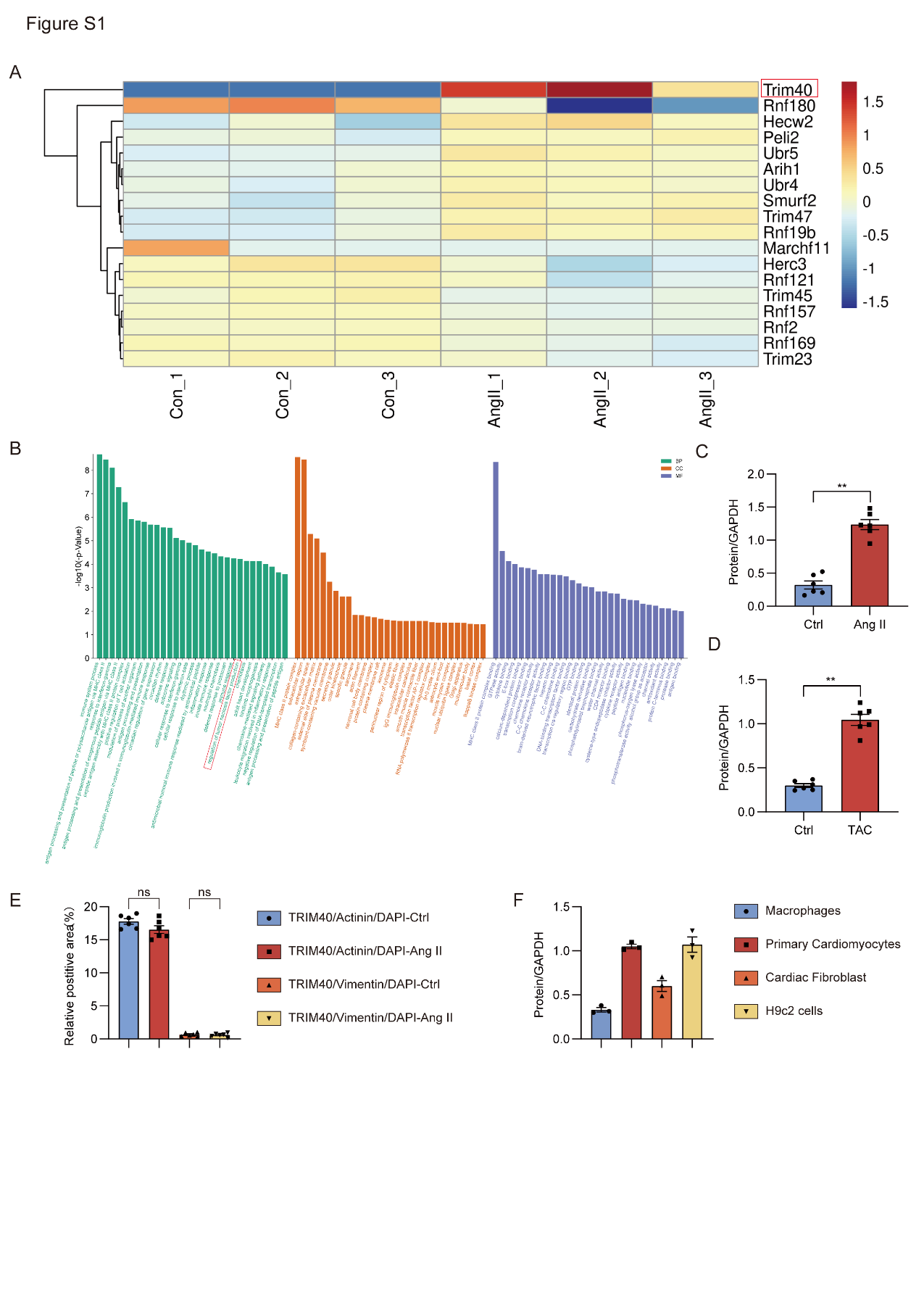


**Supplementary Figure S1: TRIM40 is induced in Ang II-mediated cardiac hypertrophy and fibrosis model. (A)** RNA sequencing was performed on wild-type C57BL/6 control mice and mice challenged with Ang II for 4 weeks. The heatmap shows the top 18 most differentially expressed E3 ubiquitin ligases (screening criterion: *p* < 0.05 for Con vs. Ang II). **(B)** Gene Ontology (GO) enrichment analysis of differentially expressed genes between the CON and Ang II groups*.* **(C)** Densitometric quantification of the Western blot bands from Figure 1B (n = 6). **(D)** Densitometric quantification of the Western blot bands from Figure 1C (n = 6). **(E)** Quantification of TRIM40 fluorescence intensity in co-localized areas with cardiomyocyte (Actinin) or fibroblast (Vimentin) markers under Ctrl or Ang II treatment (n = 6). **(F)** Densitometric quantification of the Western blot bands from Figure 1E (n = 3). All quantitative data are presented as mean ± SEM. Data between two groups were compared by independent-sample two-tailed Student’s t-test. Data among multiple groups were compared by one-way ANOVA test, followed by Tukey post hoc test. ns indicates not statistically significant; ***p* < 0.01.


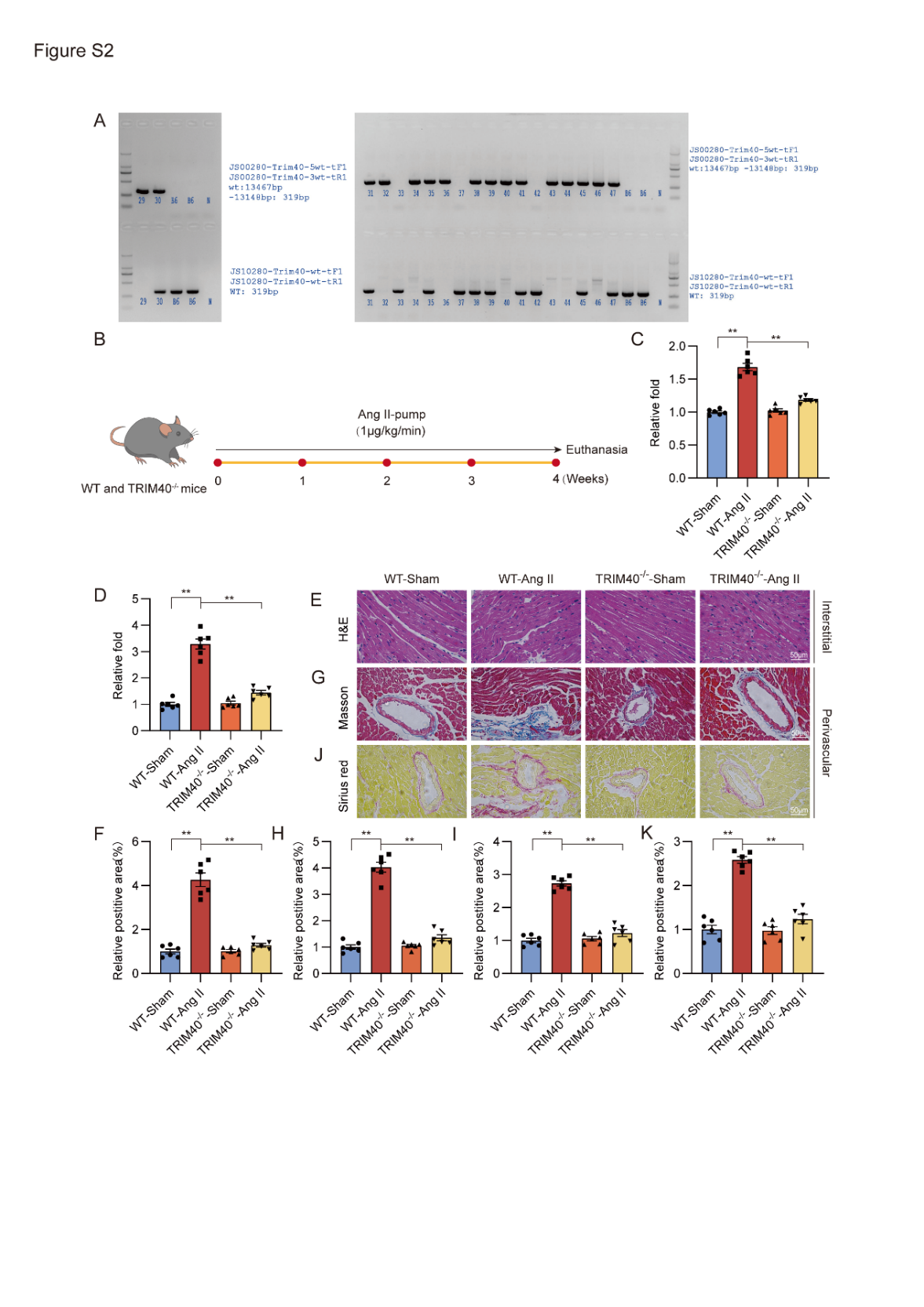


**Supplementary Figure S2:** **TRIM40 deficiency alleviates Ang II-induced cardiac remodeling. (A)** Genotyping identification of TRIM40 knockout mice (TRIM40⁻^⁄^⁻). **(B)** Study flowchart in mice illustrating Ang II treatment. **(C)** Quantification of heart size from representative heart specimen photographs in Figure 2I (n = 6). **(D)** Quantification of cardiomyocyte size from WGA staining in Figure 2J (n = 6). **(E)** H&E staining of mouse heart tissue (scale bar = 50 μm) (n = 6). **(F)** Quantification of interstitial fibrosis based on representative Masson’s trichrome-stained sections of heart tissue shown in Figure 2L (n = 6). **(G, H)** Perivascular fibrosis was detected by staining heart tissues with Masson’s trichrome (G) (scale bar = 50 μm). Quantification is shown in panel H (n = 6). **(I)** Quantification of Picro Sirius Red staining of heart tissue in Figure 2M (n = 6). **(J, K)** Picro Sirius Red staining and quantification of perivascular fibrosis sin heart tissues of mice. Representative staining images are shown in Panel J (scale bar = 50 μm). Quantification is shown in Panel K (n = 6). All quantitative data are presented as mean ± SEM. Data between two groups were compared by independent-sample two-tailed Student’s t-test. Data among multiple groups were compared by one-way ANOVA test, followed by Tukey post hoc test; ***p* < 0.01.


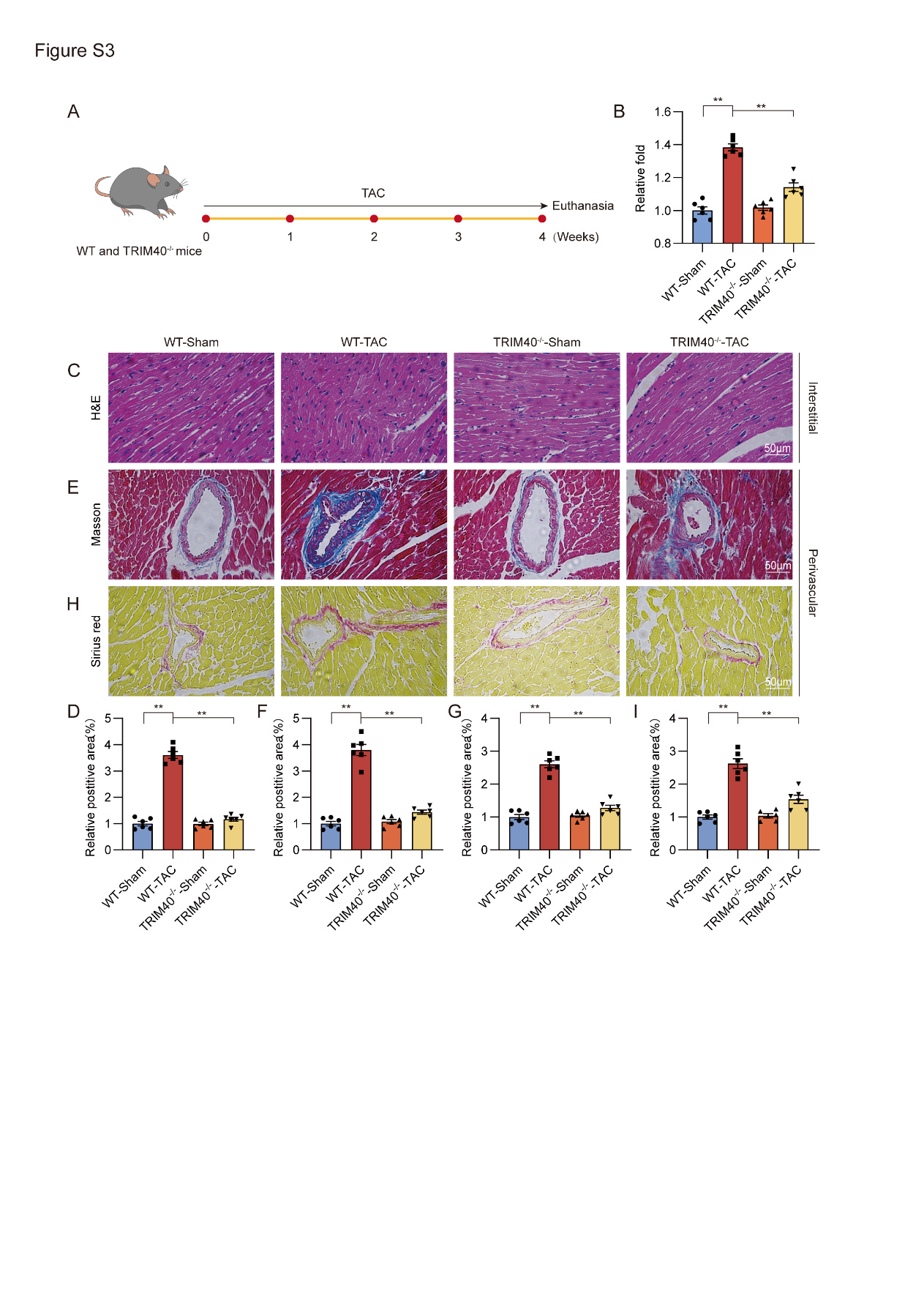


**Supplementary Figure S3: TRIM40 deficiency alleviates TAC-induced cardiac remodeling.** **(A)** Study flowchart in mice illustrating TAC treatment. **(B)** Quantification of heart size from representative heart specimen photographs in Figure 3G (n = 6). **(C)** H&E staining of mouse cardiac interstitial tissue (scale bar = 50 μm) (n = 6). **(D)** Quantification of interstitial fibrosis by Masson's trichrome staining of cardiac tissue, with representative images shown in Figure 3K (n = 6). **(E, F)** Perivascular fibrosis was detected by staining heart tissues with Masson’s trichrome (E) (scale bar = 50 μm). Quantification is shown in panel F (n = 6). **(G)** Quantitative analysis of Picro Sirius Red staining of cardiac tissue in Figure 3L (n = 6). **(H, I)** Perivascular fibrosis was detected in heart tissues by Picro Sirius Red staining (H) (scale bar = 50 μm). Quantification of staining is shown in Panel I (n = 6). All quantitative data are presented as mean ± SEM. Data between two groups were compared by independent-sample two-tailed Student’s t-test. Data among multiple groups were compared by one-way ANOVA test, followed by Tukey post hoc test; ***p* < 0.01.


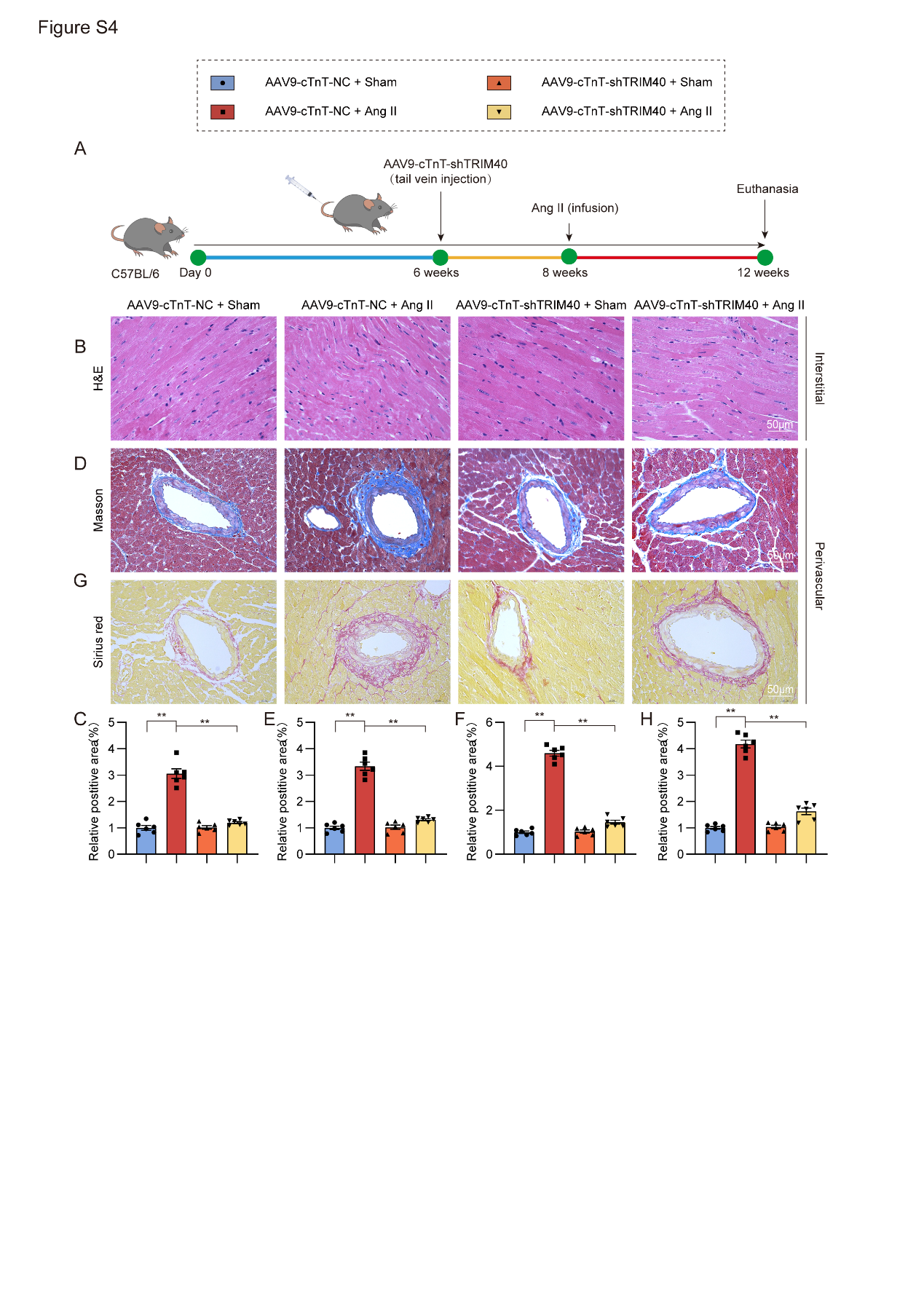


**Supplementary Figure S4. Cardiomyocyte-specific knockdown of TRIM40 alleviates Ang II-induced cardiac remodeling. (A)** Schematic of the experimental design: cardiomyocyte-specific knockdown via AAV9-cTnT-shTRIM40 followed by 4-week Ang II infusion. **(B)** H&E staining of mouse cardiac interstitial tissue (scale bar = 50 μm) (n = 6). **(C)** Quantification of interstitial fibrosis by Masson's trichrome staining of cardiac tissue, with representative images shown in Figure 4N (n = 6). **(D, E)** Perivascular fibrosis was detected by staining heart tissues with Masson’s trichrome (D) (scale bar = 50 μm). Quantification is shown in panel E (n = 6). **(F)** Quantitative analysis of Picro Sirius Red staining of cardiac tissue in Figure 4O (n = 6). **(G, H)** Perivascular fibrosis was detected in heart tissues by Picro Sirius Red staining (G) (scale bar = 50 μm). Quantification of staining is shown in Panel H (n = 6). All quantitative data are presented as mean ± SEM. Data between two groups were compared by independent-sample two-tailed Student’s t-test. Data among multiple groups were compared by one-way ANOVA test, followed by Tukey post hoc test; ***p* < 0.01.


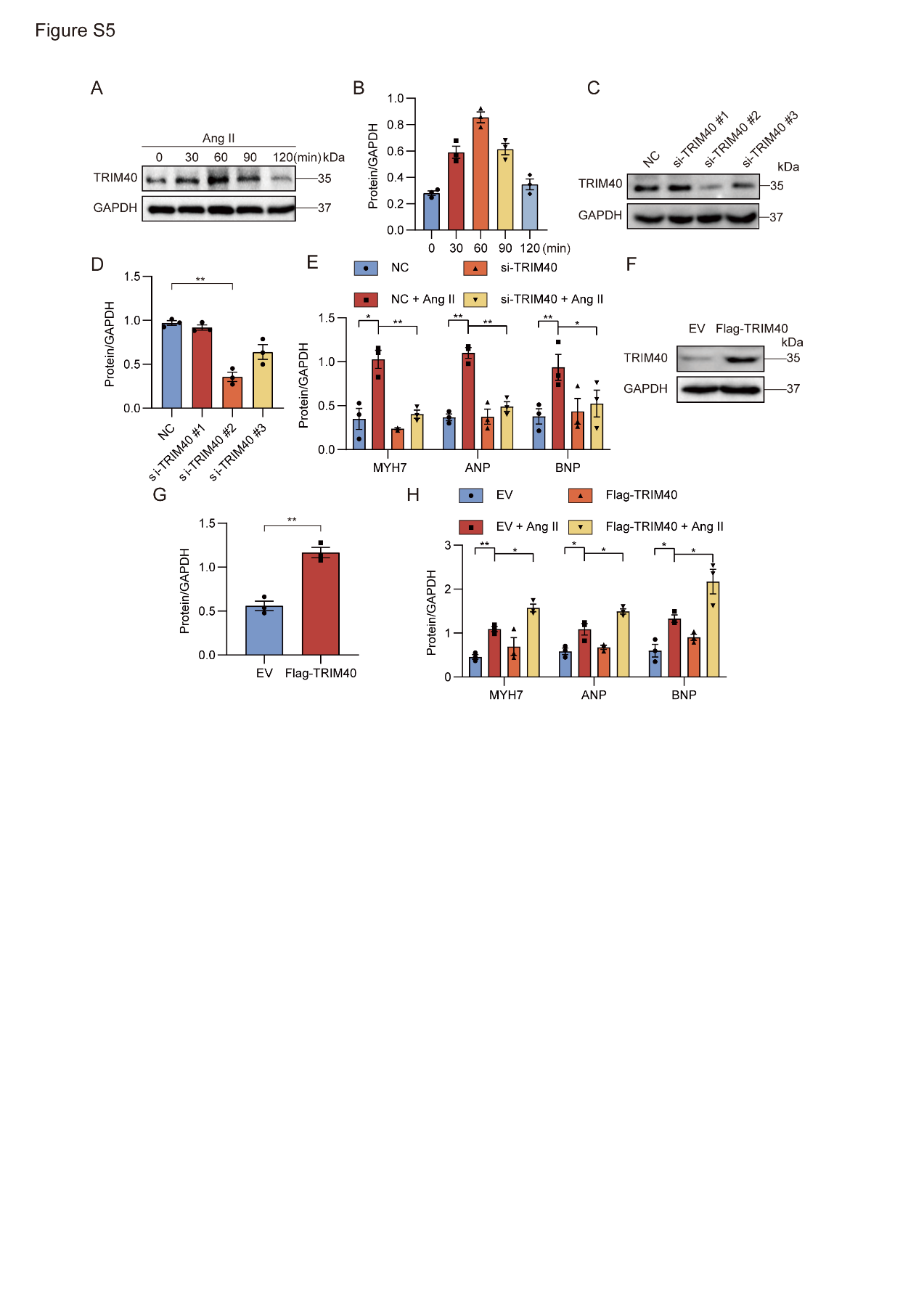


**Supplementary Figure S5: TRIM40 mediated Ang II-induced cardiomyocyte hypertrophy *in vitro*.** **(A)** Representative Western blot analysis of TRIM40 protein expression in NRVMs stimulated with Ang II at various time points. GAPDH was used as a loading control (n = 3). **(B)** Densitometric quantification of TRIM40 immunoblots from panel A (n = 3). **(C, D)** TRIM40 knockdown in NRVMs was performed using si-TRIM40, with scrambled siRNA transfections as NC. TRIM40 protein levels were detected by immunoblotting (C) and densitometric quantification (D) at 48 hours post-transfection (n = 3). **(E)** Densitometric quantification of immunoblots in Figure 5C (n = 3). **(F, G)** Overexpression of TRIM40 in NRVMs was achieved using a Flag-TRIM40, with EV transfections as control. TRIM40 protein expression was analyzed by immunoblotting (F) and densitometric quantification (G) at 48 hours post-transfection (n = 3). **(H)** Densitometric quantification of immunoblots in Figure 5H (n = 3). All quantitative data are presented as mean ± SEM. Data between two groups were compared by independent-sample two-tailed Student’s t-test. Data among multiple groups were compared by one-way ANOVA test, followed by Tukey post hoc test; **p* < 0.05, ***p* < 0.01.


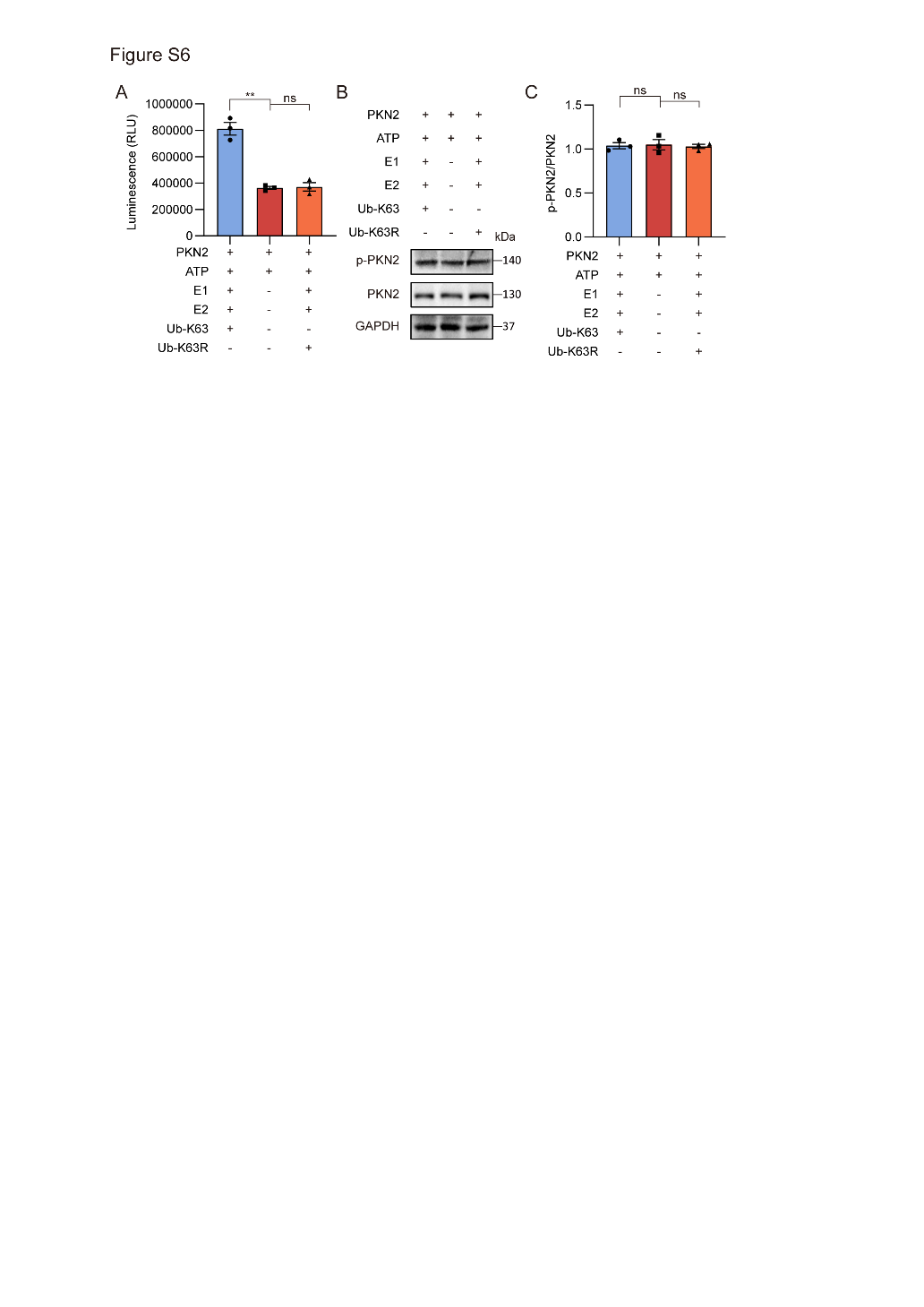


**Supplementary Figure S6:** **K63-linked polyubiquitination directly allosterically activates PKN2.** **(A)** The in vitro kinase activity of PKN2 was measured using the ADP-Glo™ kinase assay (n = 3). **(B)** Representative Western blot image of the in vitro kinase reaction products, detected with an anti-p-PKN2 antibody to assess the autophosphorylation level of PKN2 at Ser815 (n = 3). **(C)** Quantitative analysis of the Ser815 phosphorylation signals from (B) (n = 3). All quantitative data are presented as mean ± SEM. Data between two groups were compared by independent-sample two-tailed Student’s t-test. Data among multiple groups were compared by one-way ANOVA test, followed by Tukey post hoc test. ns indicates not statistically significant; ***p* < 0.01.


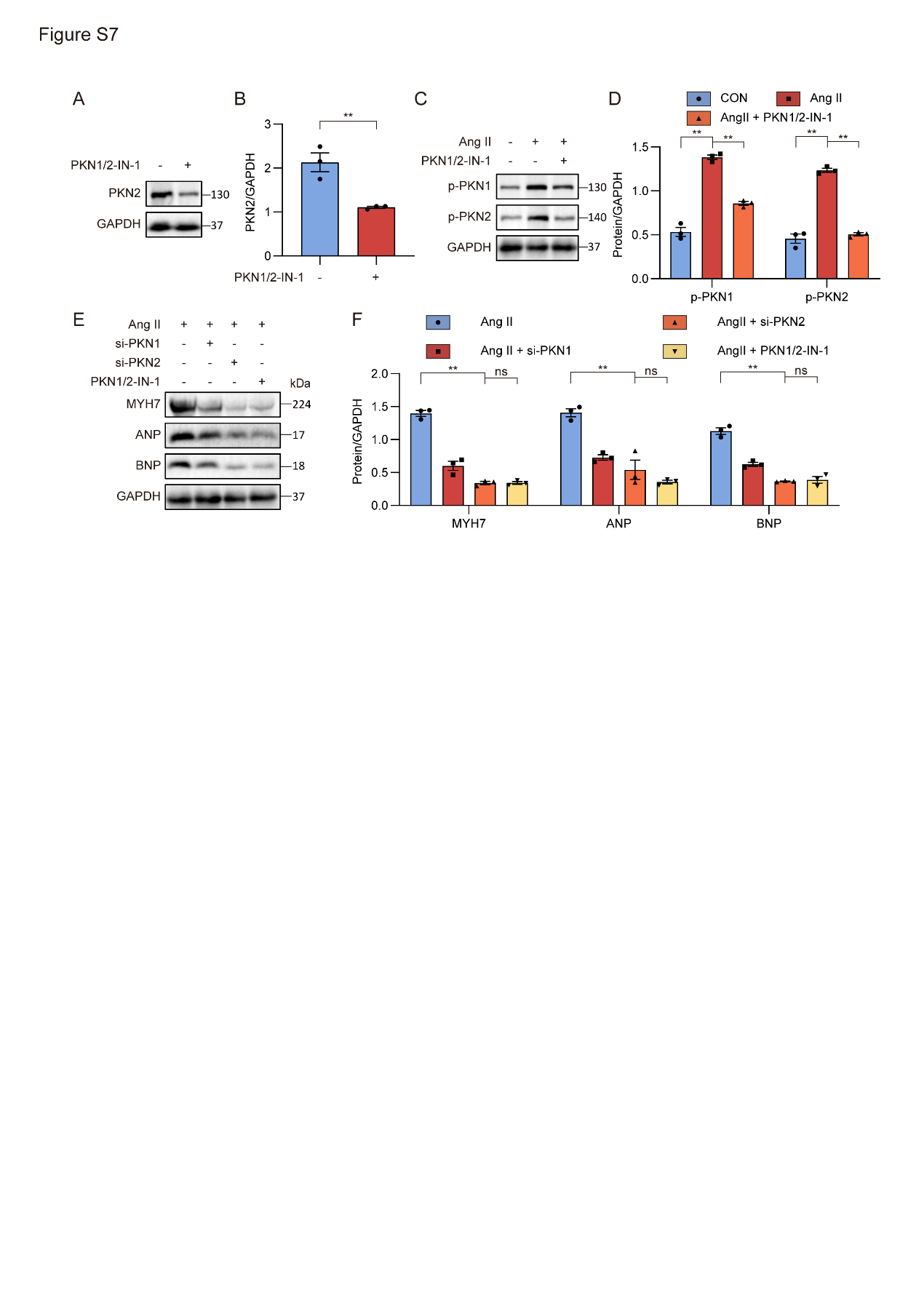


**Supplementary Figure S7: Systematic validation of the specificity of PKN2 inhibitor. (A)** Representative Western blot analysis of PKN2 expression in NRVMs treated with or without the dual inhibitor PKN1/2-IN-1. GAPDH was used as a loading control (n = 3). **(B)** Densitometric quantification of PKN2 protein levels from panel A (n = 3). **(C)** Representative Western blot analysis of p-PKN1 and p-PKN2 in NRVMs. GAPDH was used as a loading control (n = 3). **(D)** Densitometric quantification of p-PKN1 and p-PKN2 protein levels from panel C. **(E)** Representative Western blot analysis of hypertrophy markers (MYH7, ANP, BNP) in NRVMs. GAPDH was used as a loading control (n = 3). **(F)** Densitometric quantification of MYH7, ANP, and BNP protein levels from panel E (n = 3). All quantitative data are presented as mean ± SEM. Data between two groups were compared by independent-sample two-tailed Student’s t-test. Data among multiple groups were compared by one-way ANOVA test, followed by Tukey post hoc test. ns indicates not statistically significant; ***p* < 0.01.


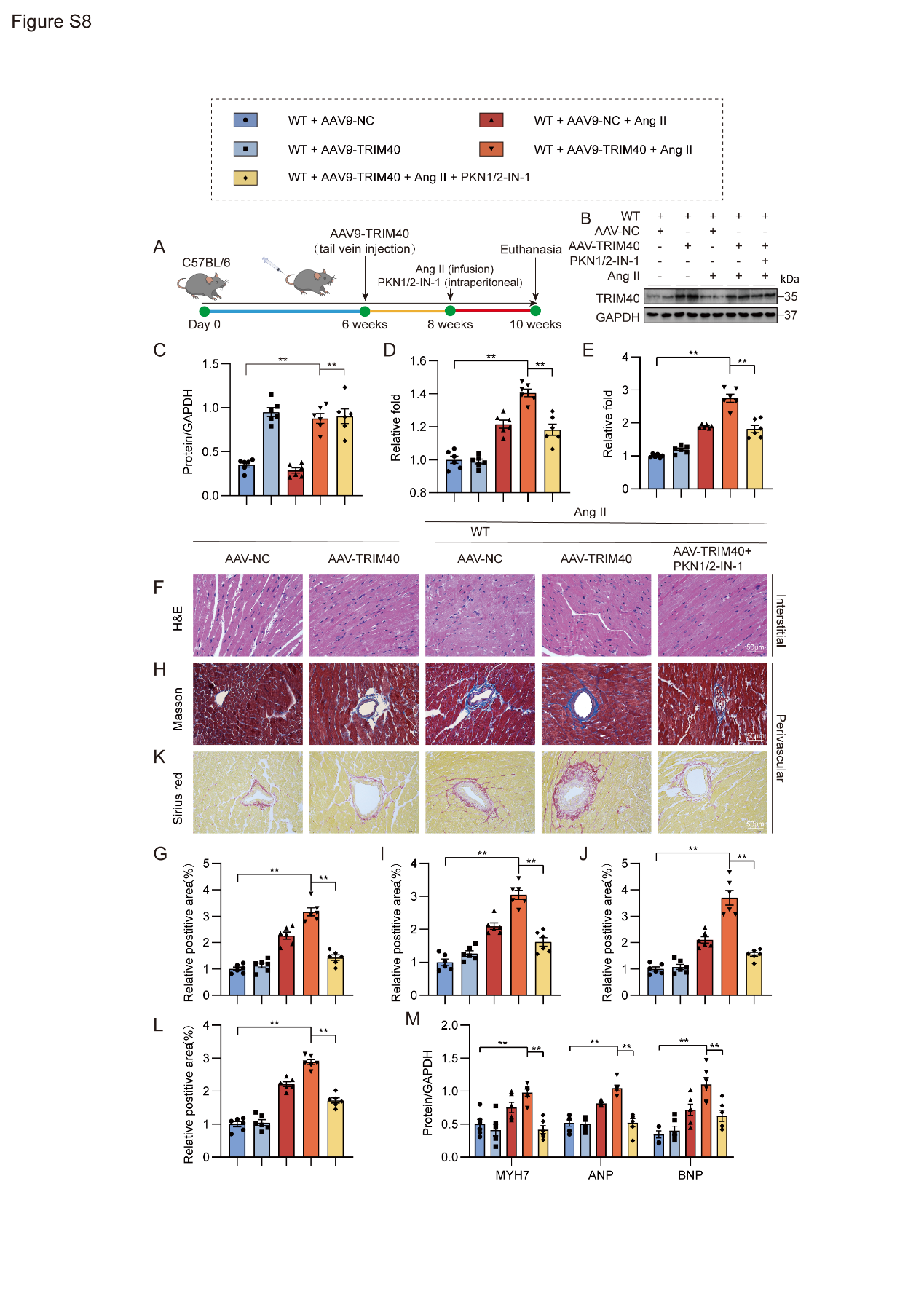


**Supplementary Figure S8: TRIM40 prevents Ang II-induced myocardial hypertrophy and fibrosis by regulating PKN2.** C57BL/6 mice received two injections of AAV9 encoding TRIM40 at one-month intervals, followed by continuous infusion of saline or Ang II for two weeks. **(A)** The flow chart of Ang II model with AAV9-TRIM40 injection and PKN1/2-IN-1 treatment. **(B, C)** TRIM40 protein levels in heart tissues were measured by immunoblotting. Representative blots are shown in Panel B and densitometric quantification is presented in Panel C (n = 6). **(D)** Quantification of heart size from representative heart specimen photographs in Figure 8I (n = 6). **(E)** Quantification of cardiomyocyte size from WGA staining in Figure 8J (n = 6). **(F)** H&E staining of heart tissue (scale bar = 50 μm) (n = 6). **(G)** Quantification of interstitial fibrosis based on representative Masson’s trichrome-stained sections of heart tissue shown in Figure 8L (n = 6). **(H, I)** Perivascular fibrosis was detected by staining heart tissues with Masson’s trichrome (H) (scale bar = 50 μm). Quantification is shown in panel I (n = 6). **(J)** Quantitative analysis of Picro Sirius Red staining of heart tissue in Figure 8M (n = 6). **(K, L)** Perivascular fibrosis was detected in heart tissues by Picro Sirius Red staining (K) (scale bar = 50 μm). Quantification of staining is shown in Panel L (n = 6). **(M)** Densitometric quantification of immunoblots in Figure 8N (n = 6). All quantitative data are presented as mean ± SEM. Data between two groups were compared by independent-sample two-tailed Student’s t-test. Data among multiple groups were compared by one-way ANOVA test, followed by Tukey post hoc test; ***p* < 0.01.


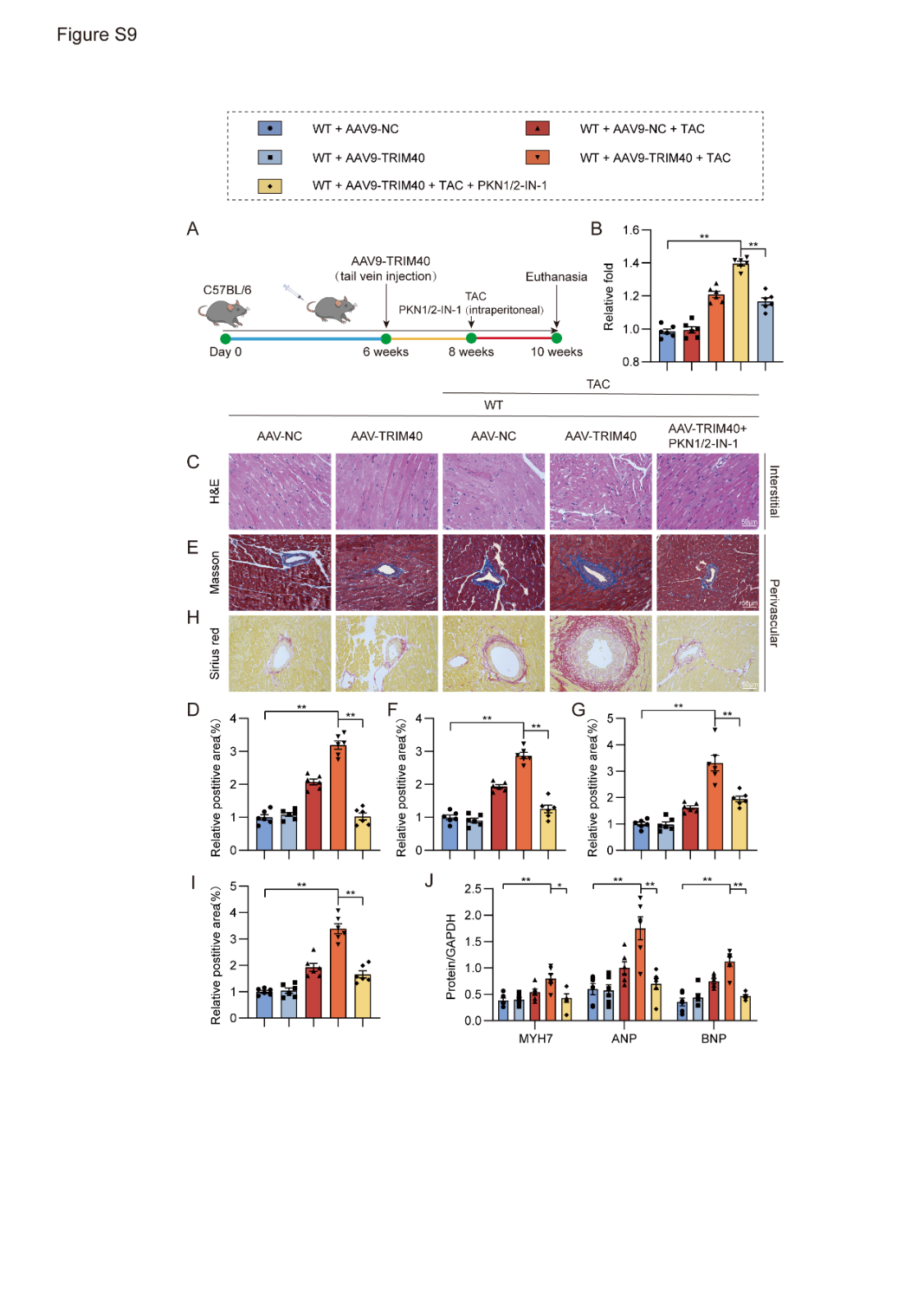


**Supplementary Figure S9: TRIM40 prevents TAC-induced myocardial hypertrophy and fibrosis by regulating PKN2.** C57BL/6 mice received two injections of AAV9 encoding TRIM40 at one-month intervals, followed by sham surgery or TAC surgery for two weeks. **(A)** The flow chart of TAC model with AAV9-TRIM40 injection and PKN1/2-IN-1 treatment. **(B)** Quantification of heart size from representative heart specimen photographs in Figure 9G (n = 6). **(C)** H&E staining of heart tissue (scale bar = 50 μm) (n = 6). **(D)** Quantification of interstitial fibrosis from Masson’s trichrome-stained heart tissue sections shown in Figure 9K (n = 6). **(E, F)** Perivascular fibrosis was detected by staining heart tissues with Masson’s trichrome (E) (scale bar = 50 μm). Quantification is shown in panel F (n = 6). **(G)** Quantitative analysis of Picro Sirius Red staining of heart tissue in Figure 9L (n = 6). **(H, I)** Perivascular fibrosis was detected in heart tissues by Picro Sirius Red staining (H) (scale bar = 50 μm). Quantification of staining is shown in Panel I (n = 6). **(J)** Densitometric quantification of blots in Figure 9M (n = 6). All quantitative data are presented as mean ± SEM. Data between two groups were compared by independent-sample two-tailed Student’s t-test. Data among multiple groups were compared by one-way ANOVA test, followed by Tukey post hoc test; ***p* < 0.01.
