# Supplementary figures and images for: TRIM40 Drives Pathological Cardiac Hypertrophy and Heart Failure via Ubiquitination of PKN2 (part 1 of 3)
Source: Adv Sci (Weinh). 2026 Jan 22;13(17):e21337. doi: 10.1002/advs.202521337 (PMC13042792; doi:10.1002/advs.202521337)

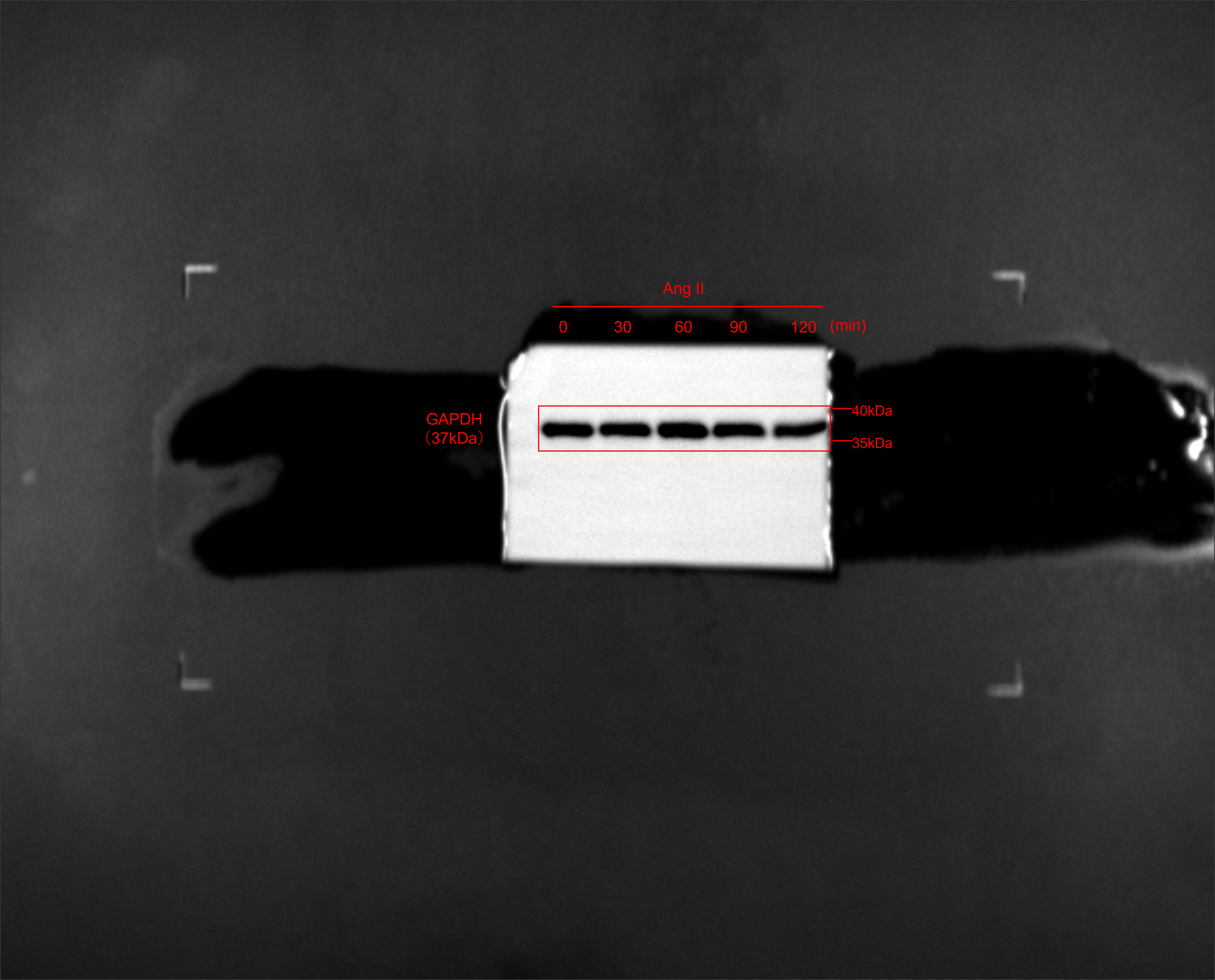

Supplement: Supplementary file 2 — Supporting File 2: advs73796‐sup‐0002‐Supplementary Figures_Raw_Data_Figures.zip. [file ADVS-13-e21337-s001.zip › Figure S5A_Raw_Data_Figures/GAPDH.tif]

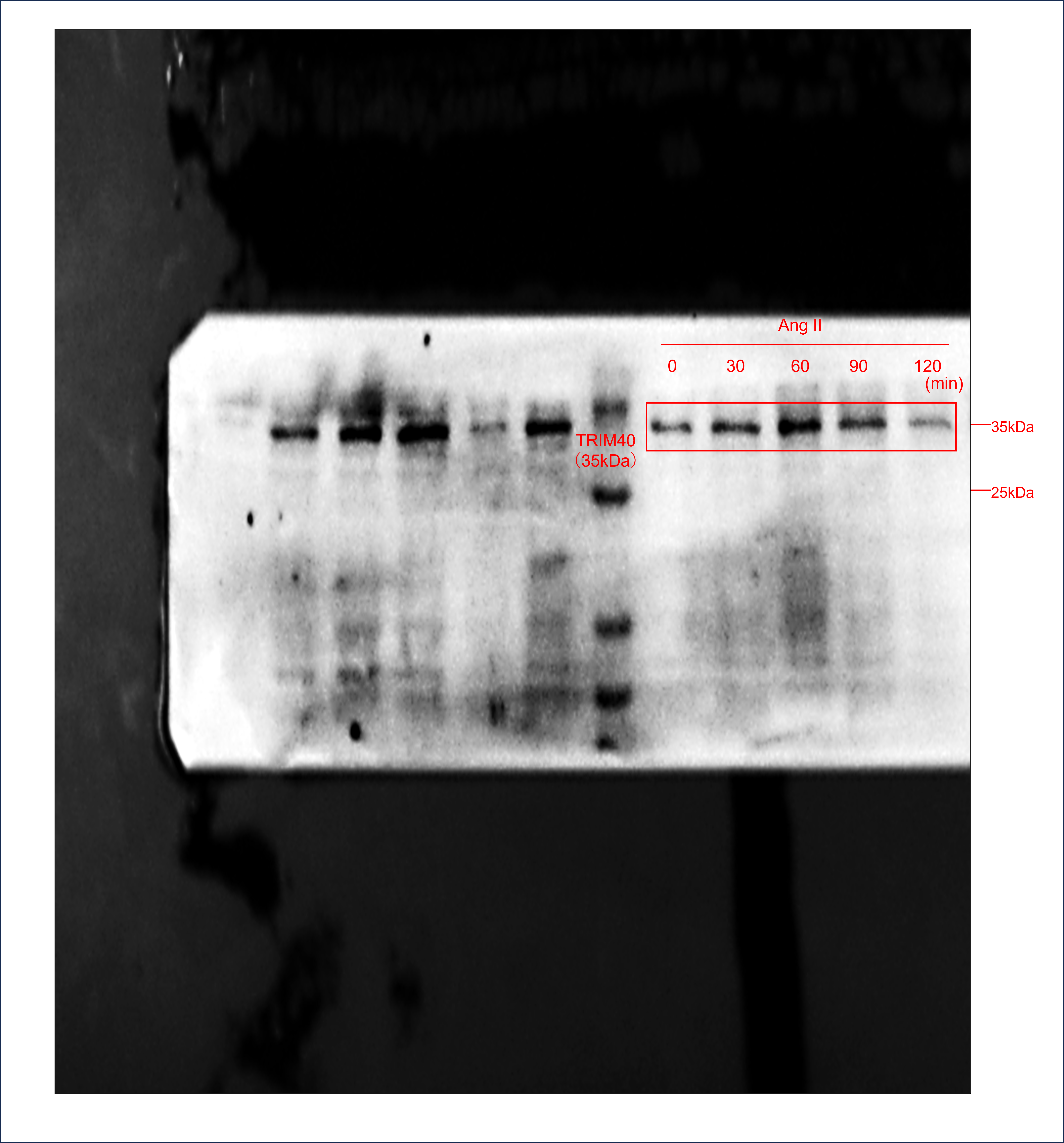

Supplement: Supplementary file 2 — Supporting File 2: advs73796‐sup‐0002‐Supplementary Figures_Raw_Data_Figures.zip. [file ADVS-13-e21337-s001.zip › Figure S5A_Raw_Data_Figures/TRIM40.tif]

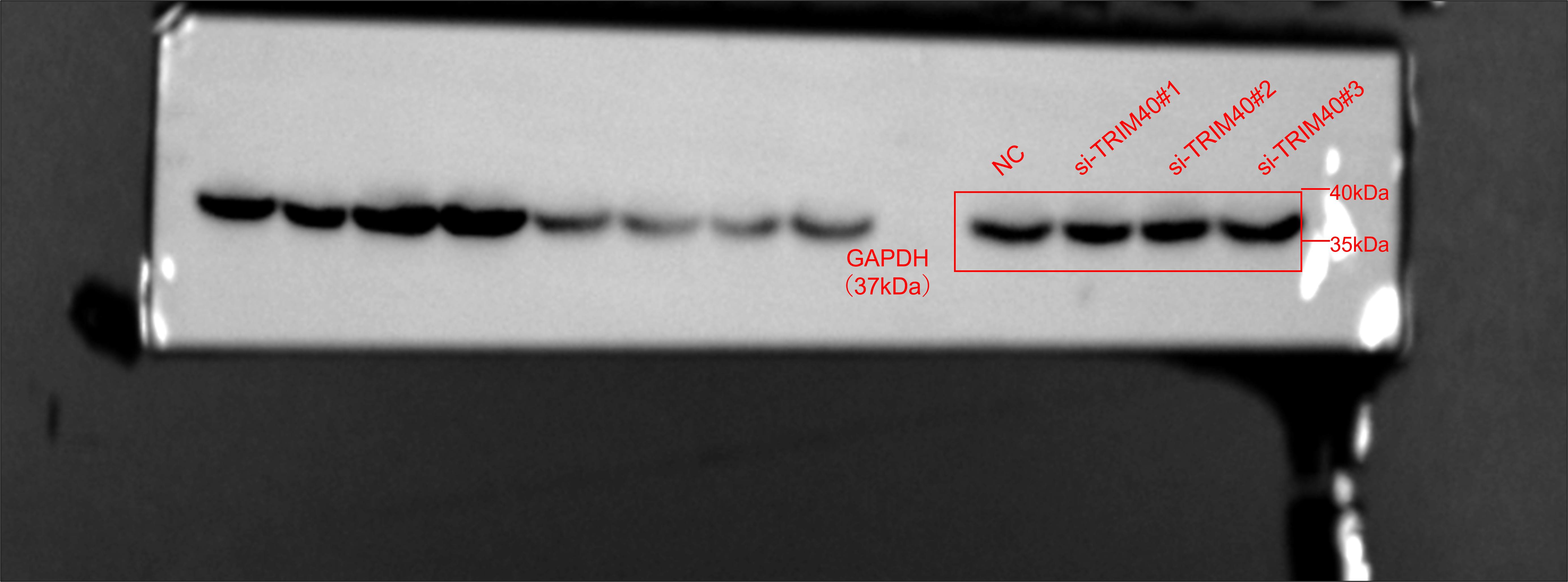

Supplement: Supplementary file 2 — Supporting File 2: advs73796‐sup‐0002‐Supplementary Figures_Raw_Data_Figures.zip. [file ADVS-13-e21337-s001.zip › Figure S5C_Raw_Data_Figures/GAPDH.tif]

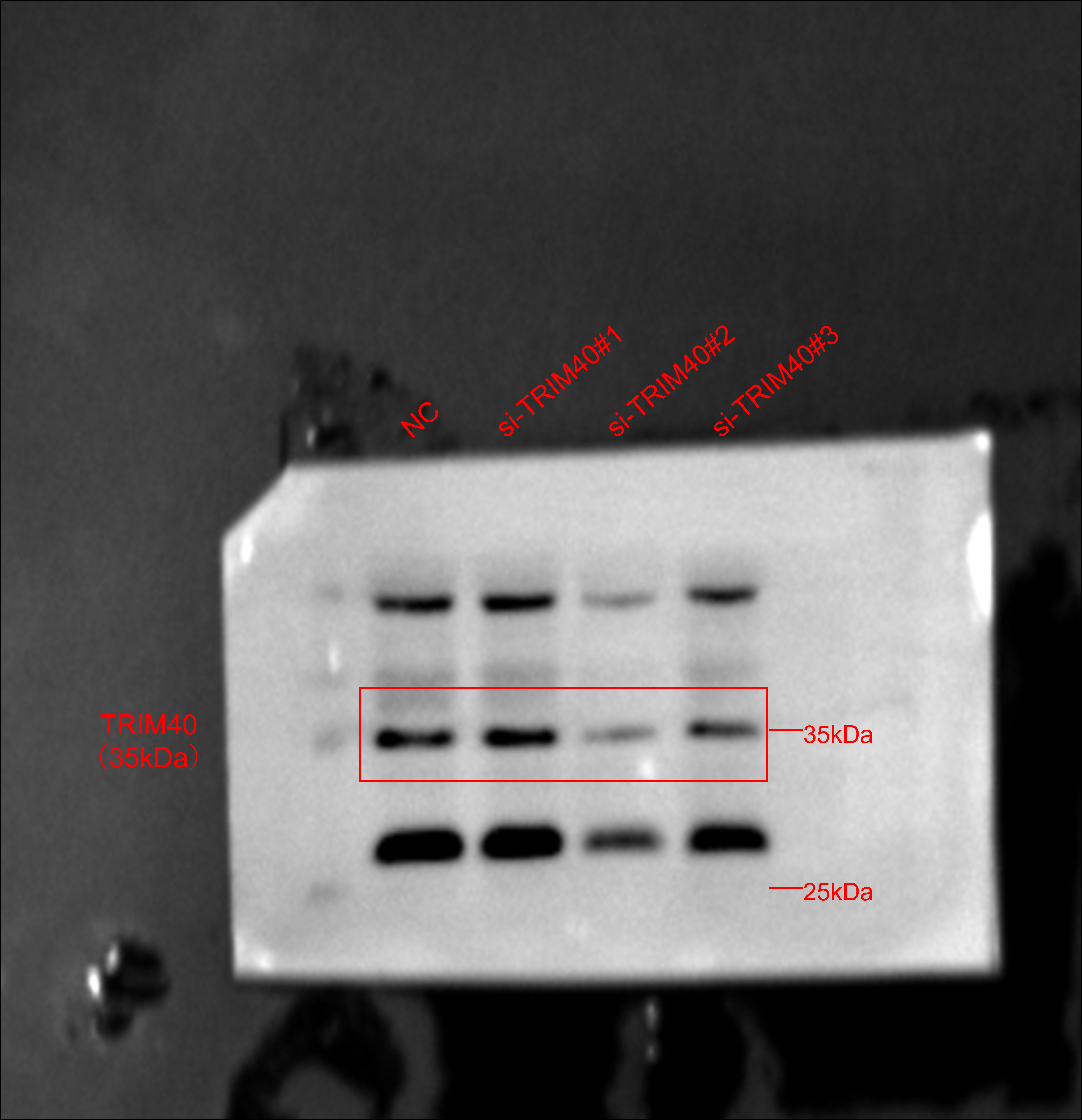

Supplement: Supplementary file 2 — Supporting File 2: advs73796‐sup‐0002‐Supplementary Figures_Raw_Data_Figures.zip. [file ADVS-13-e21337-s001.zip › Figure S5C_Raw_Data_Figures/TRIM40.tif]

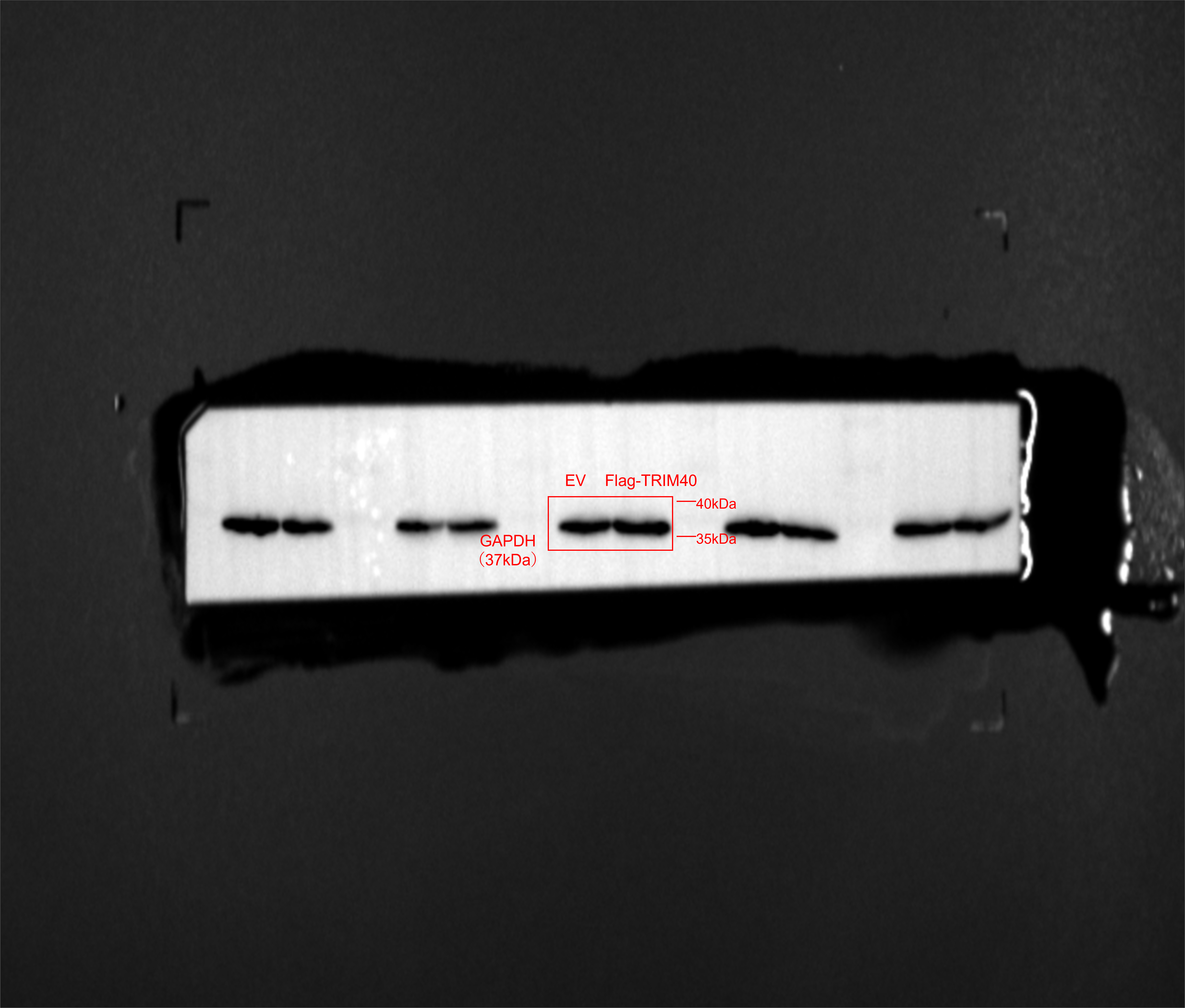

Supplement: Supplementary file 2 — Supporting File 2: advs73796‐sup‐0002‐Supplementary Figures_Raw_Data_Figures.zip. [file ADVS-13-e21337-s001.zip › Figure S5F_Raw_Data_Figures/GAPDH.tif]

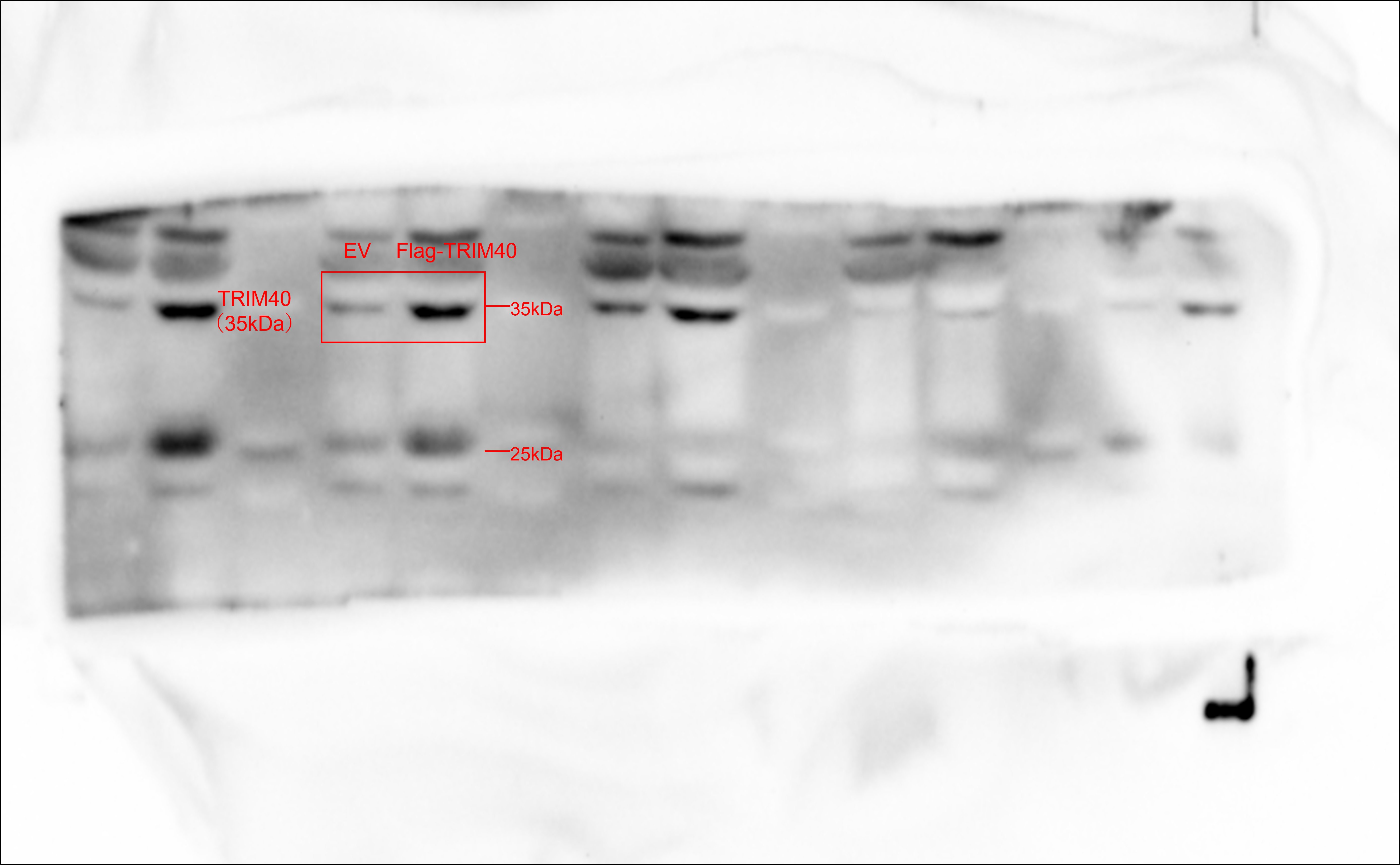

Supplement: Supplementary file 2 — Supporting File 2: advs73796‐sup‐0002‐Supplementary Figures_Raw_Data_Figures.zip. [file ADVS-13-e21337-s001.zip › Figure S5F_Raw_Data_Figures/TRIM40.tif]

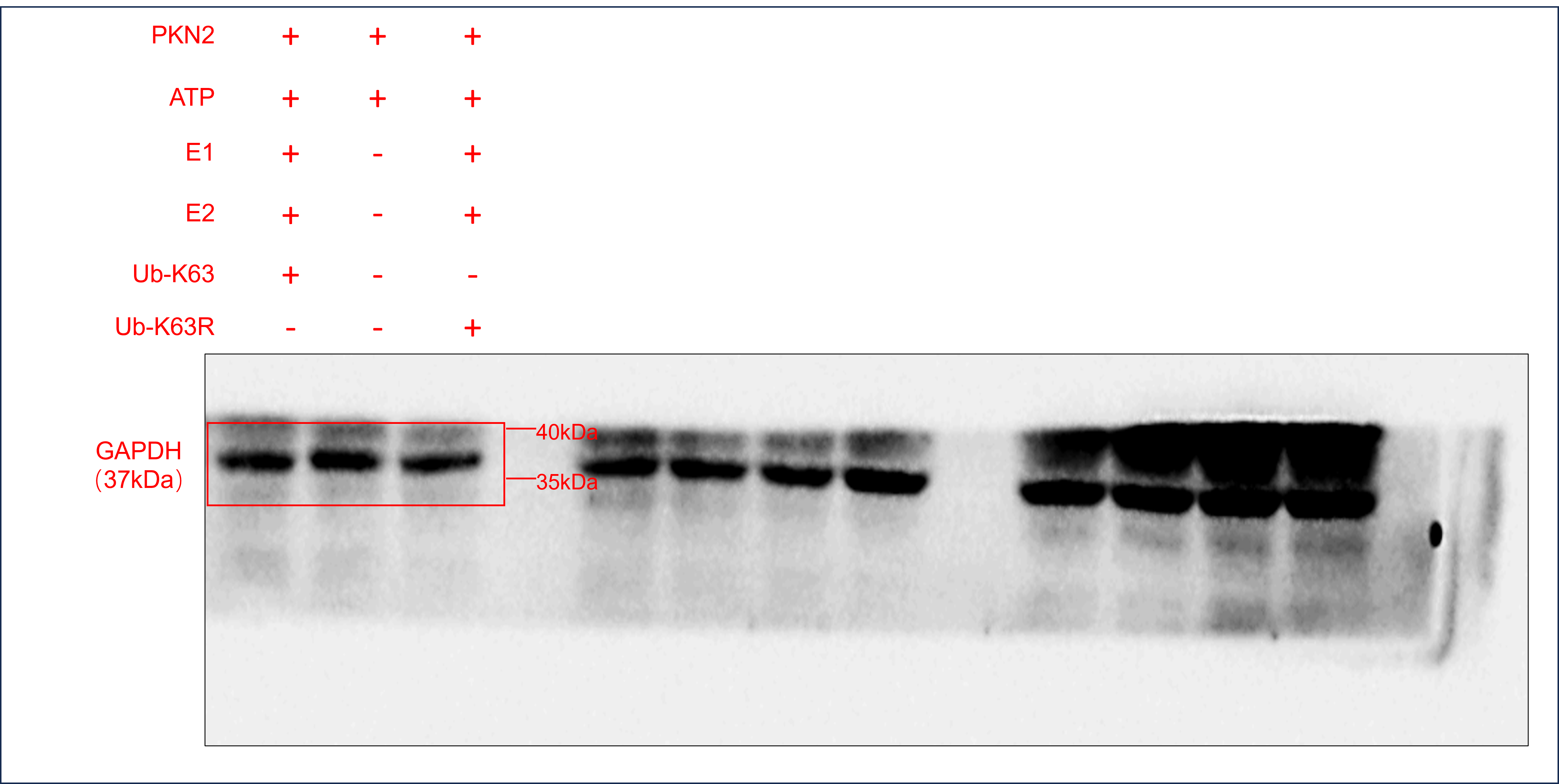

Supplement: Supplementary file 2 — Supporting File 2: advs73796‐sup‐0002‐Supplementary Figures_Raw_Data_Figures.zip. [file ADVS-13-e21337-s001.zip › Figure S6B_Raw_Data_Figures/GAPDH.tif]

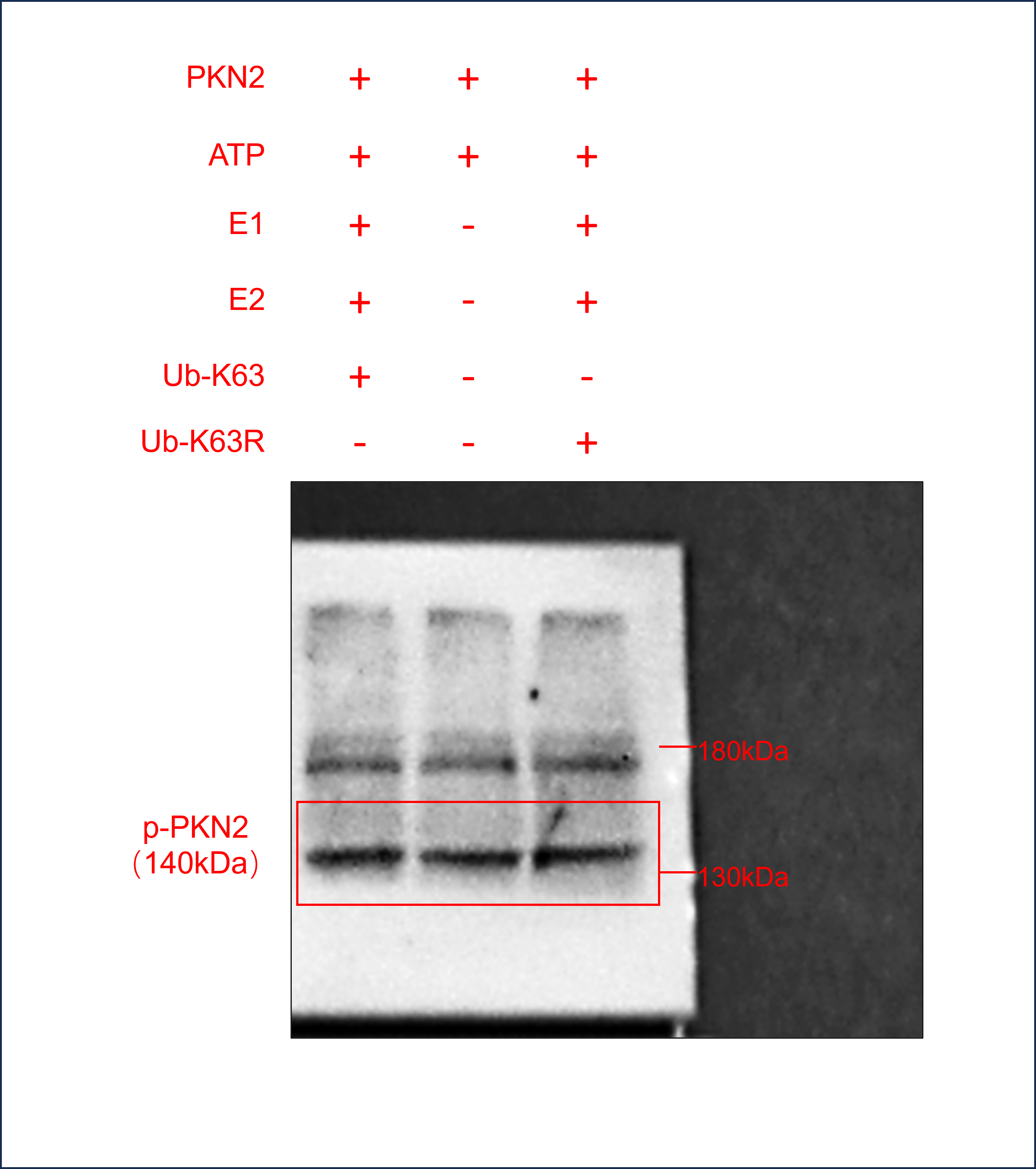

Supplement: Supplementary file 2 — Supporting File 2: advs73796‐sup‐0002‐Supplementary Figures_Raw_Data_Figures.zip. [file ADVS-13-e21337-s001.zip › Figure S6B_Raw_Data_Figures/p-PKN2.tif]

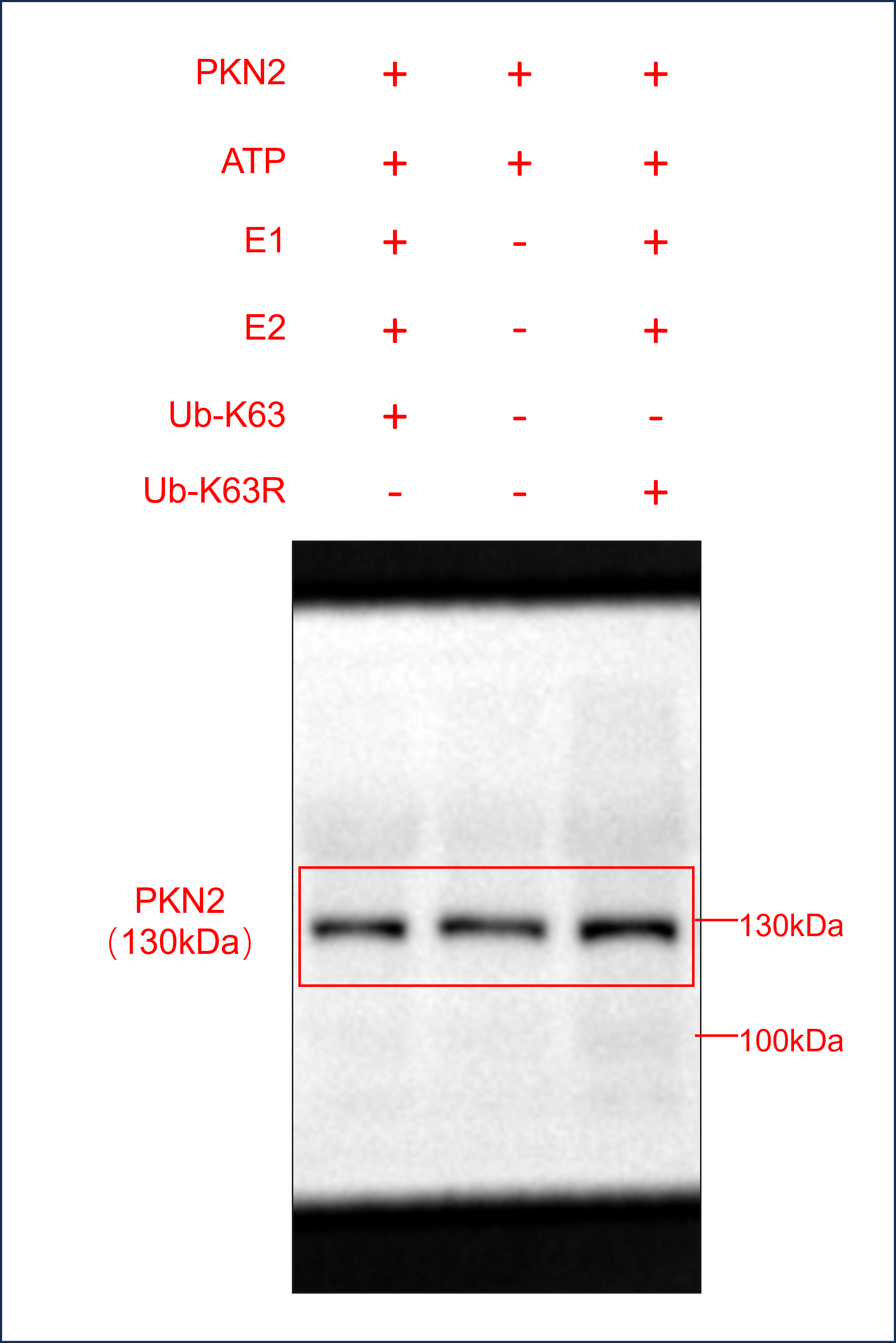

Supplement: Supplementary file 2 — Supporting File 2: advs73796‐sup‐0002‐Supplementary Figures_Raw_Data_Figures.zip. [file ADVS-13-e21337-s001.zip › Figure S6B_Raw_Data_Figures/PKN2.tif]

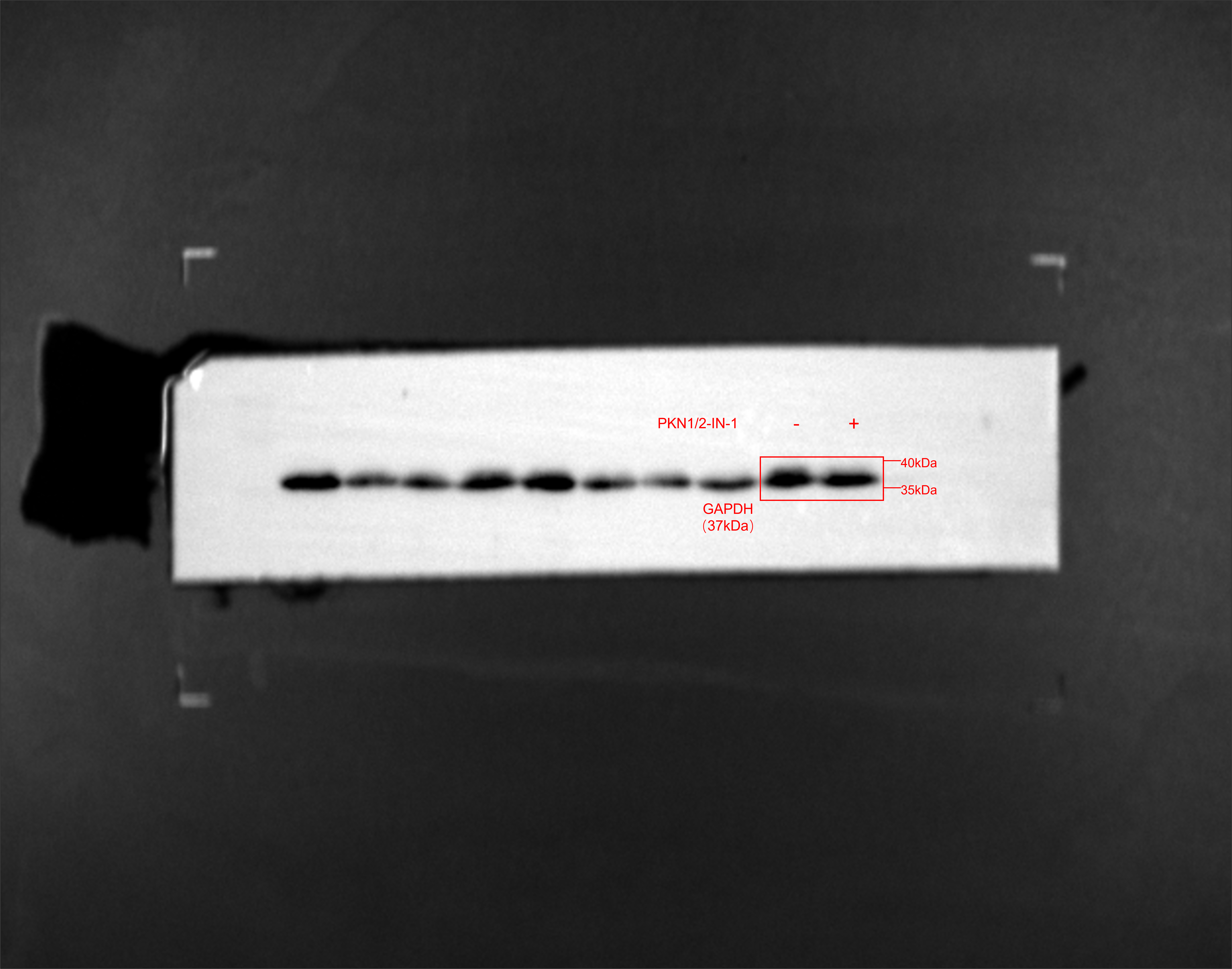

Supplement: Supplementary file 2 — Supporting File 2: advs73796‐sup‐0002‐Supplementary Figures_Raw_Data_Figures.zip. [file ADVS-13-e21337-s001.zip › Figure S7A_Raw_Data_Figures/GAPDH.tif]

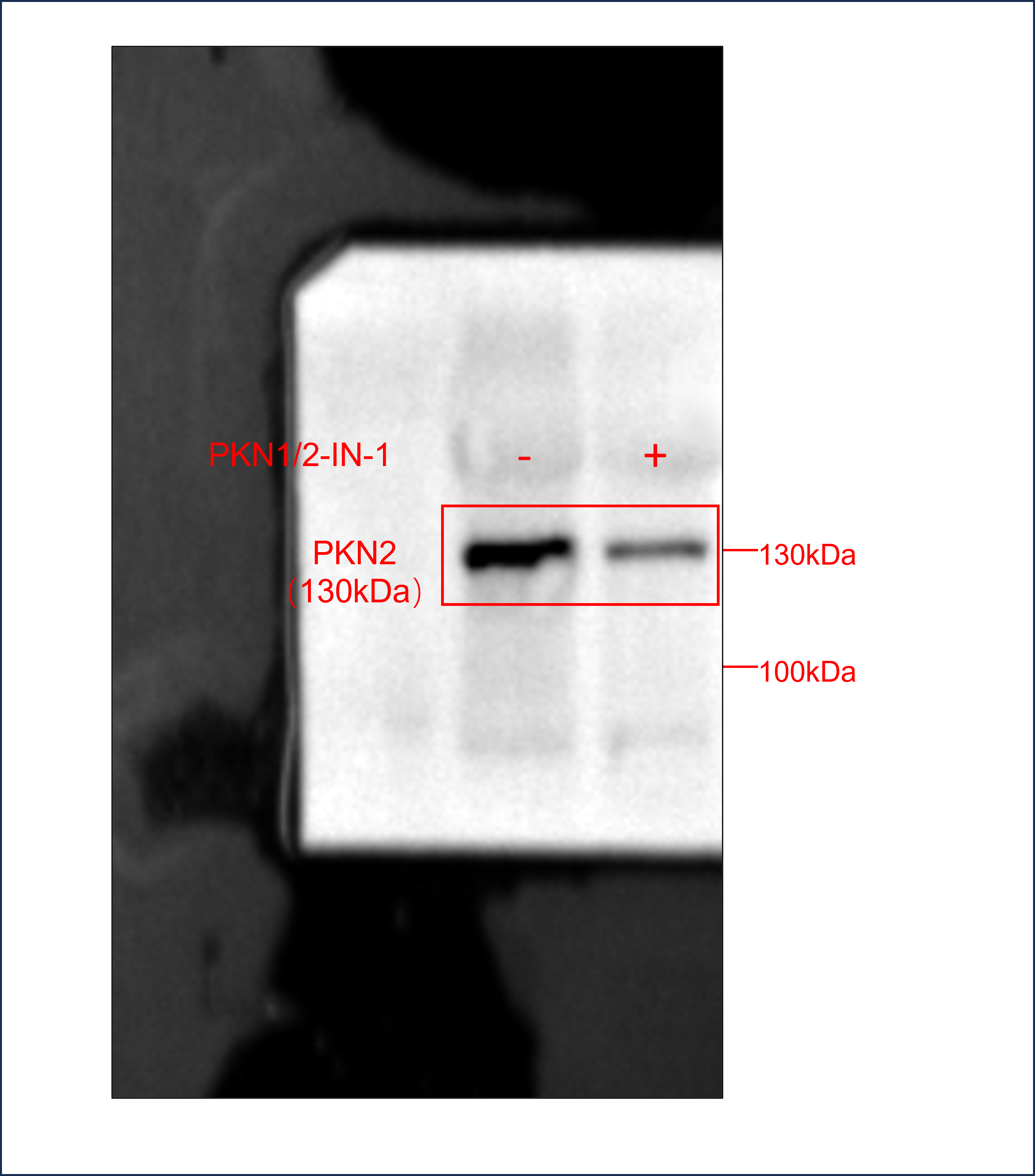

Supplement: Supplementary file 2 — Supporting File 2: advs73796‐sup‐0002‐Supplementary Figures_Raw_Data_Figures.zip. [file ADVS-13-e21337-s001.zip › Figure S7A_Raw_Data_Figures/PKN2(1).tif]

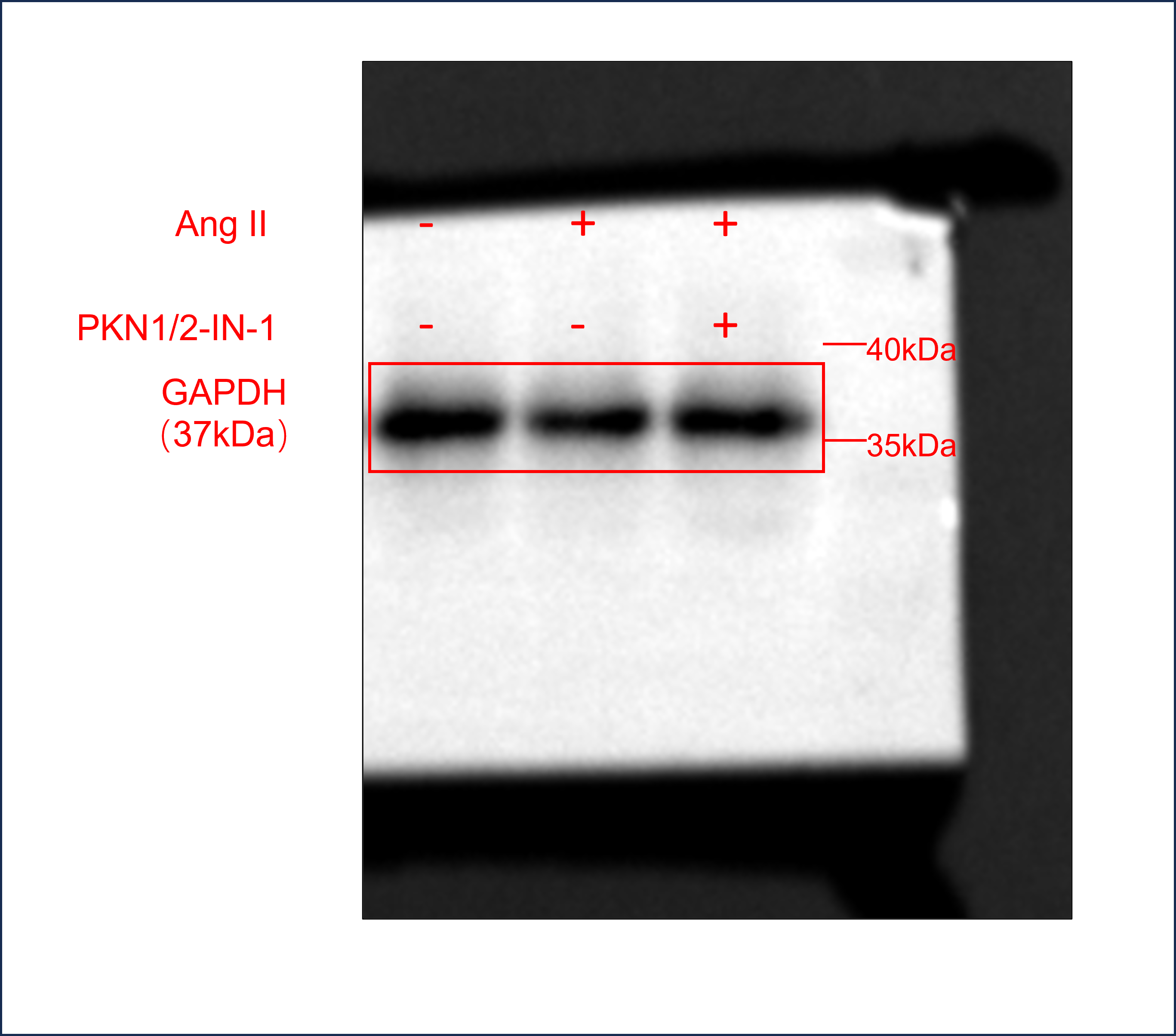

Supplement: Supplementary file 2 — Supporting File 2: advs73796‐sup‐0002‐Supplementary Figures_Raw_Data_Figures.zip. [file ADVS-13-e21337-s001.zip › Figure S7C_Raw_Data_Figures/GAPDH(3).tif]

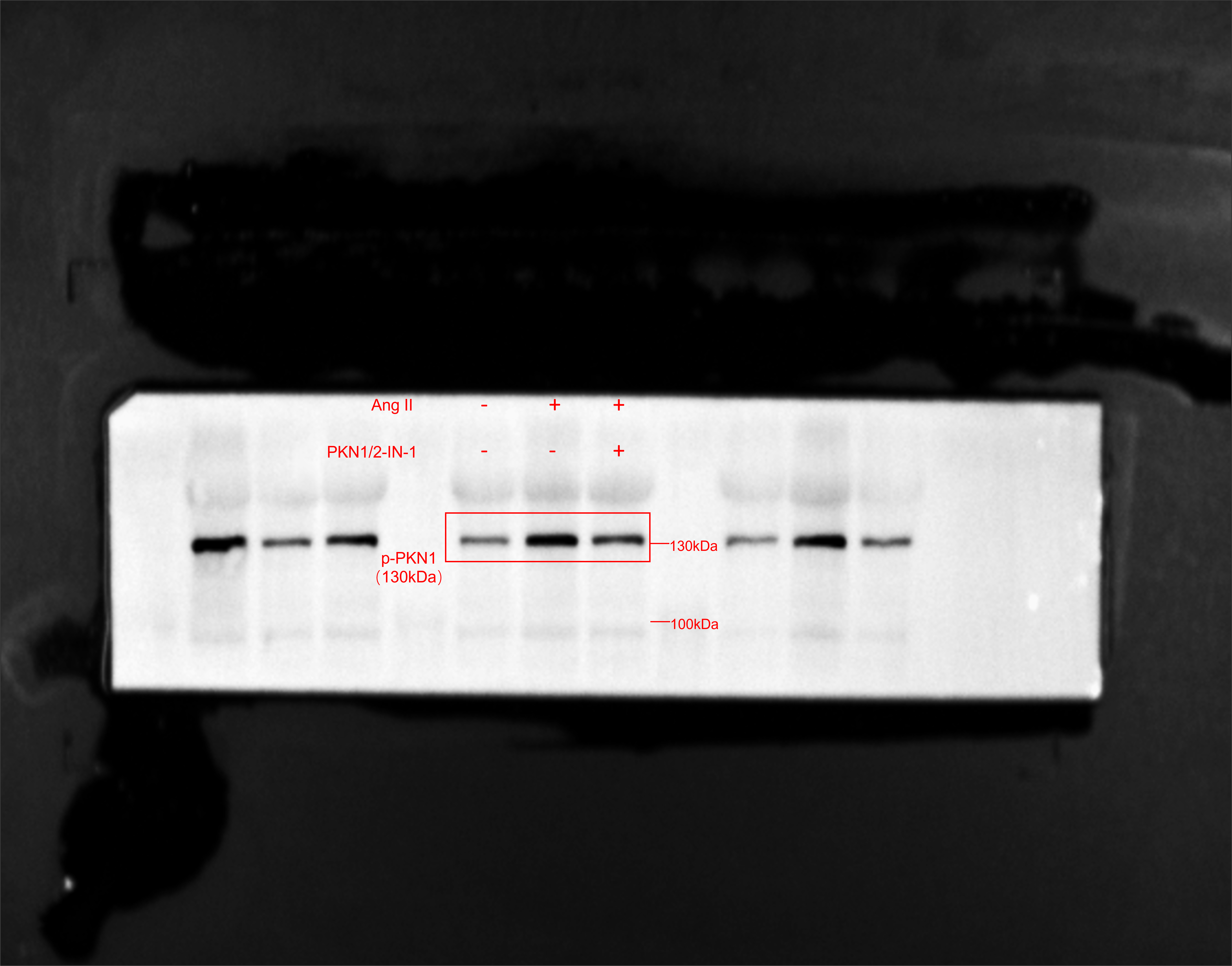

Supplement: Supplementary file 2 — Supporting File 2: advs73796‐sup‐0002‐Supplementary Figures_Raw_Data_Figures.zip. [file ADVS-13-e21337-s001.zip › Figure S7C_Raw_Data_Figures/p-PKN1.tif]

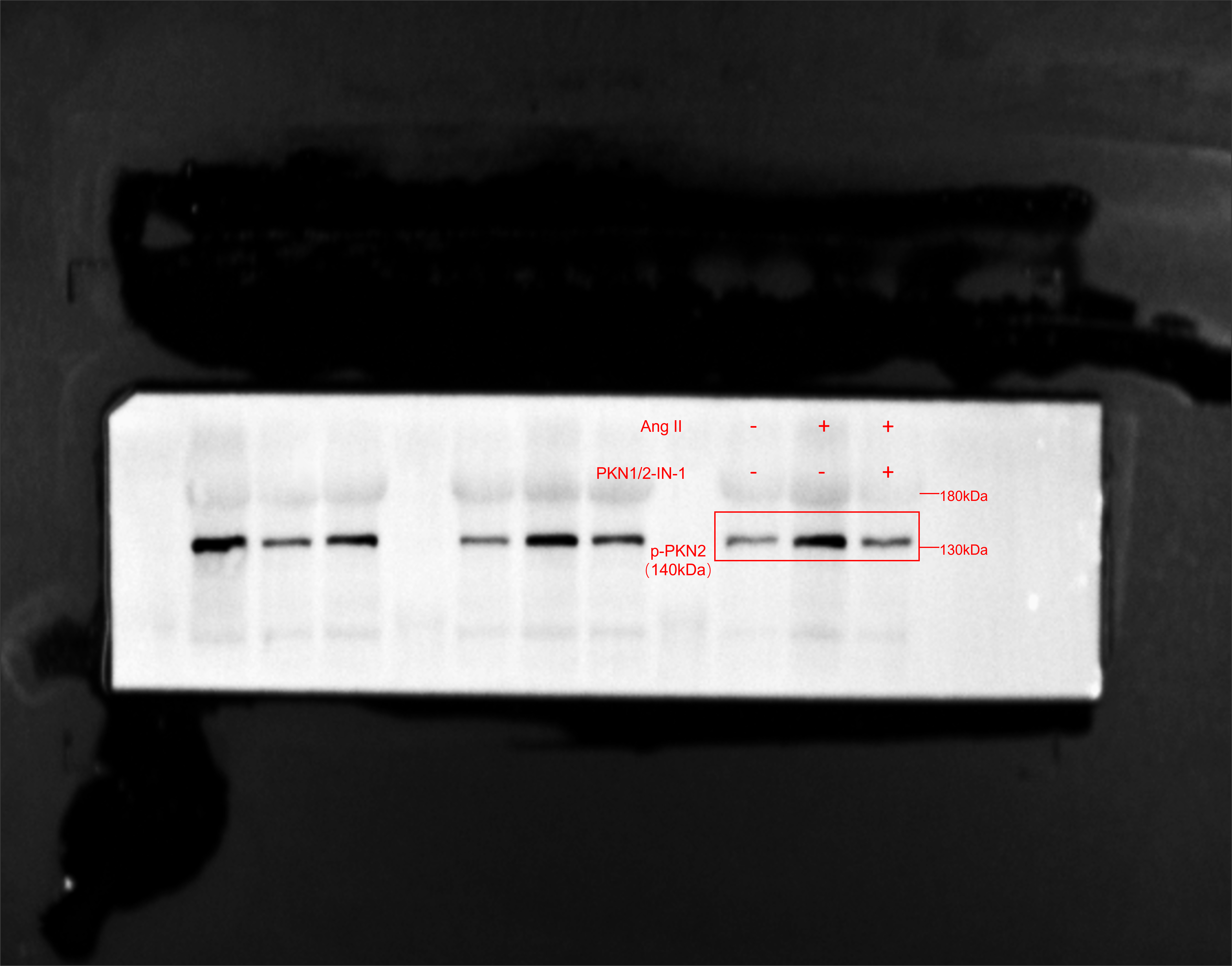

Supplement: Supplementary file 2 — Supporting File 2: advs73796‐sup‐0002‐Supplementary Figures_Raw_Data_Figures.zip. [file ADVS-13-e21337-s001.zip › Figure S7C_Raw_Data_Figures/p-PKN2.tif]

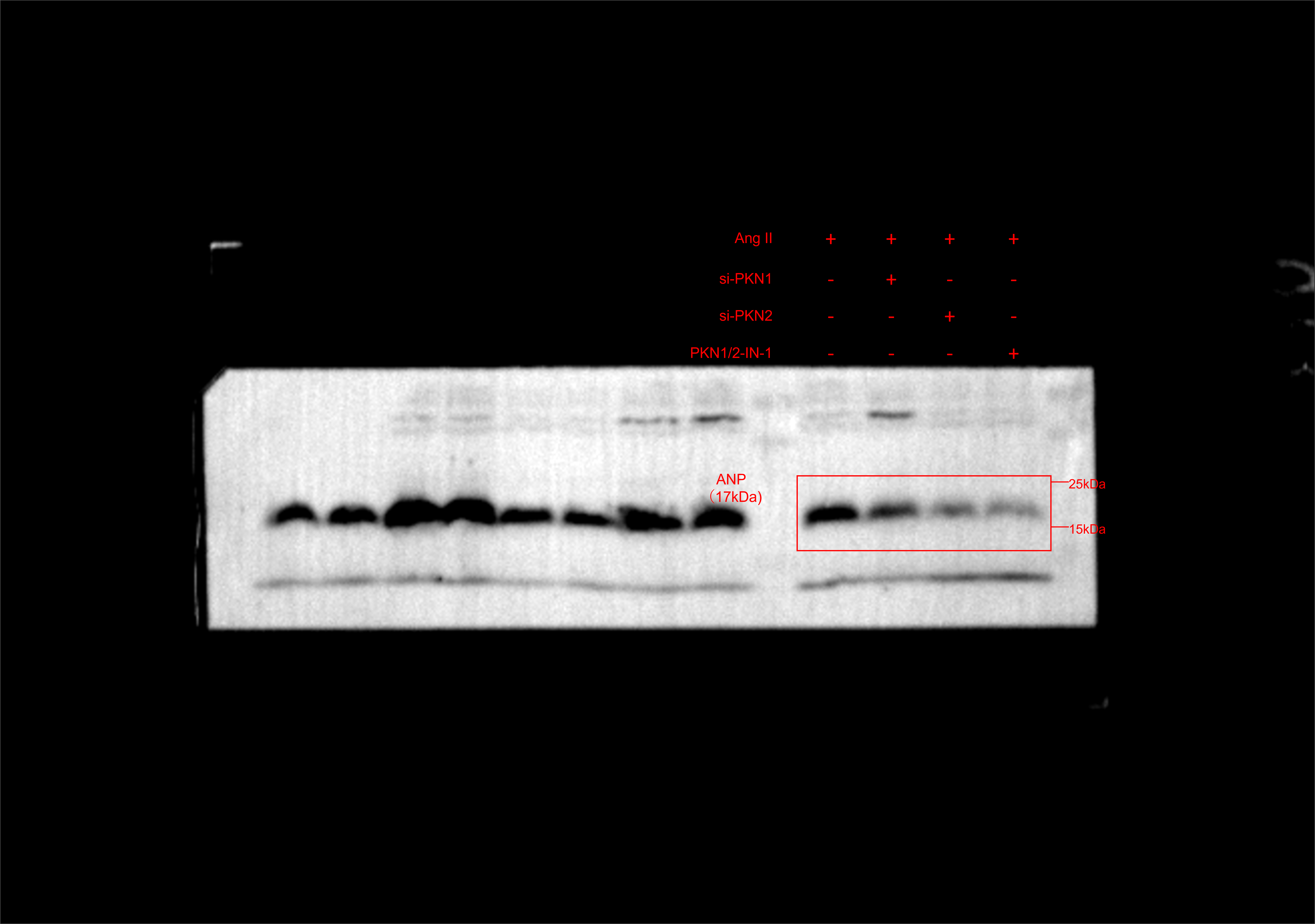

Supplement: Supplementary file 2 — Supporting File 2: advs73796‐sup‐0002‐Supplementary Figures_Raw_Data_Figures.zip. [file ADVS-13-e21337-s001.zip › Figure S7E_Raw_Data_Figures/ANP.tif]

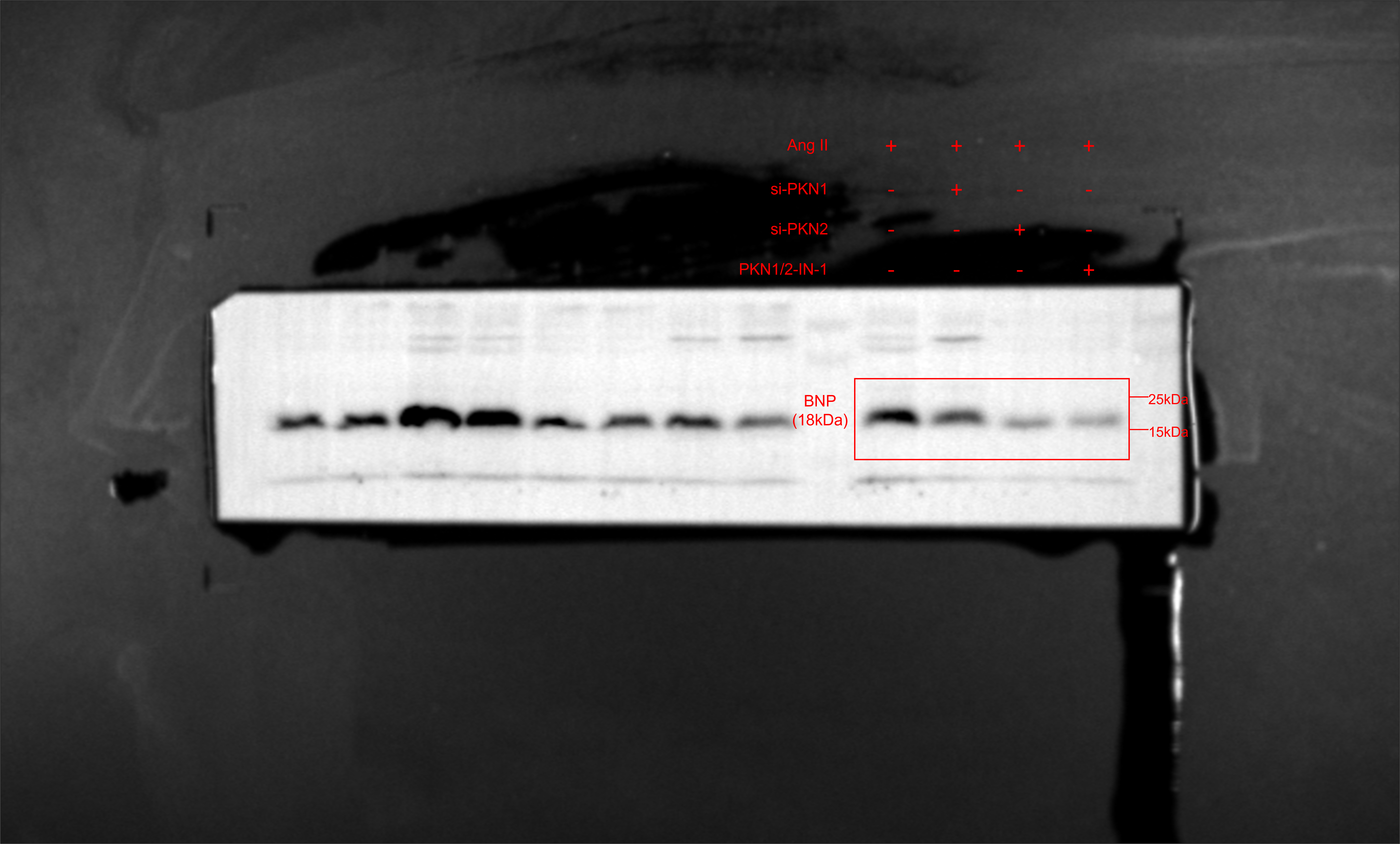

Supplement: Supplementary file 2 — Supporting File 2: advs73796‐sup‐0002‐Supplementary Figures_Raw_Data_Figures.zip. [file ADVS-13-e21337-s001.zip › Figure S7E_Raw_Data_Figures/BNP.tif]

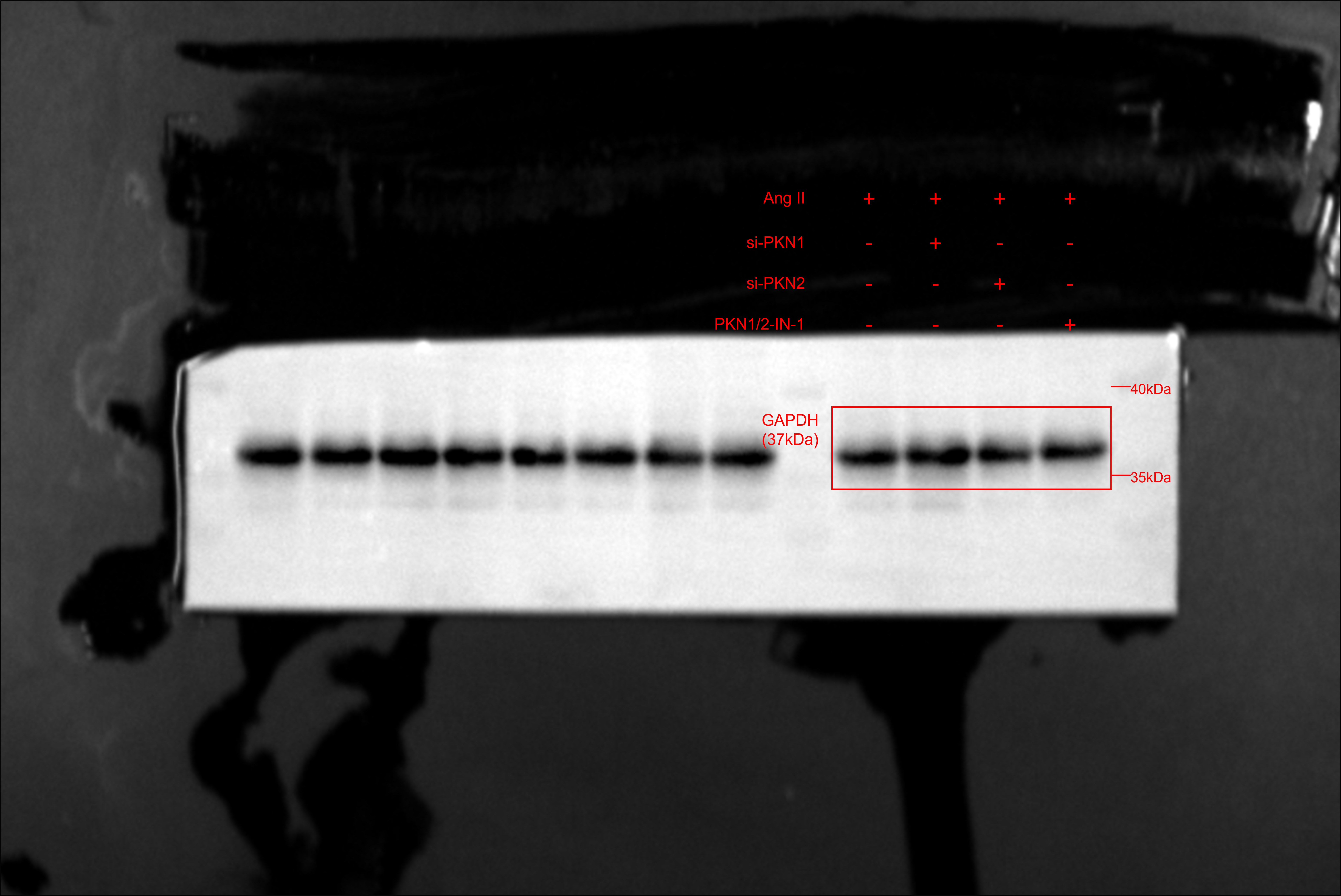

Supplement: Supplementary file 2 — Supporting File 2: advs73796‐sup‐0002‐Supplementary Figures_Raw_Data_Figures.zip. [file ADVS-13-e21337-s001.zip › Figure S7E_Raw_Data_Figures/GAPDH.tif]

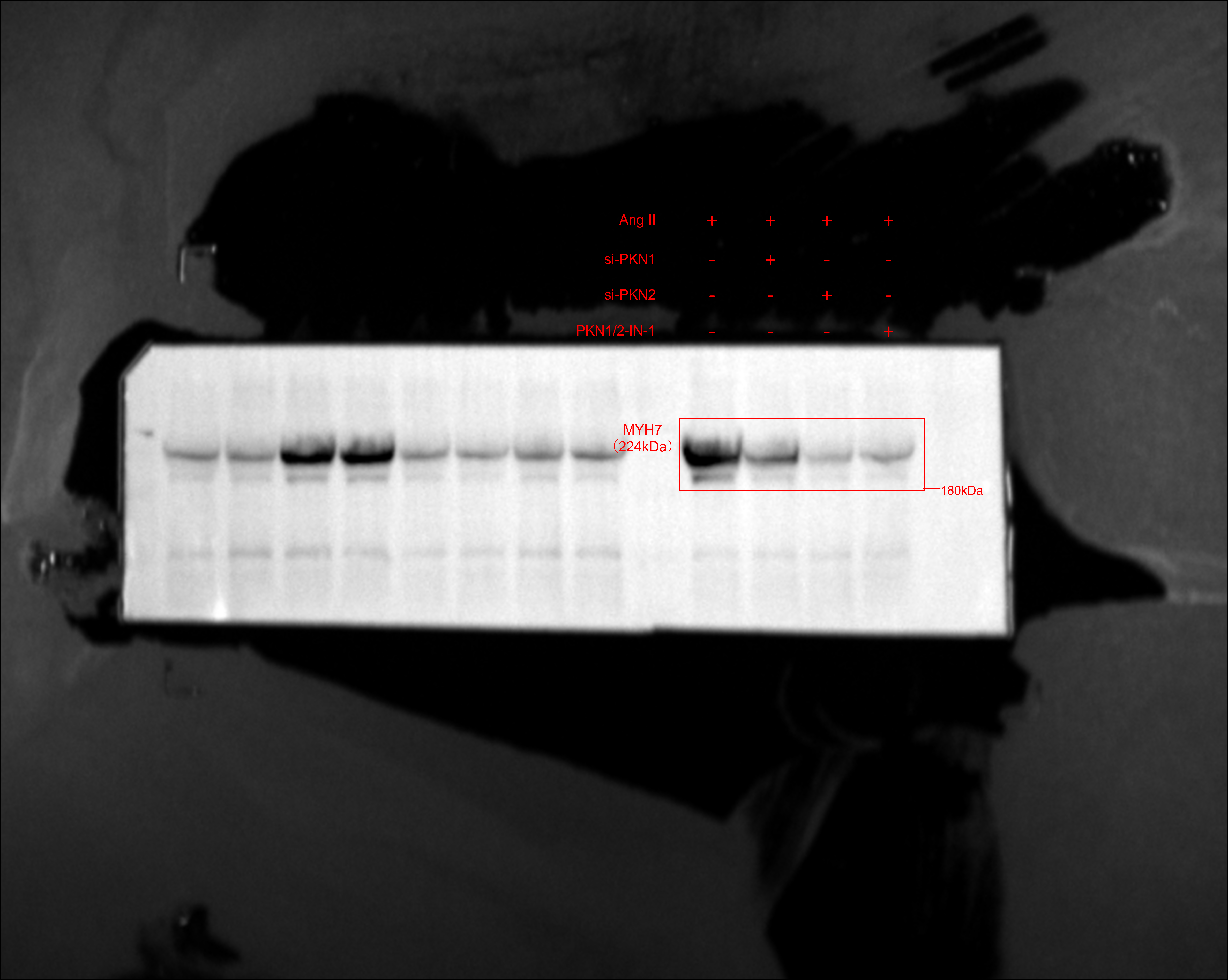

Supplement: Supplementary file 2 — Supporting File 2: advs73796‐sup‐0002‐Supplementary Figures_Raw_Data_Figures.zip. [file ADVS-13-e21337-s001.zip › Figure S7E_Raw_Data_Figures/MYH7.tif]

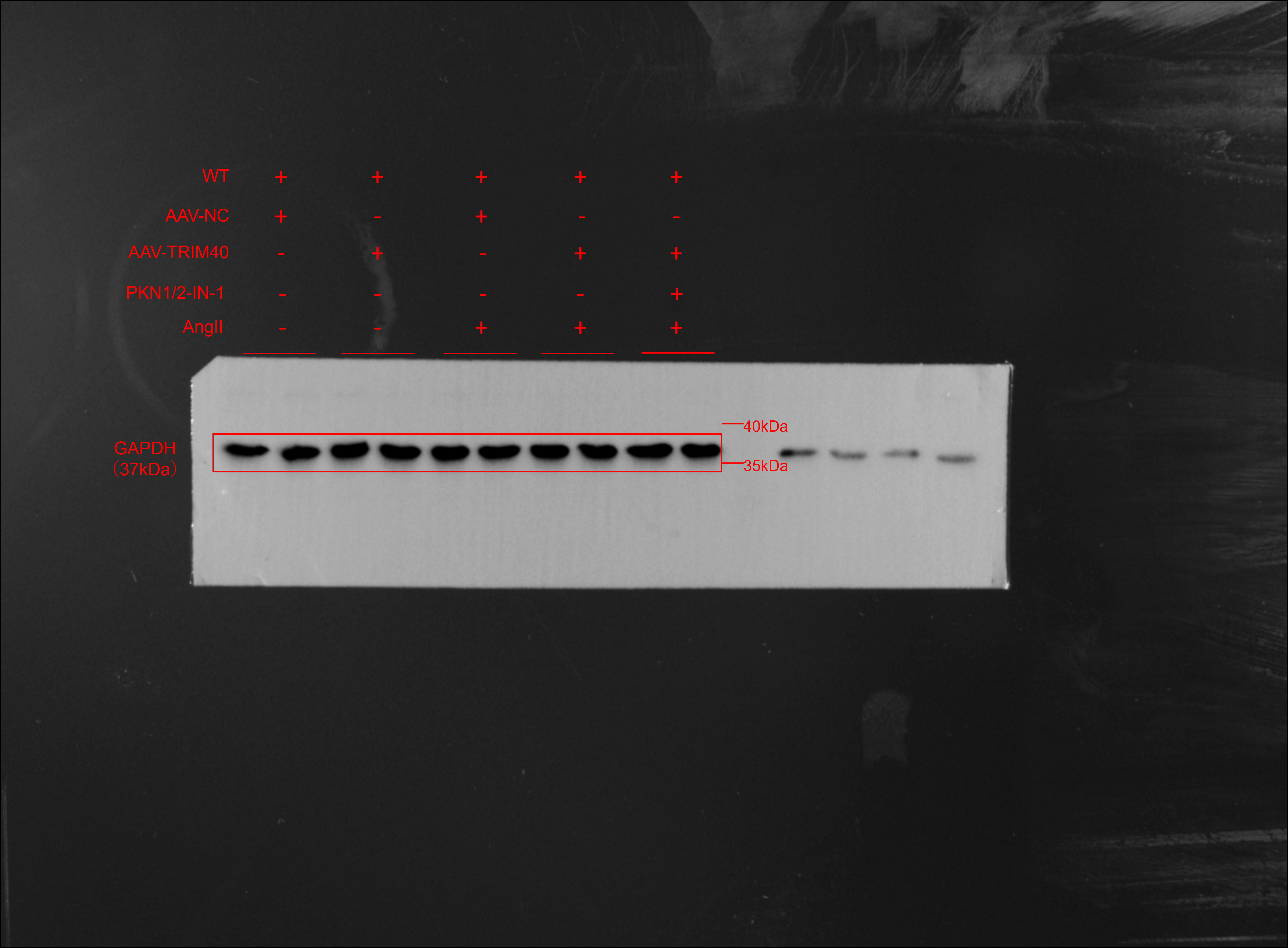

Supplement: Supplementary file 2 — Supporting File 2: advs73796‐sup‐0002‐Supplementary Figures_Raw_Data_Figures.zip. [file ADVS-13-e21337-s001.zip › Figure S8B_Raw_Data_Figures/GAPDH.tif]

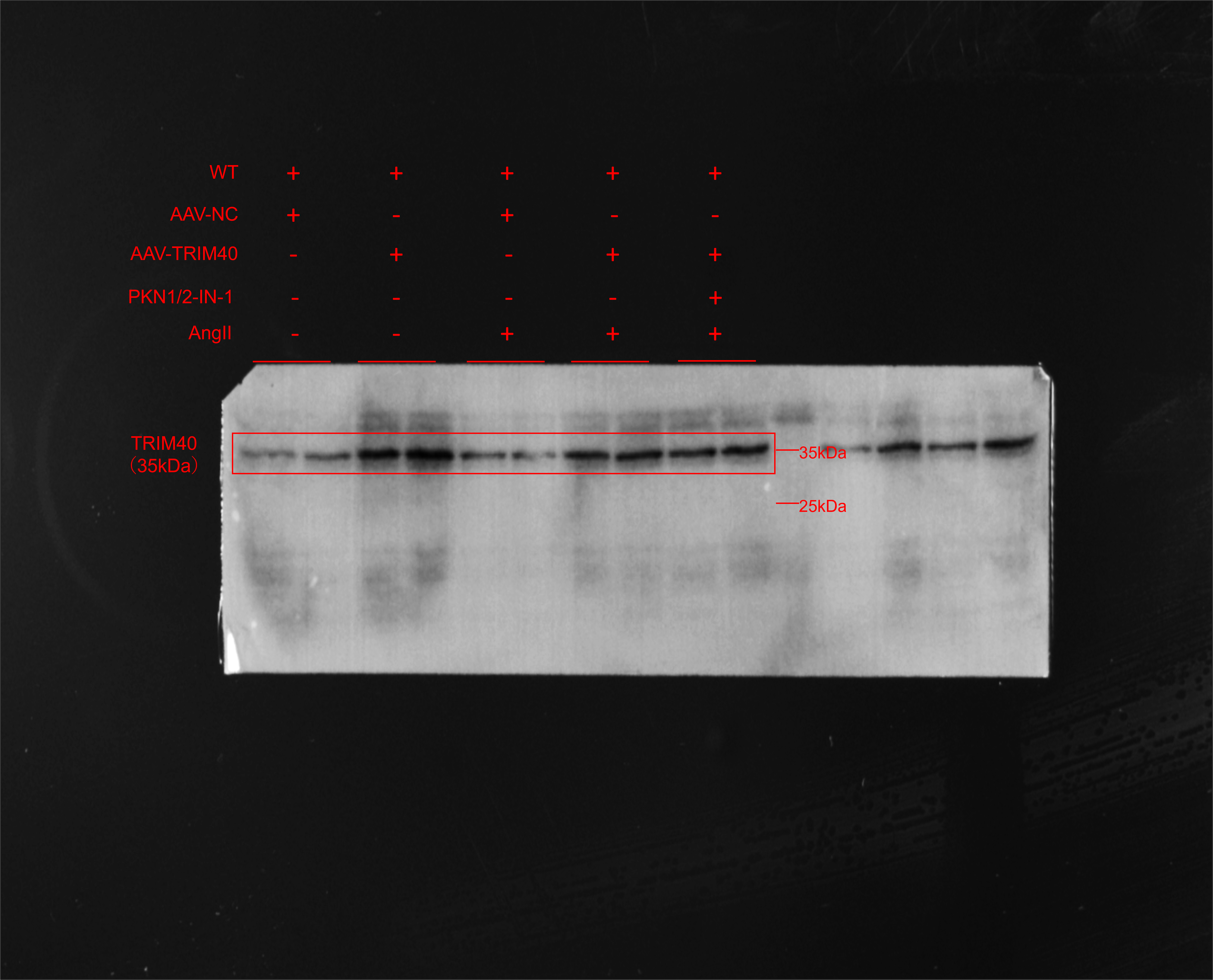

Supplement: Supplementary file 2 — Supporting File 2: advs73796‐sup‐0002‐Supplementary Figures_Raw_Data_Figures.zip. [file ADVS-13-e21337-s001.zip › Figure S8B_Raw_Data_Figures/TRIM40.tif]

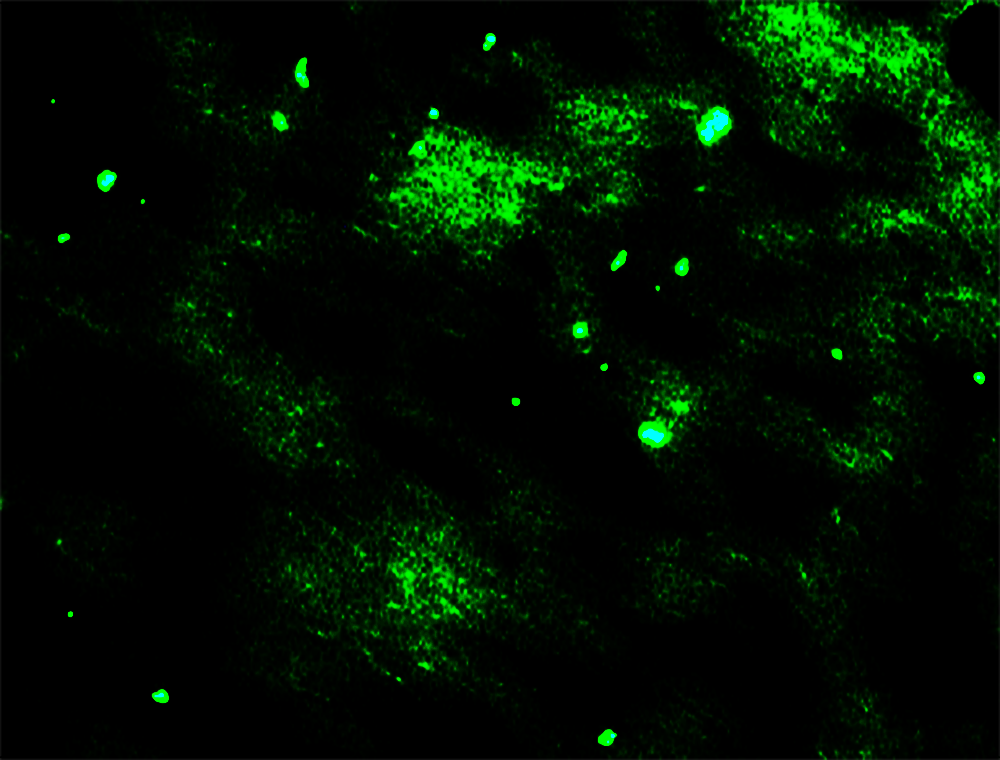

Supplement: Supplementary file 4 — Supporting File 4: advs73796‐sup‐0004‐Data.zip. [file ADVS-13-e21337-s003.zip › advs73796-sup-0004-Data/IF_Raw_Data_Figures/Figure 1D_RawData_Figures/Ang II-Actinin-40X.tif]

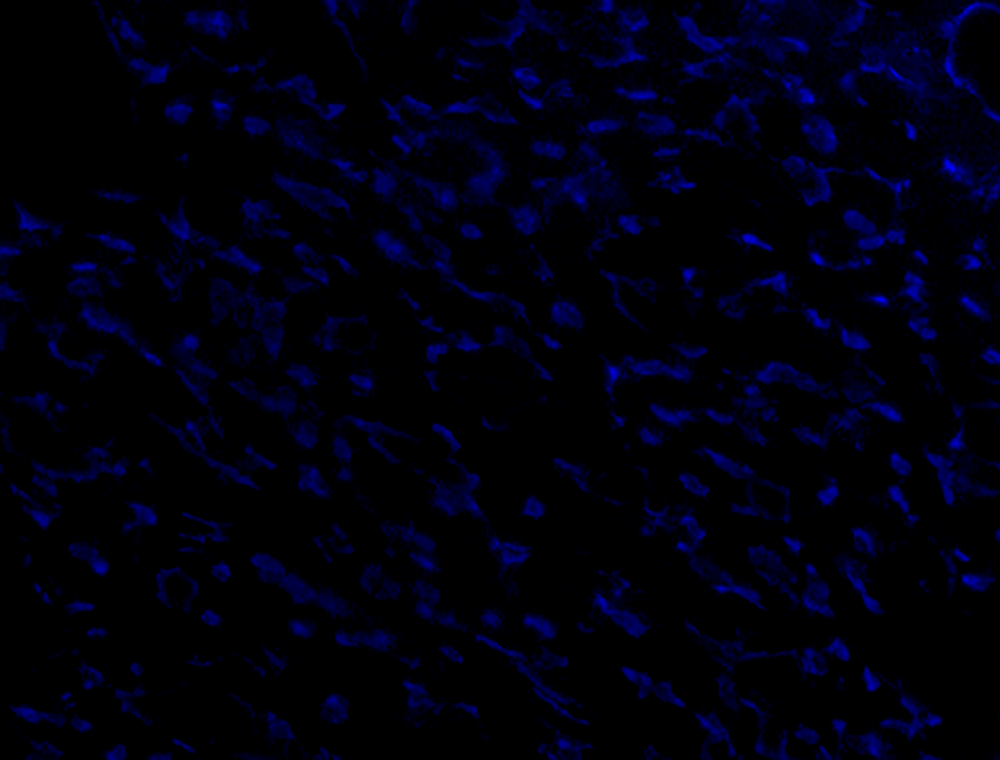

Supplement: Supplementary file 4 — Supporting File 4: advs73796‐sup‐0004‐Data.zip. [file ADVS-13-e21337-s003.zip › advs73796-sup-0004-Data/IF_Raw_Data_Figures/Figure 1D_RawData_Figures/Ang II-DAPI (Actinin)-40X.tif]

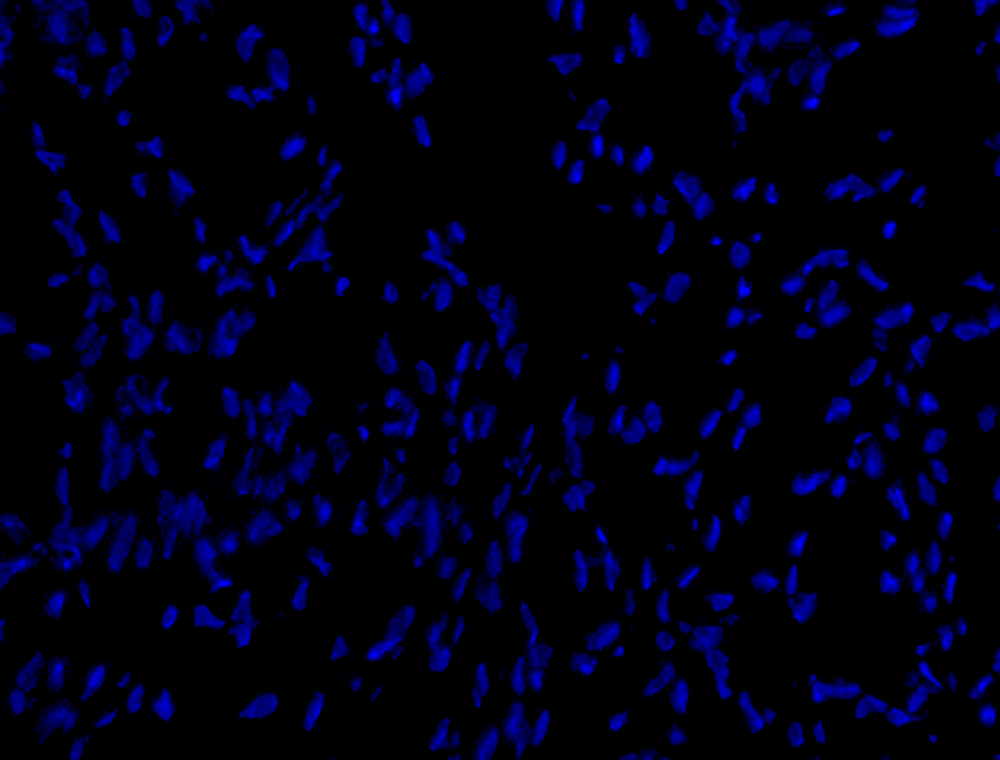

Supplement: Supplementary file 4 — Supporting File 4: advs73796‐sup‐0004‐Data.zip. [file ADVS-13-e21337-s003.zip › advs73796-sup-0004-Data/IF_Raw_Data_Figures/Figure 1D_RawData_Figures/Ang II-DAPI (Vimentin)-40X.tif]

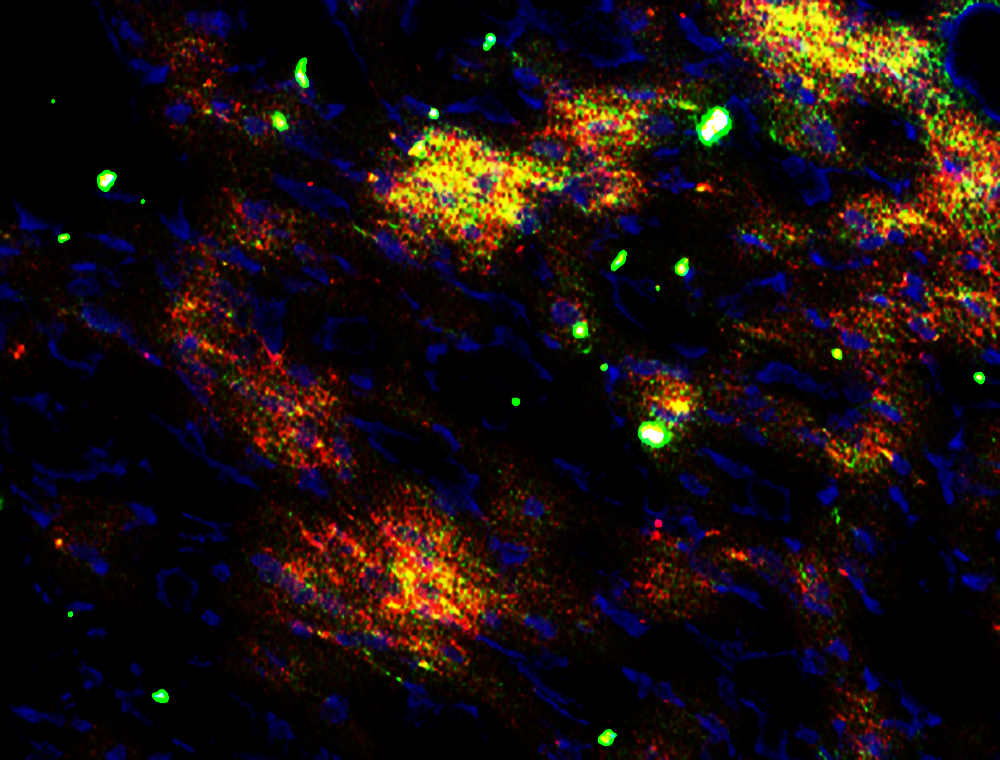

Supplement: Supplementary file 4 — Supporting File 4: advs73796‐sup‐0004‐Data.zip. [file ADVS-13-e21337-s003.zip › advs73796-sup-0004-Data/IF_Raw_Data_Figures/Figure 1D_RawData_Figures/Ang II-Merge (Actinin)-40X.tif]

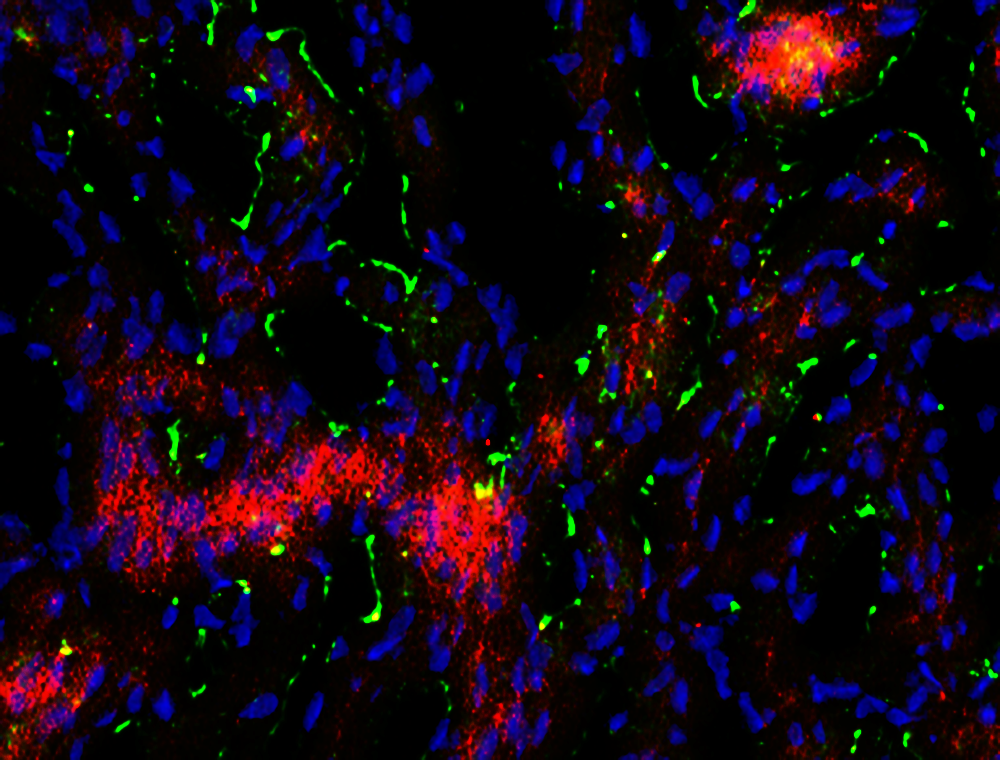

Supplement: Supplementary file 4 — Supporting File 4: advs73796‐sup‐0004‐Data.zip. [file ADVS-13-e21337-s003.zip › advs73796-sup-0004-Data/IF_Raw_Data_Figures/Figure 1D_RawData_Figures/Ang II-Merge (Vimentin)-40X.tif]

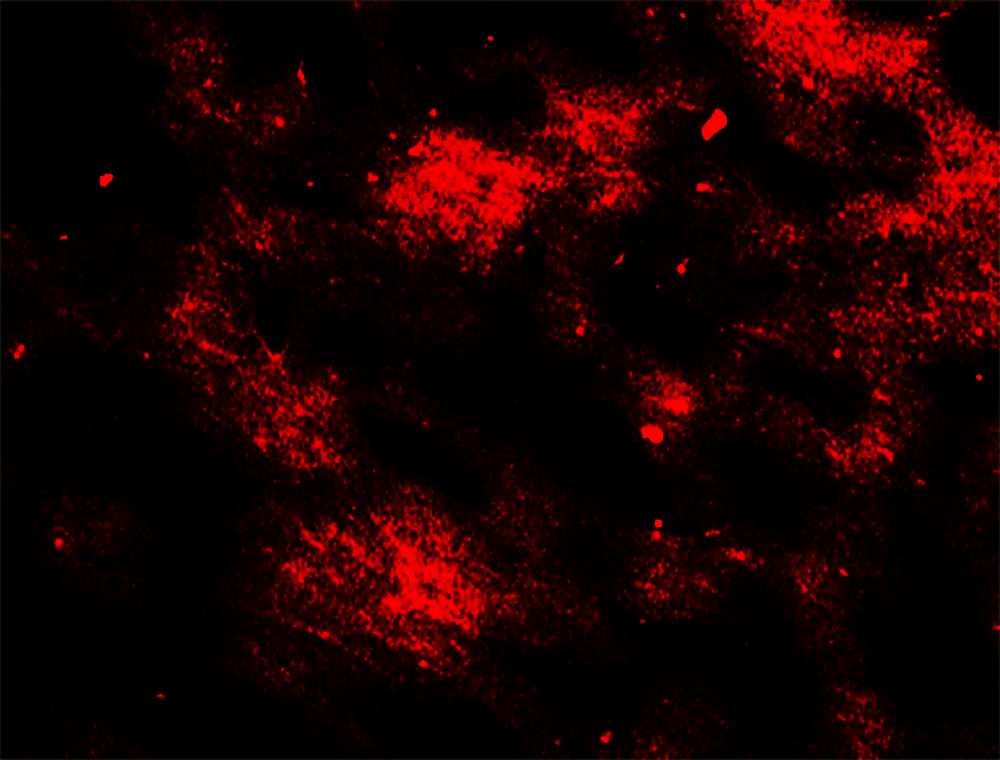

Supplement: Supplementary file 4 — Supporting File 4: advs73796‐sup‐0004‐Data.zip. [file ADVS-13-e21337-s003.zip › advs73796-sup-0004-Data/IF_Raw_Data_Figures/Figure 1D_RawData_Figures/Ang II-TRIM40 (Actinin)-40X.tif]

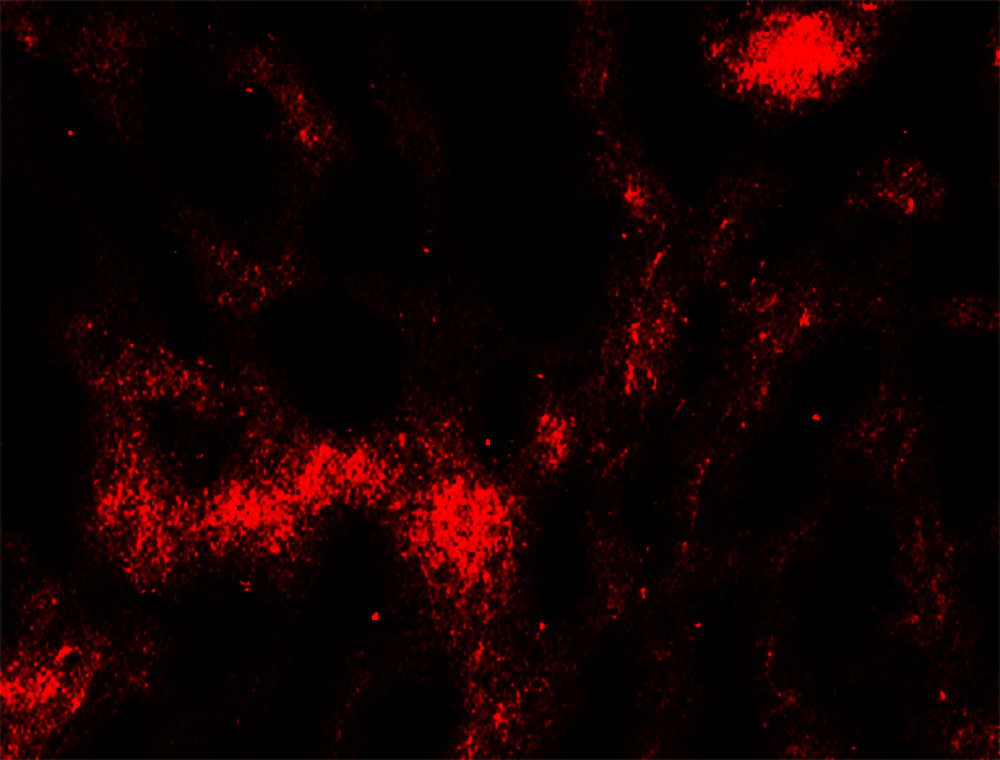

Supplement: Supplementary file 4 — Supporting File 4: advs73796‐sup‐0004‐Data.zip. [file ADVS-13-e21337-s003.zip › advs73796-sup-0004-Data/IF_Raw_Data_Figures/Figure 1D_RawData_Figures/Ang II-TRIM40 (Vimentin)-40X.tif]

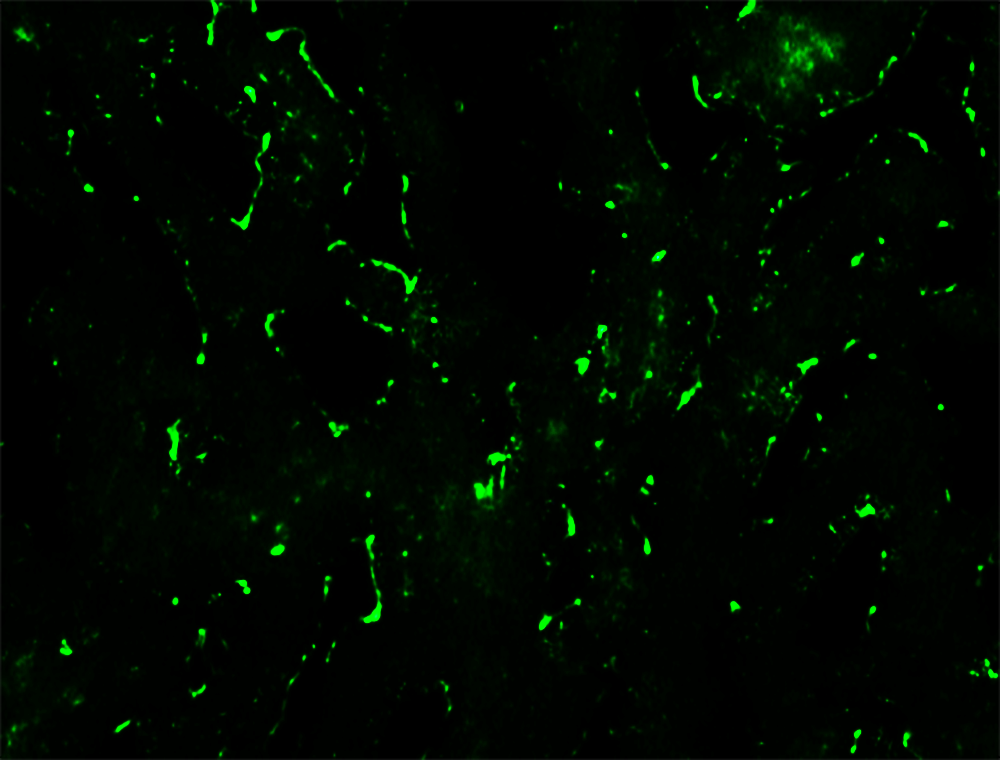

Supplement: Supplementary file 4 — Supporting File 4: advs73796‐sup‐0004‐Data.zip. [file ADVS-13-e21337-s003.zip › advs73796-sup-0004-Data/IF_Raw_Data_Figures/Figure 1D_RawData_Figures/Ang II-Vimentin-40X.tif]

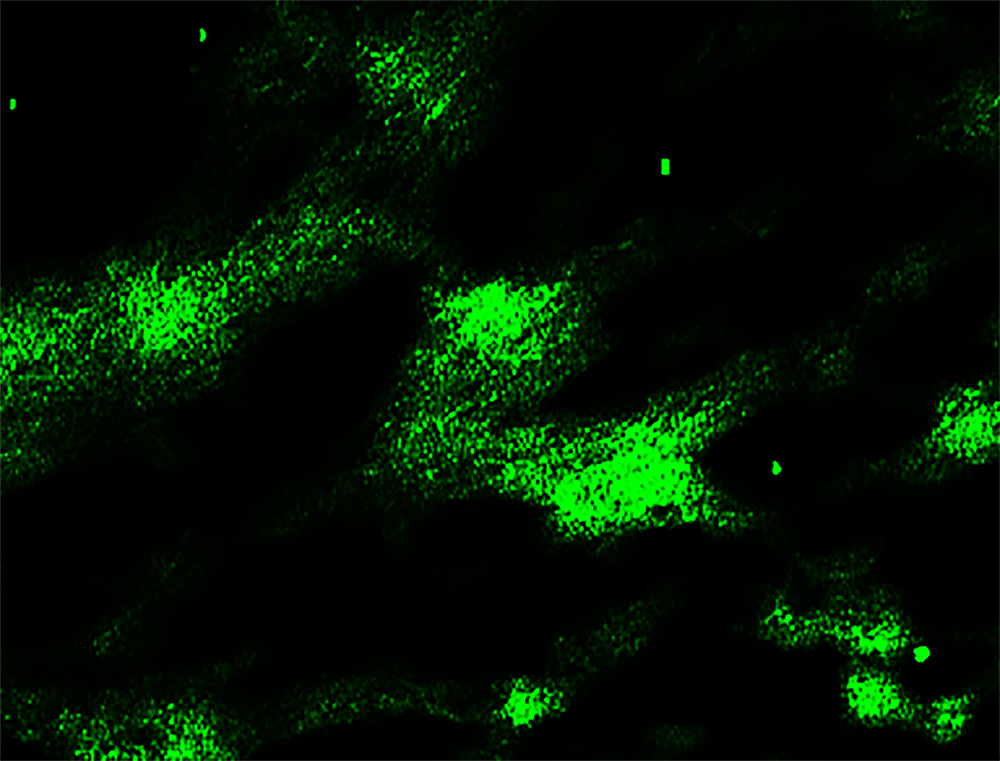

Supplement: Supplementary file 4 — Supporting File 4: advs73796‐sup‐0004‐Data.zip. [file ADVS-13-e21337-s003.zip › advs73796-sup-0004-Data/IF_Raw_Data_Figures/Figure 1D_RawData_Figures/Ctrl-Actinin-40X.tif]

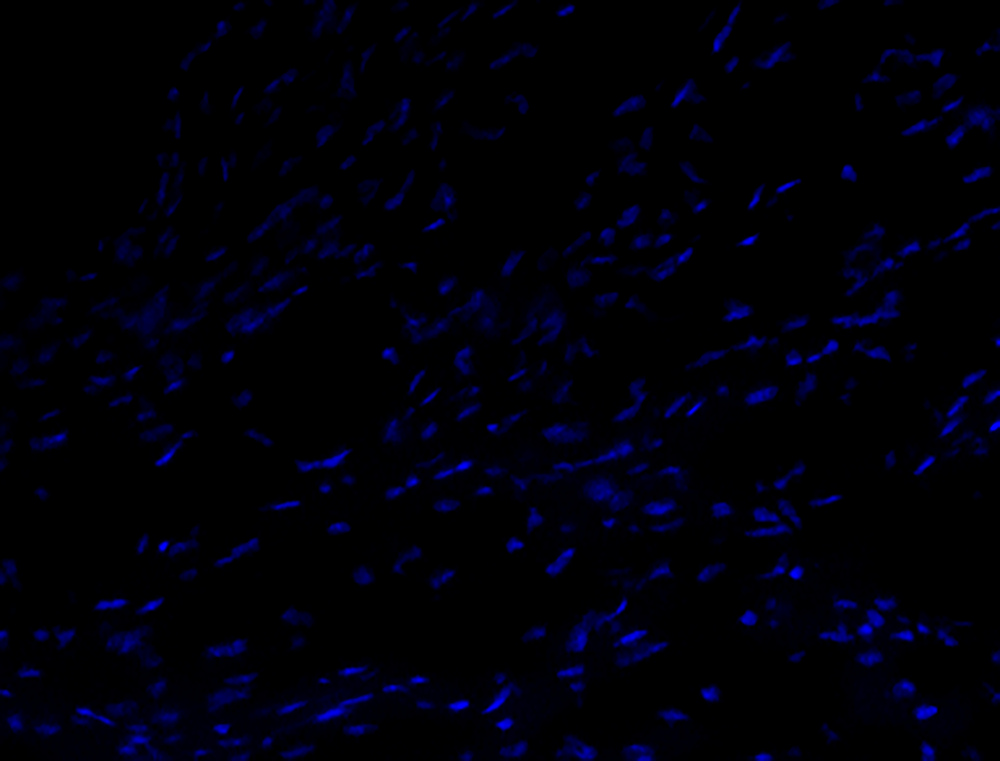

Supplement: Supplementary file 4 — Supporting File 4: advs73796‐sup‐0004‐Data.zip. [file ADVS-13-e21337-s003.zip › advs73796-sup-0004-Data/IF_Raw_Data_Figures/Figure 1D_RawData_Figures/Ctrl-DAPI (Actinin)-40X.tif]

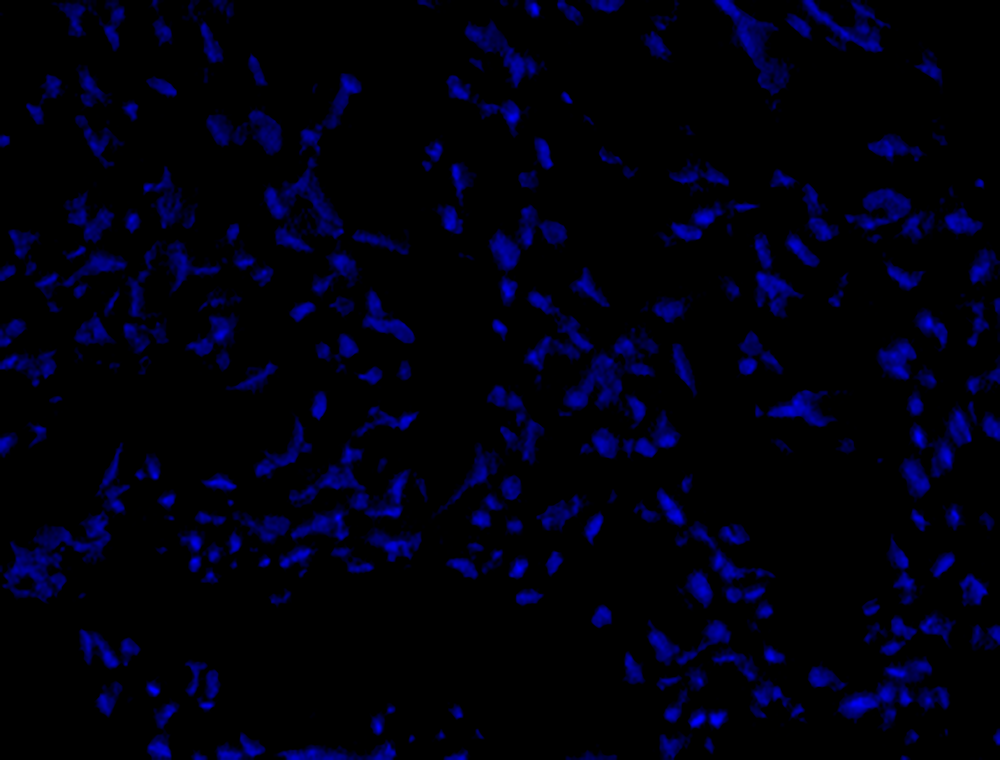

Supplement: Supplementary file 4 — Supporting File 4: advs73796‐sup‐0004‐Data.zip. [file ADVS-13-e21337-s003.zip › advs73796-sup-0004-Data/IF_Raw_Data_Figures/Figure 1D_RawData_Figures/Ctrl-DAPI (Vimentin)-40X.tif]

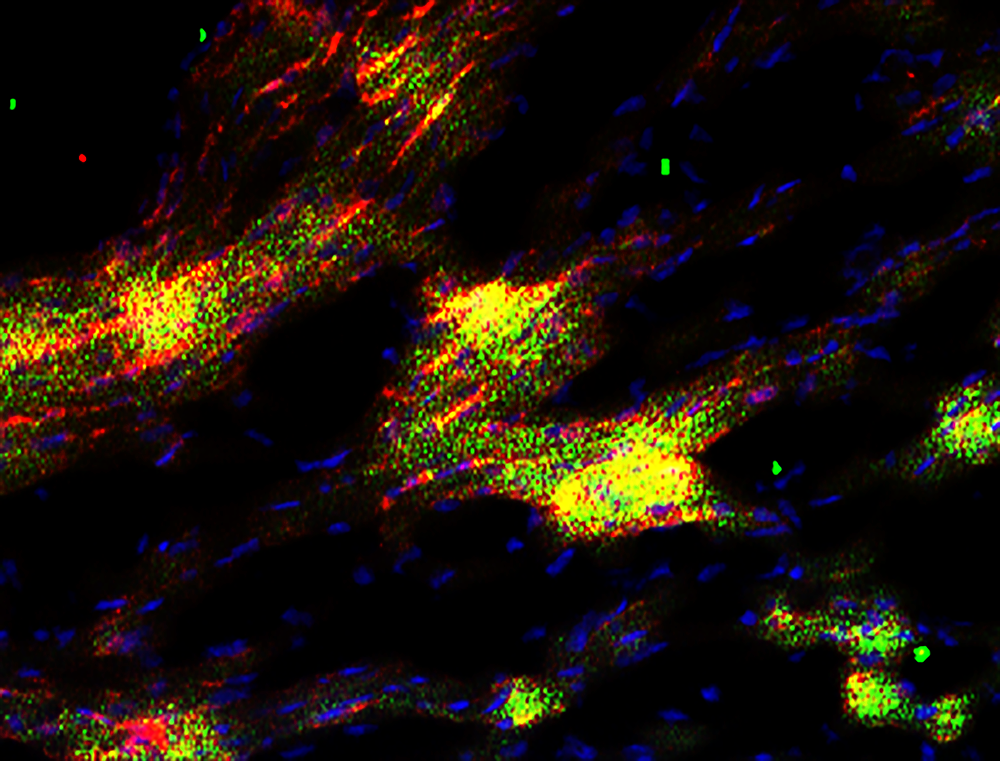

Supplement: Supplementary file 4 — Supporting File 4: advs73796‐sup‐0004‐Data.zip. [file ADVS-13-e21337-s003.zip › advs73796-sup-0004-Data/IF_Raw_Data_Figures/Figure 1D_RawData_Figures/Ctrl-Merge (Actinin)-40X.tif]

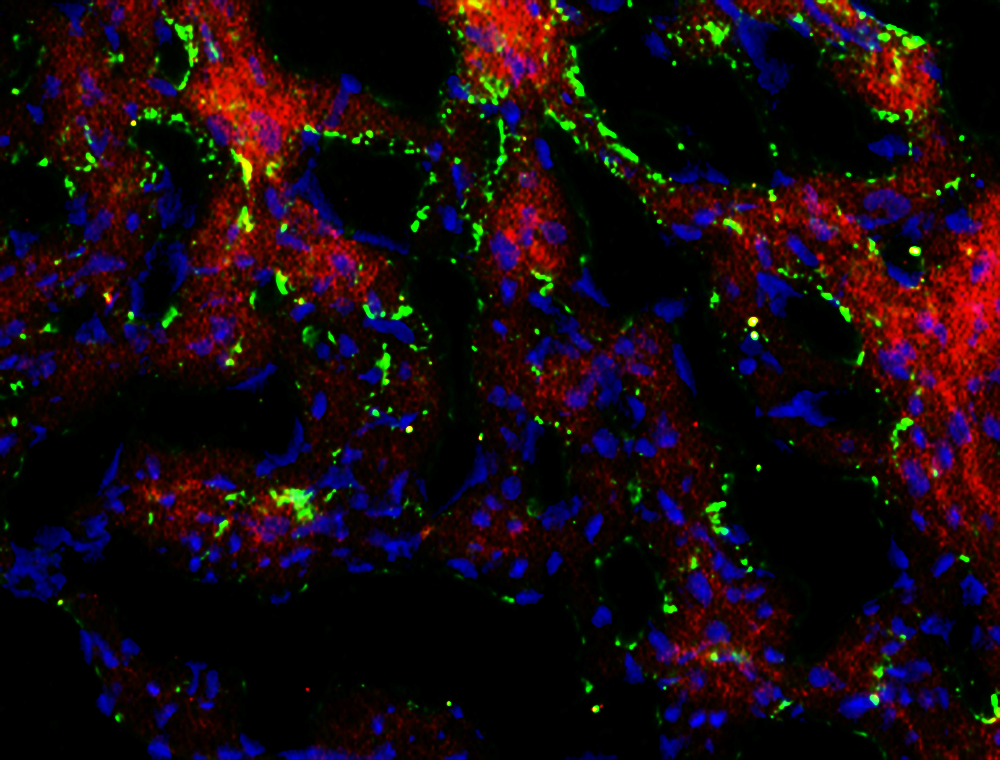

Supplement: Supplementary file 4 — Supporting File 4: advs73796‐sup‐0004‐Data.zip. [file ADVS-13-e21337-s003.zip › advs73796-sup-0004-Data/IF_Raw_Data_Figures/Figure 1D_RawData_Figures/Ctrl-Merge (Vimentin)-40X.tif]

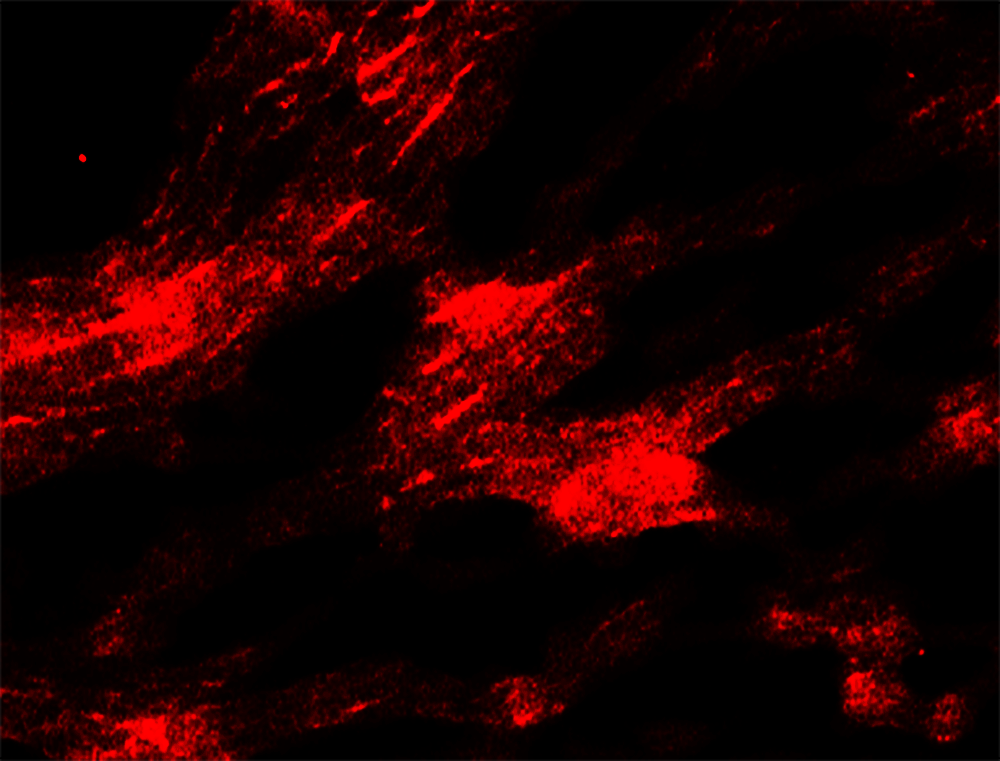

Supplement: Supplementary file 4 — Supporting File 4: advs73796‐sup‐0004‐Data.zip. [file ADVS-13-e21337-s003.zip › advs73796-sup-0004-Data/IF_Raw_Data_Figures/Figure 1D_RawData_Figures/Ctrl-TRIM40 (Actinin)-40X.tif]

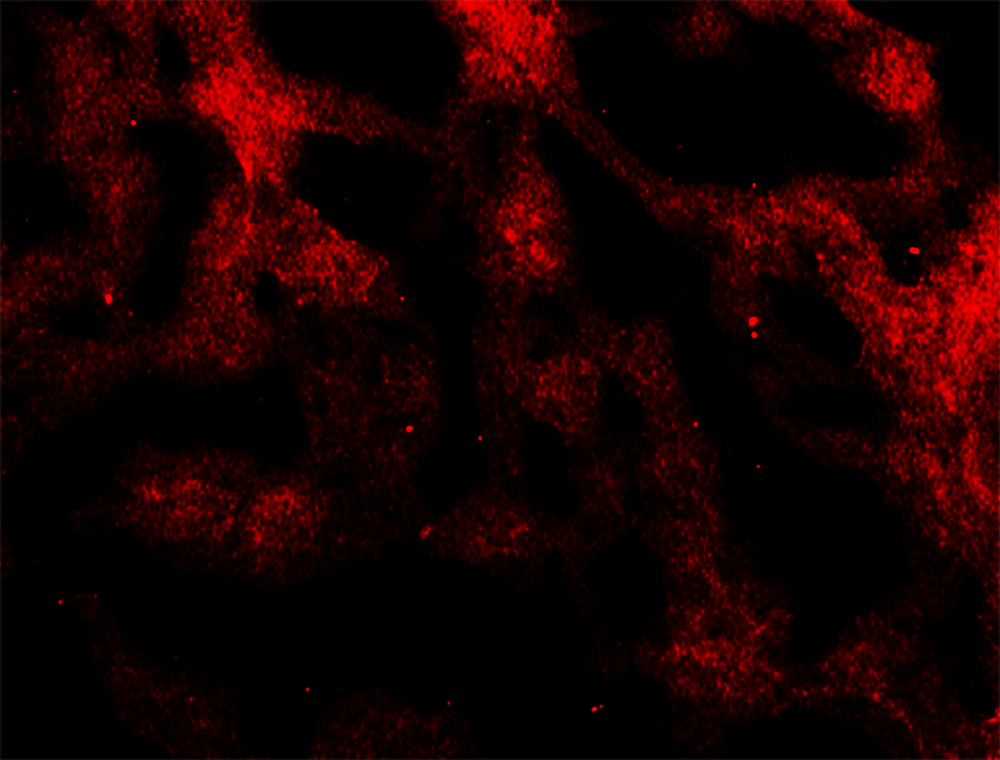

Supplement: Supplementary file 4 — Supporting File 4: advs73796‐sup‐0004‐Data.zip. [file ADVS-13-e21337-s003.zip › advs73796-sup-0004-Data/IF_Raw_Data_Figures/Figure 1D_RawData_Figures/Ctrl-TRIM40 (Vimentin)-40X.tif]

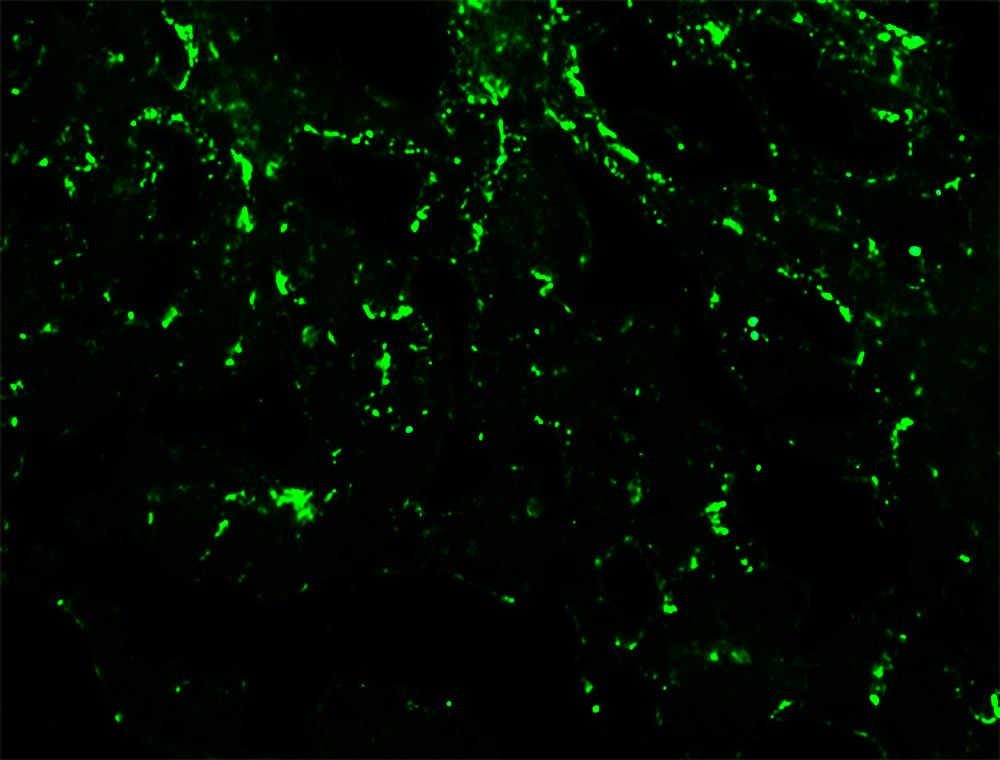

Supplement: Supplementary file 4 — Supporting File 4: advs73796‐sup‐0004‐Data.zip. [file ADVS-13-e21337-s003.zip › advs73796-sup-0004-Data/IF_Raw_Data_Figures/Figure 1D_RawData_Figures/Ctrl-Vimentin-40X.tif]

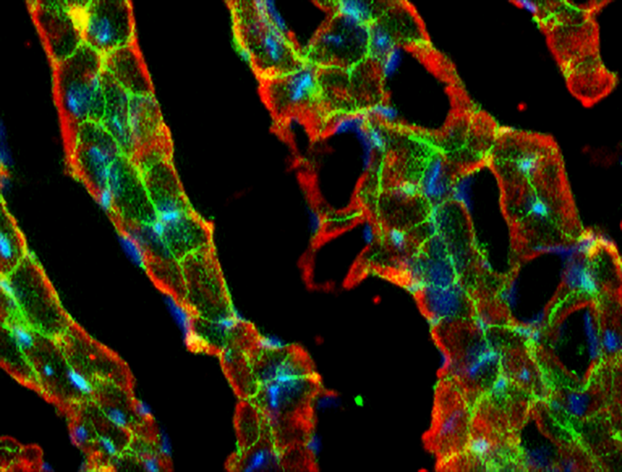

Supplement: Supplementary file 4 — Supporting File 4: advs73796‐sup‐0004‐Data.zip. [file ADVS-13-e21337-s003.zip › advs73796-sup-0004-Data/IF_Raw_Data_Figures/Figure 2J_RawData_Figures/WGA&cTnT&DAPI-TRIM40 knockout-Ang II-40X.tif]

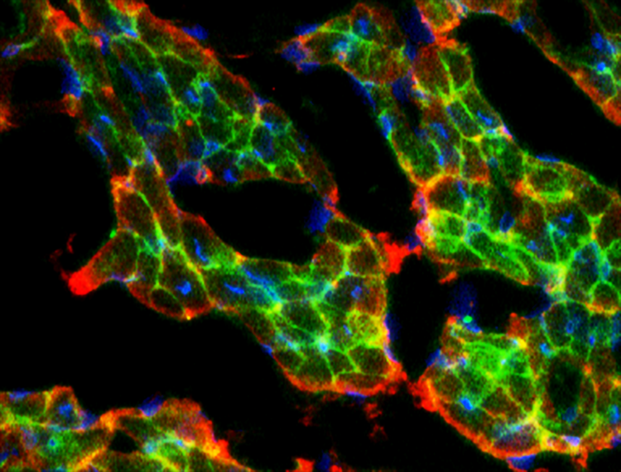

Supplement: Supplementary file 4 — Supporting File 4: advs73796‐sup‐0004‐Data.zip. [file ADVS-13-e21337-s003.zip › advs73796-sup-0004-Data/IF_Raw_Data_Figures/Figure 2J_RawData_Figures/WGA&cTnT&DAPI-TRIM40 knockout-Sham-40X.tif]

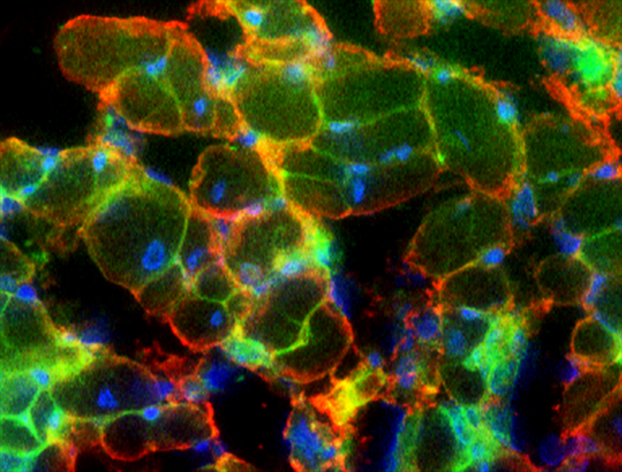

Supplement: Supplementary file 4 — Supporting File 4: advs73796‐sup‐0004‐Data.zip. [file ADVS-13-e21337-s003.zip › advs73796-sup-0004-Data/IF_Raw_Data_Figures/Figure 2J_RawData_Figures/WGA&cTnT&DAPI-WT-Ang II-40X.tif]

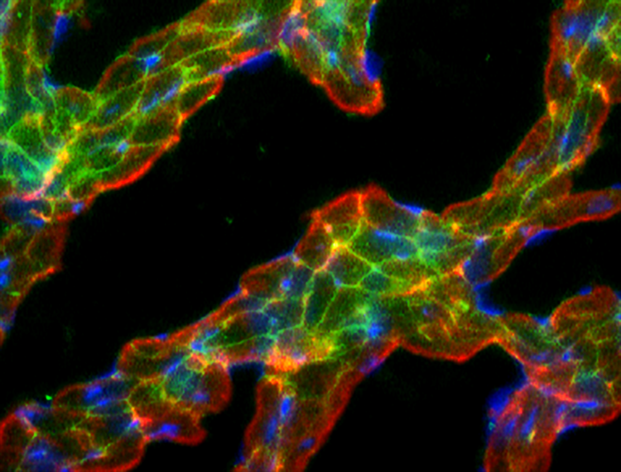

Supplement: Supplementary file 4 — Supporting File 4: advs73796‐sup‐0004‐Data.zip. [file ADVS-13-e21337-s003.zip › advs73796-sup-0004-Data/IF_Raw_Data_Figures/Figure 2J_RawData_Figures/WGA&cTnT&DAPI-WT-Sham-40X.tif]

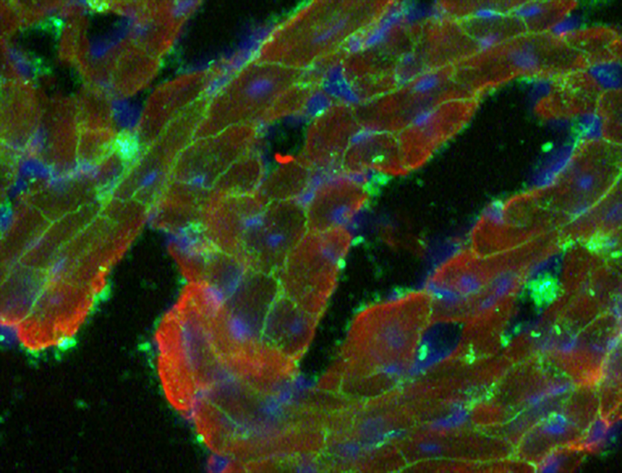

Supplement: Supplementary file 4 — Supporting File 4: advs73796‐sup‐0004‐Data.zip. [file ADVS-13-e21337-s003.zip › advs73796-sup-0004-Data/IF_Raw_Data_Figures/Figure 3H_RawData_Figures/WGA&cTnT&DAPI-TRIM40 knockout-Sham-40X.tif]

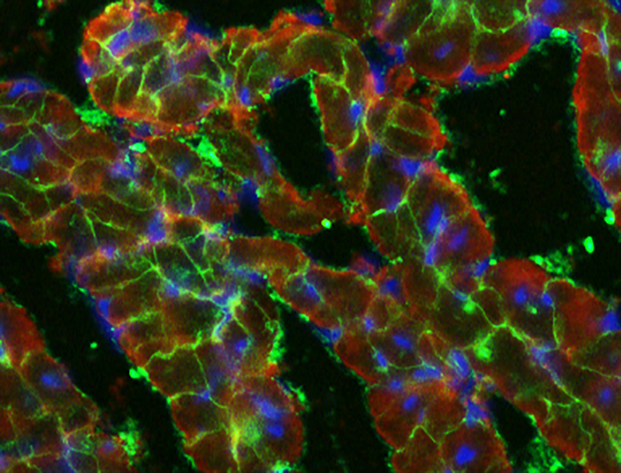

Supplement: Supplementary file 4 — Supporting File 4: advs73796‐sup‐0004‐Data.zip. [file ADVS-13-e21337-s003.zip › advs73796-sup-0004-Data/IF_Raw_Data_Figures/Figure 3H_RawData_Figures/WGA&cTnT&DAPI-TRIM40 knockout-TAC-40X.tif]

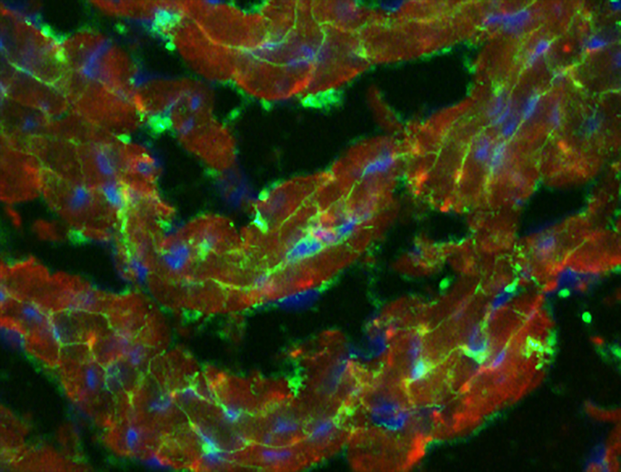

Supplement: Supplementary file 4 — Supporting File 4: advs73796‐sup‐0004‐Data.zip. [file ADVS-13-e21337-s003.zip › advs73796-sup-0004-Data/IF_Raw_Data_Figures/Figure 3H_RawData_Figures/WGA&cTnT&DAPI-WT-Sham-40X.tif]

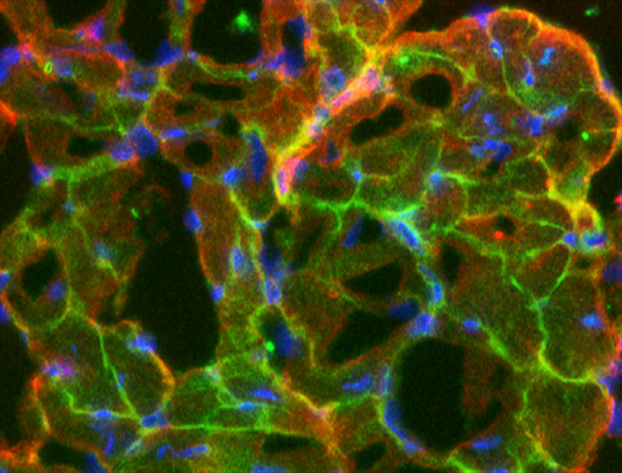

Supplement: Supplementary file 4 — Supporting File 4: advs73796‐sup‐0004‐Data.zip. [file ADVS-13-e21337-s003.zip › advs73796-sup-0004-Data/IF_Raw_Data_Figures/Figure 3H_RawData_Figures/WGA&cTnT&DAPI-WT-TAC.tif]

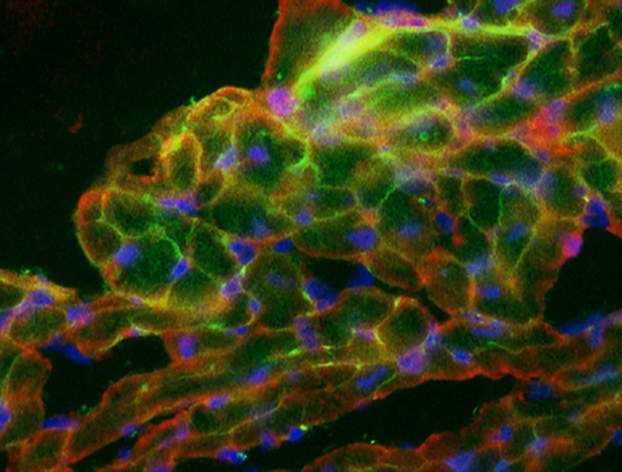

Supplement: Supplementary file 4 — Supporting File 4: advs73796‐sup‐0004‐Data.zip. [file ADVS-13-e21337-s003.zip › advs73796-sup-0004-Data/IF_Raw_Data_Figures/Figure 4K_RawData_Figures/WGA&cTnT&DAPI-AAV9-cTnT-NC-Ang II-40X.tif]

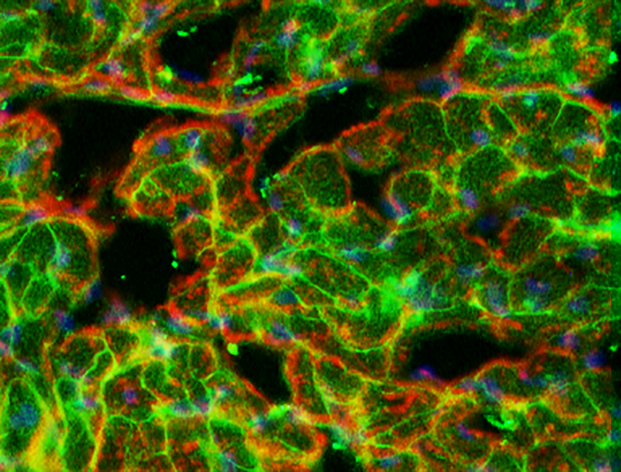

Supplement: Supplementary file 4 — Supporting File 4: advs73796‐sup‐0004‐Data.zip. [file ADVS-13-e21337-s003.zip › advs73796-sup-0004-Data/IF_Raw_Data_Figures/Figure 4K_RawData_Figures/WGA&cTnT&DAPI-AAV9-cTnT-NC-Sham-40X.tif]

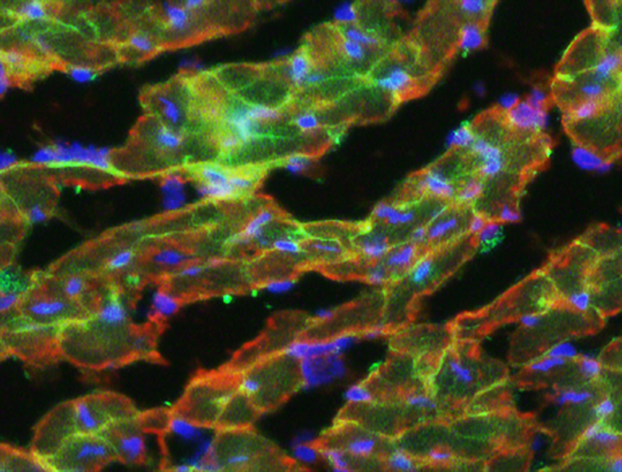

Supplement: Supplementary file 4 — Supporting File 4: advs73796‐sup‐0004‐Data.zip. [file ADVS-13-e21337-s003.zip › advs73796-sup-0004-Data/IF_Raw_Data_Figures/Figure 4K_RawData_Figures/WGA&cTnT&DAPI-AAV9-cTnT-shTRIM40-Ang II-40X.tif]

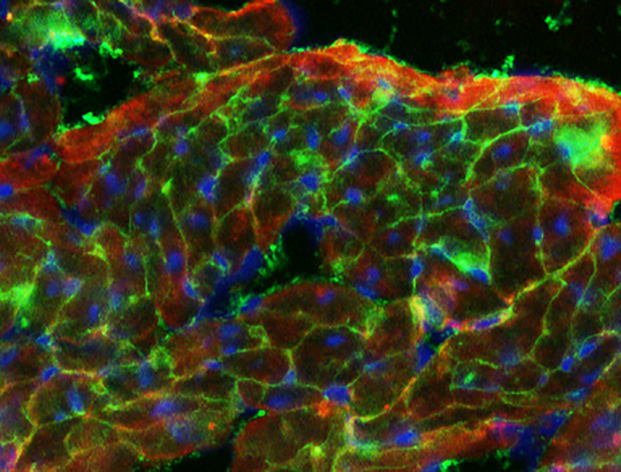

Supplement: Supplementary file 4 — Supporting File 4: advs73796‐sup‐0004‐Data.zip. [file ADVS-13-e21337-s003.zip › advs73796-sup-0004-Data/IF_Raw_Data_Figures/Figure 4K_RawData_Figures/WGA&cTnT&DAPI-AAV9-cTnT-shTRIM40-Sham.tif]

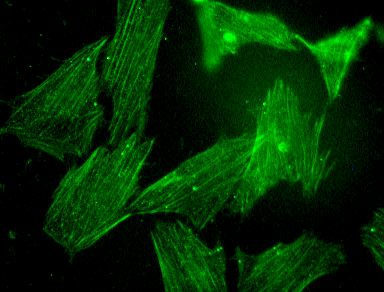

Supplement: Supplementary file 4 — Supporting File 4: advs73796‐sup‐0004‐Data.zip. [file ADVS-13-e21337-s003.zip › advs73796-sup-0004-Data/IF_Raw_Data_Figures/Figure 5A_RawData_Figures/cTnT-NC+Ang II-40X.tif]

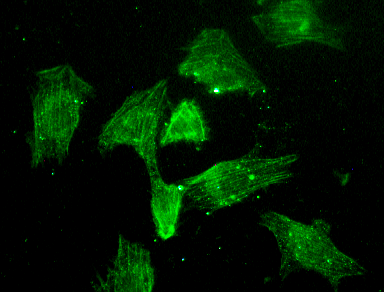

Supplement: Supplementary file 4 — Supporting File 4: advs73796‐sup‐0004‐Data.zip. [file ADVS-13-e21337-s003.zip › advs73796-sup-0004-Data/IF_Raw_Data_Figures/Figure 5A_RawData_Figures/cTnT-NC-40X.tif]

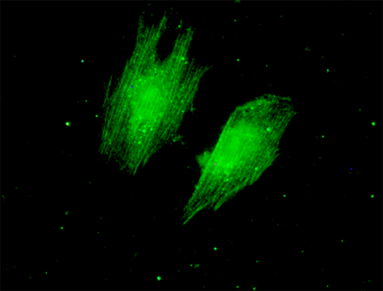

Supplement: Supplementary file 4 — Supporting File 4: advs73796‐sup‐0004‐Data.zip. [file ADVS-13-e21337-s003.zip › advs73796-sup-0004-Data/IF_Raw_Data_Figures/Figure 5A_RawData_Figures/cTnT-si TRIM40+Ang II-40X.tif]

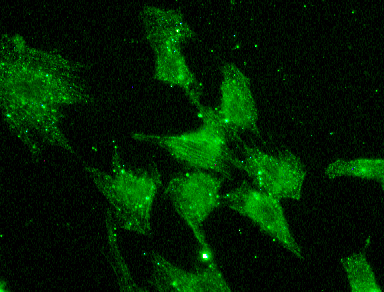

Supplement: Supplementary file 4 — Supporting File 4: advs73796‐sup‐0004‐Data.zip. [file ADVS-13-e21337-s003.zip › advs73796-sup-0004-Data/IF_Raw_Data_Figures/Figure 5A_RawData_Figures/cTnT-si TRIM40-40X.tif]

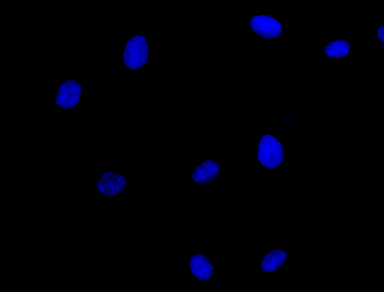

Supplement: Supplementary file 4 — Supporting File 4: advs73796‐sup‐0004‐Data.zip. [file ADVS-13-e21337-s003.zip › advs73796-sup-0004-Data/IF_Raw_Data_Figures/Figure 5A_RawData_Figures/DAPI-NC+Ang II-40X.tif]

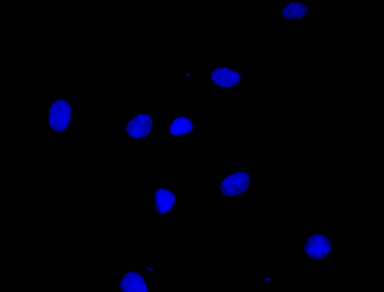

Supplement: Supplementary file 4 — Supporting File 4: advs73796‐sup‐0004‐Data.zip. [file ADVS-13-e21337-s003.zip › advs73796-sup-0004-Data/IF_Raw_Data_Figures/Figure 5A_RawData_Figures/DAPI-NC-40X-40X.tif]

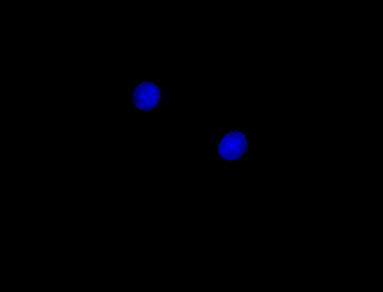

Supplement: Supplementary file 4 — Supporting File 4: advs73796‐sup‐0004‐Data.zip. [file ADVS-13-e21337-s003.zip › advs73796-sup-0004-Data/IF_Raw_Data_Figures/Figure 5A_RawData_Figures/DAPI-si TRIM40+Ang II-40X.tif]

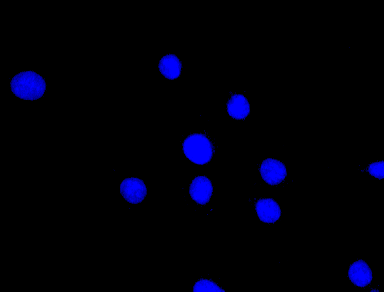

Supplement: Supplementary file 4 — Supporting File 4: advs73796‐sup‐0004‐Data.zip. [file ADVS-13-e21337-s003.zip › advs73796-sup-0004-Data/IF_Raw_Data_Figures/Figure 5A_RawData_Figures/DAPI-si TRIM40-40X.tif]

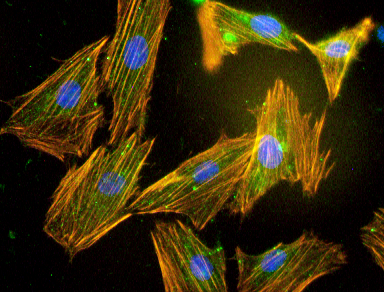

Supplement: Supplementary file 4 — Supporting File 4: advs73796‐sup‐0004‐Data.zip. [file ADVS-13-e21337-s003.zip › advs73796-sup-0004-Data/IF_Raw_Data_Figures/Figure 5A_RawData_Figures/Merge-NC+Ang II-40X.tif]

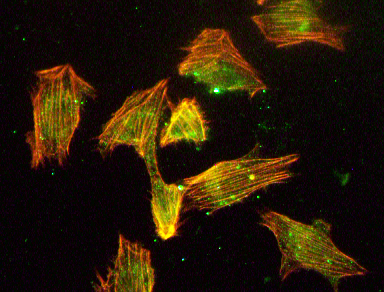

Supplement: Supplementary file 4 — Supporting File 4: advs73796‐sup‐0004‐Data.zip. [file ADVS-13-e21337-s003.zip › advs73796-sup-0004-Data/IF_Raw_Data_Figures/Figure 5A_RawData_Figures/Merge-NC-40X.tif]

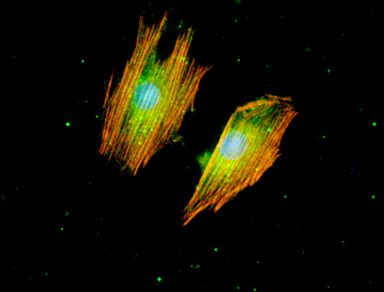

Supplement: Supplementary file 4 — Supporting File 4: advs73796‐sup‐0004‐Data.zip. [file ADVS-13-e21337-s003.zip › advs73796-sup-0004-Data/IF_Raw_Data_Figures/Figure 5A_RawData_Figures/Merge-si TRIM40+Ang II-40X.tif]

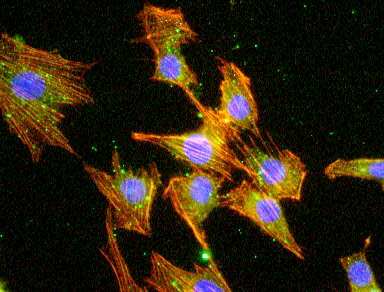

Supplement: Supplementary file 4 — Supporting File 4: advs73796‐sup‐0004‐Data.zip. [file ADVS-13-e21337-s003.zip › advs73796-sup-0004-Data/IF_Raw_Data_Figures/Figure 5A_RawData_Figures/Merge-si TRIM40-40X.tif]

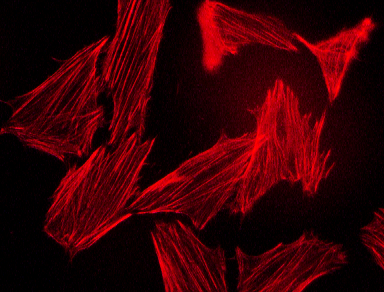

Supplement: Supplementary file 4 — Supporting File 4: advs73796‐sup‐0004‐Data.zip. [file ADVS-13-e21337-s003.zip › advs73796-sup-0004-Data/IF_Raw_Data_Figures/Figure 5A_RawData_Figures/Rhodamine-NC+Ang II-40X.tif]

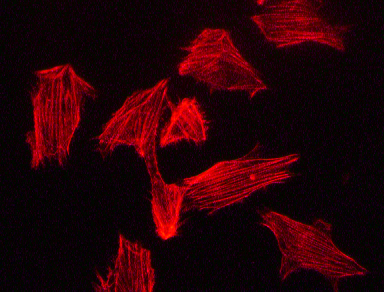

Supplement: Supplementary file 4 — Supporting File 4: advs73796‐sup‐0004‐Data.zip. [file ADVS-13-e21337-s003.zip › advs73796-sup-0004-Data/IF_Raw_Data_Figures/Figure 5A_RawData_Figures/Rhodamine-NC-40X.tif]

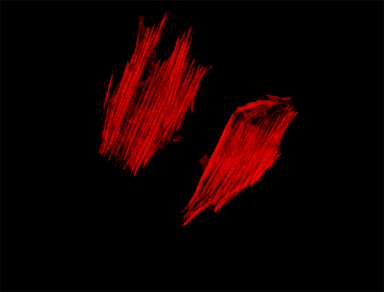

Supplement: Supplementary file 4 — Supporting File 4: advs73796‐sup‐0004‐Data.zip. [file ADVS-13-e21337-s003.zip › advs73796-sup-0004-Data/IF_Raw_Data_Figures/Figure 5A_RawData_Figures/Rhodamine-si-TRIM40+Ang II-40X.tif]

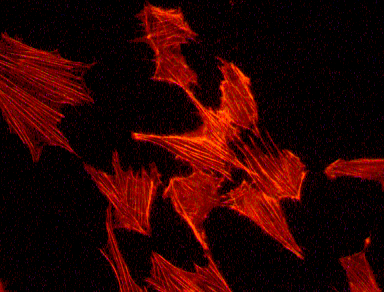

Supplement: Supplementary file 4 — Supporting File 4: advs73796‐sup‐0004‐Data.zip. [file ADVS-13-e21337-s003.zip › advs73796-sup-0004-Data/IF_Raw_Data_Figures/Figure 5A_RawData_Figures/Rhodamine-si-TRIM40-40X.tif]

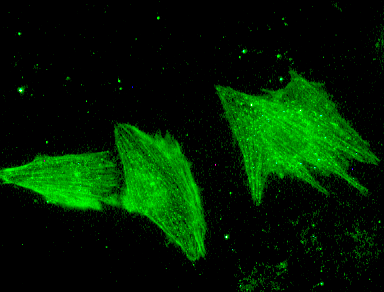

Supplement: Supplementary file 4 — Supporting File 4: advs73796‐sup‐0004‐Data.zip. [file ADVS-13-e21337-s003.zip › advs73796-sup-0004-Data/IF_Raw_Data_Figures/Figure 5F_RawData_Figures/cTnT-EV+Ang II-40X.tif]

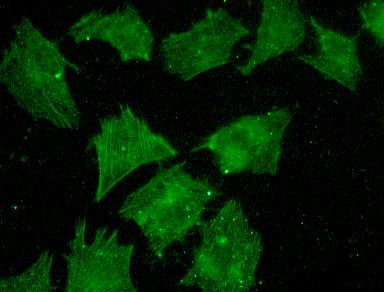

Supplement: Supplementary file 4 — Supporting File 4: advs73796‐sup‐0004‐Data.zip. [file ADVS-13-e21337-s003.zip › advs73796-sup-0004-Data/IF_Raw_Data_Figures/Figure 5F_RawData_Figures/cTnT-EV-40X.tif]

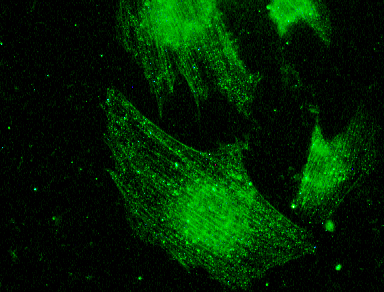

Supplement: Supplementary file 4 — Supporting File 4: advs73796‐sup‐0004‐Data.zip. [file ADVS-13-e21337-s003.zip › advs73796-sup-0004-Data/IF_Raw_Data_Figures/Figure 5F_RawData_Figures/cTnT-Flag-TRIM40+Ang II-40X.tif]

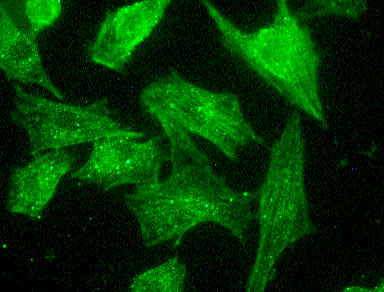

Supplement: Supplementary file 4 — Supporting File 4: advs73796‐sup‐0004‐Data.zip. [file ADVS-13-e21337-s003.zip › advs73796-sup-0004-Data/IF_Raw_Data_Figures/Figure 5F_RawData_Figures/cTnT-Flag-TRIM40-40X.tif]

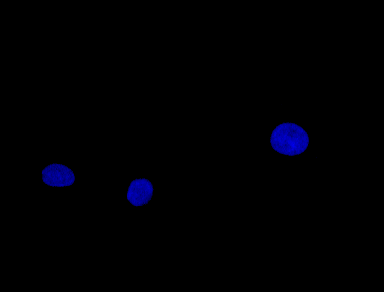

Supplement: Supplementary file 4 — Supporting File 4: advs73796‐sup‐0004‐Data.zip. [file ADVS-13-e21337-s003.zip › advs73796-sup-0004-Data/IF_Raw_Data_Figures/Figure 5F_RawData_Figures/DAPI-EV+Ang II-40X.tif]

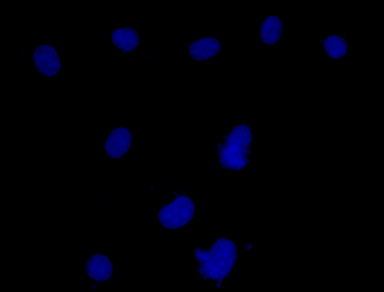

Supplement: Supplementary file 4 — Supporting File 4: advs73796‐sup‐0004‐Data.zip. [file ADVS-13-e21337-s003.zip › advs73796-sup-0004-Data/IF_Raw_Data_Figures/Figure 5F_RawData_Figures/DAPI-EV-40X.tif]

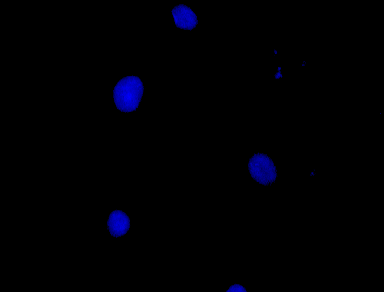

Supplement: Supplementary file 4 — Supporting File 4: advs73796‐sup‐0004‐Data.zip. [file ADVS-13-e21337-s003.zip › advs73796-sup-0004-Data/IF_Raw_Data_Figures/Figure 5F_RawData_Figures/DAPI-Flag-TRIM40+Ang II-40X.tif]

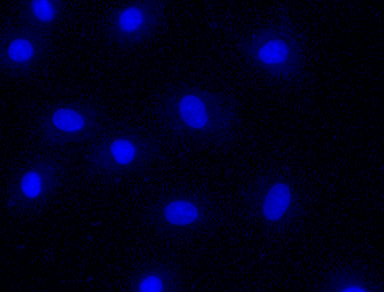

Supplement: Supplementary file 4 — Supporting File 4: advs73796‐sup‐0004‐Data.zip. [file ADVS-13-e21337-s003.zip › advs73796-sup-0004-Data/IF_Raw_Data_Figures/Figure 5F_RawData_Figures/DAPI-Flag-TRIM40-40X.tif]

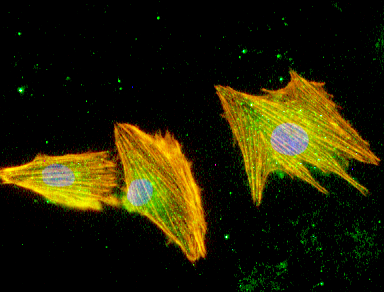

Supplement: Supplementary file 4 — Supporting File 4: advs73796‐sup‐0004‐Data.zip. [file ADVS-13-e21337-s003.zip › advs73796-sup-0004-Data/IF_Raw_Data_Figures/Figure 5F_RawData_Figures/Merge-EV+Ang II-40X.tif]

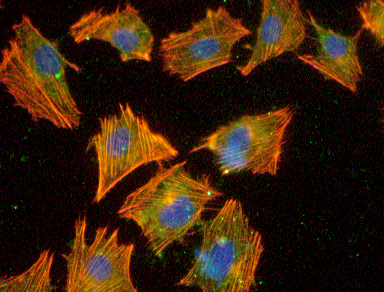

Supplement: Supplementary file 4 — Supporting File 4: advs73796‐sup‐0004‐Data.zip. [file ADVS-13-e21337-s003.zip › advs73796-sup-0004-Data/IF_Raw_Data_Figures/Figure 5F_RawData_Figures/Merge-EV-40X.tif]

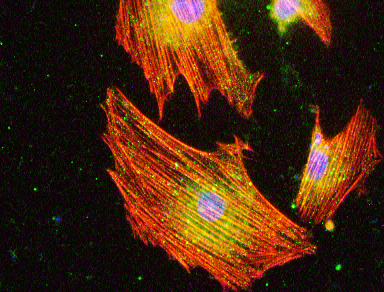

Supplement: Supplementary file 4 — Supporting File 4: advs73796‐sup‐0004‐Data.zip. [file ADVS-13-e21337-s003.zip › advs73796-sup-0004-Data/IF_Raw_Data_Figures/Figure 5F_RawData_Figures/Merge-Flag-TRIM40+Ang II-40X.tif]

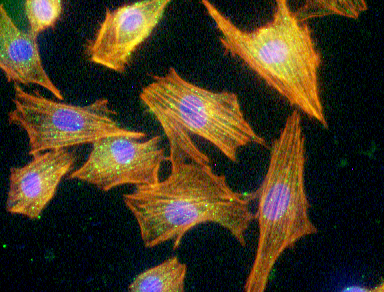

Supplement: Supplementary file 4 — Supporting File 4: advs73796‐sup‐0004‐Data.zip. [file ADVS-13-e21337-s003.zip › advs73796-sup-0004-Data/IF_Raw_Data_Figures/Figure 5F_RawData_Figures/Merge-Flag-TRIM40-40X.tif]

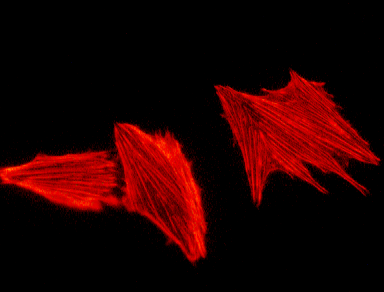

Supplement: Supplementary file 4 — Supporting File 4: advs73796‐sup‐0004‐Data.zip. [file ADVS-13-e21337-s003.zip › advs73796-sup-0004-Data/IF_Raw_Data_Figures/Figure 5F_RawData_Figures/Rhodamine-EV+Ang II-40X.tif]

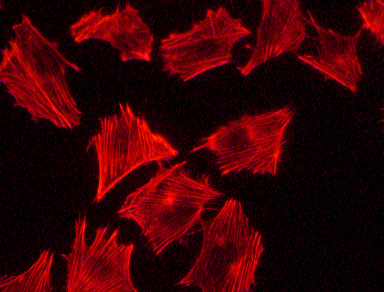

Supplement: Supplementary file 4 — Supporting File 4: advs73796‐sup‐0004‐Data.zip. [file ADVS-13-e21337-s003.zip › advs73796-sup-0004-Data/IF_Raw_Data_Figures/Figure 5F_RawData_Figures/Rhodamine-EV-40X.tif]

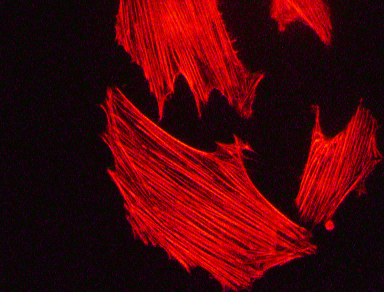

Supplement: Supplementary file 4 — Supporting File 4: advs73796‐sup‐0004‐Data.zip. [file ADVS-13-e21337-s003.zip › advs73796-sup-0004-Data/IF_Raw_Data_Figures/Figure 5F_RawData_Figures/Rhodamine-Flag-TRIM40+Ang II-40X.tif]

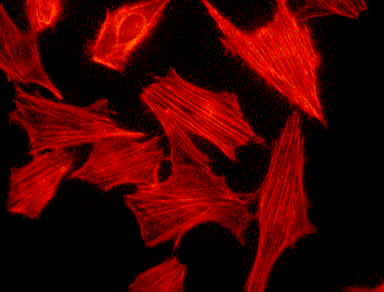

Supplement: Supplementary file 4 — Supporting File 4: advs73796‐sup‐0004‐Data.zip. [file ADVS-13-e21337-s003.zip › advs73796-sup-0004-Data/IF_Raw_Data_Figures/Figure 5F_RawData_Figures/Rhodamine-Flag-TRIM40-40X.tif]

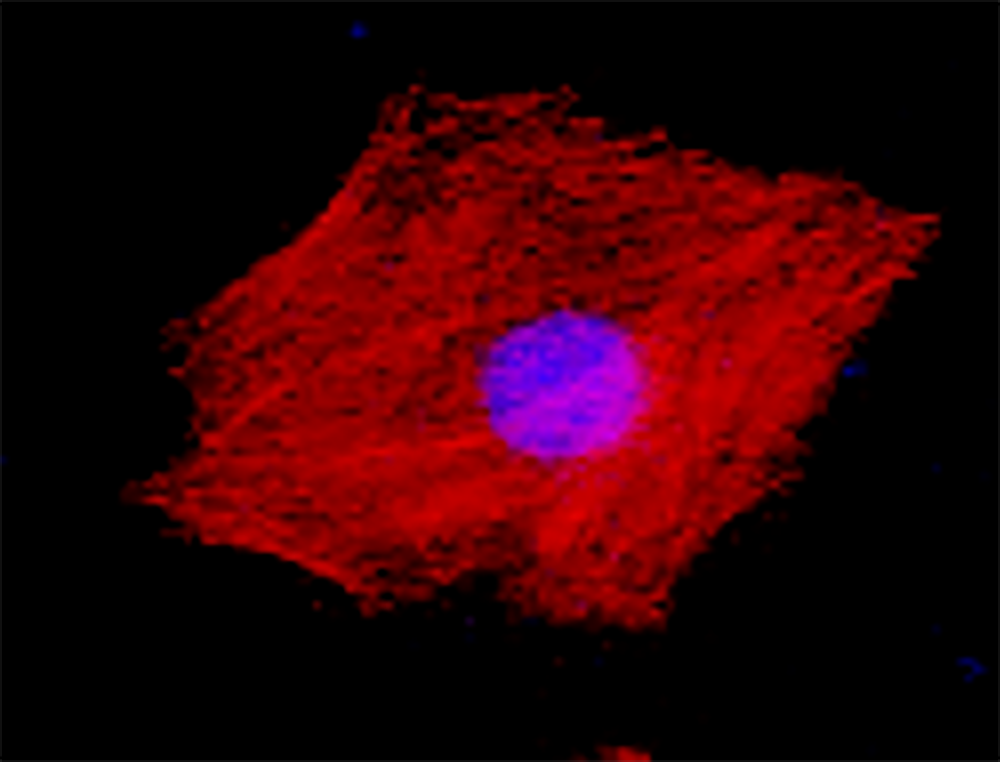

Supplement: Supplementary file 4 — Supporting File 4: advs73796‐sup‐0004‐Data.zip. [file ADVS-13-e21337-s003.zip › advs73796-sup-0004-Data/IF_Raw_Data_Figures/Figure 6L_RawData_Figures/Rhodamine-Ang II-Flag-TRIM40-40X.tif]

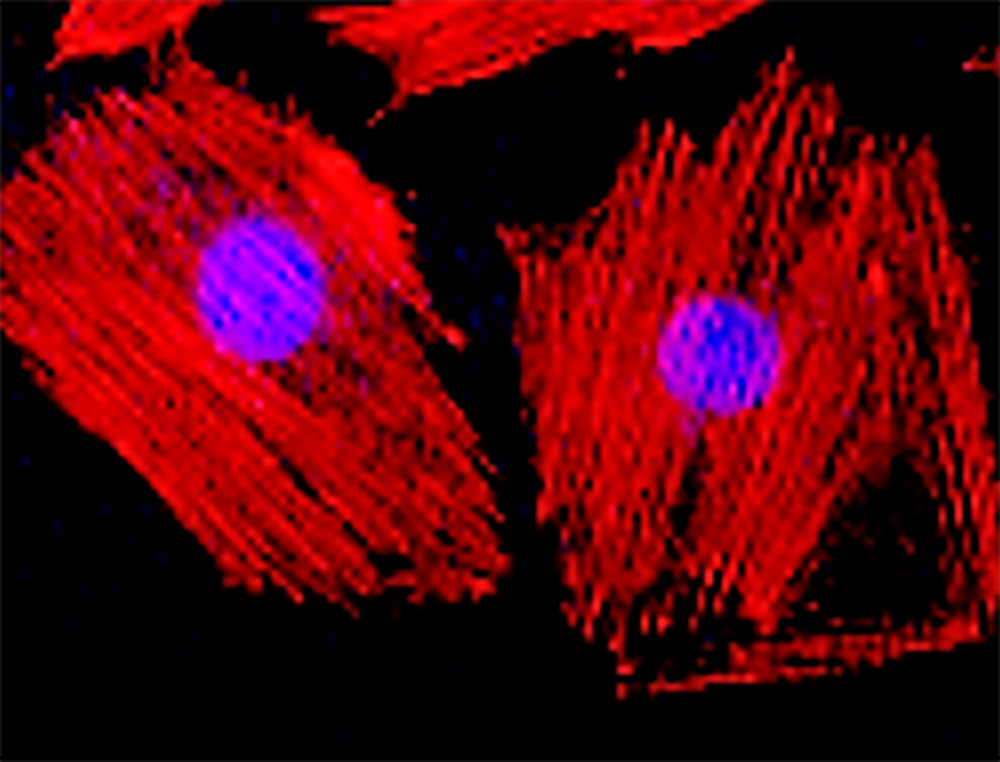

Supplement: Supplementary file 4 — Supporting File 4: advs73796‐sup‐0004‐Data.zip. [file ADVS-13-e21337-s003.zip › advs73796-sup-0004-Data/IF_Raw_Data_Figures/Figure 6L_RawData_Figures/Rhodamine-Ang II-Flag-TRIM40-C29S-40X.tif]

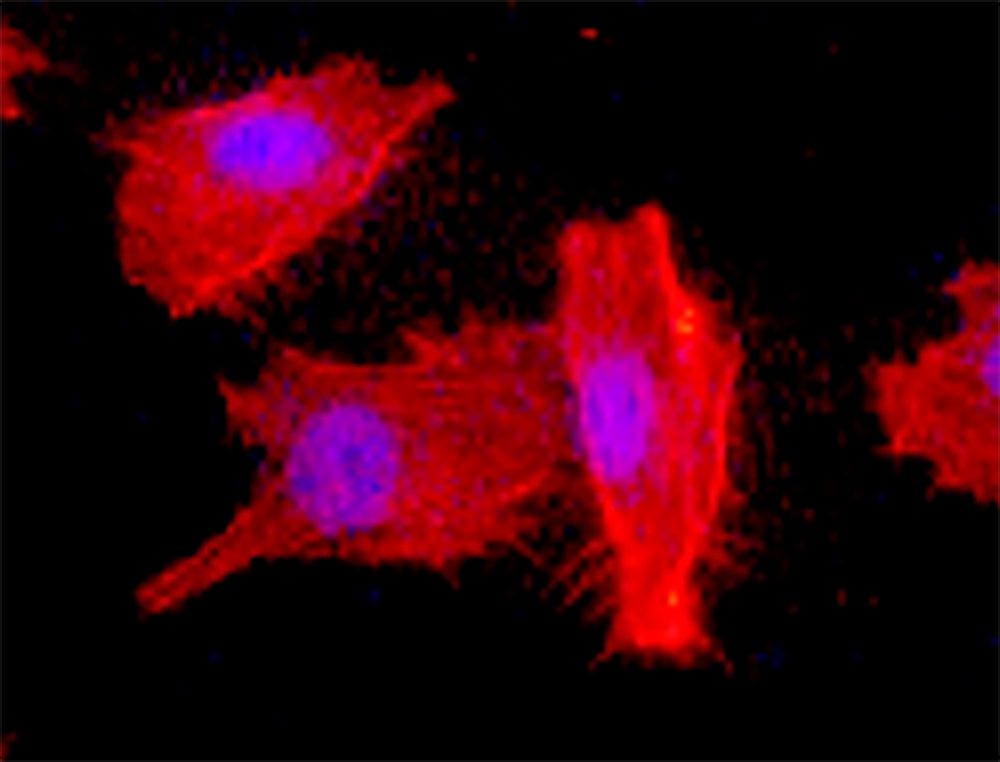

Supplement: Supplementary file 4 — Supporting File 4: advs73796‐sup‐0004‐Data.zip. [file ADVS-13-e21337-s003.zip › advs73796-sup-0004-Data/IF_Raw_Data_Figures/Figure 6L_RawData_Figures/Rhodamine-Ang II-Flag-TRIM40ΔBB-40X.tif]

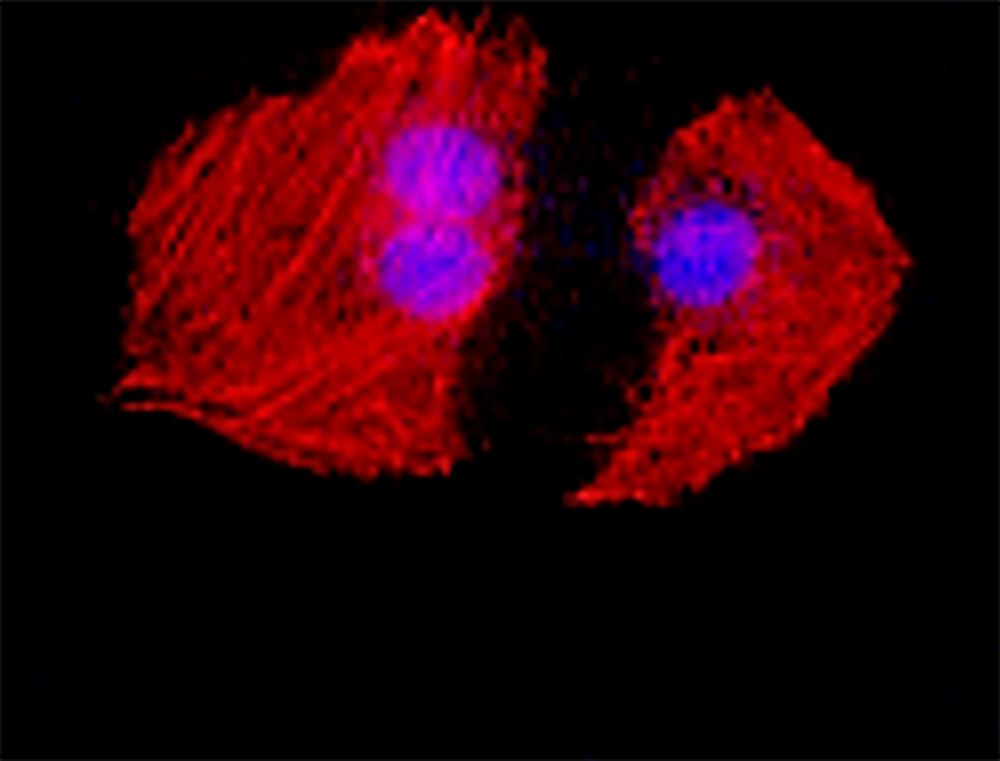

Supplement: Supplementary file 4 — Supporting File 4: advs73796‐sup‐0004‐Data.zip. [file ADVS-13-e21337-s003.zip › advs73796-sup-0004-Data/IF_Raw_Data_Figures/Figure 6L_RawData_Figures/Rhodamine-Ang II-Flag-TRIM40ΔCC-40X.tif]

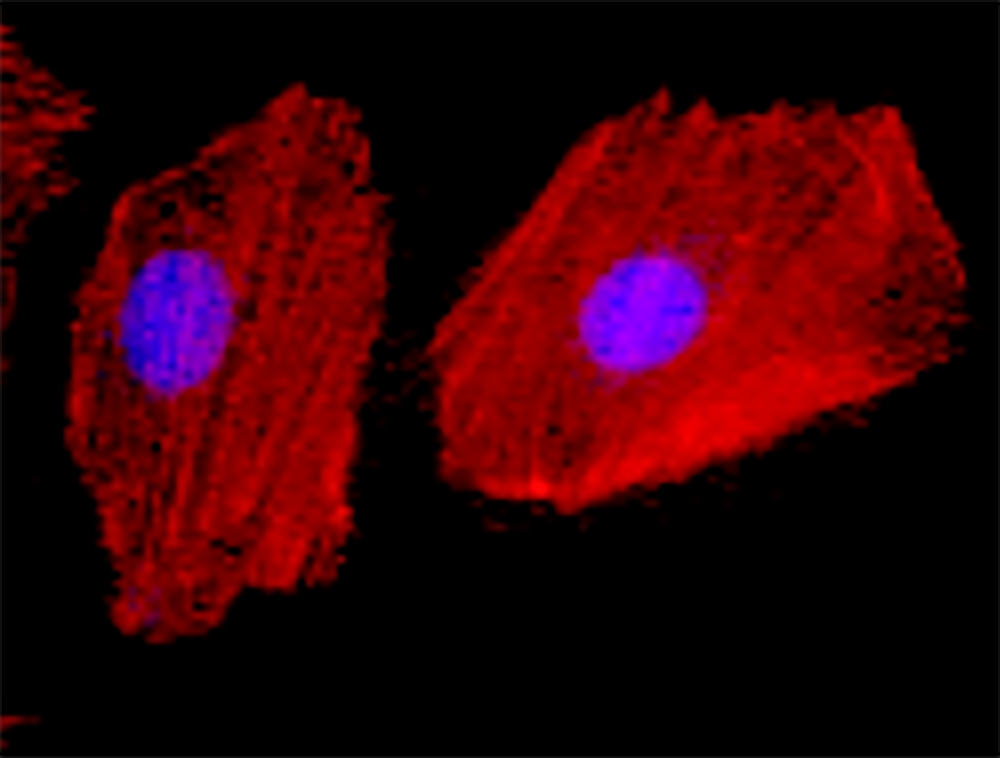

Supplement: Supplementary file 4 — Supporting File 4: advs73796‐sup‐0004‐Data.zip. [file ADVS-13-e21337-s003.zip › advs73796-sup-0004-Data/IF_Raw_Data_Figures/Figure 6L_RawData_Figures/Rhodamine-Ang II-Flag-TRIM40ΔCT-40X.tif]

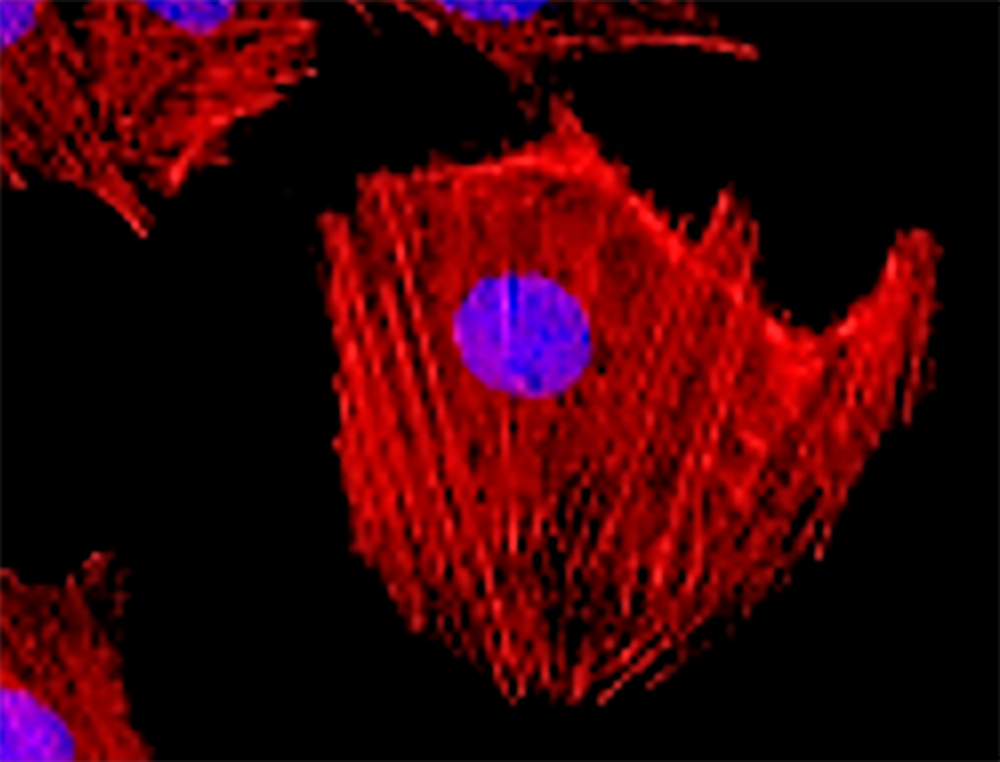

Supplement: Supplementary file 4 — Supporting File 4: advs73796‐sup‐0004‐Data.zip. [file ADVS-13-e21337-s003.zip › advs73796-sup-0004-Data/IF_Raw_Data_Figures/Figure 6L_RawData_Figures/Rhodamine-Ang II-Flag-TRIM40ΔRING-40X.tif]

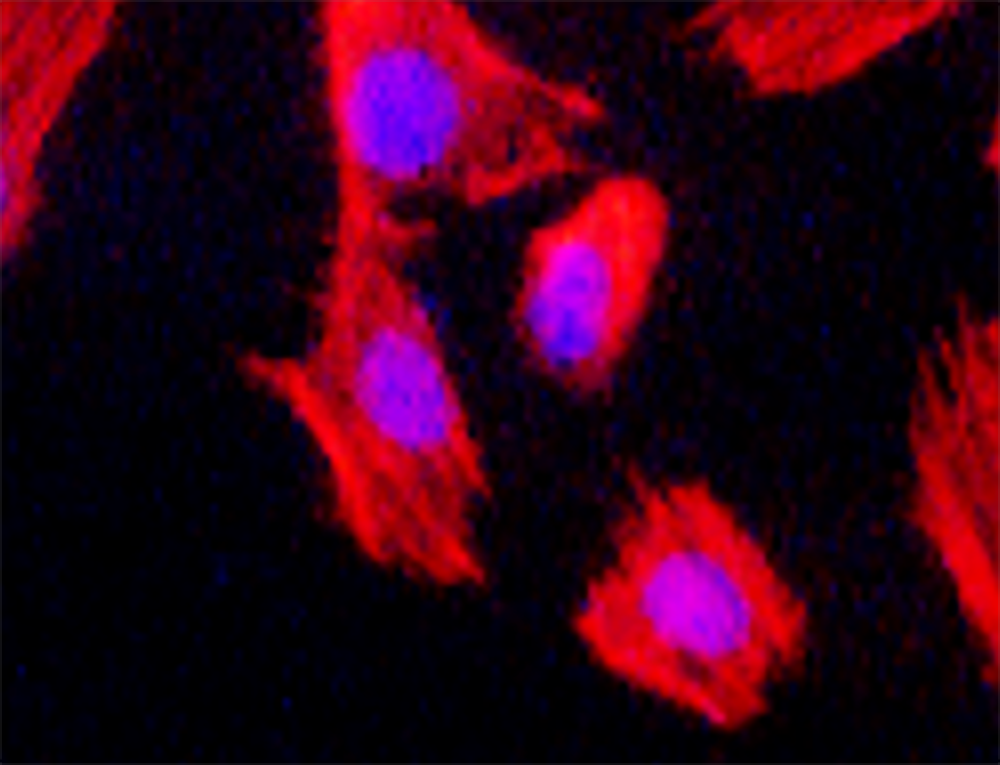

Supplement: Supplementary file 4 — Supporting File 4: advs73796‐sup‐0004‐Data.zip. [file ADVS-13-e21337-s003.zip › advs73796-sup-0004-Data/IF_Raw_Data_Figures/Figure 6L_RawData_Figures/Rhodamine-EV-40X.tif]

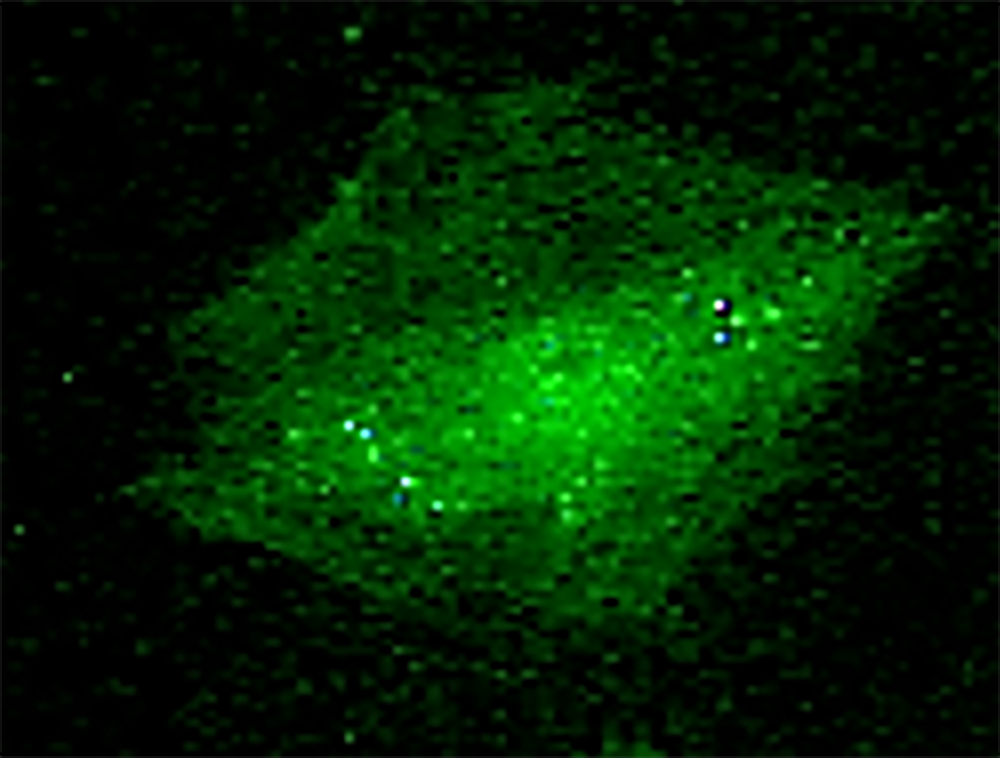

Supplement: Supplementary file 4 — Supporting File 4: advs73796‐sup‐0004‐Data.zip. [file ADVS-13-e21337-s003.zip › advs73796-sup-0004-Data/IF_Raw_Data_Figures/Figure 6L_RawData_Figures/TRIM40-Ang II-Flag-TRIM40-40X.tif]

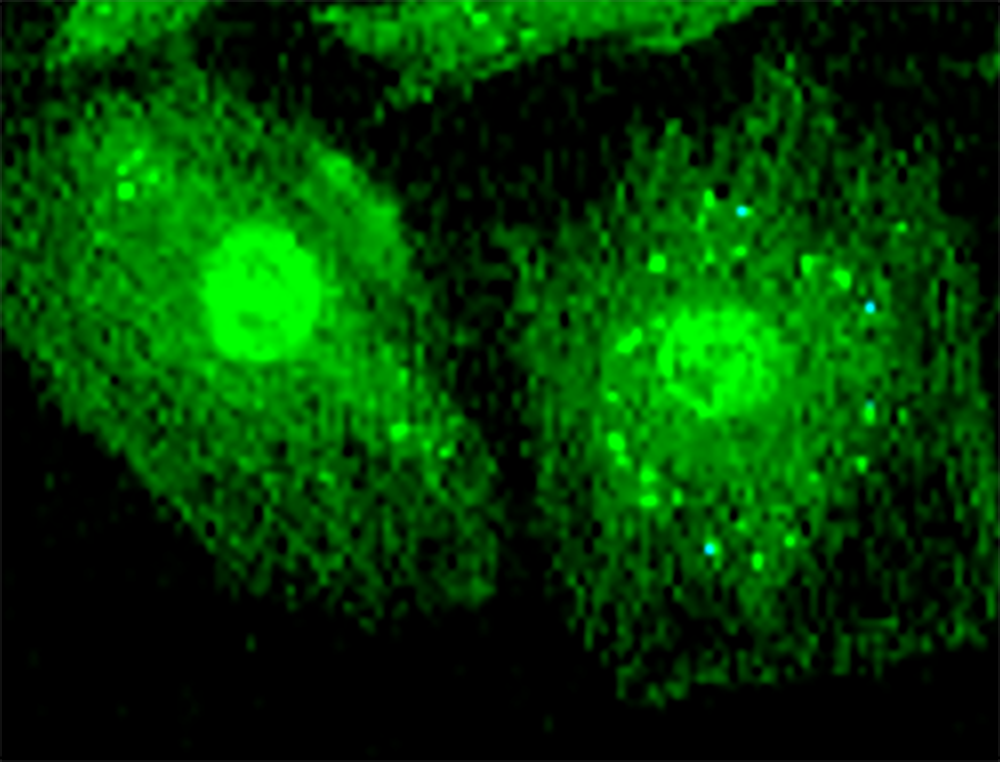

Supplement: Supplementary file 4 — Supporting File 4: advs73796‐sup‐0004‐Data.zip. [file ADVS-13-e21337-s003.zip › advs73796-sup-0004-Data/IF_Raw_Data_Figures/Figure 6L_RawData_Figures/TRIM40-Ang II-Flag-TRIM40-C29S-40X.tif]

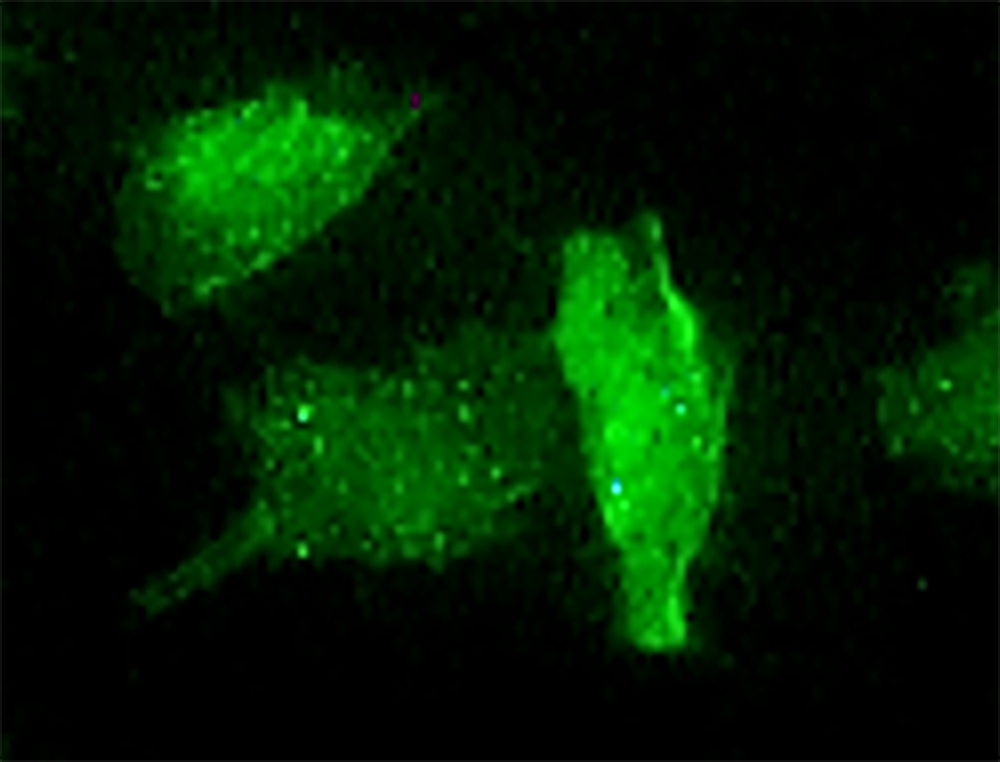

Supplement: Supplementary file 4 — Supporting File 4: advs73796‐sup‐0004‐Data.zip. [file ADVS-13-e21337-s003.zip › advs73796-sup-0004-Data/IF_Raw_Data_Figures/Figure 6L_RawData_Figures/TRIM40-Ang II-Flag-TRIM40ΔBB-40X.tif]

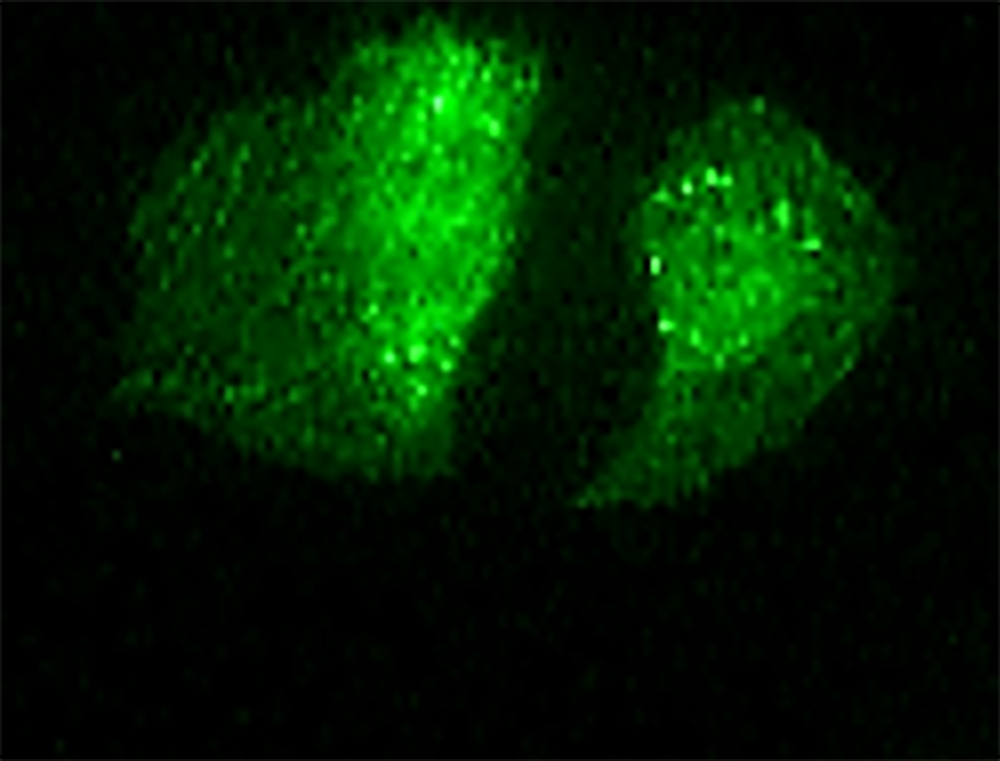

Supplement: Supplementary file 4 — Supporting File 4: advs73796‐sup‐0004‐Data.zip. [file ADVS-13-e21337-s003.zip › advs73796-sup-0004-Data/IF_Raw_Data_Figures/Figure 6L_RawData_Figures/TRIM40-Ang II-Flag-TRIM40ΔCC-40X.tif]

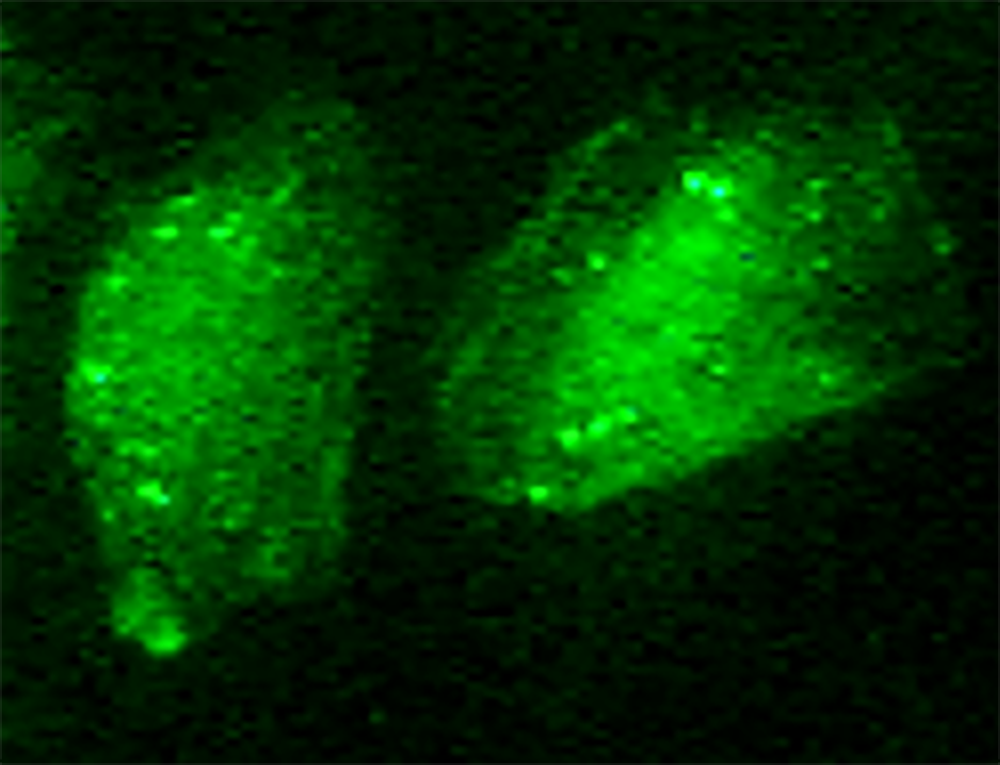

Supplement: Supplementary file 4 — Supporting File 4: advs73796‐sup‐0004‐Data.zip. [file ADVS-13-e21337-s003.zip › advs73796-sup-0004-Data/IF_Raw_Data_Figures/Figure 6L_RawData_Figures/TRIM40-Ang II-Flag-TRIM40ΔCT-40X.tif]

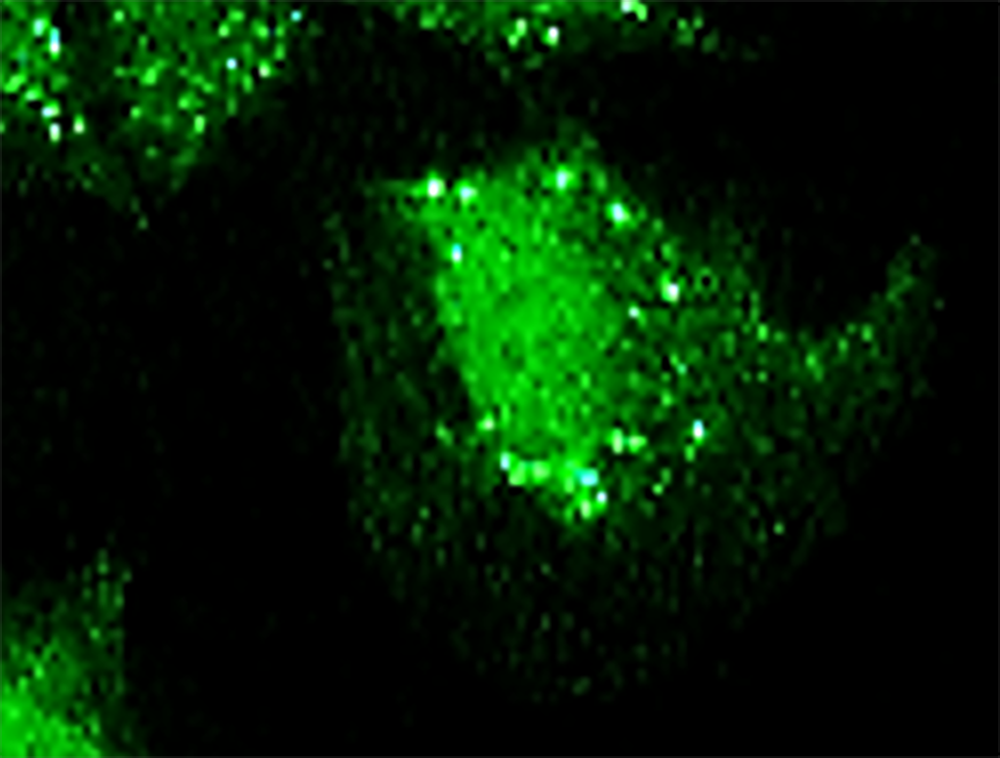

Supplement: Supplementary file 4 — Supporting File 4: advs73796‐sup‐0004‐Data.zip. [file ADVS-13-e21337-s003.zip › advs73796-sup-0004-Data/IF_Raw_Data_Figures/Figure 6L_RawData_Figures/TRIM40-Ang II-Flag-TRIM40ΔRING-40X.tif]

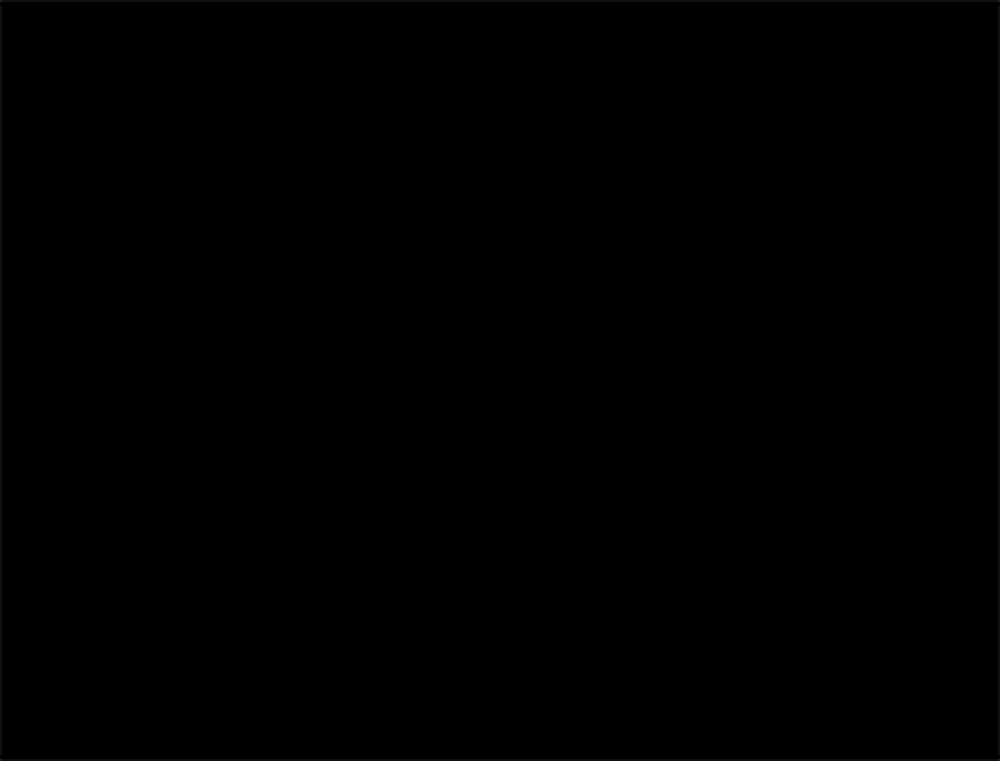

Supplement: Supplementary file 4 — Supporting File 4: advs73796‐sup‐0004‐Data.zip. [file ADVS-13-e21337-s003.zip › advs73796-sup-0004-Data/IF_Raw_Data_Figures/Figure 6L_RawData_Figures/TRIM40-EV-40X.tif]

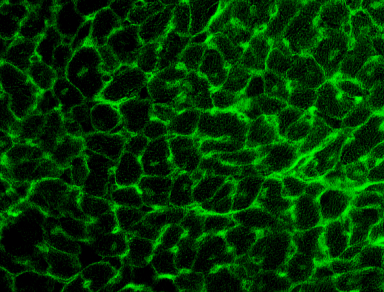

Supplement: Supplementary file 4 — Supporting File 4: advs73796‐sup‐0004‐Data.zip. [file ADVS-13-e21337-s003.zip › advs73796-sup-0004-Data/IF_Raw_Data_Figures/Figure 8J_RawData_Figures/WGA-WT-AAV-NC-40X.tif]

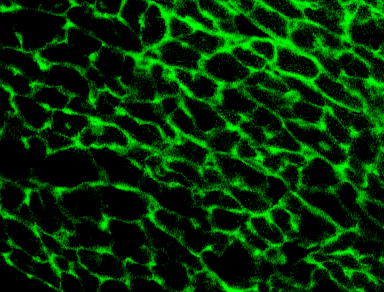

Supplement: Supplementary file 4 — Supporting File 4: advs73796‐sup‐0004‐Data.zip. [file ADVS-13-e21337-s003.zip › advs73796-sup-0004-Data/IF_Raw_Data_Figures/Figure 8J_RawData_Figures/WGA-WT-AAV-TRIM40-40X.tif]

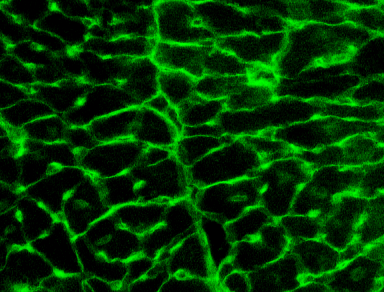

Supplement: Supplementary file 4 — Supporting File 4: advs73796‐sup‐0004‐Data.zip. [file ADVS-13-e21337-s003.zip › advs73796-sup-0004-Data/IF_Raw_Data_Figures/Figure 8J_RawData_Figures/WGA-WT-Ang II-AAV-NC-40X.tif]

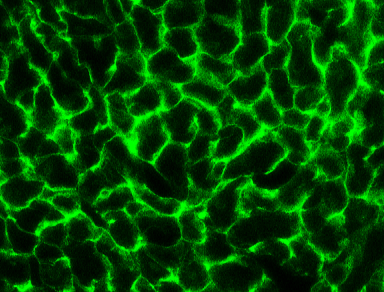

Supplement: Supplementary file 4 — Supporting File 4: advs73796‐sup‐0004‐Data.zip. [file ADVS-13-e21337-s003.zip › advs73796-sup-0004-Data/IF_Raw_Data_Figures/Figure 8J_RawData_Figures/WGA-WT-Ang II-AAV-TRIM40+PKN1 or 2-IN-1-40X.tif]

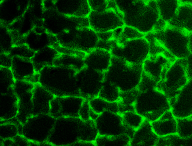

Supplement: Supplementary file 4 — Supporting File 4: advs73796‐sup‐0004‐Data.zip. [file ADVS-13-e21337-s003.zip › advs73796-sup-0004-Data/IF_Raw_Data_Figures/Figure 8J_RawData_Figures/WGA-WT-Ang II-AAV-TRIM40-40X.tif]

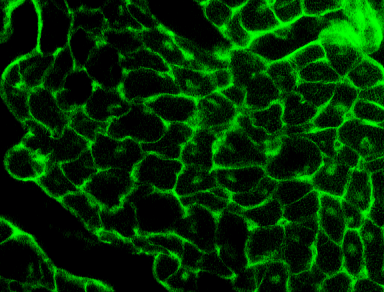

Supplement: Supplementary file 4 — Supporting File 4: advs73796‐sup‐0004‐Data.zip. [file ADVS-13-e21337-s003.zip › advs73796-sup-0004-Data/IF_Raw_Data_Figures/Figure 9H_RawData_Figures/WGA-WT+TAC-AAV-NC-40X.tif]
