# Supplementary material for: TRIM40 Drives Pathological Cardiac Hypertrophy and Heart Failure via Ubiquitination of PKN2
Source: Adv Sci (Weinh). 2026 Jan 22;13(17):e21337. doi: 10.1002/advs.202521337 (PMC13042792; doi:10.1002/advs.202521337)
Supplement: Supplementary file 3 — Supporting File 3: advs73796‐sup‐0003‐Western Blot_Raw_Data_Figures.pdf. [file ADVS-13-e21337-s004.pdf]

Figure 1B

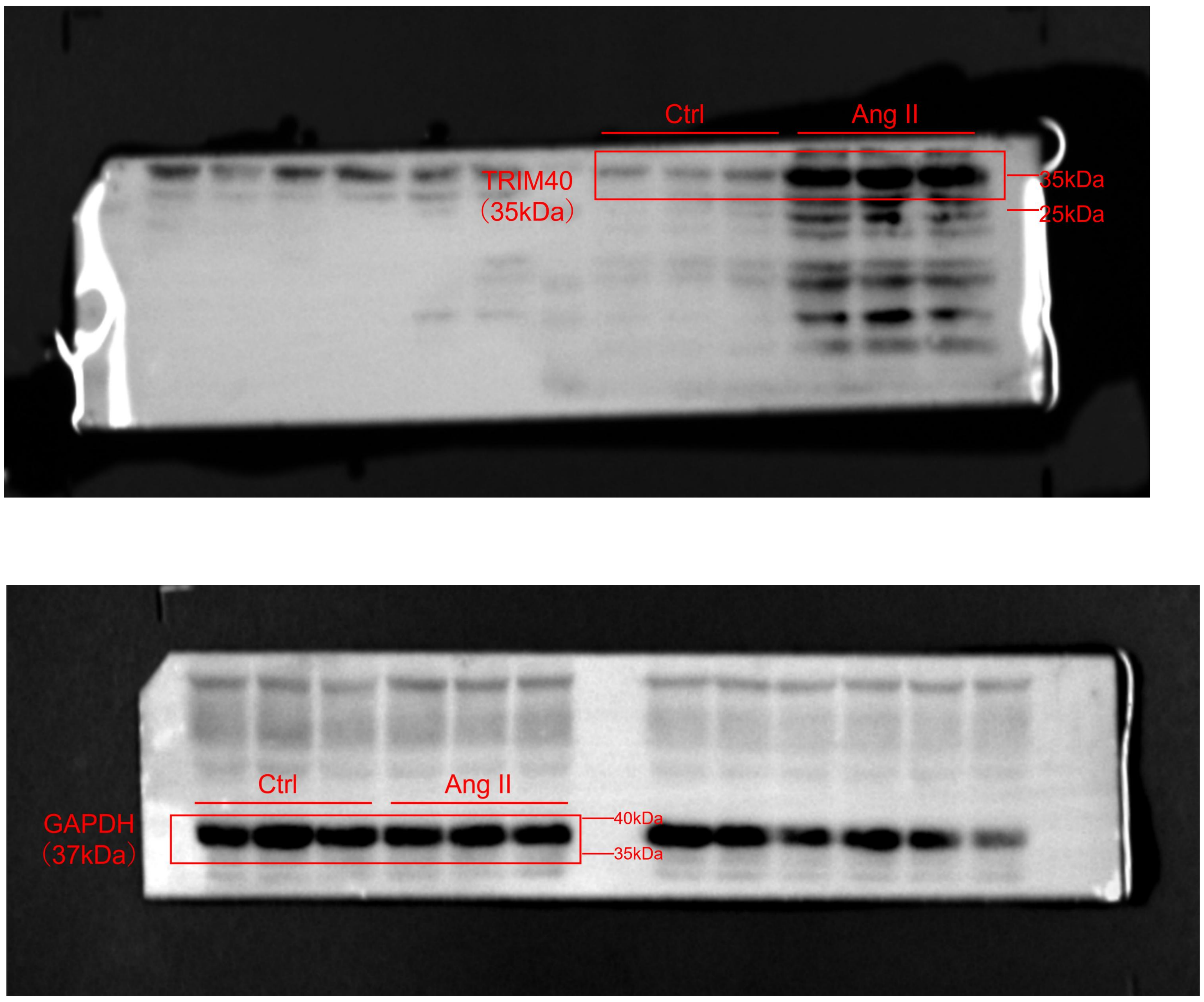

Figure 1C

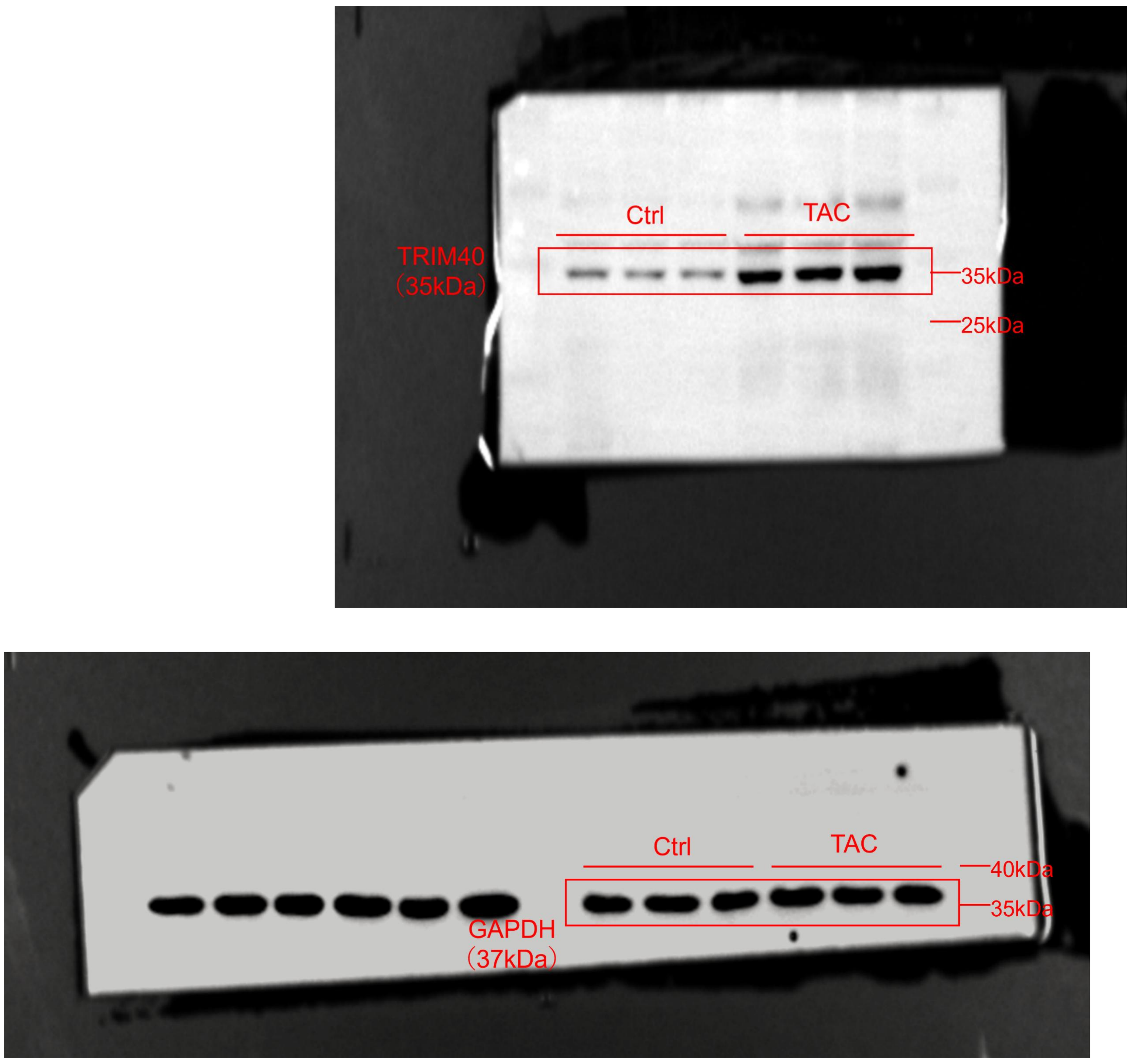

Figure 1E

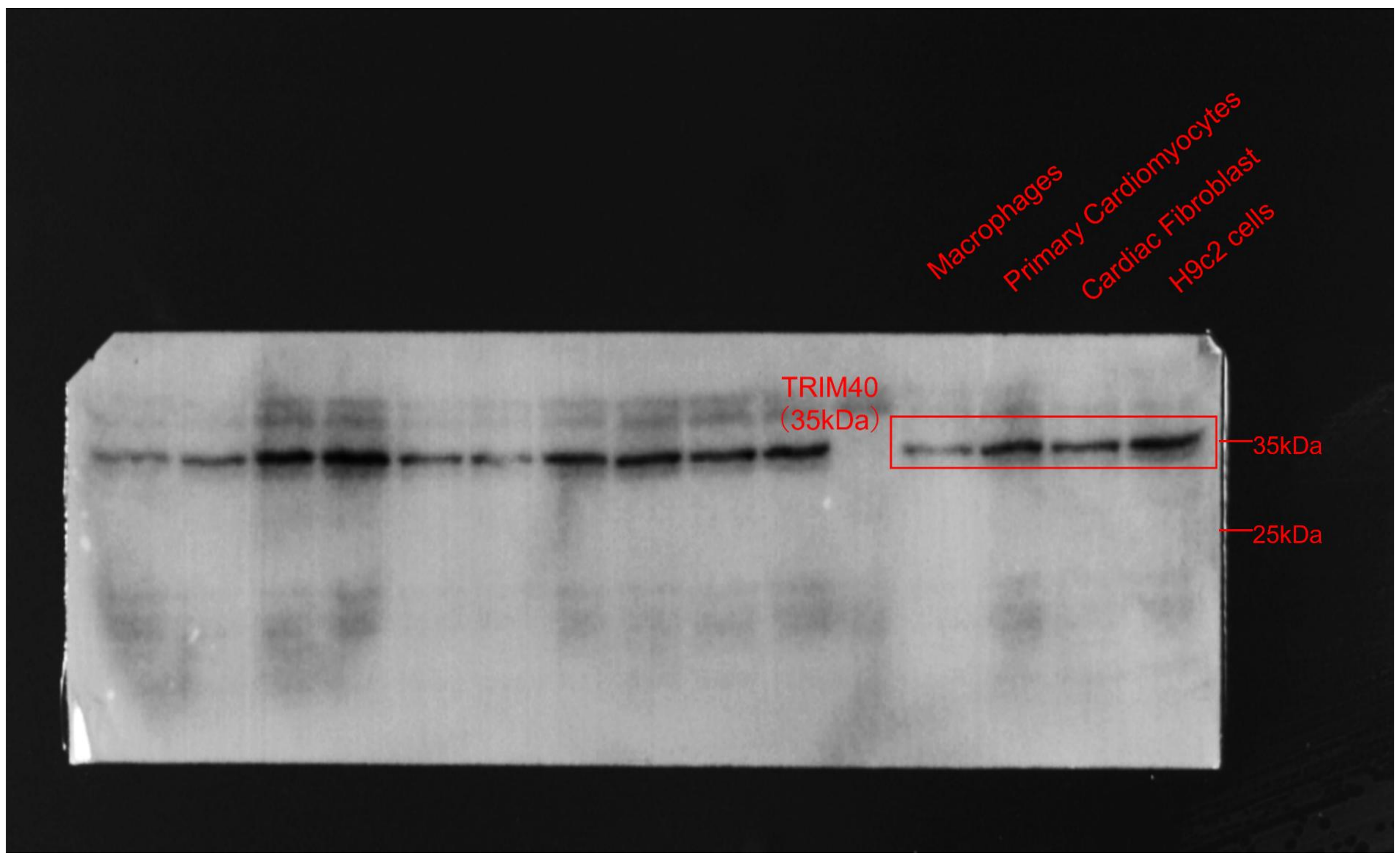
Macrophages
Primary Cardiomyocytes
Cardiac Fibroblast
H9c2 cells
TRIM40
(35kDa)
35kDa
25kDa

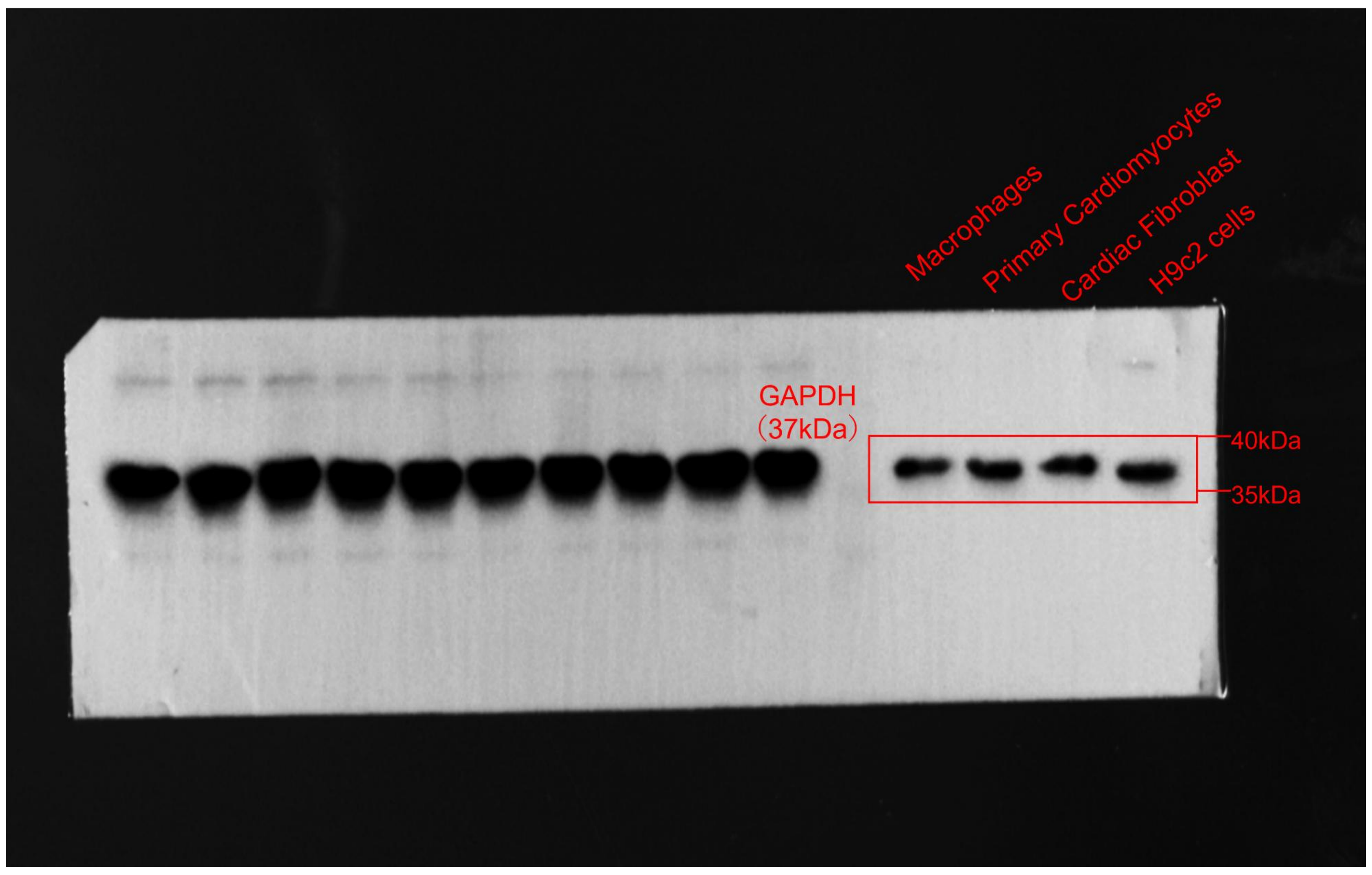
Macrophages
Primary Cardiomyocytes
Cardiac Fibroblast
H9c2 cells
GAPDH
(37kDa)
40kDa
35kDa

Figure 2N

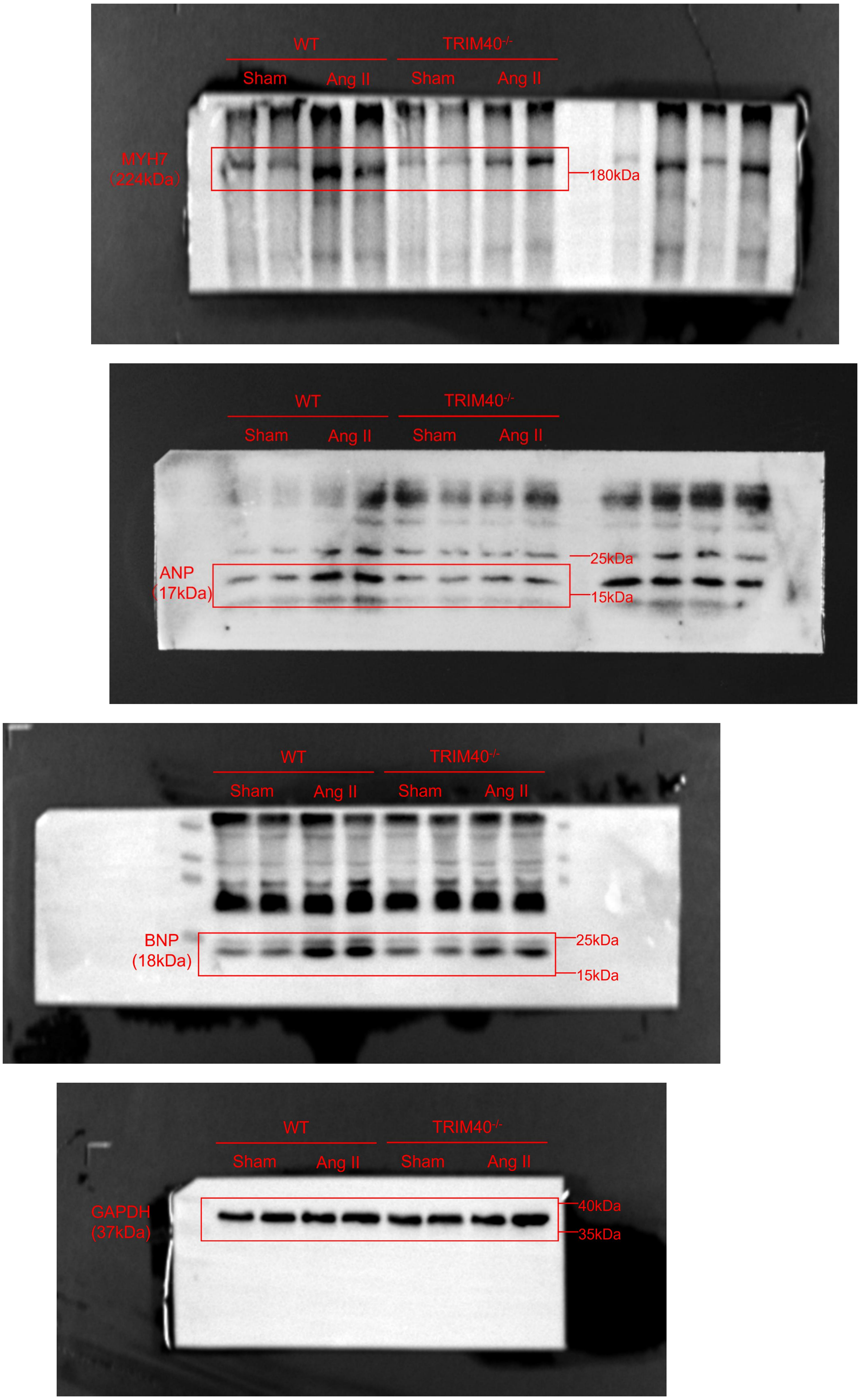
WT
TRIM40-/-
Sham
Ang II
Sham
Ang II
MYH7
(224kDa)
180kDa
WT
TRIM40-/-
Sham
Ang II
Sham
Ang II
ANP
(17kDa)
25kDa
15kDa
WT
TRIM40-/-
Sham
Ang II
Sham
Ang II
BNP
(18kDa)
25kDa
15kDa
WT
TRIM40-/-
Sham
Ang II
Sham
Ang II
GAPDH
(37kDa)
40kDa
35kDa

Figure 3M

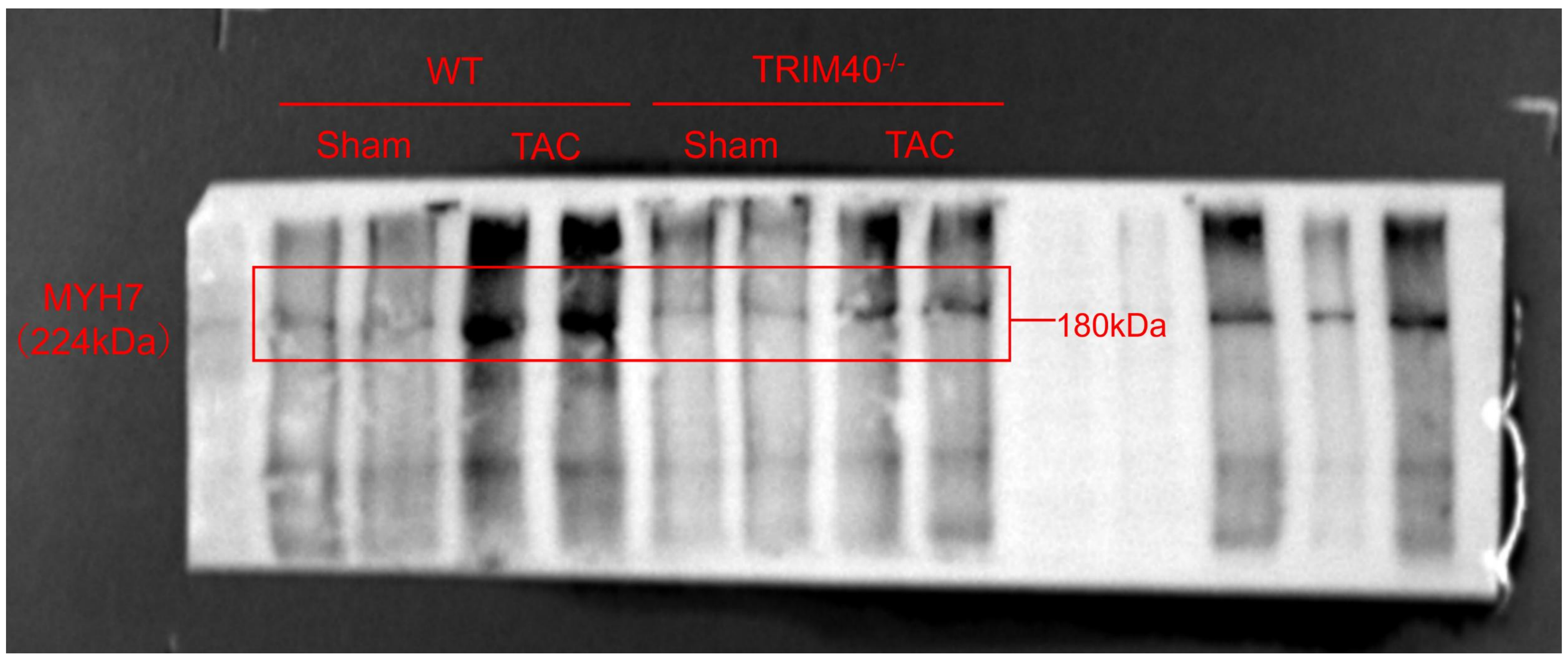

WT
TRIM40-/-
Sham
TAC
Sham
TAC
MYH7
(224kDa)
180kDa

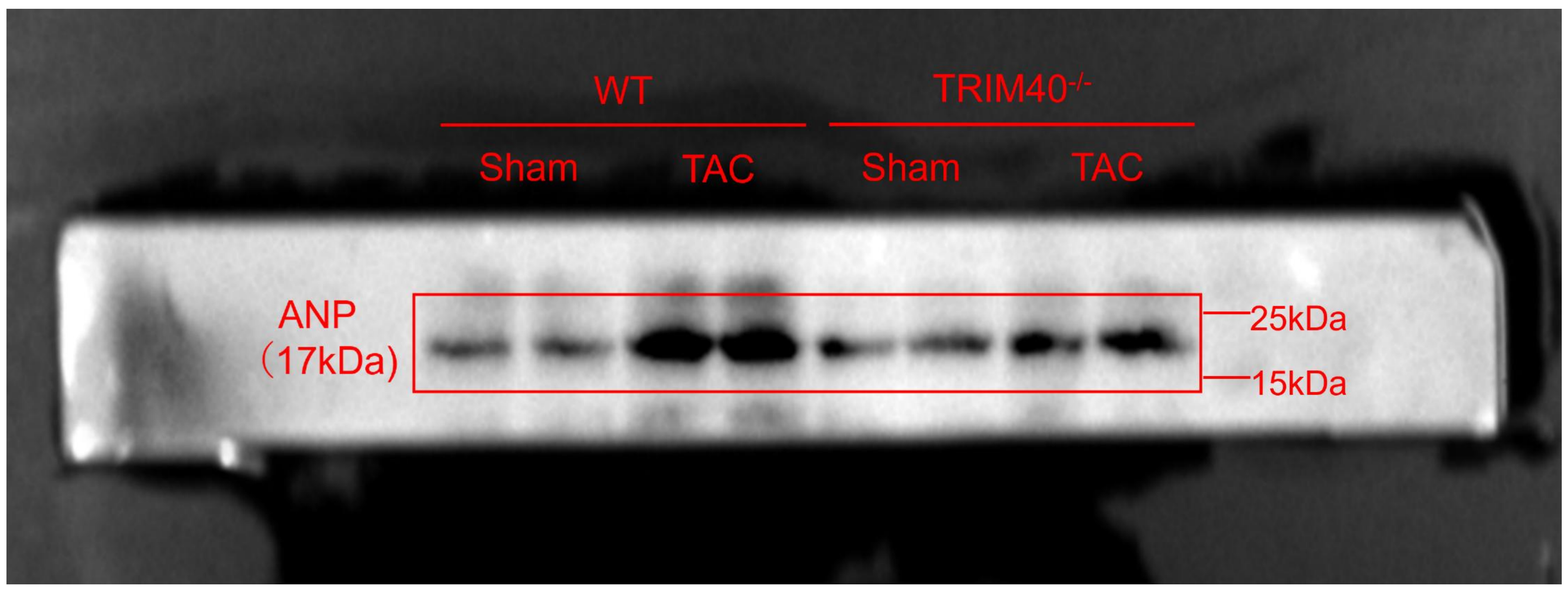

WT
TRIM40-/-
Sham
TAC
Sham
TAC
ANP
(17kDa)
25kDa
15kDa

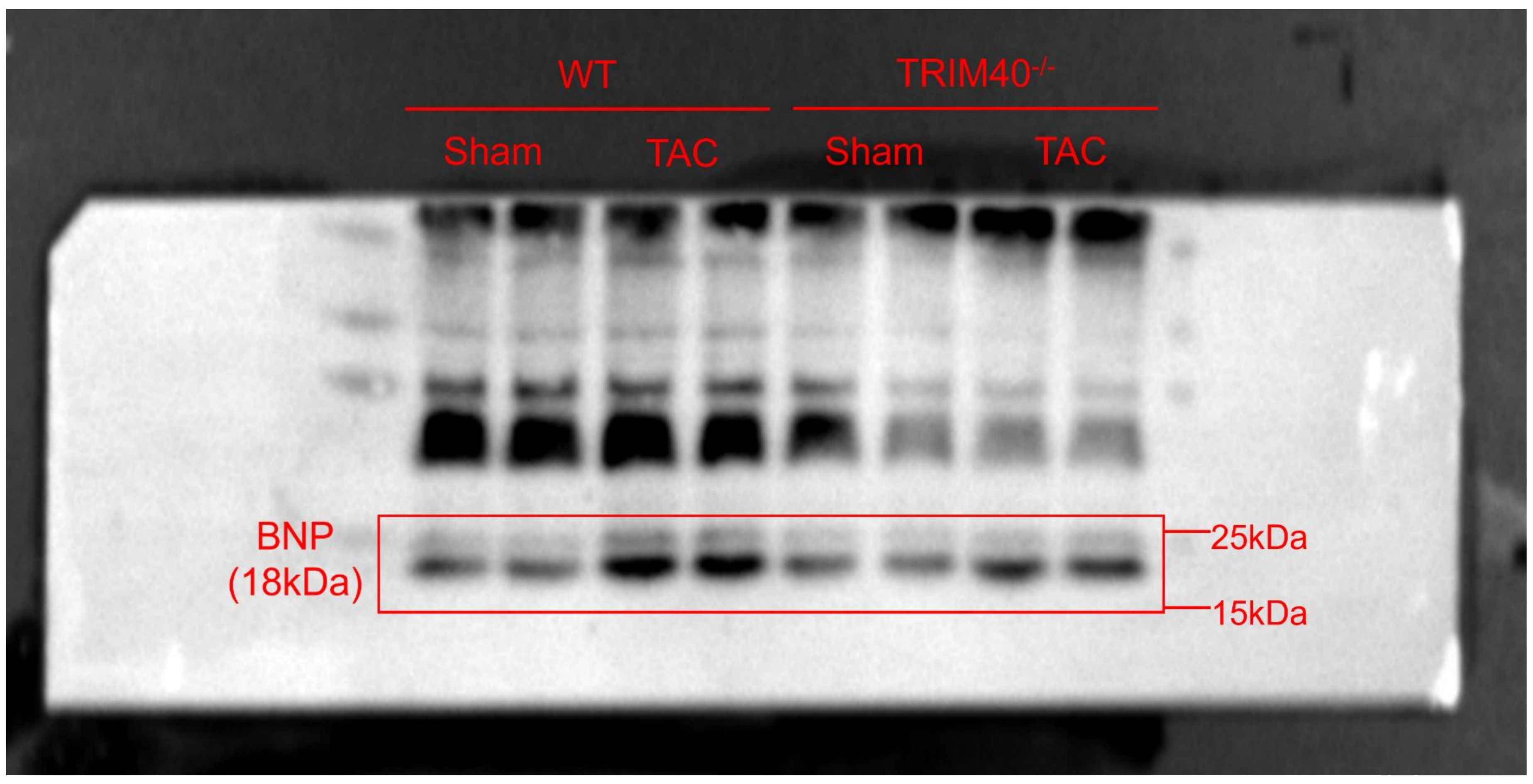

WT
TRIM40-/-
Sham
TAC
Sham
TAC
BNP
(18kDa)
25kDa
15kDa

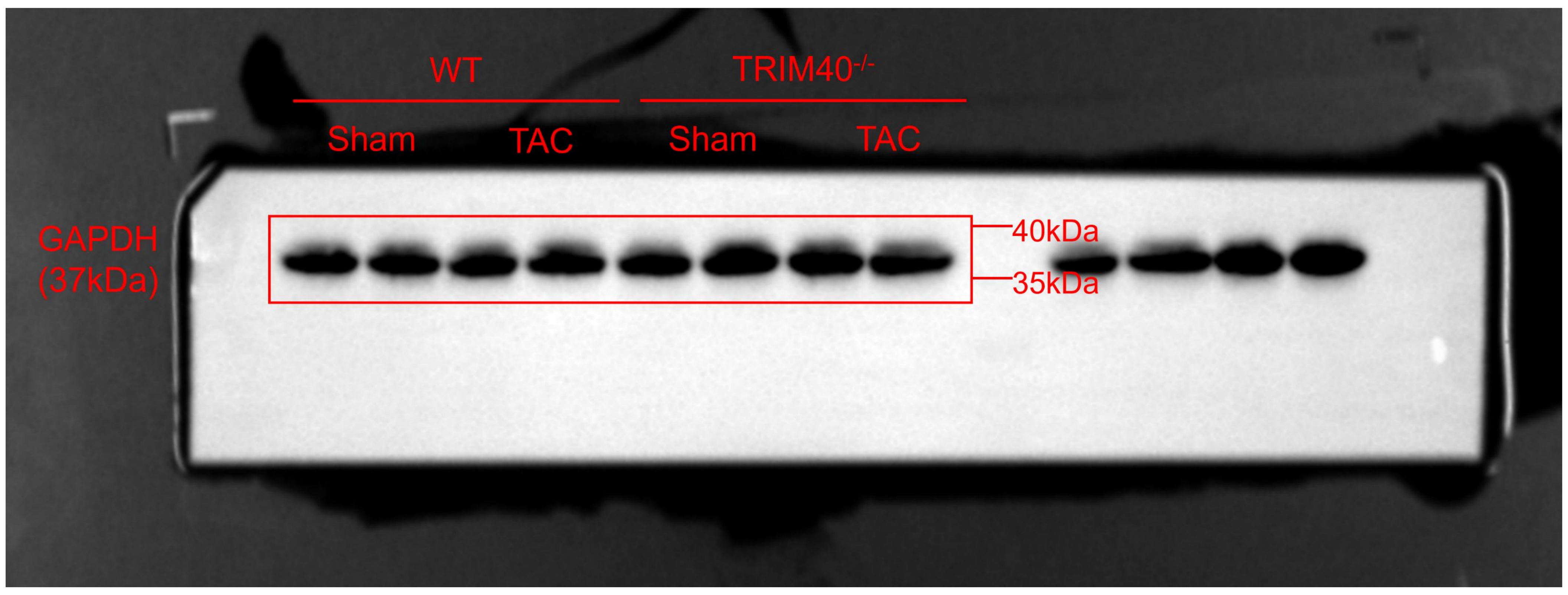

WT
TRIM40-/-
Sham
TAC
Sham
TAC
GAPDH
(37kDa)
40kDa
35kDa

Figure 4P

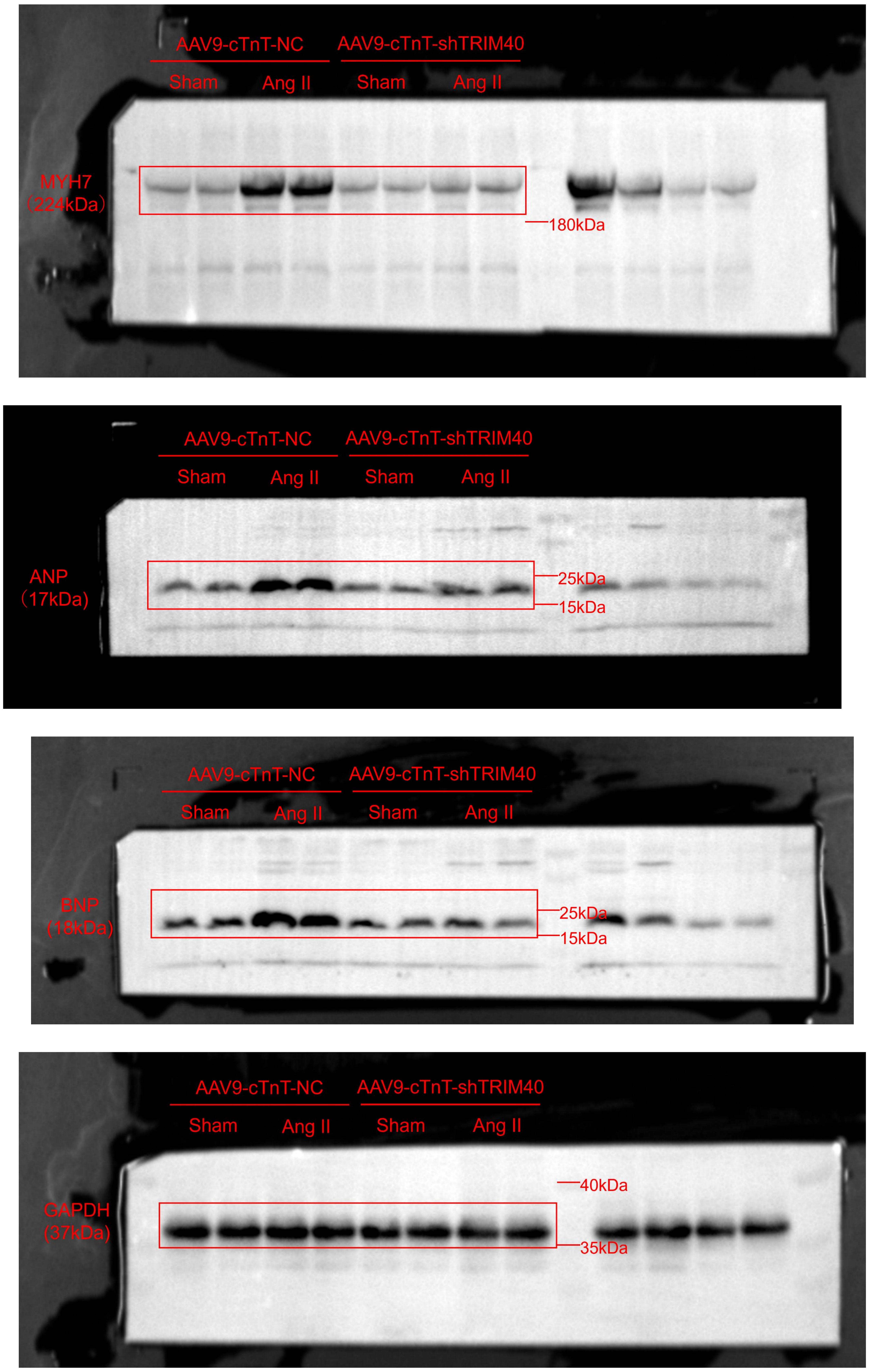
AAV9-cTnT-NC
AAV9-cTnT-shTRIM40
Sham
Ang II
Sham
Ang II
MYH7
（224kDa）
180kDa
AAV9-cTnT-NC
AAV9-cTnT-shTRIM40
Sham
Ang II
Sham
Ang II
ANP
（17kDa）
25kDa
15kDa
AAV9-cTnT-NC
AAV9-cTnT-shTRIM40
Sham
Ang II
Sham
Ang II
BNP
(18kDa)
25kDa
15kDa
AAV9-cTnT-NC
AAV9-cTnT-shTRIM40
Sham
Ang II
Sham
Ang II
GAPDH
(37kDa)
40kDa
35kDa

Figure 5C

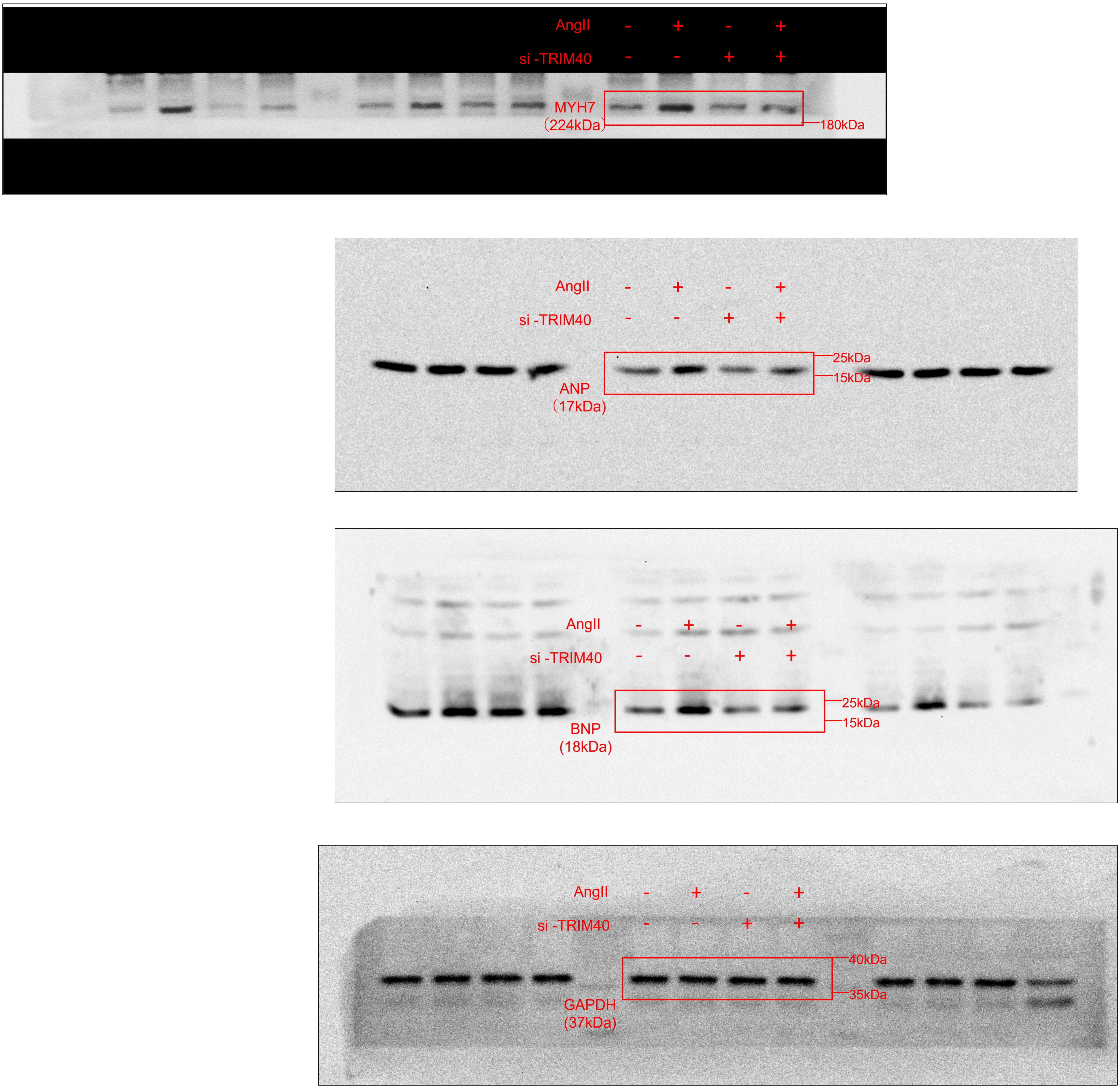
AngII - + - +
si -TRIM40 - - + +
MYH7
(224kDa)
180kDa
AngII - + - +
si -TRIM40 - - + +
25kDa
15kDa
ANP
(17kDa)
AngII - + - +
si -TRIM40 - - + +
25kDa
15kDa
BNP
(18kDa)
AngII - + - +
si -TRIM40 - - + +
40kDa
35kDa
GAPDH
(37kDa)

Figure 5H

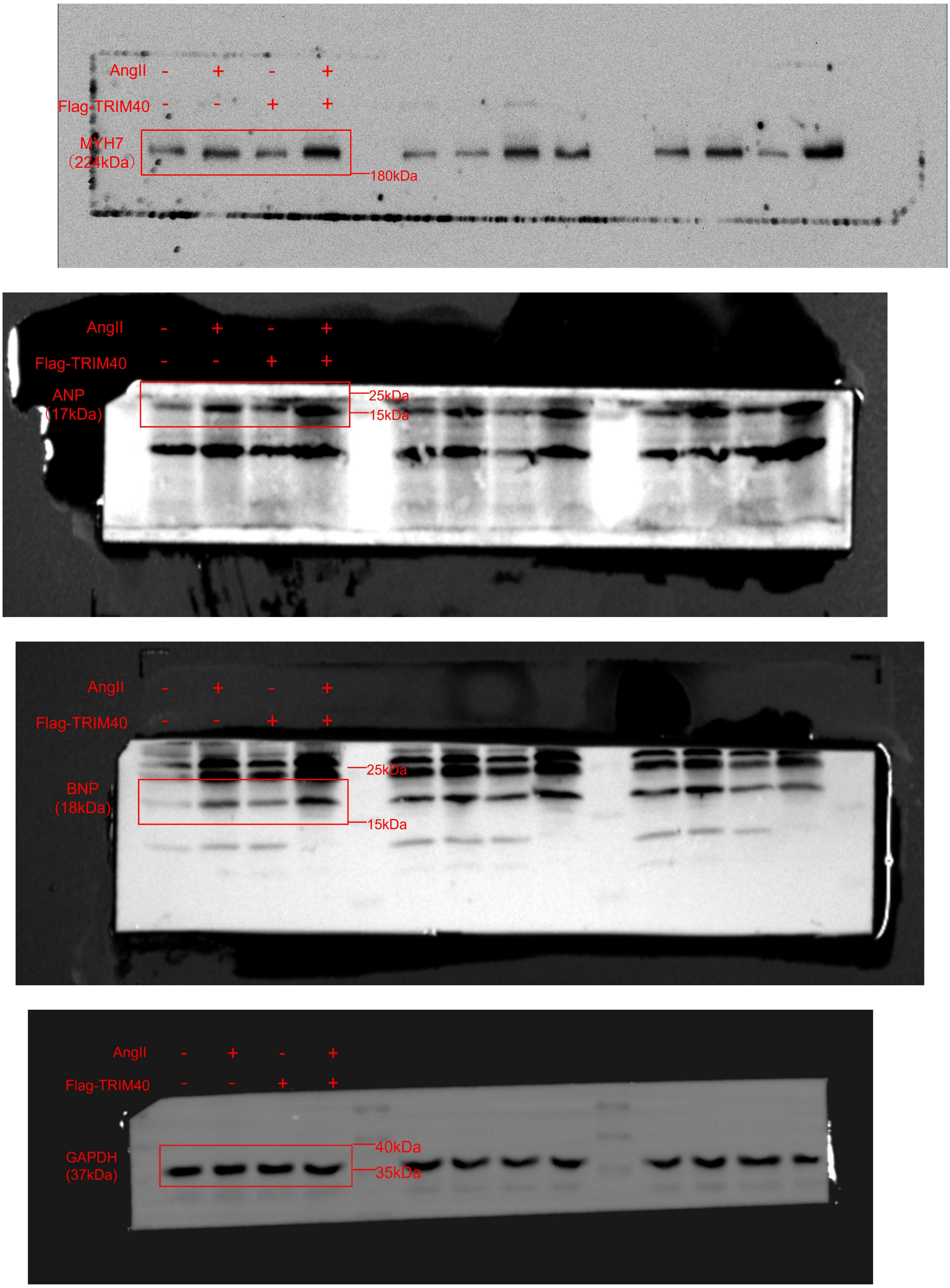
AngII - + - +
Flag-TRIM40 - - + +
MYH7
(224kDa)
180kDa
AngII - + - +
Flag-TRIM40 - - + +
ANP
(17kDa)
25kDa
15kDa
AngII - + - +
Flag-TRIM40 - - + +
BNP
(18kDa)
25kDa
15kDa
AngII - + - +
Flag-TRIM40 - - + +
GAPDH
(37kDa)
40kDa
35kDa

Figure 6D

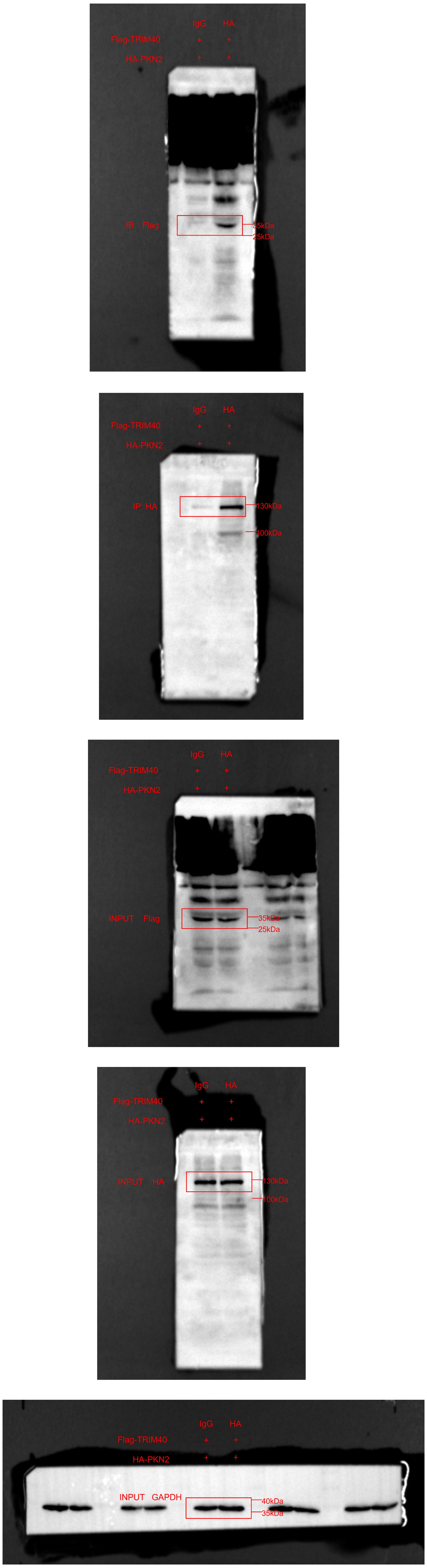

Figure 6E

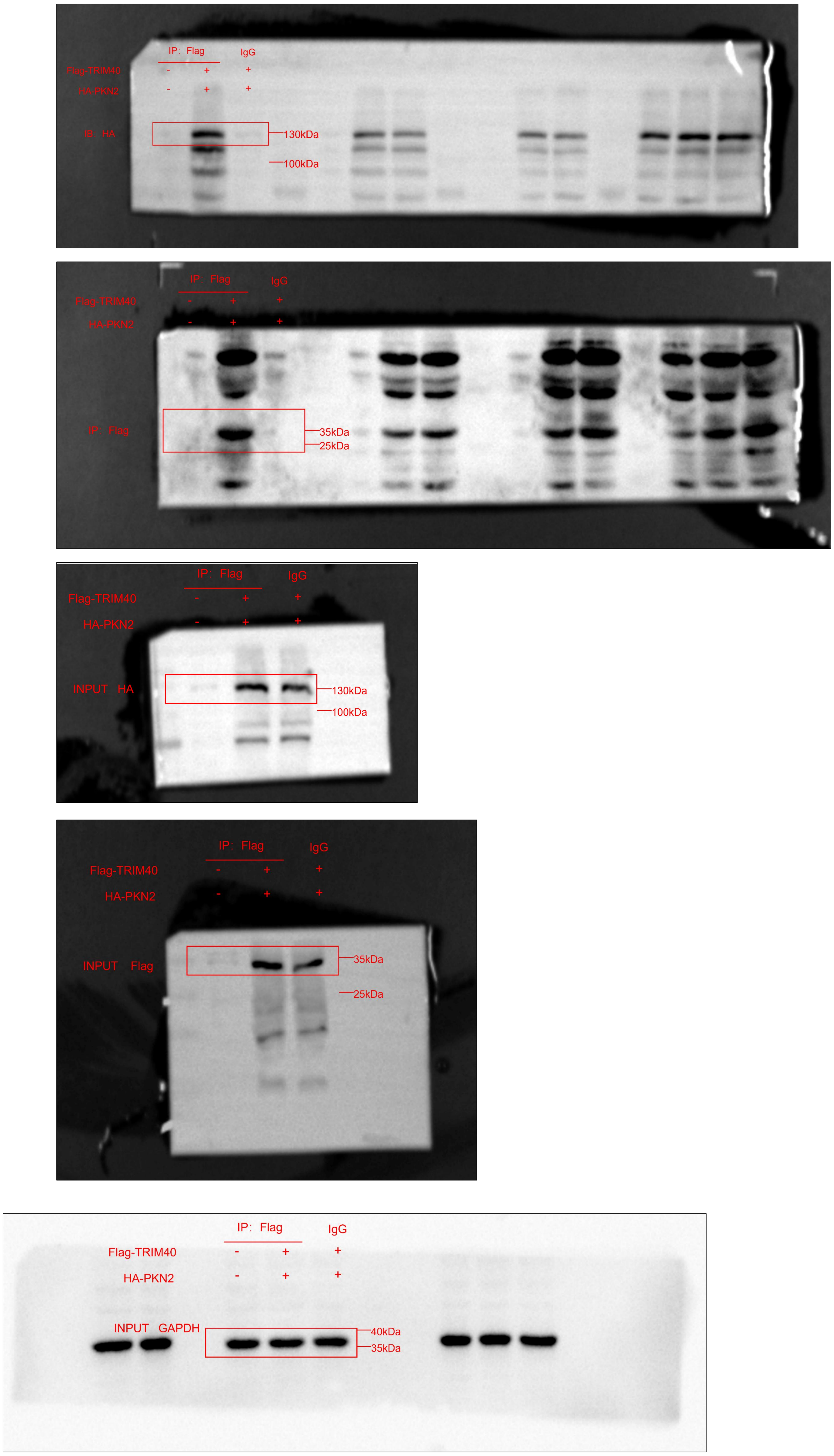

Figure 6F

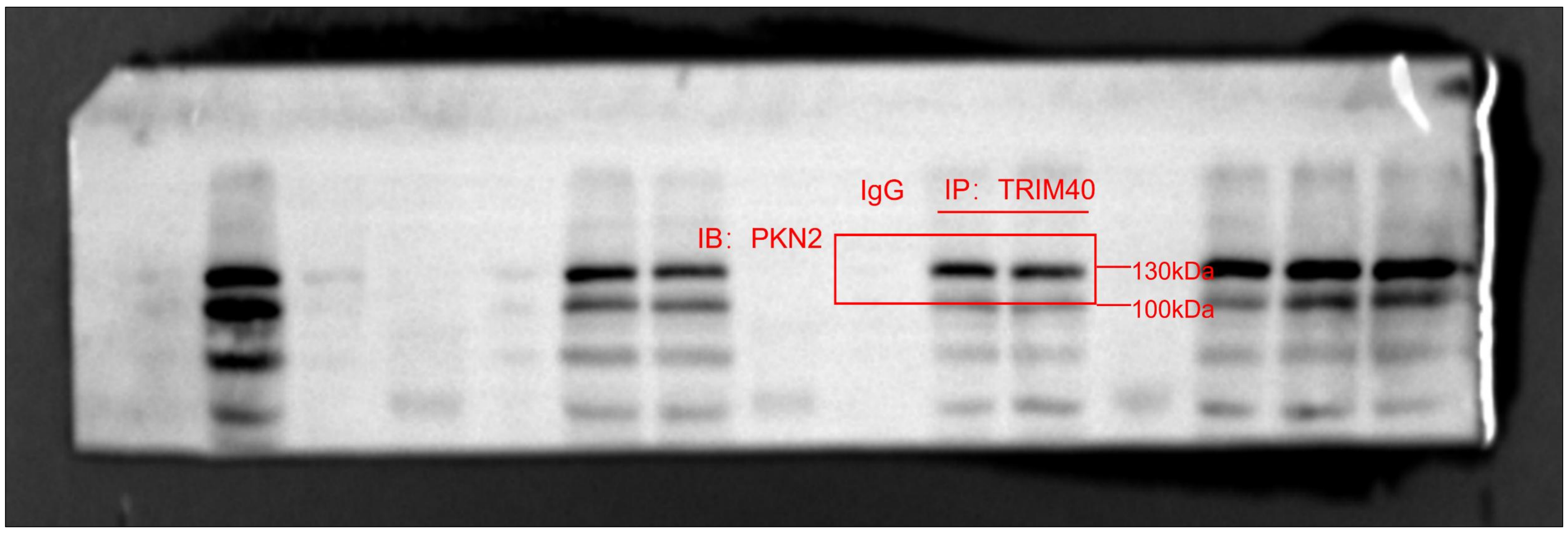
IgG
IP: TRIM40
IB: PKN2
130kDa
100kDa

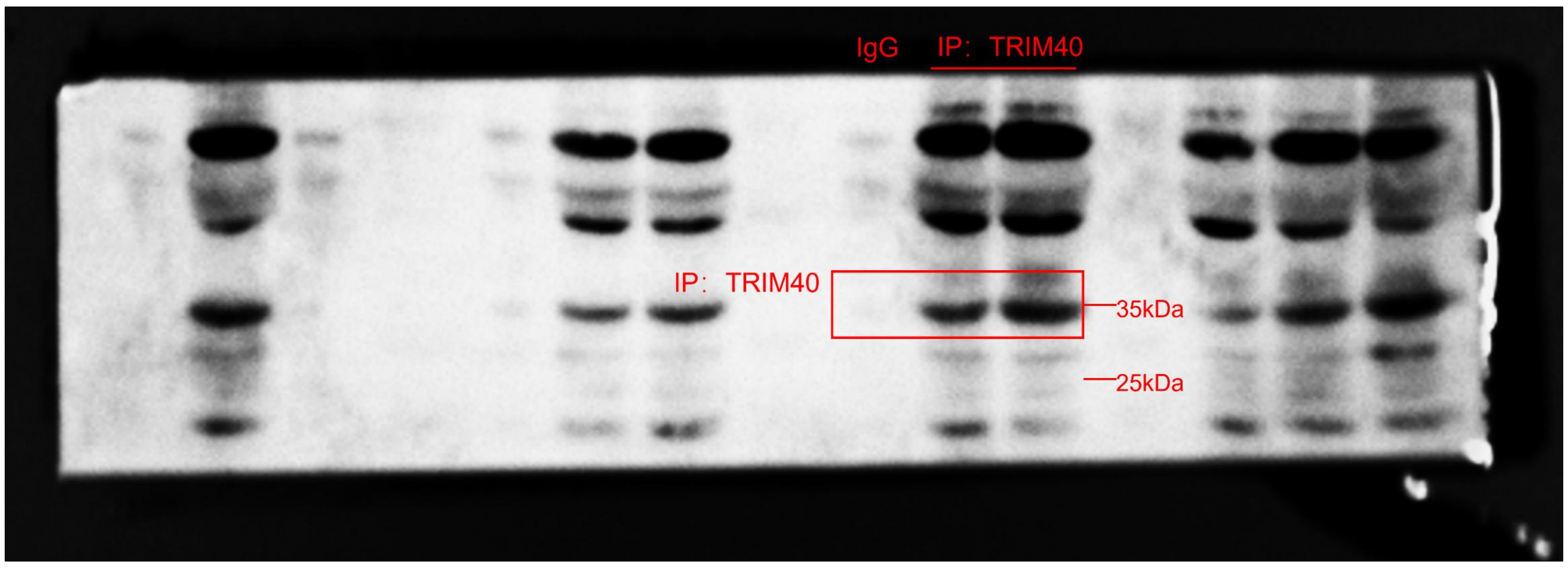
IgG
IP: TRIM40
IP: TRIM40
35kDa
25kDa

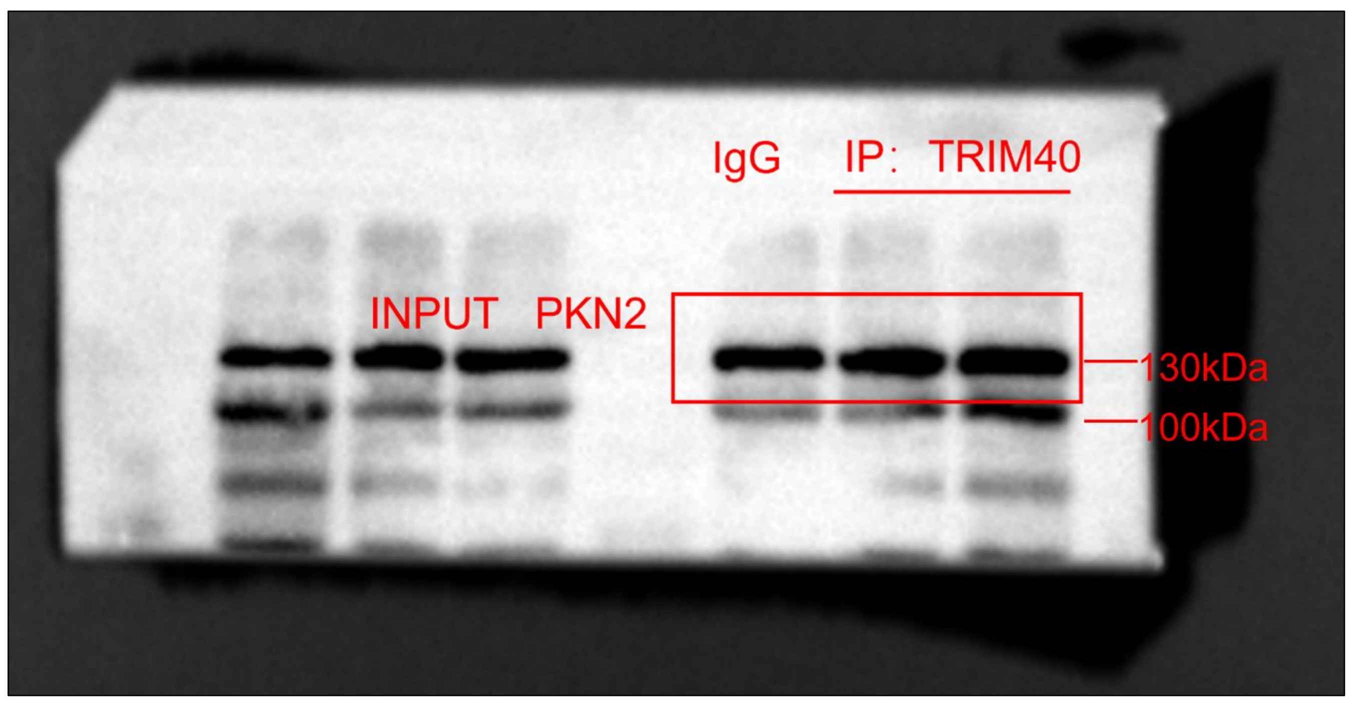
IgG
IP: TRIM40
INPUT PKN2
130kDa
100kDa

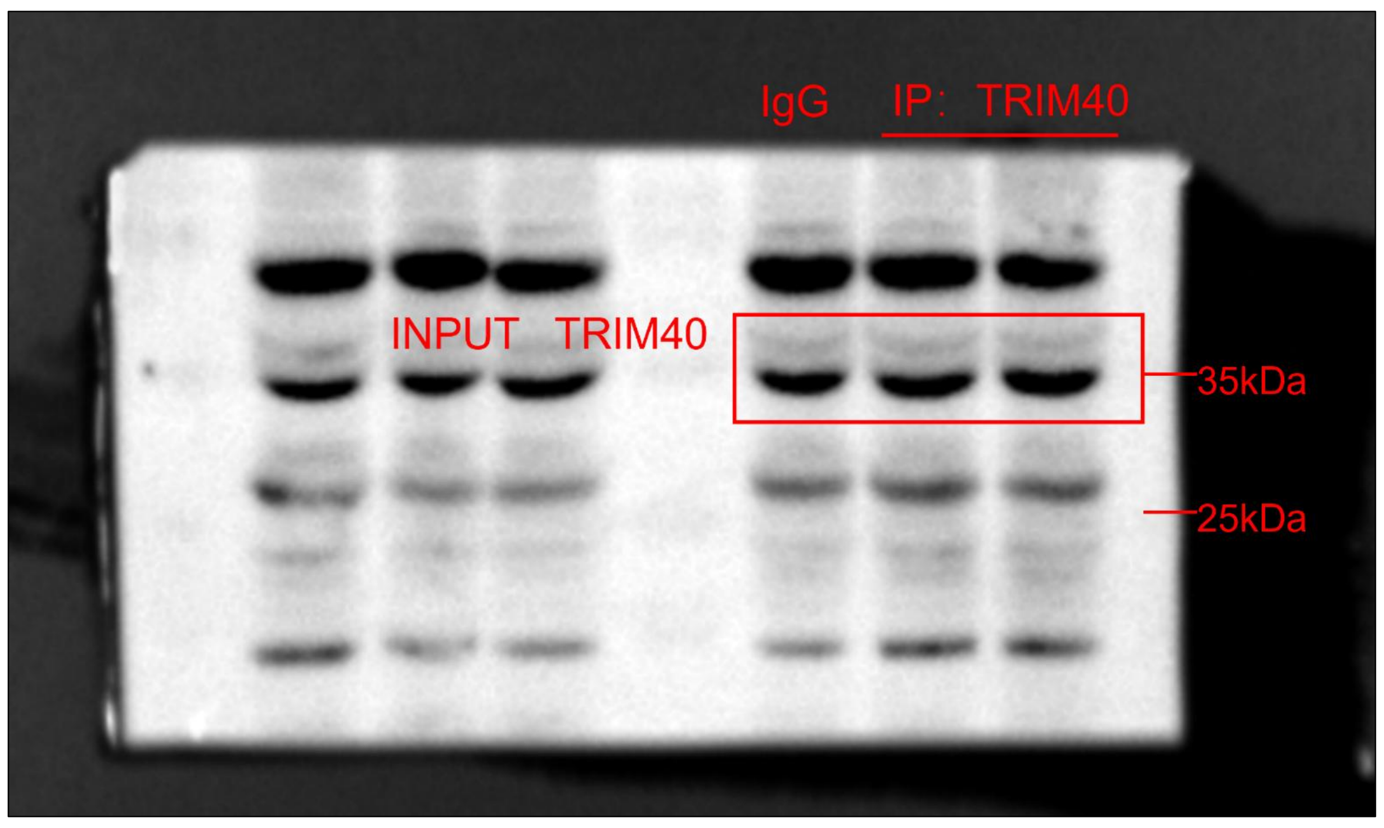
IgG
IP: TRIM40
INPUT TRIM40
35kDa
25kDa

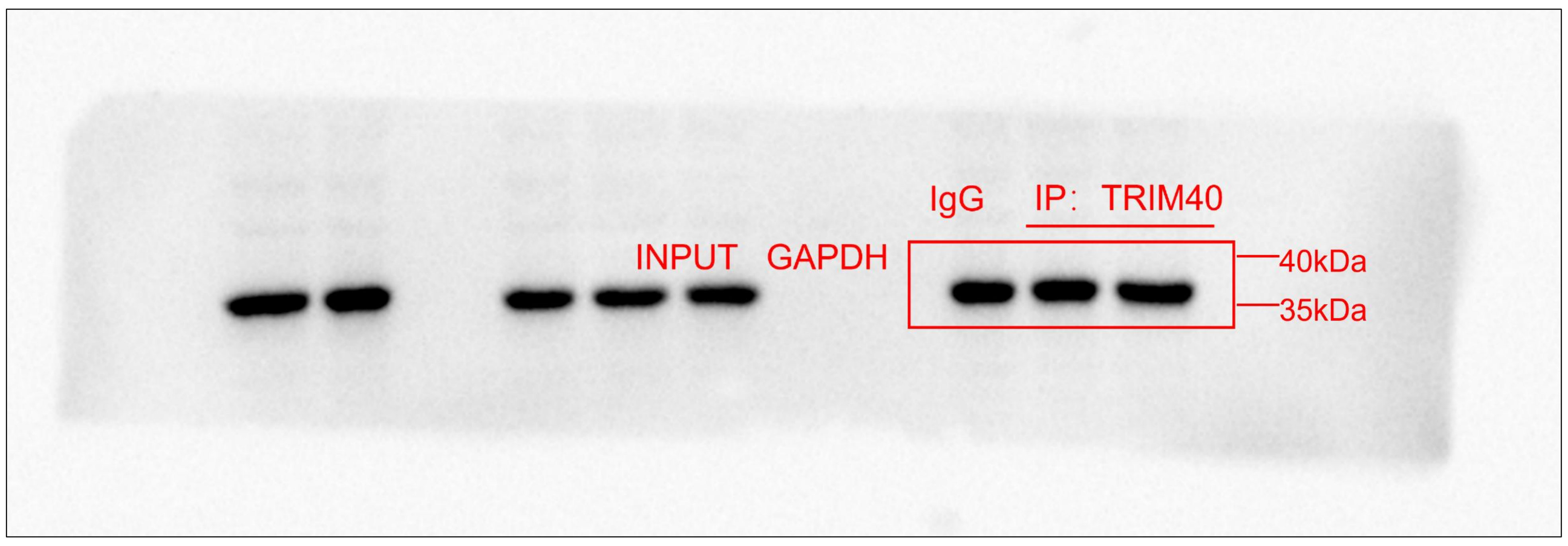
IgG
IP: TRIM40
INPUT GAPDH
40kDa
35kDa

Figure 6H

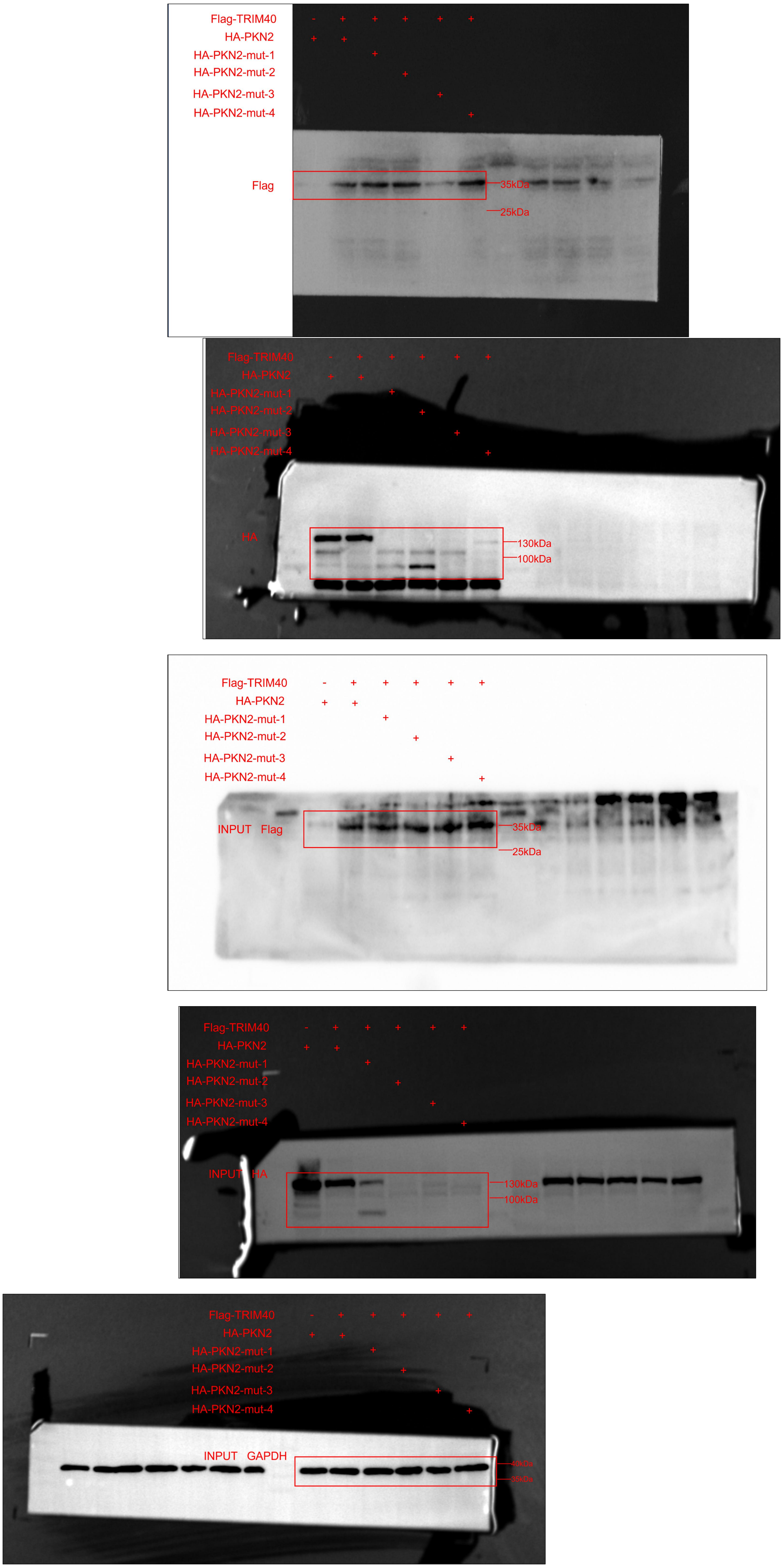
Flag-TRIM40 - + + + + +
HA-PKN2 + +
HA-PKN2-mut-1 +
HA-PKN2-mut-2 +
HA-PKN2-mut-3 +
HA-PKN2-mut-4 +
Flag
35kDa
25kDa
HA
130kDa
100kDa
INPUT Flag
35kDa
25kDa
INPUT HA
130kDa
100kDa
INPUT GAPDH

Figure 6J

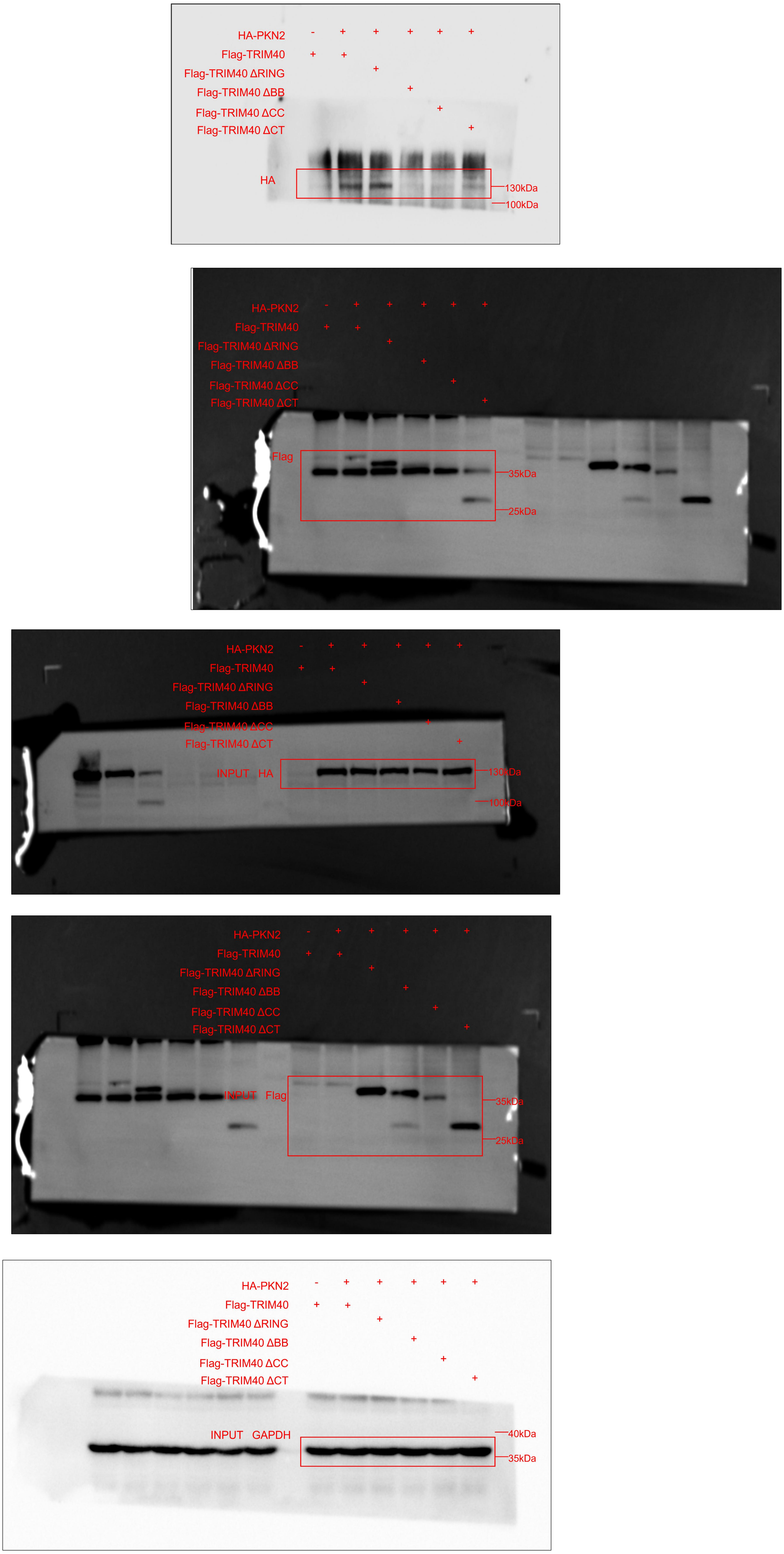
HA-PKN2 - + + + + +
Flag-TRIM40 + +
Flag-TRIM40 ΔRING +
Flag-TRIM40 ΔBB +
Flag-TRIM40 ΔCC +
Flag-TRIM40 ΔCT +
HA
130kDa
100kDa
HA-PKN2 - + + + + +
Flag-TRIM40 + +
Flag-TRIM40 ΔRING +
Flag-TRIM40 ΔBB +
Flag-TRIM40 ΔCC +
Flag-TRIM40 ΔCT +
Flag
35kDa
25kDa
HA-PKN2 - + + + + +
Flag-TRIM40 + +
Flag-TRIM40 ΔRING +
Flag-TRIM40 ΔBB +
Flag-TRIM40 ΔCC +
Flag-TRIM40 ΔCT +
INPUT HA
130kDa
100kDa
HA-PKN2 - + + + + +
Flag-TRIM40 + +
Flag-TRIM40 ΔRING +
Flag-TRIM40 ΔBB +
Flag-TRIM40 ΔCC +
Flag-TRIM40 ΔCT +
INPUT Flag
35kDa
25kDa
HA-PKN2 - + + + + +
Flag-TRIM40 + +
Flag-TRIM40 ΔRING +
Flag-TRIM40 ΔBB +
Flag-TRIM40 ΔCC +
Flag-TRIM40 ΔCT +
INPUT GAPDH
40kDa
35kDa

Figure 6K

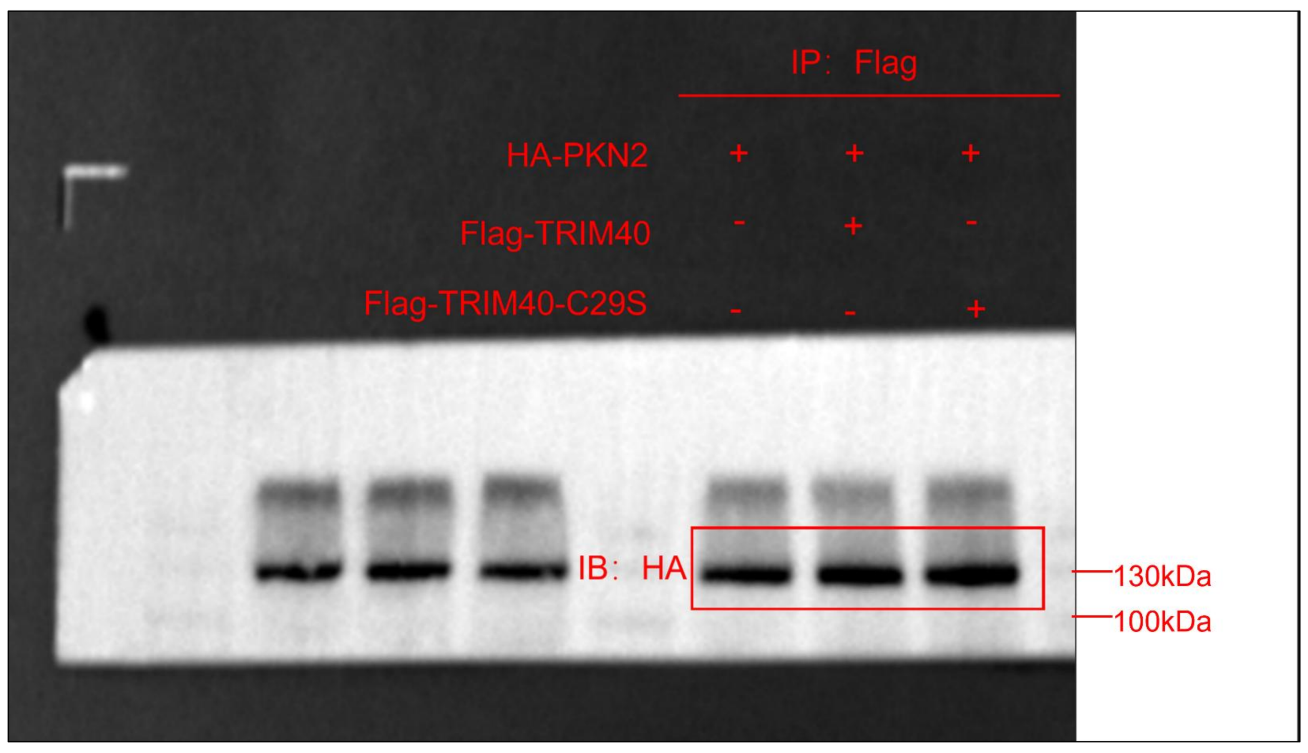
IP: Flag
HA-PKN2 + + +
Flag-TRIM40 - + -
Flag-TRIM40-C29S - - +
IB: HA
130kDa
100kDa

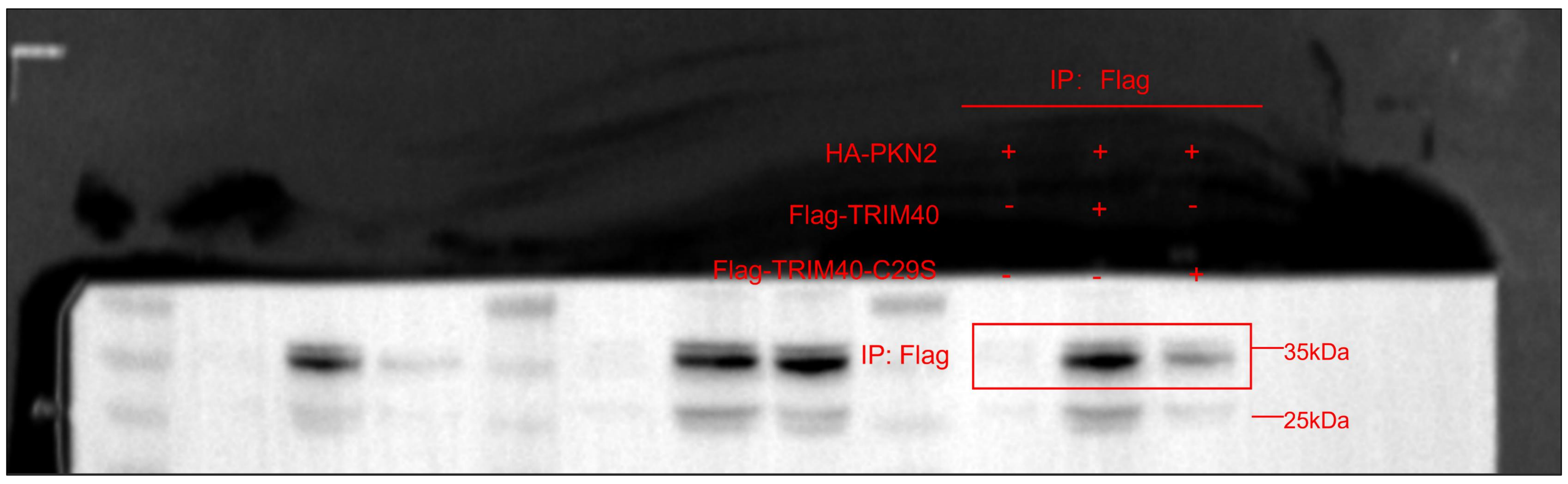
IP: Flag
HA-PKN2 + + +
Flag-TRIM40 - + -
Flag-TRIM40-C29S - - +
IP: Flag
35kDa
25kDa

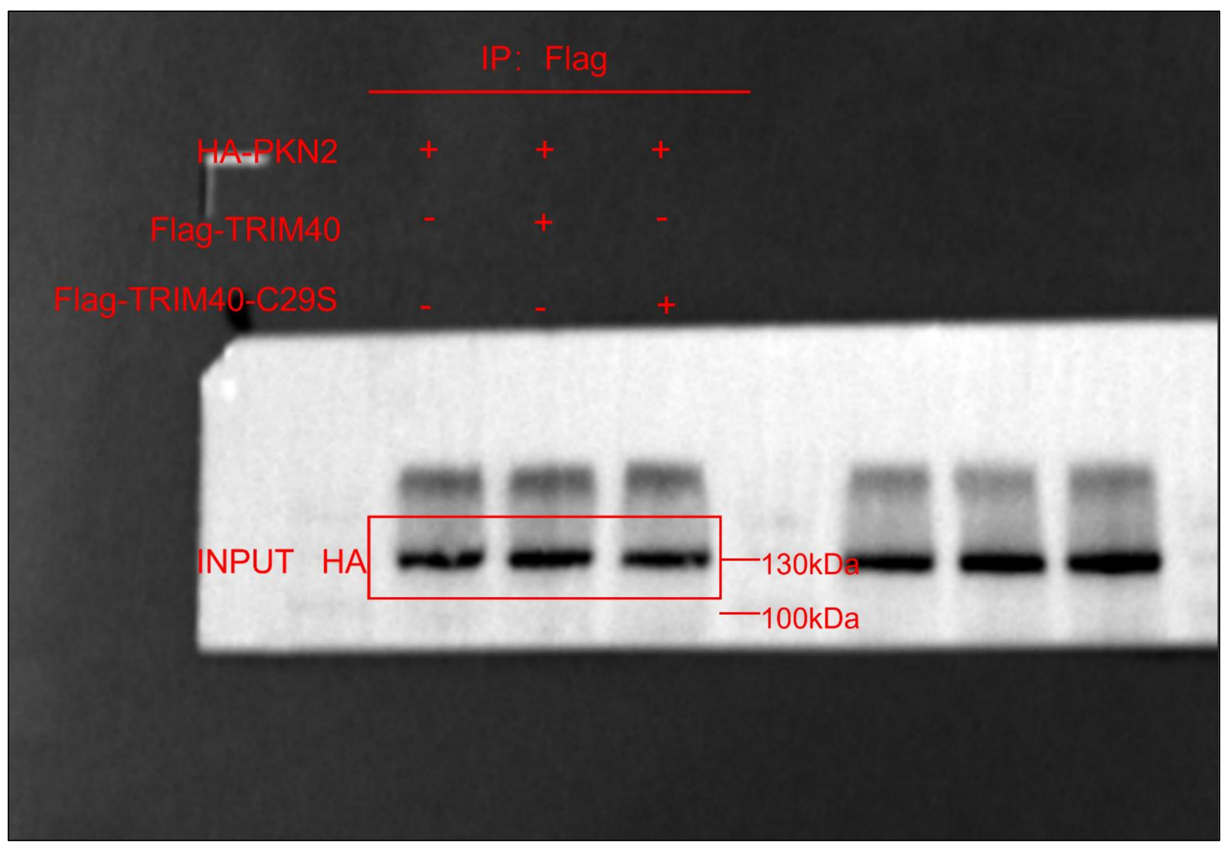
IP: Flag
HA-PKN2 + + +
Flag-TRIM40 - + -
Flag-TRIM40-C29S - - +
INPUT HA
130kDa
100kDa

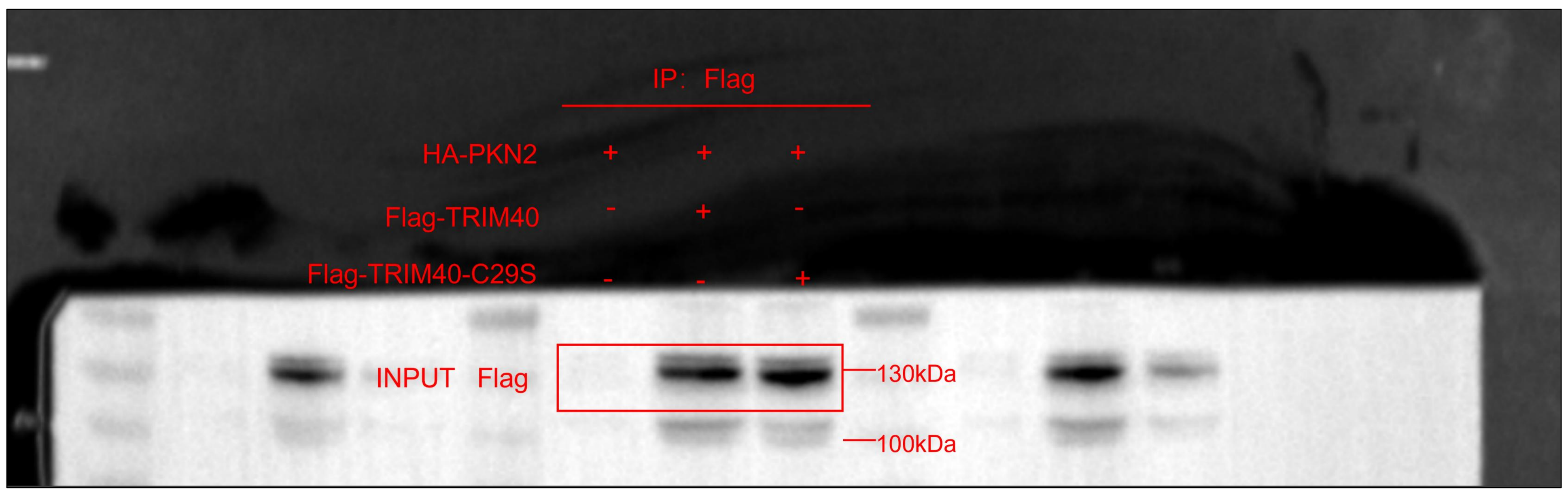
IP: Flag
HA-PKN2 + + +
Flag-TRIM40 - + -
Flag-TRIM40-C29S - - +
INPUT Flag
130kDa
100kDa

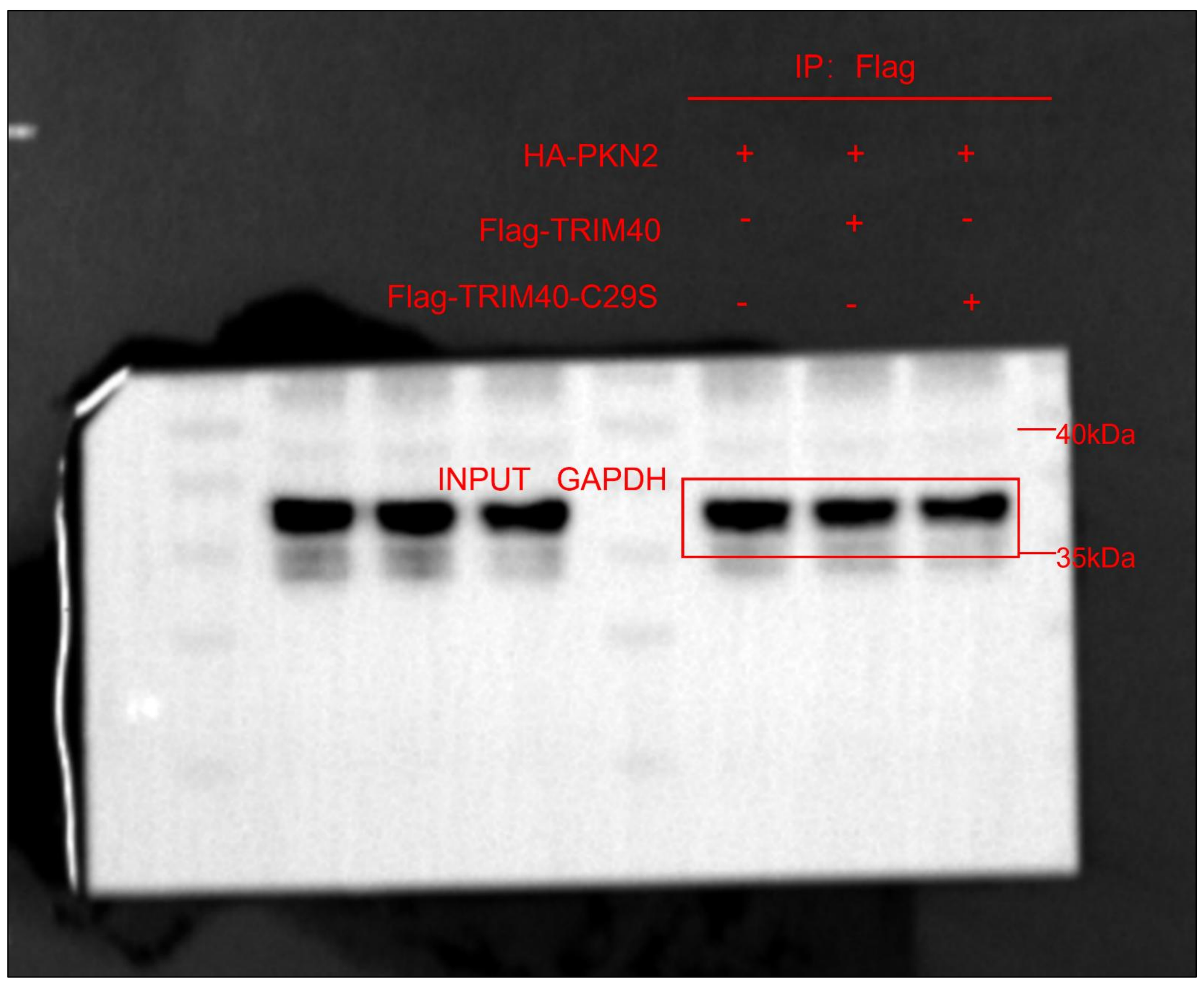
IP: Flag
HA-PKN2 + + +
Flag-TRIM40 - + -
Flag-TRIM40-C29S - - +
INPUT GAPDH
40kDa
35kDa

Figure 7A

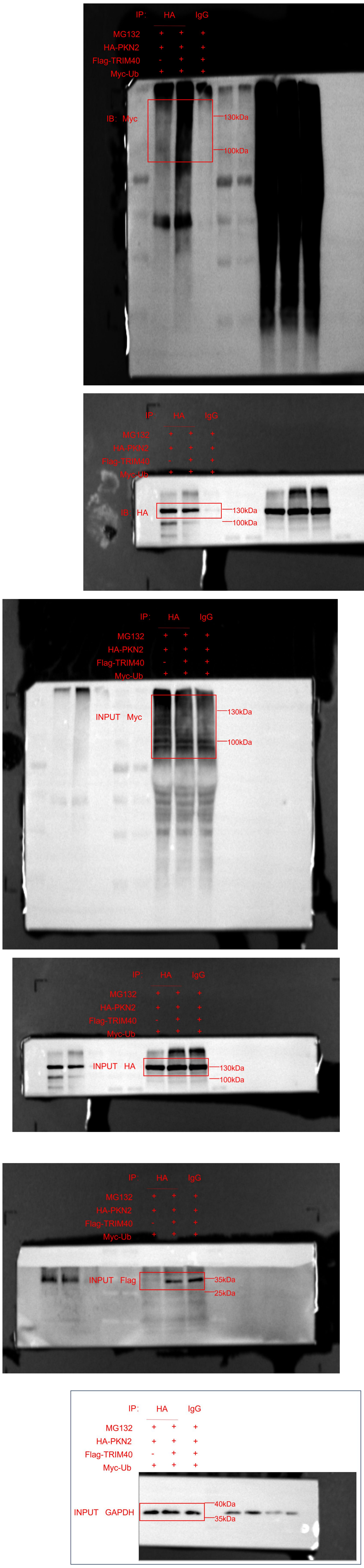
IP: HA IgG
MG132 + + +
HA-PKN2 + + +
Flag-TRIM40 - + +
Myc-Ub + + +
IB: Myc
130kDa
100kDa
IP: HA IgG
MG132 + + +
HA-PKN2 + + +
Flag-TRIM40 - + +
Myc-Ub + + +
IB: HA
130kDa
100kDa
IP: HA IgG
MG132 + + +
HA-PKN2 + + +
Flag-TRIM40 - + +
Myc-Ub + + +
INPUT Myc
130kDa
100kDa
IP: HA IgG
MG132 + + +
HA-PKN2 + + +
Flag-TRIM40 - + +
Myc-Ub + + +
INPUT HA
130kDa
100kDa
IP: HA IgG
MG132 + + +
HA-PKN2 + + +
Flag-TRIM40 - + +
Myc-Ub + + +
INPUT Flag
35kDa
25kDa
IP: HA IgG
MG132 + + +
HA-PKN2 + + +
Flag-TRIM40 - + +
Myc-Ub + + +
INPUT GAPDH
40kDa
35kDa

Figure 7B

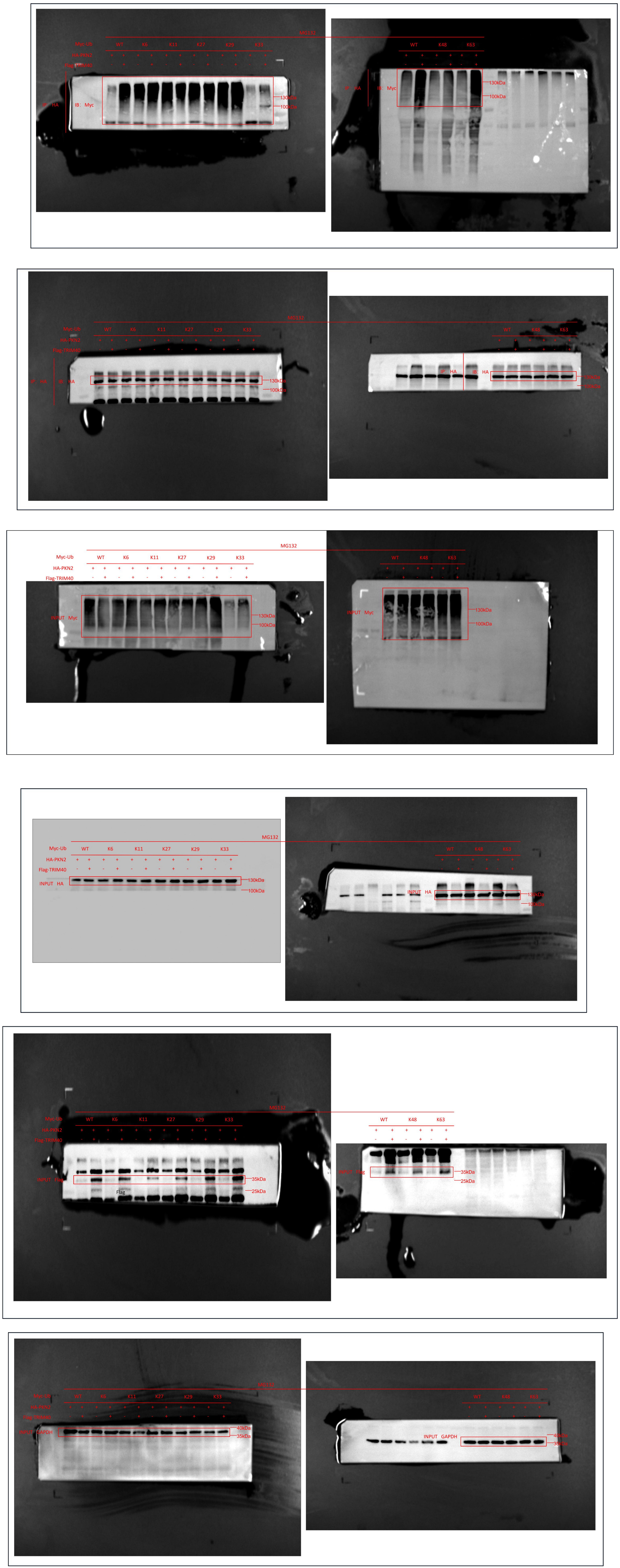
MG132
Myc-Ub
HA-PKN2
Flag-TRIM40
WT
K6
K11
K27
K29
K33
K48
K63
IP: HA
IB: Myc
IB: HA
INPUT Myc
INPUT HA
INPUT Flag
INPUT GAPDH
130kDa
100kDa
35kDa
25kDa
40kDa

Figure 7C

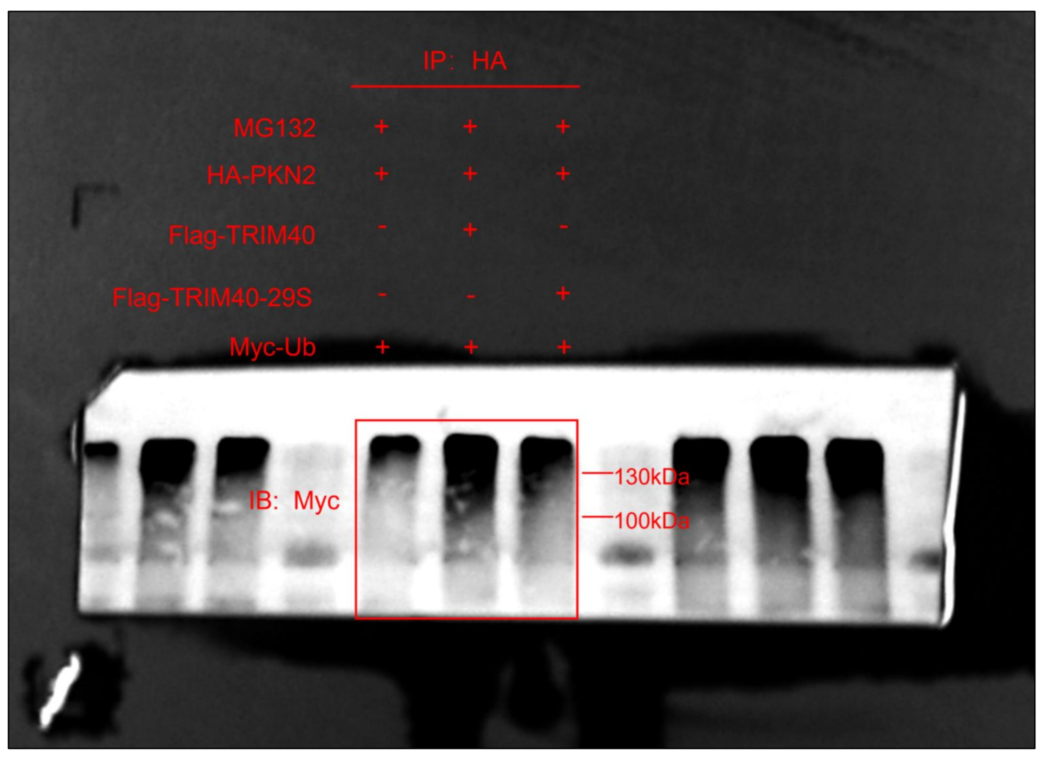

IP: HA
MG132 + + +
HA-PKN2 + + +
Flag-TRIM40 - + -
Flag-TRIM40-29S - - +
Myc-Ub + + +
IB: Myc
130kDa
100kDa

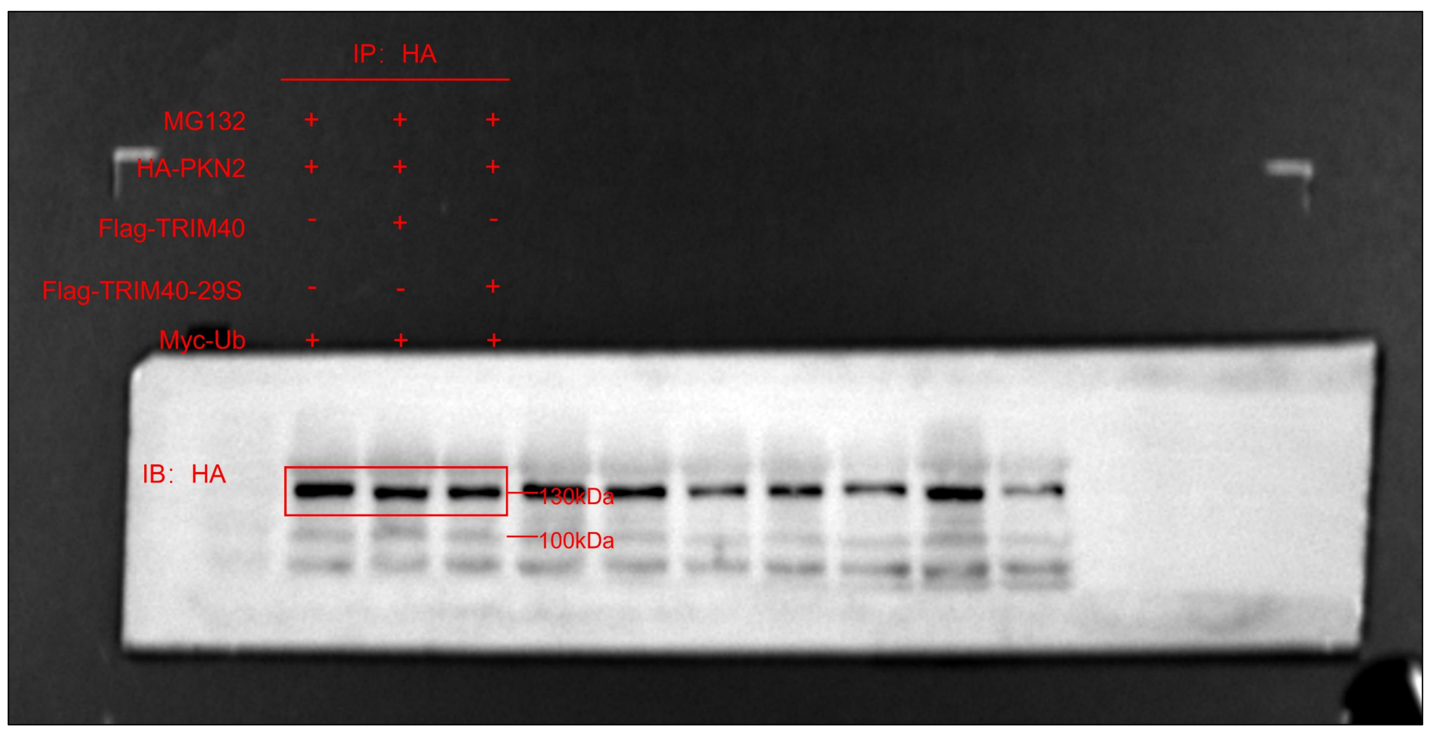

IP: HA
MG132 + + +
HA-PKN2 + + +
Flag-TRIM40 - + -
Flag-TRIM40-29S - - +
Myc-Ub + + +
IB: HA
130kDa
100kDa

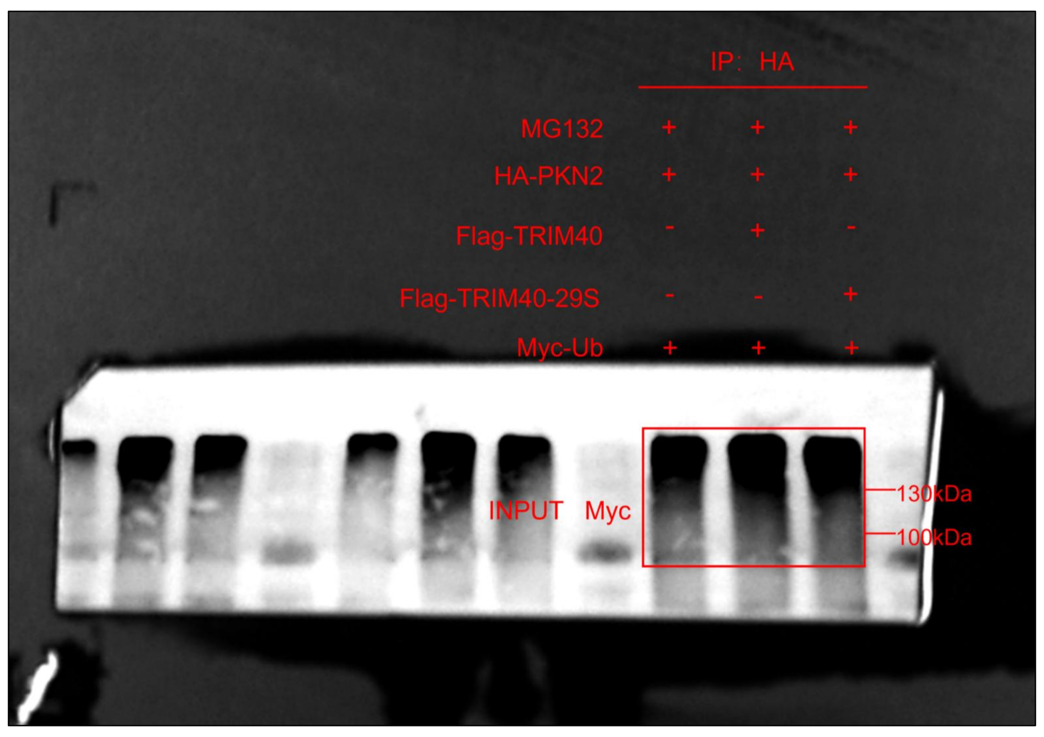

IP: HA
MG132 + + +
HA-PKN2 + + +
Flag-TRIM40 - + -
Flag-TRIM40-29S - - +
Myc-Ub + + +
INPUT Myc
130kDa
100kDa

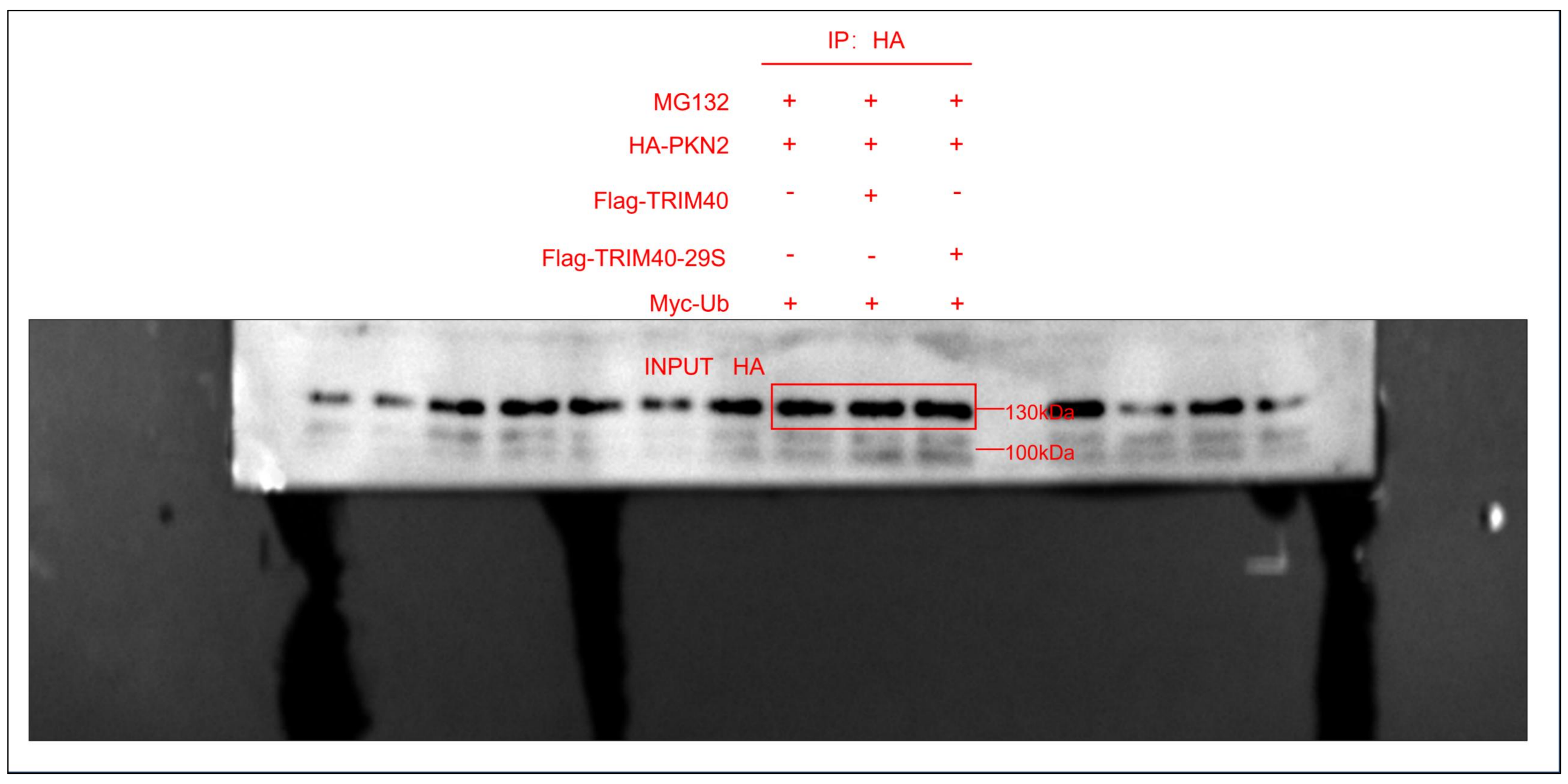

IP: HA
MG132 + + +
HA-PKN2 + + +
Flag-TRIM40 - + -
Flag-TRIM40-29S - - +
Myc-Ub + + +
INPUT HA
130kDa
100kDa

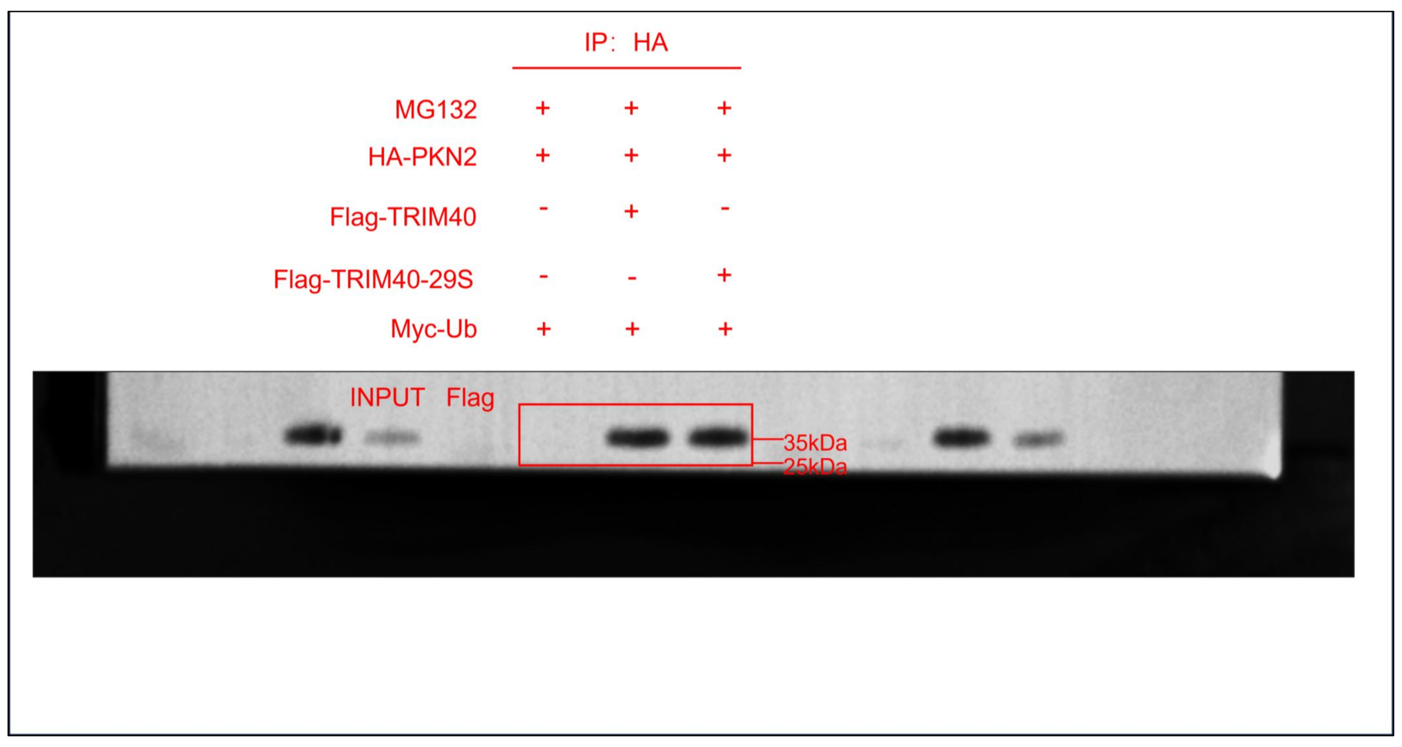

IP: HA
MG132 + + +
HA-PKN2 + + +
Flag-TRIM40 - + -
Flag-TRIM40-29S - - +
Myc-Ub + + +
INPUT Flag
35kDa
25kDa

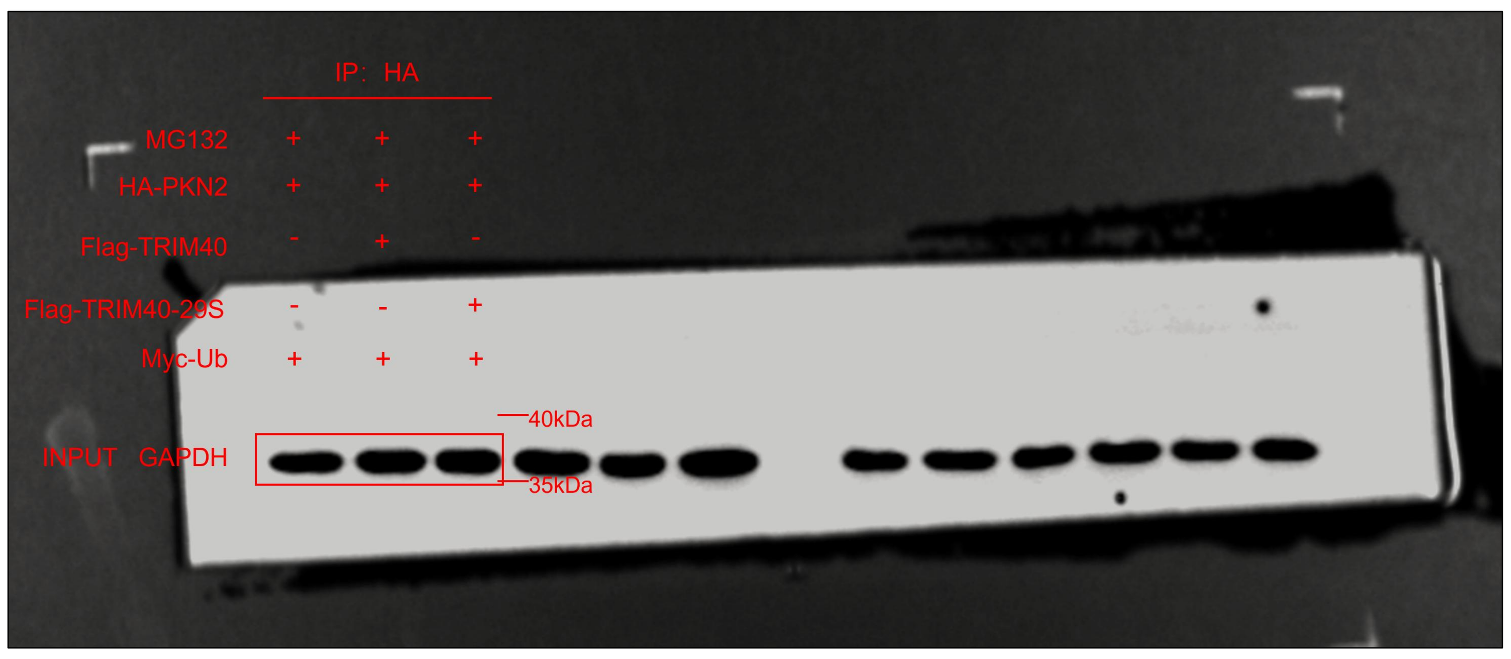

IP: HA
MG132 + + +
HA-PKN2 + + +
Flag-TRIM40 - + -
Flag-TRIM40-29S - - +
Myc-Ub + + +
INPUT GAPDH
40kDa
35kDa

Figure 7D

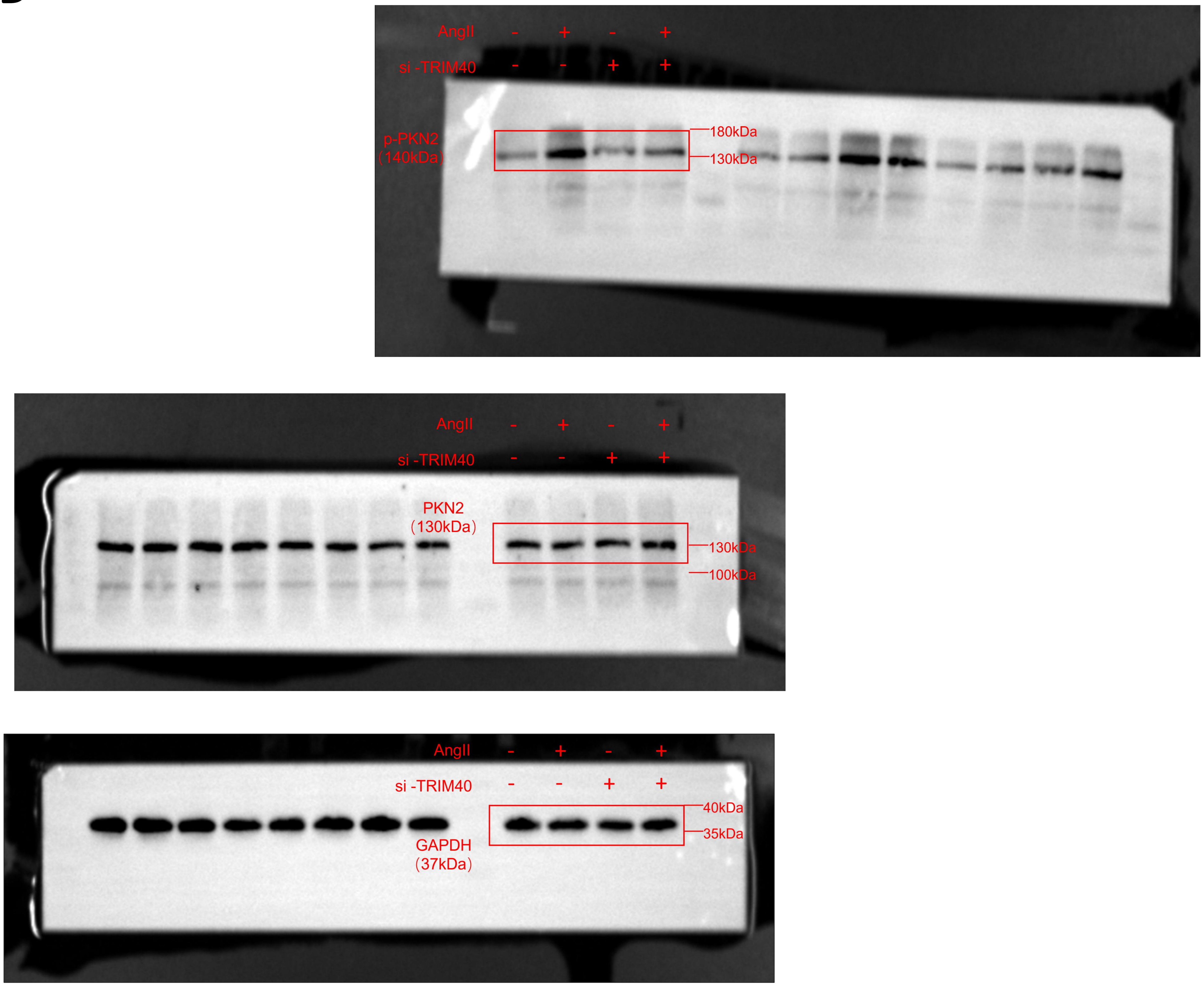

Figure 7F

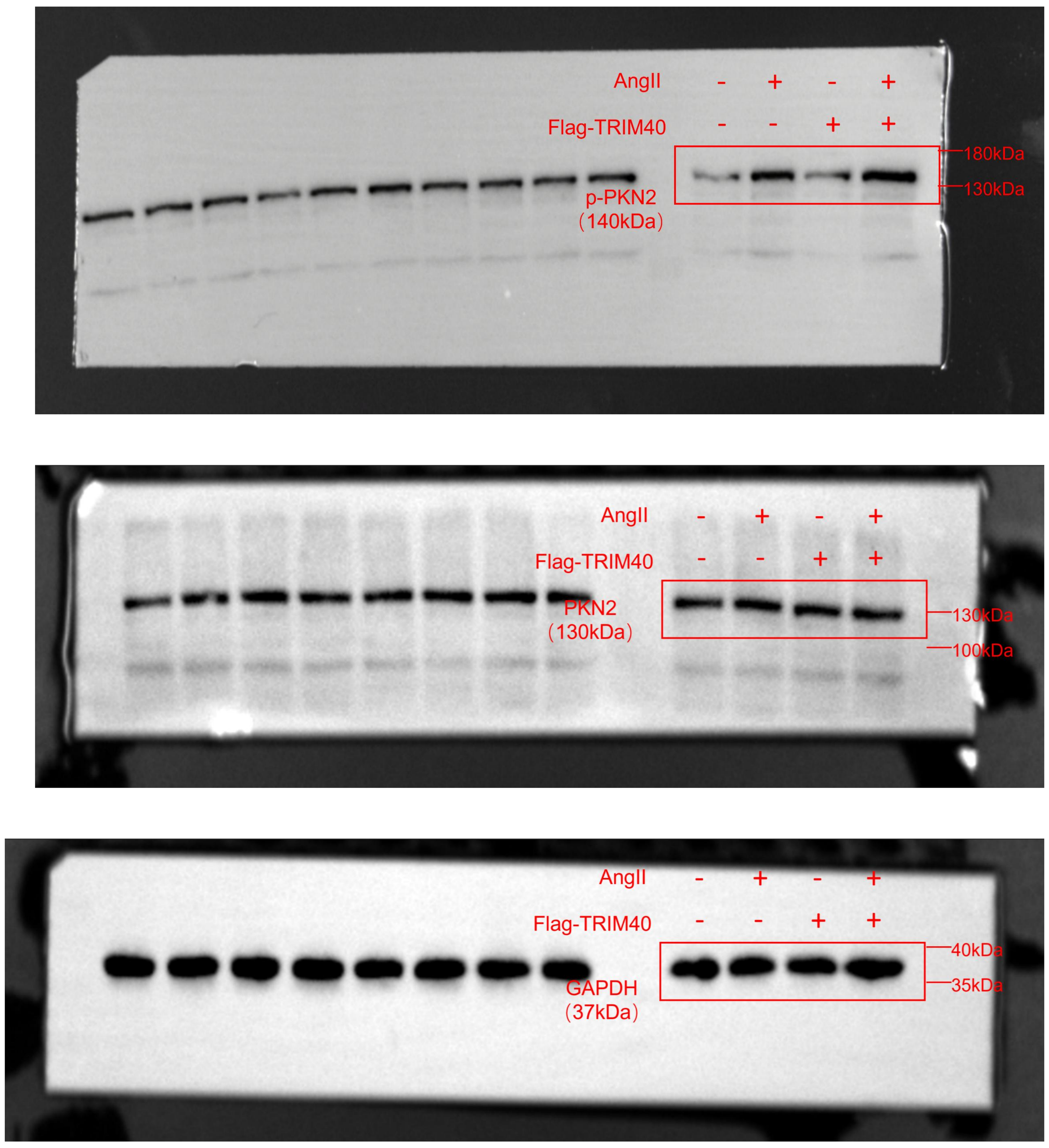

Figure 7H

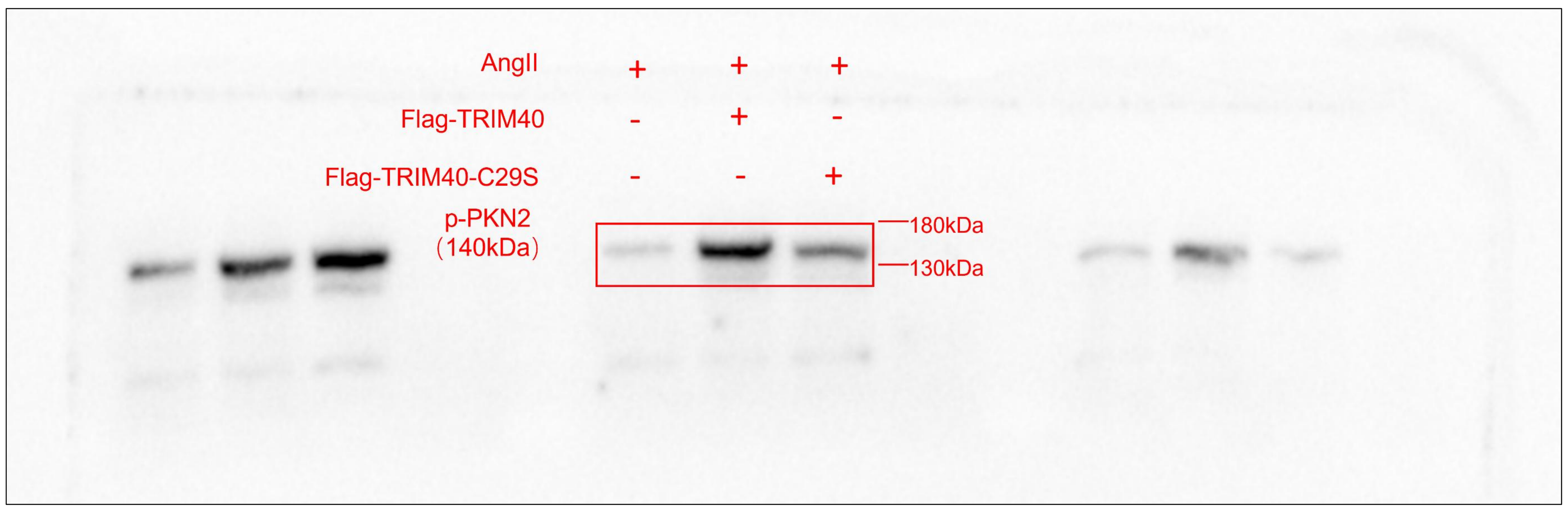

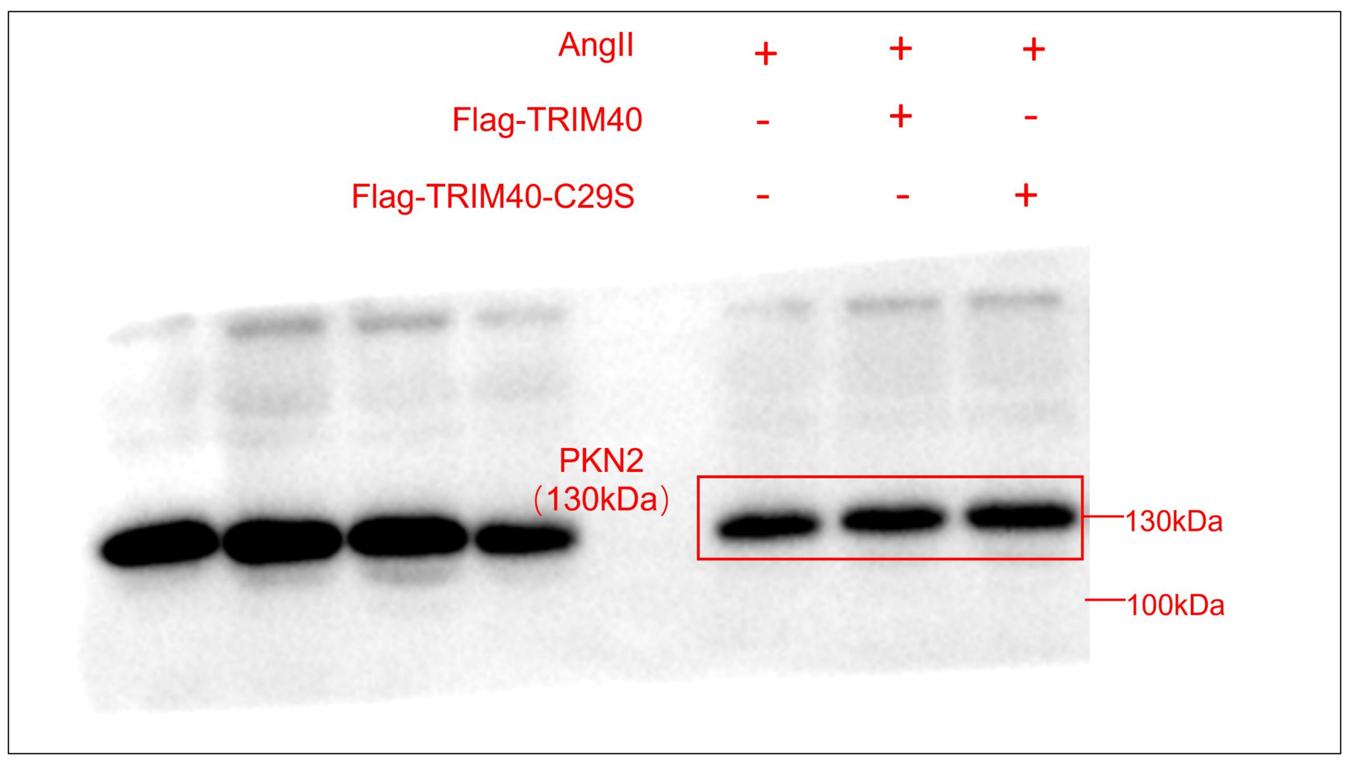

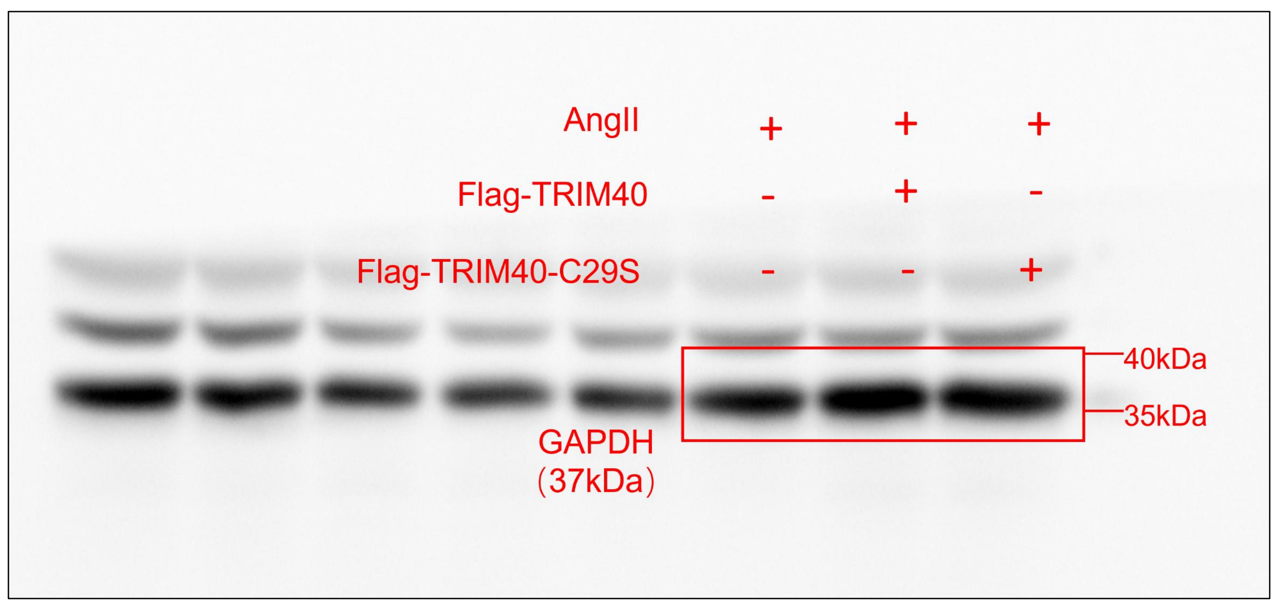

Figure 7J

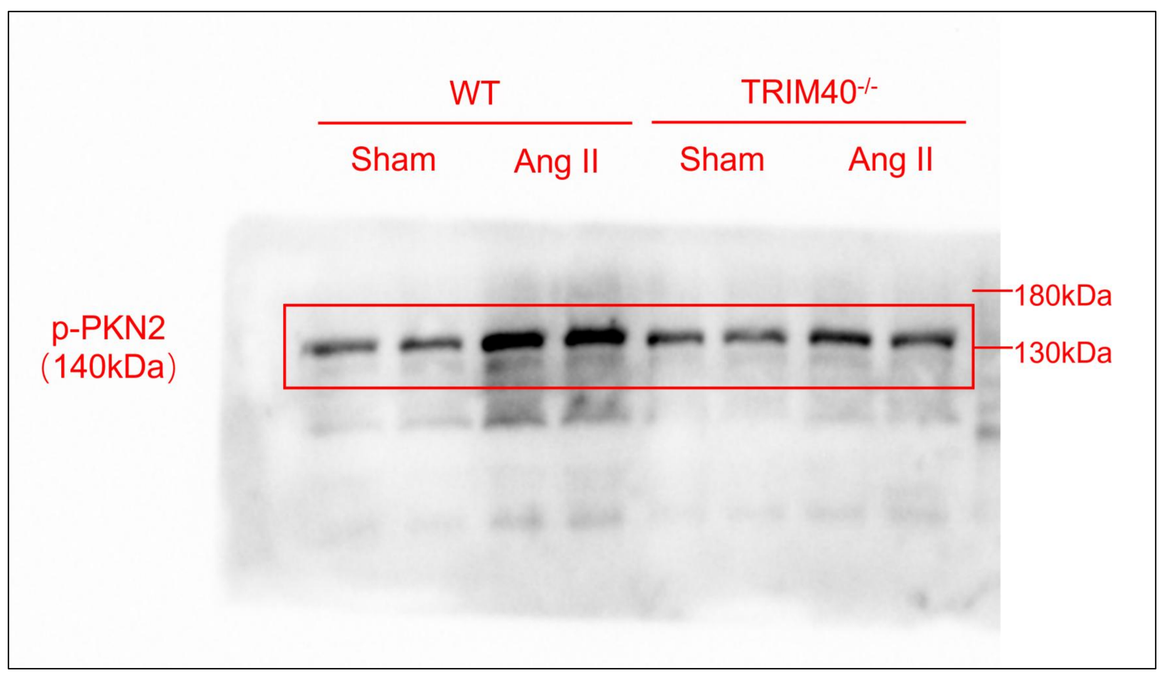

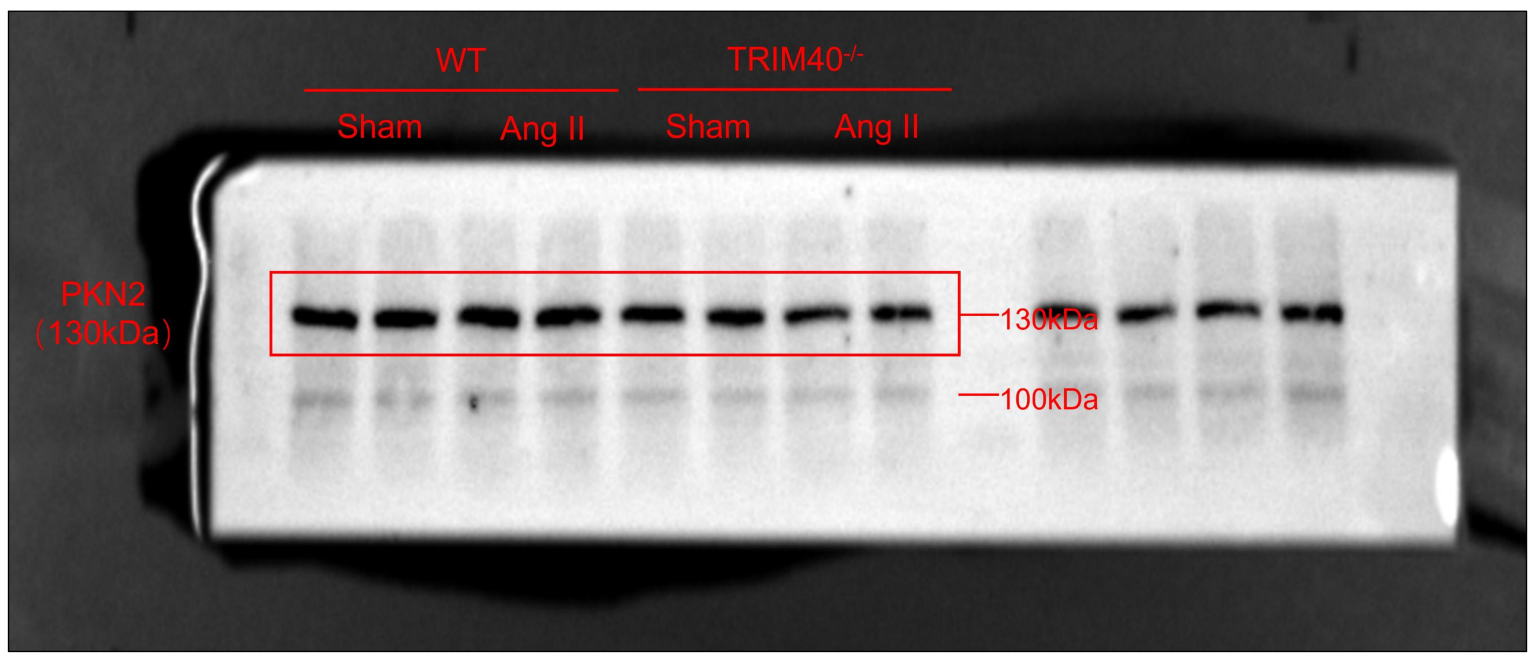

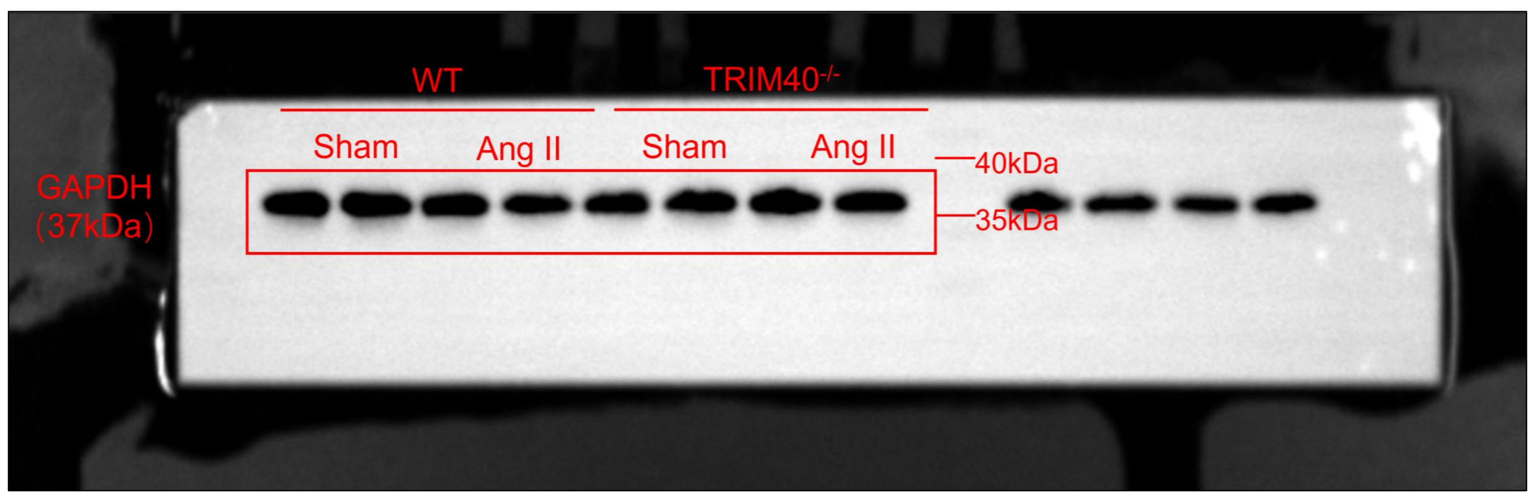

Figure 7L

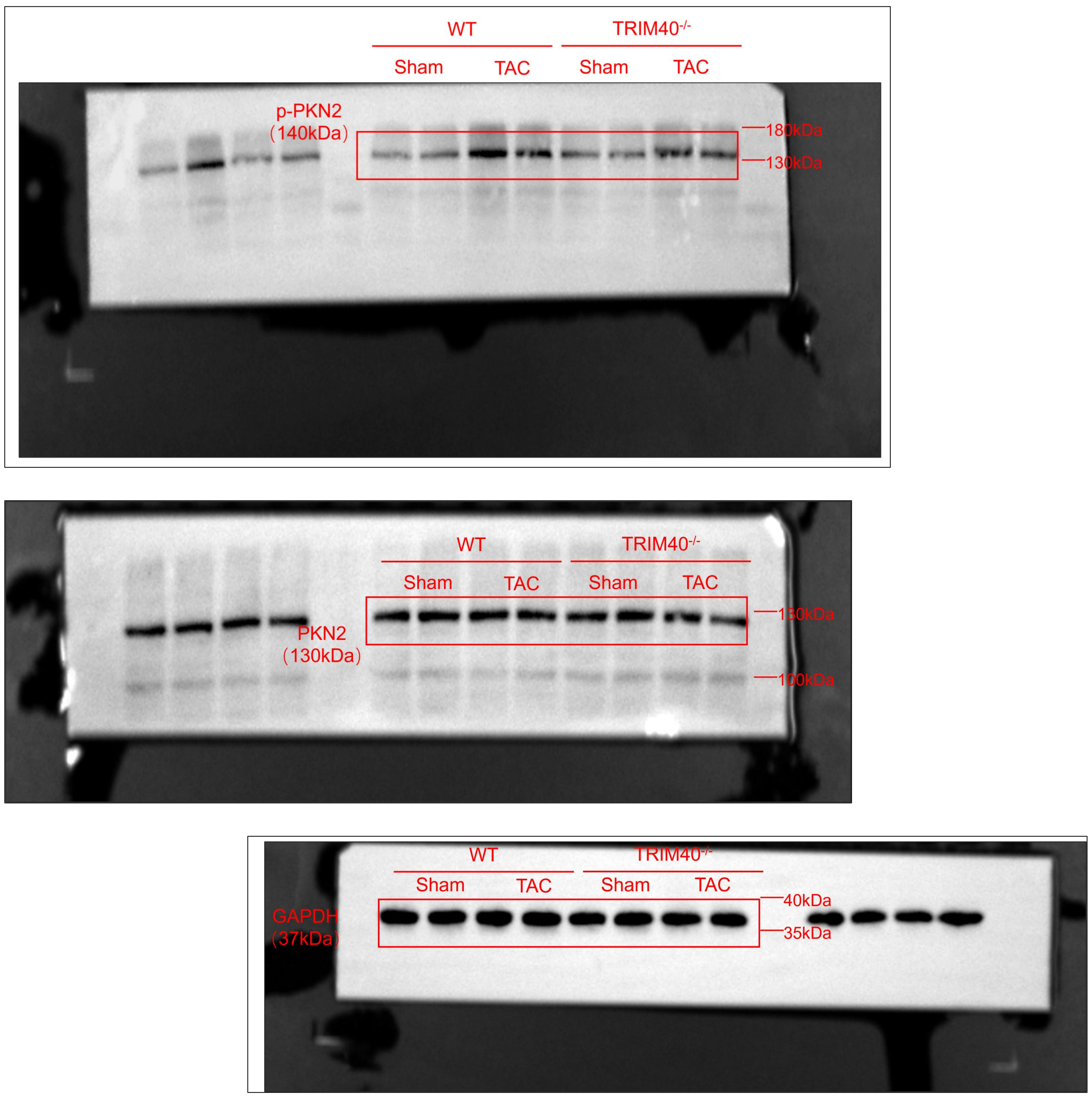

Figure 7N

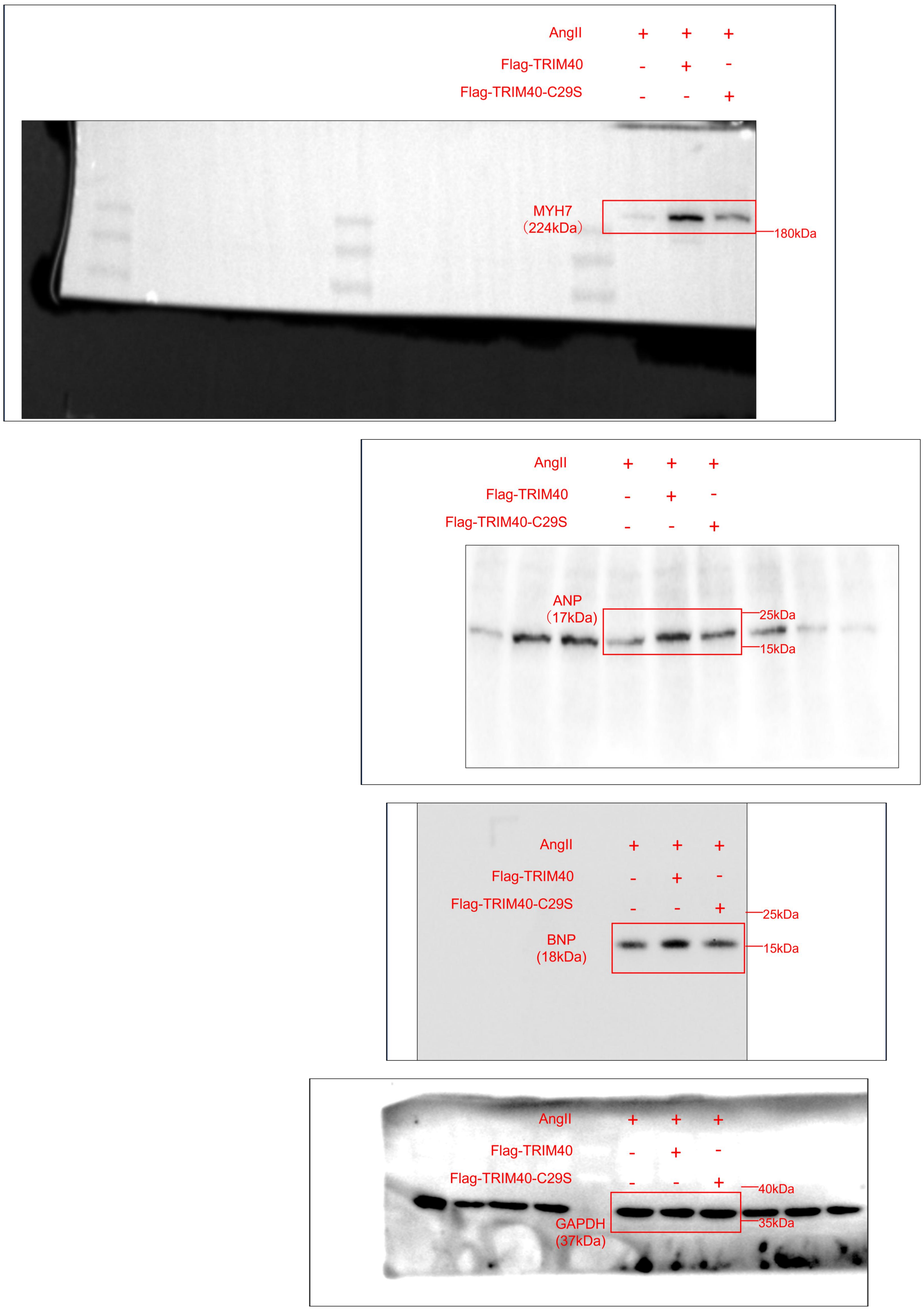

Figure 8N

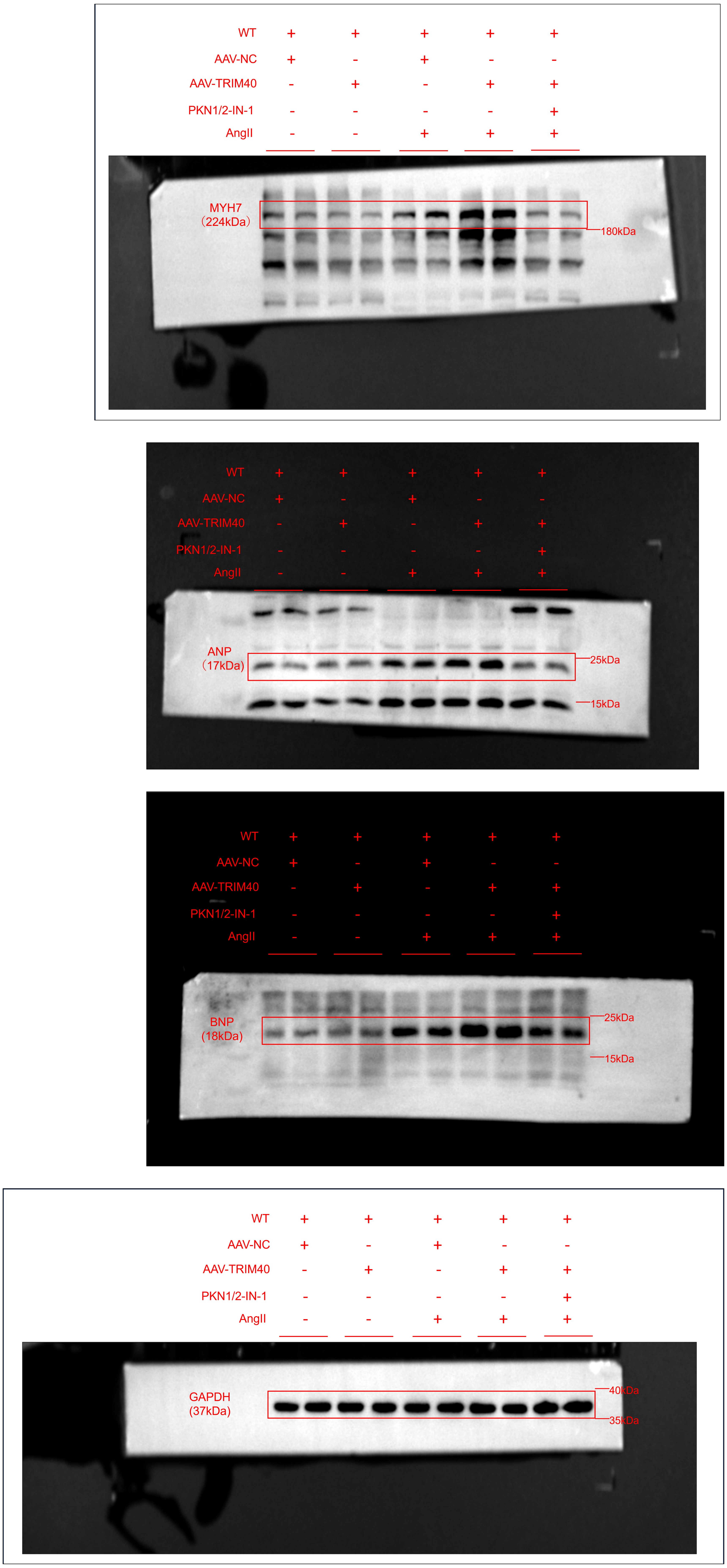

WT + + + + +
AAV-NC + - + - -
AAV-TRIM40 - + - + +
PKN1/2-IN-1 - - - - +
AngII - - + + +
MYH7 (224kDa)
180kDa
ANP (17kDa)
25kDa
15kDa
BNP (18kDa)
25kDa
15kDa
GAPDH (37kDa)
40kDa
35kDa

Figure 8N

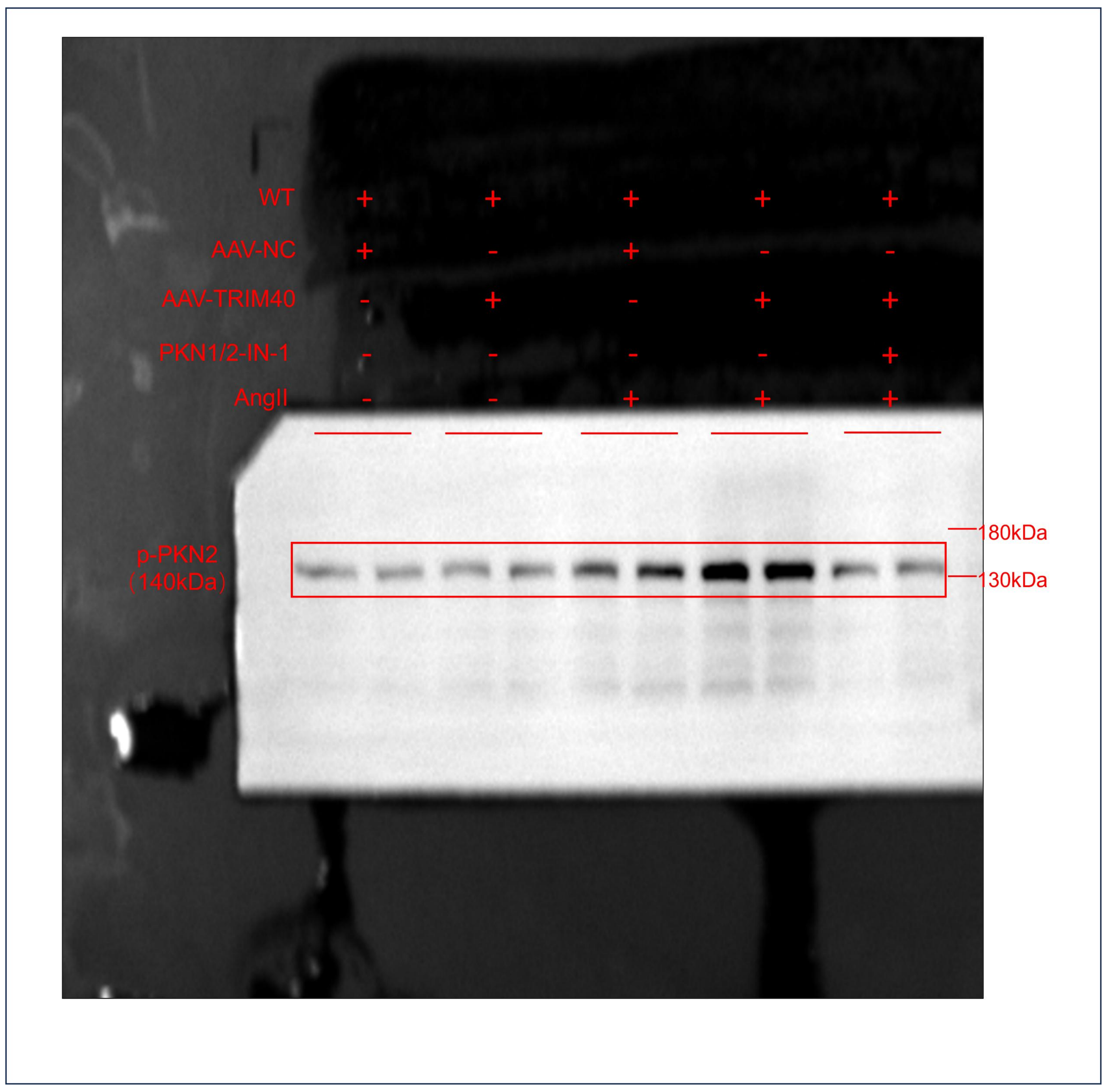
WT
AAV-NC
AAV-TRIM40
PKN1/2-IN-1
AngII
p-PKN2
(140kDa)
180kDa
130kDa

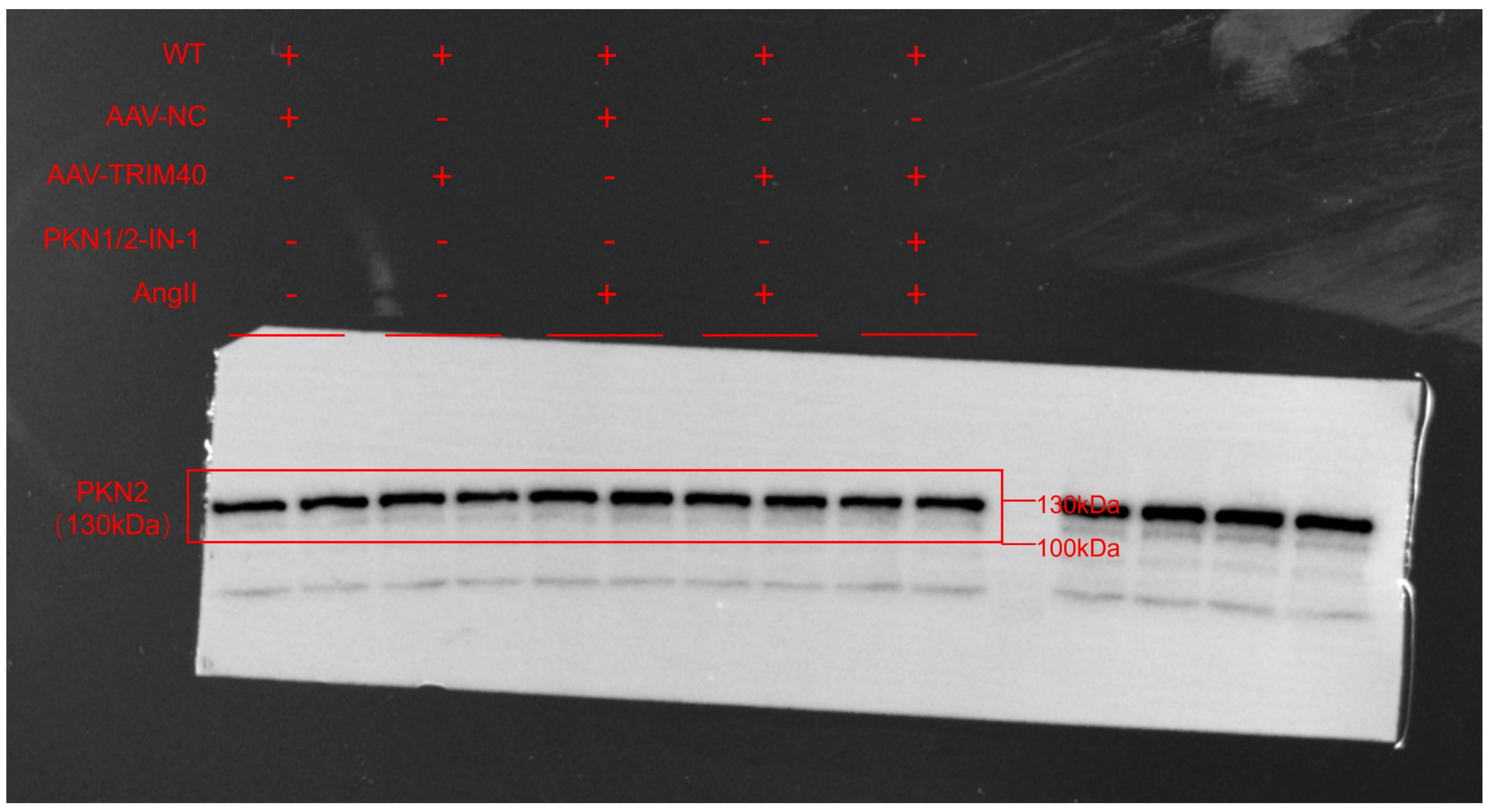
WT
AAV-NC
AAV-TRIM40
PKN1/2-IN-1
AngII
PKN2
(130kDa)
130kDa
100kDa

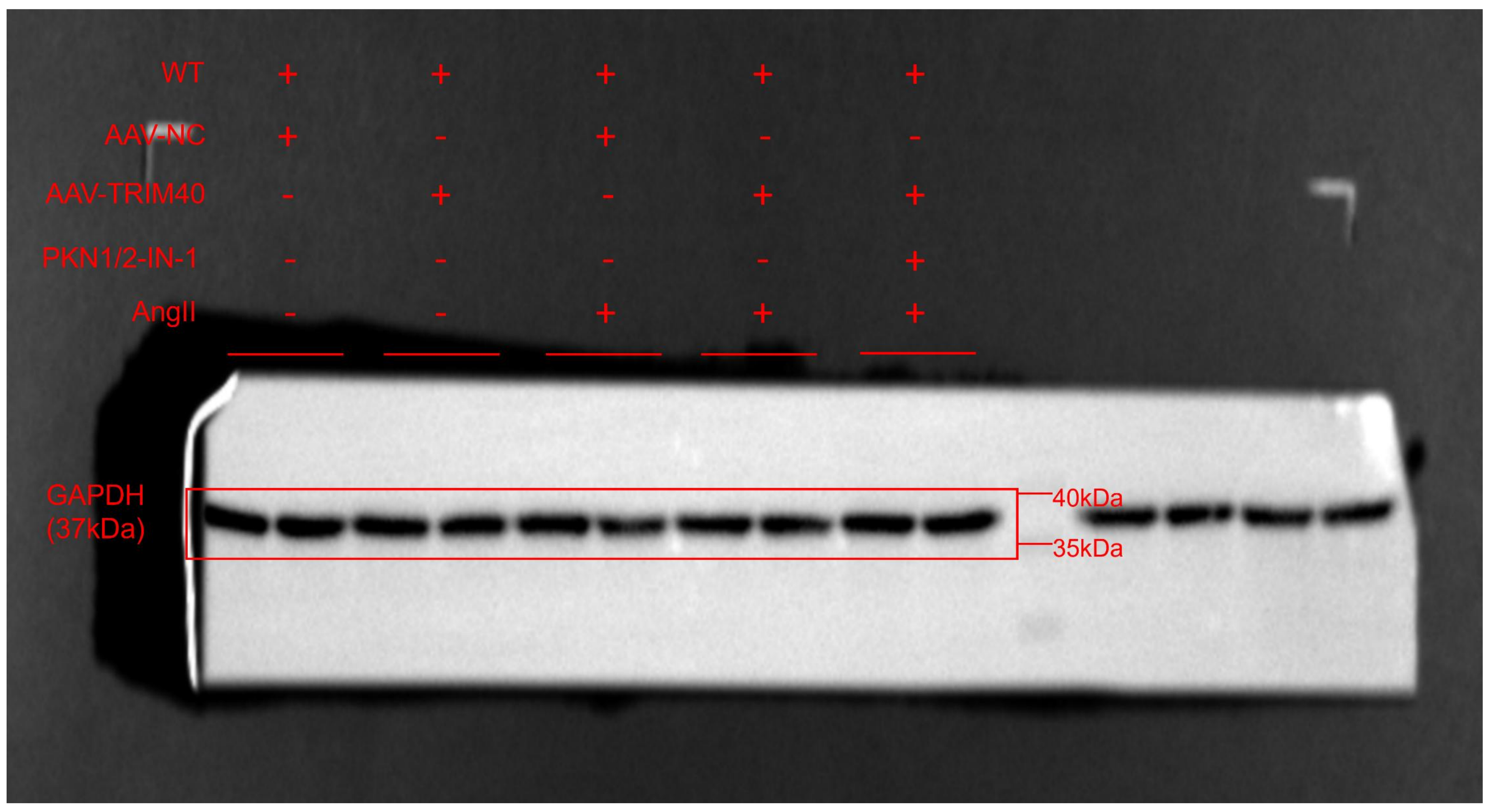
WT
AAV-NC
AAV-TRIM40
PKN1/2-IN-1
AngII
GAPDH
(37kDa)
40kDa
35kDa

Figure 9M

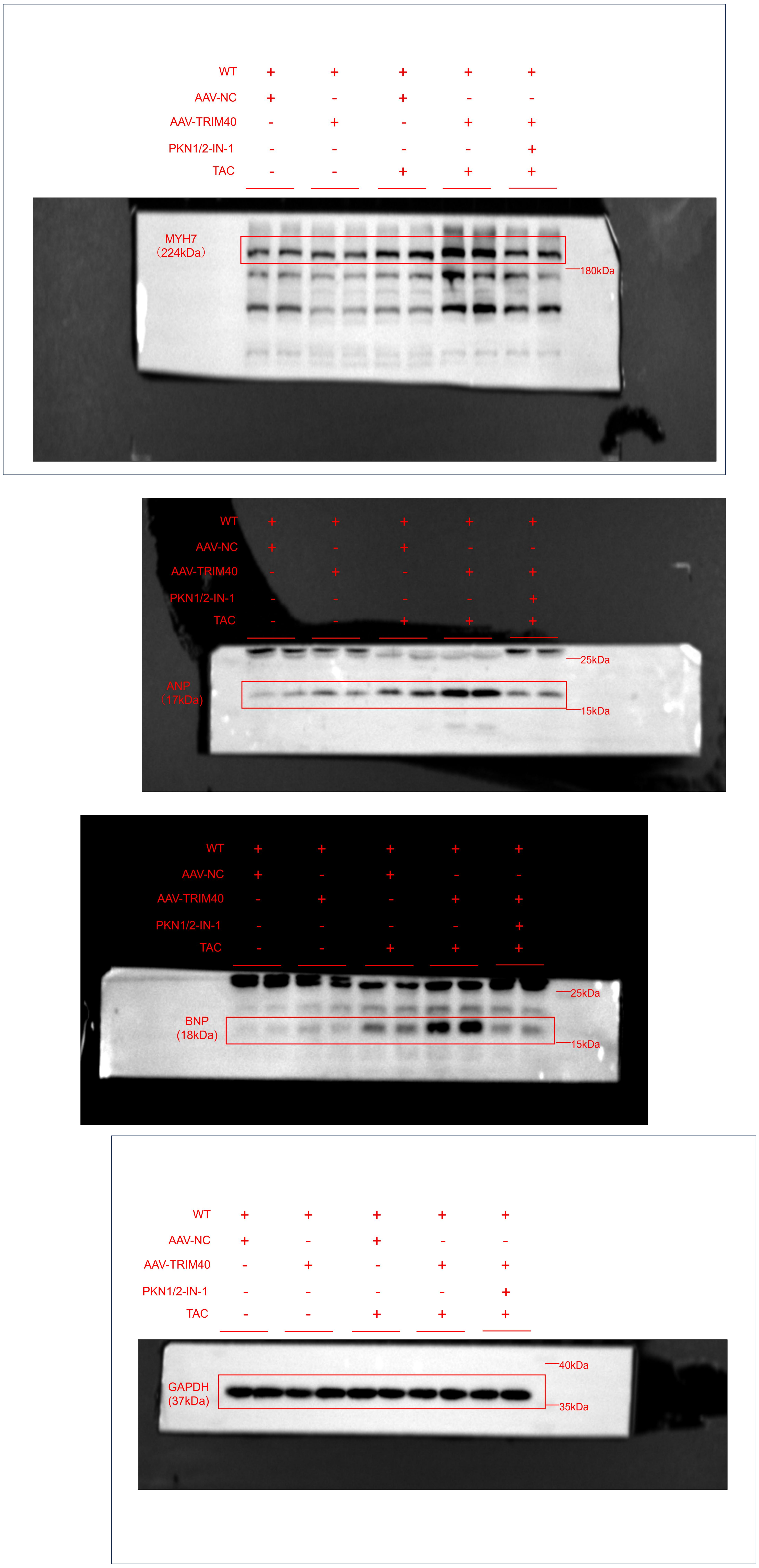

WT + + + + +
AAV-NC + - + - -
AAV-TRIM40 - + - + +
PKN1/2-IN-1 - - - - +
TAC - - + + +
MYH7 (224kDa)
180kDa
ANP (17kDa)
25kDa
15kDa
BNP (18kDa)
25kDa
15kDa
GAPDH (37kDa)
40kDa
35kDa

Figure 9P

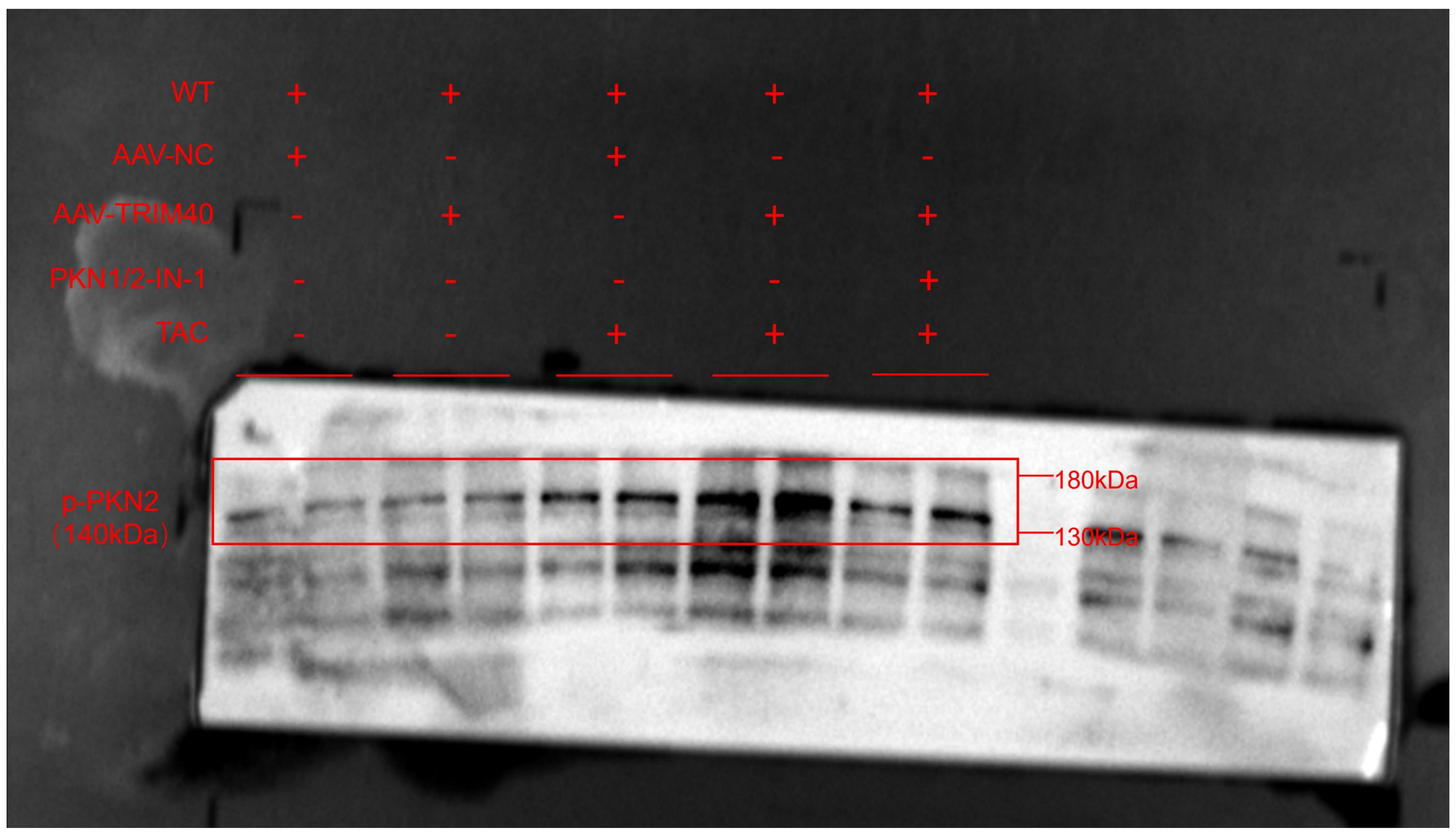
WT
AAV-NC
AAV-TRIM40
PKN1/2-IN-1
TAC
p-PKN2
(140kDa)
180kDa
130kDa

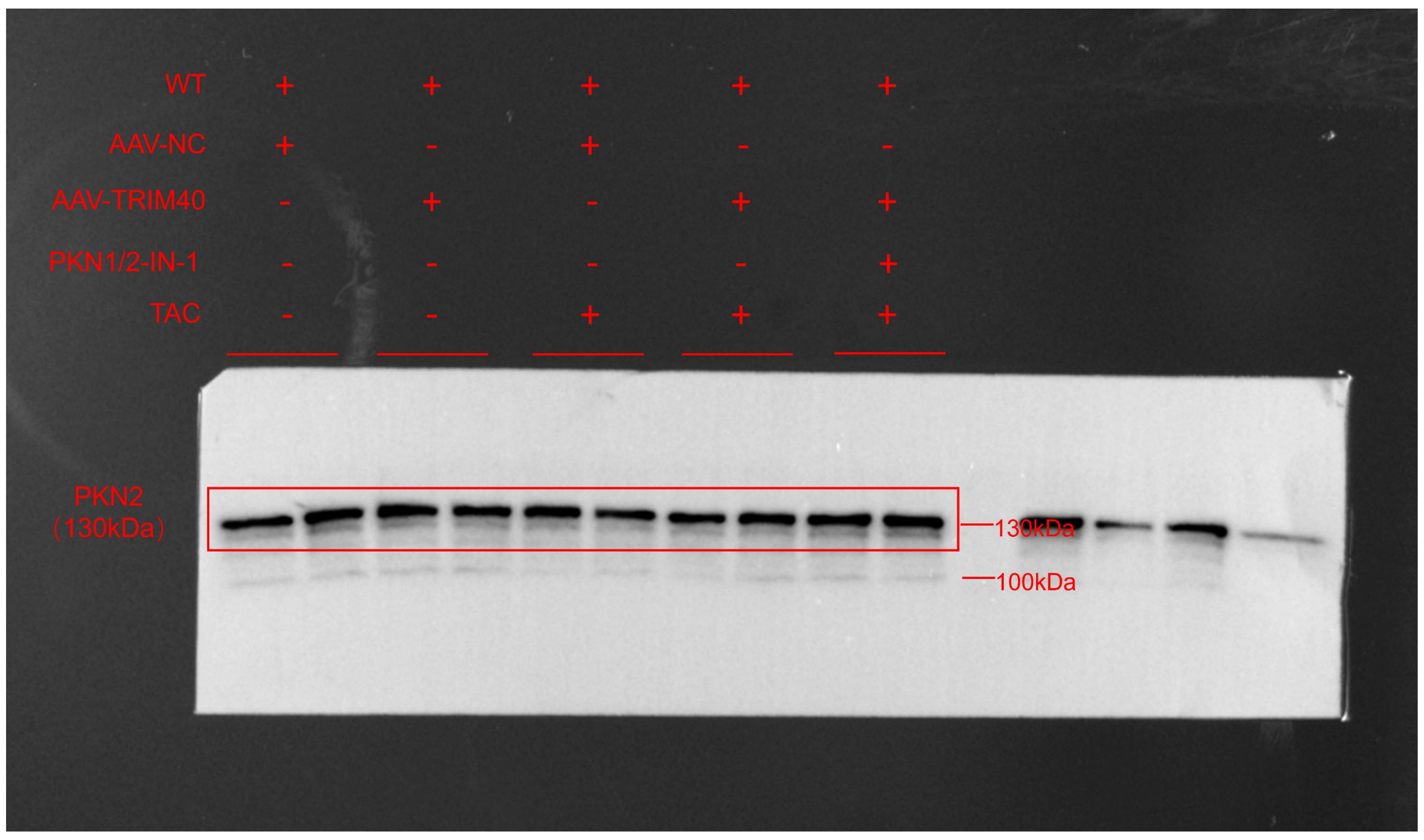
WT
AAV-NC
AAV-TRIM40
PKN1/2-IN-1
TAC
PKN2
(130kDa)
130kDa
100kDa

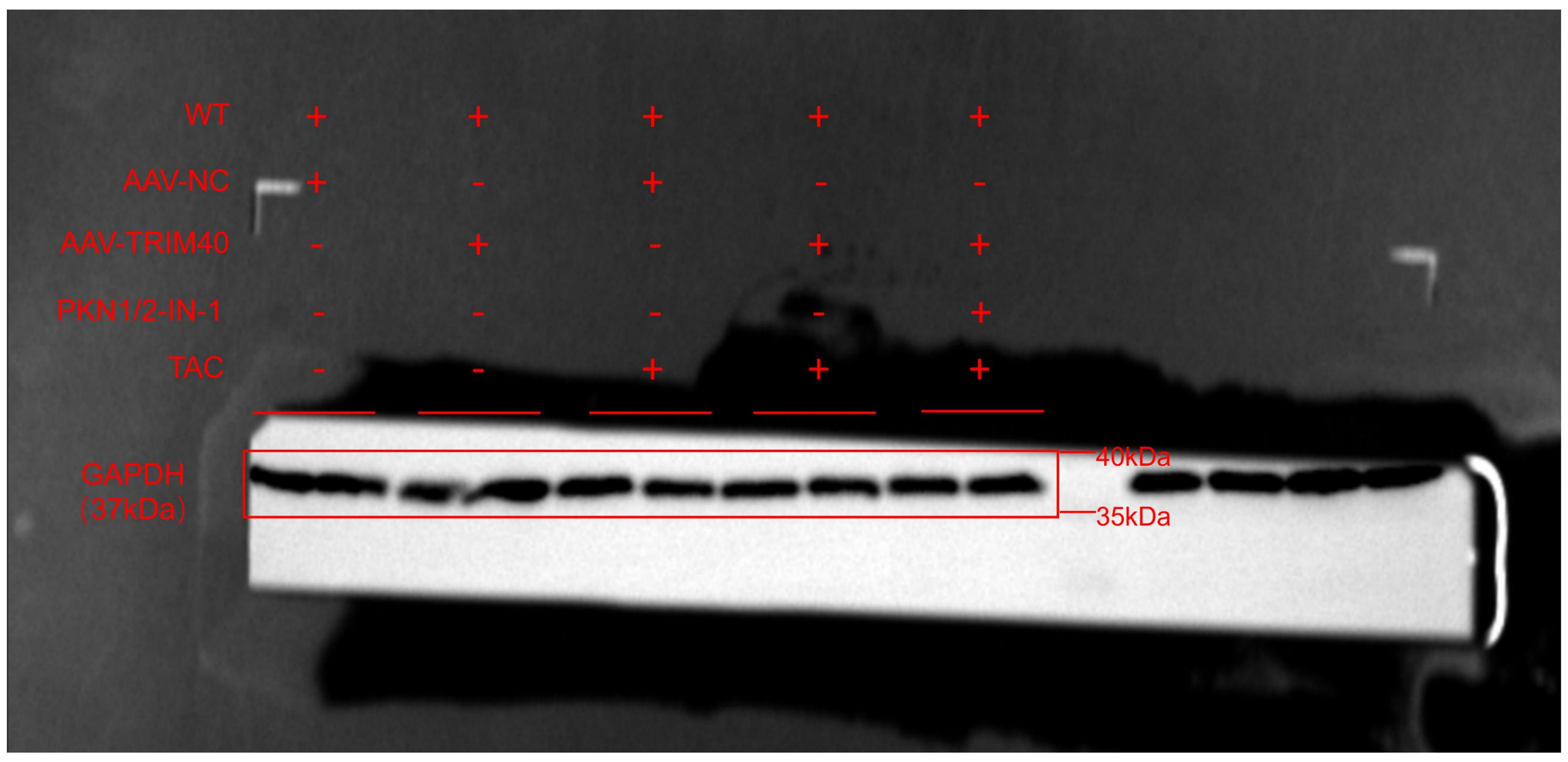
WT
AAV-NC
AAV-TRIM40
PKN1/2-IN-1
TAC
GAPDH
(37kDa)
40kDa
35kDa
