# Supplementary figures and images for: TRIM40 Drives Pathological Cardiac Hypertrophy and Heart Failure via Ubiquitination of PKN2 (part 3 of 3)
Source: Adv Sci (Weinh). 2026 Jan 22;13(17):e21337. doi: 10.1002/advs.202521337 (PMC13042792; doi:10.1002/advs.202521337)

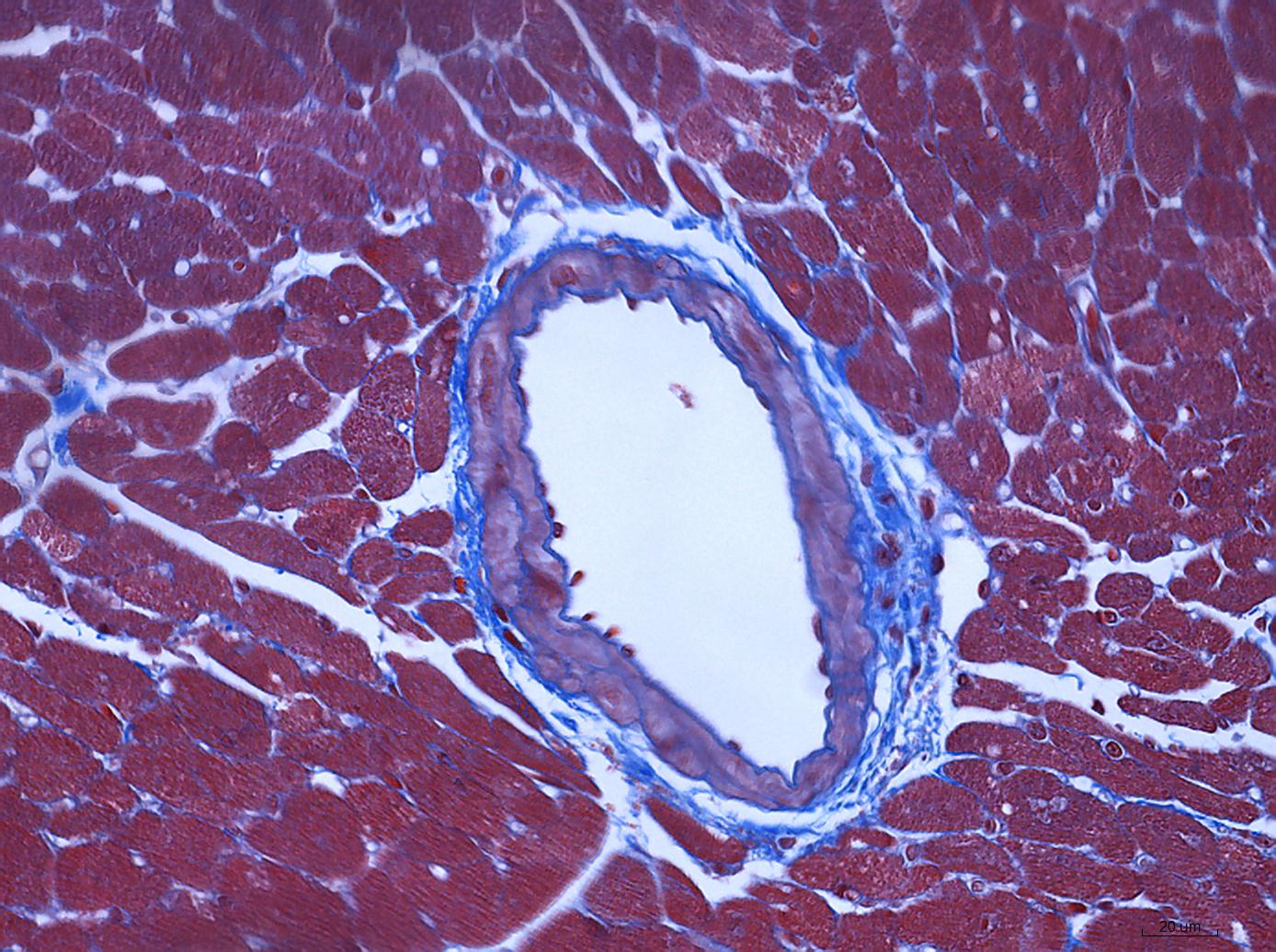

Supplement: Supplementary file 4 — Supporting File 4: advs73796‐sup‐0004‐Data.zip. [file ADVS-13-e21337-s003.zip › advs73796-sup-0004-Data/IHC_Raw_Data_Figures/Figure S4D_RawData_Figures/Masson-AAV9-cTnT- shTRIM40 + Sham-40X.jpg]

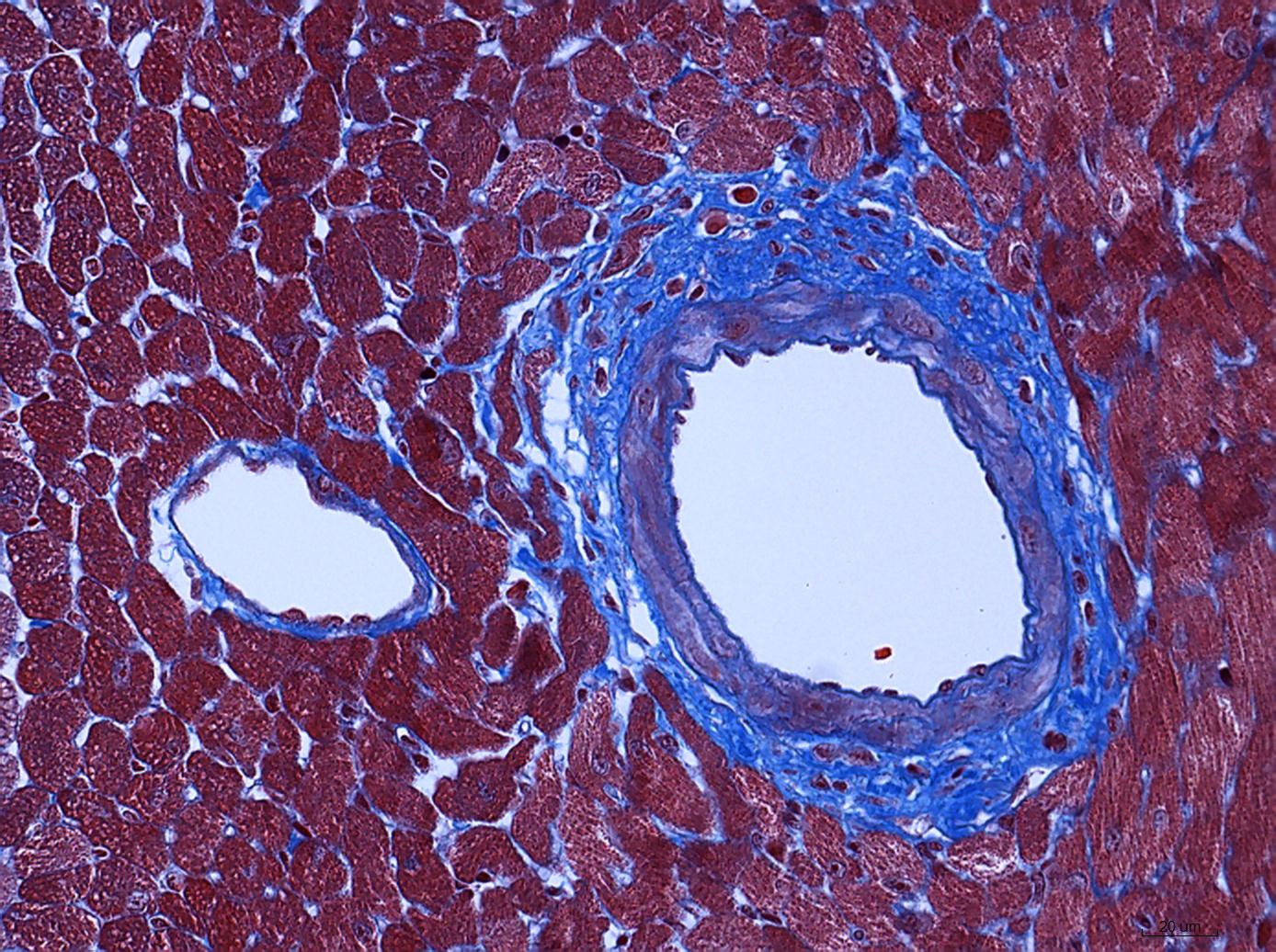

Supplement: Supplementary file 4 — Supporting File 4: advs73796‐sup‐0004‐Data.zip. [file ADVS-13-e21337-s003.zip › advs73796-sup-0004-Data/IHC_Raw_Data_Figures/Figure S4D_RawData_Figures/Masson-AAV9-cTnT-NC + AngII-40X.jpg]

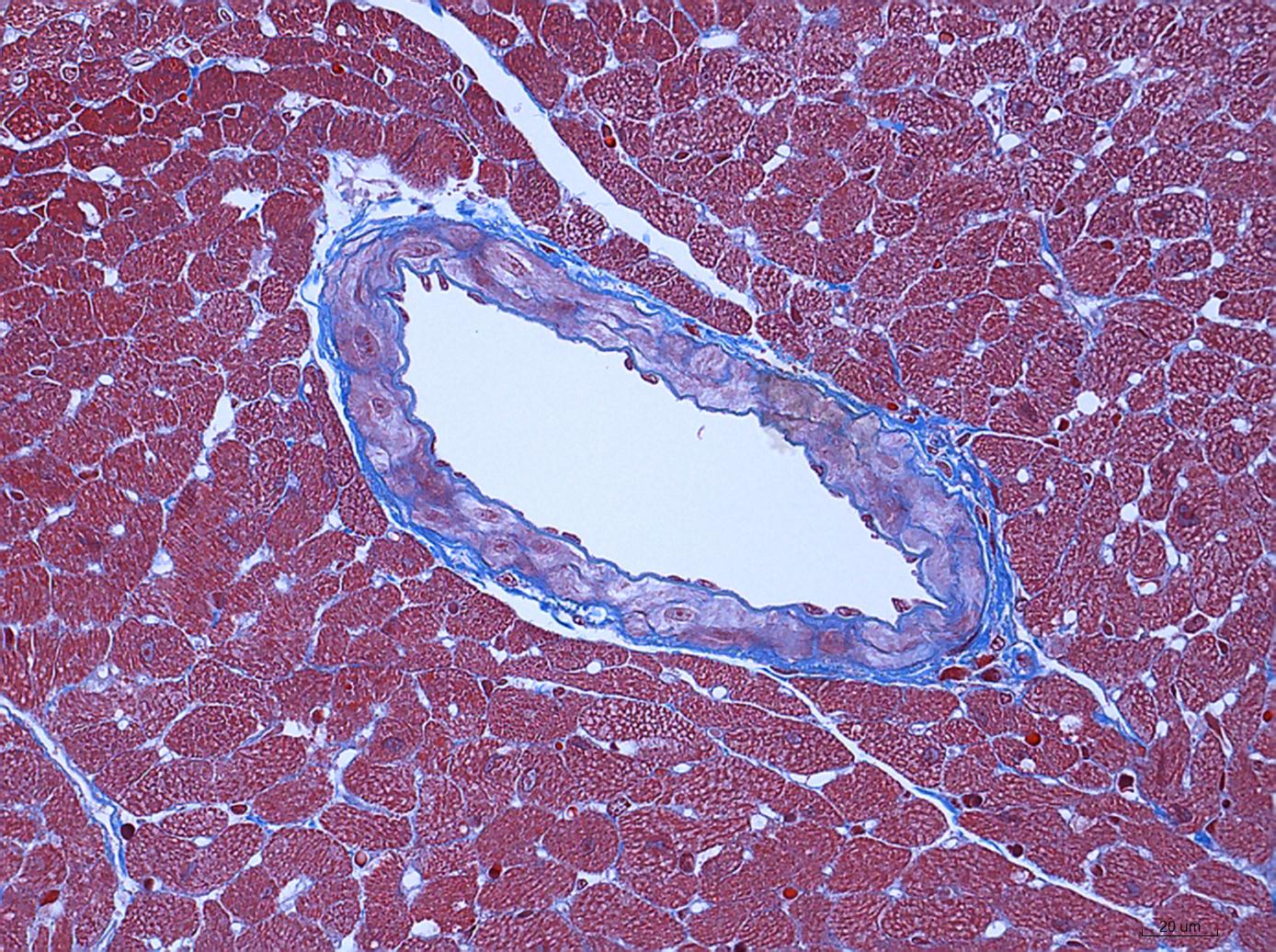

Supplement: Supplementary file 4 — Supporting File 4: advs73796‐sup‐0004‐Data.zip. [file ADVS-13-e21337-s003.zip › advs73796-sup-0004-Data/IHC_Raw_Data_Figures/Figure S4D_RawData_Figures/Masson-AAV9-cTnT-NC + Sham-40X.jpg]

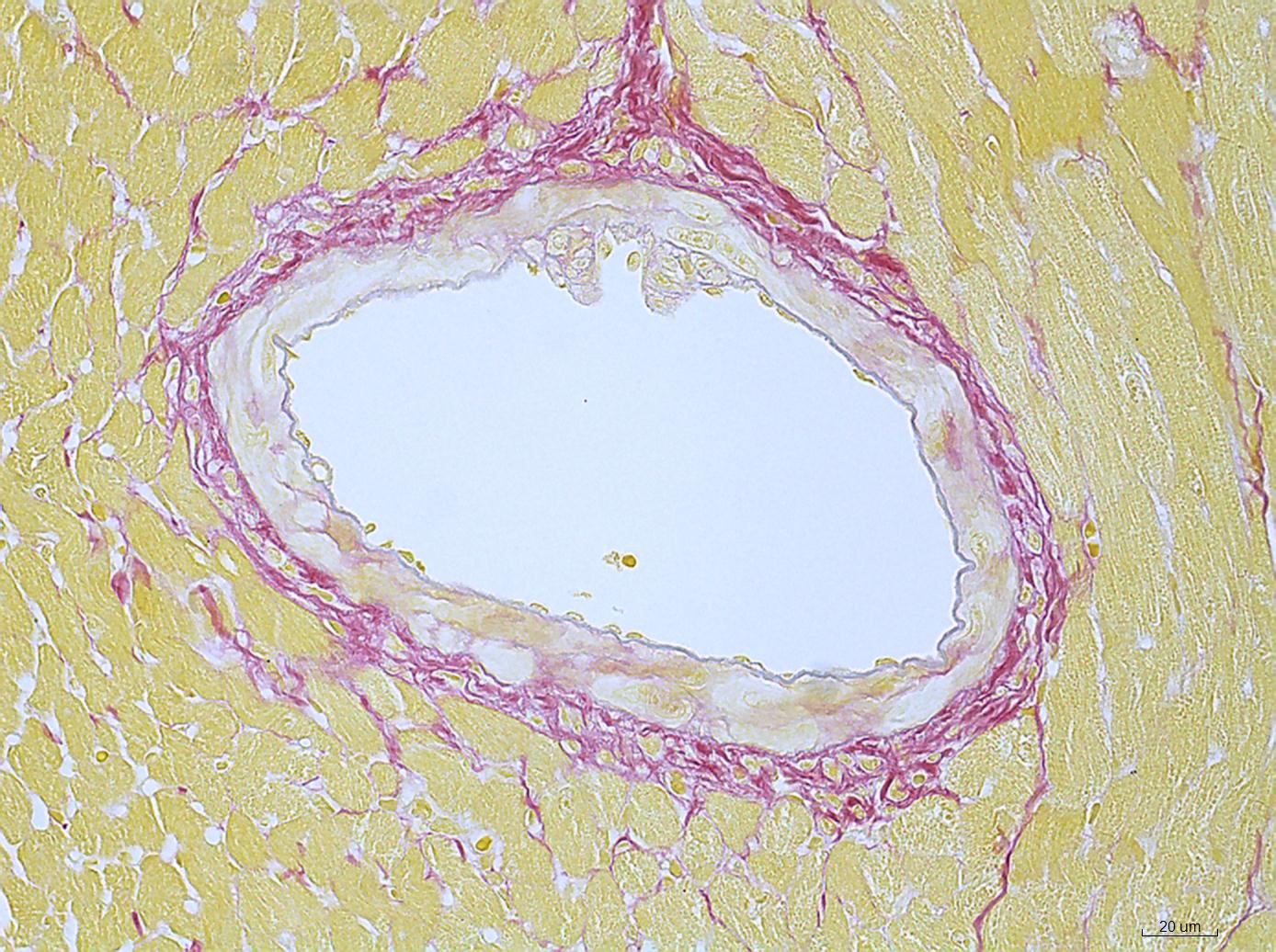

Supplement: Supplementary file 4 — Supporting File 4: advs73796‐sup‐0004‐Data.zip. [file ADVS-13-e21337-s003.zip › advs73796-sup-0004-Data/IHC_Raw_Data_Figures/Figure S4G_RawData_Figures/Sirius red-AAV9-cTnT- shTRIM40 + Ang II-40X.jpg]

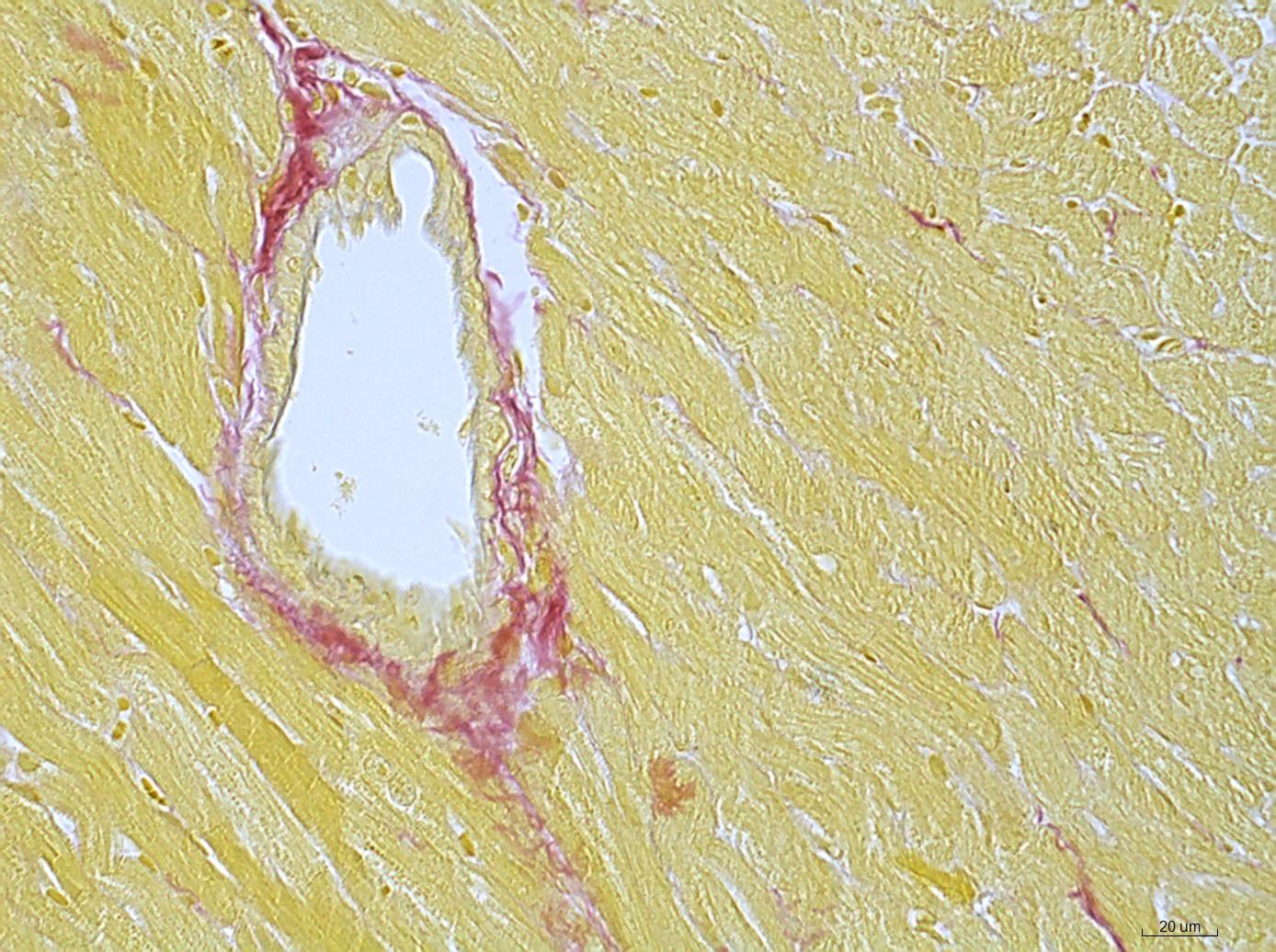

Supplement: Supplementary file 4 — Supporting File 4: advs73796‐sup‐0004‐Data.zip. [file ADVS-13-e21337-s003.zip › advs73796-sup-0004-Data/IHC_Raw_Data_Figures/Figure S4G_RawData_Figures/Sirius red-AAV9-cTnT- shTRIM40 + Sham-40X.jpg]

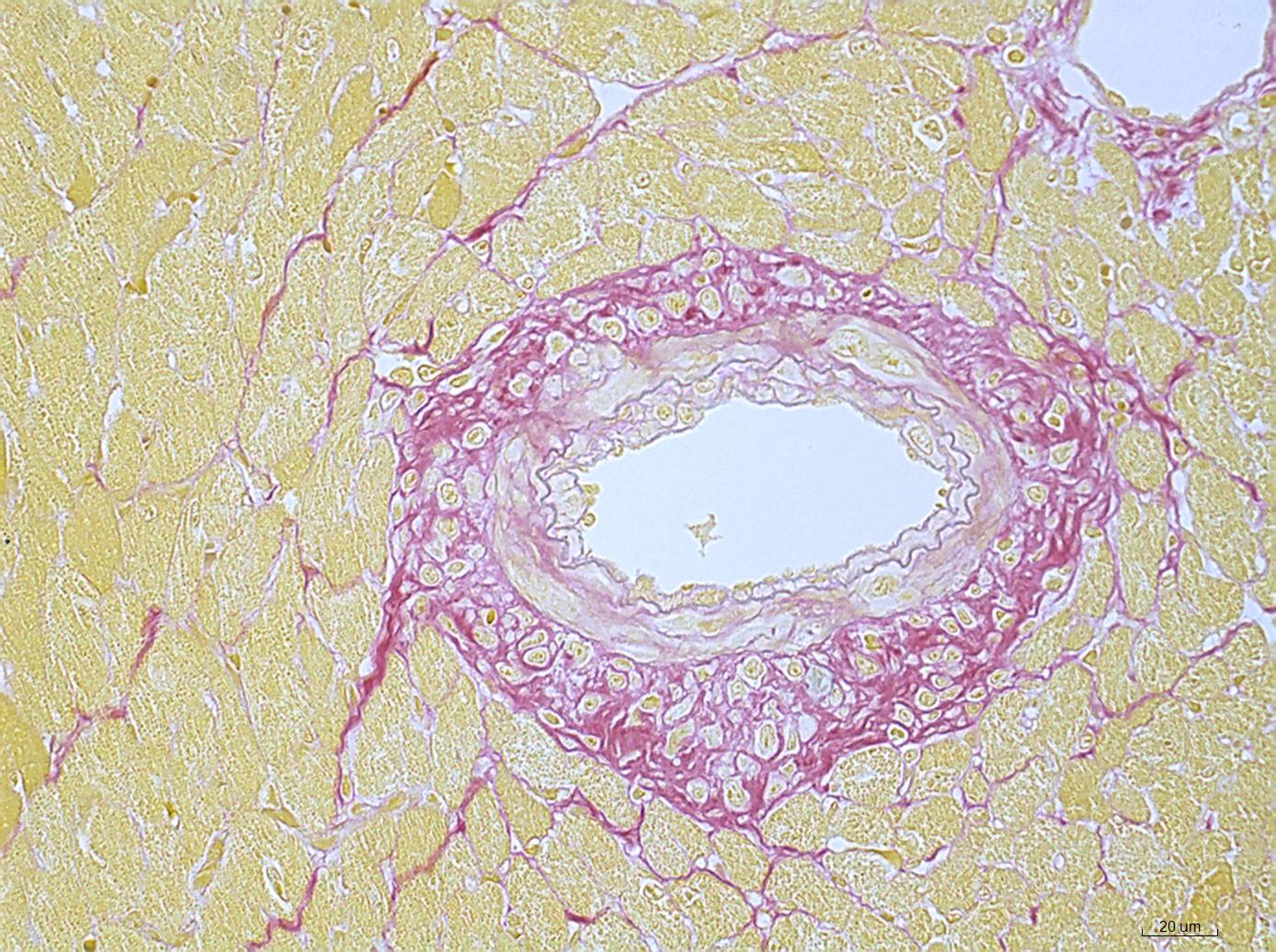

Supplement: Supplementary file 4 — Supporting File 4: advs73796‐sup‐0004‐Data.zip. [file ADVS-13-e21337-s003.zip › advs73796-sup-0004-Data/IHC_Raw_Data_Figures/Figure S4G_RawData_Figures/Sirius red-AAV9-cTnT-NC + AngII-40X.jpg]

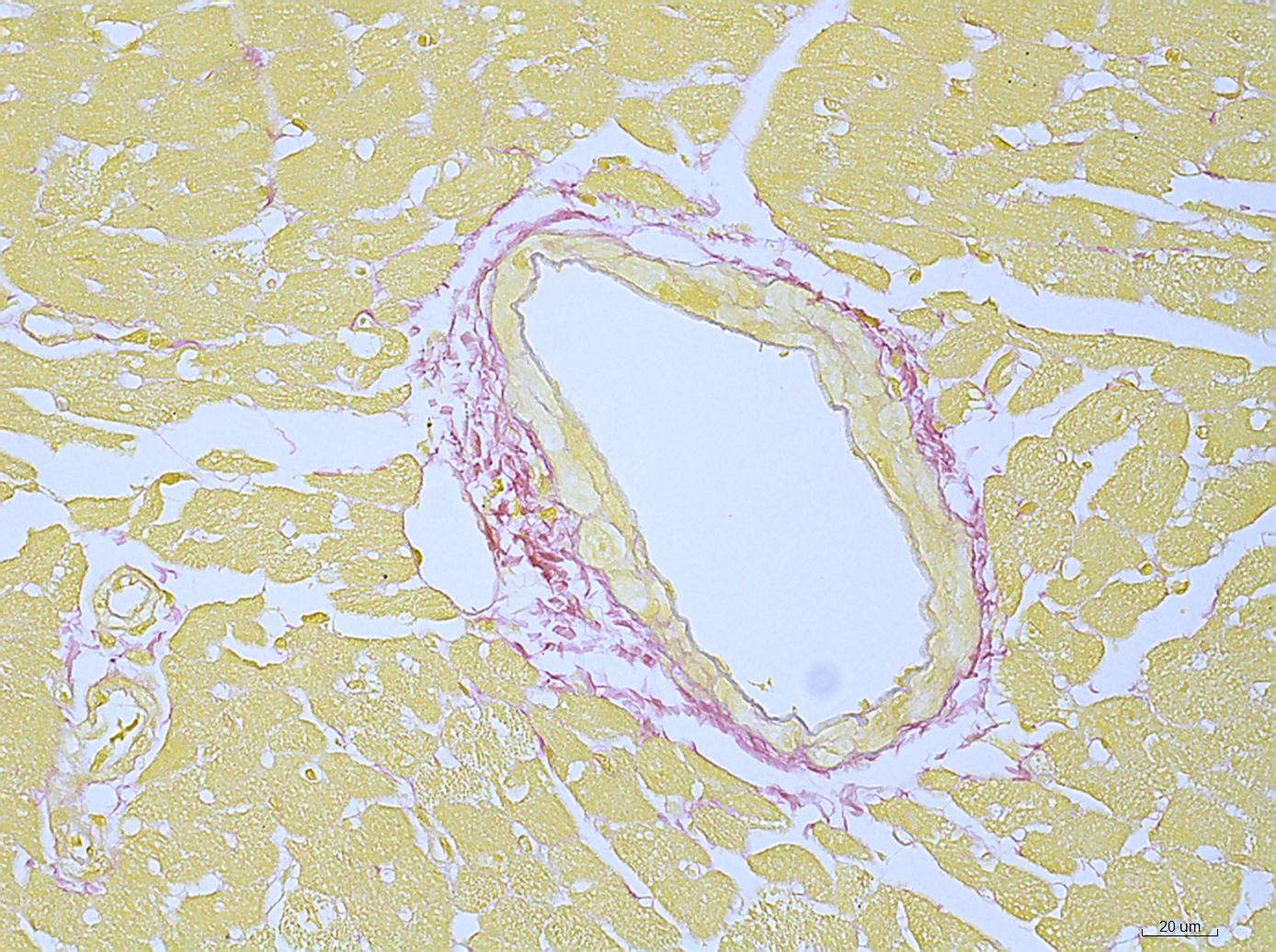

Supplement: Supplementary file 4 — Supporting File 4: advs73796‐sup‐0004‐Data.zip. [file ADVS-13-e21337-s003.zip › advs73796-sup-0004-Data/IHC_Raw_Data_Figures/Figure S4G_RawData_Figures/Sirius red-AAV9-cTnT-NC + Sham-40X.jpg]

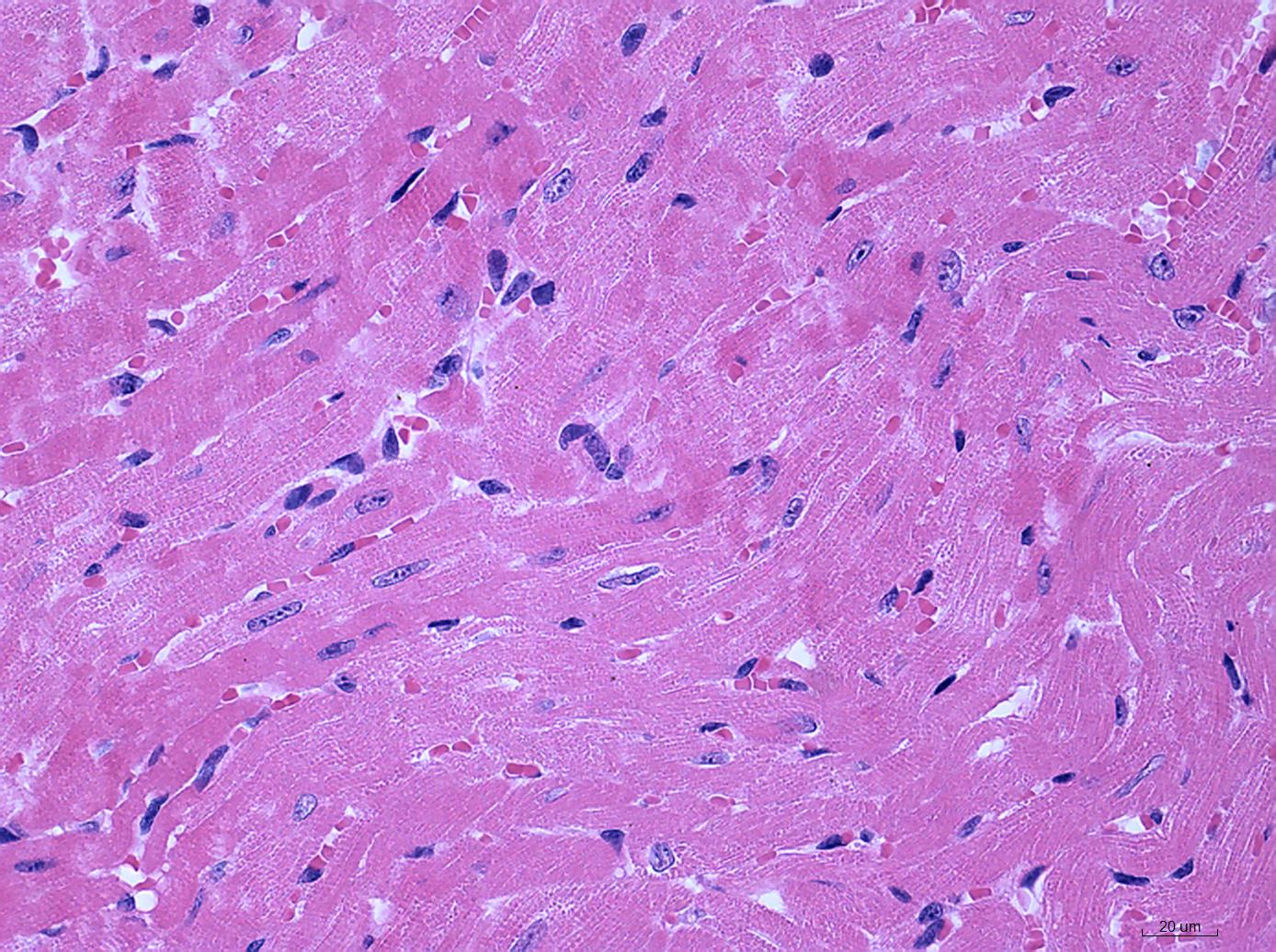

Supplement: Supplementary file 4 — Supporting File 4: advs73796‐sup‐0004‐Data.zip. [file ADVS-13-e21337-s003.zip › advs73796-sup-0004-Data/IHC_Raw_Data_Figures/Figure S8F_RawData_Figures/H&E-Ang II-WT-AAV9-NC-40X.jpg]

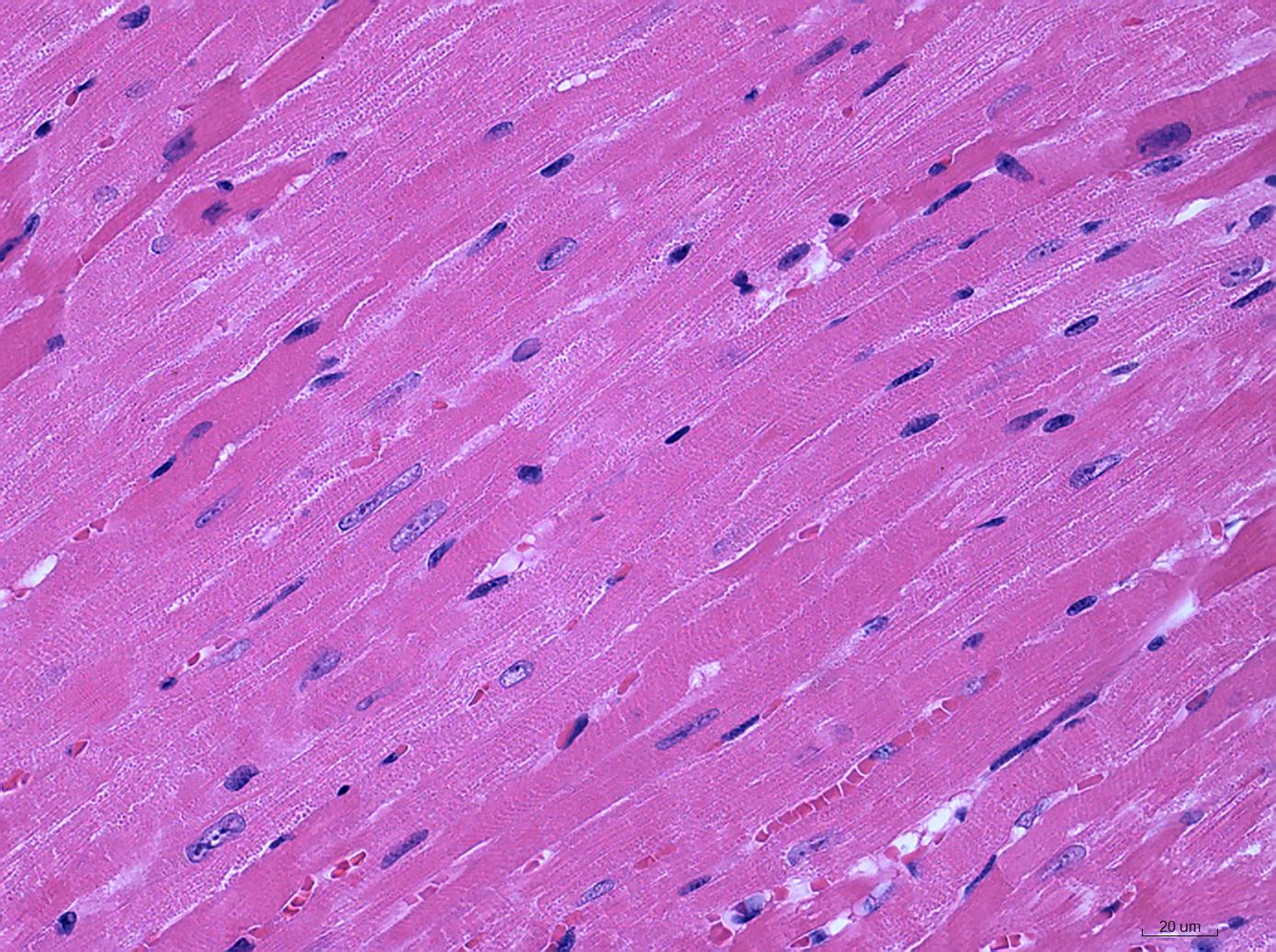

Supplement: Supplementary file 4 — Supporting File 4: advs73796‐sup‐0004‐Data.zip. [file ADVS-13-e21337-s003.zip › advs73796-sup-0004-Data/IHC_Raw_Data_Figures/Figure S8F_RawData_Figures/H&E-Ang II-WT-AAV9-TRIM40+PKN1&2-IN-1-40X.jpg]

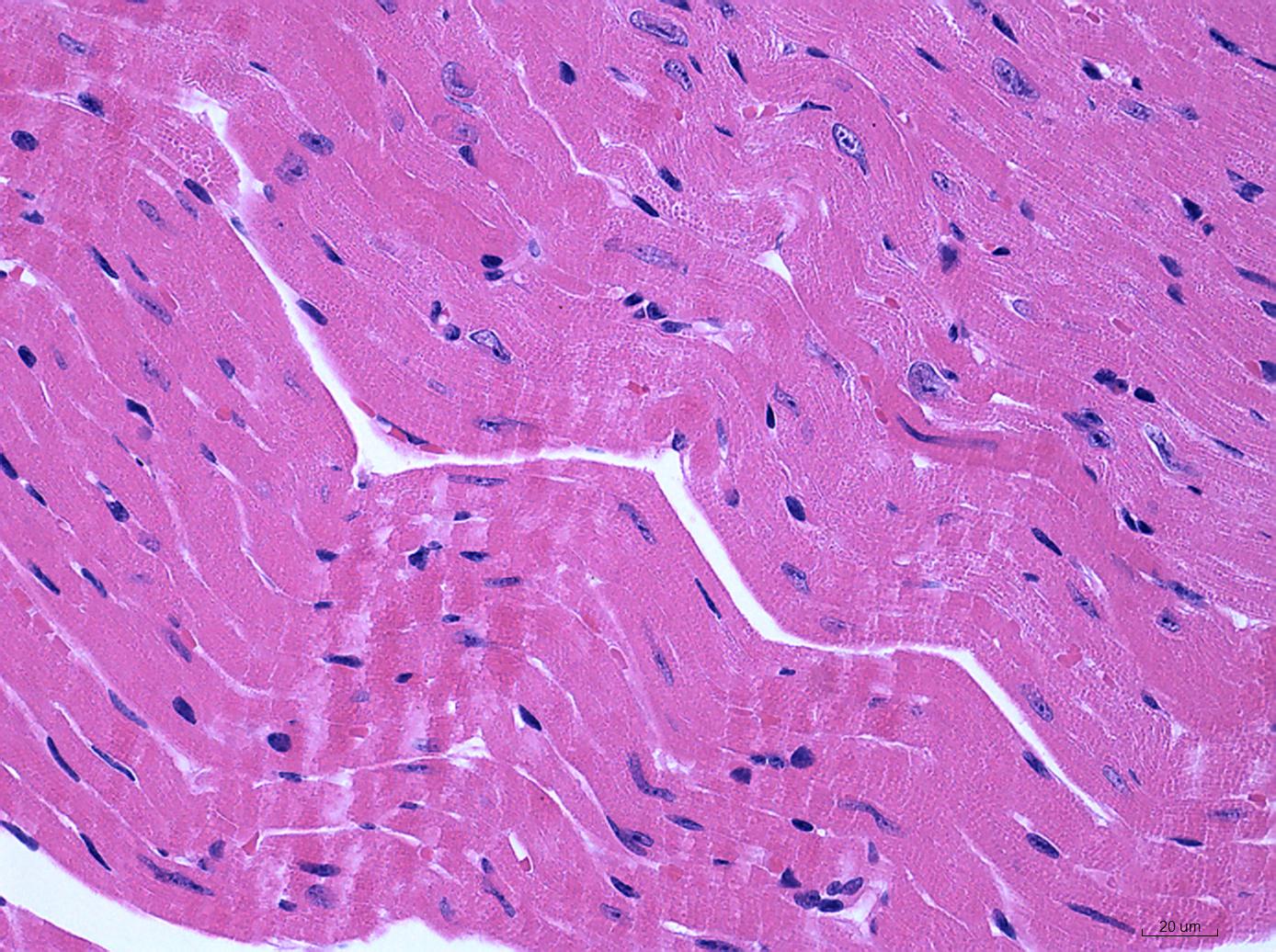

Supplement: Supplementary file 4 — Supporting File 4: advs73796‐sup‐0004‐Data.zip. [file ADVS-13-e21337-s003.zip › advs73796-sup-0004-Data/IHC_Raw_Data_Figures/Figure S8F_RawData_Figures/H&E-Ang II-WT-AAV9-TRIM40-40X.jpg]

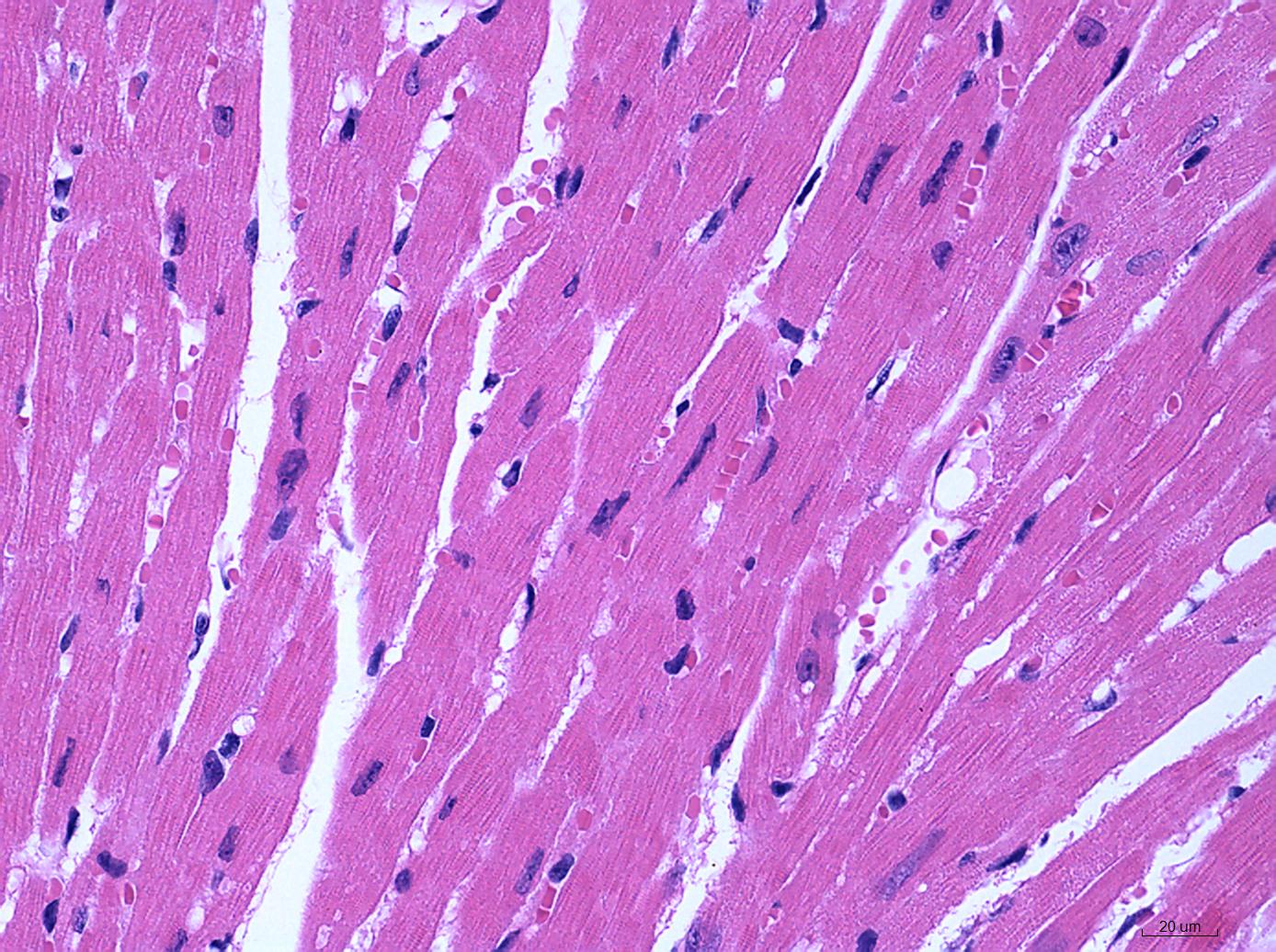

Supplement: Supplementary file 4 — Supporting File 4: advs73796‐sup‐0004‐Data.zip. [file ADVS-13-e21337-s003.zip › advs73796-sup-0004-Data/IHC_Raw_Data_Figures/Figure S8F_RawData_Figures/H&E-WT-AAV9-NC-40X.jpg]

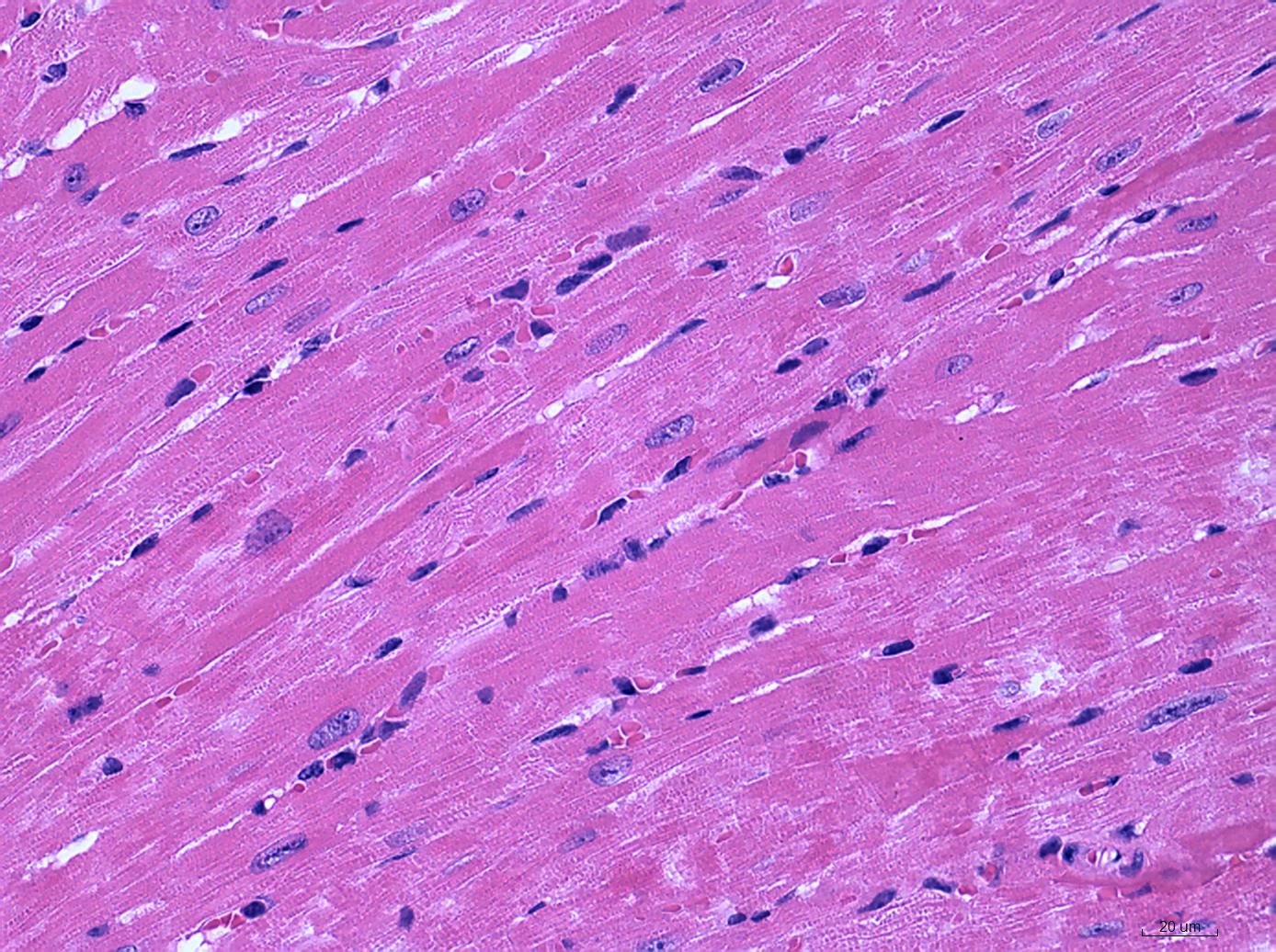

Supplement: Supplementary file 4 — Supporting File 4: advs73796‐sup‐0004‐Data.zip. [file ADVS-13-e21337-s003.zip › advs73796-sup-0004-Data/IHC_Raw_Data_Figures/Figure S8F_RawData_Figures/H&E-WT-AAV9-TRIM40-40X.jpg]

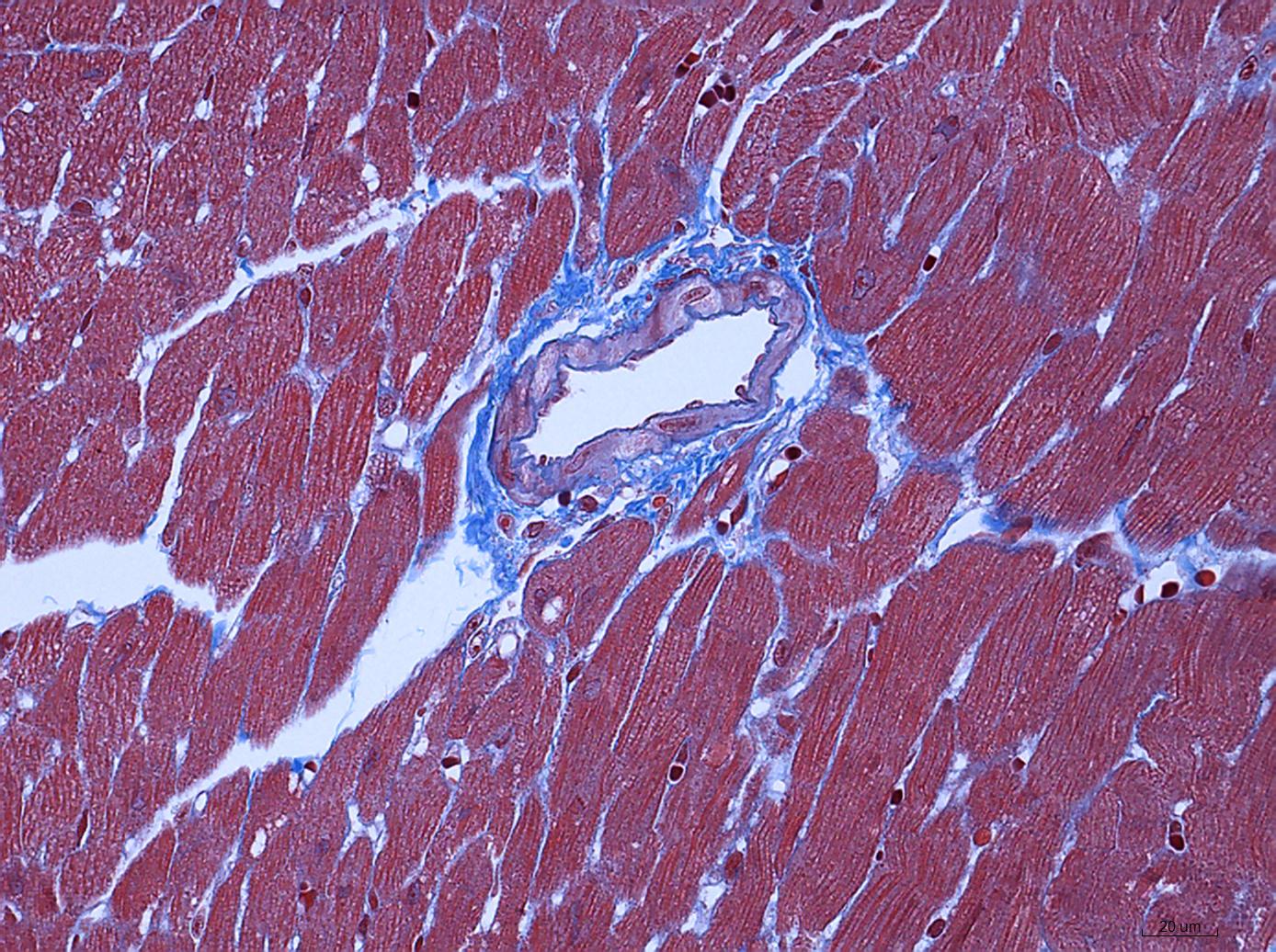

Supplement: Supplementary file 4 — Supporting File 4: advs73796‐sup‐0004‐Data.zip. [file ADVS-13-e21337-s003.zip › advs73796-sup-0004-Data/IHC_Raw_Data_Figures/Figure S8H_RawData_Figures/Masson-Ang II-WT-AAV9-NC-40X.jpg]

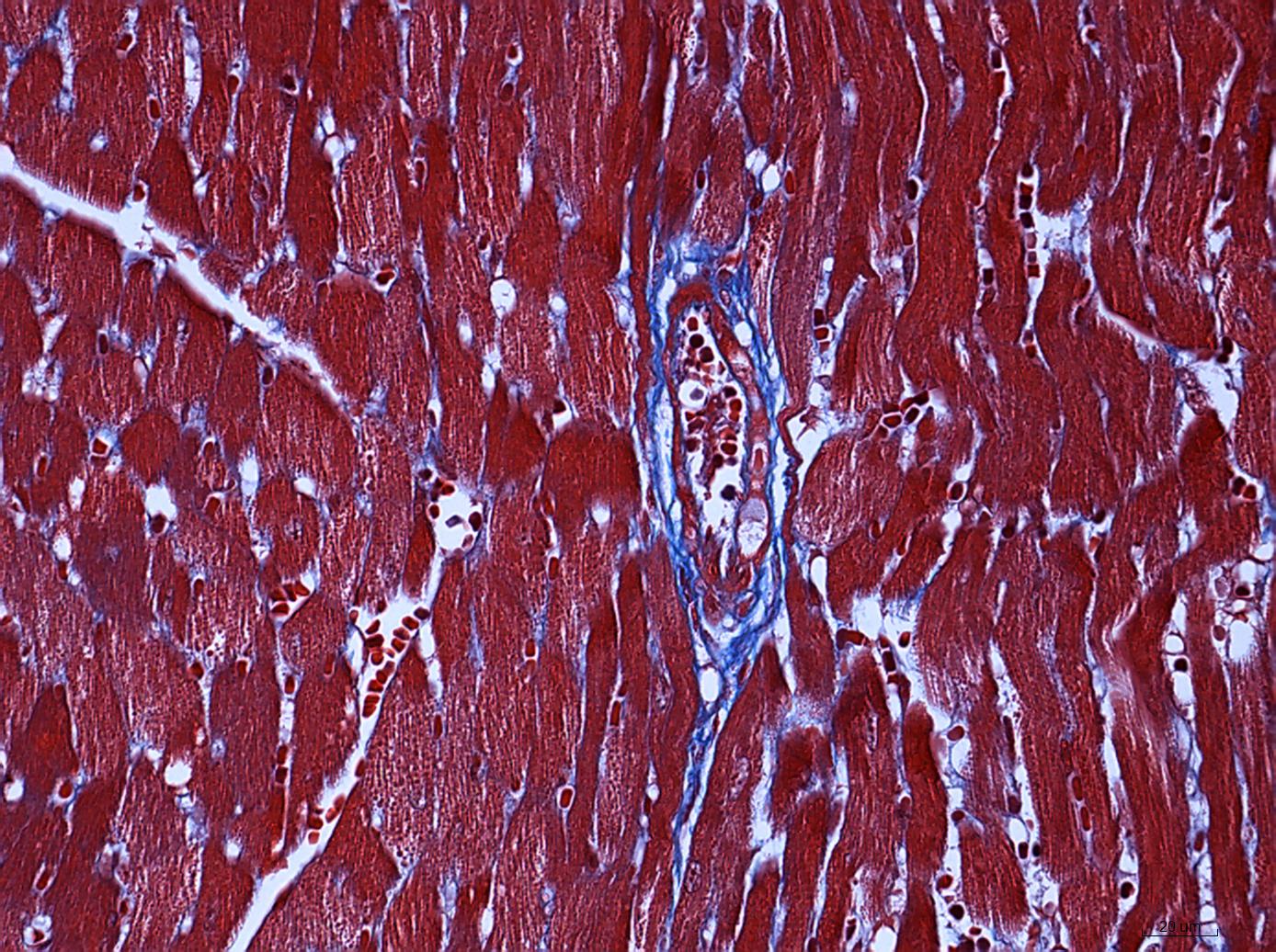

Supplement: Supplementary file 4 — Supporting File 4: advs73796‐sup‐0004‐Data.zip. [file ADVS-13-e21337-s003.zip › advs73796-sup-0004-Data/IHC_Raw_Data_Figures/Figure S8H_RawData_Figures/Masson-Ang II-WT-AAV9-TRIM40+PKN1&2-IN-1-40X.jpg]

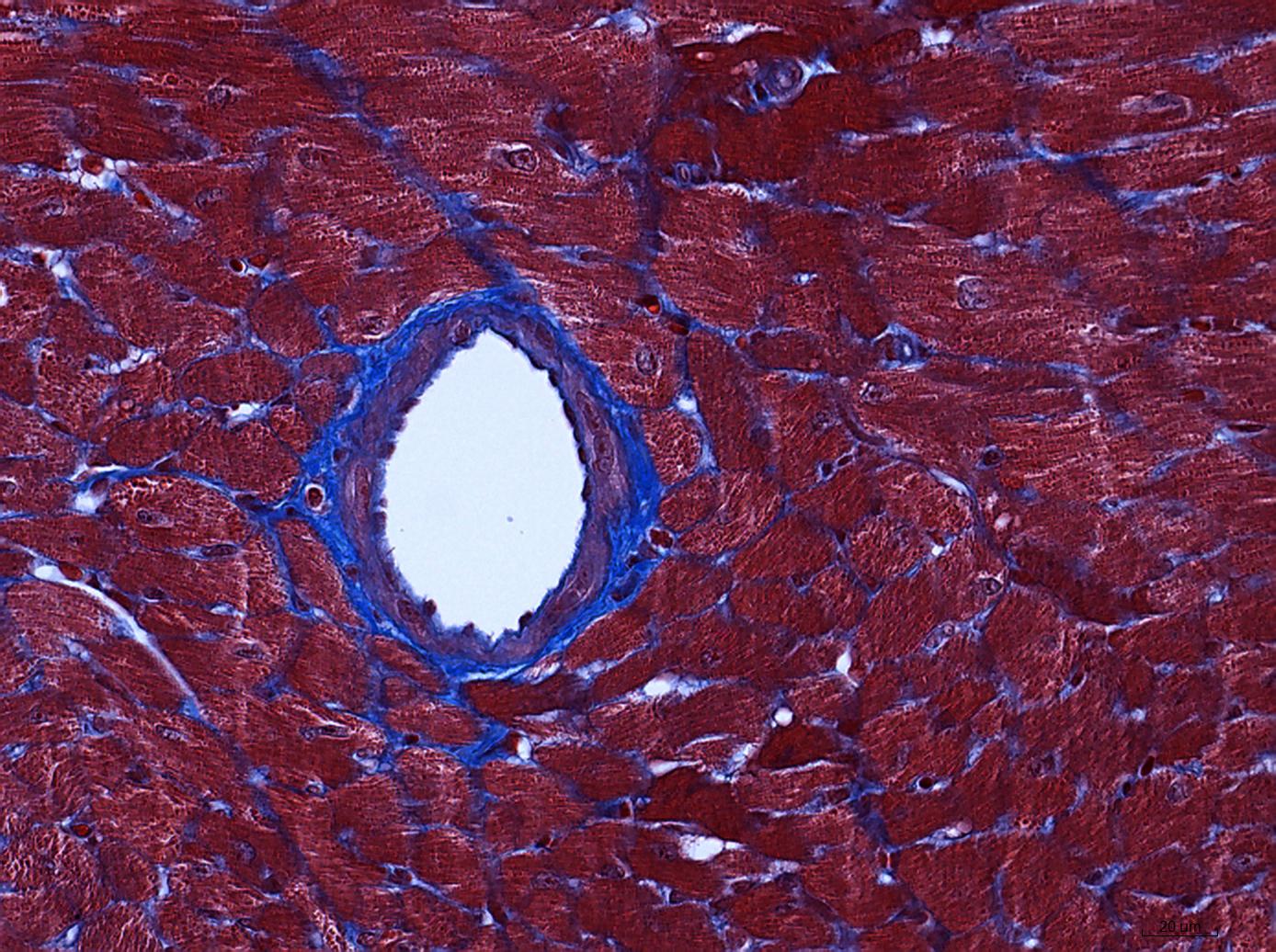

Supplement: Supplementary file 4 — Supporting File 4: advs73796‐sup‐0004‐Data.zip. [file ADVS-13-e21337-s003.zip › advs73796-sup-0004-Data/IHC_Raw_Data_Figures/Figure S8H_RawData_Figures/Masson-Ang II-WT-AAV9-TRIM40-40X.jpg]

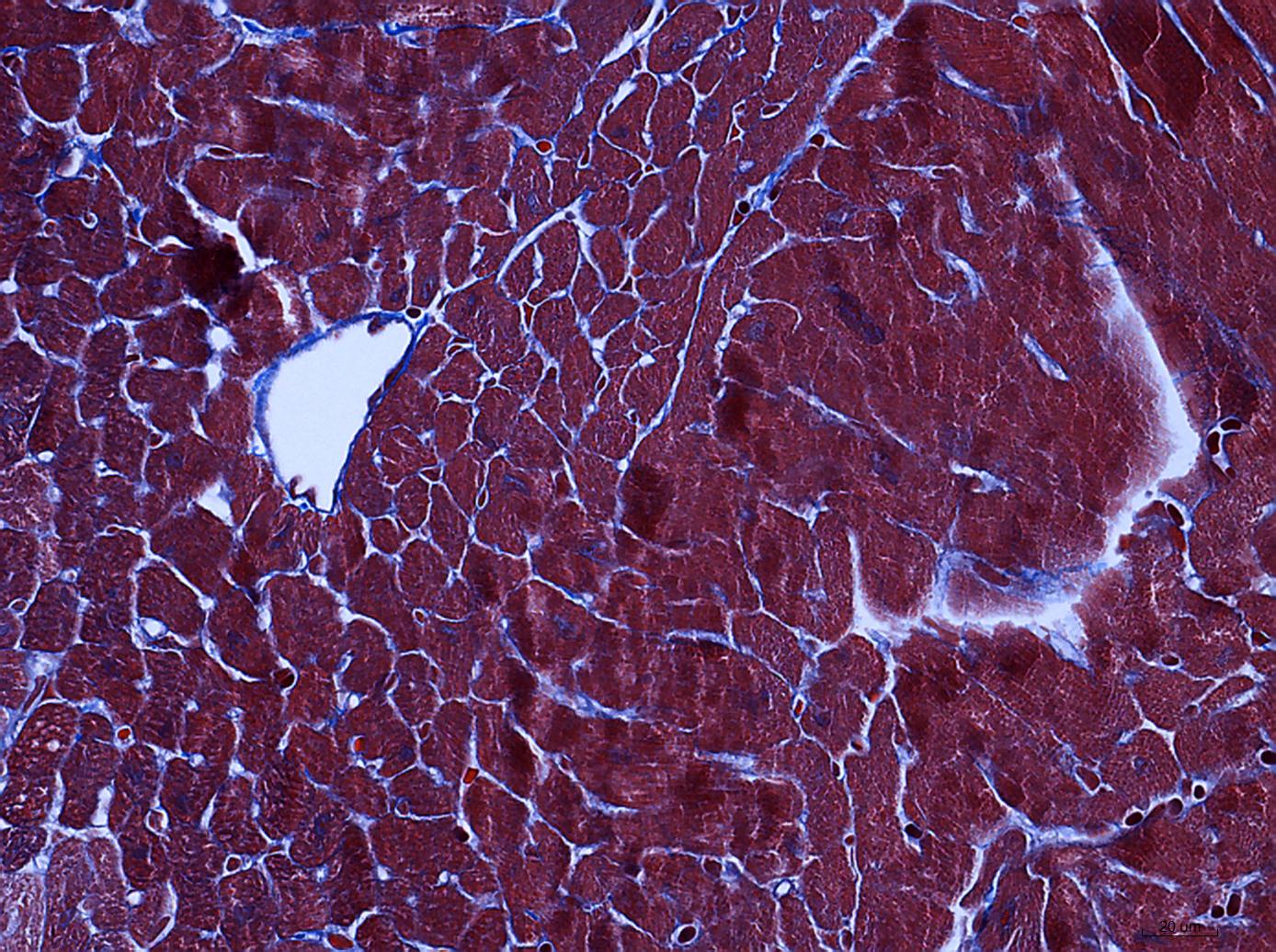

Supplement: Supplementary file 4 — Supporting File 4: advs73796‐sup‐0004‐Data.zip. [file ADVS-13-e21337-s003.zip › advs73796-sup-0004-Data/IHC_Raw_Data_Figures/Figure S8H_RawData_Figures/Masson-WT-AAV9-NC-40X.jpg]

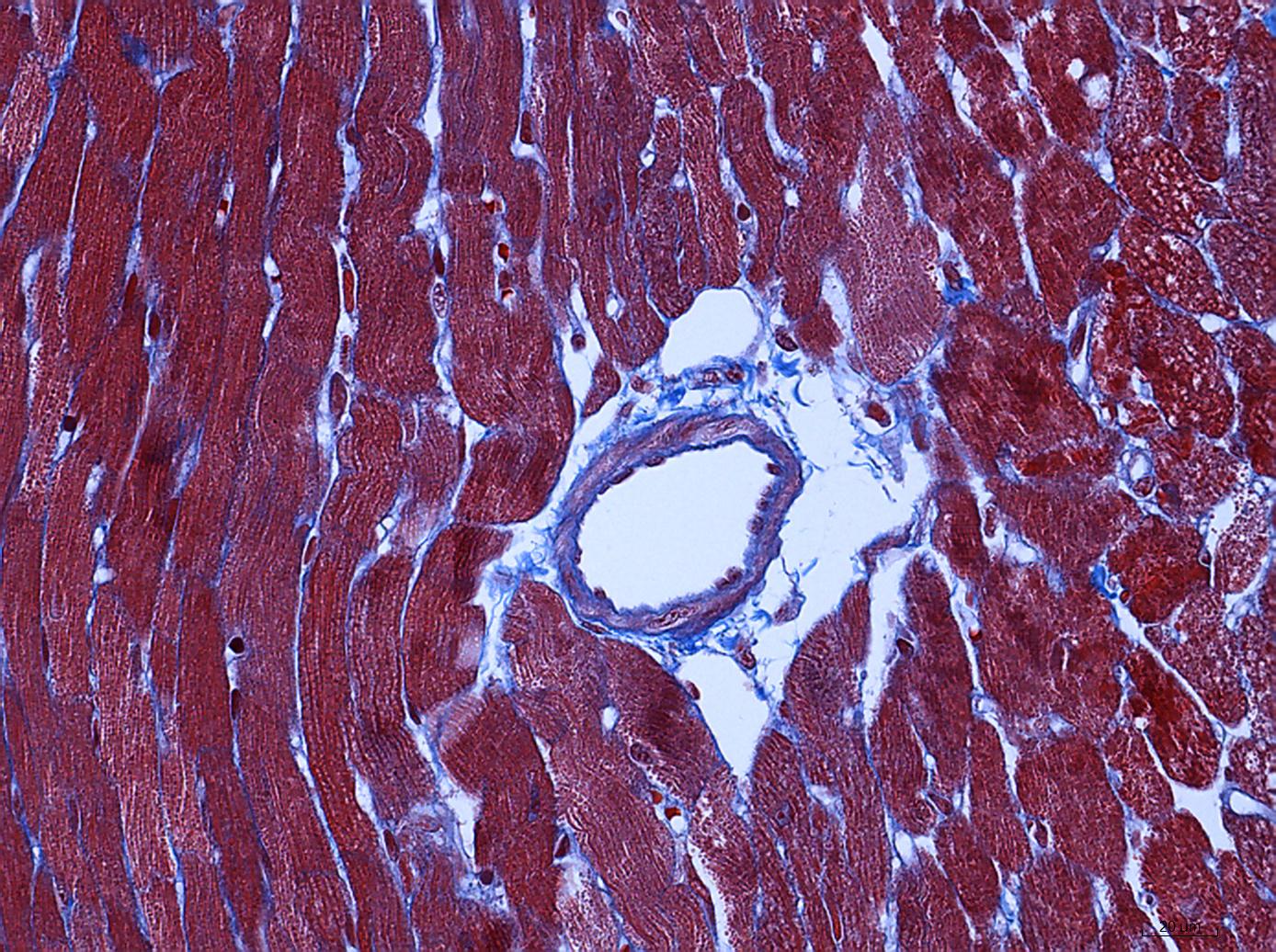

Supplement: Supplementary file 4 — Supporting File 4: advs73796‐sup‐0004‐Data.zip. [file ADVS-13-e21337-s003.zip › advs73796-sup-0004-Data/IHC_Raw_Data_Figures/Figure S8H_RawData_Figures/Masson-WT-AAV9-TRIM40-40X.jpg]

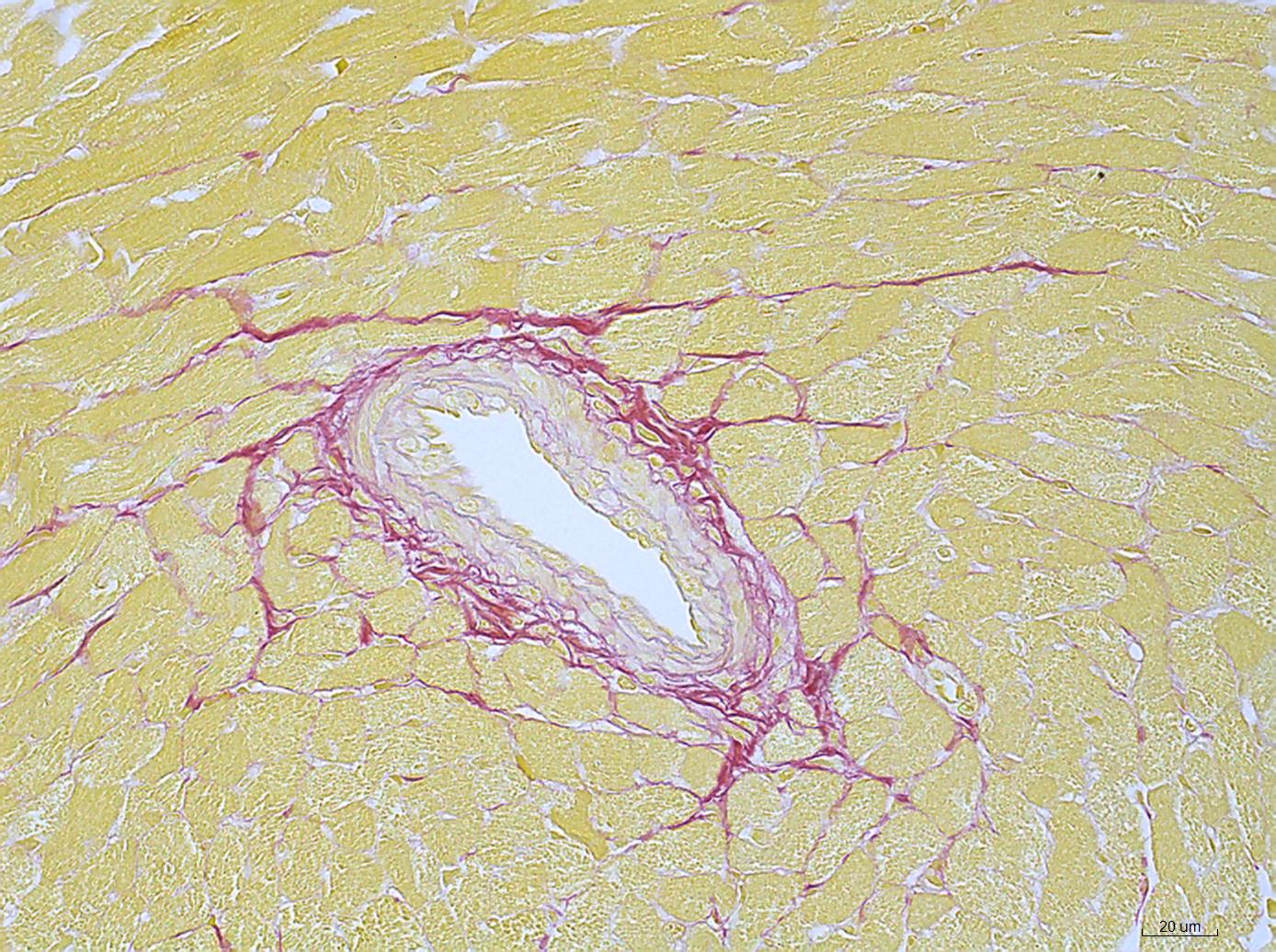

Supplement: Supplementary file 4 — Supporting File 4: advs73796‐sup‐0004‐Data.zip. [file ADVS-13-e21337-s003.zip › advs73796-sup-0004-Data/IHC_Raw_Data_Figures/Figure S8K_RawData_Figures/Sirius red-Ang II-WT-AAV9-NC-40X.jpg]

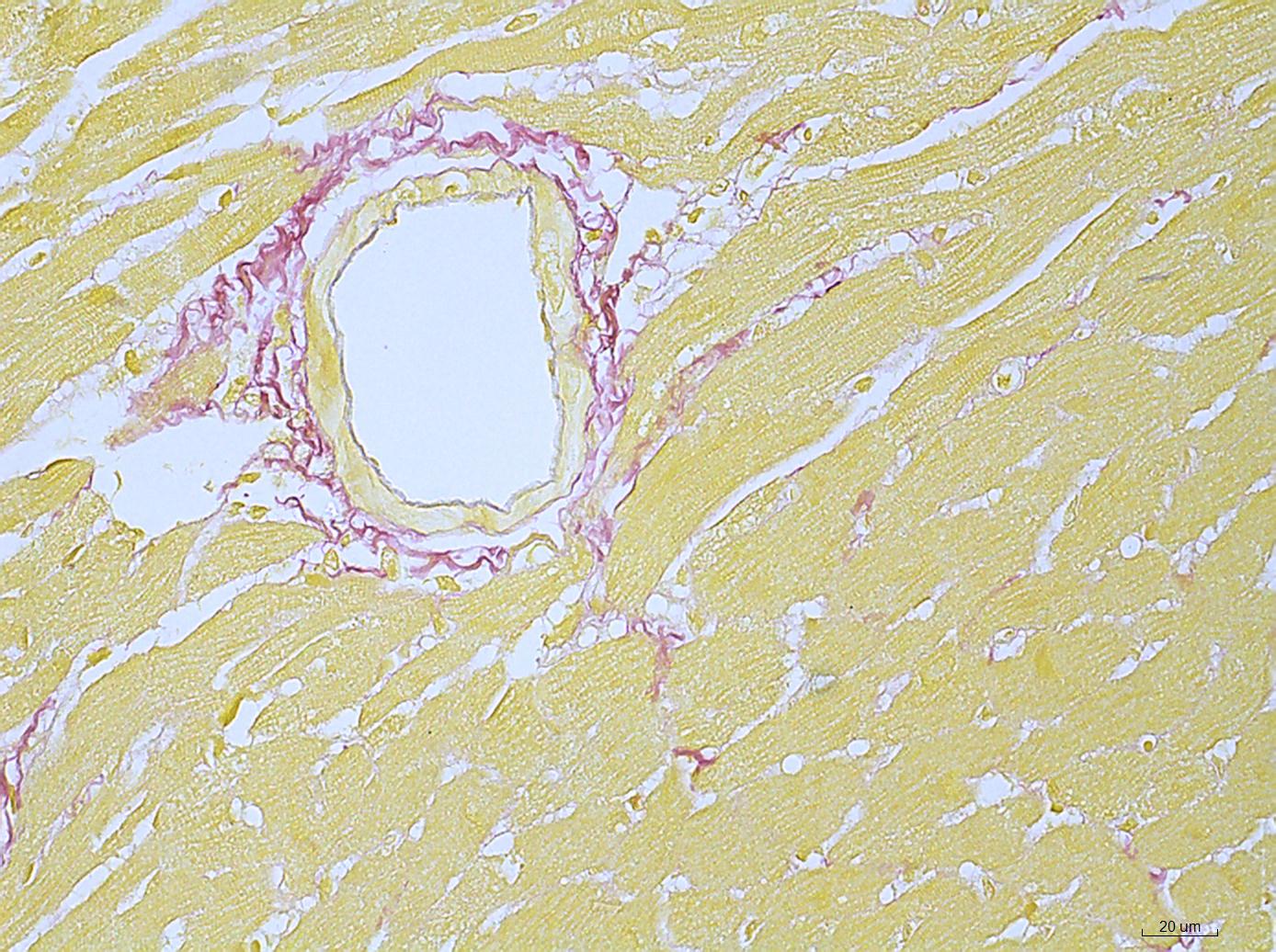

Supplement: Supplementary file 4 — Supporting File 4: advs73796‐sup‐0004‐Data.zip. [file ADVS-13-e21337-s003.zip › advs73796-sup-0004-Data/IHC_Raw_Data_Figures/Figure S8K_RawData_Figures/Sirius red-Ang II-WT-AAV9-TRIM40+PKN1&2-IN-1-40X.jpg]

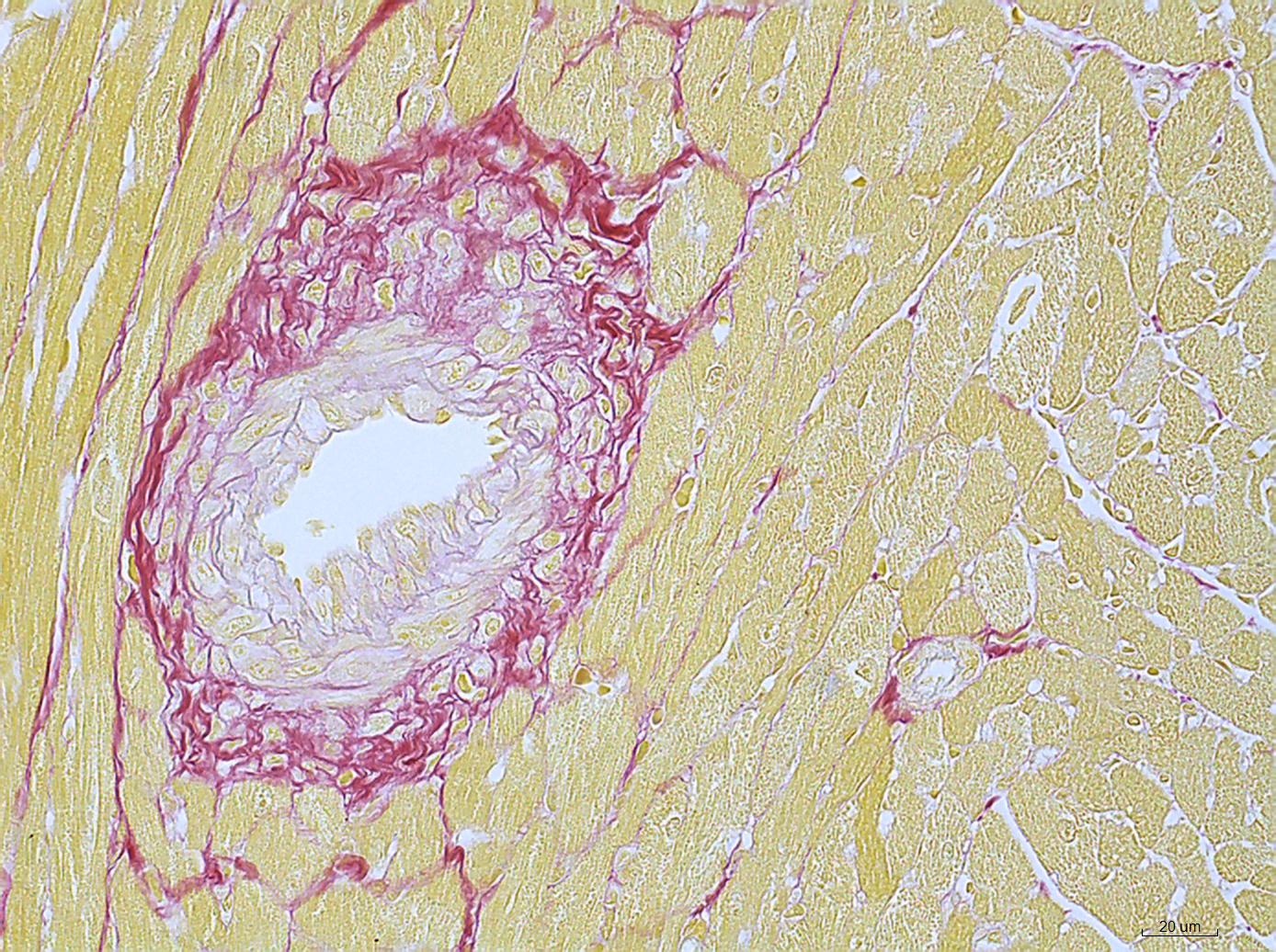

Supplement: Supplementary file 4 — Supporting File 4: advs73796‐sup‐0004‐Data.zip. [file ADVS-13-e21337-s003.zip › advs73796-sup-0004-Data/IHC_Raw_Data_Figures/Figure S8K_RawData_Figures/Sirius red-Ang II-WT-AAV9-TRIM40-40X.jpg]
