# Supplementary figures and images for: TRIM40 Drives Pathological Cardiac Hypertrophy and Heart Failure via Ubiquitination of PKN2 (part 2 of 3)
Source: Adv Sci (Weinh). 2026 Jan 22;13(17):e21337. doi: 10.1002/advs.202521337 (PMC13042792; doi:10.1002/advs.202521337)

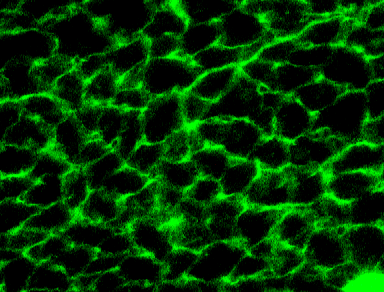

Supplement: Supplementary file 4 — Supporting File 4: advs73796‐sup‐0004‐Data.zip. [file ADVS-13-e21337-s003.zip › advs73796-sup-0004-Data/IF_Raw_Data_Figures/Figure 9H_RawData_Figures/WGA-WT+TAC-AAV-TRIM40+PKN1 or 2-IN-1-40X.tif]

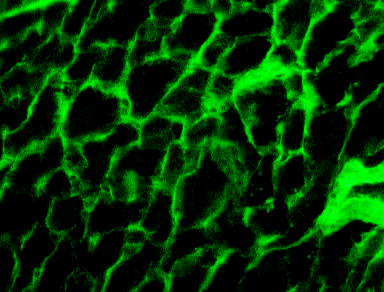

Supplement: Supplementary file 4 — Supporting File 4: advs73796‐sup‐0004‐Data.zip. [file ADVS-13-e21337-s003.zip › advs73796-sup-0004-Data/IF_Raw_Data_Figures/Figure 9H_RawData_Figures/WGA-WT+TAC-AAV-TRIM40-40X.tif]

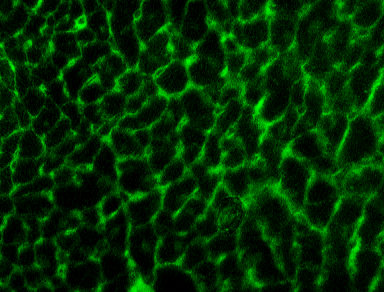

Supplement: Supplementary file 4 — Supporting File 4: advs73796‐sup‐0004‐Data.zip. [file ADVS-13-e21337-s003.zip › advs73796-sup-0004-Data/IF_Raw_Data_Figures/Figure 9H_RawData_Figures/WGA-WT-AAV-NC-40X.tif]

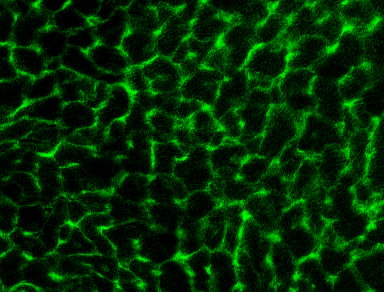

Supplement: Supplementary file 4 — Supporting File 4: advs73796‐sup‐0004‐Data.zip. [file ADVS-13-e21337-s003.zip › advs73796-sup-0004-Data/IF_Raw_Data_Figures/Figure 9H_RawData_Figures/WGA-WT-AAV-TRIM40-40X.tif]

Figure 1D

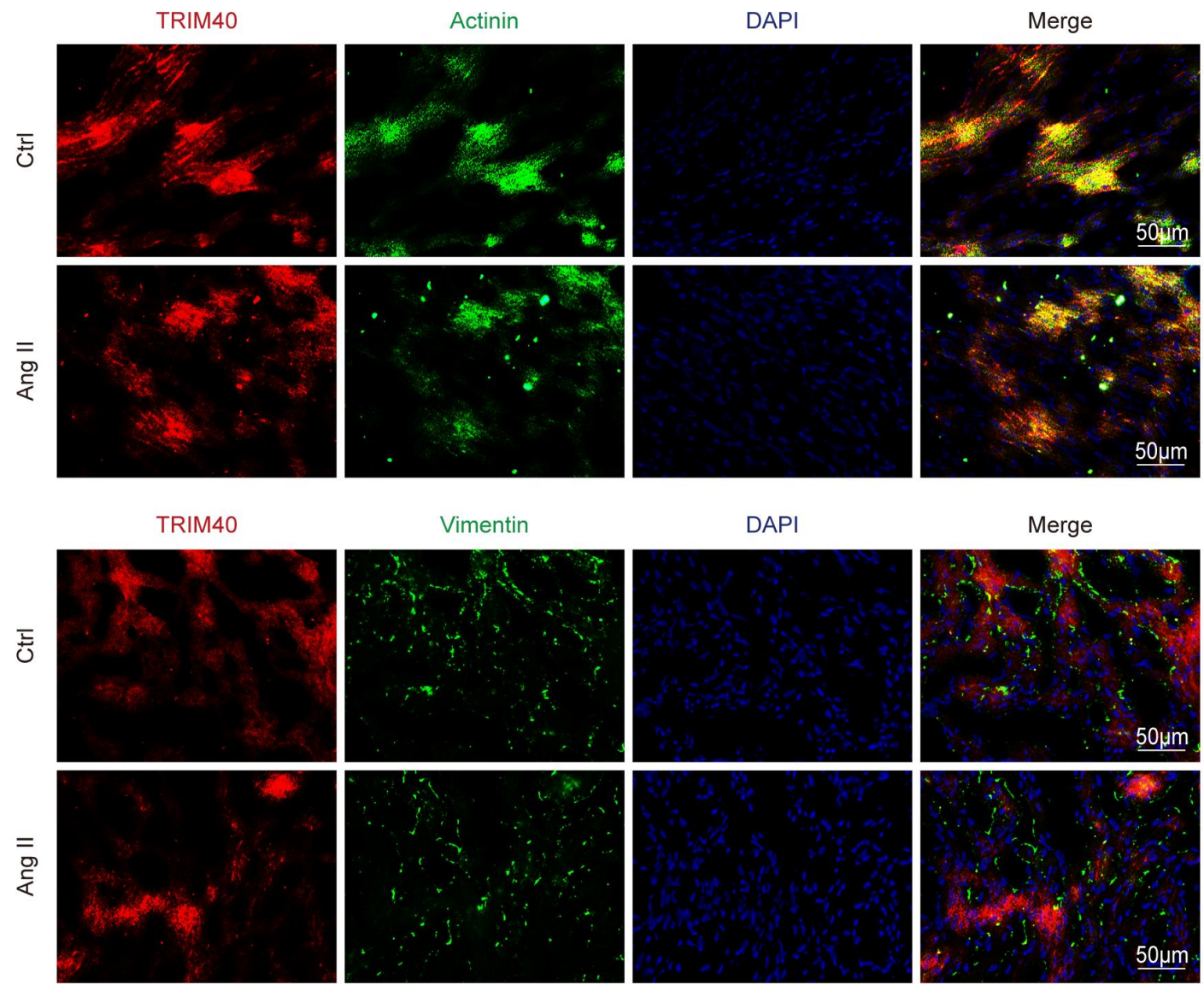

Figure 2J

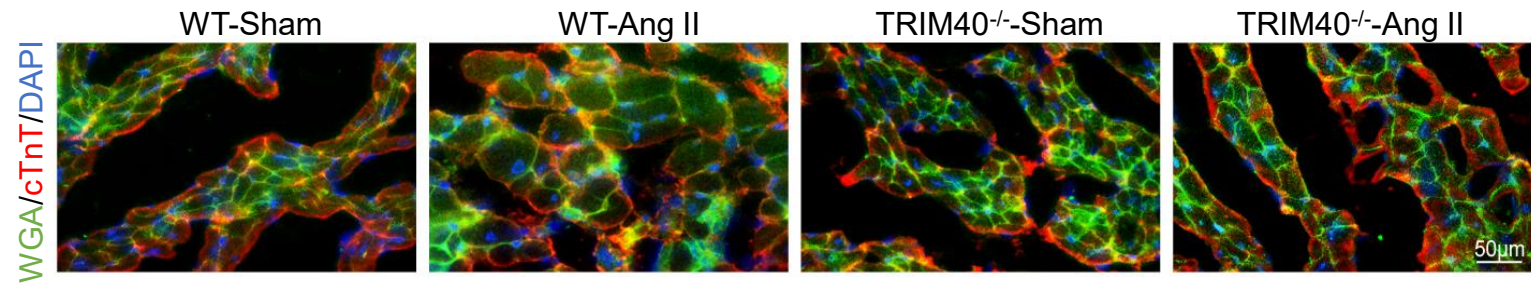

Figure 3H

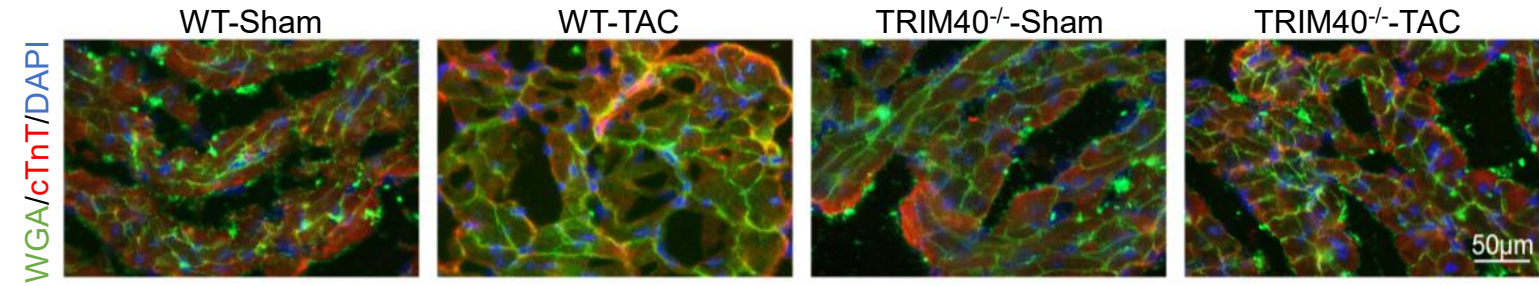

Figure 4K

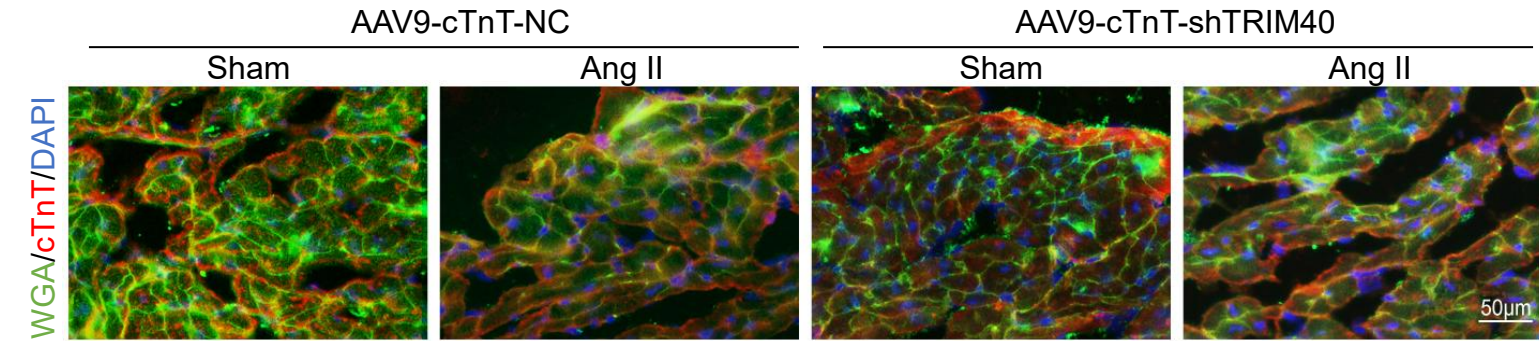

Figure 5A

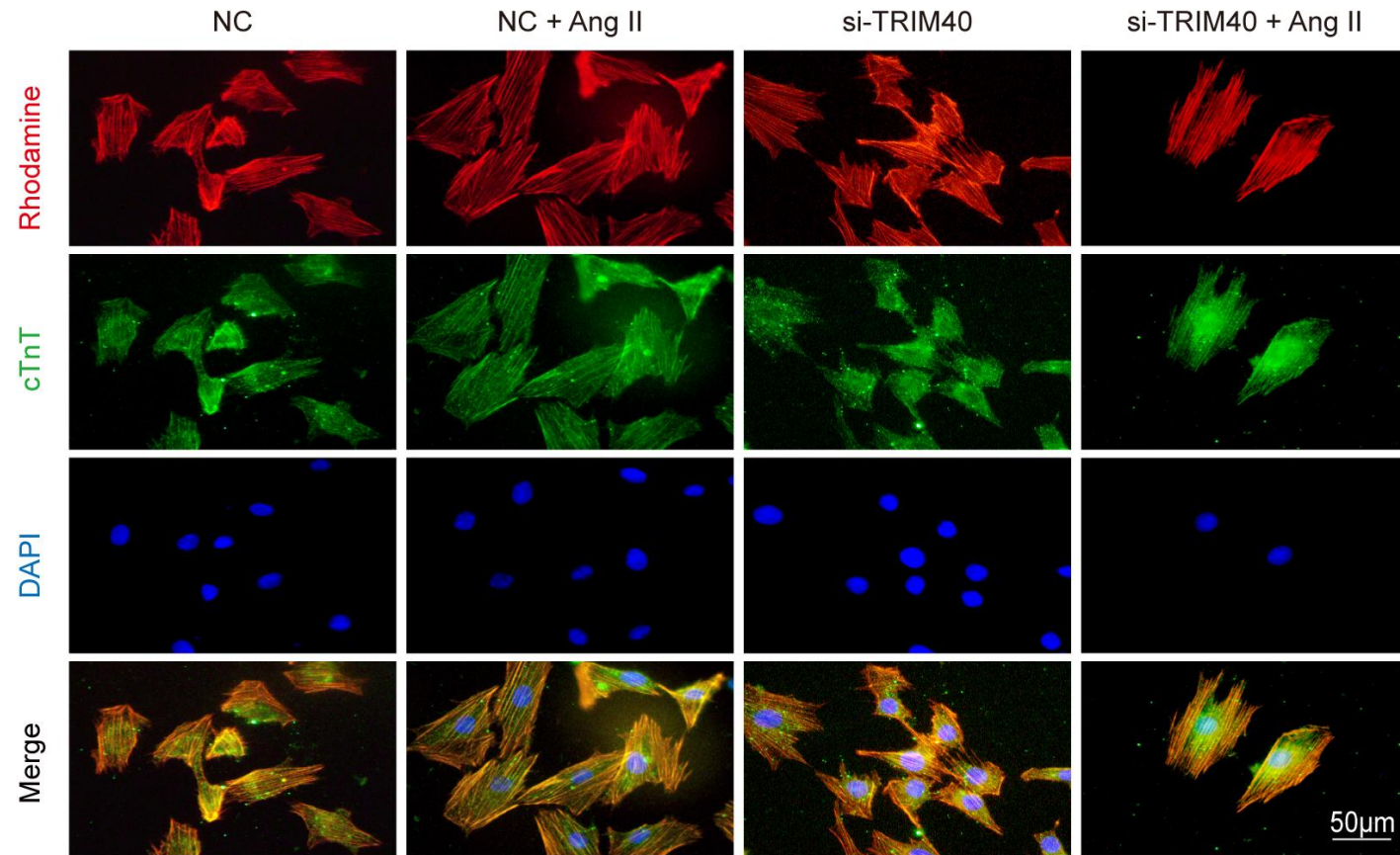

Figure 5F

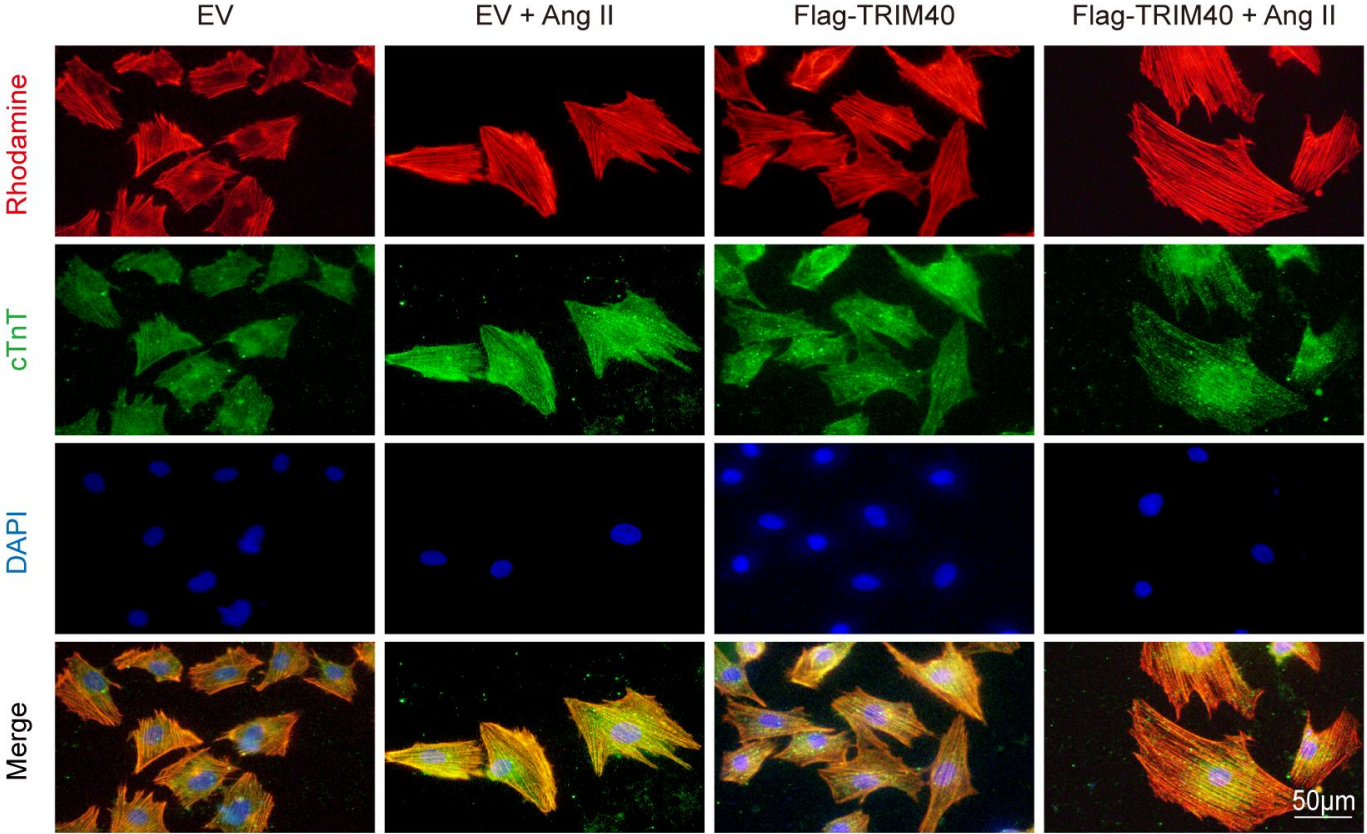

Figure 6L

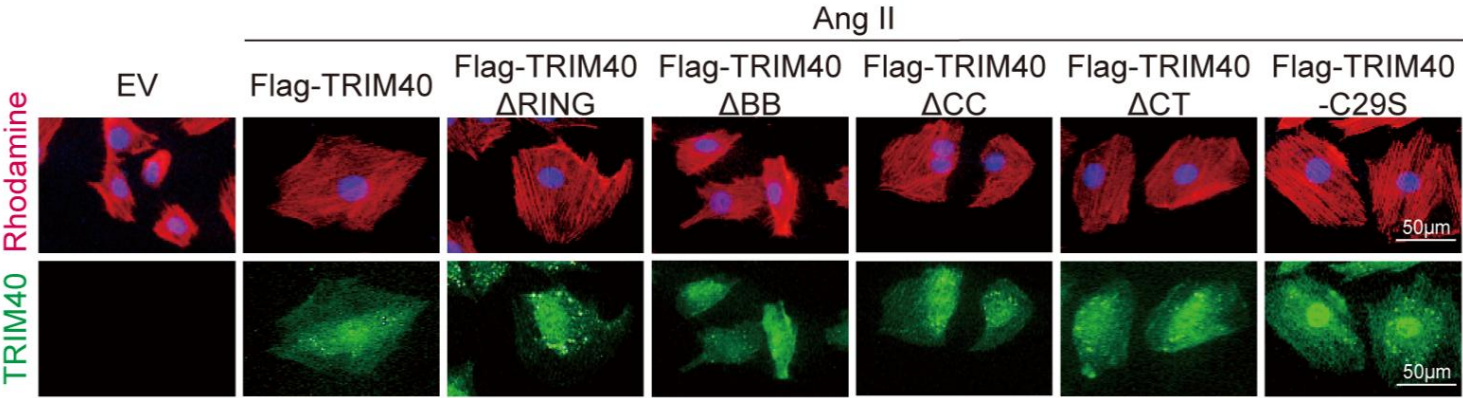

Figure 8J

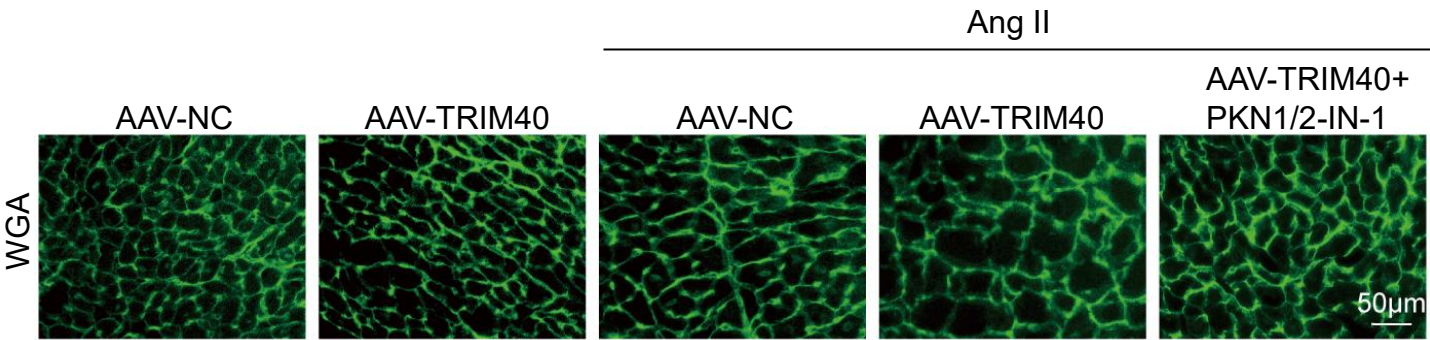

Figure 9H

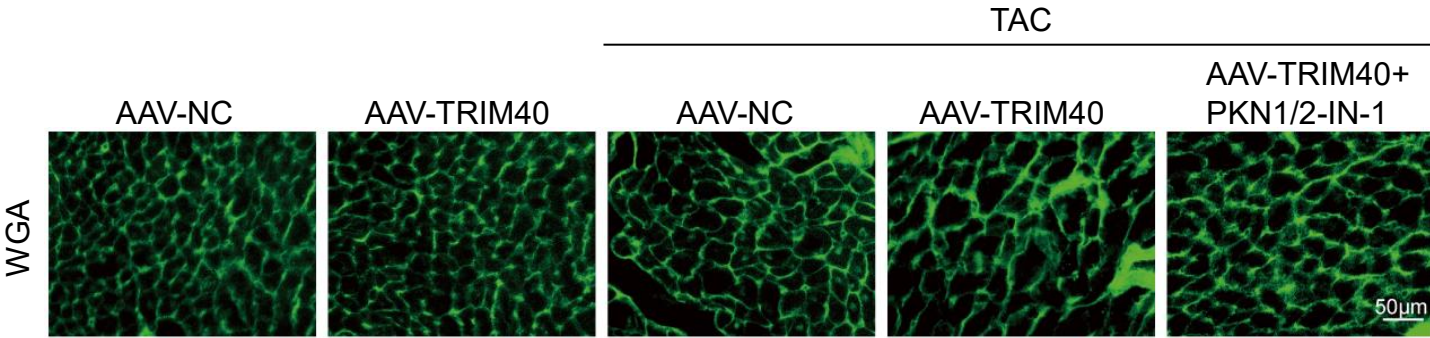

Supplement: Supplementary file 4 — Supporting File 4: advs73796‐sup‐0004‐Data.zip. [file ADVS-13-e21337-s003.zip › advs73796-sup-0004-Data/IF_Raw_Data_Figures/IF_Raw_Data_Figures.pdf]

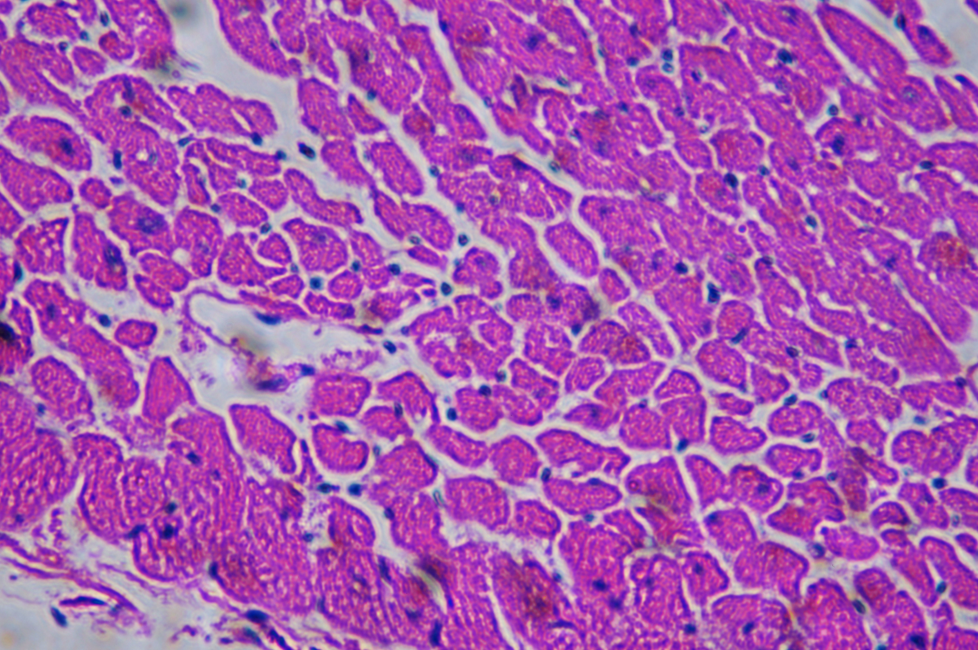

Supplement: Supplementary file 4 — Supporting File 4: advs73796‐sup‐0004‐Data.zip. [file ADVS-13-e21337-s003.zip › advs73796-sup-0004-Data/IHC_Raw_Data_Figures/Figure 2K_RawData_Figures/H&E-TRIM40 knockout-Ang II-40X.tif]

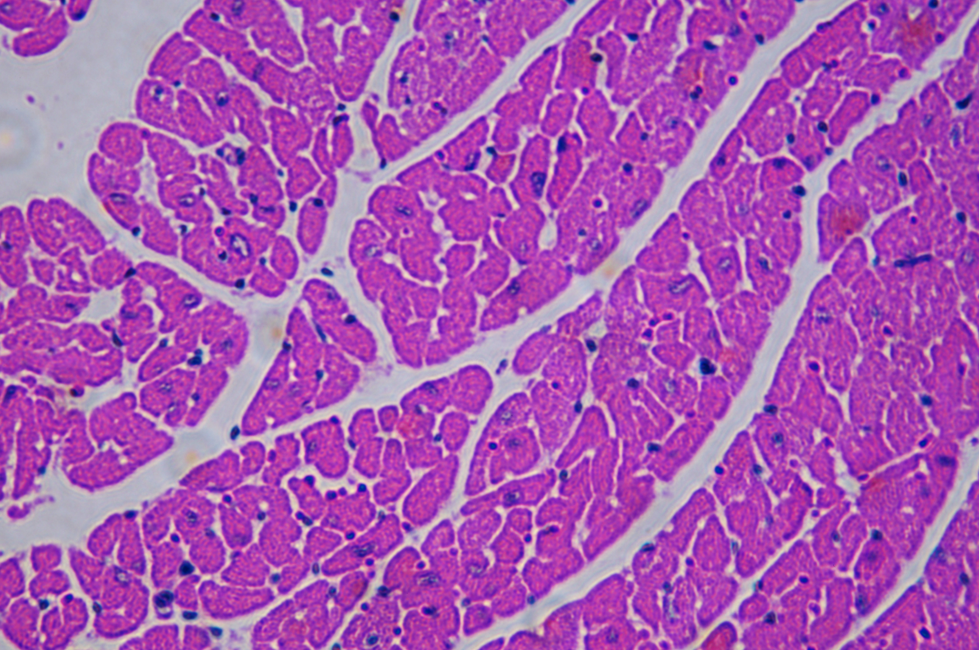

Supplement: Supplementary file 4 — Supporting File 4: advs73796‐sup‐0004‐Data.zip. [file ADVS-13-e21337-s003.zip › advs73796-sup-0004-Data/IHC_Raw_Data_Figures/Figure 2K_RawData_Figures/H&E-TRIM40 knockout-Sham-40X.tif]

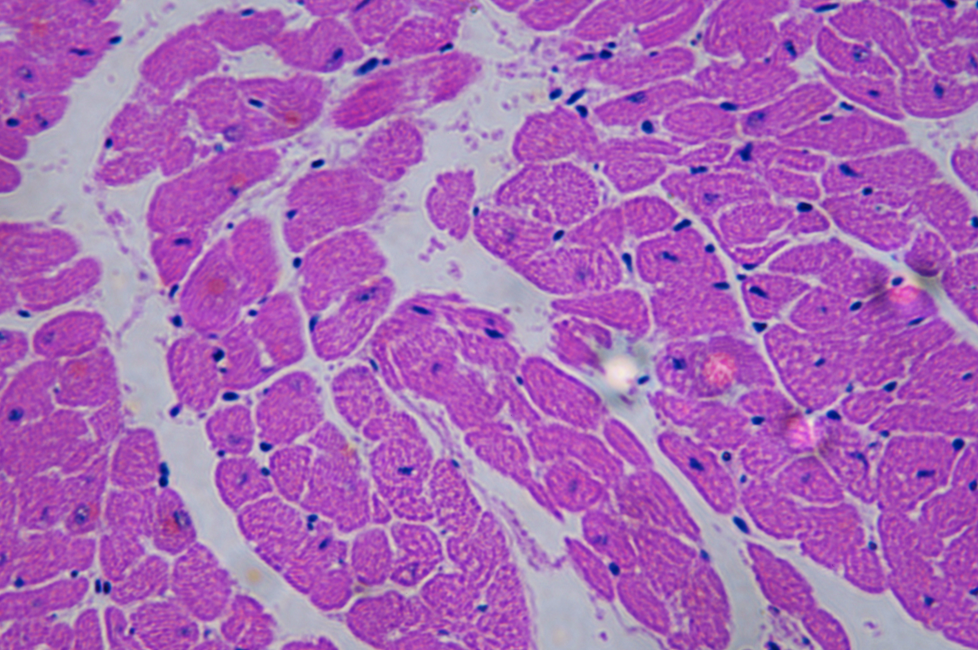

Supplement: Supplementary file 4 — Supporting File 4: advs73796‐sup‐0004‐Data.zip. [file ADVS-13-e21337-s003.zip › advs73796-sup-0004-Data/IHC_Raw_Data_Figures/Figure 2K_RawData_Figures/H&E-WT-Ang II-40X.tif]

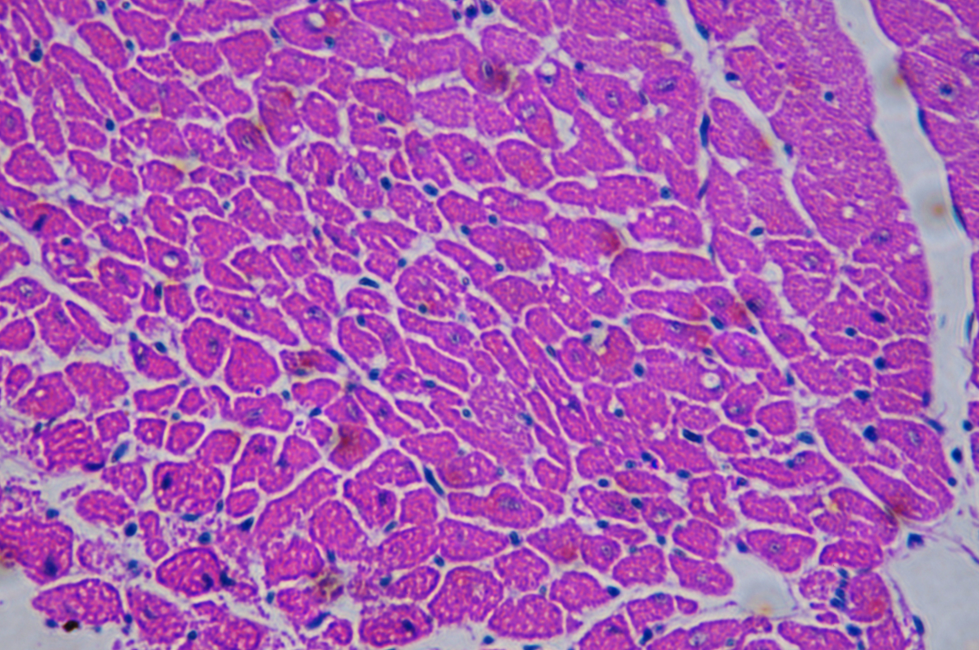

Supplement: Supplementary file 4 — Supporting File 4: advs73796‐sup‐0004‐Data.zip. [file ADVS-13-e21337-s003.zip › advs73796-sup-0004-Data/IHC_Raw_Data_Figures/Figure 2K_RawData_Figures/H&E-WT-Sham-40X.tif]

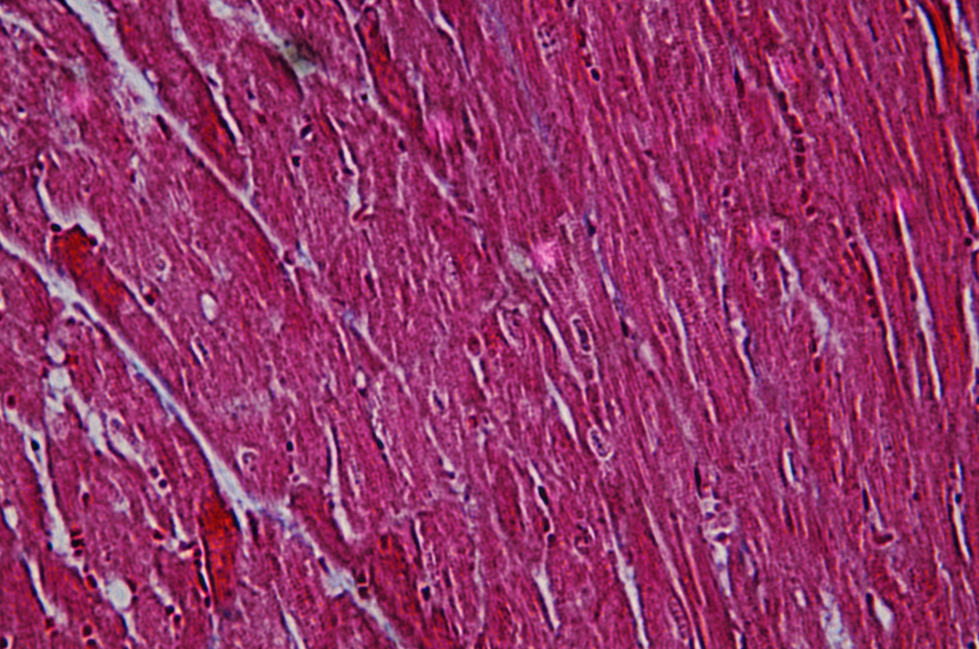

Supplement: Supplementary file 4 — Supporting File 4: advs73796‐sup‐0004‐Data.zip. [file ADVS-13-e21337-s003.zip › advs73796-sup-0004-Data/IHC_Raw_Data_Figures/Figure 2L_RawData_Figures/Masson-TRIM40 knockout-Ang II-40X.tif]

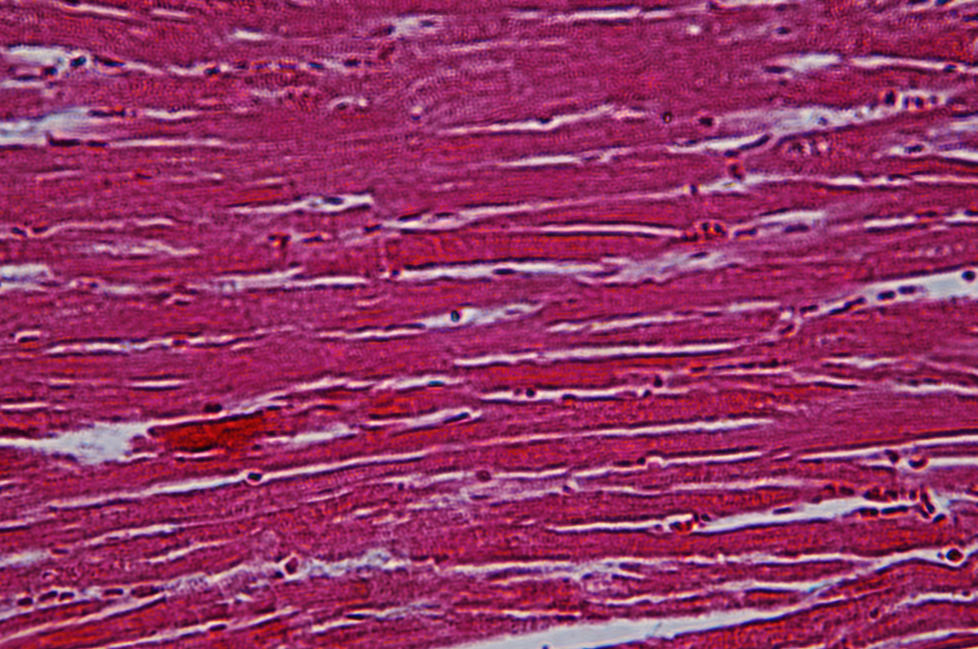

Supplement: Supplementary file 4 — Supporting File 4: advs73796‐sup‐0004‐Data.zip. [file ADVS-13-e21337-s003.zip › advs73796-sup-0004-Data/IHC_Raw_Data_Figures/Figure 2L_RawData_Figures/Masson-TRIM40 knockout-Sham-40X.tif]

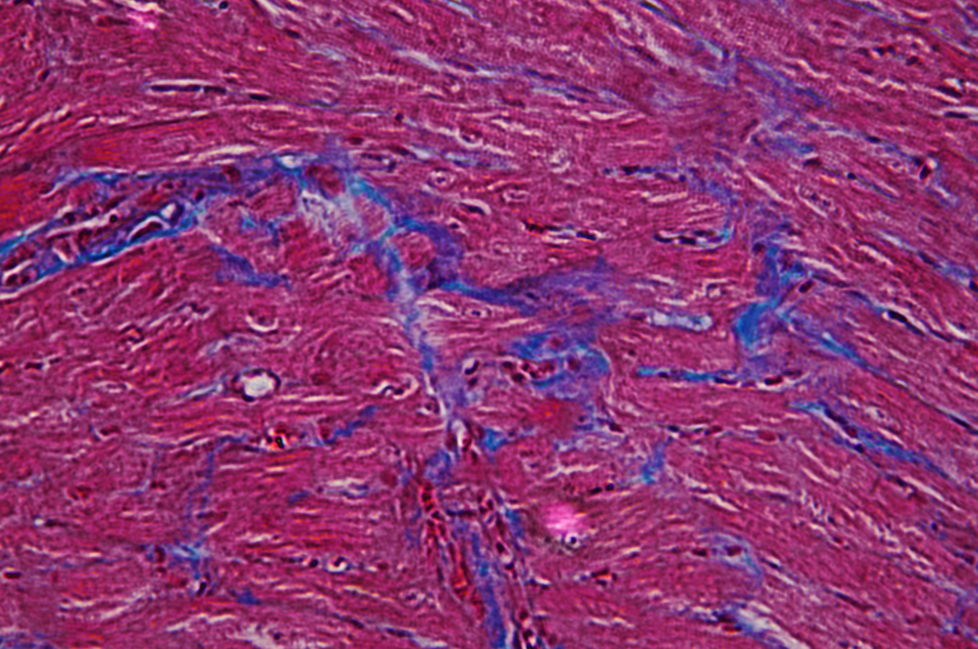

Supplement: Supplementary file 4 — Supporting File 4: advs73796‐sup‐0004‐Data.zip. [file ADVS-13-e21337-s003.zip › advs73796-sup-0004-Data/IHC_Raw_Data_Figures/Figure 2L_RawData_Figures/Masson-WT-Ang II-40X.tif]

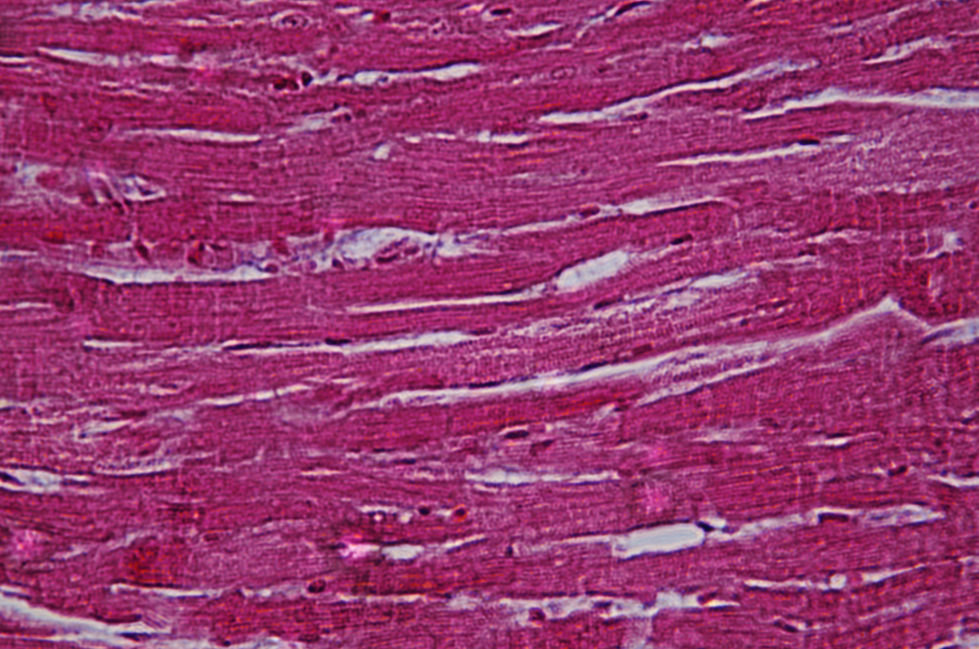

Supplement: Supplementary file 4 — Supporting File 4: advs73796‐sup‐0004‐Data.zip. [file ADVS-13-e21337-s003.zip › advs73796-sup-0004-Data/IHC_Raw_Data_Figures/Figure 2L_RawData_Figures/Masson-WT-Sham-40X.tif]

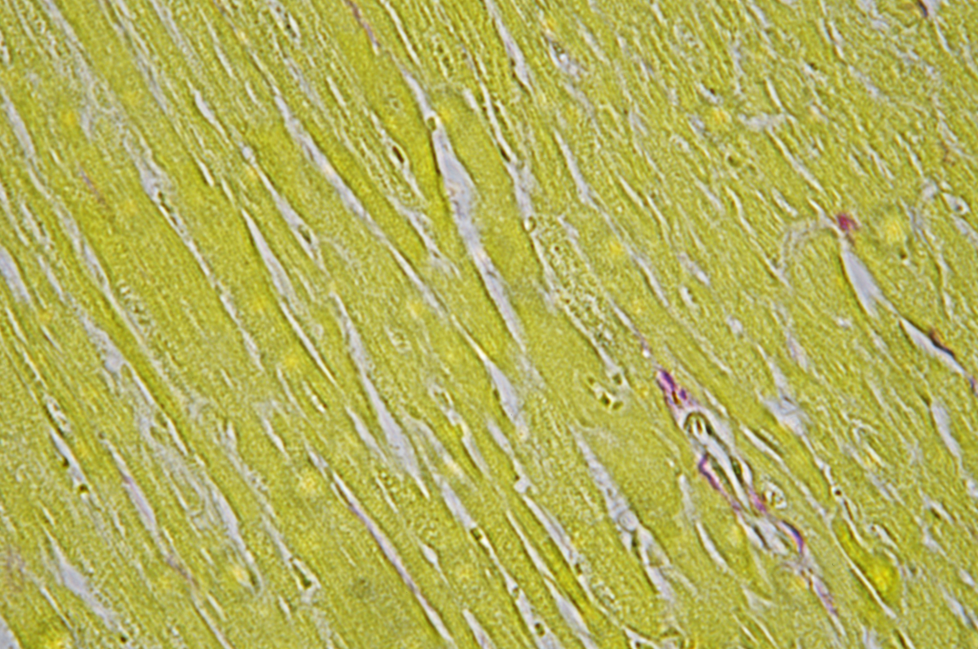

Supplement: Supplementary file 4 — Supporting File 4: advs73796‐sup‐0004‐Data.zip. [file ADVS-13-e21337-s003.zip › advs73796-sup-0004-Data/IHC_Raw_Data_Figures/Figure 2M_RawData_Figures/Sirius red-TRIM40 knockout-Ang II-40X.tif]

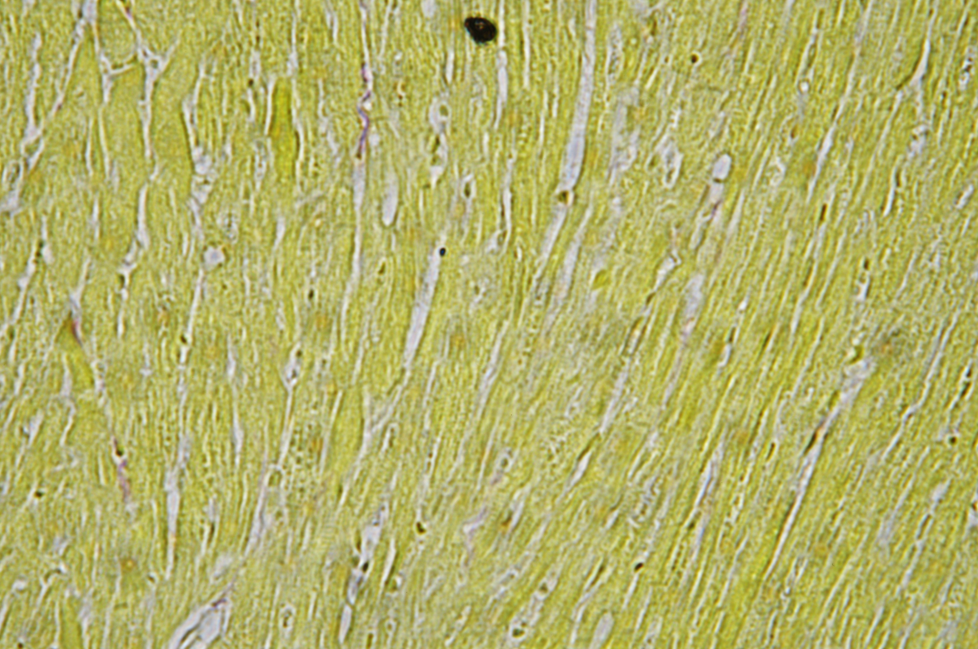

Supplement: Supplementary file 4 — Supporting File 4: advs73796‐sup‐0004‐Data.zip. [file ADVS-13-e21337-s003.zip › advs73796-sup-0004-Data/IHC_Raw_Data_Figures/Figure 2M_RawData_Figures/Sirius red-TRIM40 knockout-Sham-40X.tif]

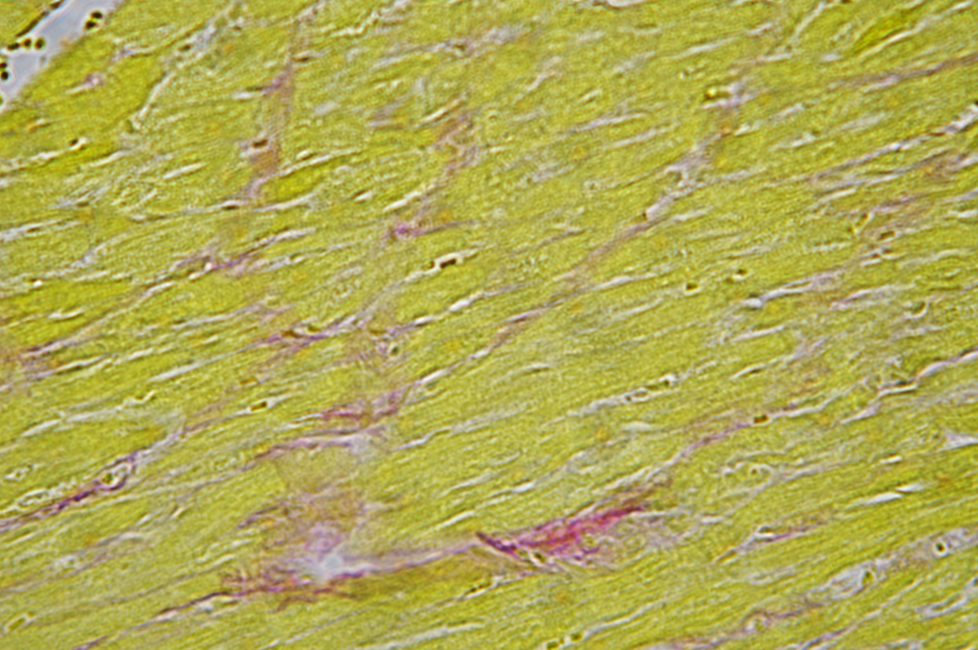

Supplement: Supplementary file 4 — Supporting File 4: advs73796‐sup‐0004‐Data.zip. [file ADVS-13-e21337-s003.zip › advs73796-sup-0004-Data/IHC_Raw_Data_Figures/Figure 2M_RawData_Figures/Sirius red-WT-Ang II-40X.tif]

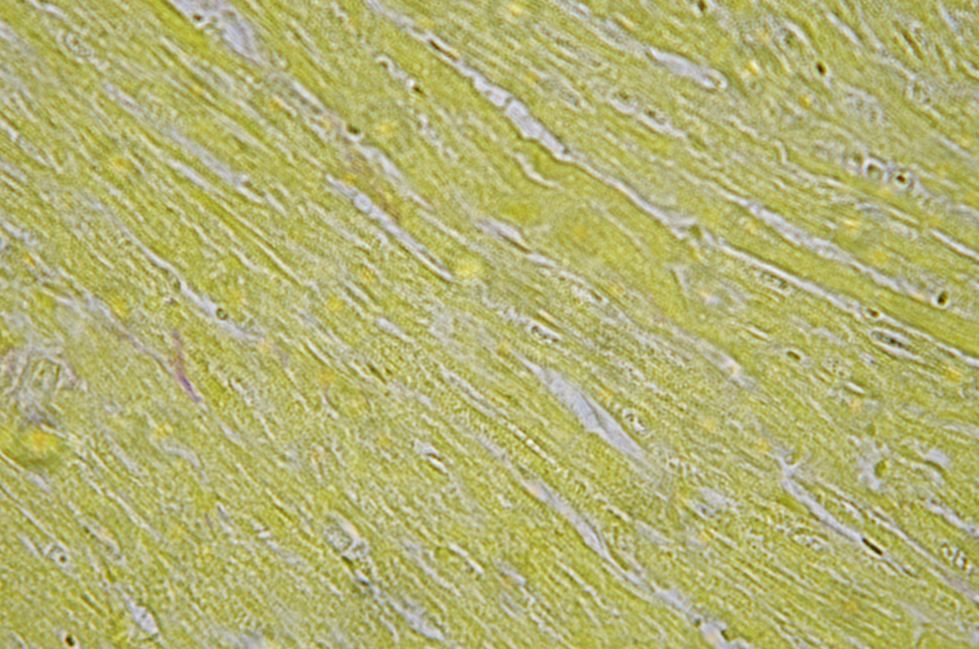

Supplement: Supplementary file 4 — Supporting File 4: advs73796‐sup‐0004‐Data.zip. [file ADVS-13-e21337-s003.zip › advs73796-sup-0004-Data/IHC_Raw_Data_Figures/Figure 2M_RawData_Figures/Sirius red-WT-Sham-40X.tif]

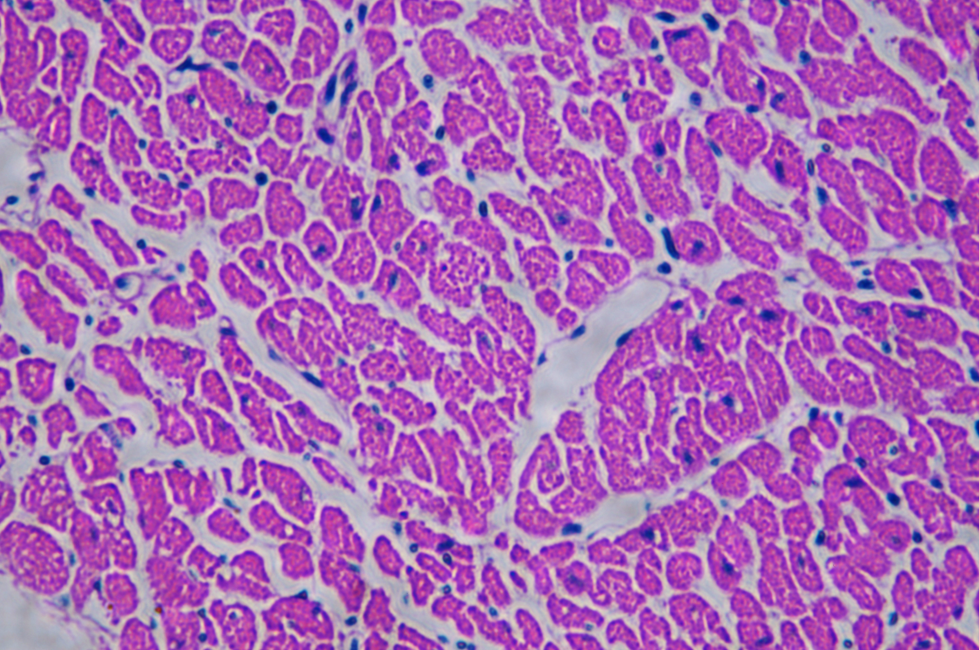

Supplement: Supplementary file 4 — Supporting File 4: advs73796‐sup‐0004‐Data.zip. [file ADVS-13-e21337-s003.zip › advs73796-sup-0004-Data/IHC_Raw_Data_Figures/Figure 3J_RawData_Figures/H&E-TRIM40 knockout-Sham-40X.tif]

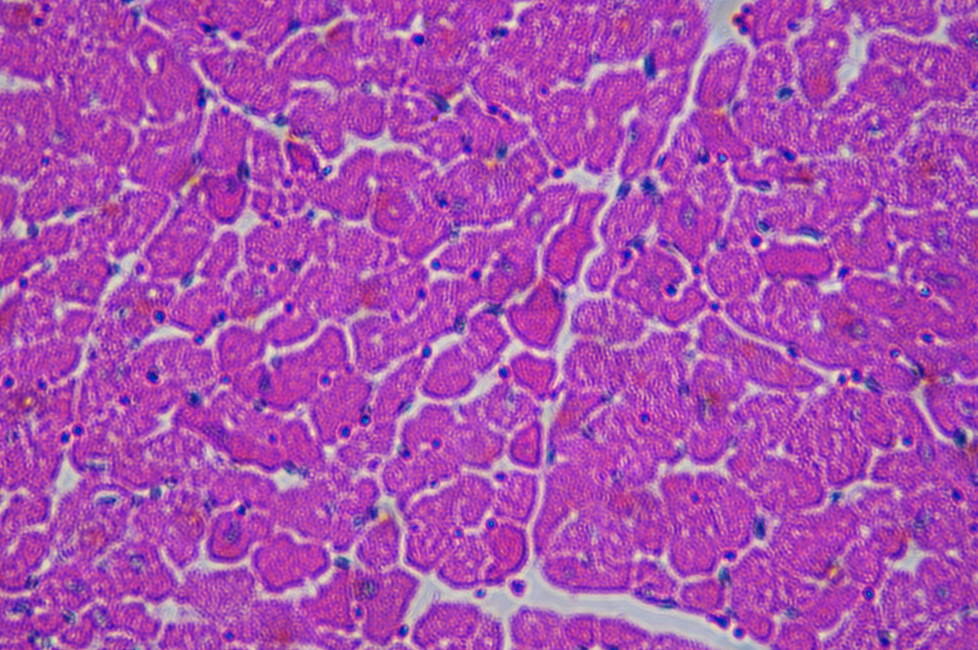

Supplement: Supplementary file 4 — Supporting File 4: advs73796‐sup‐0004‐Data.zip. [file ADVS-13-e21337-s003.zip › advs73796-sup-0004-Data/IHC_Raw_Data_Figures/Figure 3J_RawData_Figures/H&E-TRIM40 knockout-TAC-40X.tif]

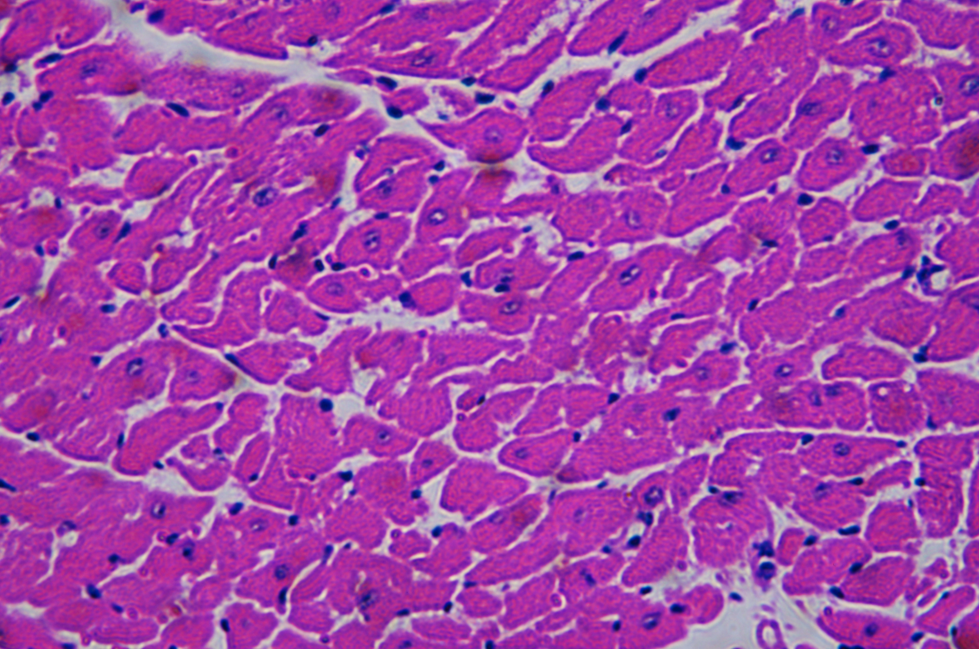

Supplement: Supplementary file 4 — Supporting File 4: advs73796‐sup‐0004‐Data.zip. [file ADVS-13-e21337-s003.zip › advs73796-sup-0004-Data/IHC_Raw_Data_Figures/Figure 3J_RawData_Figures/H&E-WT-Sham-40X.tif]

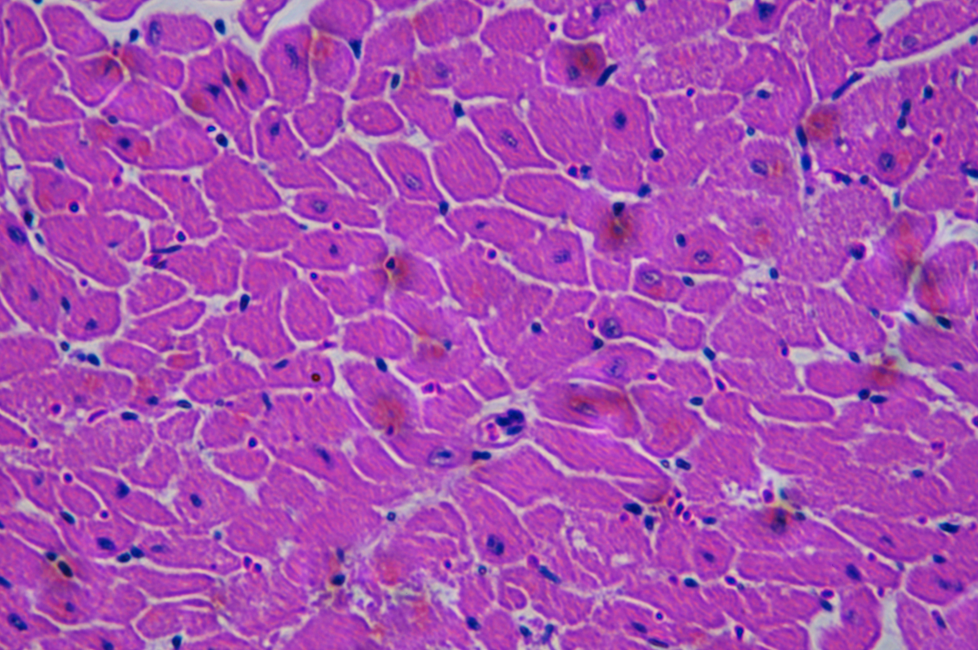

Supplement: Supplementary file 4 — Supporting File 4: advs73796‐sup‐0004‐Data.zip. [file ADVS-13-e21337-s003.zip › advs73796-sup-0004-Data/IHC_Raw_Data_Figures/Figure 3J_RawData_Figures/H&E-WT-TAC-40X.tif]

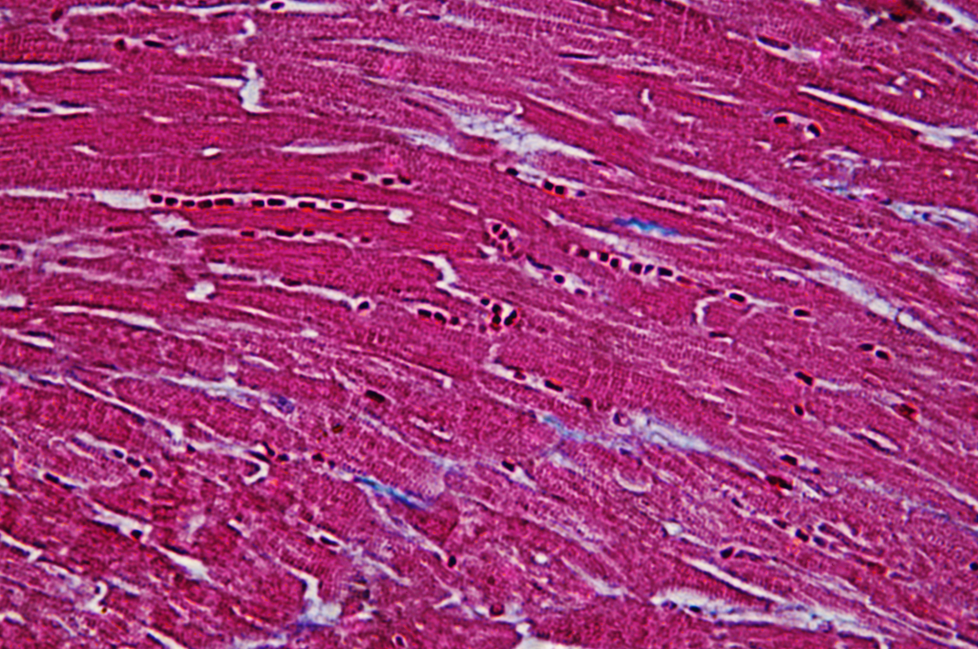

Supplement: Supplementary file 4 — Supporting File 4: advs73796‐sup‐0004‐Data.zip. [file ADVS-13-e21337-s003.zip › advs73796-sup-0004-Data/IHC_Raw_Data_Figures/Figure 3K_RawData_Figures/Masson-TRIM40 knockout-Sham-40X.tif]

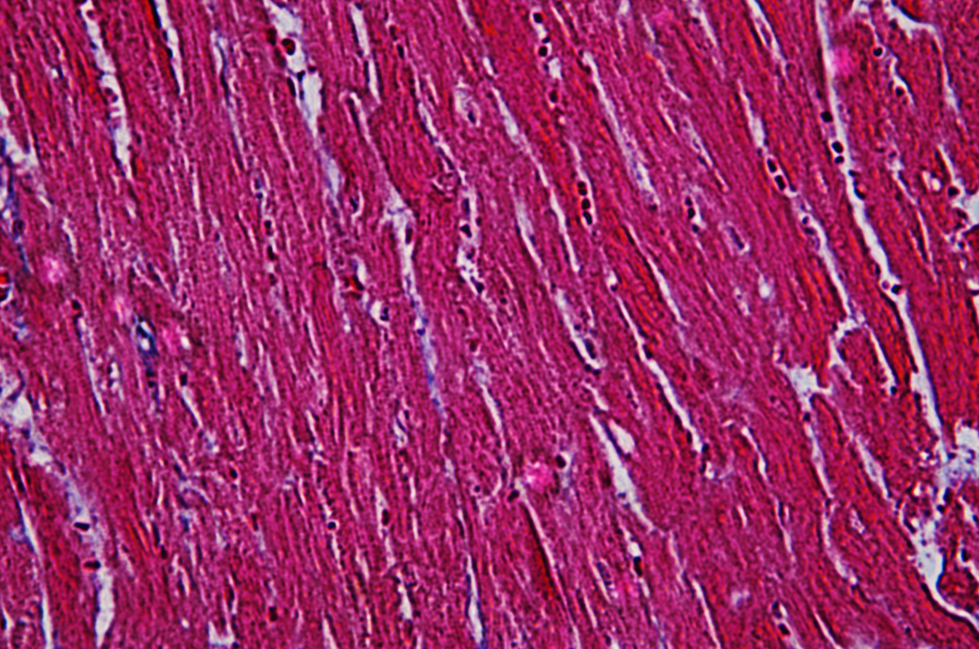

Supplement: Supplementary file 4 — Supporting File 4: advs73796‐sup‐0004‐Data.zip. [file ADVS-13-e21337-s003.zip › advs73796-sup-0004-Data/IHC_Raw_Data_Figures/Figure 3K_RawData_Figures/Masson-TRIM40 knockout-TAC-40X.tif]

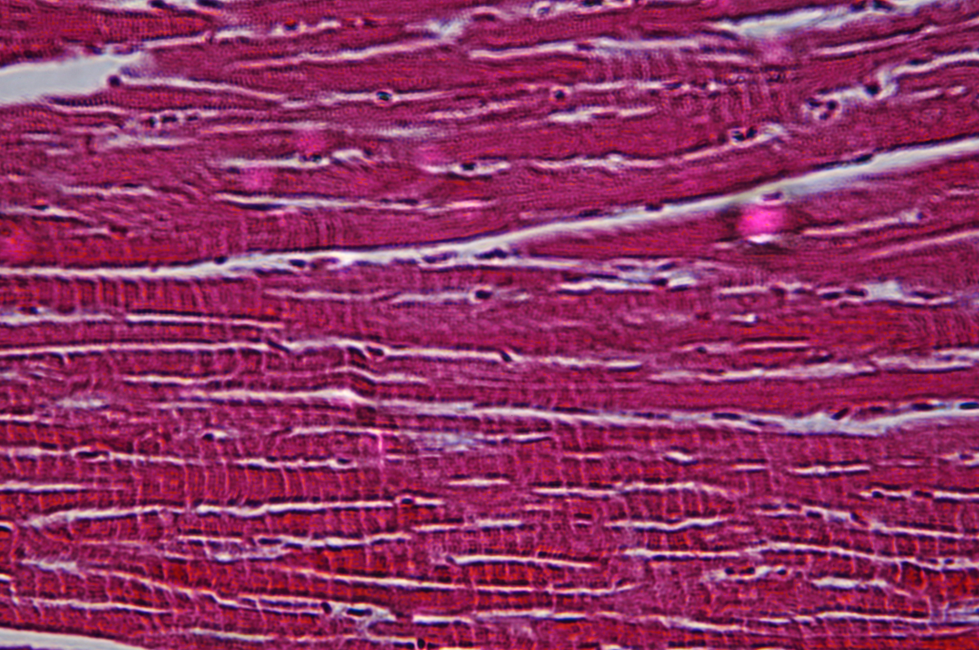

Supplement: Supplementary file 4 — Supporting File 4: advs73796‐sup‐0004‐Data.zip. [file ADVS-13-e21337-s003.zip › advs73796-sup-0004-Data/IHC_Raw_Data_Figures/Figure 3K_RawData_Figures/Masson-WT-Sham-40X.tif]

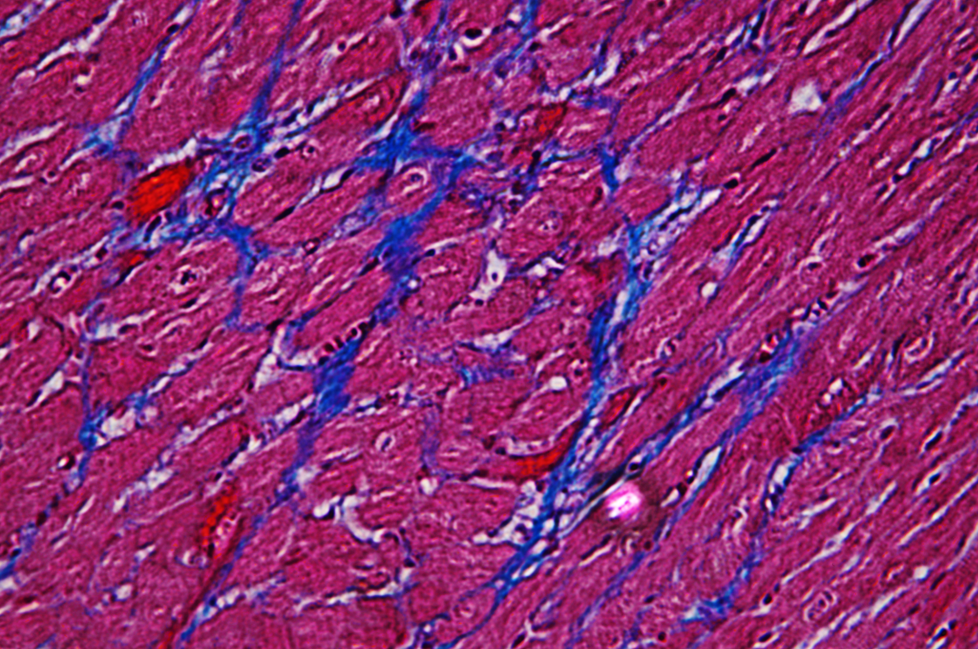

Supplement: Supplementary file 4 — Supporting File 4: advs73796‐sup‐0004‐Data.zip. [file ADVS-13-e21337-s003.zip › advs73796-sup-0004-Data/IHC_Raw_Data_Figures/Figure 3K_RawData_Figures/Masson-WT-TAC-40X.tif]

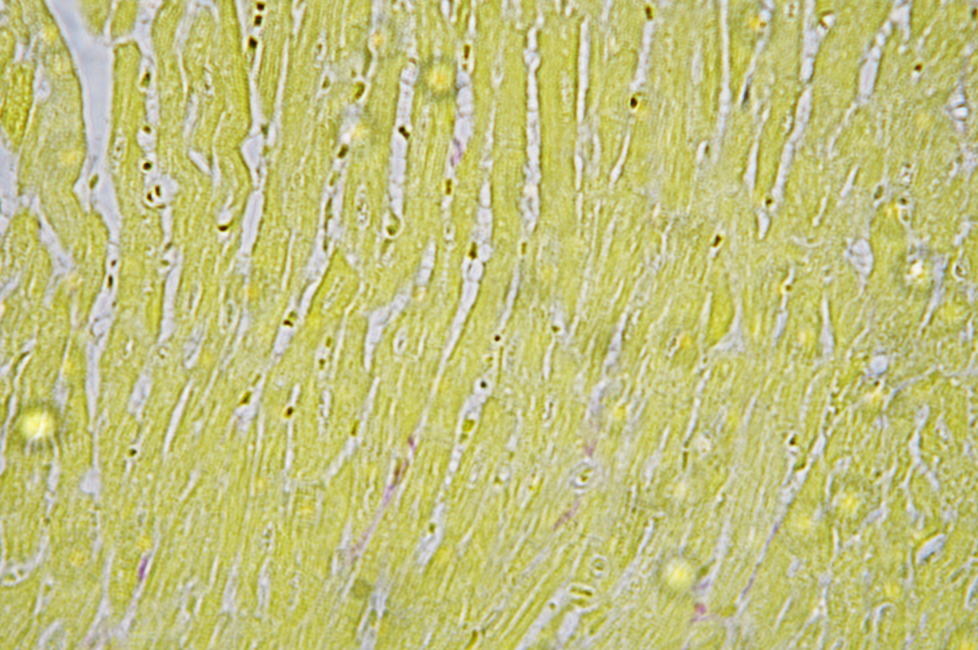

Supplement: Supplementary file 4 — Supporting File 4: advs73796‐sup‐0004‐Data.zip. [file ADVS-13-e21337-s003.zip › advs73796-sup-0004-Data/IHC_Raw_Data_Figures/Figure 3L_RawData_Figures/Sirius red-TRIM40 knockout-Sham-40X.tif]

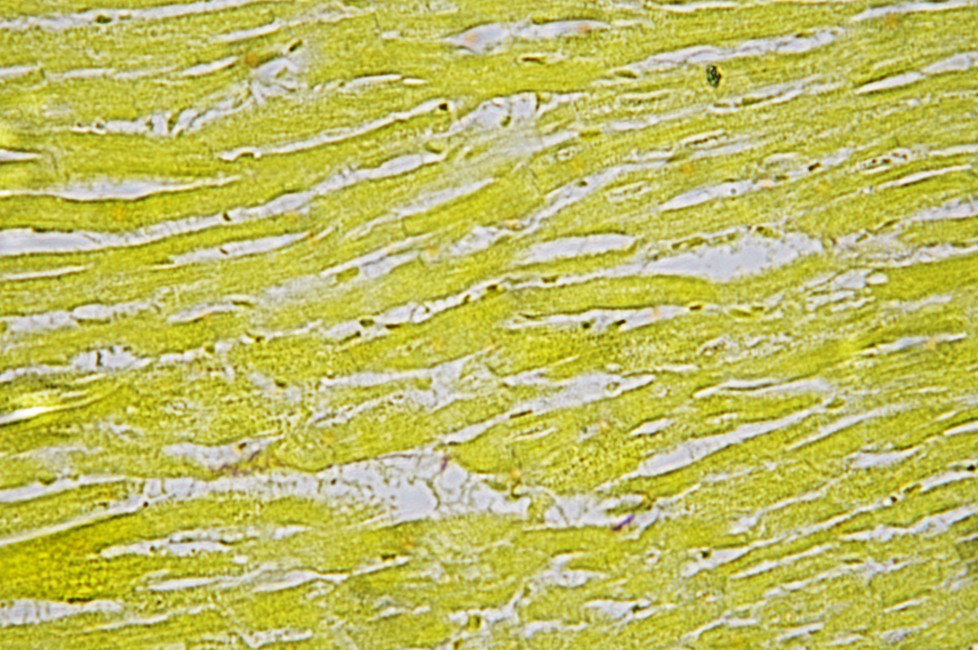

Supplement: Supplementary file 4 — Supporting File 4: advs73796‐sup‐0004‐Data.zip. [file ADVS-13-e21337-s003.zip › advs73796-sup-0004-Data/IHC_Raw_Data_Figures/Figure 3L_RawData_Figures/Sirius red-TRIM40 knockout-TAC-40X.tif]

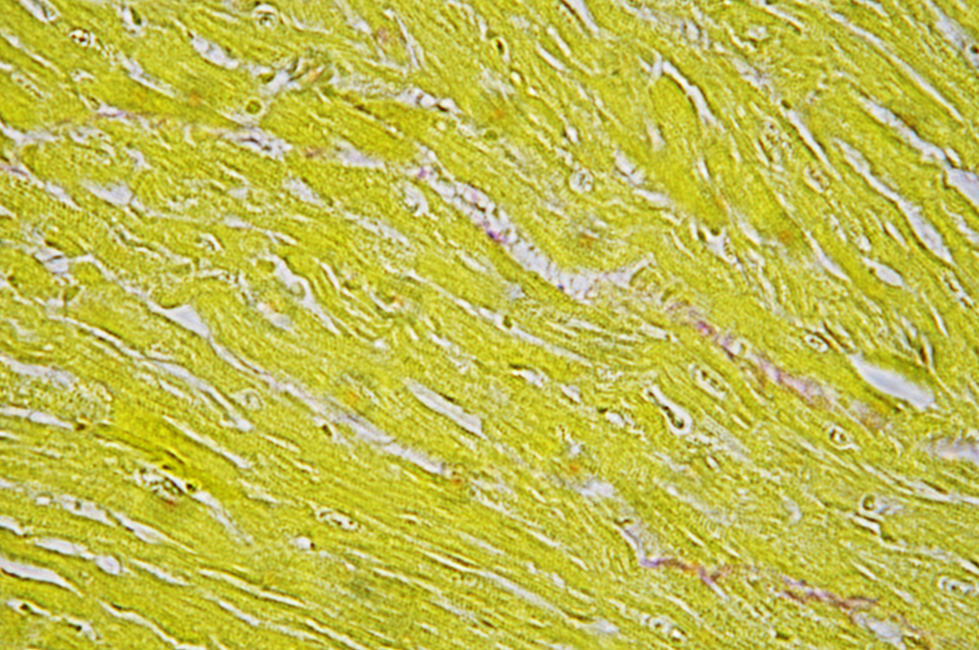

Supplement: Supplementary file 4 — Supporting File 4: advs73796‐sup‐0004‐Data.zip. [file ADVS-13-e21337-s003.zip › advs73796-sup-0004-Data/IHC_Raw_Data_Figures/Figure 3L_RawData_Figures/Sirius red-WT-Sham-40X.tif]

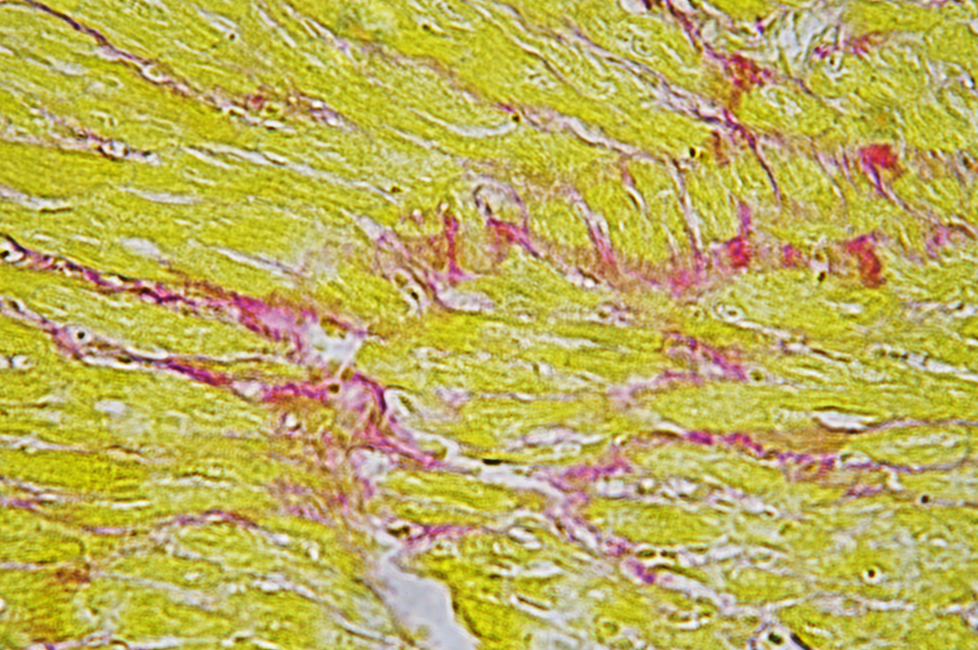

Supplement: Supplementary file 4 — Supporting File 4: advs73796‐sup‐0004‐Data.zip. [file ADVS-13-e21337-s003.zip › advs73796-sup-0004-Data/IHC_Raw_Data_Figures/Figure 3L_RawData_Figures/Sirius red-WT-TAC-40X.tif]

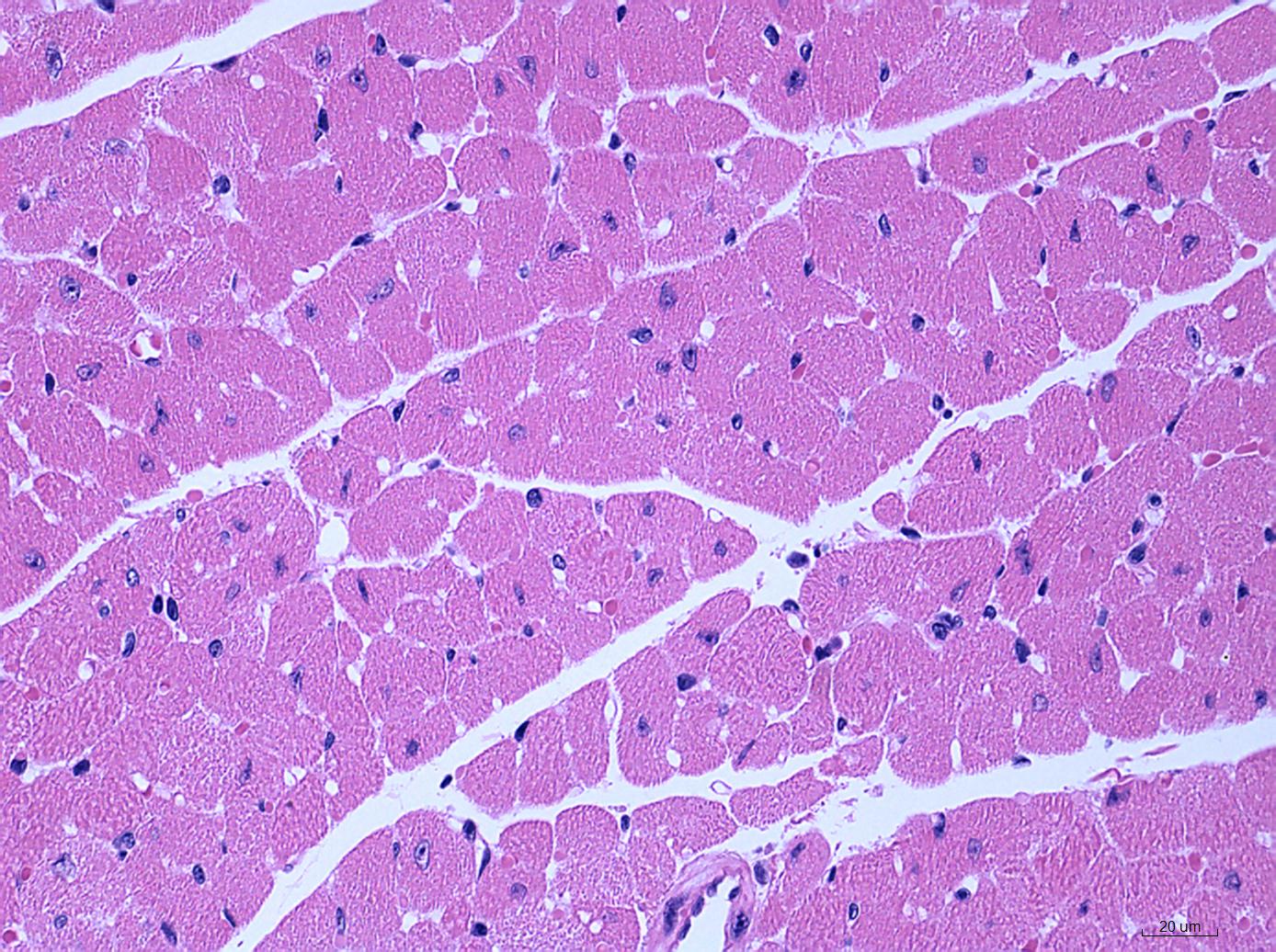

Supplement: Supplementary file 4 — Supporting File 4: advs73796‐sup‐0004‐Data.zip. [file ADVS-13-e21337-s003.zip › advs73796-sup-0004-Data/IHC_Raw_Data_Figures/Figure 4M_RawData_Figures/H&E-AAV9-cTnT-NC-Ang II-40X.jpg]

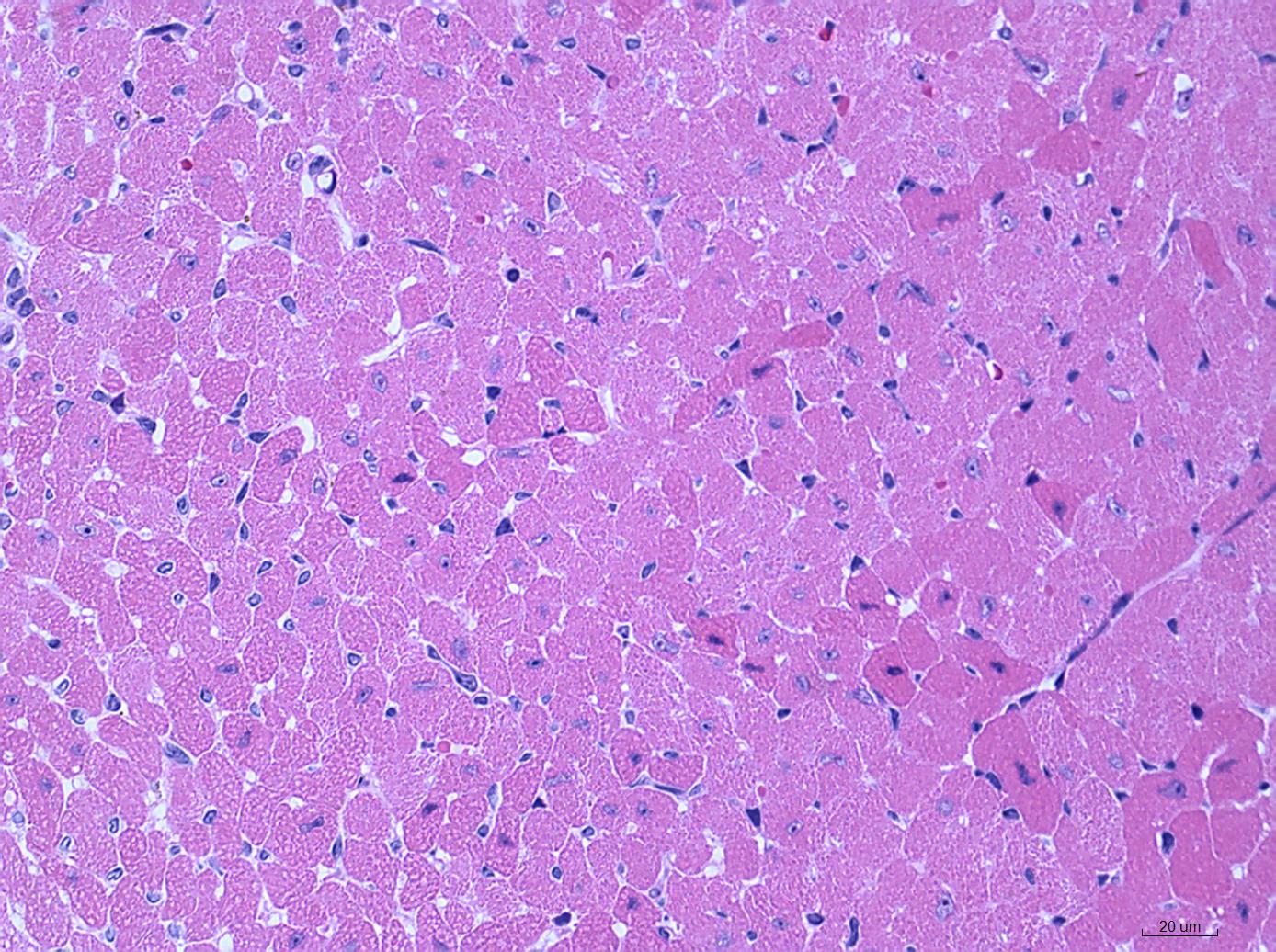

Supplement: Supplementary file 4 — Supporting File 4: advs73796‐sup‐0004‐Data.zip. [file ADVS-13-e21337-s003.zip › advs73796-sup-0004-Data/IHC_Raw_Data_Figures/Figure 4M_RawData_Figures/H&E-AAV9-cTnT-NC-Sham-40X.jpg]

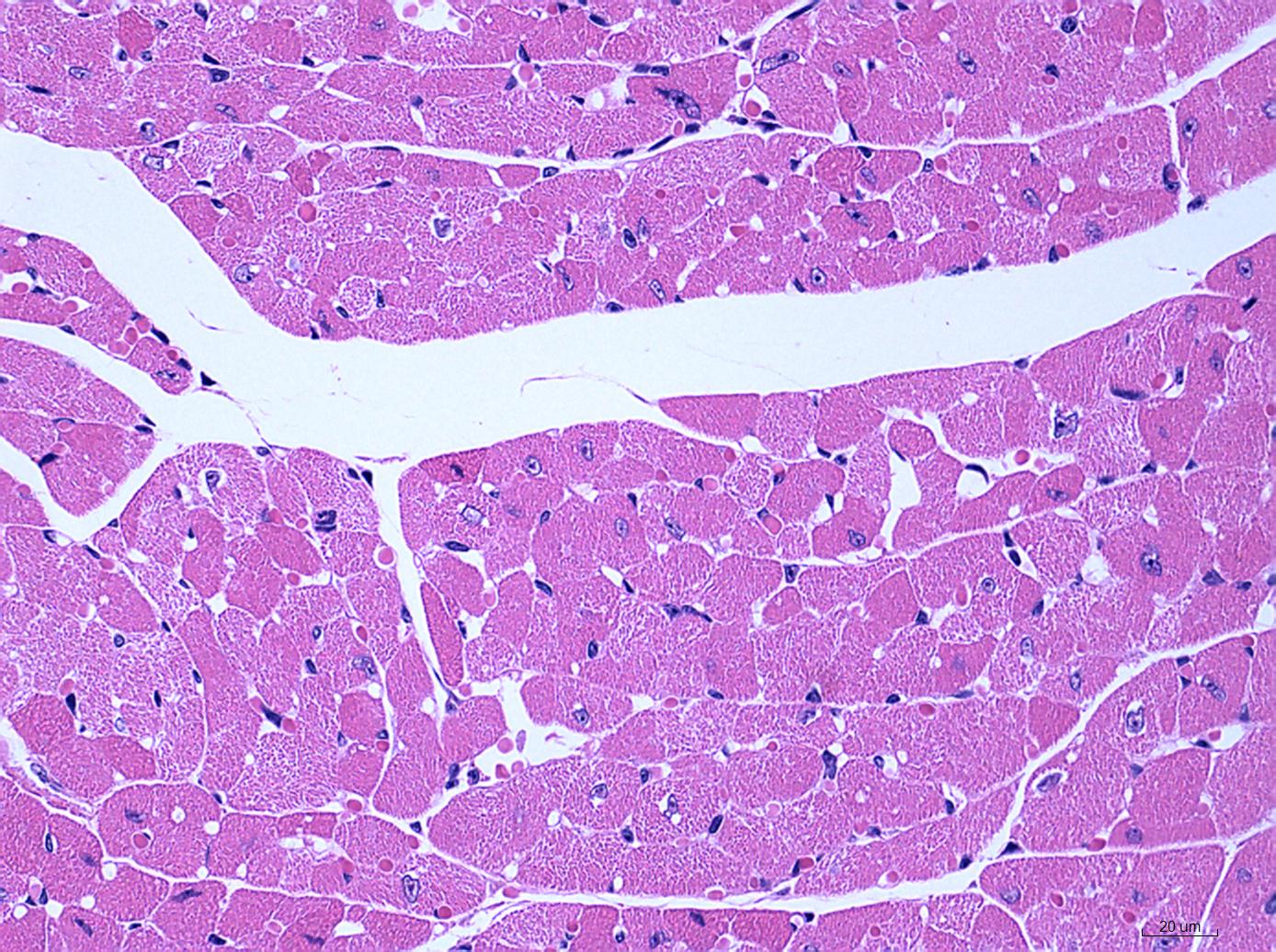

Supplement: Supplementary file 4 — Supporting File 4: advs73796‐sup‐0004‐Data.zip. [file ADVS-13-e21337-s003.zip › advs73796-sup-0004-Data/IHC_Raw_Data_Figures/Figure 4M_RawData_Figures/H&E-AAV9-cTnT-shTRIM40-Ang II-40X.jpg]

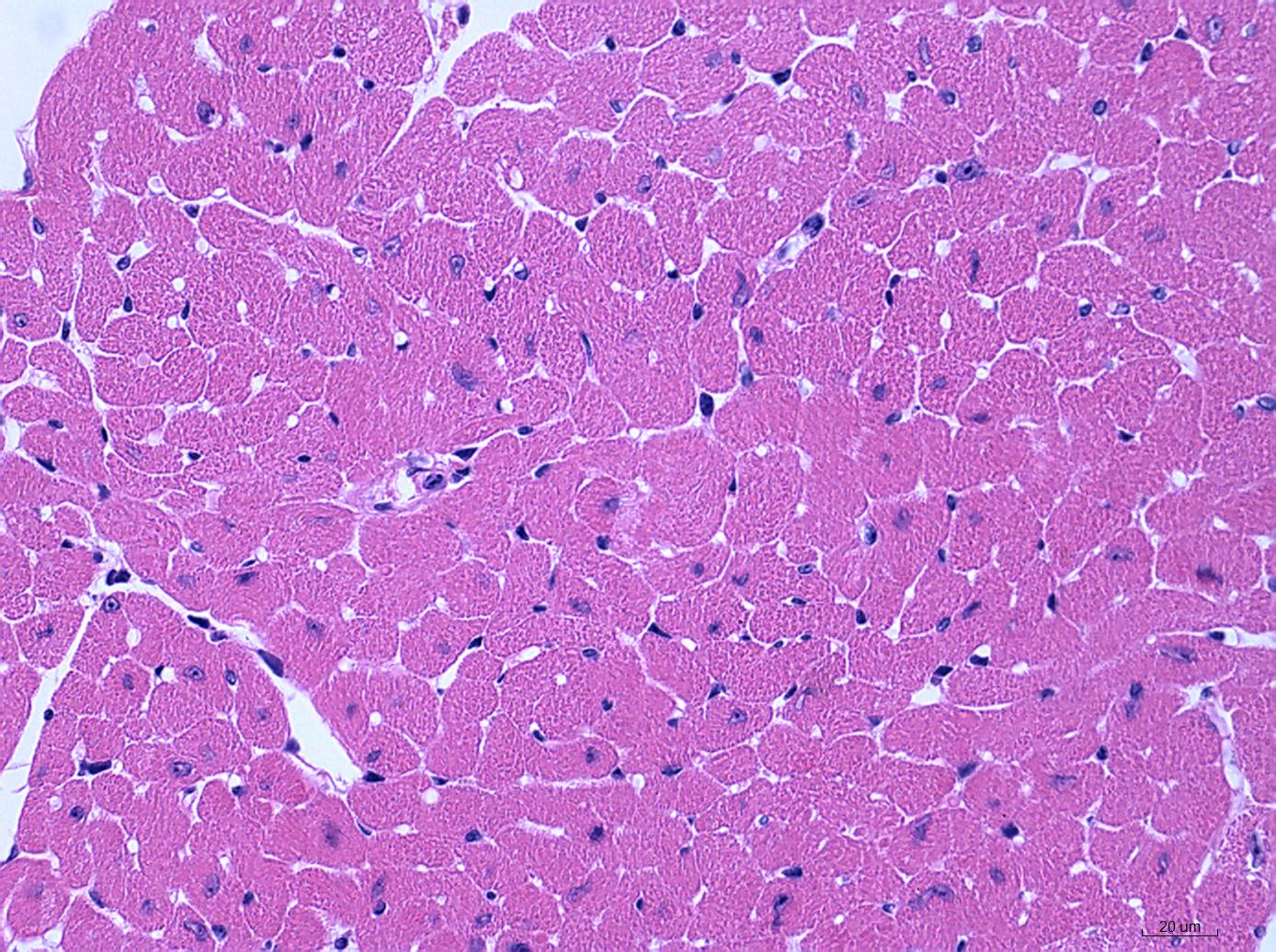

Supplement: Supplementary file 4 — Supporting File 4: advs73796‐sup‐0004‐Data.zip. [file ADVS-13-e21337-s003.zip › advs73796-sup-0004-Data/IHC_Raw_Data_Figures/Figure 4M_RawData_Figures/H&E-AAV9-cTnT-shTRIM40-Sham-40X.jpg]

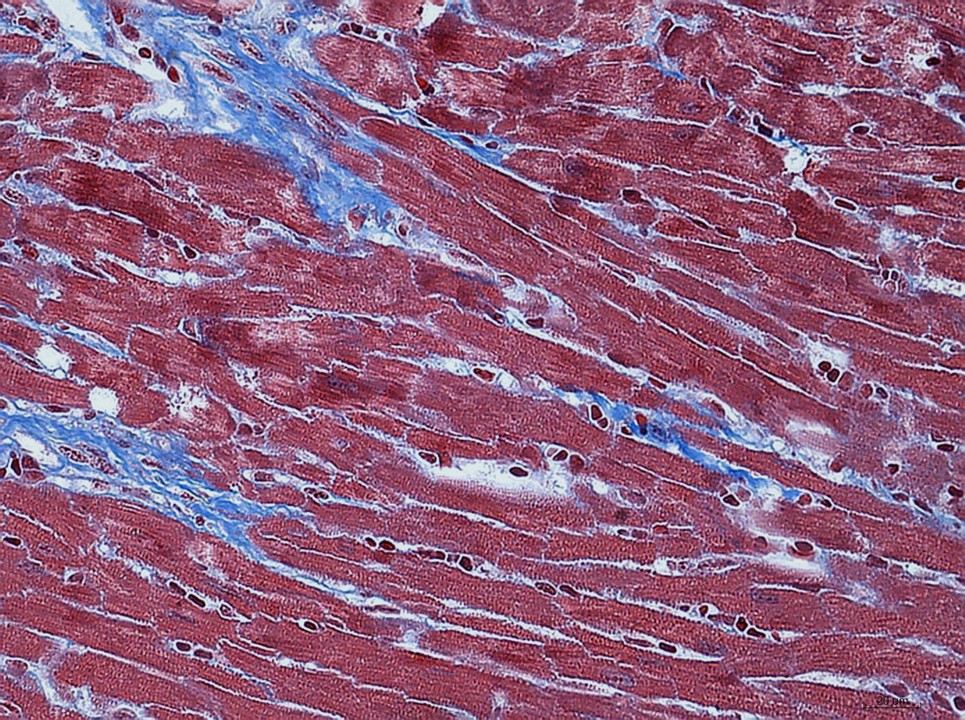

Supplement: Supplementary file 4 — Supporting File 4: advs73796‐sup‐0004‐Data.zip. [file ADVS-13-e21337-s003.zip › advs73796-sup-0004-Data/IHC_Raw_Data_Figures/Figure 4N_RawData_Figures/Masson-AAV9-cTnT-NC-Ang II-40X.jpg]

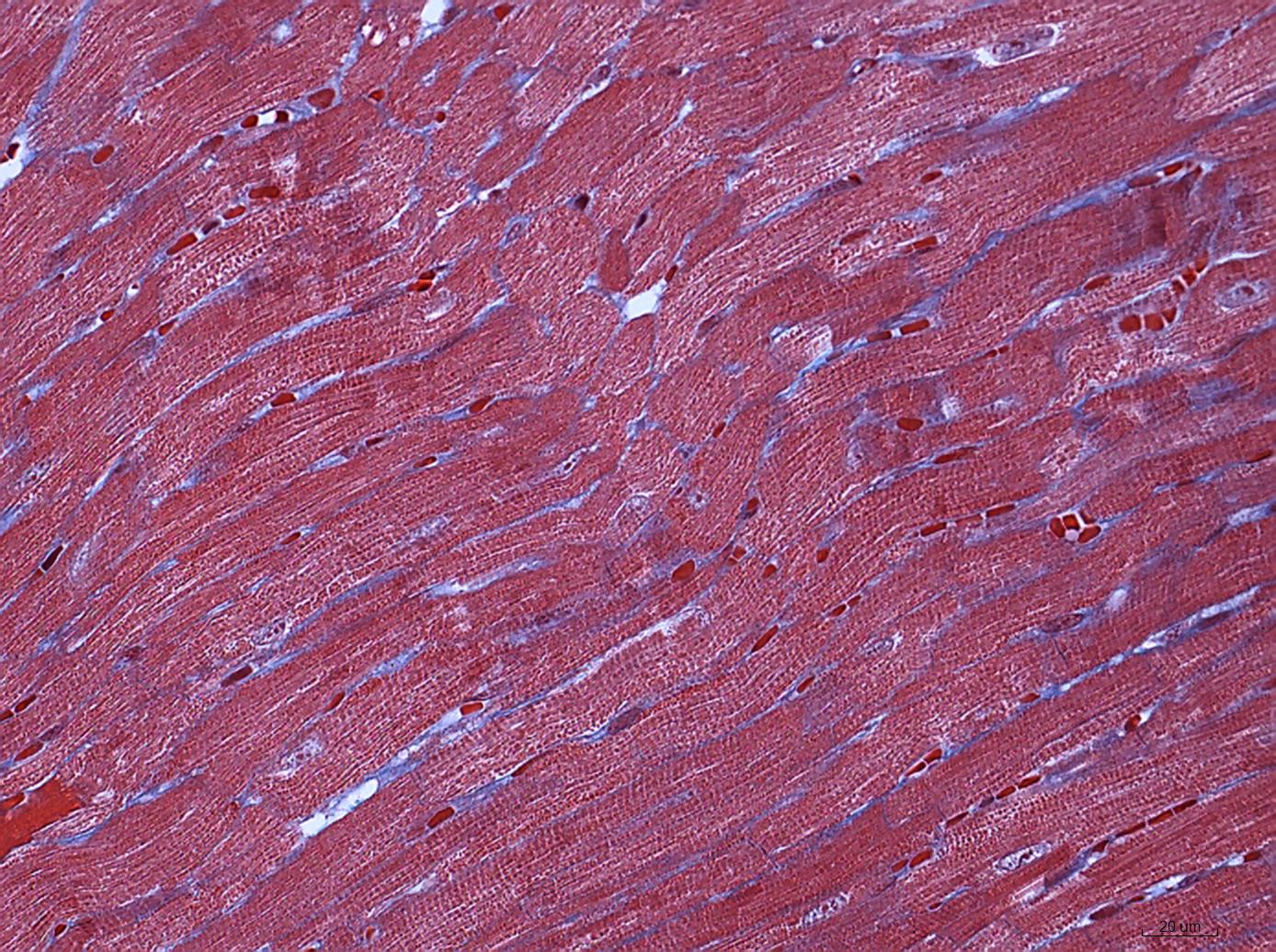

Supplement: Supplementary file 4 — Supporting File 4: advs73796‐sup‐0004‐Data.zip. [file ADVS-13-e21337-s003.zip › advs73796-sup-0004-Data/IHC_Raw_Data_Figures/Figure 4N_RawData_Figures/Masson-AAV9-cTnT-NC-Sham-40X.jpg]

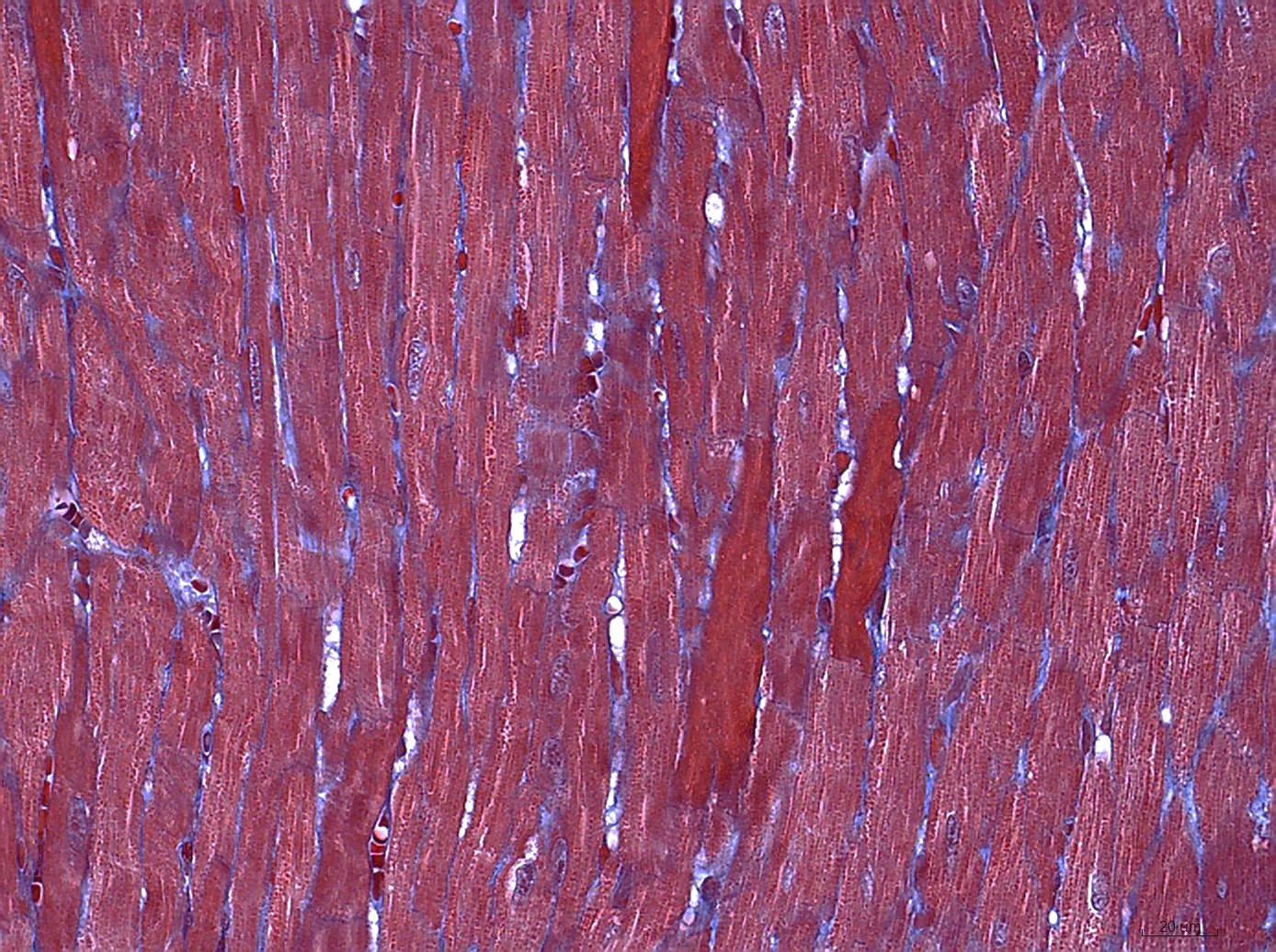

Supplement: Supplementary file 4 — Supporting File 4: advs73796‐sup‐0004‐Data.zip. [file ADVS-13-e21337-s003.zip › advs73796-sup-0004-Data/IHC_Raw_Data_Figures/Figure 4N_RawData_Figures/Masson-AAV9-cTnT-shTRIM40-Ang II-40X.jpg]

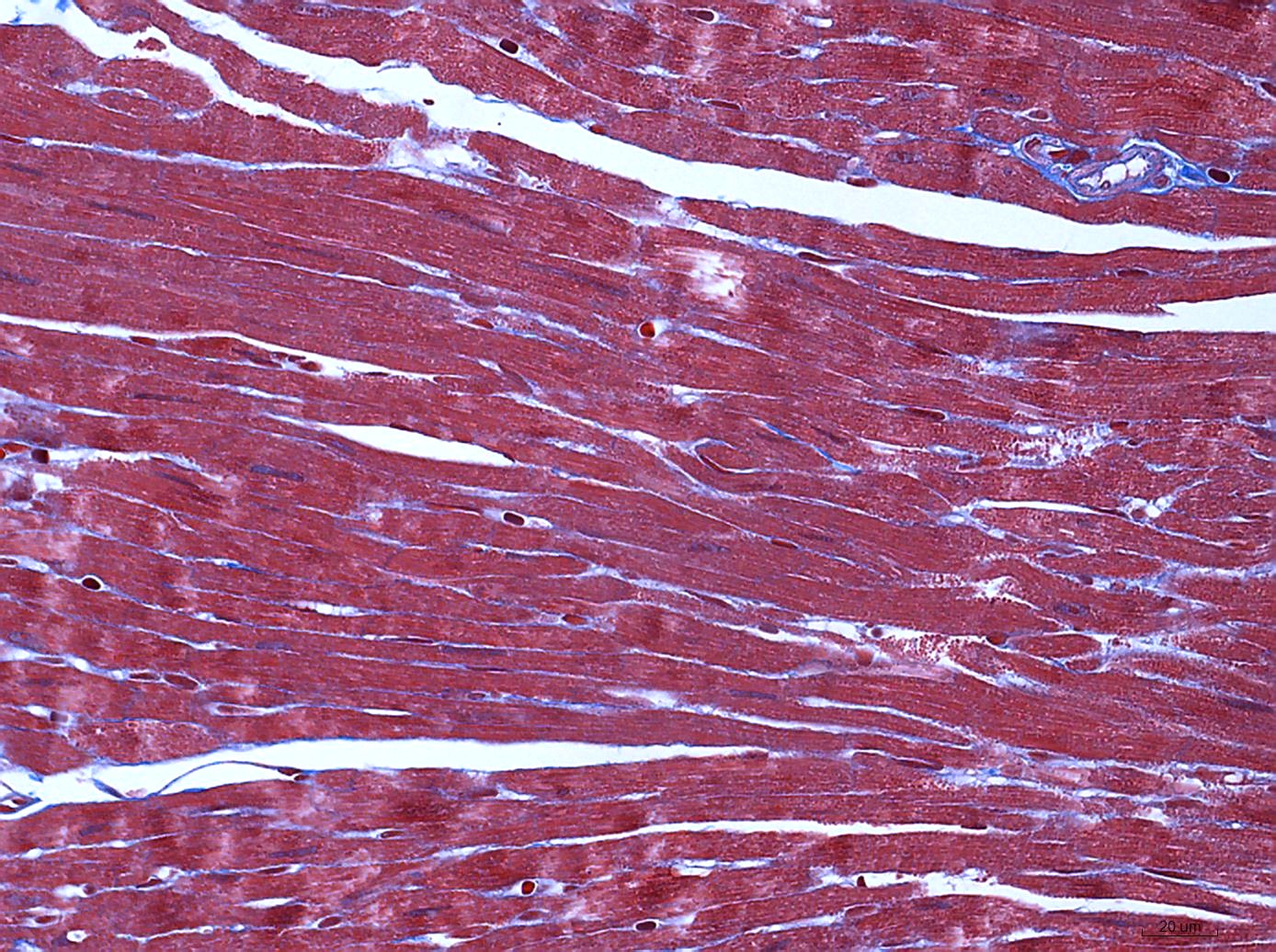

Supplement: Supplementary file 4 — Supporting File 4: advs73796‐sup‐0004‐Data.zip. [file ADVS-13-e21337-s003.zip › advs73796-sup-0004-Data/IHC_Raw_Data_Figures/Figure 4N_RawData_Figures/Masson-AAV9-cTnT-shTRIM40-Sham-40X.jpg]

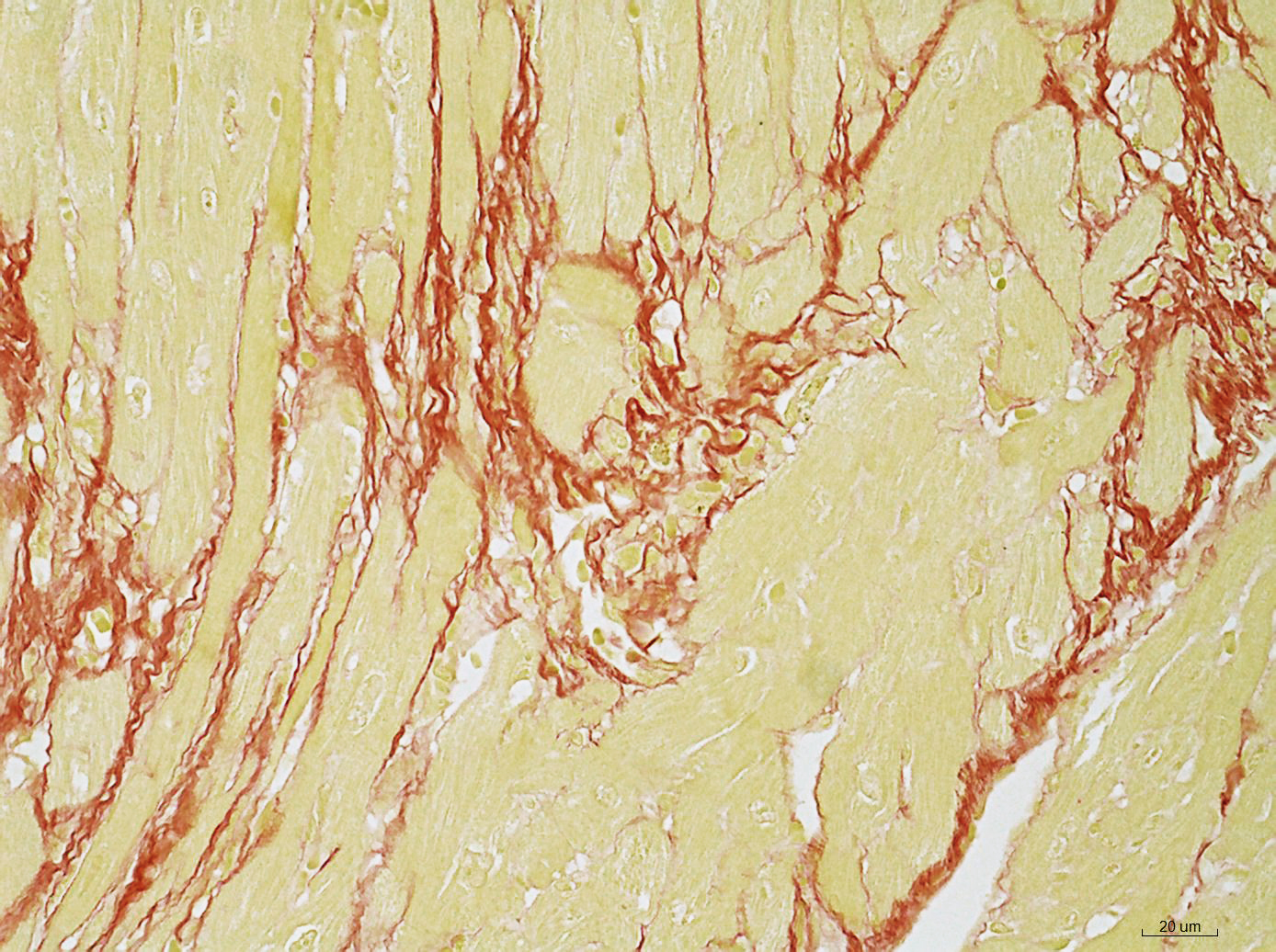

Supplement: Supplementary file 4 — Supporting File 4: advs73796‐sup‐0004‐Data.zip. [file ADVS-13-e21337-s003.zip › advs73796-sup-0004-Data/IHC_Raw_Data_Figures/Figure 4O_RawData_Figures/Sirius red-AAV9-cTnT-NC-Ang II-40X.jpg]

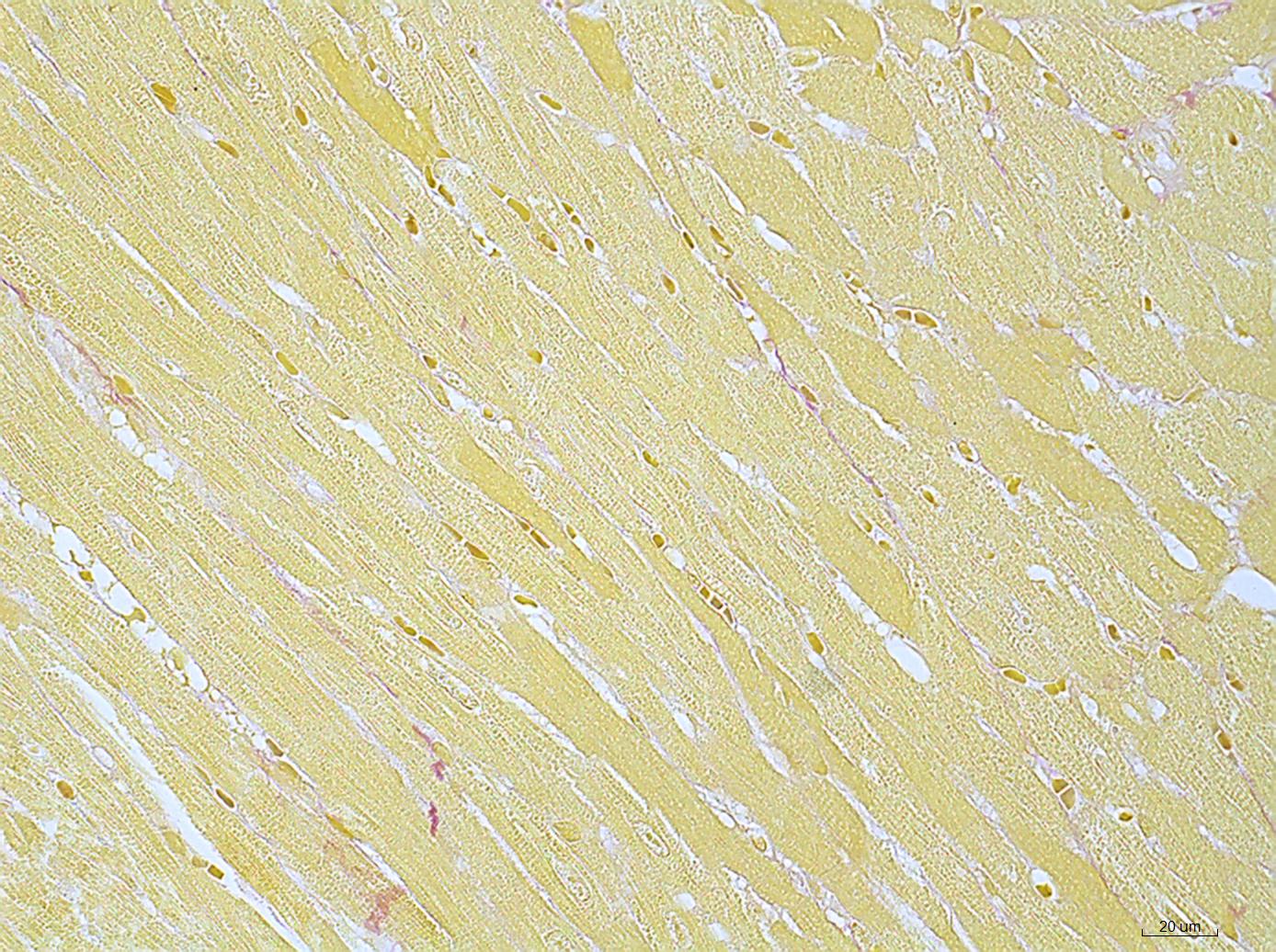

Supplement: Supplementary file 4 — Supporting File 4: advs73796‐sup‐0004‐Data.zip. [file ADVS-13-e21337-s003.zip › advs73796-sup-0004-Data/IHC_Raw_Data_Figures/Figure 4O_RawData_Figures/Sirius red-AAV9-cTnT-NC-Sham-40X.jpg]

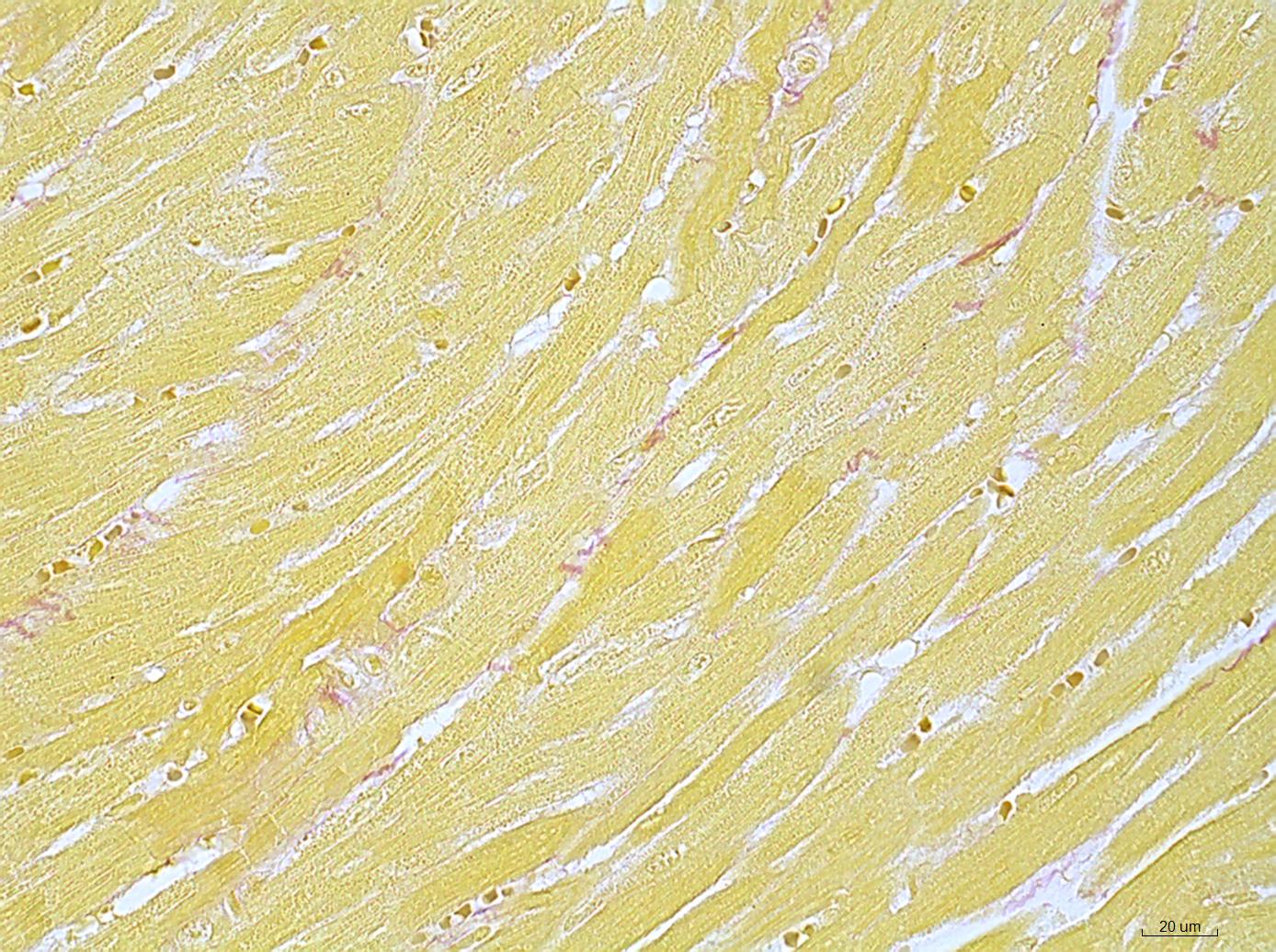

Supplement: Supplementary file 4 — Supporting File 4: advs73796‐sup‐0004‐Data.zip. [file ADVS-13-e21337-s003.zip › advs73796-sup-0004-Data/IHC_Raw_Data_Figures/Figure 4O_RawData_Figures/Sirius red-AAV9-cTnT-shTRIM40-Ang II-40X.jpg]

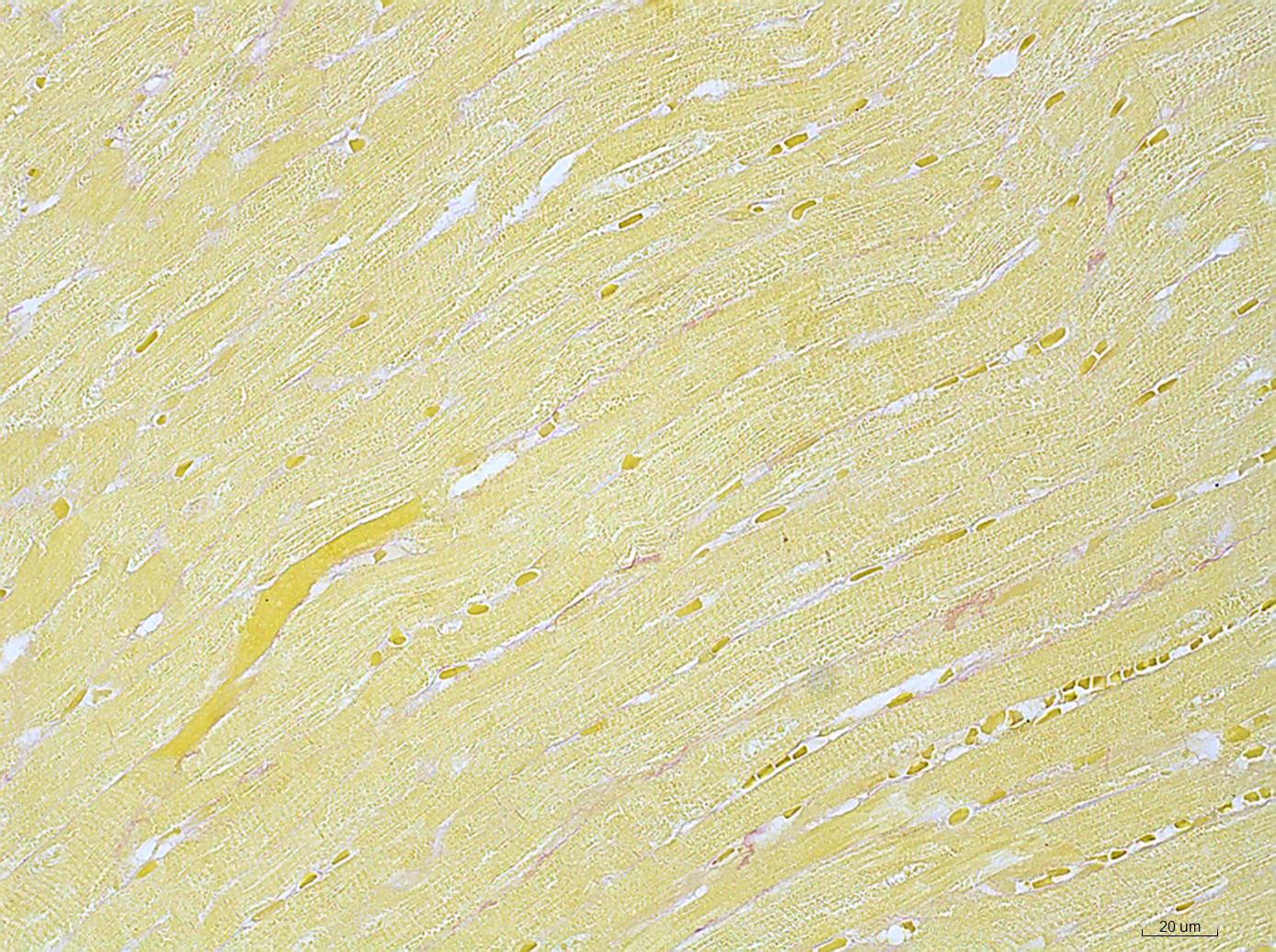

Supplement: Supplementary file 4 — Supporting File 4: advs73796‐sup‐0004‐Data.zip. [file ADVS-13-e21337-s003.zip › advs73796-sup-0004-Data/IHC_Raw_Data_Figures/Figure 4O_RawData_Figures/Sirius red-AAV9-cTnT-shTRIM40-Sham-40X.jpg]

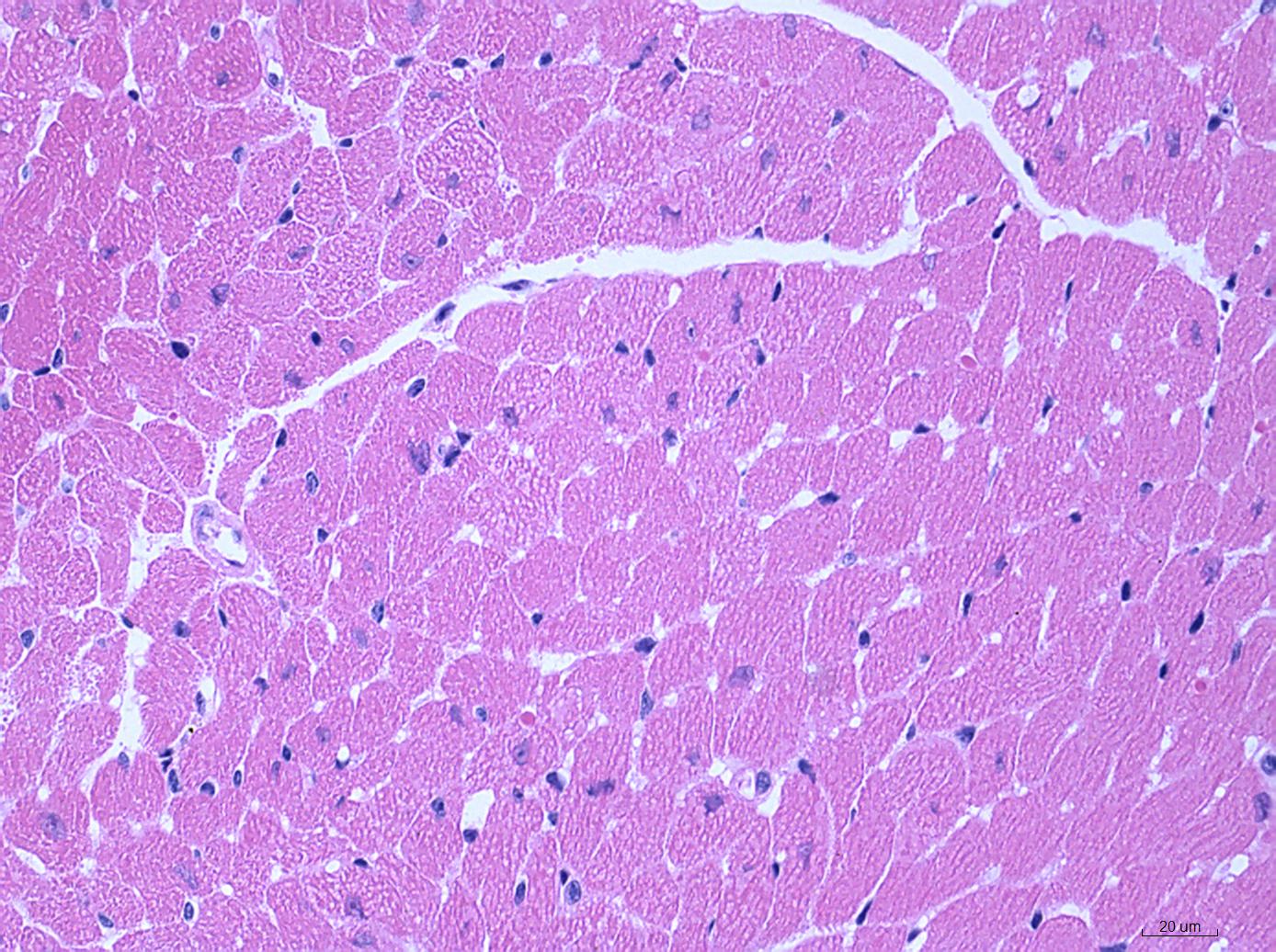

Supplement: Supplementary file 4 — Supporting File 4: advs73796‐sup‐0004‐Data.zip. [file ADVS-13-e21337-s003.zip › advs73796-sup-0004-Data/IHC_Raw_Data_Figures/Figure 8K_RawData_Figures/H&E-WT-AAV-NC-40X.jpg]

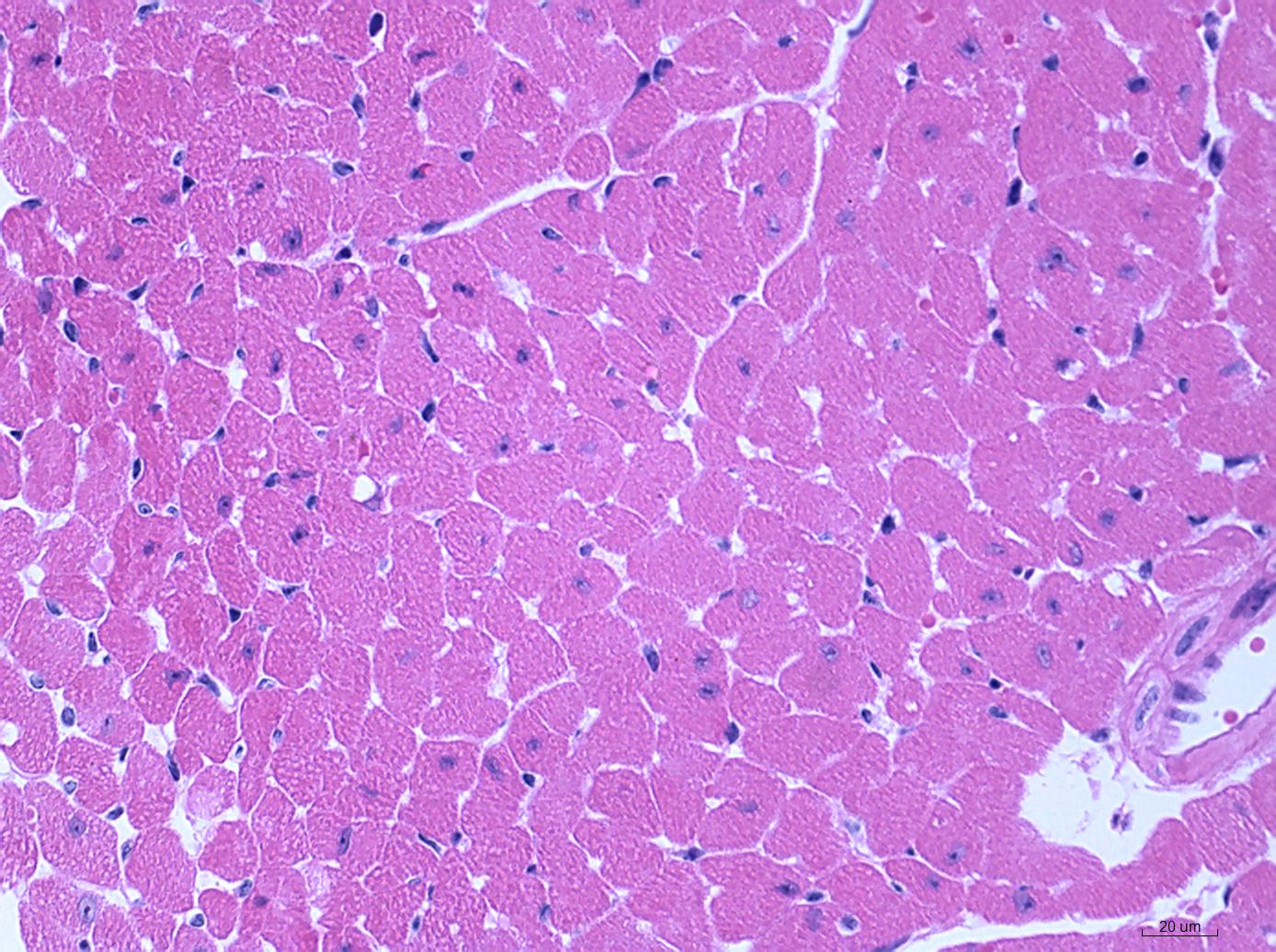

Supplement: Supplementary file 4 — Supporting File 4: advs73796‐sup‐0004‐Data.zip. [file ADVS-13-e21337-s003.zip › advs73796-sup-0004-Data/IHC_Raw_Data_Figures/Figure 8K_RawData_Figures/H&E-WT-AAV-TRIM40-40X.jpg]

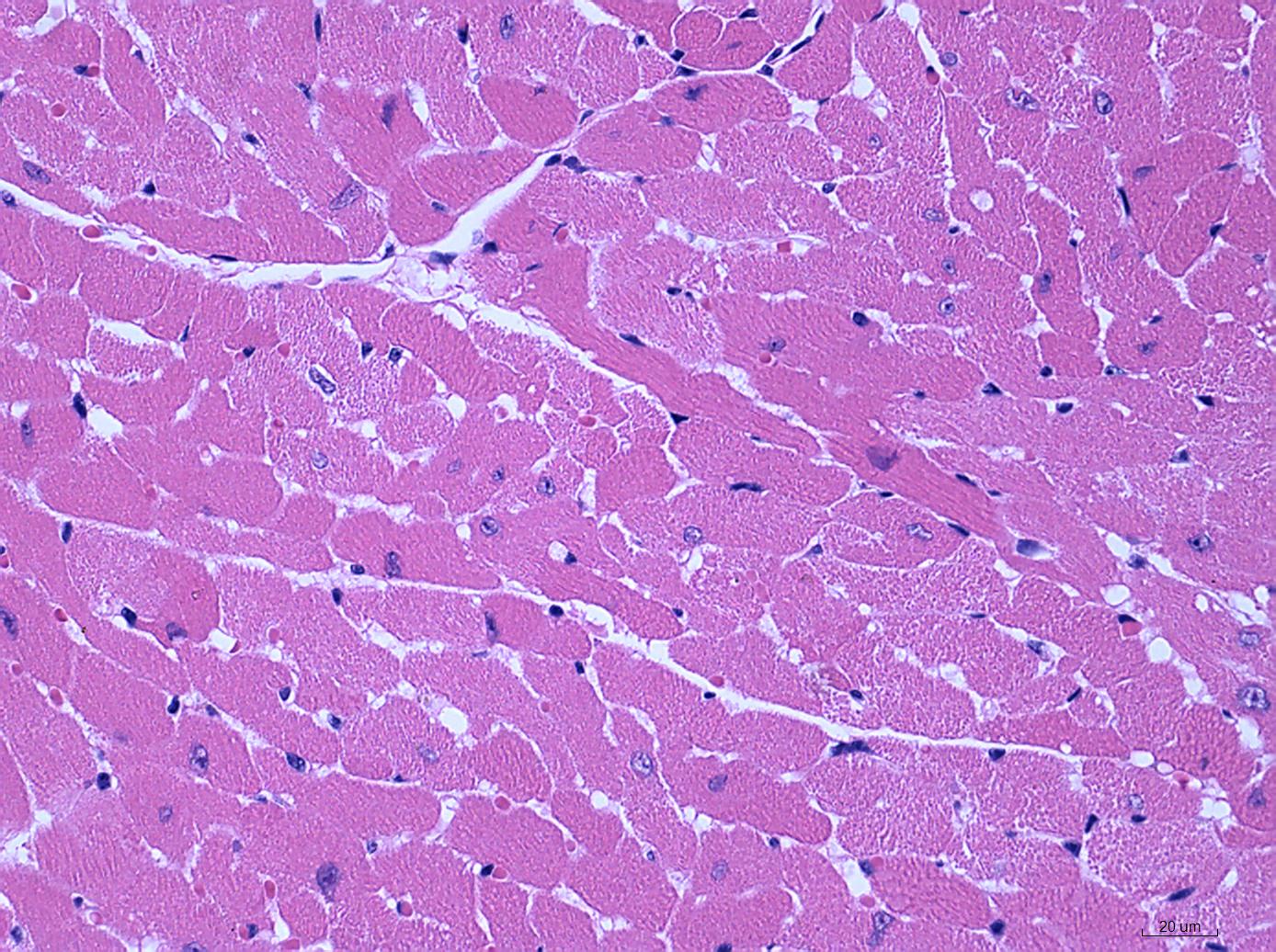

Supplement: Supplementary file 4 — Supporting File 4: advs73796‐sup‐0004‐Data.zip. [file ADVS-13-e21337-s003.zip › advs73796-sup-0004-Data/IHC_Raw_Data_Figures/Figure 8K_RawData_Figures/H&E-WT-Ang II-AAV-NC-40X.jpg]

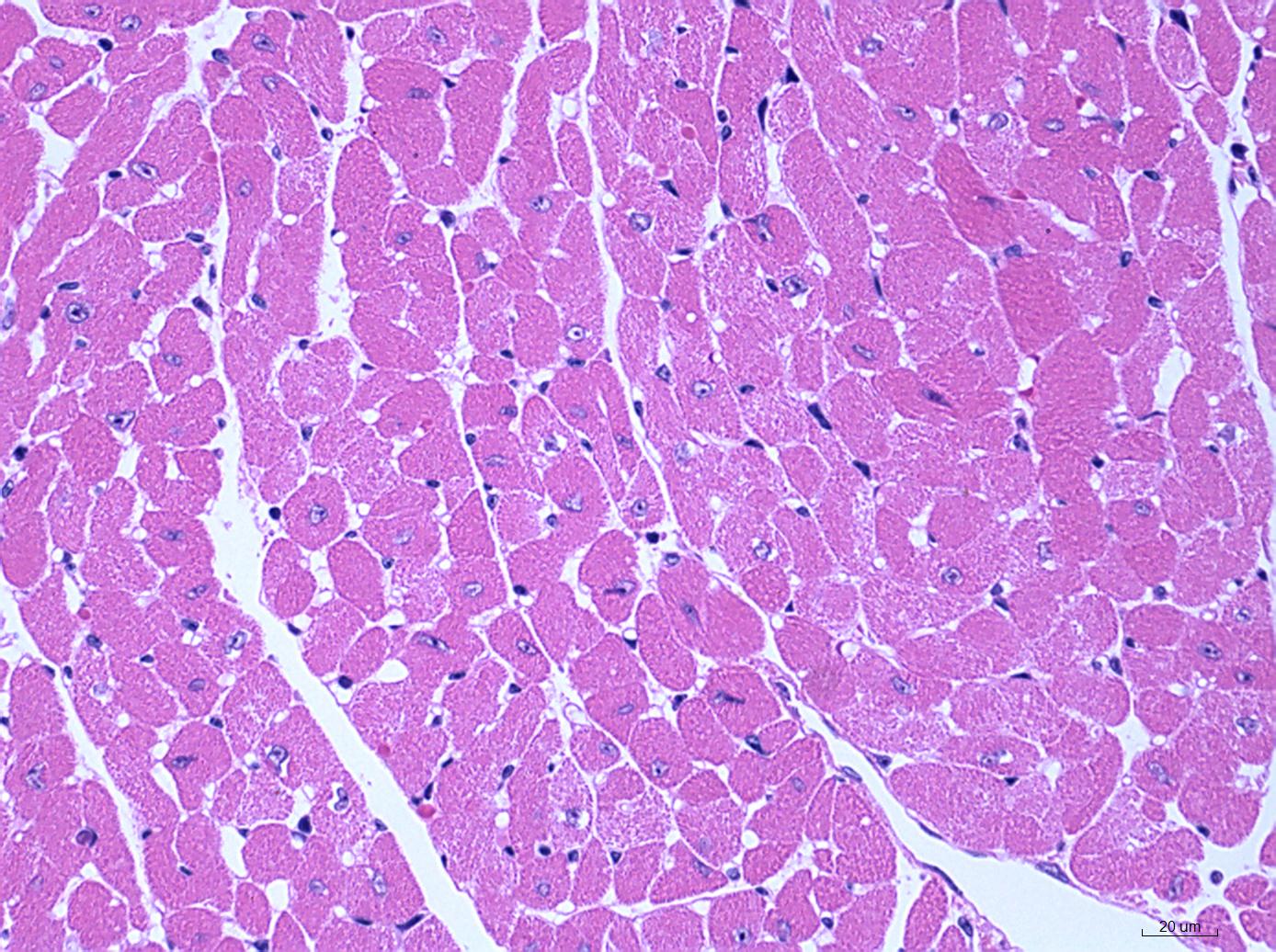

Supplement: Supplementary file 4 — Supporting File 4: advs73796‐sup‐0004‐Data.zip. [file ADVS-13-e21337-s003.zip › advs73796-sup-0004-Data/IHC_Raw_Data_Figures/Figure 8K_RawData_Figures/H&E-WT-Ang II-AAV-TRIM40+PKN1 or 2-IN-1-40X.jpg]

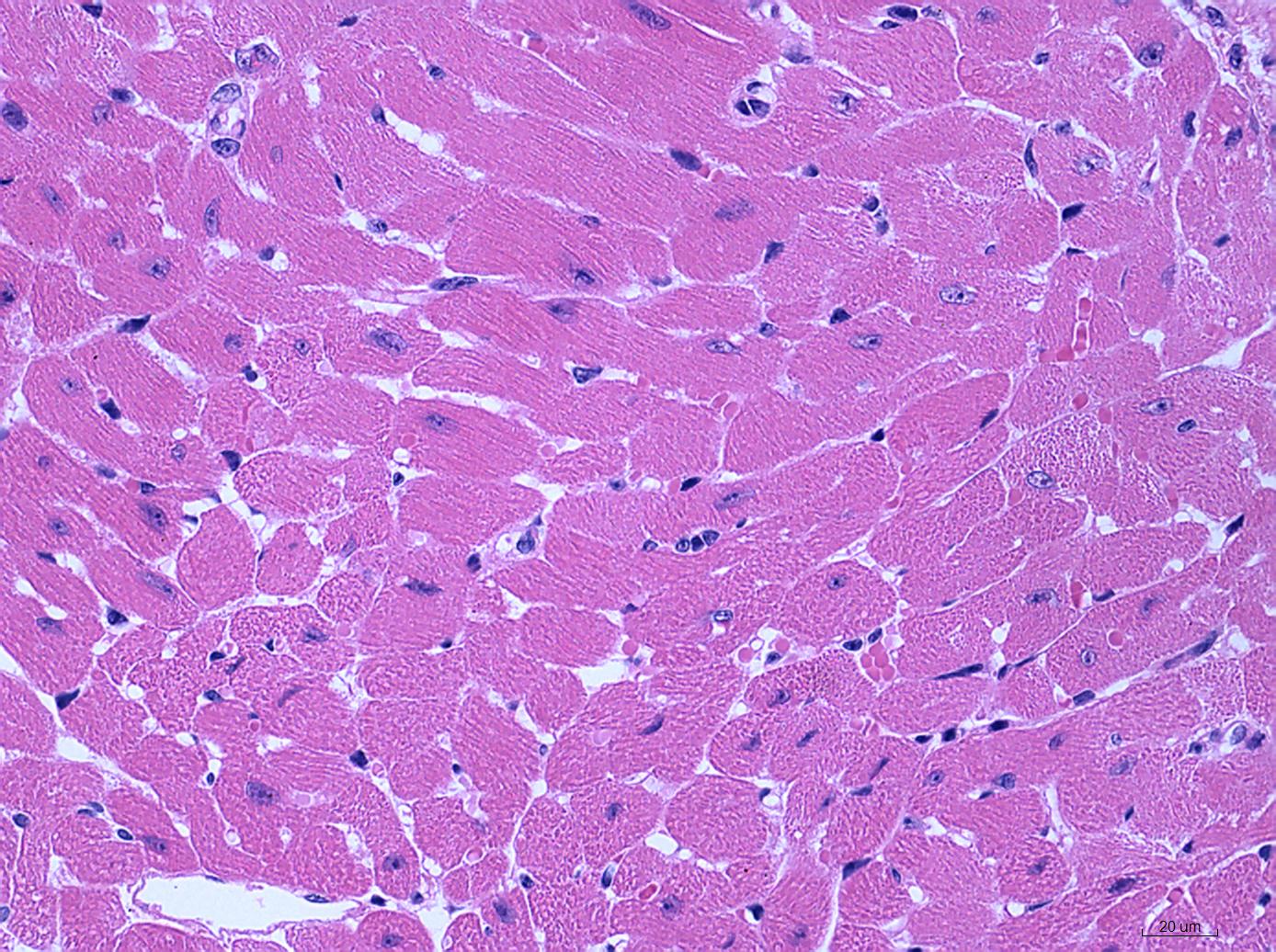

Supplement: Supplementary file 4 — Supporting File 4: advs73796‐sup‐0004‐Data.zip. [file ADVS-13-e21337-s003.zip › advs73796-sup-0004-Data/IHC_Raw_Data_Figures/Figure 8K_RawData_Figures/H&E-WT-Ang II-AAV-TRIM40-40X.jpg]

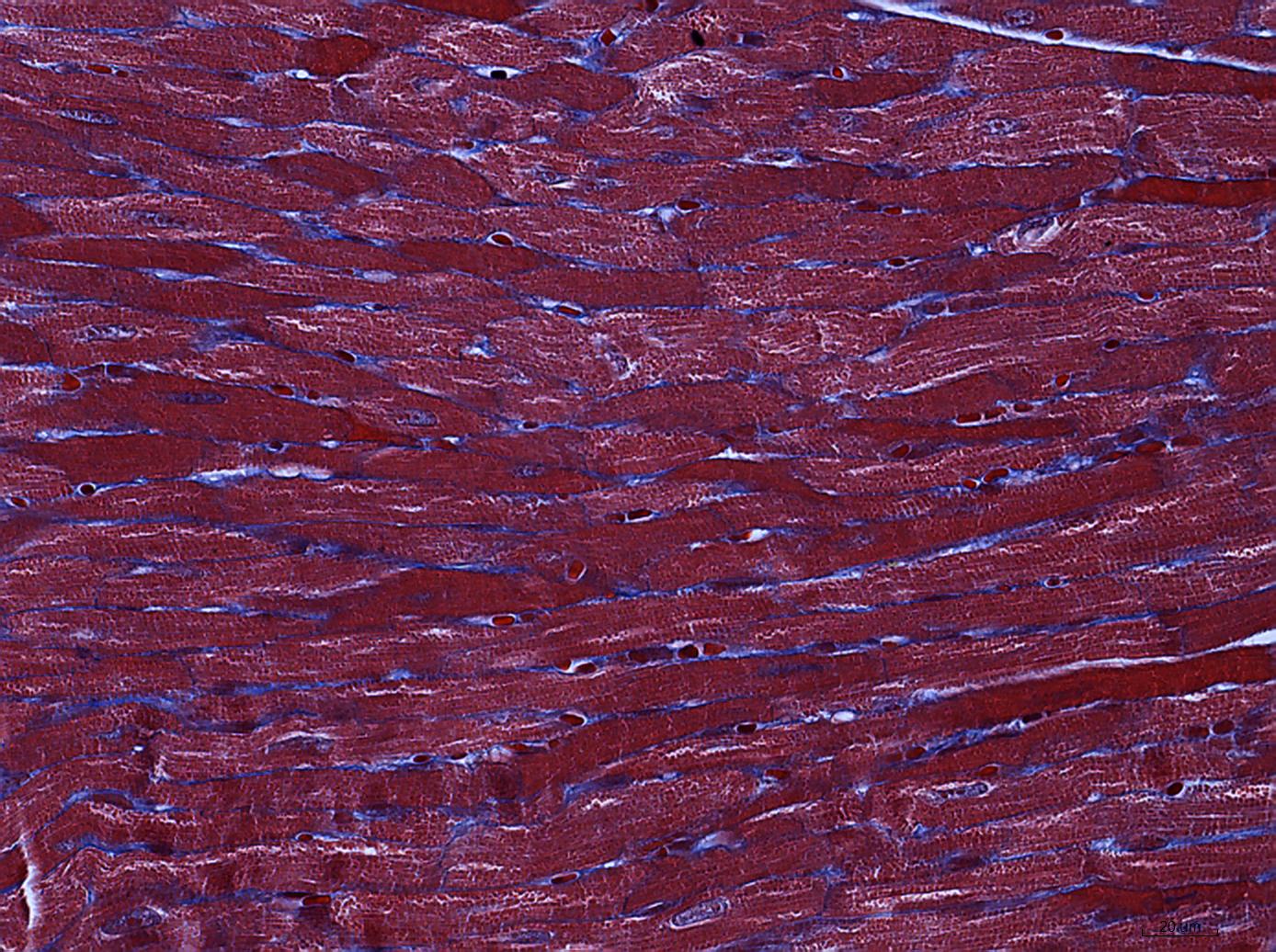

Supplement: Supplementary file 4 — Supporting File 4: advs73796‐sup‐0004‐Data.zip. [file ADVS-13-e21337-s003.zip › advs73796-sup-0004-Data/IHC_Raw_Data_Figures/Figure 8L_RawData_Figures/Masson-WT-AAV-NC-40X.jpg]

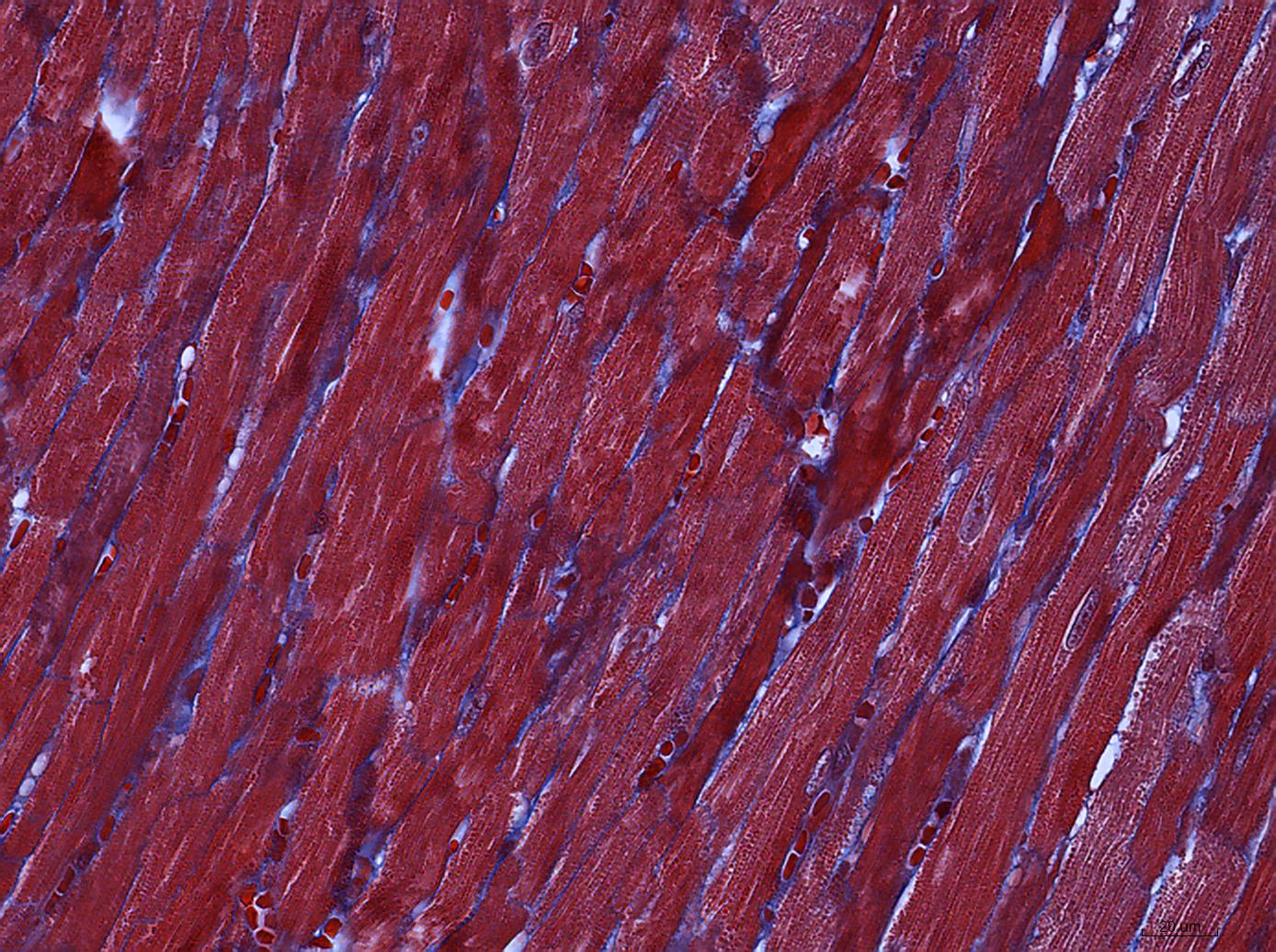

Supplement: Supplementary file 4 — Supporting File 4: advs73796‐sup‐0004‐Data.zip. [file ADVS-13-e21337-s003.zip › advs73796-sup-0004-Data/IHC_Raw_Data_Figures/Figure 8L_RawData_Figures/Masson-WT-AAV-TRIM40-40X.jpg]

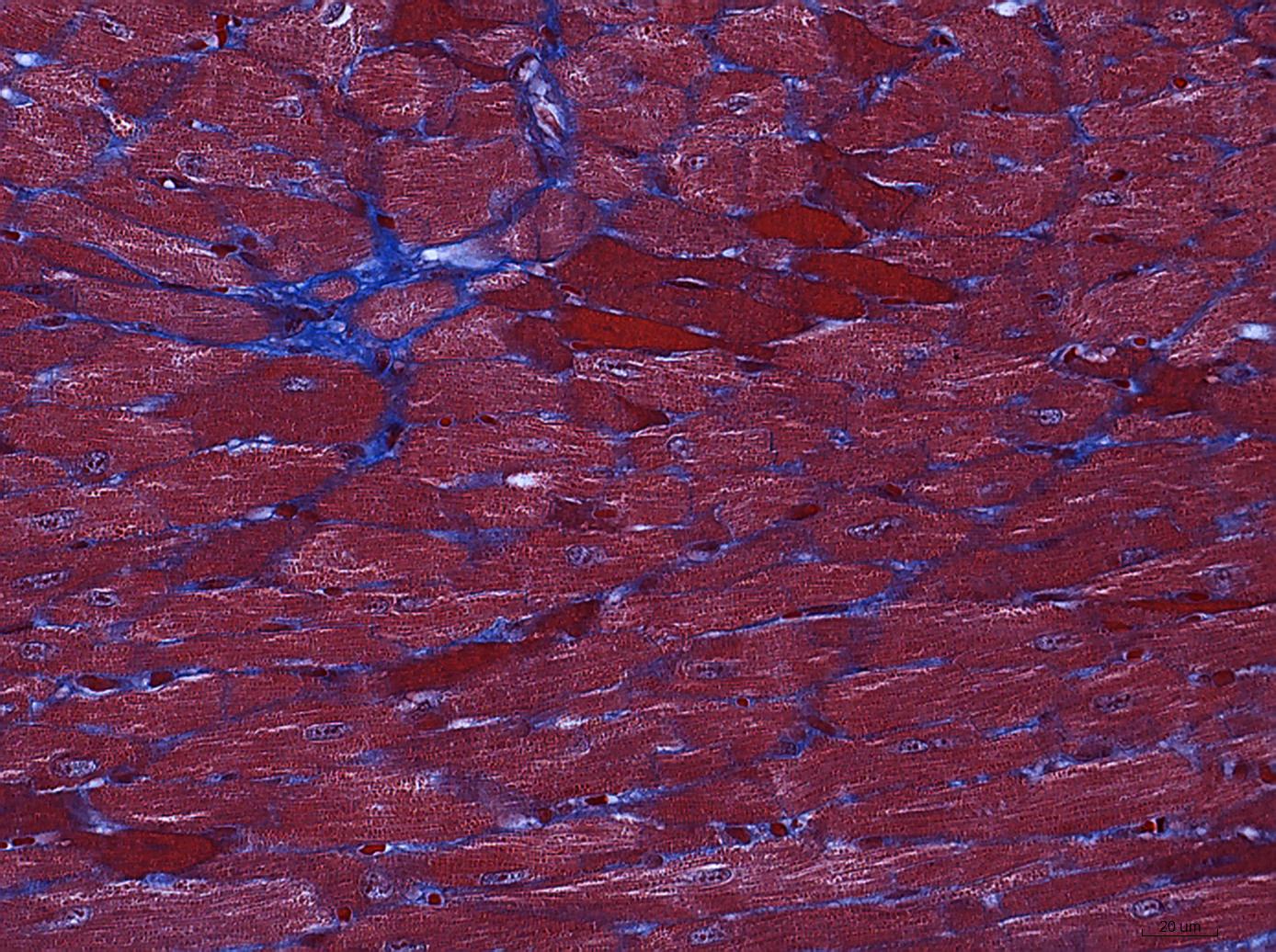

Supplement: Supplementary file 4 — Supporting File 4: advs73796‐sup‐0004‐Data.zip. [file ADVS-13-e21337-s003.zip › advs73796-sup-0004-Data/IHC_Raw_Data_Figures/Figure 8L_RawData_Figures/Masson-WT-Ang II-AAV-NC-40X.jpg]

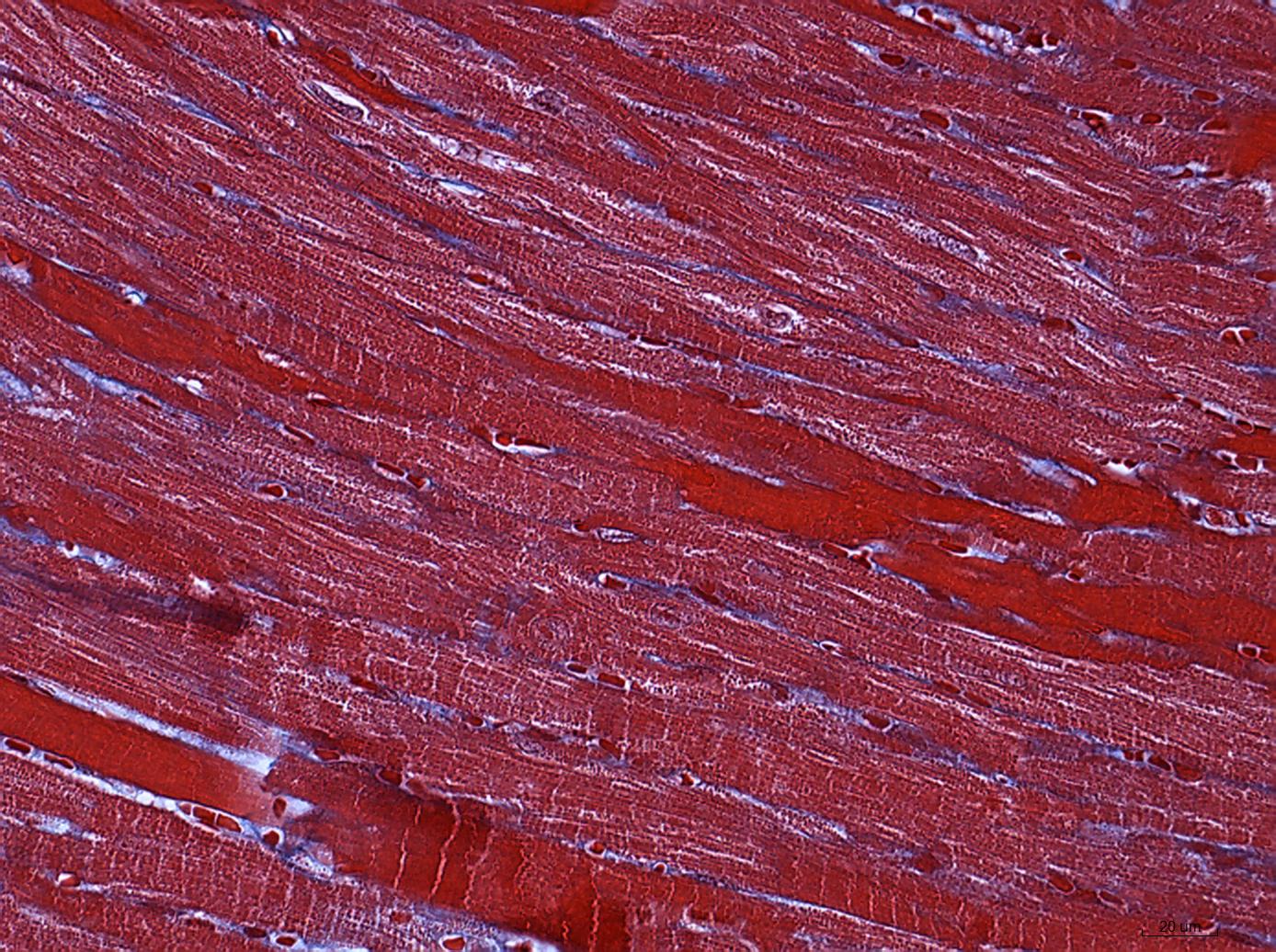

Supplement: Supplementary file 4 — Supporting File 4: advs73796‐sup‐0004‐Data.zip. [file ADVS-13-e21337-s003.zip › advs73796-sup-0004-Data/IHC_Raw_Data_Figures/Figure 8L_RawData_Figures/Masson-WT-Ang II-AAV-TRIM40+PKN1 or 2-IN-1-40X.jpg]

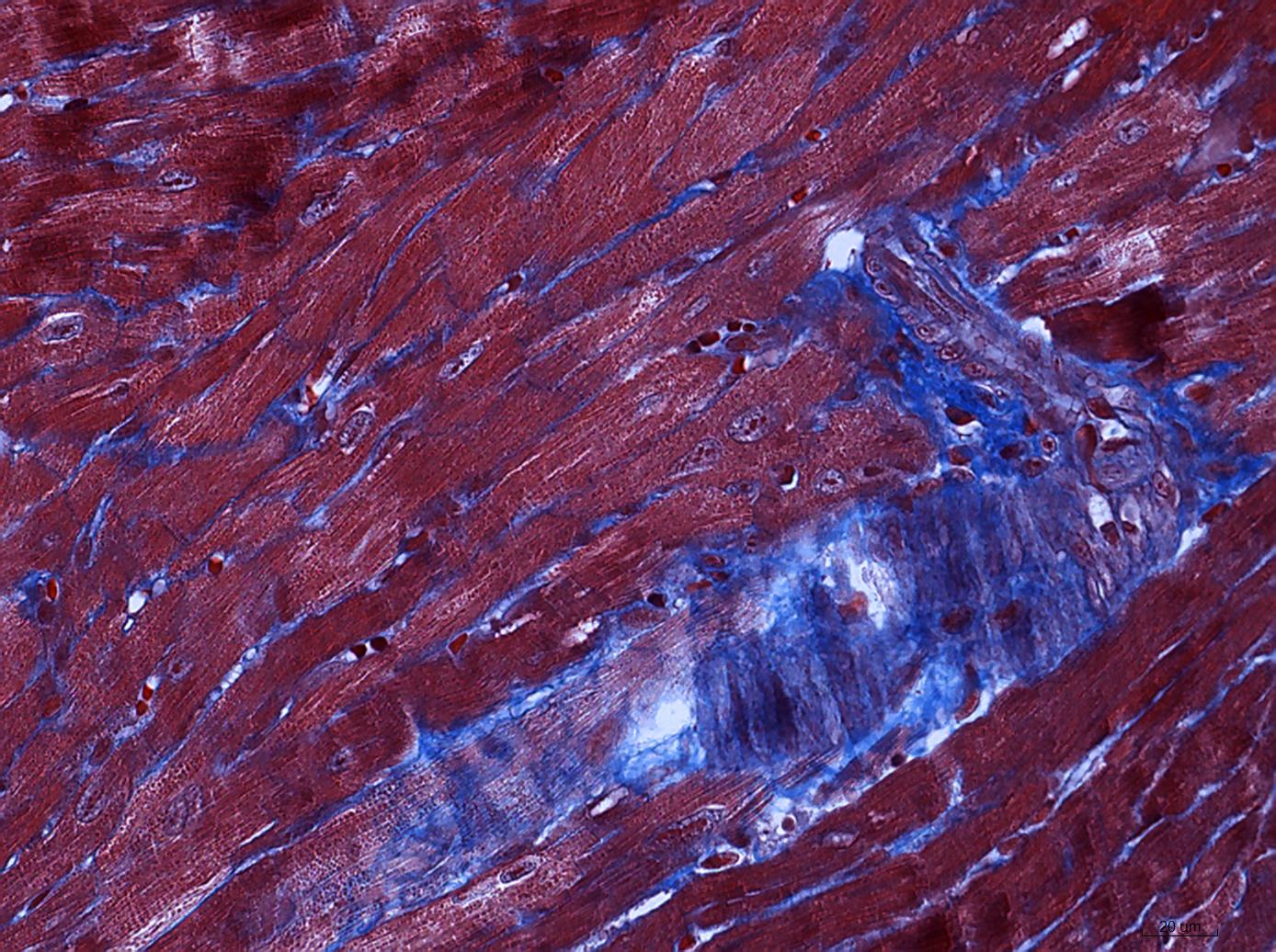

Supplement: Supplementary file 4 — Supporting File 4: advs73796‐sup‐0004‐Data.zip. [file ADVS-13-e21337-s003.zip › advs73796-sup-0004-Data/IHC_Raw_Data_Figures/Figure 8L_RawData_Figures/Masson-WT-Ang II-AAV-TRIM40-40X.jpg]

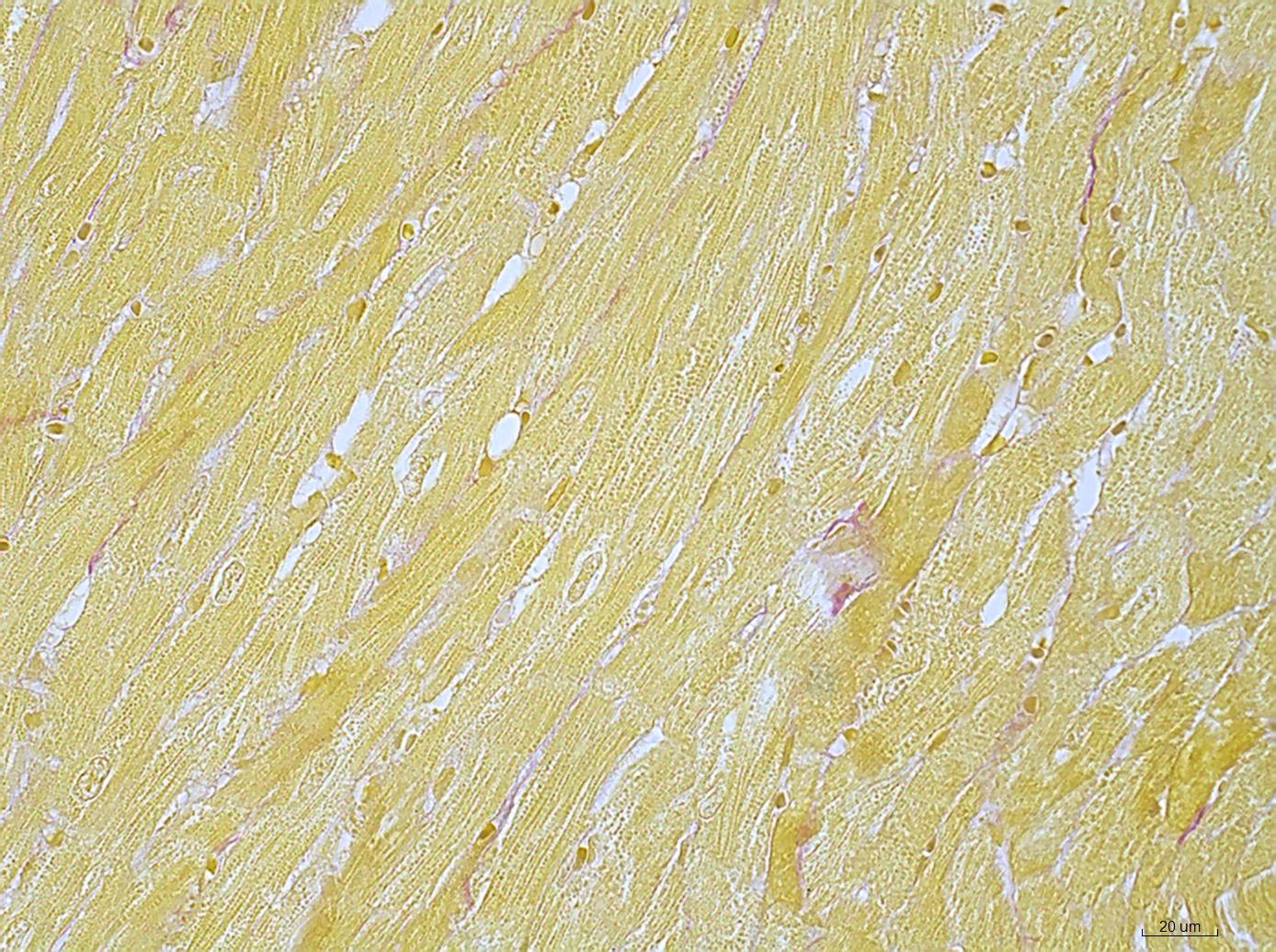

Supplement: Supplementary file 4 — Supporting File 4: advs73796‐sup‐0004‐Data.zip. [file ADVS-13-e21337-s003.zip › advs73796-sup-0004-Data/IHC_Raw_Data_Figures/Figure 8M_RawData_Figures/Sirius red-WT-AAV-NC-40X.jpg]

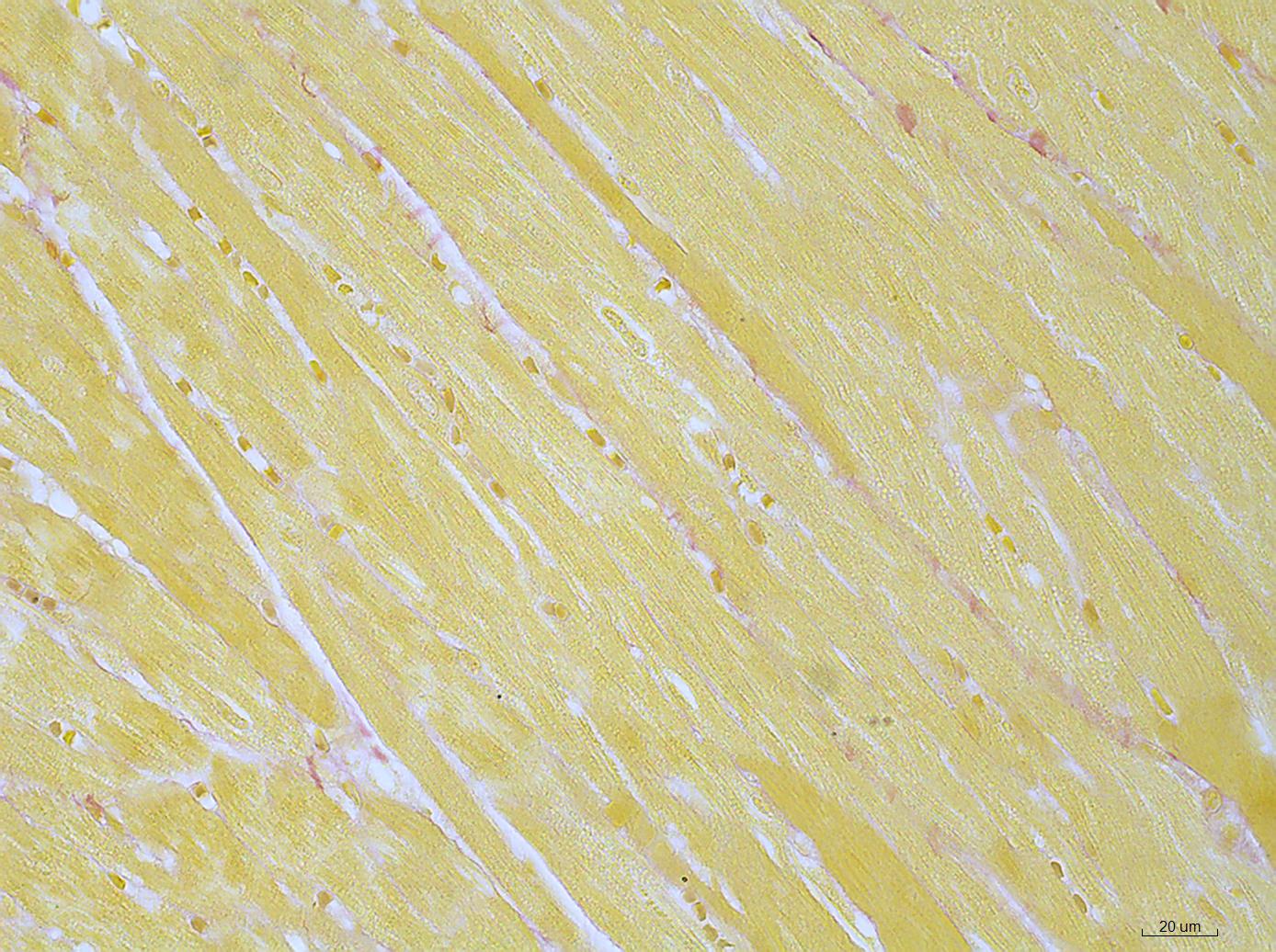

Supplement: Supplementary file 4 — Supporting File 4: advs73796‐sup‐0004‐Data.zip. [file ADVS-13-e21337-s003.zip › advs73796-sup-0004-Data/IHC_Raw_Data_Figures/Figure 8M_RawData_Figures/Sirius red-WT-AAV-TRIM40-40X.jpg]

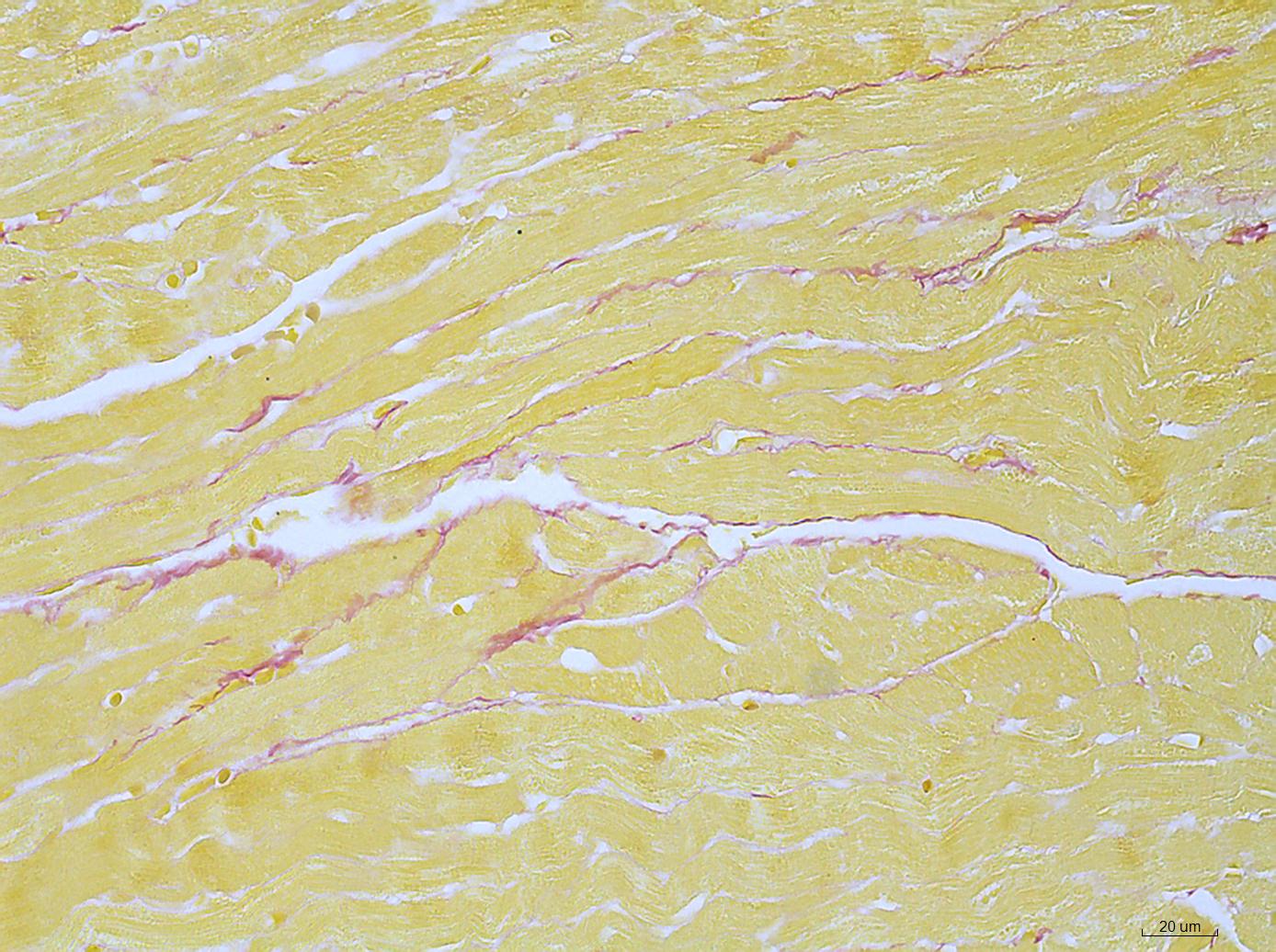

Supplement: Supplementary file 4 — Supporting File 4: advs73796‐sup‐0004‐Data.zip. [file ADVS-13-e21337-s003.zip › advs73796-sup-0004-Data/IHC_Raw_Data_Figures/Figure 8M_RawData_Figures/Sirius red-WT-Ang II-AAV-NC-40X.jpg]

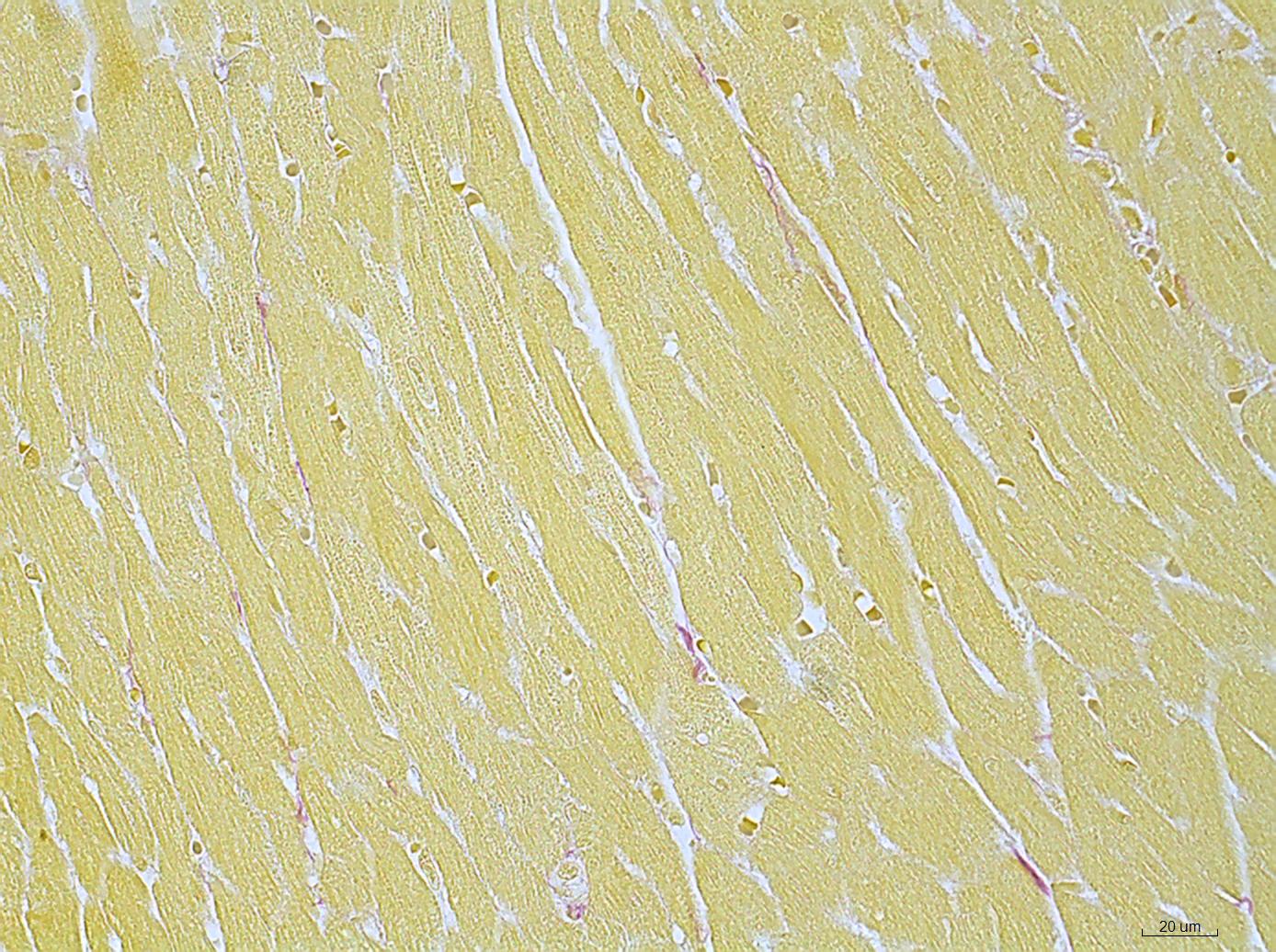

Supplement: Supplementary file 4 — Supporting File 4: advs73796‐sup‐0004‐Data.zip. [file ADVS-13-e21337-s003.zip › advs73796-sup-0004-Data/IHC_Raw_Data_Figures/Figure 8M_RawData_Figures/Sirius red-WT-Ang II-AAV-TRIM40+PKN1 or 2-IN-1-40X.jpg]

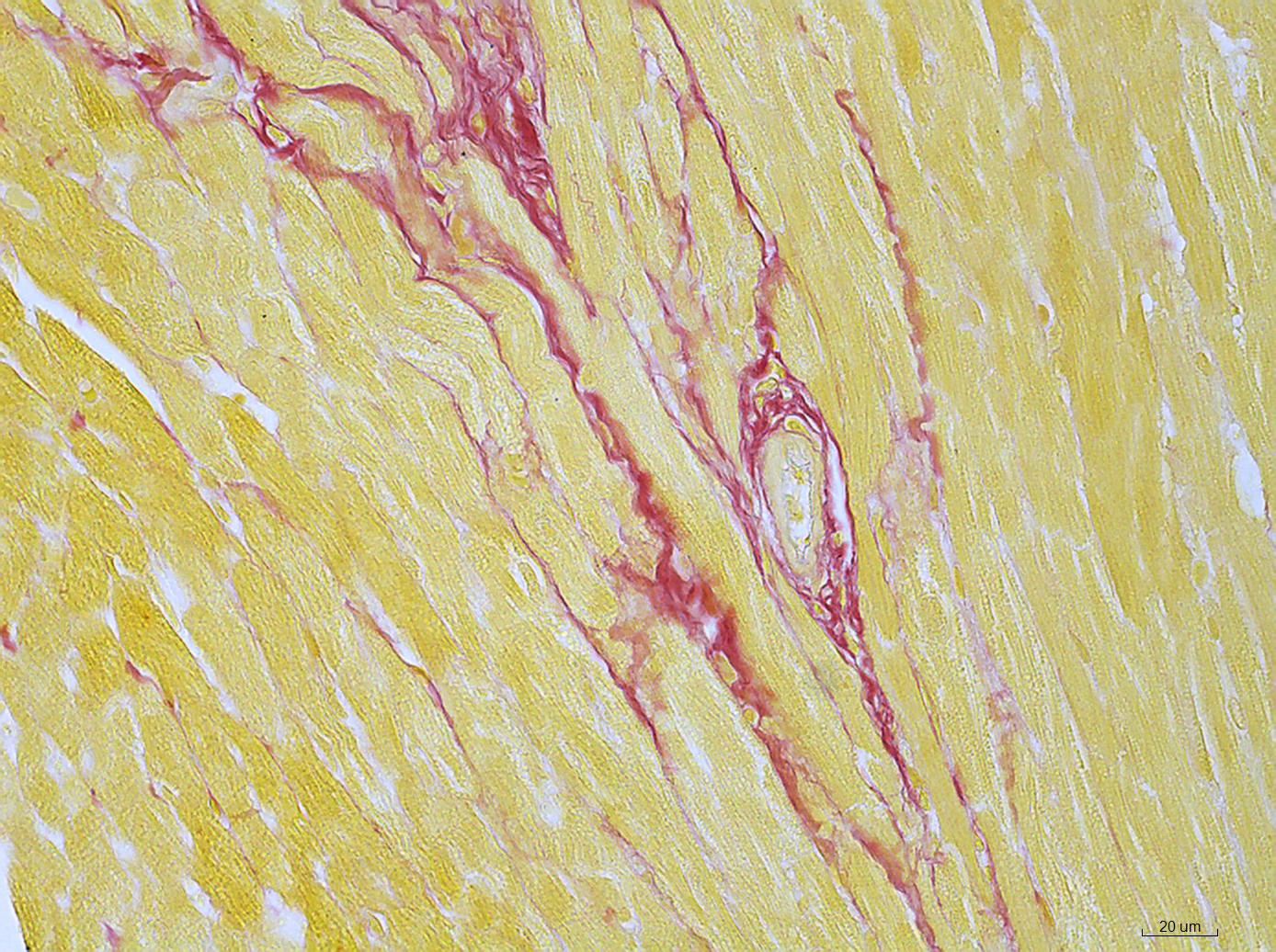

Supplement: Supplementary file 4 — Supporting File 4: advs73796‐sup‐0004‐Data.zip. [file ADVS-13-e21337-s003.zip › advs73796-sup-0004-Data/IHC_Raw_Data_Figures/Figure 8M_RawData_Figures/Sirius red-WT-Ang II-AAV-TRIM40-40X.jpg]

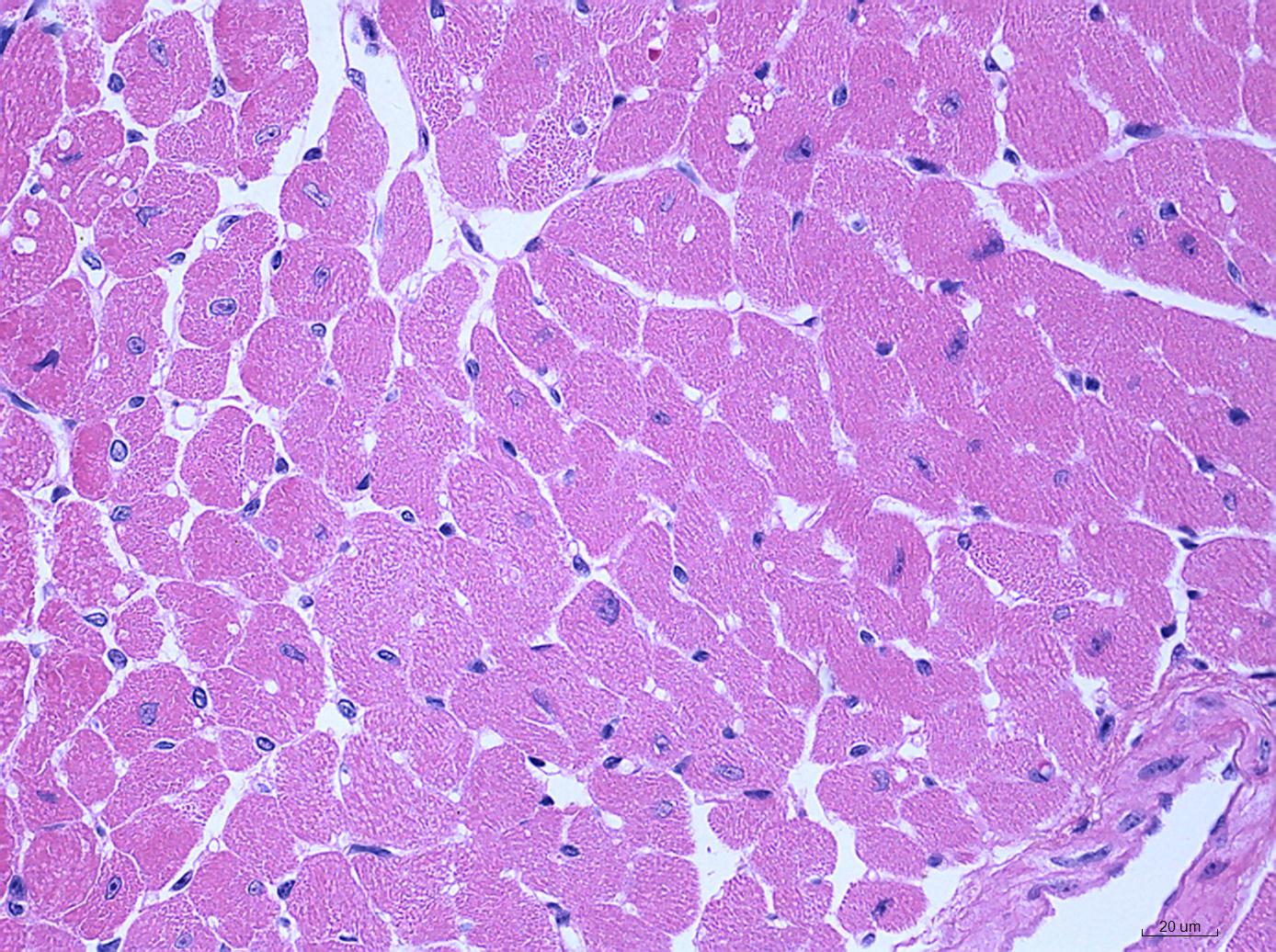

Supplement: Supplementary file 4 — Supporting File 4: advs73796‐sup‐0004‐Data.zip. [file ADVS-13-e21337-s003.zip › advs73796-sup-0004-Data/IHC_Raw_Data_Figures/Figure 9J_RawData_Figures/H&E-WT+TAC-AAV-NC-40X.jpg]

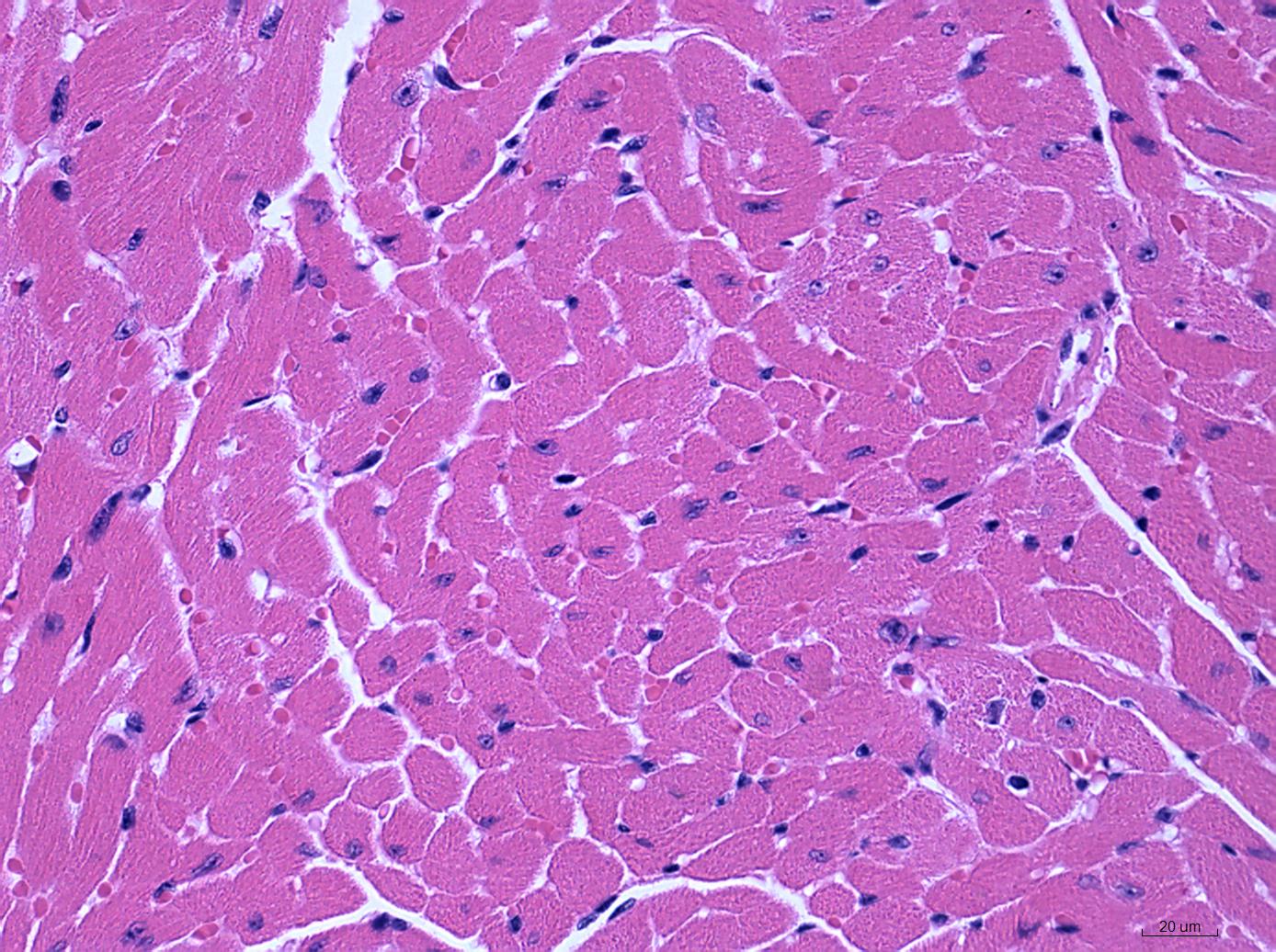

Supplement: Supplementary file 4 — Supporting File 4: advs73796‐sup‐0004‐Data.zip. [file ADVS-13-e21337-s003.zip › advs73796-sup-0004-Data/IHC_Raw_Data_Figures/Figure 9J_RawData_Figures/H&E-WT+TAC-AAV-TRIM40+PKN1 or 2-IN-1-40X.jpg]

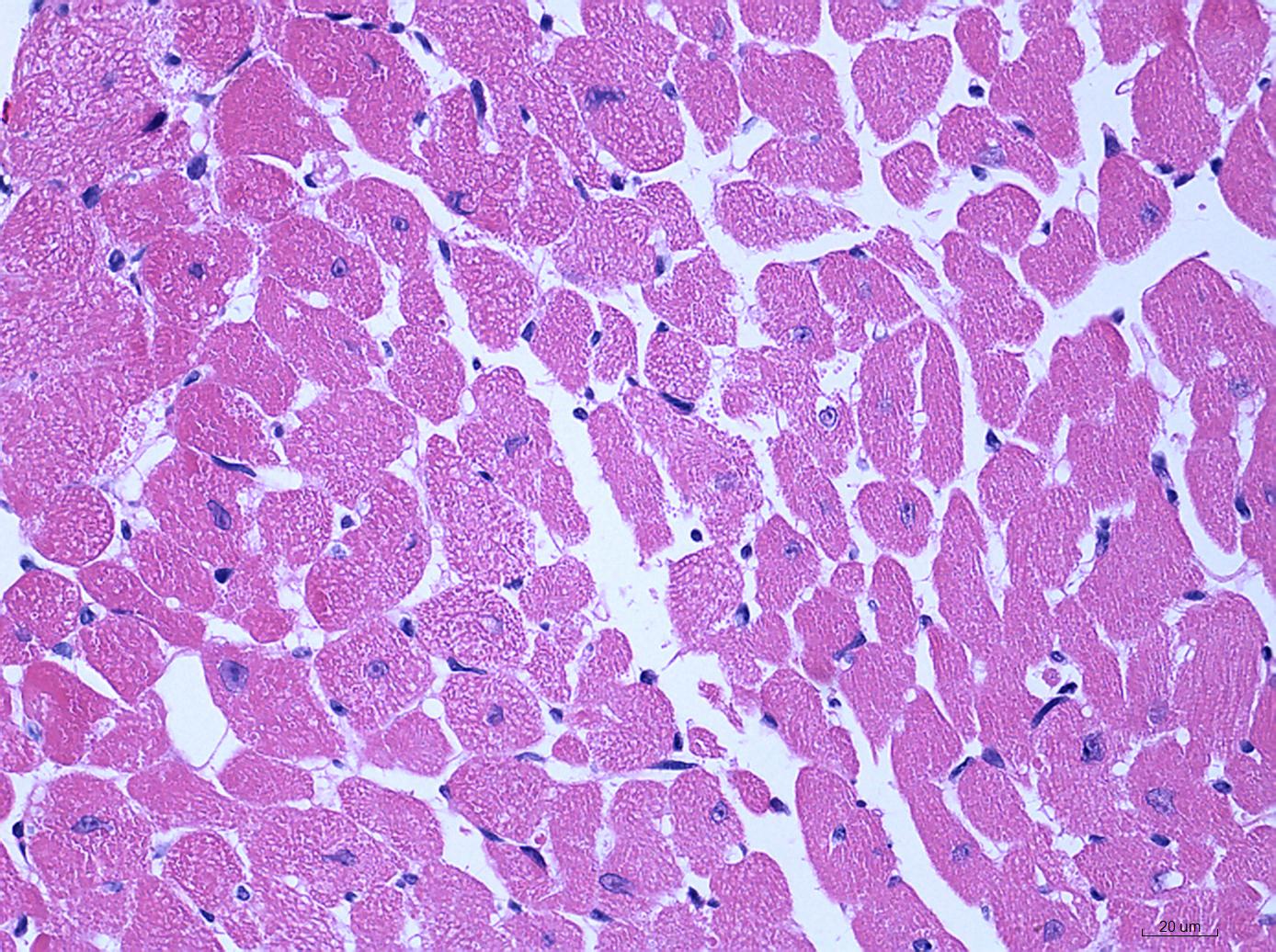

Supplement: Supplementary file 4 — Supporting File 4: advs73796‐sup‐0004‐Data.zip. [file ADVS-13-e21337-s003.zip › advs73796-sup-0004-Data/IHC_Raw_Data_Figures/Figure 9J_RawData_Figures/H&E-WT+TAC-AAV-TRIM40-40X.jpg]

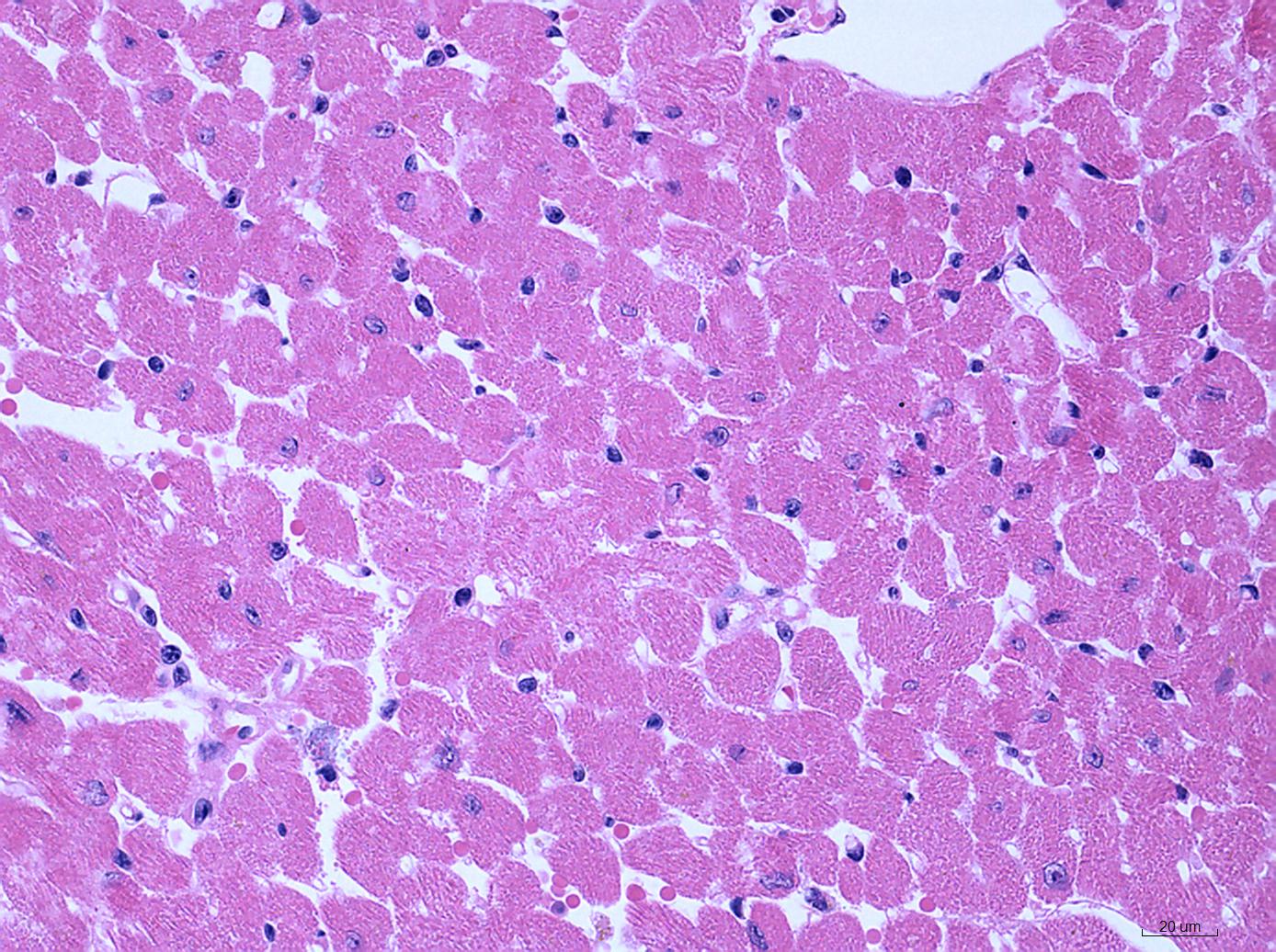

Supplement: Supplementary file 4 — Supporting File 4: advs73796‐sup‐0004‐Data.zip. [file ADVS-13-e21337-s003.zip › advs73796-sup-0004-Data/IHC_Raw_Data_Figures/Figure 9J_RawData_Figures/H&E-WT-AAV-NC-40X.jpg]

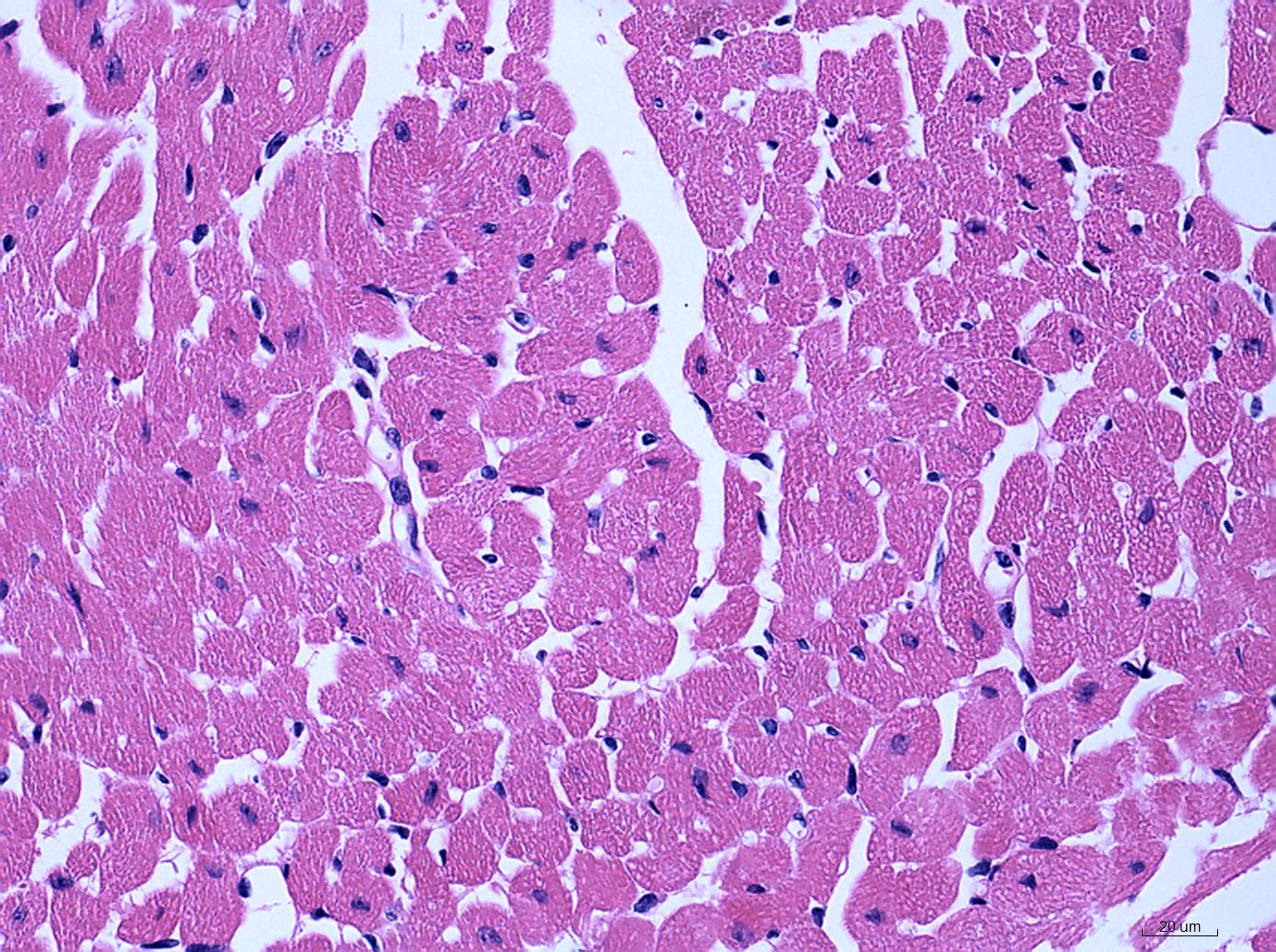

Supplement: Supplementary file 4 — Supporting File 4: advs73796‐sup‐0004‐Data.zip. [file ADVS-13-e21337-s003.zip › advs73796-sup-0004-Data/IHC_Raw_Data_Figures/Figure 9J_RawData_Figures/H&E-WT-AAV-TRIM40-40X.jpg]

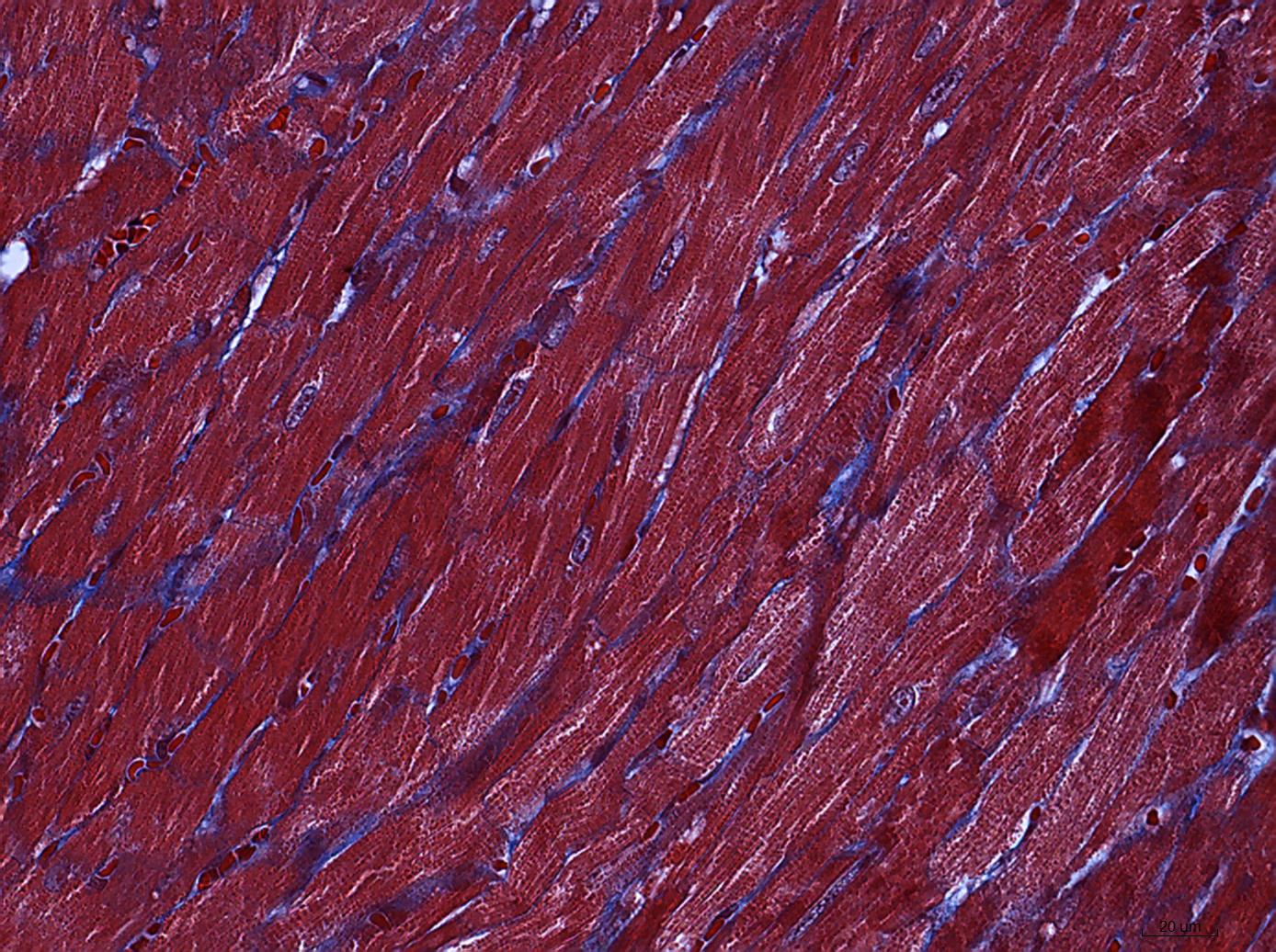

Supplement: Supplementary file 4 — Supporting File 4: advs73796‐sup‐0004‐Data.zip. [file ADVS-13-e21337-s003.zip › advs73796-sup-0004-Data/IHC_Raw_Data_Figures/Figure 9K_RawData_Figures/Masson-WT+TAC-AAV-NC-40X.jpg]

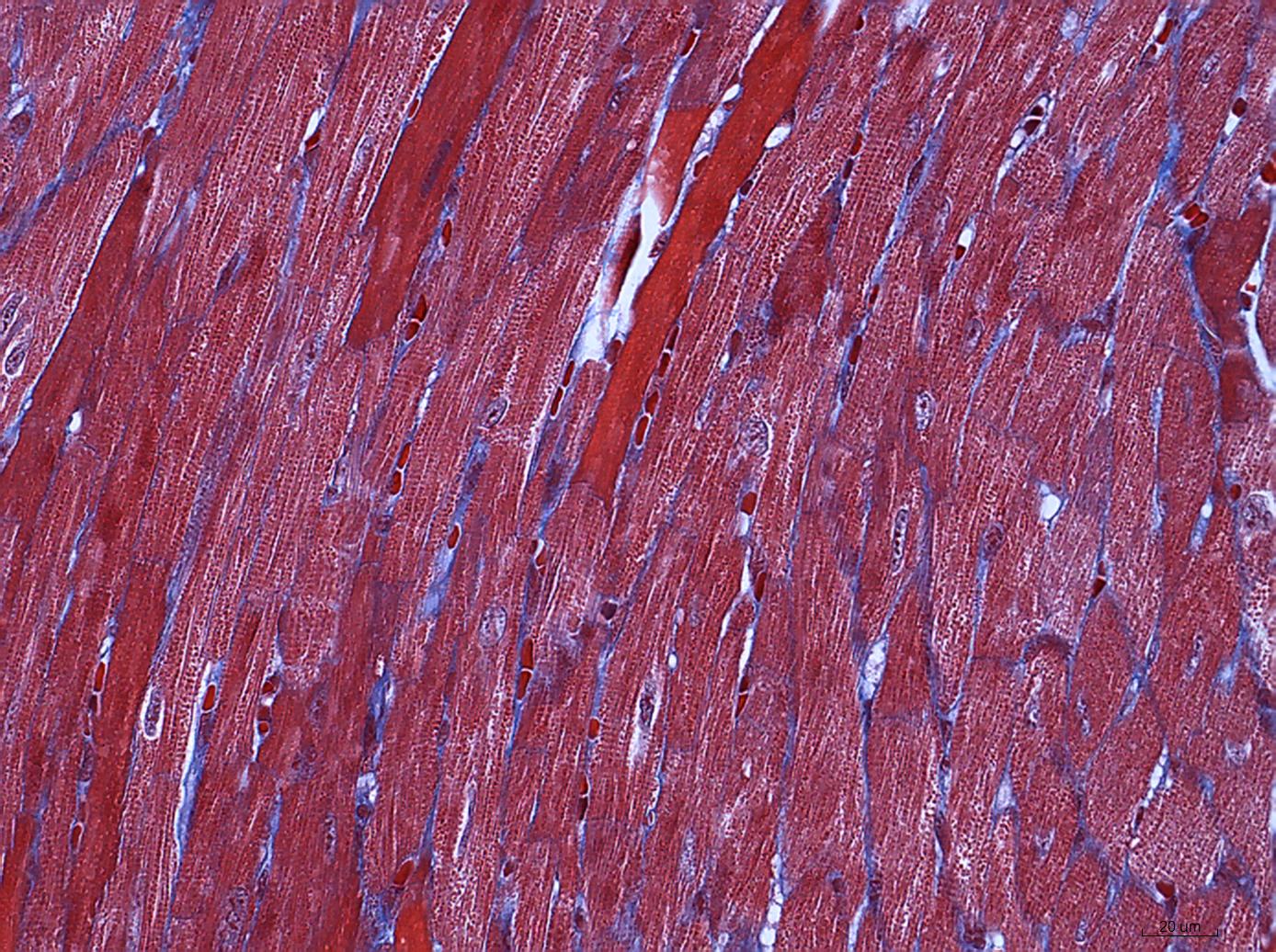

Supplement: Supplementary file 4 — Supporting File 4: advs73796‐sup‐0004‐Data.zip. [file ADVS-13-e21337-s003.zip › advs73796-sup-0004-Data/IHC_Raw_Data_Figures/Figure 9K_RawData_Figures/Masson-WT+TAC-AAV-TRIM40+PKN1 or 2-IN-1-40X.jpg]

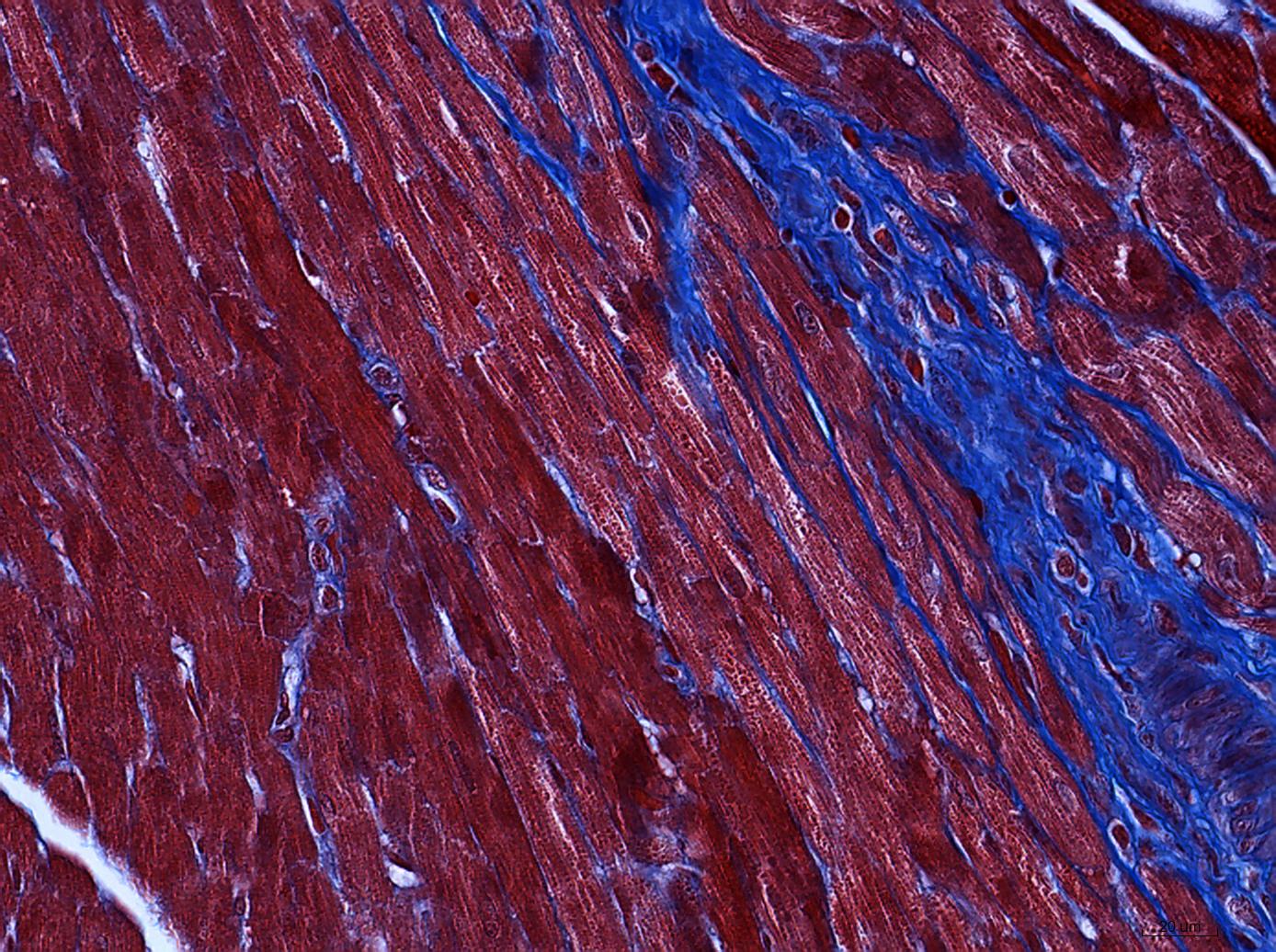

Supplement: Supplementary file 4 — Supporting File 4: advs73796‐sup‐0004‐Data.zip. [file ADVS-13-e21337-s003.zip › advs73796-sup-0004-Data/IHC_Raw_Data_Figures/Figure 9K_RawData_Figures/Masson-WT+TAC-AAV-TRIM40-40X.jpg]

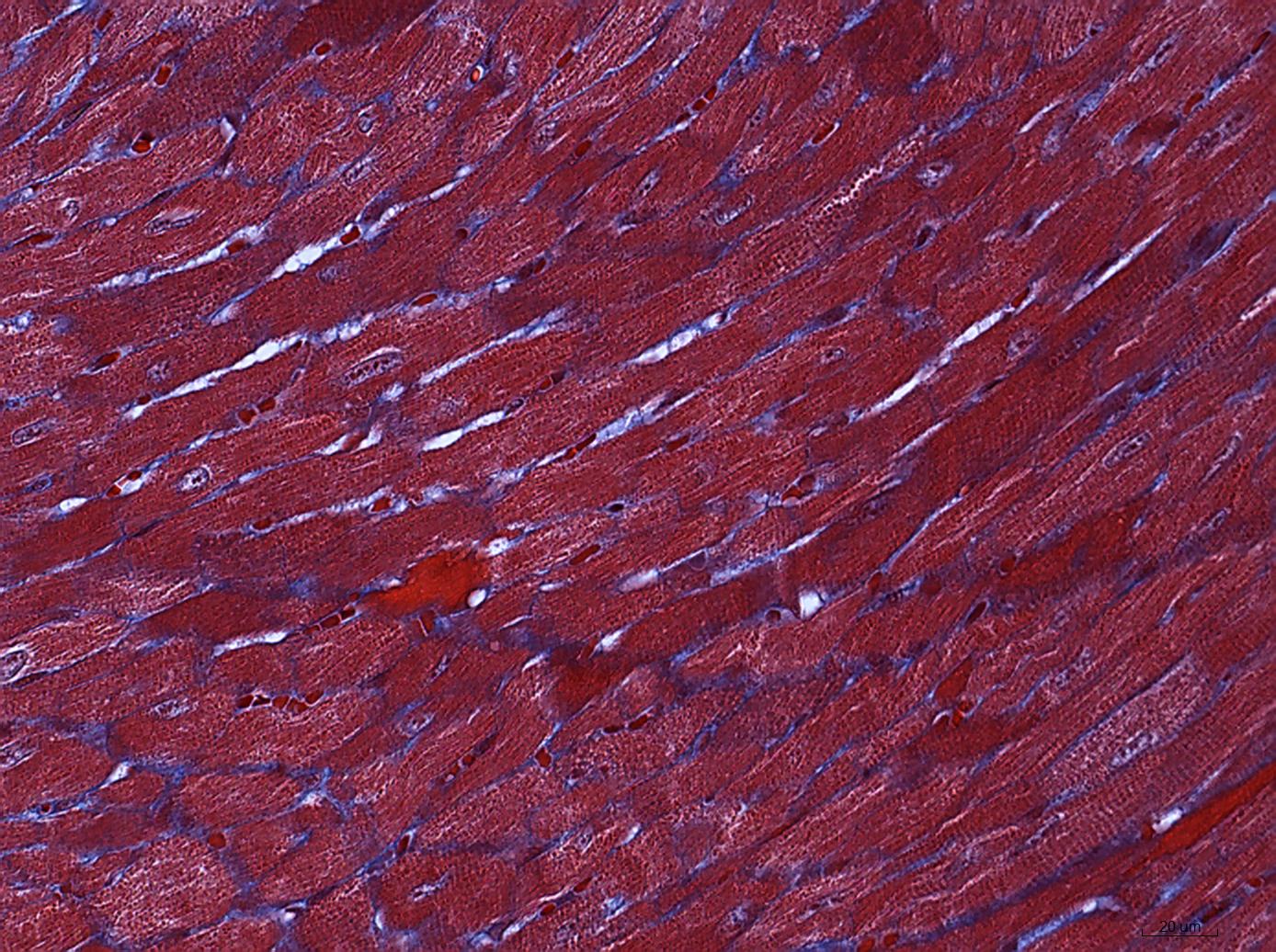

Supplement: Supplementary file 4 — Supporting File 4: advs73796‐sup‐0004‐Data.zip. [file ADVS-13-e21337-s003.zip › advs73796-sup-0004-Data/IHC_Raw_Data_Figures/Figure 9K_RawData_Figures/Masson-WT-AAV-NC-40X.jpg]

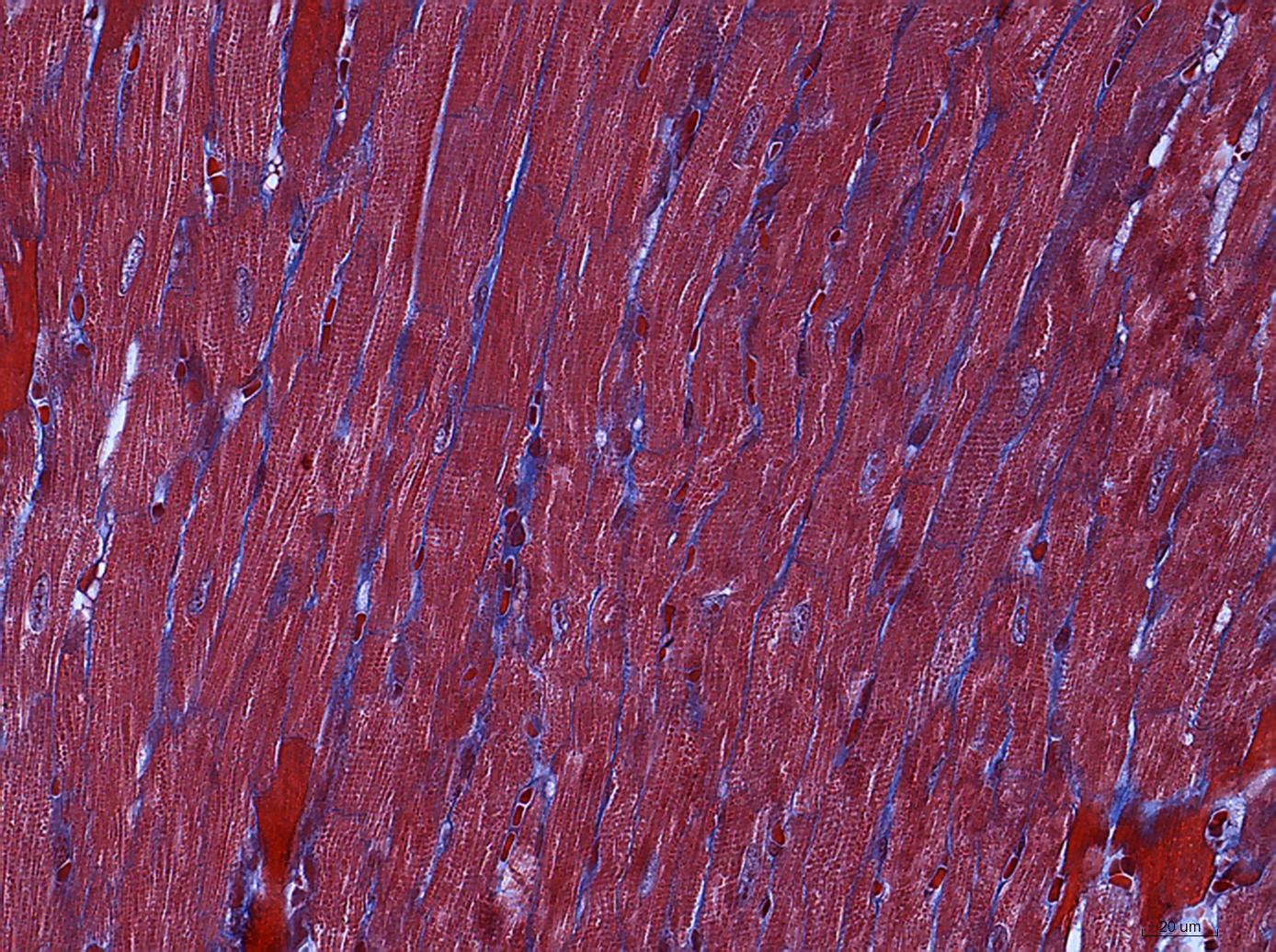

Supplement: Supplementary file 4 — Supporting File 4: advs73796‐sup‐0004‐Data.zip. [file ADVS-13-e21337-s003.zip › advs73796-sup-0004-Data/IHC_Raw_Data_Figures/Figure 9K_RawData_Figures/Masson-WT-AAV-TRIM40-40X.jpg]

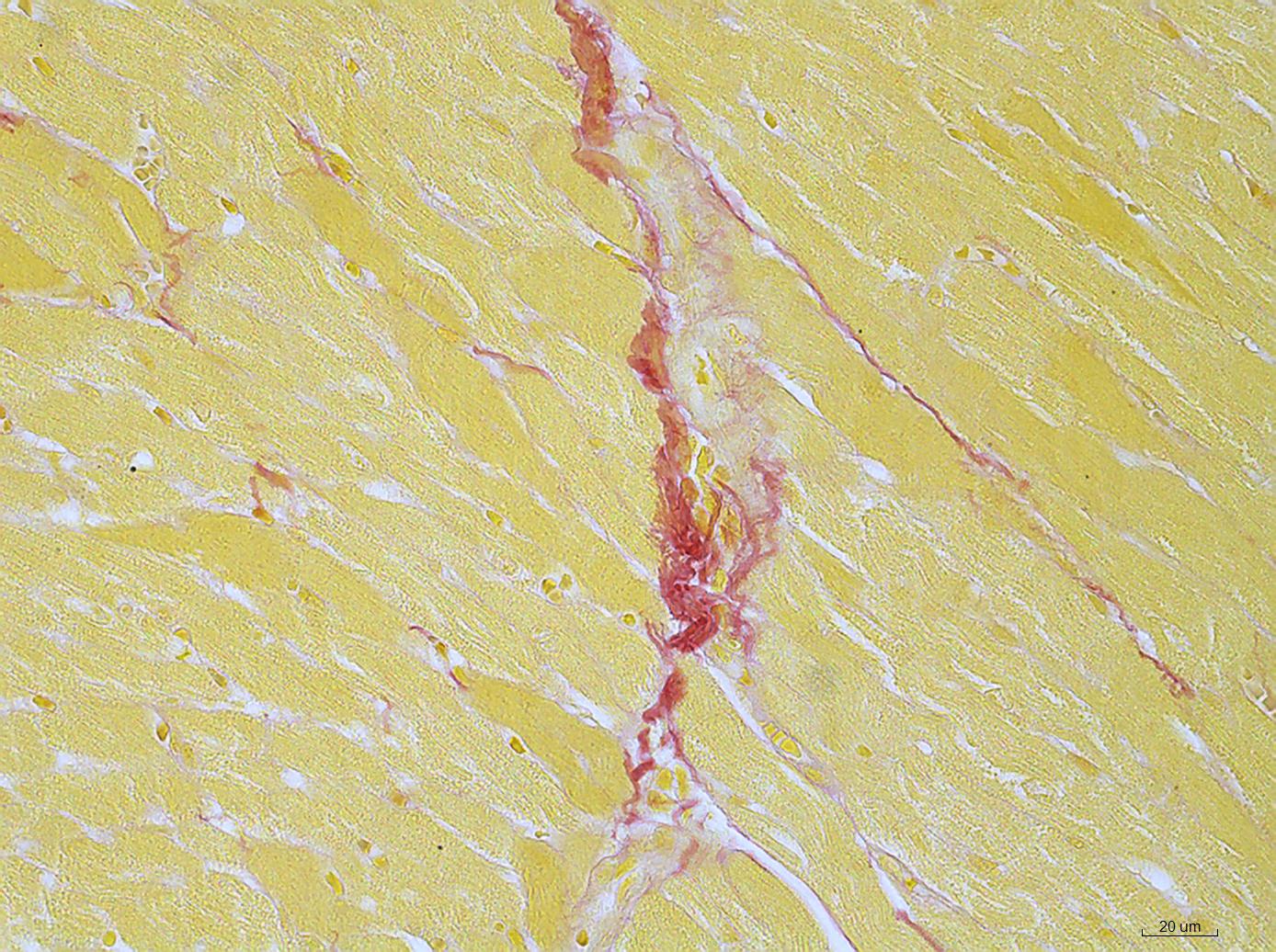

Supplement: Supplementary file 4 — Supporting File 4: advs73796‐sup‐0004‐Data.zip. [file ADVS-13-e21337-s003.zip › advs73796-sup-0004-Data/IHC_Raw_Data_Figures/Figure 9L_RawData_Figures/Sirius red-WT+TAC-AAV-NC-40X.jpg]

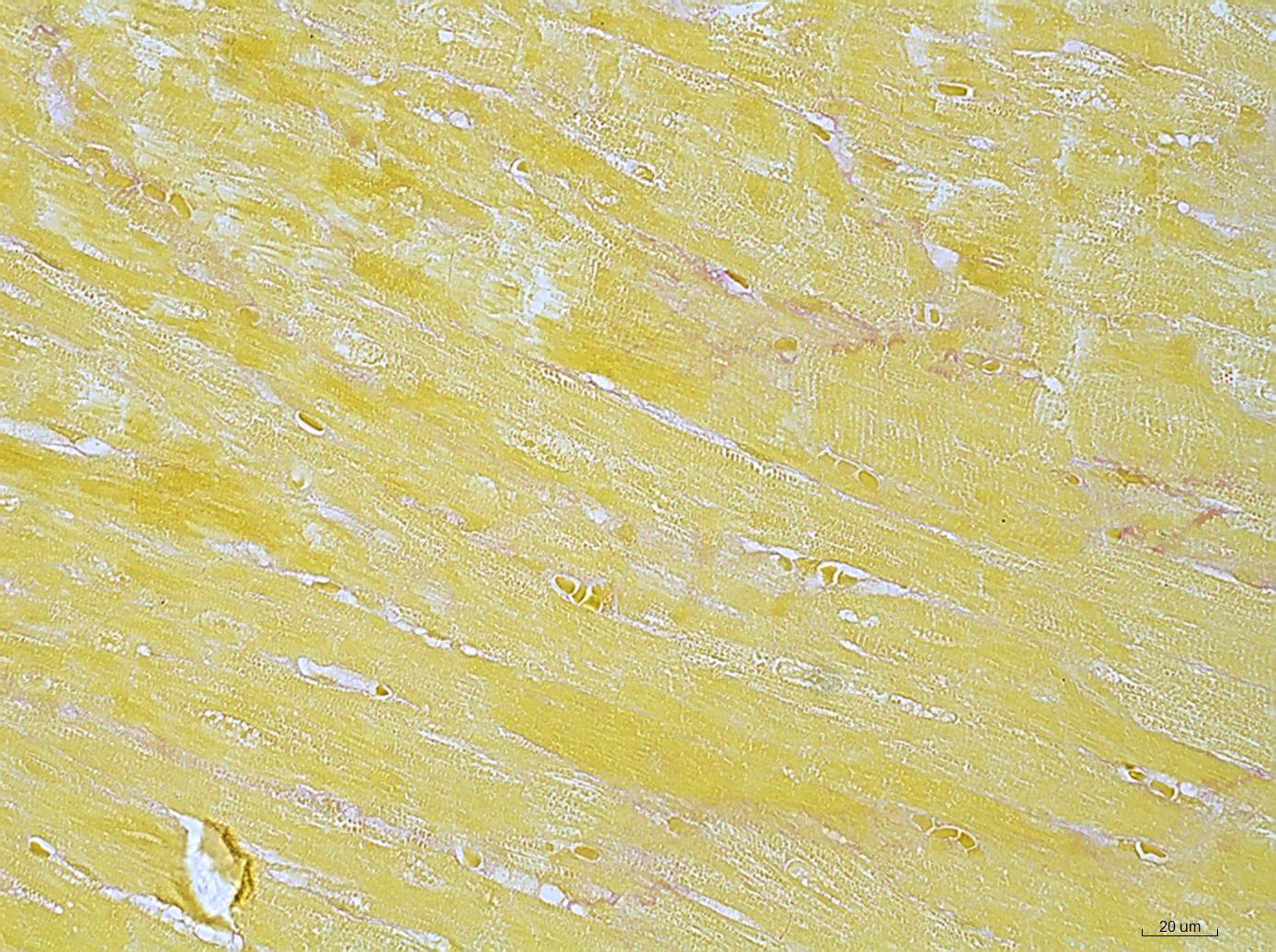

Supplement: Supplementary file 4 — Supporting File 4: advs73796‐sup‐0004‐Data.zip. [file ADVS-13-e21337-s003.zip › advs73796-sup-0004-Data/IHC_Raw_Data_Figures/Figure 9L_RawData_Figures/Sirius red-WT+TAC-AAV-TRIM40+PKN1 or 2-IN-1-40X.jpg]

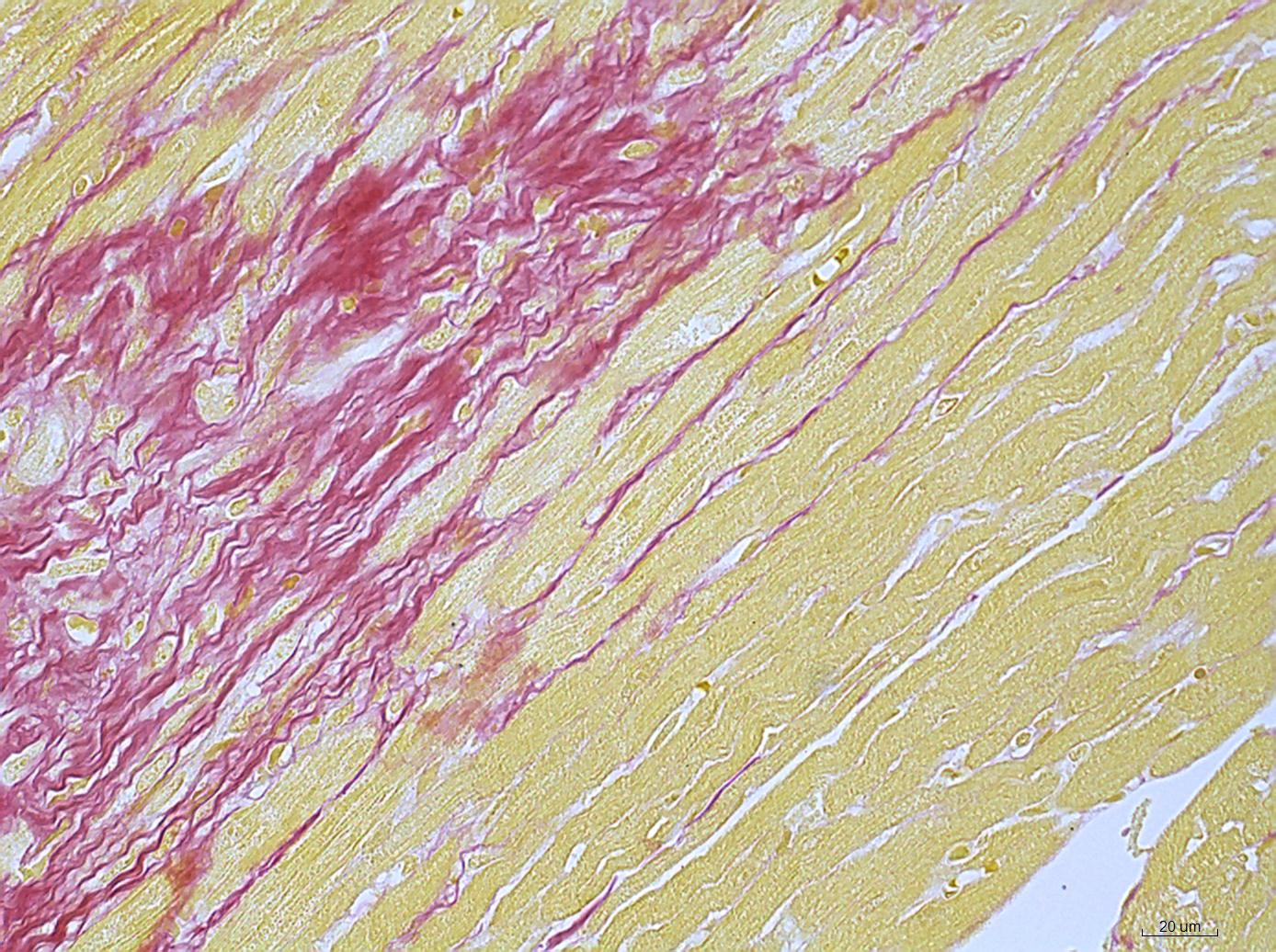

Supplement: Supplementary file 4 — Supporting File 4: advs73796‐sup‐0004‐Data.zip. [file ADVS-13-e21337-s003.zip › advs73796-sup-0004-Data/IHC_Raw_Data_Figures/Figure 9L_RawData_Figures/Sirius red-WT+TAC-AAV-TRIM40-40X.jpg]

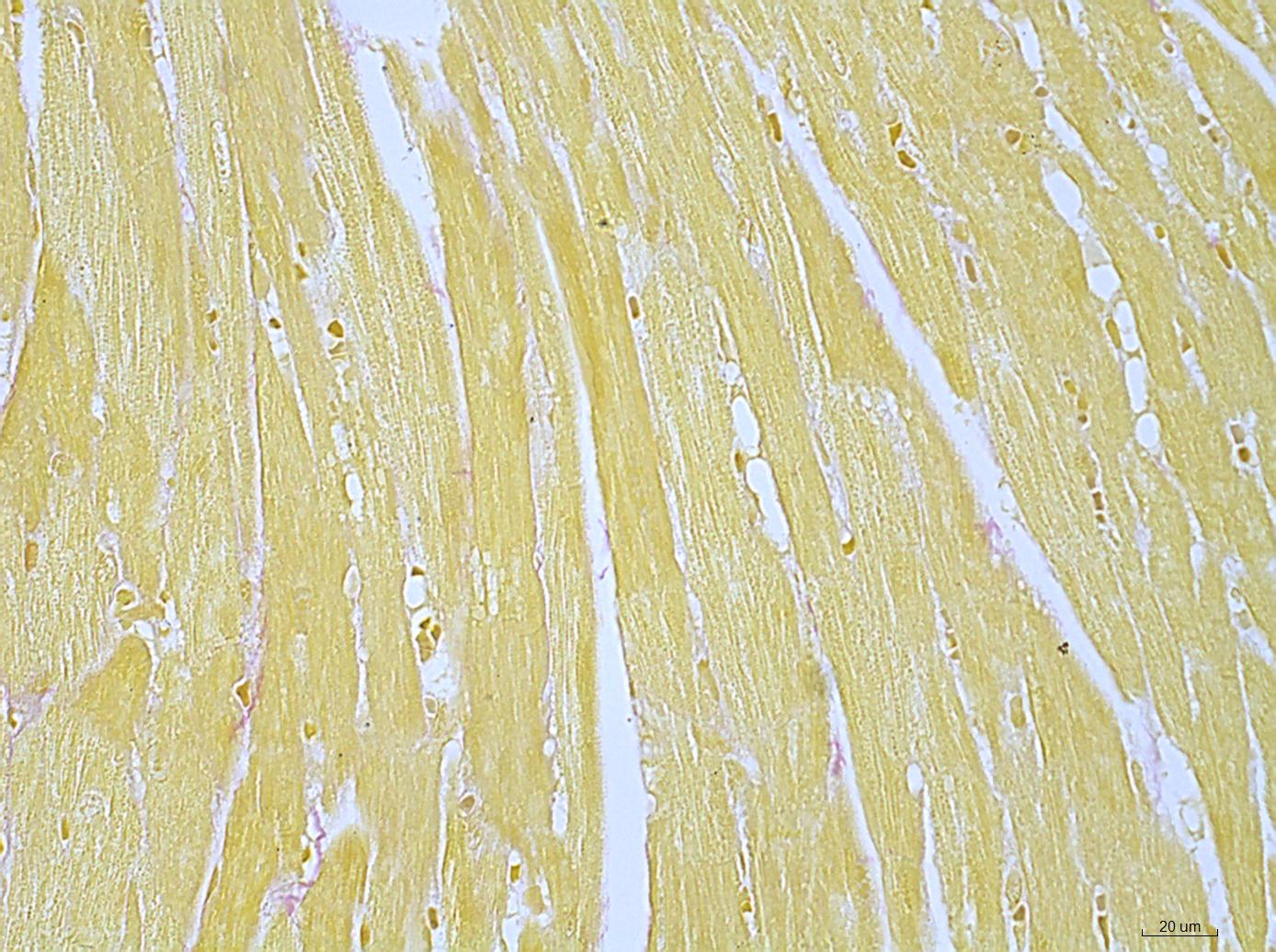

Supplement: Supplementary file 4 — Supporting File 4: advs73796‐sup‐0004‐Data.zip. [file ADVS-13-e21337-s003.zip › advs73796-sup-0004-Data/IHC_Raw_Data_Figures/Figure 9L_RawData_Figures/Sirius red-WT-AAV-NC-40X.jpg]

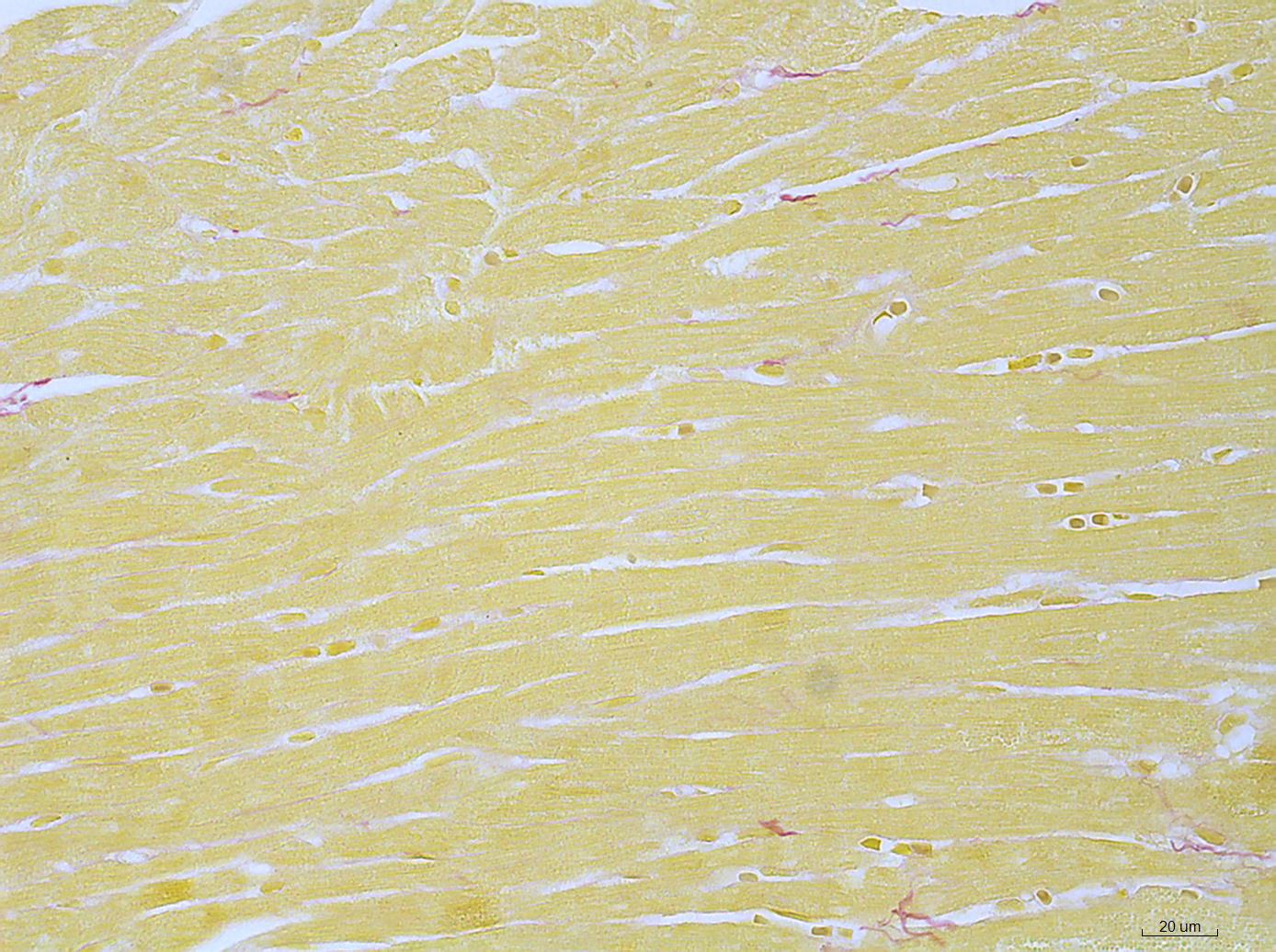

Supplement: Supplementary file 4 — Supporting File 4: advs73796‐sup‐0004‐Data.zip. [file ADVS-13-e21337-s003.zip › advs73796-sup-0004-Data/IHC_Raw_Data_Figures/Figure 9L_RawData_Figures/Sirius red-WT-AAV-TRIM40-40X.jpg]

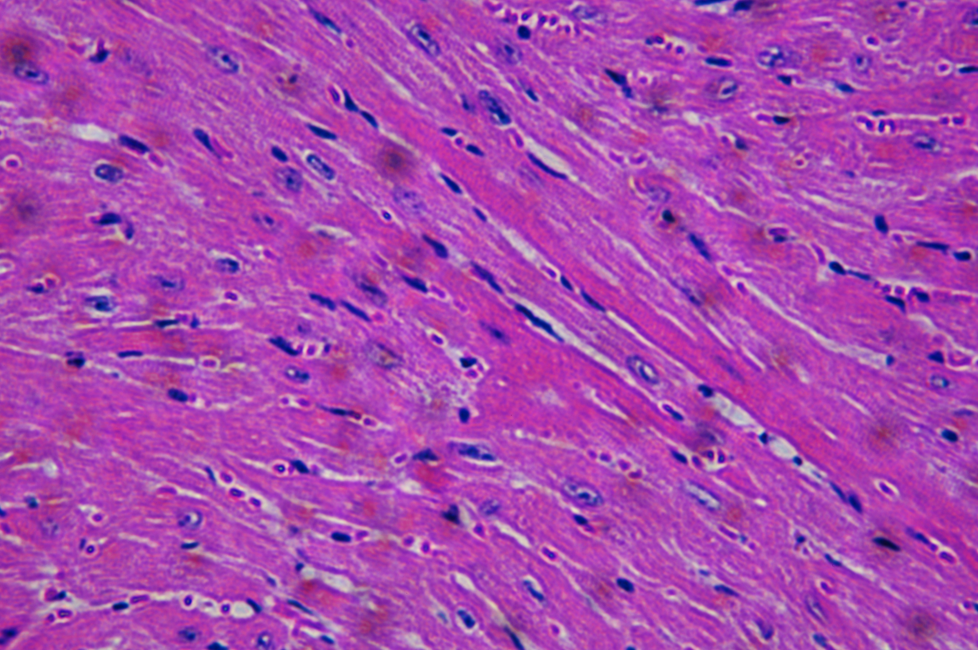

Supplement: Supplementary file 4 — Supporting File 4: advs73796‐sup‐0004‐Data.zip. [file ADVS-13-e21337-s003.zip › advs73796-sup-0004-Data/IHC_Raw_Data_Figures/Figure S2E_RawData_Figures/H&E-TRIM40 knockout-Ang II -40X.tif]

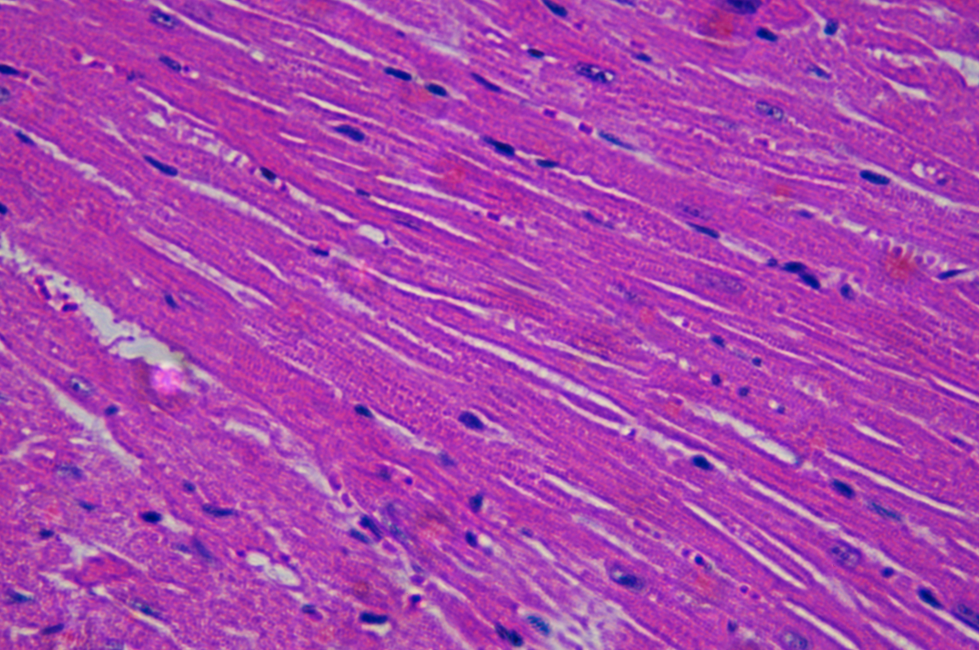

Supplement: Supplementary file 4 — Supporting File 4: advs73796‐sup‐0004‐Data.zip. [file ADVS-13-e21337-s003.zip › advs73796-sup-0004-Data/IHC_Raw_Data_Figures/Figure S2E_RawData_Figures/H&E-TRIM40 knockout-Sham-40X.tif]

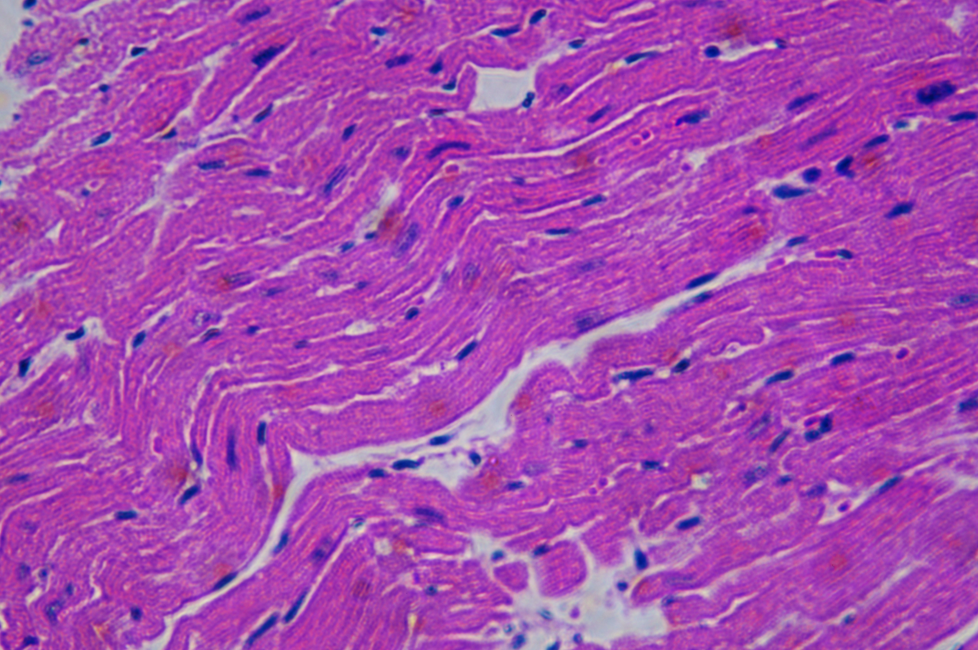

Supplement: Supplementary file 4 — Supporting File 4: advs73796‐sup‐0004‐Data.zip. [file ADVS-13-e21337-s003.zip › advs73796-sup-0004-Data/IHC_Raw_Data_Figures/Figure S2E_RawData_Figures/H&E-WT-Ang II-40X.tif]

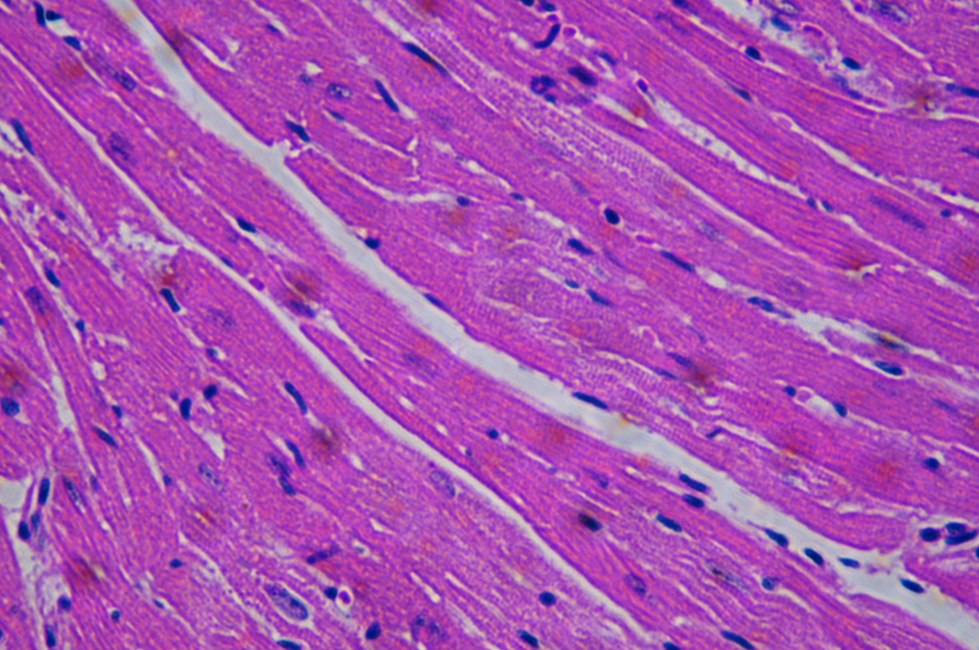

Supplement: Supplementary file 4 — Supporting File 4: advs73796‐sup‐0004‐Data.zip. [file ADVS-13-e21337-s003.zip › advs73796-sup-0004-Data/IHC_Raw_Data_Figures/Figure S2E_RawData_Figures/H&E-WT-Sham-40X.tif]

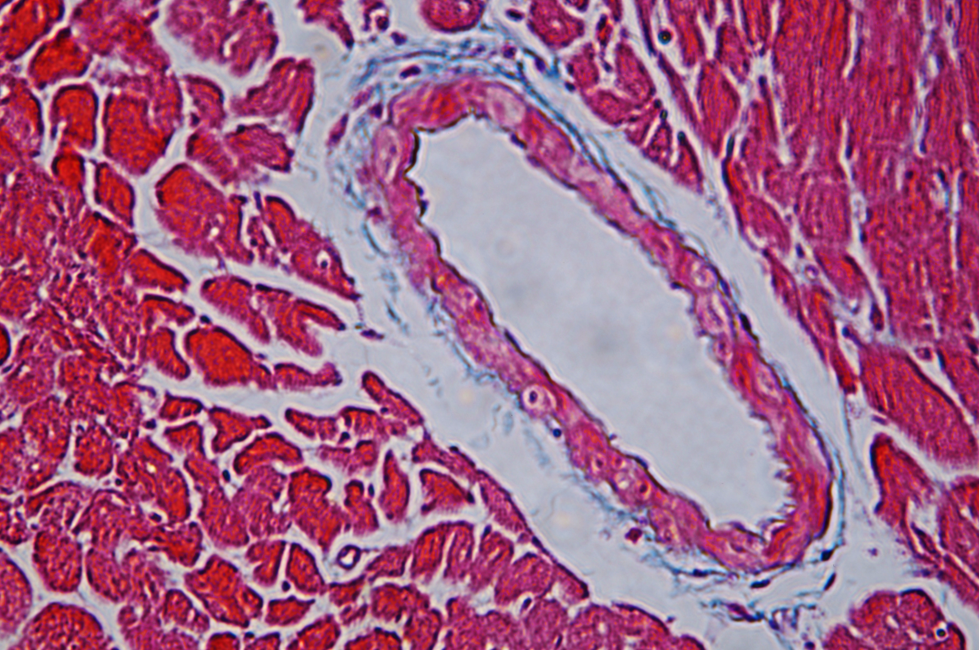

Supplement: Supplementary file 4 — Supporting File 4: advs73796‐sup‐0004‐Data.zip. [file ADVS-13-e21337-s003.zip › advs73796-sup-0004-Data/IHC_Raw_Data_Figures/Figure S2G_RawData_Figures/Masson-TRIM40 knockout-Ang II-40X.tif]

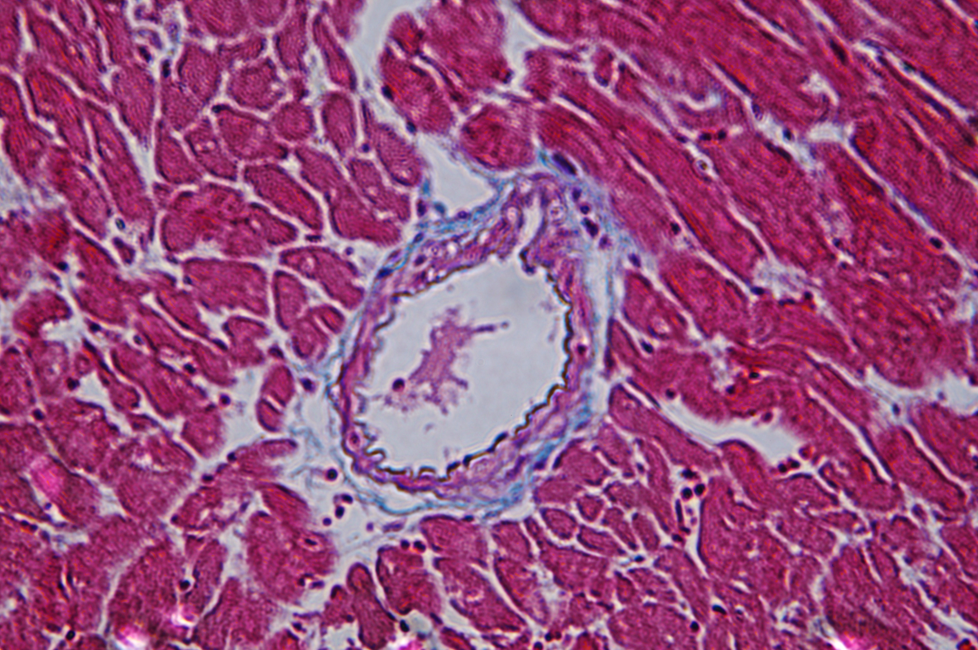

Supplement: Supplementary file 4 — Supporting File 4: advs73796‐sup‐0004‐Data.zip. [file ADVS-13-e21337-s003.zip › advs73796-sup-0004-Data/IHC_Raw_Data_Figures/Figure S2G_RawData_Figures/Masson-TRIM40 knockout-Sham-40X.tif]

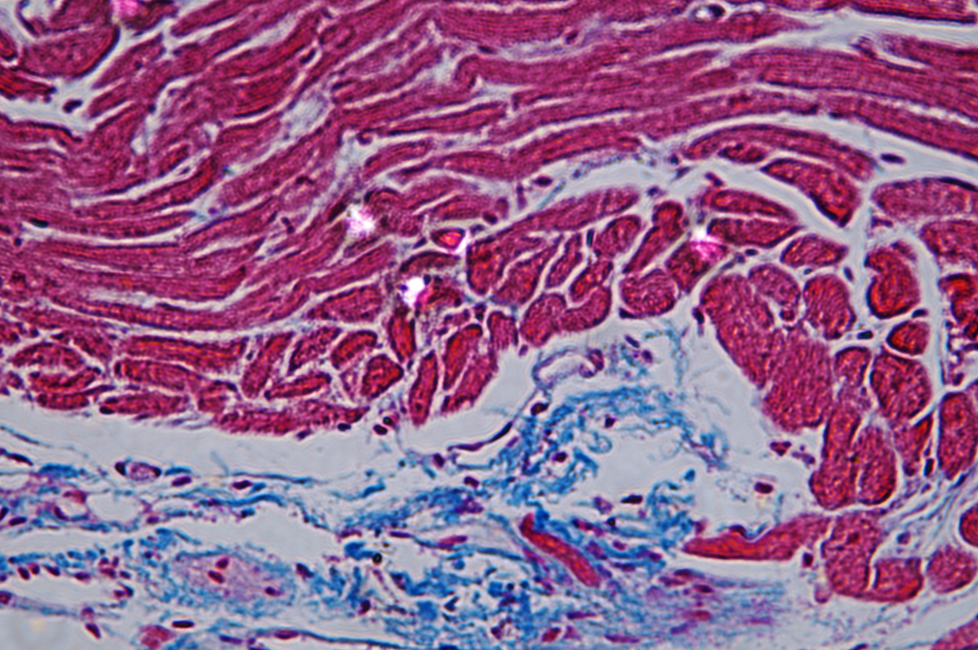

Supplement: Supplementary file 4 — Supporting File 4: advs73796‐sup‐0004‐Data.zip. [file ADVS-13-e21337-s003.zip › advs73796-sup-0004-Data/IHC_Raw_Data_Figures/Figure S2G_RawData_Figures/Masson-WT-Ang II-40X.tif]

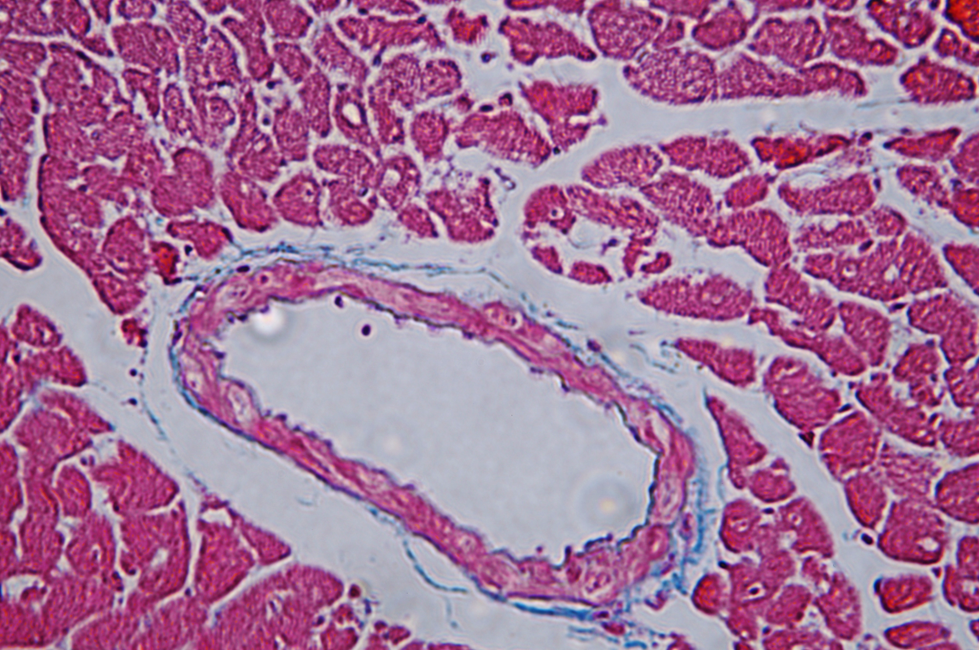

Supplement: Supplementary file 4 — Supporting File 4: advs73796‐sup‐0004‐Data.zip. [file ADVS-13-e21337-s003.zip › advs73796-sup-0004-Data/IHC_Raw_Data_Figures/Figure S2G_RawData_Figures/Masson-WT-Sham-40X.tif]

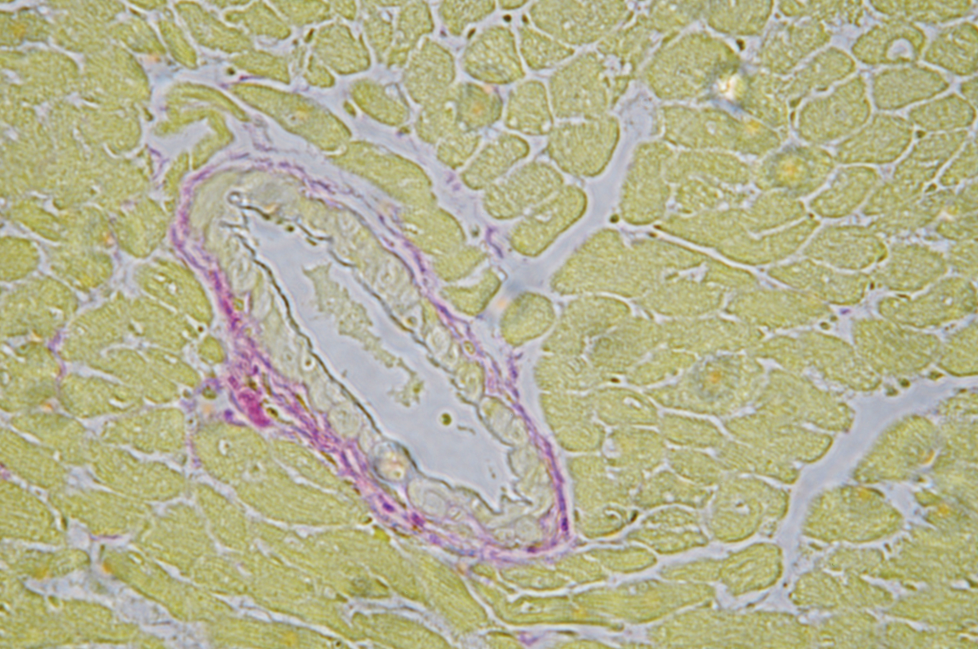

Supplement: Supplementary file 4 — Supporting File 4: advs73796‐sup‐0004‐Data.zip. [file ADVS-13-e21337-s003.zip › advs73796-sup-0004-Data/IHC_Raw_Data_Figures/Figure S2J_RawData_Figures/Sirius red-TRIM40 knockout-Ang II-40X.tif]

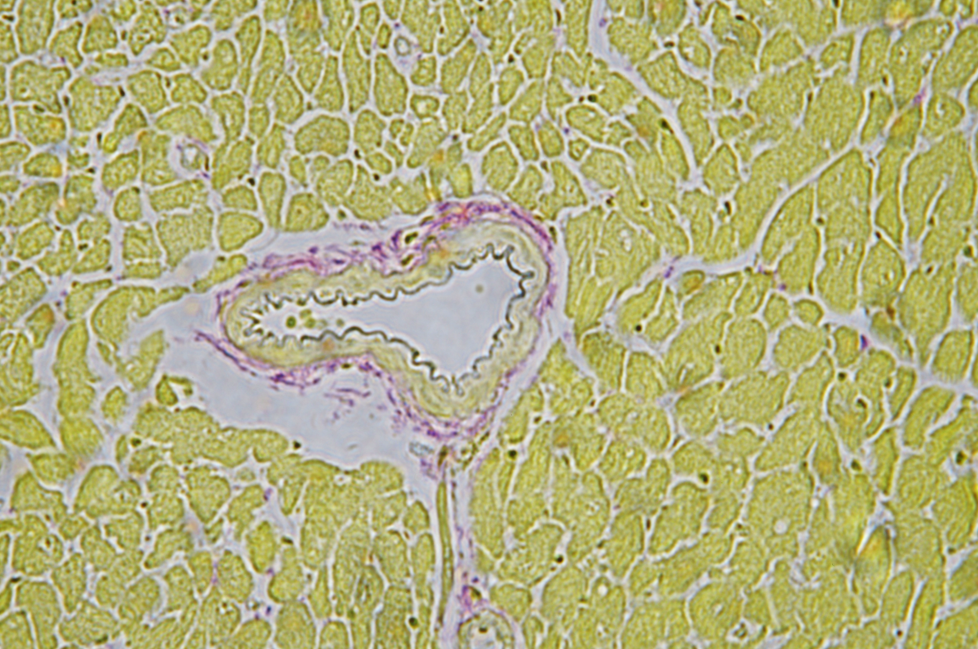

Supplement: Supplementary file 4 — Supporting File 4: advs73796‐sup‐0004‐Data.zip. [file ADVS-13-e21337-s003.zip › advs73796-sup-0004-Data/IHC_Raw_Data_Figures/Figure S2J_RawData_Figures/Sirius red-TRIM40 knockout-Sham-40X.tif]

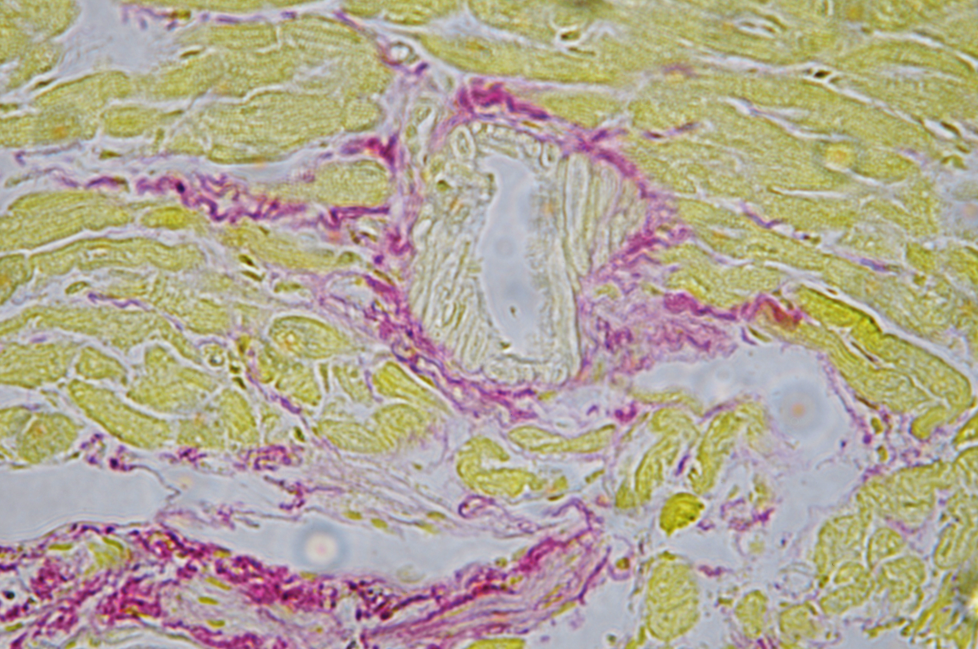

Supplement: Supplementary file 4 — Supporting File 4: advs73796‐sup‐0004‐Data.zip. [file ADVS-13-e21337-s003.zip › advs73796-sup-0004-Data/IHC_Raw_Data_Figures/Figure S2J_RawData_Figures/Sirius red-WT-Ang II-40X.tif]

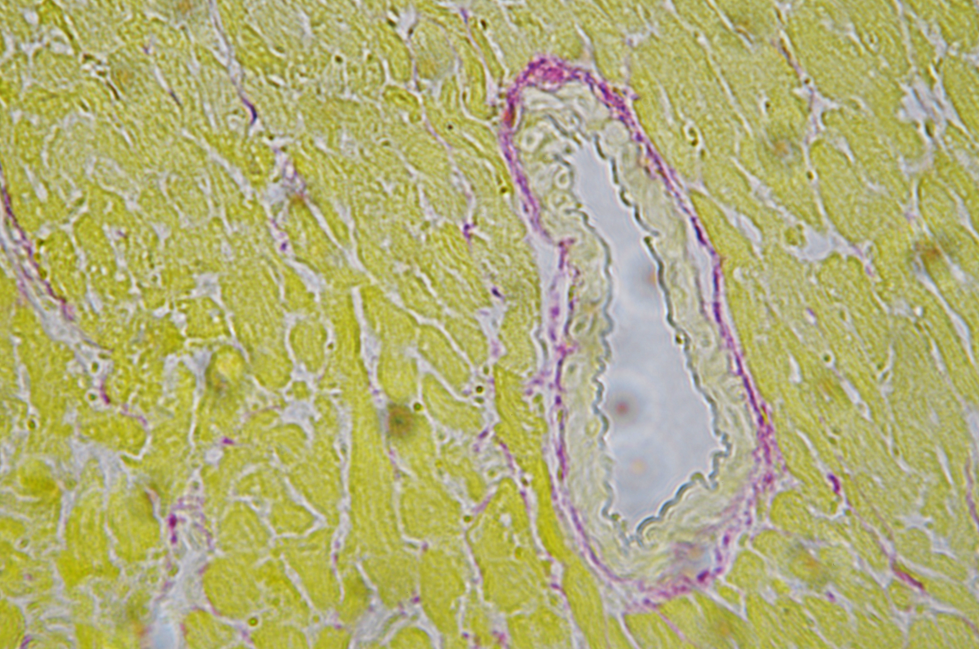

Supplement: Supplementary file 4 — Supporting File 4: advs73796‐sup‐0004‐Data.zip. [file ADVS-13-e21337-s003.zip › advs73796-sup-0004-Data/IHC_Raw_Data_Figures/Figure S2J_RawData_Figures/Sirius red-WT-Sham-40X.tif]

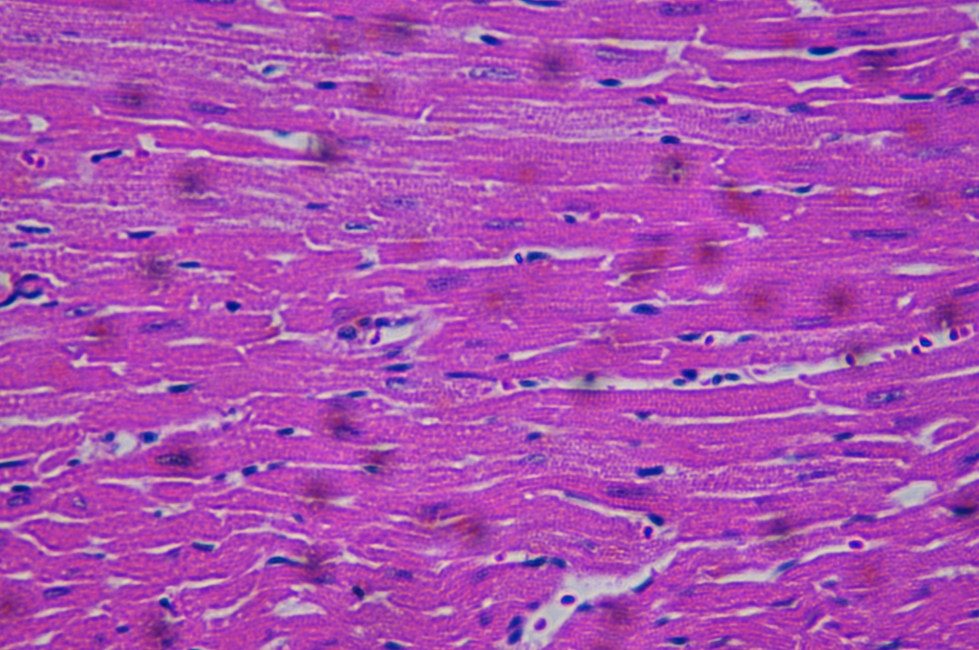

Supplement: Supplementary file 4 — Supporting File 4: advs73796‐sup‐0004‐Data.zip. [file ADVS-13-e21337-s003.zip › advs73796-sup-0004-Data/IHC_Raw_Data_Figures/Figure S3C_RawData_Figures/H&E-TRIM40 knockout-Sham-40X.tif]

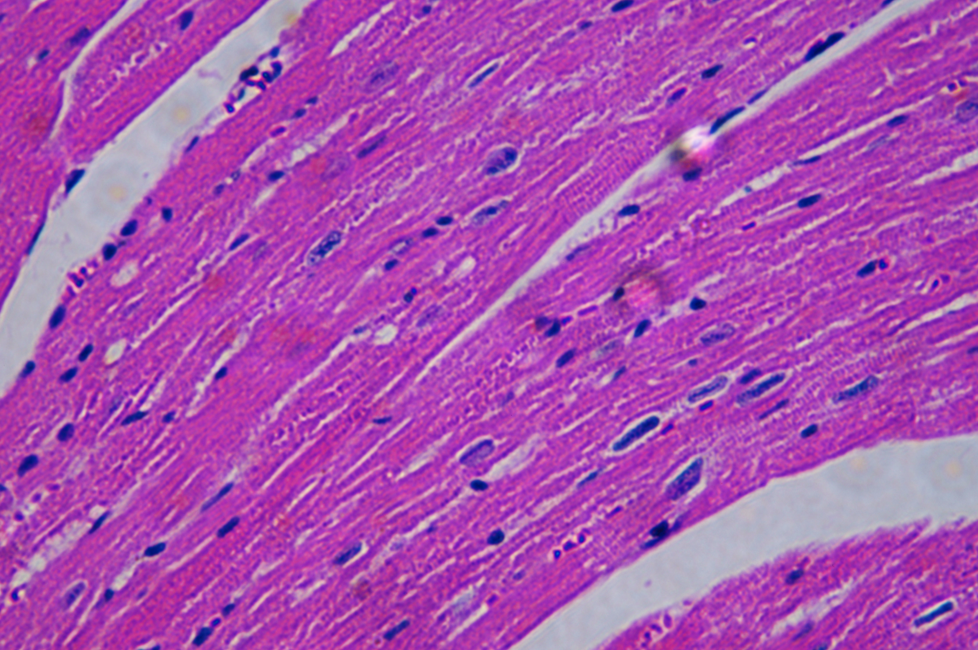

Supplement: Supplementary file 4 — Supporting File 4: advs73796‐sup‐0004‐Data.zip. [file ADVS-13-e21337-s003.zip › advs73796-sup-0004-Data/IHC_Raw_Data_Figures/Figure S3C_RawData_Figures/H&E-TRIM40 knockout-TAC-40X.tif]

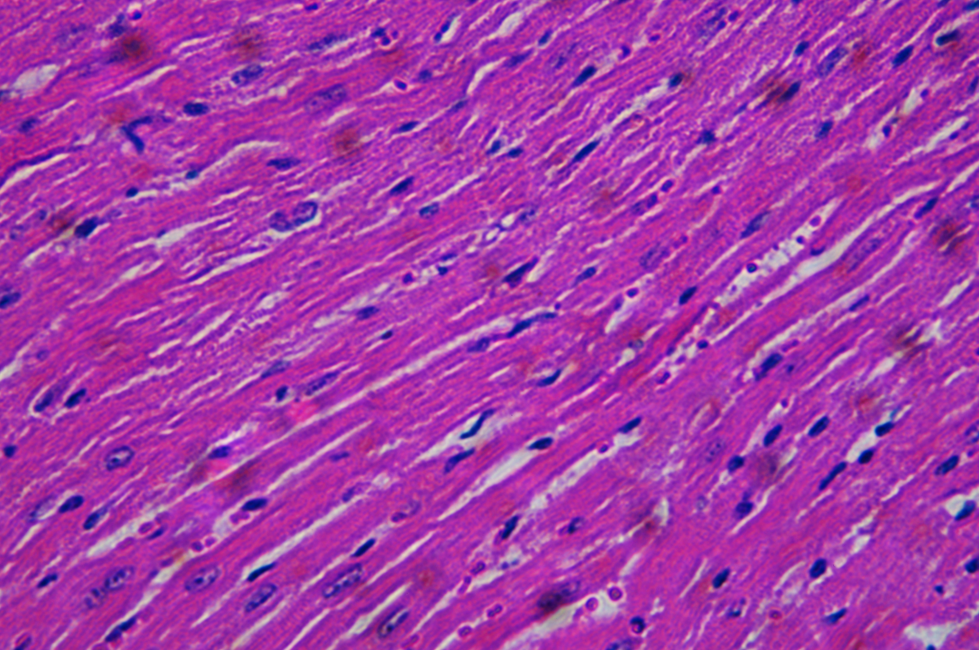

Supplement: Supplementary file 4 — Supporting File 4: advs73796‐sup‐0004‐Data.zip. [file ADVS-13-e21337-s003.zip › advs73796-sup-0004-Data/IHC_Raw_Data_Figures/Figure S3C_RawData_Figures/H&E-WT-Sham-40X.tif]

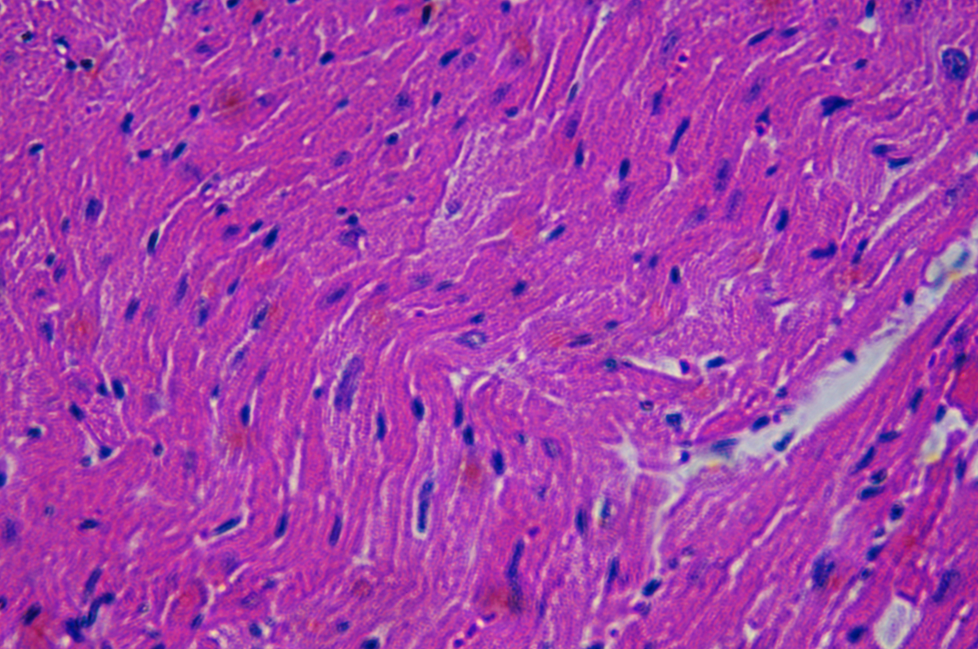

Supplement: Supplementary file 4 — Supporting File 4: advs73796‐sup‐0004‐Data.zip. [file ADVS-13-e21337-s003.zip › advs73796-sup-0004-Data/IHC_Raw_Data_Figures/Figure S3C_RawData_Figures/H&E-WT-TAC-40X.tif]

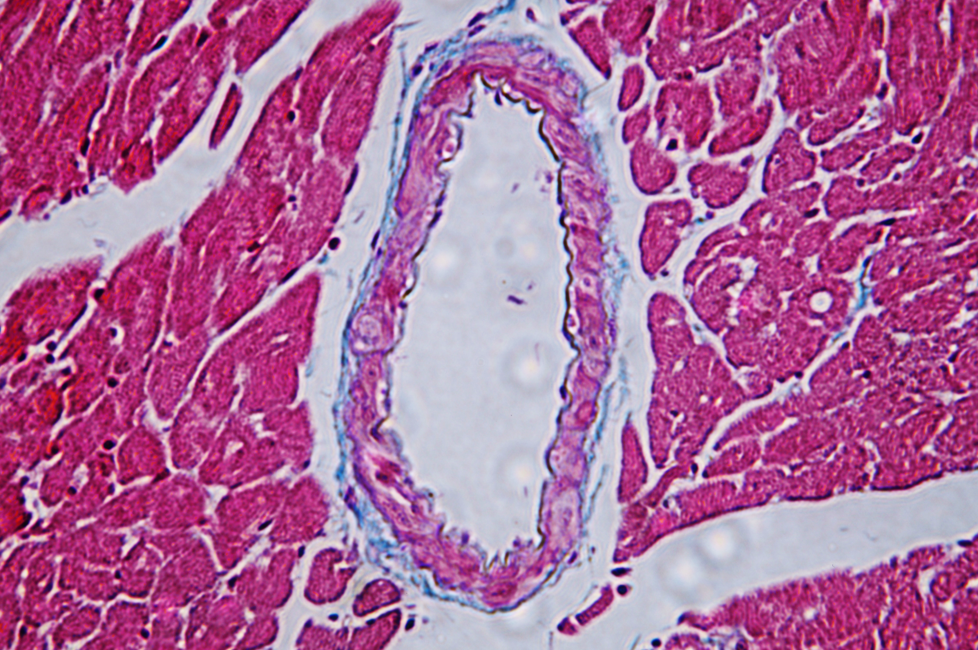

Supplement: Supplementary file 4 — Supporting File 4: advs73796‐sup‐0004‐Data.zip. [file ADVS-13-e21337-s003.zip › advs73796-sup-0004-Data/IHC_Raw_Data_Figures/Figure S3E_RawData_Figures/Masson-TRIM40 knockout-Sham-40X.tif]

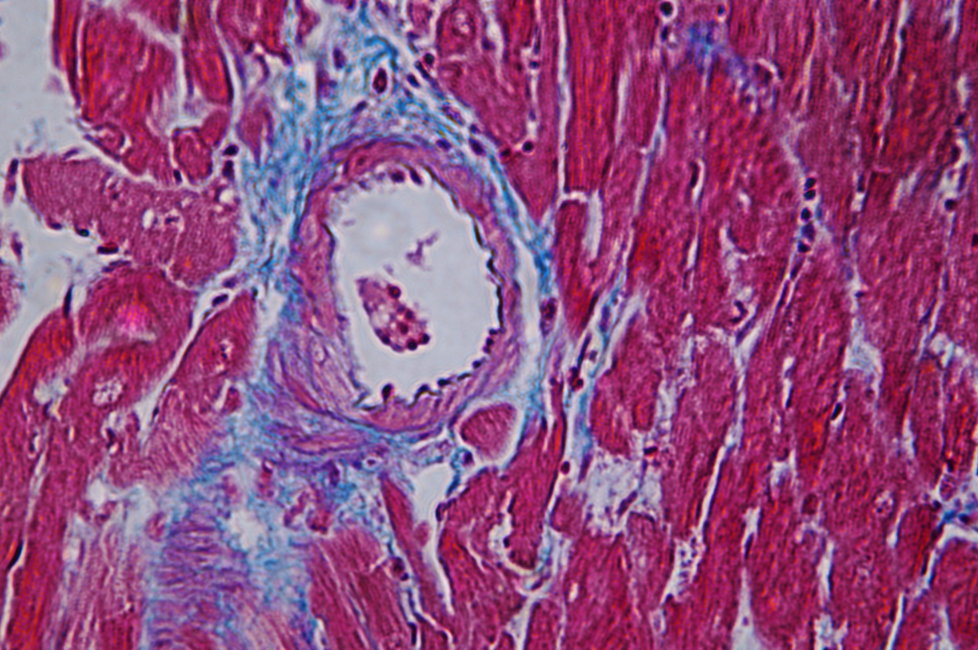

Supplement: Supplementary file 4 — Supporting File 4: advs73796‐sup‐0004‐Data.zip. [file ADVS-13-e21337-s003.zip › advs73796-sup-0004-Data/IHC_Raw_Data_Figures/Figure S3E_RawData_Figures/Masson-TRIM40 knockout-TAC-40X.tif]

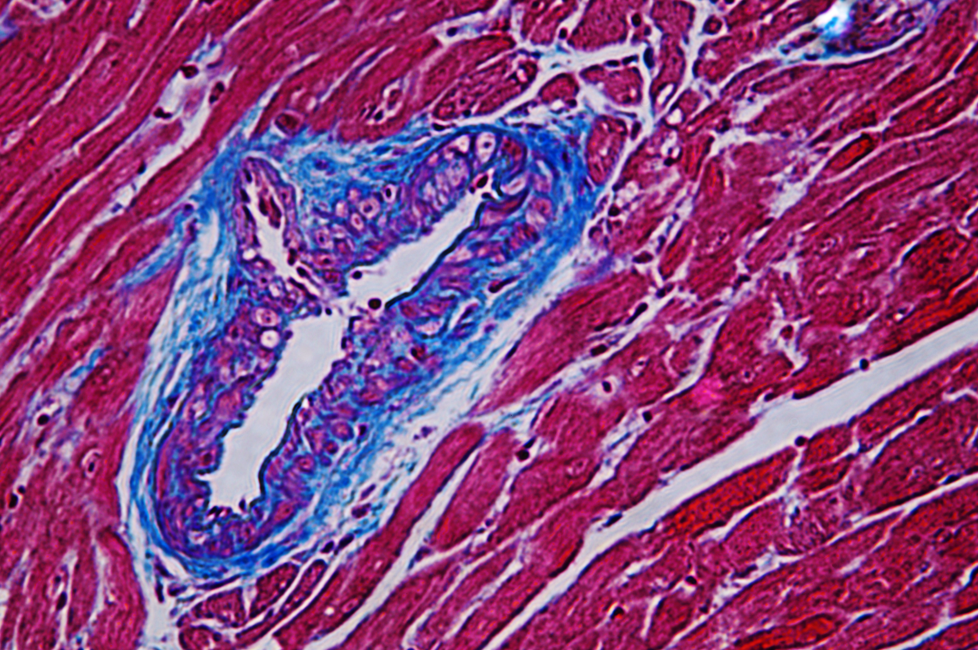

Supplement: Supplementary file 4 — Supporting File 4: advs73796‐sup‐0004‐Data.zip. [file ADVS-13-e21337-s003.zip › advs73796-sup-0004-Data/IHC_Raw_Data_Figures/Figure S3E_RawData_Figures/Masson-WT-TAC-40X.tif]

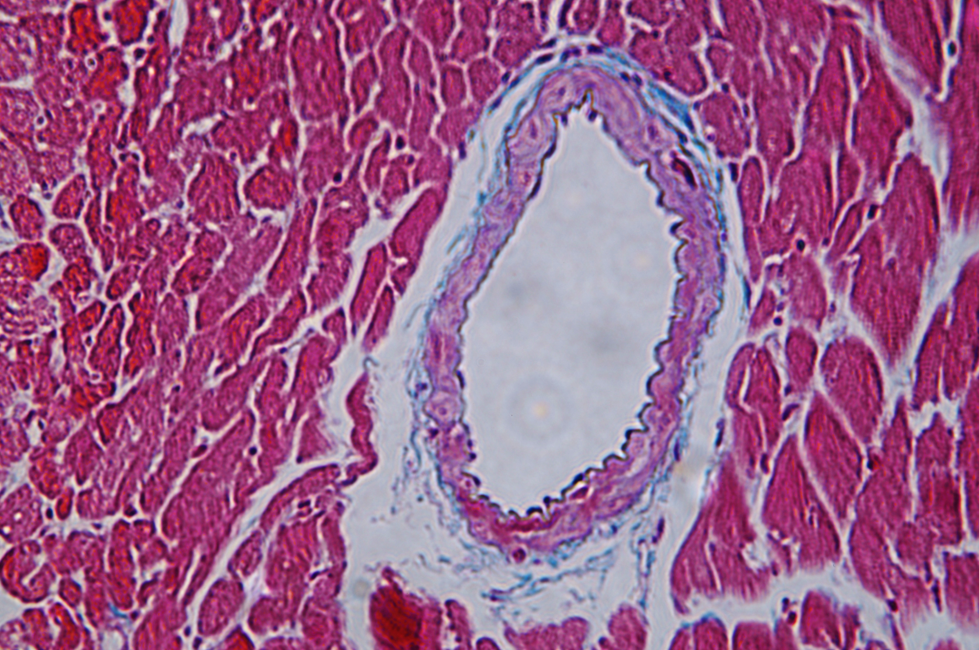

Supplement: Supplementary file 4 — Supporting File 4: advs73796‐sup‐0004‐Data.zip. [file ADVS-13-e21337-s003.zip › advs73796-sup-0004-Data/IHC_Raw_Data_Figures/Figure S3E_RawData_Figures/Msson-WT-Sham-40X.tif]

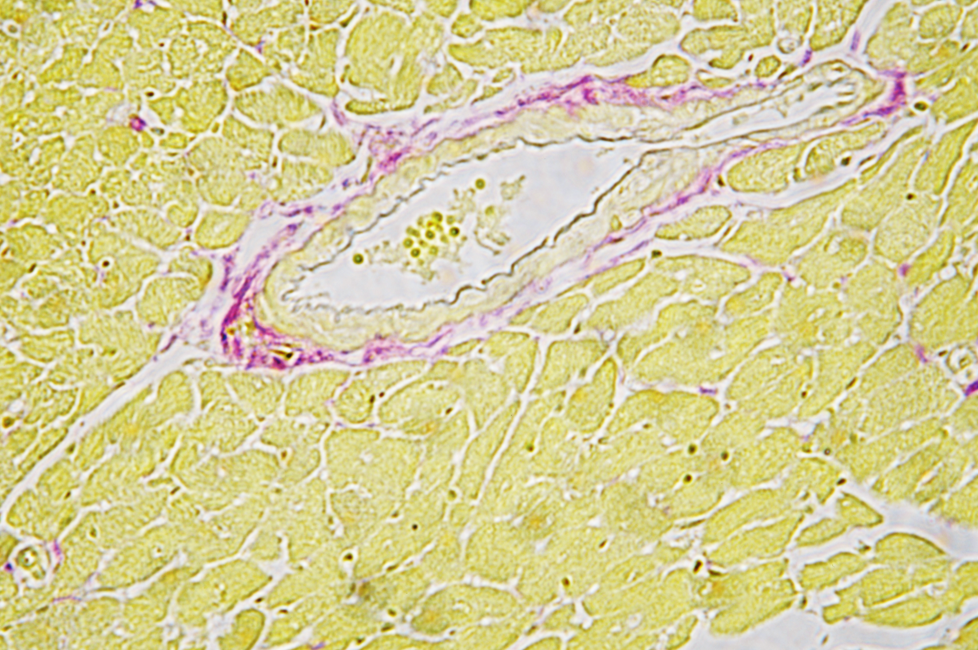

Supplement: Supplementary file 4 — Supporting File 4: advs73796‐sup‐0004‐Data.zip. [file ADVS-13-e21337-s003.zip › advs73796-sup-0004-Data/IHC_Raw_Data_Figures/Figure S3H_RawData_Figures/Sirius red-TRIM40 knockout-Sham-40X.tif]

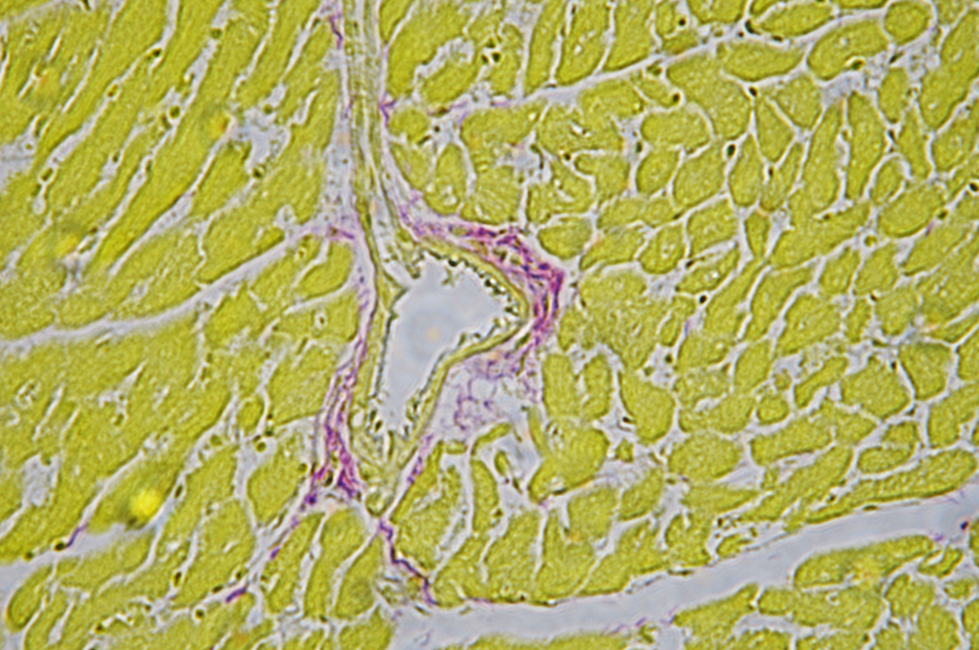

Supplement: Supplementary file 4 — Supporting File 4: advs73796‐sup‐0004‐Data.zip. [file ADVS-13-e21337-s003.zip › advs73796-sup-0004-Data/IHC_Raw_Data_Figures/Figure S3H_RawData_Figures/Sirius red-WT-Sham-40X.tif]

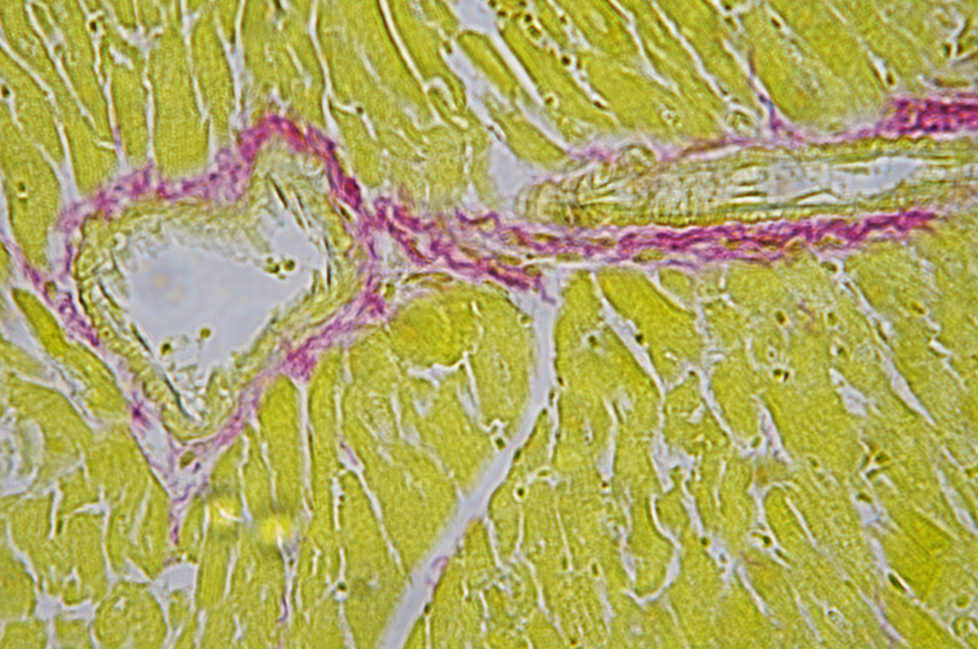

Supplement: Supplementary file 4 — Supporting File 4: advs73796‐sup‐0004‐Data.zip. [file ADVS-13-e21337-s003.zip › advs73796-sup-0004-Data/IHC_Raw_Data_Figures/Figure S3H_RawData_Figures/Sirius red-WT-TAC-40X.tif]

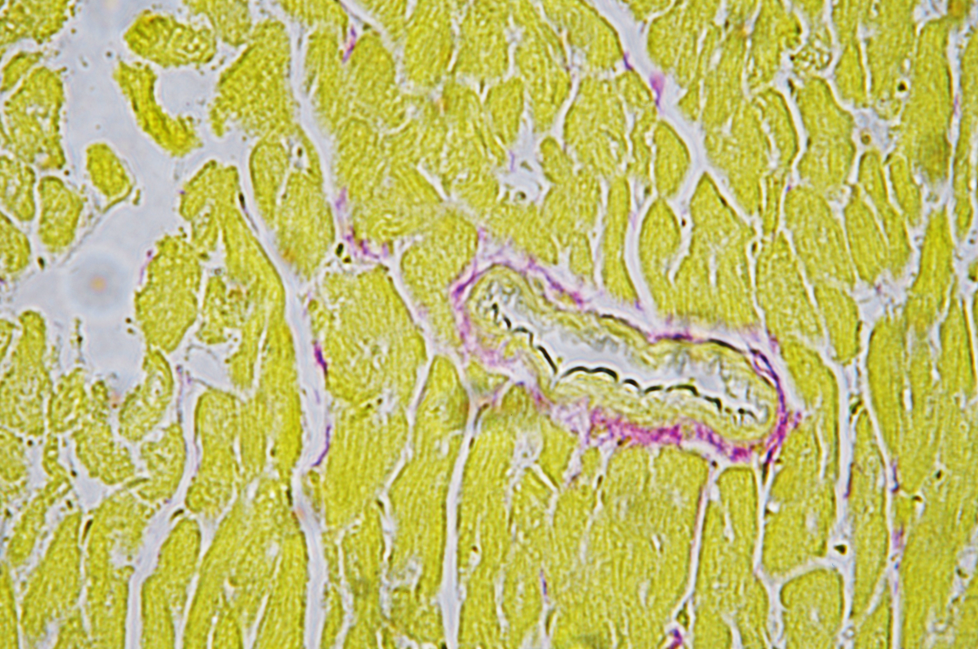

Supplement: Supplementary file 4 — Supporting File 4: advs73796‐sup‐0004‐Data.zip. [file ADVS-13-e21337-s003.zip › advs73796-sup-0004-Data/IHC_Raw_Data_Figures/Figure S3H_RawData_Figures/Sirrius red-TRIM40 knockout-TAC-40X.tif]

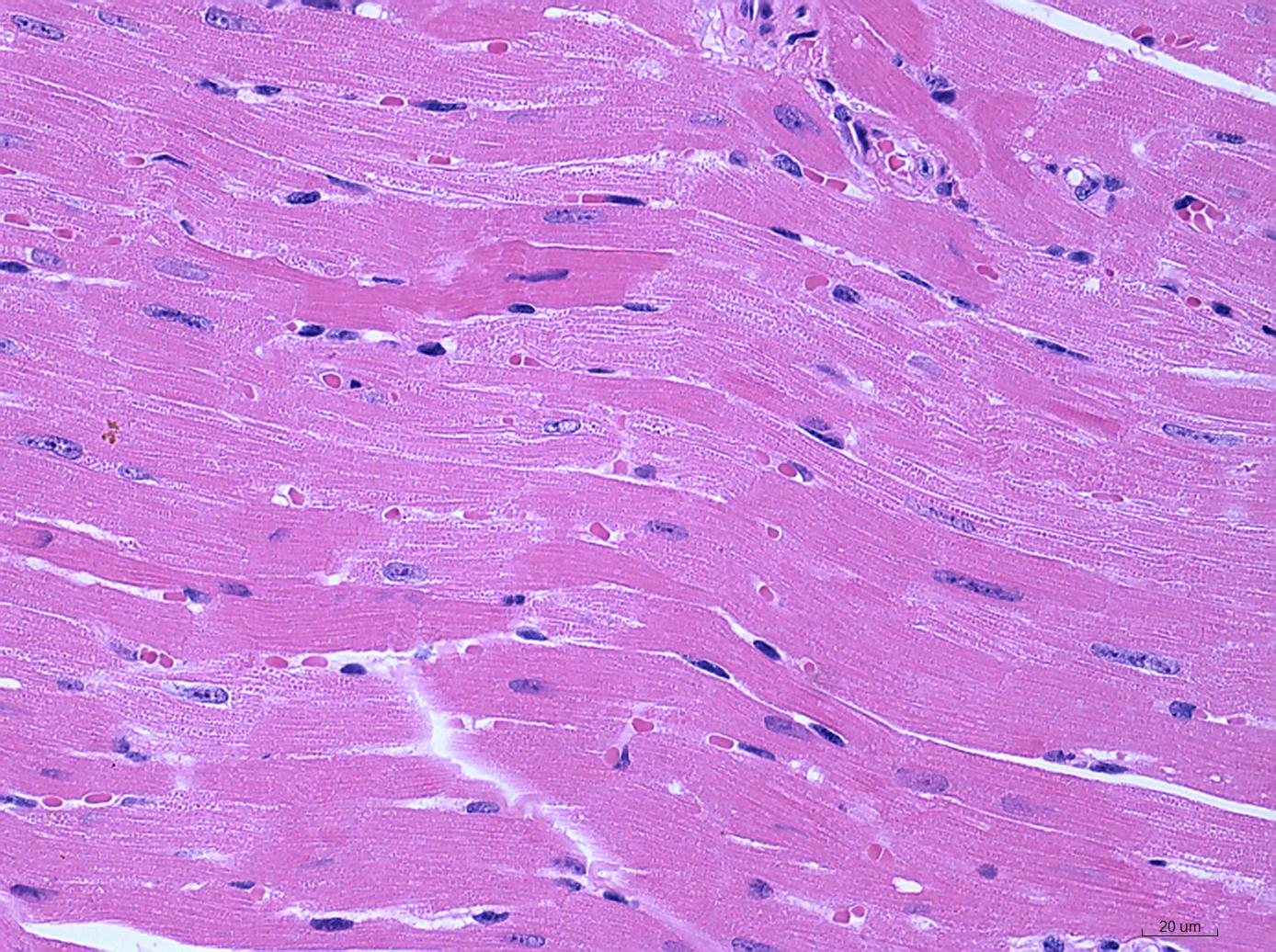

Supplement: Supplementary file 4 — Supporting File 4: advs73796‐sup‐0004‐Data.zip. [file ADVS-13-e21337-s003.zip › advs73796-sup-0004-Data/IHC_Raw_Data_Figures/Figure S4B_RawData_Figures/H&E-AAV9-cTnT- shTRIM40 + Ang II-40X.jpg]

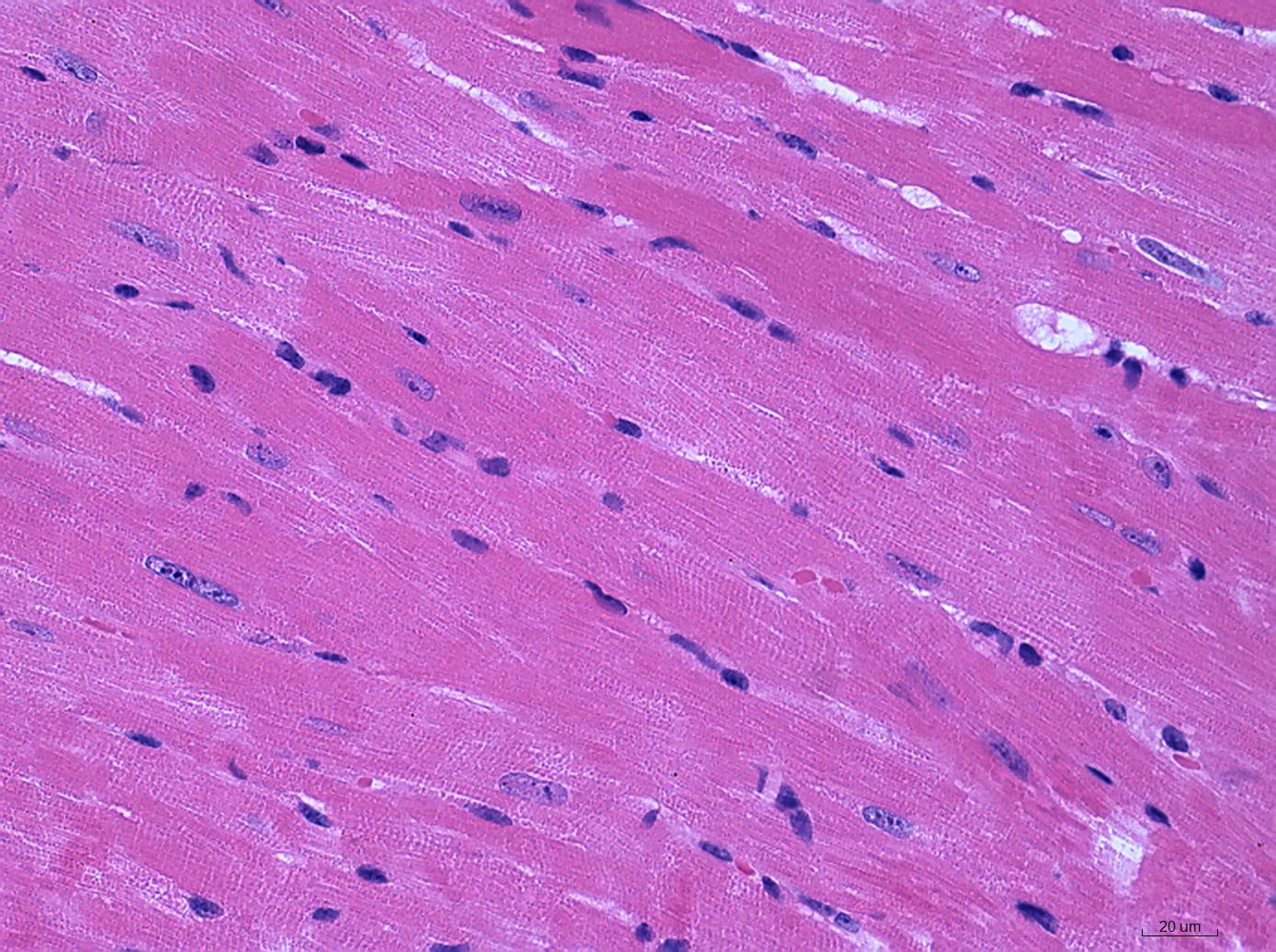

Supplement: Supplementary file 4 — Supporting File 4: advs73796‐sup‐0004‐Data.zip. [file ADVS-13-e21337-s003.zip › advs73796-sup-0004-Data/IHC_Raw_Data_Figures/Figure S4B_RawData_Figures/H&E-AAV9-cTnT- shTRIM40 + Sham-40X.jpg]

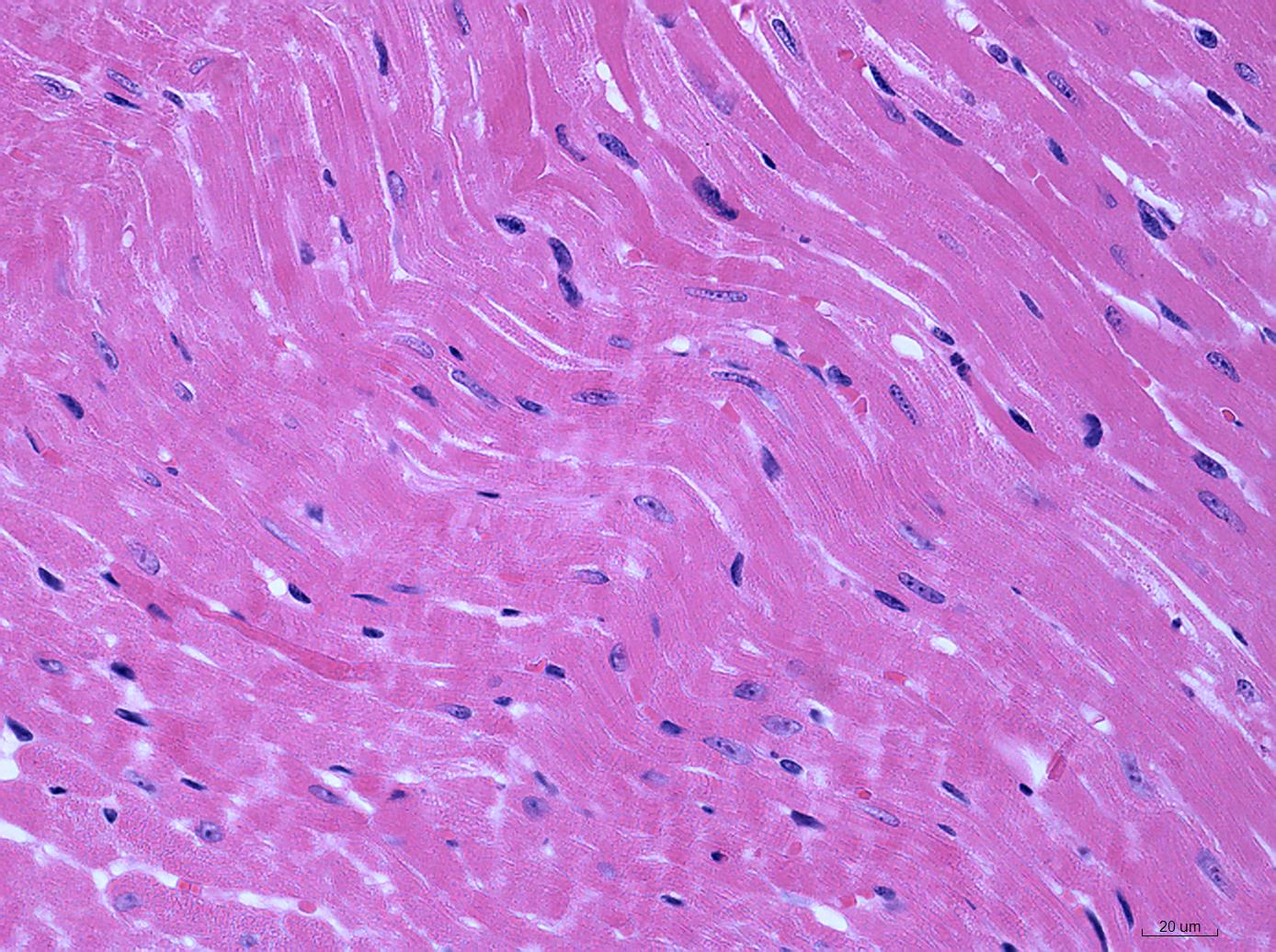

Supplement: Supplementary file 4 — Supporting File 4: advs73796‐sup‐0004‐Data.zip. [file ADVS-13-e21337-s003.zip › advs73796-sup-0004-Data/IHC_Raw_Data_Figures/Figure S4B_RawData_Figures/H&E-AAV9-cTnT-NC + AngII-40X.jpg]

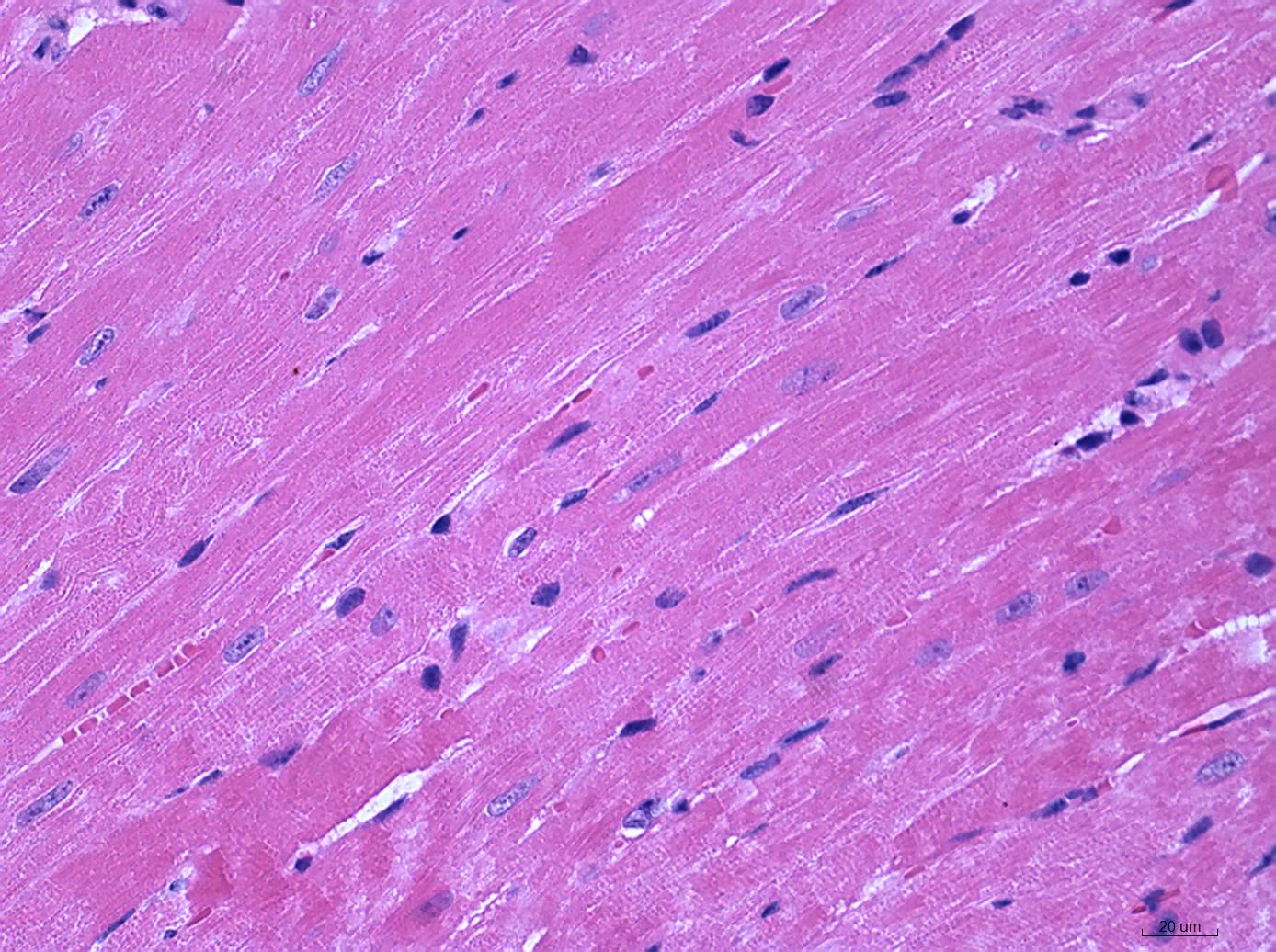

Supplement: Supplementary file 4 — Supporting File 4: advs73796‐sup‐0004‐Data.zip. [file ADVS-13-e21337-s003.zip › advs73796-sup-0004-Data/IHC_Raw_Data_Figures/Figure S4B_RawData_Figures/H&E-AAV9-cTnT-NC + Sham-40X.jpg]

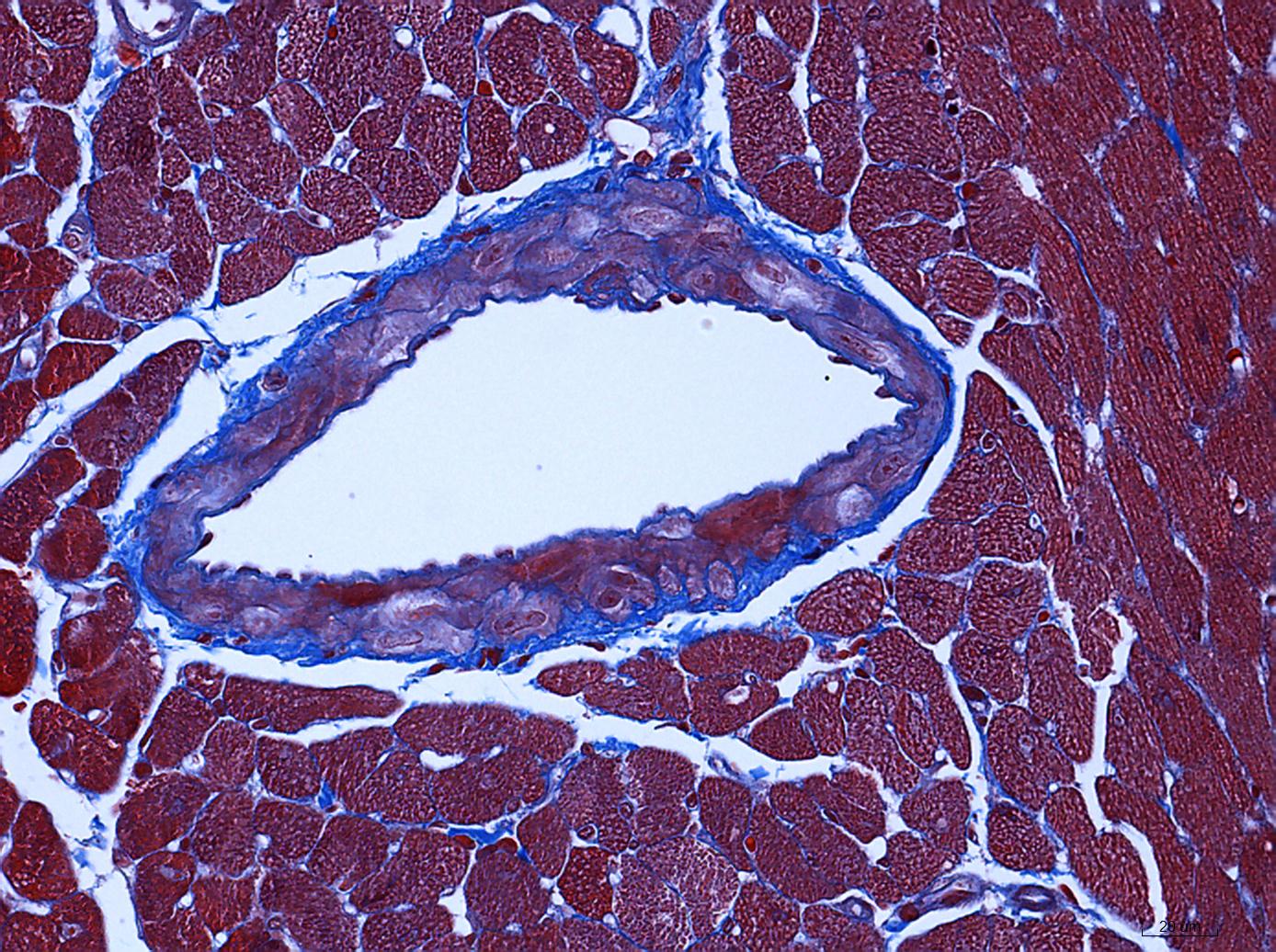

Supplement: Supplementary file 4 — Supporting File 4: advs73796‐sup‐0004‐Data.zip. [file ADVS-13-e21337-s003.zip › advs73796-sup-0004-Data/IHC_Raw_Data_Figures/Figure S4D_RawData_Figures/Masson-AAV9-cTnT- shTRIM40 + Ang II-40X.jpg]
