# Supplementary material for: TRIM40 Drives Pathological Cardiac Hypertrophy and Heart Failure via Ubiquitination of PKN2
Source: Adv Sci (Weinh). 2026 Jan 22;13(17):e21337. doi: 10.1002/advs.202521337 (PMC13042792; doi:10.1002/advs.202521337)

Figure 2K

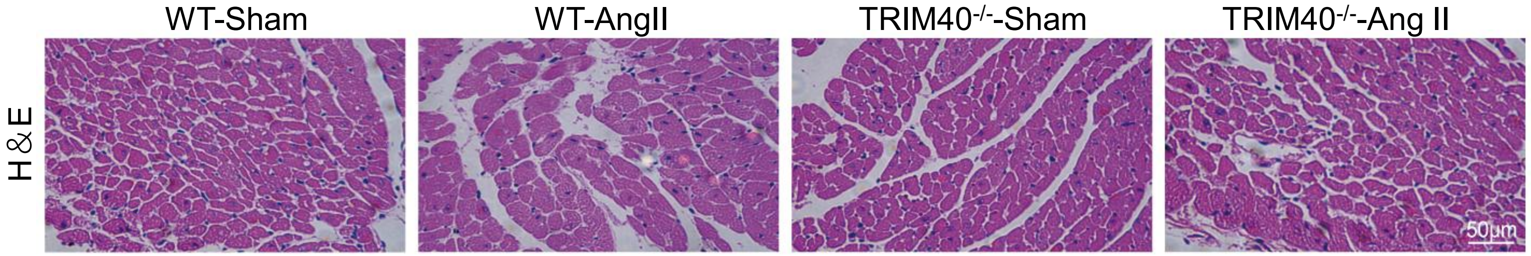


Figure 2L

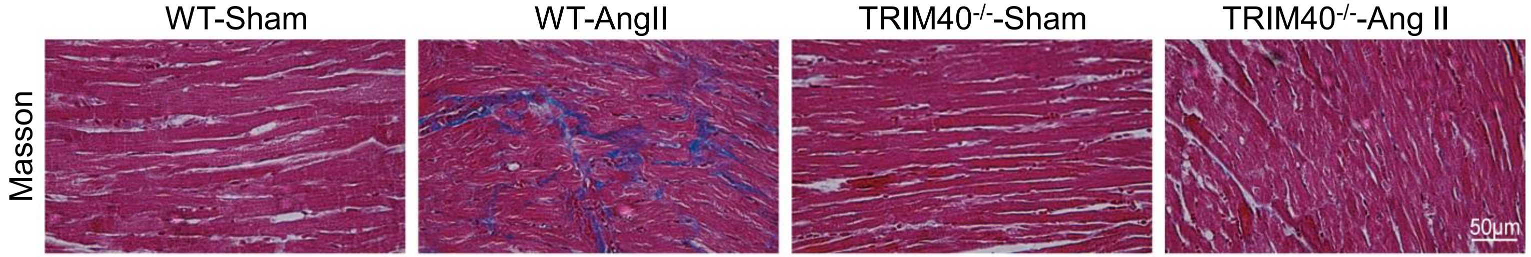


Figure 2M

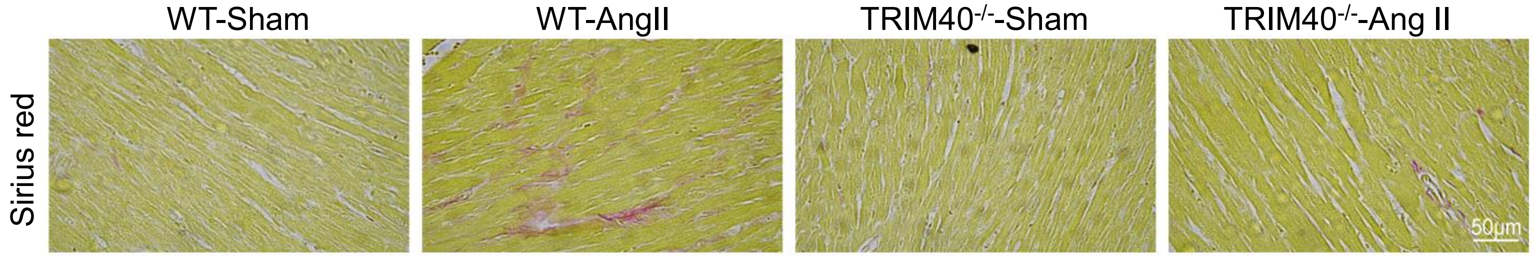

Figure 3J

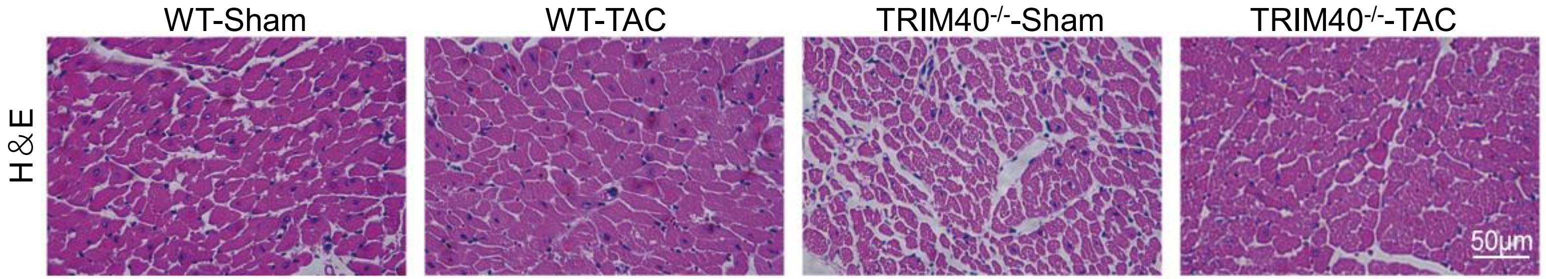


Figure 3K

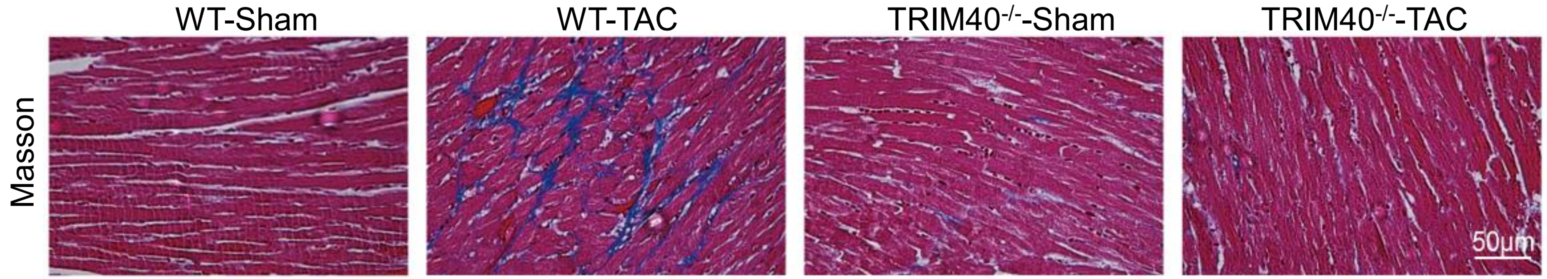


Figure 3L

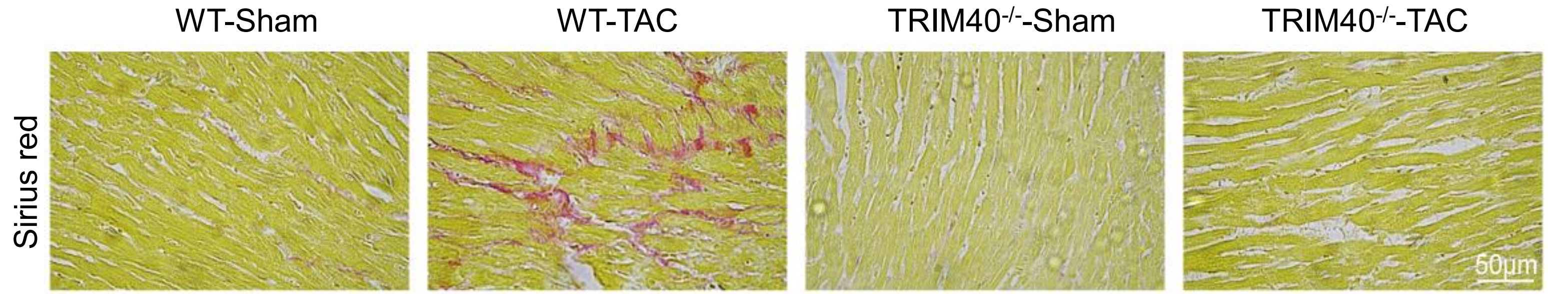

Figure 4M

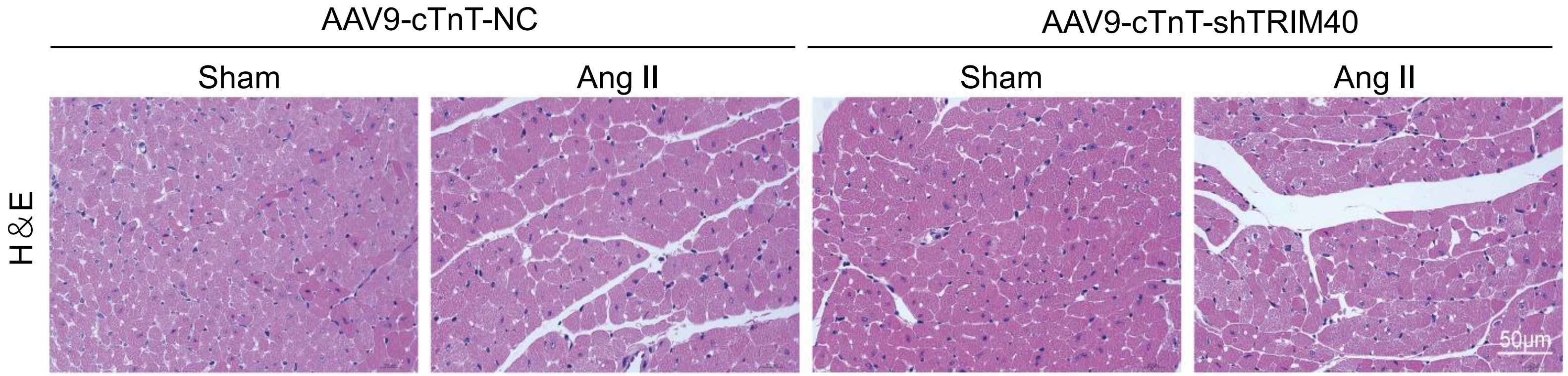


Figure 4N

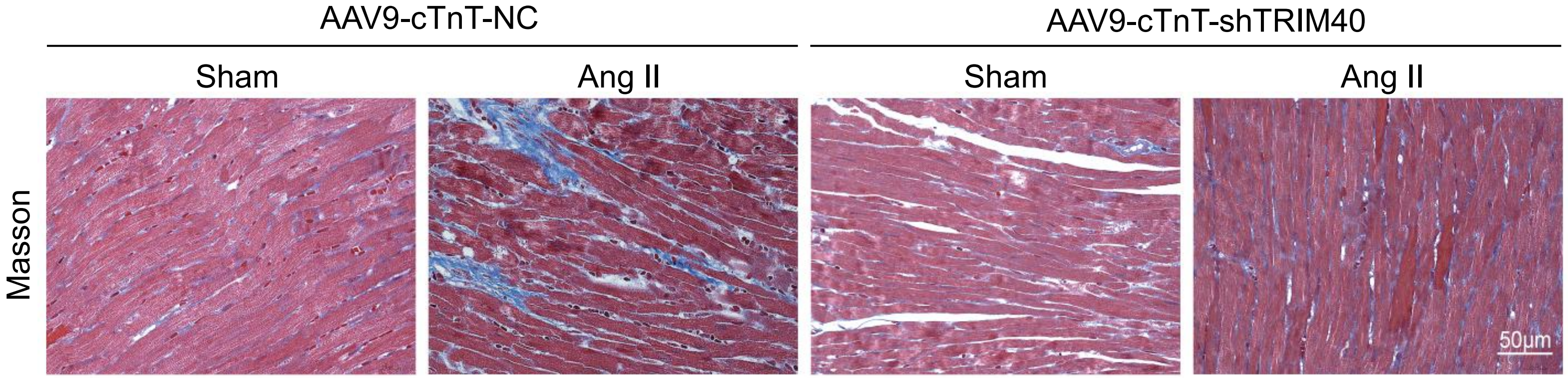


Figure 4O

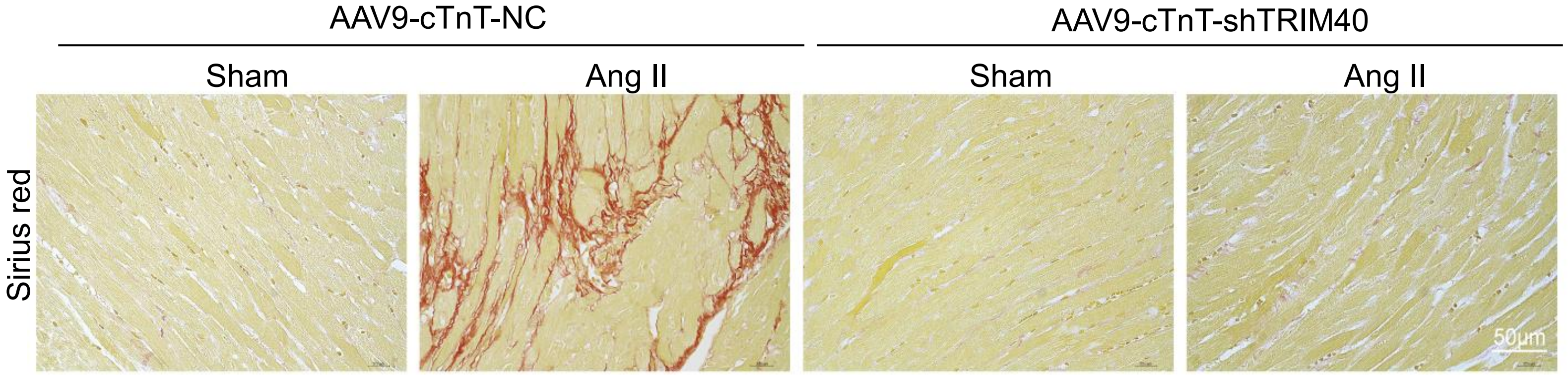

Figure 8K

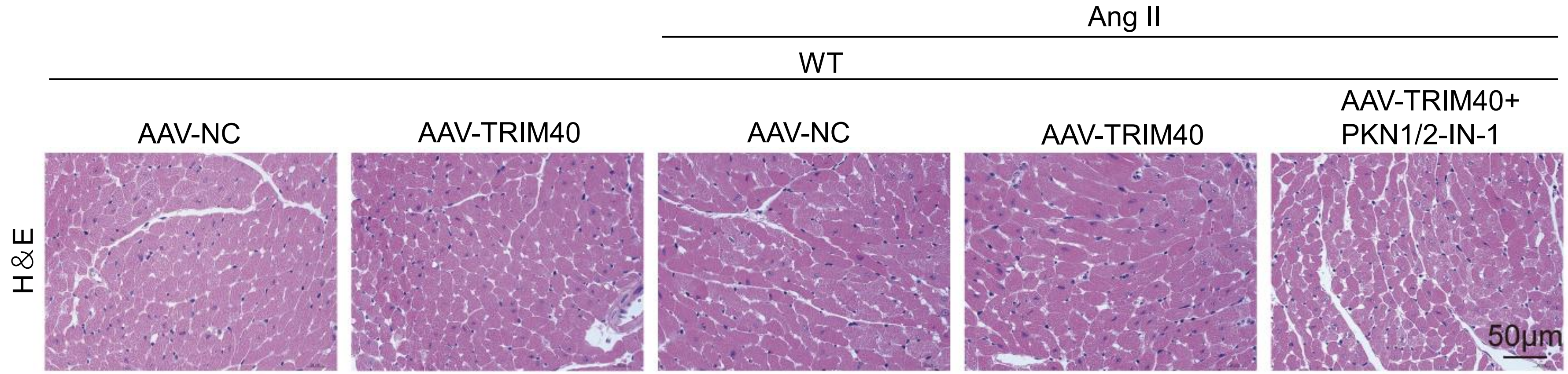


Figure 8L

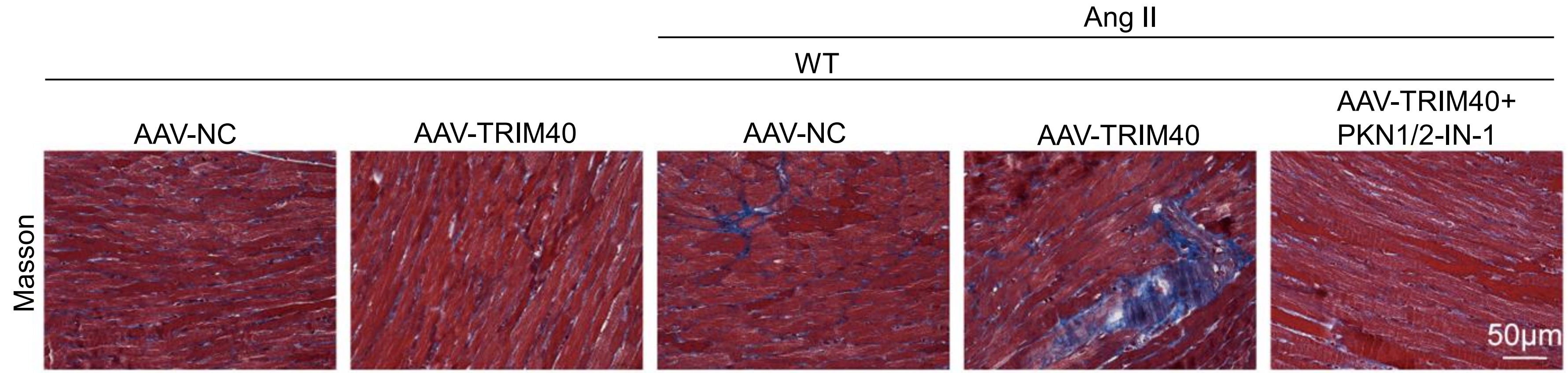


Figure 8M

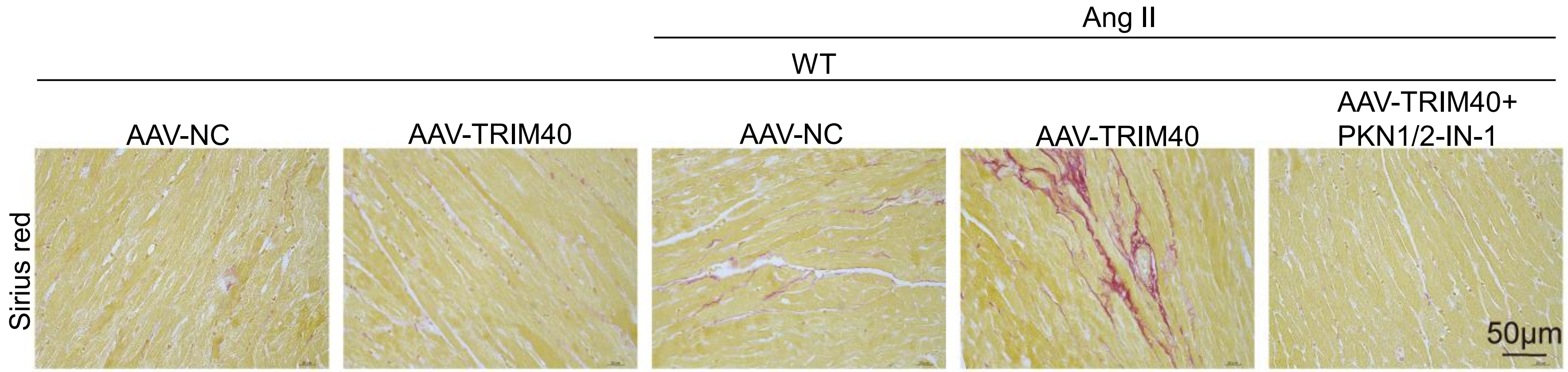

Figure 9J

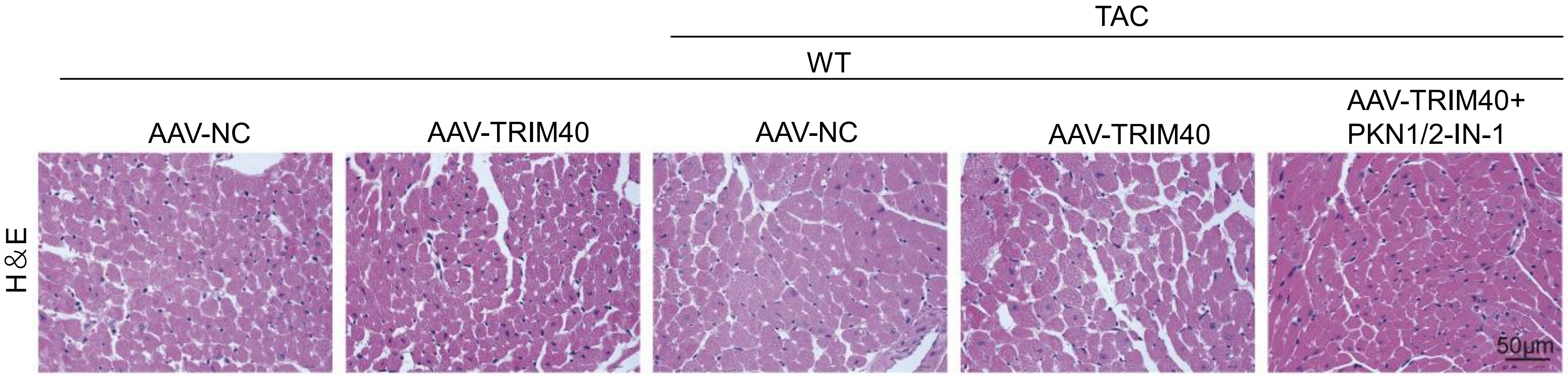


Figure 9K

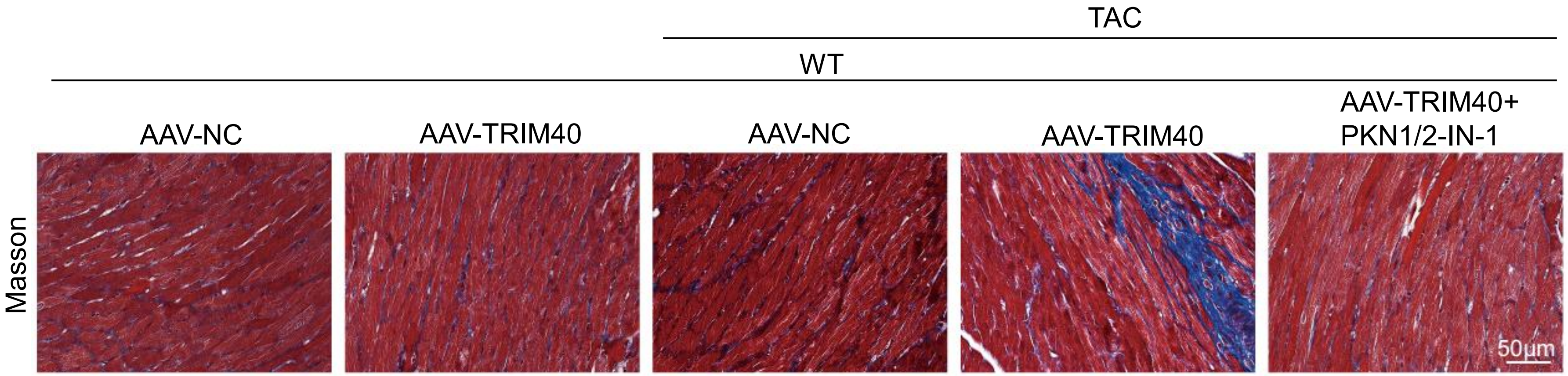


Figure 9L

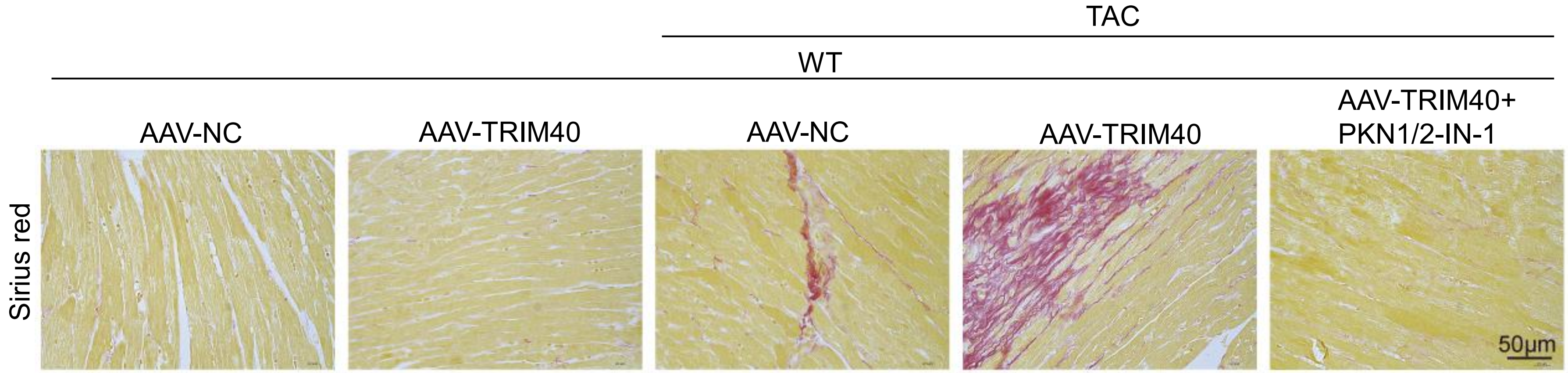

Figure S2E

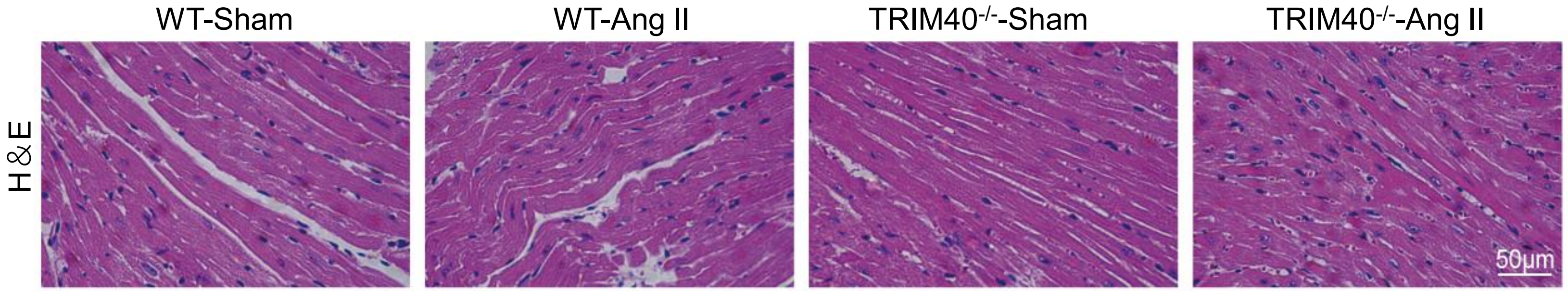


Figure S2G

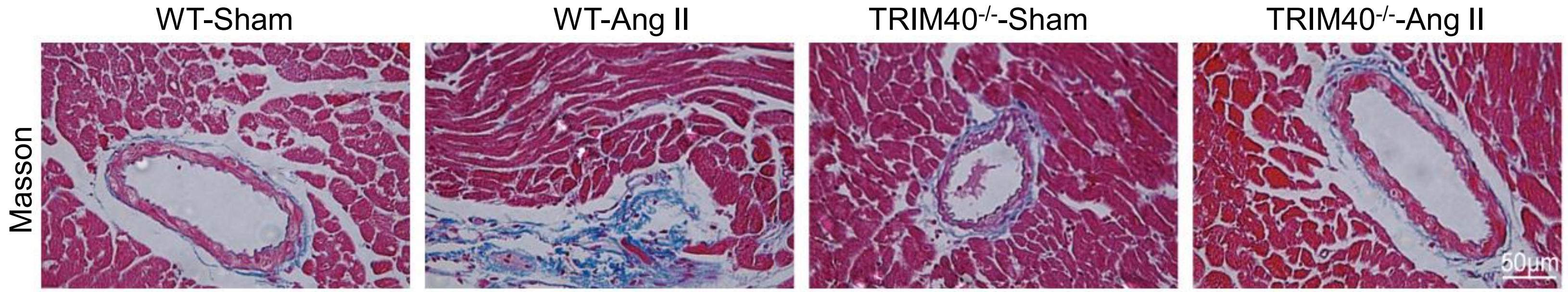


Figure S2J

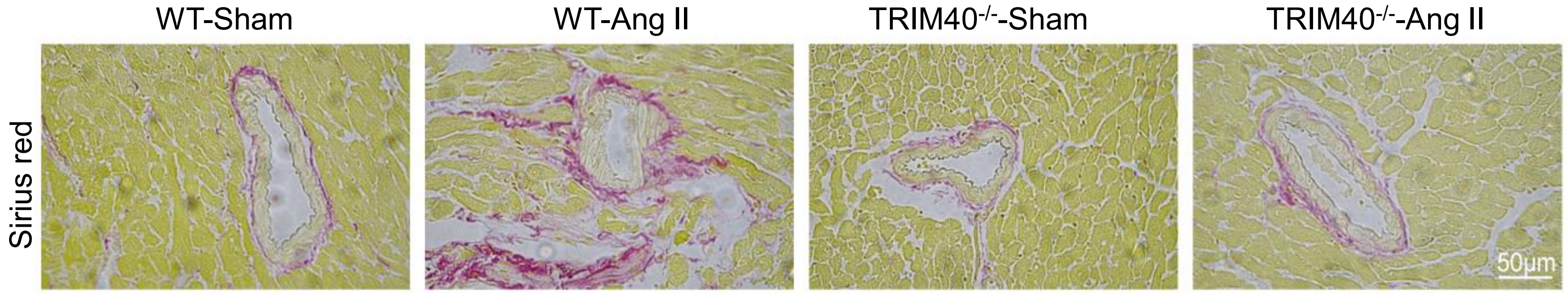

Figure S3C

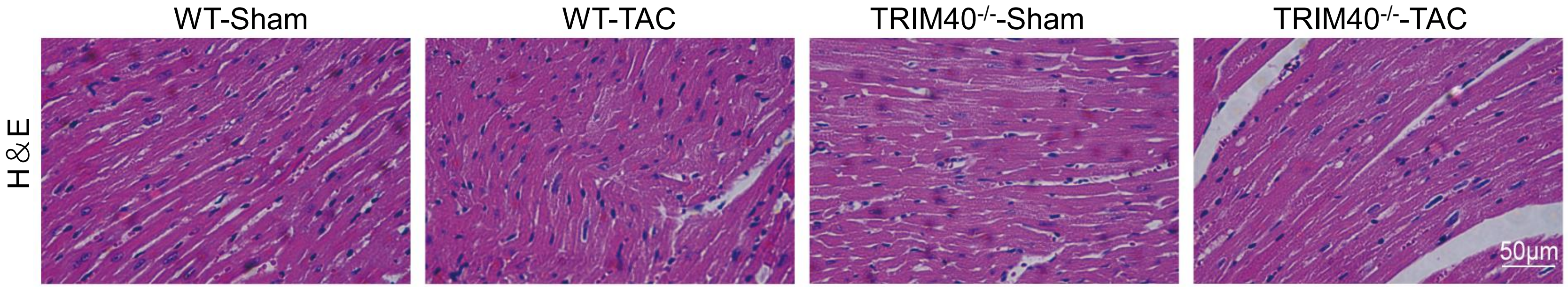


Figure S3E

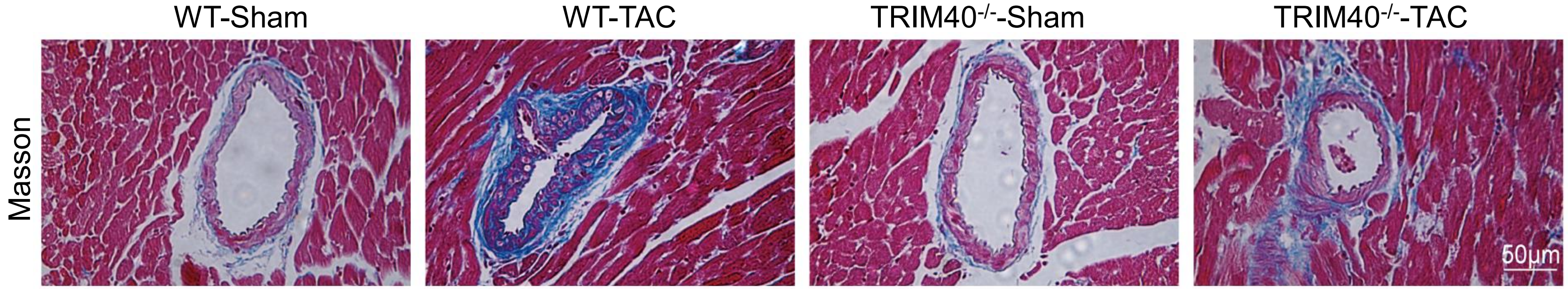


Figure S3H

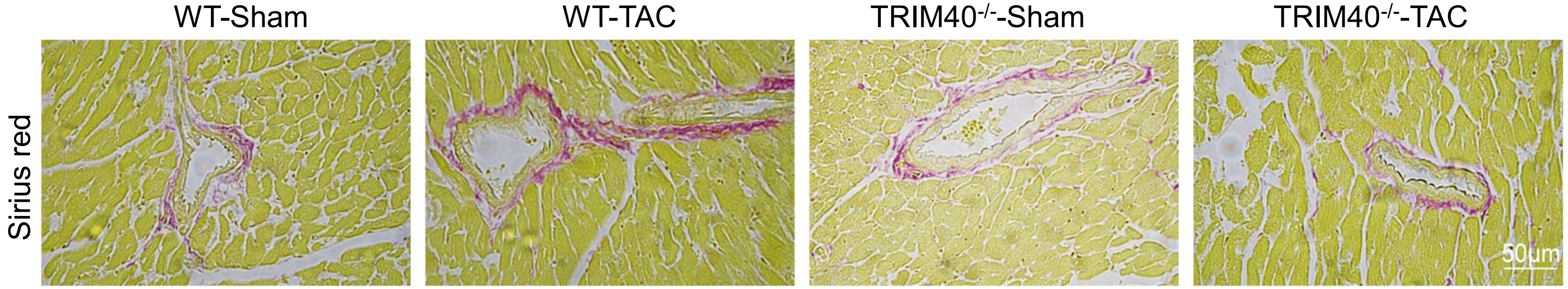

Figure S4B

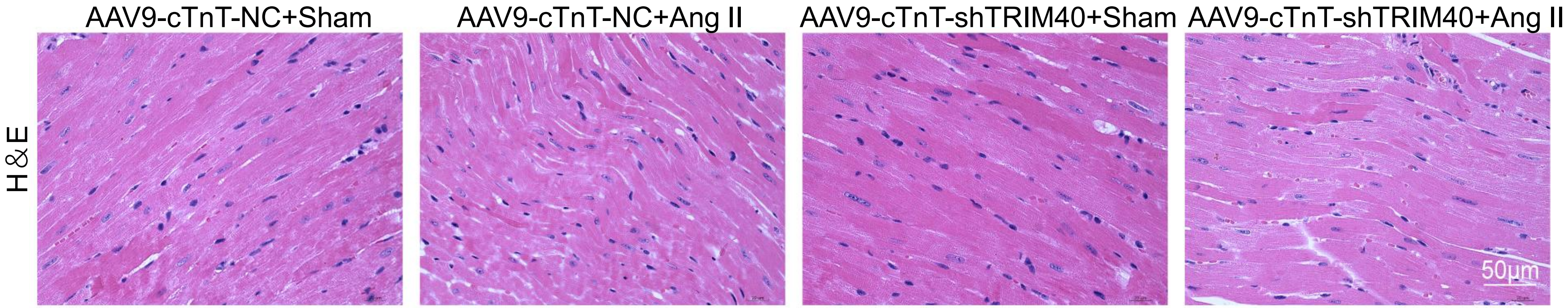


Figure S4D

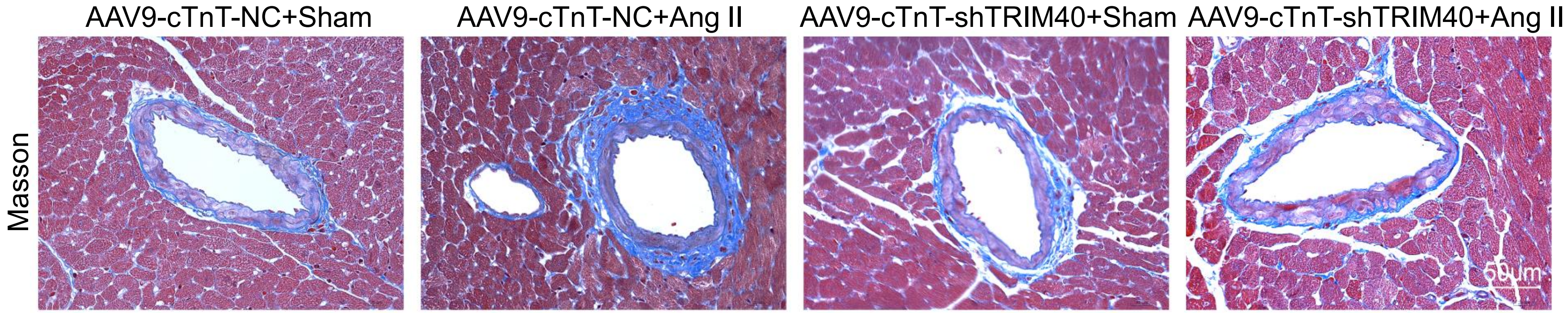


Figure S4D

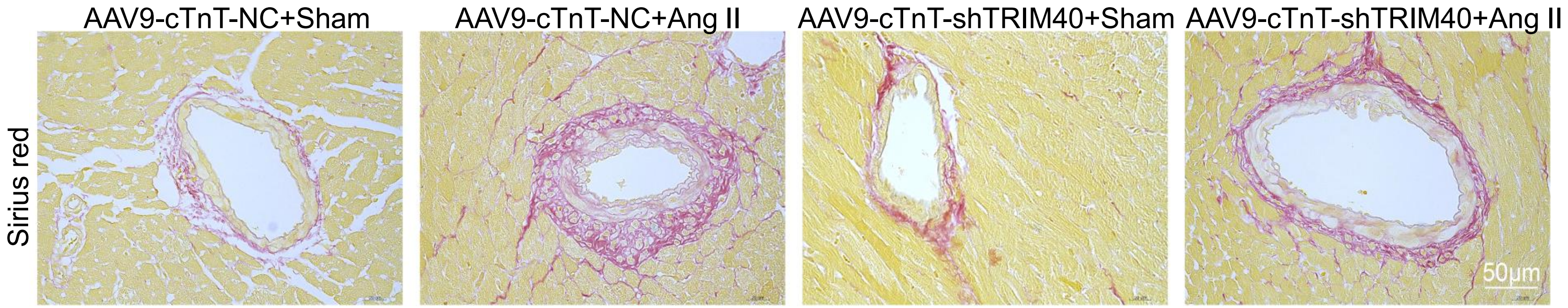

Figure S8F

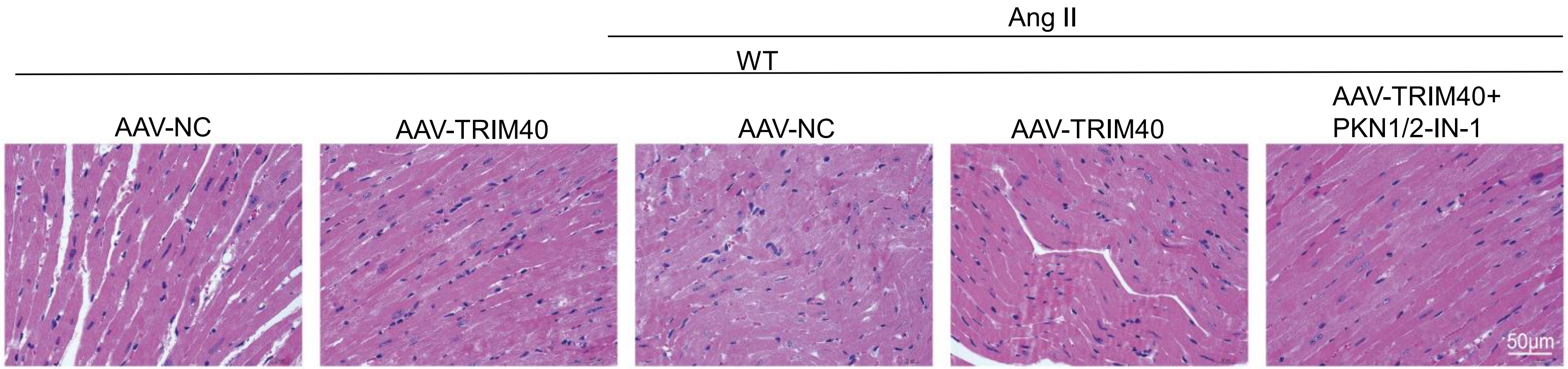


Figure S8H

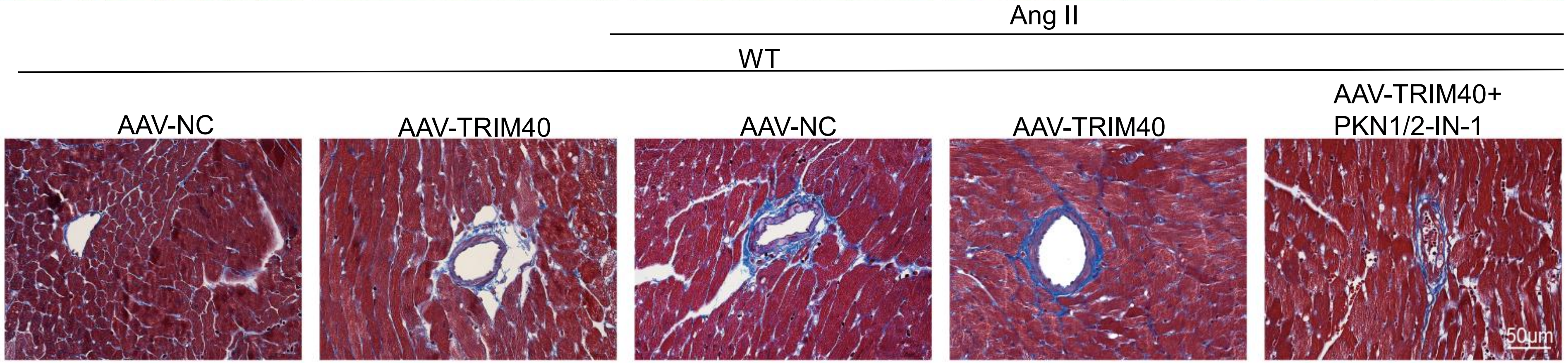


Figure S8K

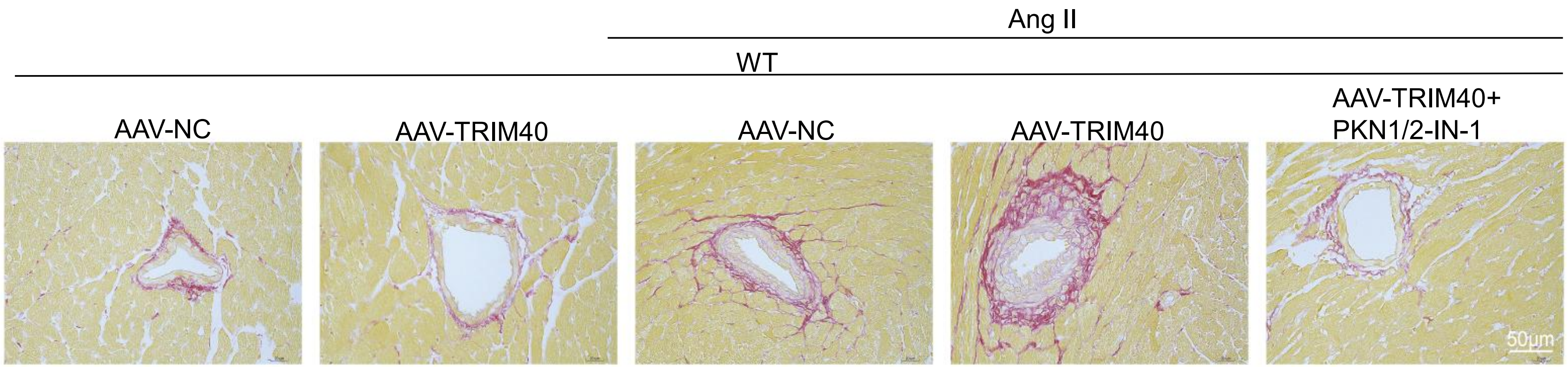

Figure S9C

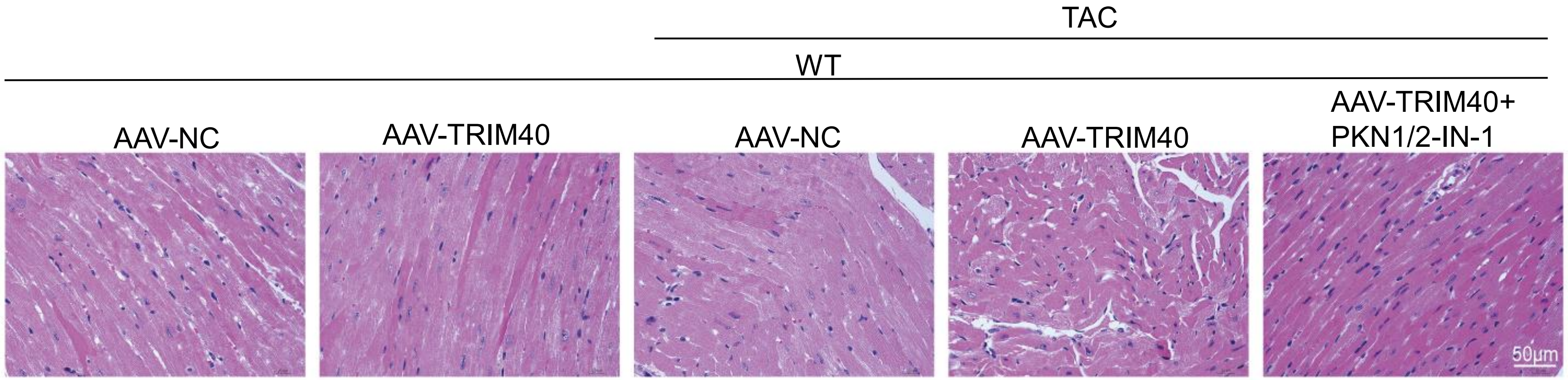


Figure S9E

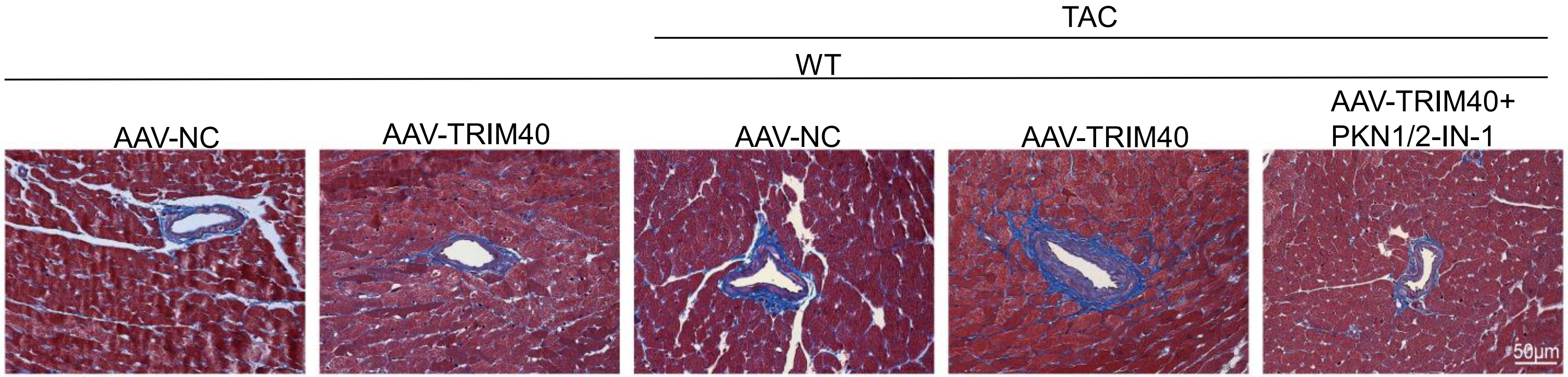


Figure S9H

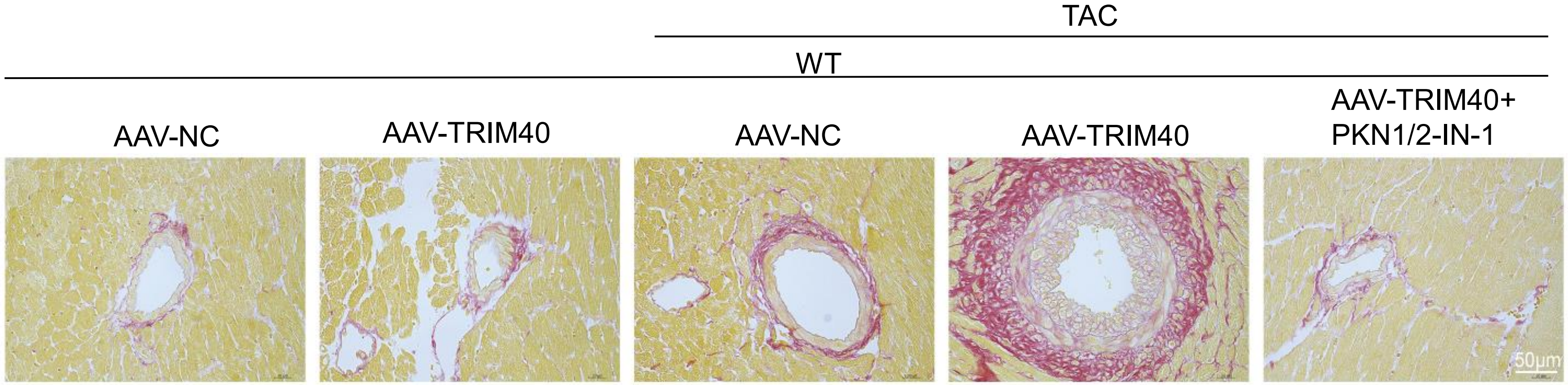

Supplement: Supplementary file 4 — Supporting File 4: advs73796‐sup‐0004‐Data.zip. [file ADVS-13-e21337-s003.zip › advs73796-sup-0004-Data/IHC_Raw_Data_Figures/IHC_Raw_Data_Figures.pdf]
